# Supplementary material for: What does social cognition look like in everyday social functioning in Huntington’s disease? A protocol for a scoping review to explore and synthesise knowledge about social cognition alongside day-to-day social functioning of people with Huntington’s disease
Source: BMJ Open. 2023 Jul 14;13(7):e073655. doi: 10.1136/bmjopen-2023-073655 (PMC10351301; doi:10.1136/bmjopen-2023-073655)
Supplement: Supplementary data [file bmjopen-2023-073655supp001.pdf]

Search strategy

**Database**	**Stage**	**Keywords**	**Limits / other details**
Embase / Ovid Medline (R) / Psychinfo	1. The umbrella scoping review of pre-existing systematic reviews	Huntington* AND (Social cogniti* OR Social function OR Social behaviour OR Social interaction OR Social judgement OR Social psychology OR Social understanding OR Social skills OR Social competence OR Social communication OR Social decision making OR Social withdrawal OR avoidance OR disengage)	Humans / English Language / Review articles / 2003 to current (June 2023)<br><br>Reference lists of articles which are clear **review articles** will be scanned for additional reviews related to the key words and these included in the scope if they meet the inclusion criteria
Web of Science	Stage 1 – as above	Huntingtons* Social* Cog*	Limits as previously applied
Pubmed	Stage 1 – as above	Huntington* AND social AND cognition	Defaulted to 2004 to current day<br><br>No other limits applied but searching for titles/abstracts with clear review article hallmarks
Scopus	Stage 1 – as above	Huntington* AND social AND cognition	Limits as previously applied + review articles only
Cinahl	Stage 1 – as above	Social cognition AND Huntingtons disease	Limits as previously applied
Embase / Ovid Medline (R) / Psychinfo	2. A further (non-umbrella) scoping review of quantitative and mixed methods literature on social cognition, social behaviour, and social functioning to the current date	Huntington* AND (Social cogniti* OR Social function OR Social behaviour OR Social interaction OR Social judgement OR Social psychology OR Social understanding OR Social skills OR Social competence OR Social communication	Humans / English Language / 2003 to current (June 2023)<br><br>Due to there being a maximum of key words (which differs in each database), separate searches will be undertaken for different concepts i.e. social cognitive processes such as

		OR Social decision making OR<br>Social withdrawal OR avoidance OR disengage)	emotional recognition, or social functioning which will include work<br><br>These will be then be combined if the parameters of the database allow
Embase / Ovid Medline (R) / Psychinfo<br><br>Web of Science<br><br>Pubmed<br><br>Scopus<br><br>Cinahl	**2.** A further (non-umbrella) scoping review of quantitative and mixed methods literature on social cognition, social behaviour, and social functioning	Huntington* AND Non-verbal communication<br><br>Huntington* AND Body language<br><br>Huntington* AND Facial expression<br><br>Huntington* AND Recognition<br><br>Huntington* AND (Theory of mind OR TOM OR simulation theory)<br><br>Huntington* AND (Empath* OR empath* communication)<br><br>Huntington* AND (Mentalizing OR mental state)<br><br>Huntington* AND Emotion*<br><br>Huntington* AND (Inference OR Intuition)	Reflecting social cognitive processes
Embase / Ovid Medline (R) / Psychinfo<br><br>Web of Science<br><br>Pubmed	**2.** A further (non-umbrella) scoping review of quantitative and mixed methods literature on social cognition, social behaviour, and social functioning	Huntington* AND (Relationships OR interpersonal relationships)<br>Huntington* AND (Social networks OR social connections)	Reflecting social behaviour and functioning

Scopus<br><br>Cinahl		Huntington* AND Work<br><br>Huntington* AND Sex*<br><br>Huntington* AND (Function OR skills)	
Embase / Ovid Medline (R) / Psychinfo<br><br>Web of Science<br><br>Pubmed<br><br>Scopus<br><br>Cinahl	**3.** A scoping review of the qualitative literature concerning social cognition, social behaviour and social functioning.	Huntington* AND Non-verbal communication<br><br>Huntington* AND Body language<br><br>Huntington* AND Facial expression<br><br>Huntington* AND Recognition<br><br>Huntington* AND (Theory of mind OR TOM OR simulation theory)<br><br>Huntington* AND (Empath* OR empath* communication)<br><br>Huntington* AND (Mentalizing OR mental state)<br><br>Huntington* AND Emotion*<br><br>Huntington* AND (Inference OR Intuition)	Reflecting social cognitive processes
Embase / Ovid Medline (R) / Psychinfo<br><br>Web of Science<br><br>Pubmed	**3.** A scoping review of the qualitative literature concerning social cognition, social behaviour and social functioning.	Huntington* AND (Relationships OR interpersonal relationships)<br><br>Huntington* AND (Social networks OR social connections)	Reflecting social behaviour and functioning

Scopus<br><br>Cinahl		Huntington* AND Work<br><br>Huntington* AND Sex*<br><br>Huntington* AND (Function OR skills)	
Open Grey (EU and UK version)	**4.** A grey literature scope	Huntingtons disease	Neither databases allow for limits
Google Scholar	A grey literature scope	Huntington* AND (Relationships OR interpersonal relationships)<br><br>Huntington* AND (Social networks OR social connections)<br><br>Huntington* AND Work<br><br>Huntington* AND Sex*<br><br>Huntington* AND (Function OR skills)	Limits 2003 – 2023
Researchgate	A grey literature scope	Huntingtons disease	Author to scan first 25 pages on results for all literature relevant to the concepts of social cognition OR social functioning

Stage 5 - Please apply to the author for the search details of the message board as per the protocol published
