# Supplementary material for: What does social cognition look like in everyday social functioning in Huntington’s disease? A protocol for a scoping review to explore and synthesise knowledge about social cognition alongside day-to-day social functioning of people with Huntington’s disease
Source: BMJ Open. 2023 Jul 14;13(7):e073655. doi: 10.1136/bmjopen-2023-073655 (PMC10351301; doi:10.1136/bmjopen-2023-073655)
Supplement: Author's manuscript [file bmjopen-2023-073655.draft_revisions.pdf]

BMJ Open is committed to open peer review. As part of this commitment we make the peer review history of every article we publish publicly available.

When an article is published we post the peer reviewers' comments and the authors' responses online. We also post the versions of the paper that were used during peer review. These are the versions that the peer review comments apply to.

The versions of the paper that follow are the versions that were submitted during the peer review process. They are not the versions of record or the final published versions. They should not be cited or distributed as the published version of this manuscript.

BMJ Open is an open access journal and the full, final, typeset and author-corrected version of record of the manuscript is available on our site with no access controls, subscription charges or pay-per-view fees (http://bmjopen.bmj.com).

If you have any questions on BMJ Open's open peer review process please email info.bmjopen@bmj.com

BMJ Open

What does social cognition look like in everyday social functioning in Huntington's disease? - A protocol for a scoping review to explore and synthesise knowledge about social cognition alongside day-to-day social functioning of people with Huntington's disease

Journal:	*BMJ Open*
Manuscript ID	bmjopen-2023-073655
Article Type:	Protocol
Date Submitted by the Author:	13-Mar-2023

Complete List of Authors:	Fisher, Alexandra; University of Birmingham, Institute of Applied Health; Birmingham and Solihull Mental Health NHS Foundation Trust, West Midlands HD Service, Neuropsychiatry<br>Lavis, Anna; University of Birmingham, Institute of Applied Health Research<br>Greenfield, Sheila; University of Birmingham, Institute of Applied Health Research<br>Rickards, Hugh; University of Birmingham College of Medical and Dental Sciences, Clinical & Experimental Medicine; Birmingham and Solihull Mental Health NHS Foundation Trust, West Midlands HD Service, Neuropsychiatry

Keywords:	Social Cognition, Social Interaction, NEUROLOGY, Literature, PSYCHIATRY

SCHOLARONE™
Manuscripts

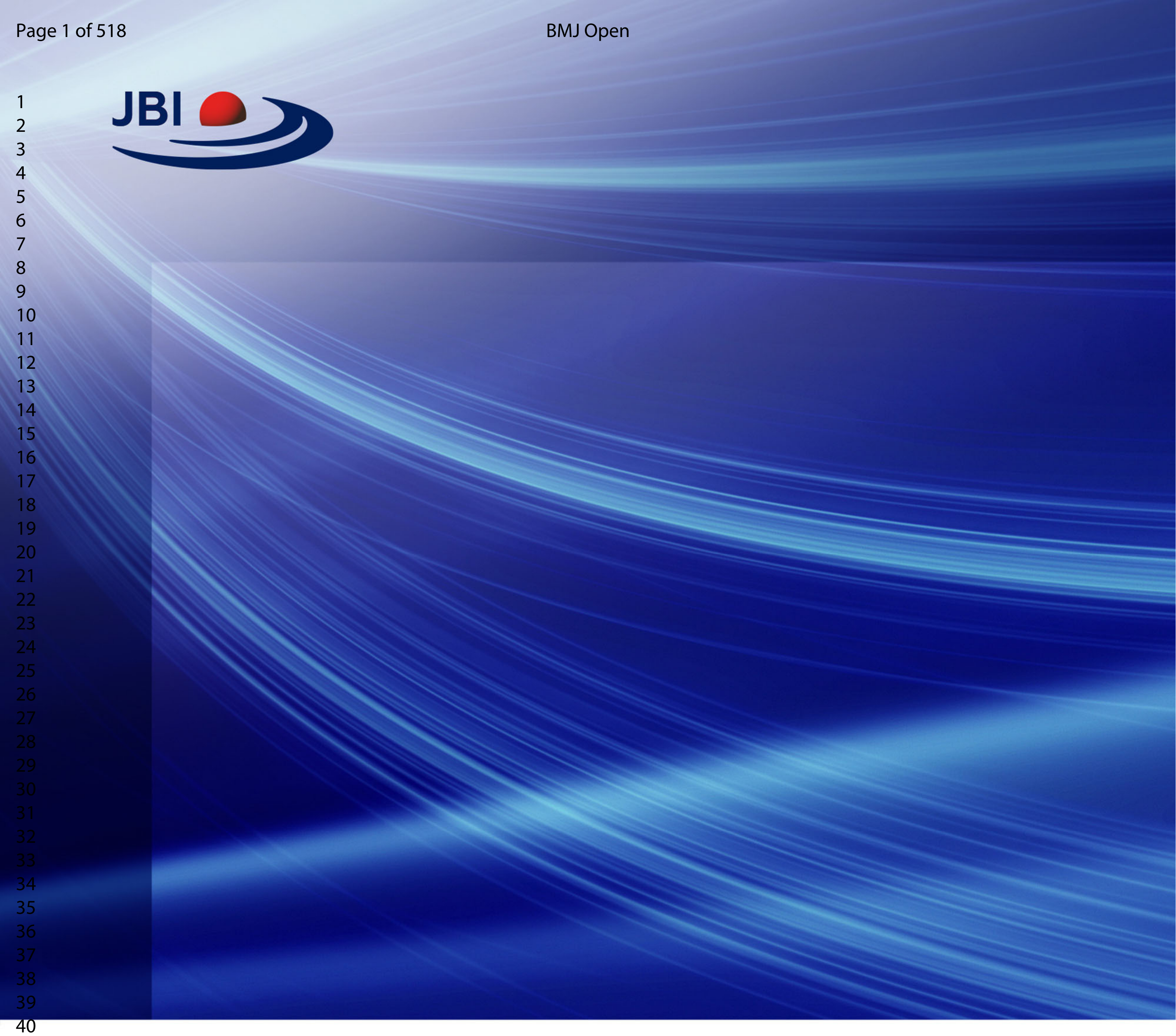

JBIMANUAL FOR EVIDENCE SYNTHESIS

April 2021

CRICOS Provider Number 00123M

1. JBI Manual for Evidence Synthesis . . . 6
1.1 About this Manual . . . 7
1.2 Contributors . . . 8
1.3 Chapter 1: JBI Systematic Reviews . . . 14
1.3.1 1.1 Introduction to JBI Systematic reviews . . . 15
1.3.2 1.2 Planning a JBI Review . . . 16
1.3.3 1.3 Registering a review title and protocol . . . 17
1.3.4 1.4 Publishing a JBI Systematic review . . . 18
1.3.5 1.5 Systematic review standards . . . 19
1.3.6 1.6 Disclosures and contributions . . . 20
1.3.7 1.7 Chapter References . . . 21
1.4 Chapter 2: Systematic reviews of qualitative evidence . . . 22
1.4.1 2.1 Introduction and purpose of this guidance . . . 23
1.4.2 2.2 Introduction to qualitative evidence and evidence-based healthcare . . . 24
1.4.3 2.3 Introduction to qualitative systematic reviews . . . 26
1.4.4 2.4 The JBI Approach to qualitative synthesis . . . 27
1.4.5 2.5 Core definitions in meta-aggregative reviews . . . 28
1.4.6 2.6 Developing a qualitative review protocol . . . 29
1.4.6.1 2.6.1 Title of a qualitative review protocol . . . 30
1.4.6.2 2.6.2 Review question . . . 31
1.4.6.3 2.6.3 Introduction . . . 32
1.4.6.4 2.6.4 Inclusion criteria . . . 33
1.4.6.4.1 2.6.4.1 Types of participants . . . 34
1.4.6.4.2 2.6.4.2 Phenomena of interest . . . 35
1.4.6.4.3 2.6.4.3 Context . . . 36
1.4.6.4.4 2.6.4.4 Types of studies . . . 37
1.4.6.4.5 2.6.4.5 Example inclusion criteria . . . 38
1.4.6.5 2.6.5 Search strategy . . . 39
1.4.6.6 2.6.6 Assessment of methodological quality . . . 41
1.4.6.7 2.6.7 Data extraction . . . 42
1.4.6.8 2.6.8 Data synthesis . . . 43
1.4.6.9 2.6.9 Conflicts of interest and acknowledgements . . . 44
1.4.7 2.7 Systematic review and synthesis of qualitative data . . . 45
1.4.7.1 2.7.1 Title . . . 46
1.4.7.2 2.7.2 Abstract . . . 47
1.4.7.3 2.7.3 ConQual 'Summary of Findings' . . . 48
1.4.7.4 2.7.4 Introduction . . . 50
1.4.7.5 2.7.5 Inclusion criteria . . . 51
1.4.7.6 2.7.6 Methods . . . 52
1.4.7.6.1 2.7.6.1 Search strategy . . . 53
1.4.7.6.2 2.7.6.2 Assessment of methodological quality . . . 54
1.4.7.6.3 2.7.6.3 Data extraction . . . 55
1.4.7.6.4 2.7.6.4 Data synthesis . . . 56
1.4.7.7 2.7.7 Results . . . 57
1.4.7.7.1 2.7.7.1 Study inclusion . . . 58
1.4.7.7.2 2.7.7.2 Methodological quality . . . 59
1.4.7.7.3 2.7.7.3 Characteristics of included studies . . . 60
1.4.7.7.4 2.7.7.4 Findings of the review . . . 61
1.4.7.8 2.7.8 Discussion . . . 62
1.4.7.9 2.7.9 Conclusions and Recommendations . . . 63
1.4.7.10 2.7.10 Conflicts and acknowledgements . . . 64
1.4.7.11 2.7.11 Review appendices . . . 65
1.4.8 2.8 Chapter references . . . 66
1.4.9 Appendix 2.1: JBI Critical Appraisal Checklist for Qualitative Research . . . 67
1.4.10 Appendix 2.2: Discussion of JBI Qualitative critical appraisal criteria . . . 68
1.4.11 Appendix 2.3: JBI Qualitative data extraction tool . . . 70
1.5 Chapter 3: Systematic reviews of effectiveness . . . 71
1.5.1 3.1 Introduction to quantitative evidence and evidence-based practice . . . 72
1.5.2 3.2 Development of a protocol for a systematic review of effectiveness evidence . . . 73
1.5.2.1 3.2.1 Title of the systematic review protocol . . . 74
1.5.2.2 3.2.2 Review question(s) . . . 75
1.5.2.3 3.2.3 Introduction . . . 76
1.5.2.4 3.2.4 Inclusion criteria . . . 77
1.5.2.4.1 3.2.4.1 Population (types of participants) . . . 78
1.5.2.4.2 3.2.4.2 Intervention (types of interventions) . . . 79
1.5.2.4.3 3.2.4.3 Comparison (types of comparators) . . . 80
1.5.2.4.4 3.2.4.4 Outcomes . . . 81
1.5.2.4.5 3.2.4.5 Types of studies . . . 82
1.5.2.5 3.2.5 Search strategy . . . 83
1.5.2.6 3.2.6 Selection of studies . . . 84
1.5.2.7 3.2.7 Critical appraisal . . . 85
1.5.2.8 3.2.8 Data extraction . . . 86
1.5.2.9 3.2.9 Data synthesis . . . 87
1.5.3 3.3 Meta-analysis . . . 88
1.5.3.1 3.3.1 Objectives of meta-analysis . . . 89
1.5.3.2 3.3.2 Statistical models for meta-analysis . . . 90
1.5.3.3 3.3.3 Effect sizes . . . 91
1.5.3.4 3.3.4 Considerations for the meta-analysis of dichotomous data . . . 92
1.5.3.5 3.3.5 Considerations for the meta-analysis of continuous data . . . 93

For peer review only

1.5.3.6 3.3.6 Meta-analysis: Statistical Methods . . . 94
1.5.3.7 3.3.7 Subgroups in meta-analysis . . . 95
1.5.3.8 3.3.8 Sensitivity analysis in meta-analysis . . . 96
1.5.3.9 3.3.9 Meta-regression . . . 97
1.5.3.10 3.3.10 Heterogeneity . . . 98
1.5.3.10.1 3.3.10.1 Standard chi-squared test (Cochran test) . . . 99
1.5.3.10.2 3.3.10.2 Quantification of the statistical heterogeneity: I squared . . . 100
1.5.3.10.3 3.3.10.3 Tau-squared for random effects model meta-analysis . . . 101
1.5.3.11 3.3.11 Publication bias . . . 102
1.5.4 3.4 Systematic review of effectiveness . . . 103
1.5.4.1 3.4.1 Title . . . 104
1.5.4.2 3.4.2 Abstract . . . 105
1.5.4.3 3.4.3 GRADE 'Summary of Findings' table . . . 106
1.5.4.4 3.4.4 Introduction . . . 107
1.5.4.5 3.4.5 Review question(s) . . . 108
1.5.4.6 3.4.6 Inclusion criteria . . . 109
1.5.4.7 3.4.7 Methods . . . 110
1.5.4.7.1 3.4.7.1 Search strategy . . . 111
1.5.4.7.2 3.4.7.2 Study screening and selection . . . 112
1.5.4.7.3 3.4.7.3 Critical appraisal . . . 113
1.5.4.7.4 3.4.7.4 Data extraction . . . 114
1.5.4.7.5 3.4.7.5 Data synthesis . . . 115
1.5.4.8 3.4.8 Results . . . 116
1.5.4.8.1 3.4.8.1 Study inclusion . . . 117
1.5.4.8.2 3.4.8.2 Methodological quality . . . 118
1.5.4.8.3 3.4.8.3 Characteristics of included studies . . . 119
1.5.4.8.4 3.4.8.4 Results and meta-analysis . . . 120
1.5.4.9 3.4.9 Discussion . . . 121
1.5.4.10 3.4.10 Conclusions and recommendations . . . 122
1.5.4.11 3.4.11 Conflicts and acknowledgements . . . 123
1.5.4.12 3.4.12 Review Appendices . . . 124
1.5.5 3.5 Chapter References . . . 125
1.5.6 Appendix 3.1: JBI Critical appraisal checklist for randomized controlled trials . . . 127
1.5.7 Appendix 3.2: Discussion of JBI appraisal criteria for randomized controlled trials . . . 128
1.5.8 Appendix 3.3: JBI Critical appraisal Checklist for Quasi-Experimental Studies (non-randomized experimental studies) . . . 131
1.5.9 Appendix 3.4: Discussion of JBI appraisal criteria for Quasi-Experimental Studies (non-randomized experimental studies) 132
1.6 Chapter 4: Systematic reviews of text and opinion . . . 134
1.6.1 4.1 Text and opinion reviews and evidence-based practice . . . 135
1.6.1.1 4.1.1 Evidence, the practice gap and re-consideration of textual evidence . . . 136
1.6.1.2 4.1.2 The synthesis of text and opinion . . . 137
1.6.2 4.2 Protocol development for reviews of textual, non-research evidence . . . 138
1.6.2.1 4.2.1 Title development and author information . . . 139
1.6.2.2 4.2.2 Protocol title . . . 140
1.6.2.3 4.2.3 Background . . . 141
1.6.2.4 4.2.4 Review objectives/questions . . . 142
1.6.2.5 4.2.5 Inclusion criteria . . . 143
1.6.2.6 4.2.6 Search strategy . . . 144
1.6.2.7 4.2.7 Assessment of methodological quality . . . 145
1.6.2.8 4.2.8 Textual data extraction . . . 146
1.6.2.9 4.2.9 Data synthesis . . . 147
1.6.2.10 4.2.10 References . . . 148
1.6.3 4.3 The systematic review and synthesis of text and opinion data . . . 149
1.6.3.1 4.3.1 Title of systematic review . . . 150
1.6.3.2 4.3.2 Review authors . . . 151
1.6.3.3 4.3.3 Executive summary . . . 152
1.6.3.4 4.3.4 Main body of the report . . . 153
1.6.3.4.1 4.3.4.1 Background . . . 154
1.6.3.4.2 4.3.4.2 Review objectives/questions . . . 155
1.6.3.4.3 4.3.4.3 Inclusion criteria . . . 156
1.6.3.4.4 4.3.4.4 Search strategy . . . 157
1.6.3.4.5 4.3.4.5 Method of the review . . . 158
1.6.3.4.6 4.3.4.6 Results . . . 163
1.6.3.4.7 4.3.4.7 Discussion . . . 167
1.6.3.4.8 4.3.4.8 Conclusions . . . 168
1.6.3.4.9 4.3.4.9 References . . . 169
1.6.3.5 4.3.5 Appendices . . . 170
1.6.4 4.4 Chapter references . . . 171
1.6.5 Appendix 4.1: JBI Critical Appraisal Checklist for Text and Opinion Papers . . . 172
1.6.6 Appendix 4.2: Explanation of Text and Expert Opinion critical appraisal tool . . . 173
1.6.7 Appendix 4.3: Textual data extraction form for text and opinion publications . . . 174
1.7 Chapter 5: Systematic reviews of prevalence and incidence . . . 175
1.7.1 5.1 Introduction to systematic reviews of prevalence and incidence . . . 176
1.7.2 5.2 Study designs reporting prevalence and incidence data . . . 177
1.7.3 5.3 Systematic reviews of prevalence and incidence . . . 178
1.7.3.1 5.3.1 Indications for systematic reviews of prevalence and incidence . . . 179
1.7.4 5.4 Developing a review protocol . . . 180
1.7.4.1 5.4.1 Protocol and review title . . . 181
1.7.4.2 5.4.2 Review question . . . 182
1.7.4.3 5.4.3 Inclusion criteria . . . 183

For peer review only

1.7.4.3.1 5.4.3.1 Types of participants (population) . . . 184
1.7.4.3.2 5.4.3.2 Condition . . . 185
1.7.4.3.3 5.4.3.3 Context . . . 186
1.7.4.3.4 5.4.3.4 Types of studies . . . 187
1.7.4.4 5.4.4 Search strategy . . . 188
1.7.4.5 5.4.5 Assessment of methodological quality . . . 189
1.7.4.6 5.4.6 Data extraction . . . 190
1.7.4.7 5.4.7 Data synthesis . . . 191
1.7.5 5.5 Conducting and reporting systematic reviews of prevalence and incidence data . . . 192
1.7.5.1 5.5.1 Executive summary . . . 193
1.7.5.2 5.5.2 Background . . . 194
1.7.5.3 5.5.3 Review questions . . . 195
1.7.5.4 5.5.4 Inclusion criteria . . . 196
1.7.5.5 5.5.5 Search strategy . . . 197
1.7.5.6 5.5.6 Assessment of methodological quality . . . 198
1.7.5.7 5.5.7 Data extraction . . . 199
1.7.5.8 5.5.8 Data synthesis and meta-analysis . . . 200
1.7.5.8.1 5.5.8.1 Narrative and non-statistical summary . . . 201
1.7.5.8.2 5.5.8.2 Meta-analysis . . . 202
1.7.5.9 5.5.9 Results . . . 206
1.7.5.10 5.5.10 Discussion . . . 208
1.7.5.11 5.5.11 Conclusions . . . 209
1.7.6 5.6 Chapter references . . . 210
1.7.7 Appendix 5.1: Critical Appraisal Instrument for Studies Reporting Prevalence Data . . . 212
1.7.8 Appendix 5.2: Data extraction form for prevalence studies . . . 215
1.8 Chapter 6: Systematic reviews of economic evidence . . . 216
1.9 Chapter 7: Systematic reviews of etiology and risk . . . 217
1.9.1 7.1 Introduction to etiological evidence and systematic reviews . . . 218
1.9.2 7.2 Study designs for etiology and risk . . . 219
1.9.2.1 7.2.1 Observational Study Designs . . . 220
1.9.2.1.1 7.2.1.1 Cohort Studies . . . 221
1.9.2.1.2 7.2.1.2 Case-control studies . . . 222
1.9.2.1.3 7.2.1.3 Cross-sectional studies (Analytical) . . . 223
1.9.2.2 7.2.2. Descriptive study designs . . . 224
1.9.3 7.3 The systematic review protocol and report . . . 225
1.9.3.1 7.3.1 Title of the systematic review . . . 226
1.9.3.2 7.3.2 Abstract . . . 227
1.9.3.3 7.3.3 Objective and review question . . . 228
1.9.3.4 7.3.4 Background . . . 229
1.9.3.5 7.3.5 Inclusion criteria . . . 230
1.9.3.5.1 7.3.5.1 Population (types of participants) . . . 231
1.9.3.5.2 7.3.5.2 Exposure of interest (Independent variable) . . . 232
1.9.3.5.3 7.3.5.3 Outcome (dependent variable) . . . 233
1.9.3.5.4 7.3.5.4 Types of studies . . . 234
1.9.3.6 7.3.6 Methods . . . 235
1.9.3.6.1 7.3.6.1 Search strategy . . . 236
1.9.3.6.2 7.3.6.2 Sources to search . . . 237
1.9.3.6.3 7.3.6.3 Assessment of methodological quality . . . 238
1.9.3.6.4 7.3.6.4 Data extraction . . . 241
1.9.3.6.5 7.3.6.5 Data synthesis . . . 242
1.9.3.7 7.3.7 Results . . . 246
1.9.3.7.1 7.3.7.1 Description of studies . . . 247
1.9.3.7.2 7.3.7.2 Methodological quality . . . 248
1.9.3.7.3 7.3.7.3 Findings of the review . . . 249
1.9.3.8 7.3.8 Discussion . . . 250
1.9.3.9 7.3.9 Conclusion and Recommendations . . . 251
1.9.3.10 7.3.10 Appendices . . . 252
1.9.4 7.4 Chapter references . . . 253
1.9.5 Appendix 7.1 Critical appraisal checklist for cohort studies . . . 255
1.9.6 Appendix 7.2 Critical appraisal checklist for case-control studies . . . 258
1.9.7 Appendix 7.3 Critical appraisal checklists for case series . . . 261
1.9.8 Appendix 7.4 Critical appraisal checklist for case reports . . . 265
1.9.9 Appendix 7.5 Critical appraisal checklist for analytical cross-sectional studies . . . 267
1.10 Chapter 8: Mixed methods systematic reviews . . . 270
1.10.1 8.1 Introduction to mixed methods systematic reviews . . . 271
1.10.2 8.2 Concepts and considerations for mixed methods systematic reviews . . . 272
1.10.3 8.3 The JBI approach to mixed method systematic reviews . . . 275
1.10.4 8.4 Developing a mixed methods review protocol . . . 280
1.10.4.1 8.4.1 MMSR questions that take a CONVERGENT INTEGRATED approach to synthesis and integration . . . 281
1.10.4.2 8.4.2 MMSR questions that take a CONVERGENT SEGREGATED approach to synthesis and integration . . . 286
1.10.5 8.5 Conducting and reporting a JBI MMSR . . . 291
1.10.5.1 8.5.1 Mixed methods systematic review using a CONVERGENT INTEGRATED approach to synthesis and integration . . . 292
1.10.5.2 8.5.2 Mixed methods systematic review using a CONVERGENT SEGREGATED approach to synthesis and integration . . . 298
1.10.6 Chapter References . . . 305
1.10.7 Appendix 8.1 JBI Mixed Methods Data Extraction Form following a Convergent Integrated Approach . . . 307
1.11 Chapter 9: Diagnostic test accuracy systematic reviews . . . 309
1.11.1 9.1 Background . . . 310

For peer review only

1.11.1.1 9.1.1 Measures of diagnostic test accuracy . . . 311
1.11.1.1.1 9.1.1.1 Sensitivity and specificity . . . 312
1.11.1.1.2 9.1.1.2 Predictive values . . . 313
1.11.1.1.3 9.1.1.3 Likelihood ratios . . . 314
1.11.1.1.4 9.1.1.4 ROC analyses . . . 315
1.11.2 9.2 Protocol and title development . . . 316
1.11.2.1 9.2.1Title . . . 317
1.11.2.2 9.2.2 Review question or objective . . . 318
1.11.2.3 9.2.3 Inclusion/exclusion criteria . . . 319
1.11.2.3.1 9.2.3.1 Population . . . 320
1.11.2.3.2 9.2.3.2 Index test . . . 321
1.11.2.3.3 9.2.3.3 Reference test . . . 322
1.11.2.3.4 9.2.3.4 Diagnosis of interest . . . 323
1.11.2.3.5 9.2.3.5 Types of studies . . . 324
1.11.2.4 9.2.4 Search strategy . . . 325
1.11.2.5 9.2.5 Assessment of methodological quality . . . 326
1.11.2.6 9.2.6 Data extraction . . . 329
1.11.2.7 9.2.7 Data synthesis . . . 330
1.11.2.7.1 9.2.7.1 Graphic representation . . . 331
1.11.2.7.2 9.2.7.2 Meta-analysis . . . 332
1.11.2.8 9.2.8 References . . . 336
1.11.3 9.3 Searching for studies of diagnostic test accuracy . . . 337
1.11.4 9.4 The systematic review of studies of diagnostic test accuracy . . . 340
1.11.4.1 9.4.1 Executive summary . . . 341
1.11.4.2 9.4.2 Summary of findings table . . . 342
1.11.4.3 9.4.3 Background . . . 344
1.11.4.4 9.4.4 Methods . . . 345
1.11.4.5 9.4.5 Results . . . 346
1.11.4.6 9.4.6 Discussion . . . 348
1.11.4.7 9.4.7 References . . . 349
1.11.4.8 9.4.8 Appendices . . . 350
1.11.5 9.5 Chaper references . . . 351
1.11.6 Appendix 9.1 Critical appraisal checklist . . . 353
1.11.7 Appendix 9.2 Data extraction instrument . . . 355
1.11.8 Appendix 9.3 Meta-analysis equations and models . . . 356
1.11.9 Appendix 9.4 Examples of databases . . . 358
1.12 Chapter 10: Umbrella reviews . . . 360
1.12.1 10.1 Umbrella reviews and evidence-based practice . . . 361
1.12.1.1 10.1.1 - Why an umbrella review? . . . 362
1.12.1.2 10.1.2 - Not just effectiveness - JBI umbrella reviews . . . 363
1.12.2 10.2 Development of an Umbrella review protocol . . . 364
1.12.2.1 10.2.1 Title and author information . . . 365
1.12.2.2 10.2.2 Developing the title and question . . . 366
1.12.2.3 10.2.3 Introduction . . . 367
1.12.2.4 10.2.4 Review question(s) . . . 368
1.12.2.5 10.2.5 Inclusion criteria . . . 369
1.12.2.6 10.2.6 Search Strategy . . . 370
1.12.2.7 10.2.7 Study Selection . . . 371
1.12.2.8 10.2.8 Assessment of methodological quality . . . 372
1.12.2.9 10.2.9 Data collection . . . 373
1.12.2.10 10.2.10 Data summary . . . 374
1.12.3 10.3 Umbrella Review and Summary of the evidence of research syntheses . . . 375
1.12.3.1 10.3.1 Title of the Umbrella Review . . . 376
1.12.3.2 10.3.2 Review Authors . . . 377
1.12.3.3 10.3.3 Abstract . . . 378
1.12.3.4 10.3.4 Introduction . . . 379
1.12.3.5 10.3.5 Review question(s) . . . 380
1.12.3.6 10.3.6 Inclusion criteria . . . 381
1.12.3.7 10.3.7 Methods . . . 382
1.12.3.7.1 10.3.7.1 Search strategy . . . 383
1.12.3.7.2 10.3.7.2 Study screening and selection . . . 384
1.12.3.7.3 10.3.7.3 Assessment of methodological quality/critical appraisal . . . 385
1.12.3.7.4 10.3.7.4 Data collection . . . 386
1.12.3.7.5 10.3.7.5 Data Summary . . . 388
1.12.3.8 10.3.8 Results . . . 389
1.12.3.8.1 10.3.8.1 Study inclusion . . . 390
1.12.3.8.2 10.3.8.2 Methodological quality . . . 391
1.12.3.8.3 10.3.8.3 Characteristics of included studies . . . 392
1.12.3.8.4 10.3.8.4 Findings of the review . . . 393
1.12.3.9 10.3.9 Summary of Evidence . . . 394
1.12.3.10 10.3.10 Discussion . . . 395
1.12.3.11 10.3.11 Conclusions and recommendations . . . 396
1.12.3.12 10.3.12 Conflicts and acknowledgements . . . 397
1.12.3.13 10.3.13 Review Appendices . . . 398
1.12.4 10.4 Chapter references . . . 399
1.12.5 Appendix 10.1 JBI Critical Appraisal Checklist for Systematic reviews and Research Syntheses . . . 400
1.12.6 Appendix 10.2. Discussion of JBI Critical Appraisal Checklist for systematic reviews and research syntheses . . . 403
1.12.7 Appendix 10.3 JBI Data Extraction Form for Review for Systematic Reviews and Research Syntheses . . . 405
1.13 Chapter 11: Scoping reviews . . . 406

For peer review only

1.13.1 11.1 Introduction to Scoping reviews . . . 407
1.13.1.1 11.1.1 Why a scoping review? . . . 408
1.13.1.2 11.1.2 Scoping reviews compared to other types of review . . . 410
1.13.1.3 11.1.3 The scoping review framework . . . 412
1.13.2 11.2 Development of a scoping review protocol . . . 413
1.13.2.1 11.2.1 Title . . . 414
1.13.2.2 11.2.2 Developing the title and question . . . 415
1.13.2.3 11.2.3 Introduction . . . 416
1.13.2.4 11.2.4 Inclusion criteria . . . 417
1.13.2.5 11.2.5 Search Strategy . . . 418
1.13.2.6 11.2.6 Source of evidence selection . . . 419
1.13.2.7 11.2.7 Data extraction . . . 420
1.13.2.8 11.2.8 Analysis of the evidence . . . 421
1.13.2.9 11.2.9 Presentation of the results . . . 422
1.13.3 11.3 The scoping review and summary of the evidence . . . 425
1.13.3.1 11.3.1 Title of the scoping review . . . 426
1.13.3.2 11.3.2 Review authors . . . 427
1.13.3.3 11.3.3 Abstract . . . 428
1.13.3.4 11.3.4 Introduction . . . 429
1.13.3.5 11.3.5 Review question(s) . . . 430
1.13.3.6 11.3.6 Inclusion Criteria . . . 431
1.13.3.7 11.3.7 Methods . . . 432
1.13.3.7.1 11.3.7.1 Search strategy . . . 433
1.13.3.7.2 11.3.7.2 Source of evidence screening and selection . . . 434
1.13.3.7.3 11.3.7.3 Data extraction . . . 435
1.13.3.7.4 11.3.7.4 Analysis and Presentation of results . . . 436
1.13.3.8 11.3.8 Results . . . 437
1.13.3.8.1 11.3.8.1 Search results . . . 438
1.13.3.8.2 11.3.8.2 Inclusion of sources of evidence . . . 439
1.13.3.8.3 11.3.8.3 Review findings . . . 440
1.13.3.9 11.3.9 Discussion . . . 441
1.13.3.10 11.3.10 Conclusions and recommendations . . . 442
1.13.3.11 11.3.11 Conflicts and acknowledgements . . . 443
1.13.3.12 11.3.12 References . . . 444
1.13.3.13 11.3.13 Review appendices . . . 445
1.13.4 11.4 Chapter references . . . 446
1.13.5 Appendix 11.1 JBI template source of evidence details, characteristics and results extraction instrument . . . 450
1.13.6 Appendix 11.2 PRISMA ScR Extension Fillable Checklist . . . 451
1.14 Chapter 12: Systematic reviews of measurement properties . . . 452
1.14.1 12.1 Measurement properties of instruments and evidence-based practice . . . 453
1.14.1.1 12.1.1 Rationale for a systematic review of measurement properties . . . 454
1.14.2 12.2 Protocol development . . . 455
1.14.2.1 12.2.1 Title . . . 456
1.14.2.2 12.2.2 Review question(s) . . . 457
1.14.2.3 12.2.3 Introduction . . . 458
1.14.2.4 12.2.4 Keywords . . . 459
1.14.2.5 12.2.5 Inclusion criteria . . . 460
1.14.2.6 12.2.6 Search strategy . . . 462
1.14.2.7 12.2.7 Study selection . . . 464
1.14.2.8 12.2.8 Assessment of methodological quality . . . 465
1.14.2.9 12.2.9 Data extraction . . . 466
1.14.2.10 12.2.10 Data synthesis . . . 467
1.14.2.11 12.2.11 Assessing certainty in the findings (i.e. GRADE approach) . . . 468
1.14.3 12.3 The systematic review . . . 469
1.14.3.1 12.3.1 Title . . . 470
1.14.3.2 12.3.2 Abstract . . . 471
1.14.3.3 12.3.3 Keywords . . . 472
1.14.3.4 12.3.4 Summary of findings . . . 473
1.14.3.5 12.3.5 Review question(s) . . . 474
1.14.3.6 12.3.6 Introduction . . . 475
1.14.3.7 12.3.7 Inclusion criteria . . . 476
1.14.3.8 12.3.8 Methods . . . 477
1.14.3.9 12.3.9 Results . . . 478
1.14.3.10 12.3.10 Discussion . . . 479
1.14.3.11 12.3.11 Conclusions . . . 480
1.14.4 12.4 Glossary of terms . . . 481
1.14.5 12.5 Chapter references . . . 483
1.14.6 Appendix 12.1 Data extraction / characteristics of included studies template . . . 485
1.14.7 Appendix 12.2 Table of results template . . . 486

For peer review only

JBI Manual for Evidence Synthesis

Welcome to the JBI Manual for Evidence Synthesis

JBI is an international evidence-based healthcare research organisation that works with 70+ Universities and Hospitals (known as the JBI Collaboration) around the world. The organisation focuses on improving health outcomes globally by producing and disseminating research evidence, software, training, resources and publications relating to evidence-based healthcare. Learn about the JBI approach to evidence-based healthcare.

We collaborate internationally with over 70 Collaborating Entities who subscribe to our definition of what constitutes evidence and our methodologies and methods in relation to evidence synthesis. JBI and its Collaborating Entities promote and support the synthesis, transfer and implementation of evidence through identifying feasible, appropriate, meaningful and effective healthcare practices to assist in the improvement of healthcare outcomes globally.

Our major role is the translation of research evidence into practice. One of our strengths is in the conduct of systematic reviews that reflect a broad, inclusive approach to evidence and accommodate a range of diverse questions and study designs.

The JBI Manual for Evidence Synthesis is designed to provide authors with a comprehensive guide to conducting JBI systematic reviews. It describes in detail the process of planning, undertaking and writing up a systematic review using JBI methods. The JBI Manual for Evidence Synthesis should be used in conjunction with the support and tutorials offered at the JBI SUMARI Knowledge Base.

We highly value the contribution of reviewers to the international body of literature used to inform clinical decision-making at the point of care. It is important that this work continues and is distributed in a variety of formats to both those working in and using health systems across the world. We hope that this work will contribute to improved global health outcomes.

How to cite

Aromataris E, Munn Z (Editors). *JBI Manual for Evidence Synthesis.* JBI, 2020. Available from https://synthesismanual.jbi.global. https://doi.org/10.46658/JBIMES-20-01

ISBN: 978-0-6488488-0-6

For peer review only

About this Manual

The JBI Manual for Evidence Synthesis provides guidance to authors for the conduct and preparation of JBI systematic reviews and evidence syntheses. The JBI Manual for Evidence Synthesis has separate chapters devoted synthesis of different types of evidence and to address different types of review questions.

The first three editions of the JBI Manual for Evidence Synthesis were published in book format; subsequent editions have been published online since 2017.

This manual is presented in an online wiki format to facilitate rapid inclusion of developments and updates to the JBI methodologies and methods for evidence synthesis that are presented. A .pdf version of the JBI Manual for Evidence Synthesis is also available here. The .pdf version is updated periodically (see date), however may not contain all of the latest revisions to the Manual. Users are advised to cross reference the relevant sections of the online manual during the conduct of their review.

Updates

This version of the JBI Manual for Evidence Synthesis includes changes that correspond to the latest methodological developments determined by the JBI Methodology Groups and JBI Scientific Committee, the latest developments with the JBI SUMARI software and also feedback by users.

To stay up to date with methods appropriate for JBI systematic reviews, it is recommended potential reviewer's attend the JBI Comprehensive Systematic Review Training Program.

Permissions

Please contact jbisynthesis@adelaide.edu.au for queries regarding reproduction and other permissions.

How to cite

Aromataris E, Munn Z (Editors). *JBI Manual for Evidence Synthesis.* JBI, 2020. Available from https://synthesismanual.jbi.global. https://doi.org/10.46658/JBIMES-20-01

ISBN: 978-0-6488488-0-6

For peer review only

Contributors

Current Contributors

Listed in alphabetical order (by surname).

João Apóstolo RN, PhD

The Portugal Centre for Evidence Based Practice: A JBI Centre of Excellence, Coimbra, Portugal

Edoardo Aromataris B.Sc.(Hons), PhD

JBI, Faculty of Health and Medical Sciences, University of Adelaide, South Australia, Australia

Merete Bjerrum MA, PhD

Department of Public Health, University of Aarhus, Aarhus, Denmark

Danish Centre of Systematic Reviews: A JBI Centre of Excellence, Aarhus, Denmark

Jared Campbell PhD

JBI, Faculty of Health and Medical Sciences, University of Adelaide, South Australia, Australia

Dennis Carmody PhD

Rutgers Robert Wood Johnson Medical School, Institute for the Study of Child Development, New Brunswick, United States

The Northeast Institute for Evidence Synthesis and Translation (NEST): A JBI Centre of Excellence, Newark NJ, USA

Judith Carrier PhD, MSC, PGCE, RGN

The Wales Centre For Evidence Based Care: A JBI Centre of Excellence, Cardiff, Wales

Kay Cooper PhD, MSc Physiotherapy, BSc Physiotherapy

Clinical Professor Allied Health Professions, School of Health Sciences, Robert Gordon University, Aberdeen, Scotland

The Scottish Centre for Evidence-based, Multi-professional Practice: A JBI Centre of Excellence, Aberdeen, Scotland

Marian Currie RM, PhD

Faculty of Health, University of Canberra, Canberra, Australia

Sandrine Ding PhD

University of Applied Sciences Western Switzerland, Haute École de Santé Vaud (HESAV), Department of Technical Medical Radiology, Lausanne, Switzerland

Bureau d'Echange des Savoirs pour des praTiques exemplaires de soins (BEST): a JBI Centre of Excellence, Lausanne, Switzerland

Ritin Fernandez RN, MN(Critical Care), PhD

For peer review only

Professor of Nursing, University of Wollongong, New South Wales

Centre for Evidence Based Initiatives in Health Care: A JBI Centre of Excellence, Wollongong, New South Wales, Australia

Silvia Florescu MD, PhD

National School of Public Health, Management and Professional Development, Bucharest

Public Health Evidence Based Romanian Centre: A JBI Centre of Excellence, Bucharest, Romania

Christina Godfrey RN, PhD

School of Nursing, Queen's University, Kingston, Ontario, Canada

Queen's Collaboration for Health Care Quality: A JBI Centre of Excellence, Kingston, Canada

Sasja Jul Håkonsen RN, MScN

Danish Centre of Systematic Reviews: A JBI Centre of Excellence, Aarhus, Denmark

Cheryl Holly EdD, RN, ANEF, FAAN

The Northeast Institute for Evidence Synthesis and Translation (NEST): A JBI Centre of Excellence, Newark NJ, USA

Lisa Hopp PhD, RN

Indiana Center for Evidence Based Nursing Practice: A JBI Centre of Excellence, Hammond IN, USA

Yuri Jadotte MD, PhD

The Northeast Institute for Evidence Synthesis and Translation (NEST): A JBI Centre of Excellence, Newark NJ, USA

Rutgers University, Nursing Science, Nothreast Institute for evidence synthesis and translation, New Jersey, USA

Hanan Khalil BPharm, MPharm, PhD

Monash University, Victoria, Australia

Pamela Kirkpatrick MSc, MA(Hons), BA

The Scottish Centre for Evidence-based, Multi-professional Practice: A JBI Centre of Excellence, Aberdeen, UK

Miloslav Klugar PhD

The Czech Republic (Middle European) Centre for Evidence-Based Health Care: A JBI Cenrre of Excellence, Prague, Czech Republic

Palacky University Olomouc, Department of Social Medicine and Public Health, Czech Republic

Jitka Klugarova PhD

The Czech Republic (Middle European) Centre for Evidence-Based Health Care: A JBI Centre of Excellence, Prague, Czech Republic

For peer review only

Samuel Lapkin PhD

Senior Lecturer, School of Nursing, University of Wollongong, Australia

Centre for Evidence Based Initiatives in Health Care: A JBI Centre of Excellence, Wollongong, New South Wales, Australia

Jo Leonardi-Bee PhD

Professor, Faculty of Medicine & Health Sciences, University of Nottingham, UK

The Nottingham Centre for Evidence-Based Healthcare: A JBI Centre of Excellence, Nottingham, UK

Karolina Lisy BSc(Hons), PhD

JBI, Faculty of Health and Medical Sciences, University of Adelaide, South Australia, Australia

Lucylynn Lizarondo BPhysio, MPhysio, MPsych, PhD

JBI, Faculty of Health and Medical Sciences, University of Adelaide, South Australia, Australia

Craig Lockwood RN, BNurs, MNSc, PhD

JBI, Faculty of Health and Medical Sciences, University of Adelaide, South Australia, Australia

Suzy Lockwood PhD

Texas Christian University, Fort Worth, Texas USA

The Center for Translational Research: A JBI Centre of Excellence, Fort Worth TX, USA

Heather Loveday RN, MA, PGCRM, PGCEA, Dip N(Lond)

The University of West London Centre for Evidence-Based Healthcare: A JBI Affiliated Group, London, UK

Cedric Mabire RN, PhD

Senior Lecturer at Institute of Higher Education and Research in Healthcare, University of Lausanne, UNIL

Bureau d'Echange des Savoirs pour des praTiques exemplaires de soins (BEST): a JBI Centre of Excellence, Lausanne, Switzerland

Patrick Mattis DNP, MSCS, RN, BC, CPHIMS

University of Medicine and Dentristry of New Jersey School of Nursing

The New Jersey Centre for Evidence Based Nursing: A Collaborating Centre of JBI at the University of Medicine and Dentistry of New Jersey

Alexa McArthur RN, RM, MPHC, MClinSc

JBI, Faculty of Health and Medical Sciences, University of Adelaide, South Australia, Australia

Trish McInerney PhD

The Wits-JBI Centre for Evidenced-Based Practice: A JBI Centre of Excellence, Johannesburg, South Africa

For peer review only

Diná de Almeida Lopes Monteiro da Cruz BSN, MNSc, PhD

University of São Paulo, Departamento de Enfermagem Médico-Cirúrgica

The Brazilian Centre for Evidence-based Healthcare: A JBI Centre of Excellence, São Paulo, Brazil

Sandeep Moola BDS, MHSM (Hons), MPhil, PhD

JBI, Faculty of Health and Medical Sciences, University of Adelaide, South Australia, Australia

Maria Teresa Moreno-Casbas RN, MSc, PhD FEAN

Instituto de Salud Carlos III, Nursing and healthcare research Unit

The Spanish Centre for Evidence Based Nursing and Healthcare: A JBI Centre of Excellence, Madrid, Spain

Peifan Mu RN, PhD

Taiwan Evidence Based Practice Centre: A JBI Centre of Excellence, Taipei, Taiwan

Zachary Munn BMedRad, GradDip HlthSc, PhD

JBI, Faculty of Health and Medical Sciences, University of Adelaide, South Australia, Australia

Micah Peters BHSc, MA(Q), PhD

JBI, Faculty of Health and Medical Sciences, University of Adelaide, South Australia, Australia

Kylie Porritt PhD, MNSc, BNurs, GradDipNursSc(Cardiac)

JBI, Faculty of Health and Medical Sciences, University of Adelaide, South Australia, Australia

Alin Preda MD, MPH

The Romanian Centre for Evidence Based Nursing: A JBI Centre of Excellence, Bucharest, Romania

Rubab Qureshi PhD, MD

Rutgers The State University of New Jersey, School of Nursing, New Jersey, USA

Dru Riddle PhD, DNP, CRNA

Texas Christian University, Fort Worth, Texas USA

The Center for Translational Research: A JBI Centre of Excellence, Fort Worth, Texas, USA

Kendra Rieger, RN, BN, PhD

Assistant Professor, College of Nursing, University of Manitoba, Winnipeg, Canada

Dagmara Riitano BA, GradDip(Psych), BHlthSc(Hons)

JBI, Faculty of Health and Medical Sciences, University of Adelaide, South Australia, Australia

For peer review only

Leslie Rittenmeyer RN, MS, CNS, Psy.D., CNE

Indiana Center for Evidence Based Nursing Practice: A JBI Centre of Excellence, Hammond, Indiana, USA

Susan Salmond EdD

Rutgers University, School of Nursing, New Jersey, USA

Kim Sears RN, PhD

Queen's Collaboration for Health Care Quality: A JBI Centre of Excellence, Kingston, Canada

Raluca Sfetcu PhD, MS

Spiru Haret University, Strada Ion Ghica, Bucharest, Romania

Daphne Stannard RN, PhD, CCRN, CCNS, FCCM

University of California, San Francisco (UCSF) Medical Center

UCSF Centre for Evidence Synthesis & Implementation: A JBI Centre of Excellence, San Franscisco, USA

Matthew Stephenson BBiotech (Hons), PhD

JBI, Faculty of Health and Medical Sciences, University of Adelaide, South Australia, Australia

Cindy Stern BHSc (Hons), PhD

JBI, Faculty of Health and Medical Sciences, University of Adelaide, South Australia, Australia

Judith Streak-Gomersall PhD

School of Public Health, Faculty of Health and Medical Sciences, University of Adelaide, South Australia, Australia

Andrea C Tricco BHSc (Hons), MSc, PhD

Queen's Collaboration for Health Care Quality: A JBI Centre of Excellence

Catalin Tufanaru MD, MPH

JBI, Faculty of Health and Medical Sciences, University of Adelaide, South Australia, Australia

Patraporn Tungpunkom RN, PhD

The Thailand Centre for Evidence Based Health Care: A JBI Centre of Excellence, Chiang Mai, Thailand

Sarahlouise White BSc (Hons), PhD

JBI, Faculty of Health and Medical Sciences, University of Adelaide, South Australia, Australia

Sally Wilson PhD, MSc(Curtin), BSc(Curtin), RN, RM, Paed Cert, CHN, MACN

For peer review only

Adjunct Research Fellow, School of Nursing, Midwifery and Paramedicine, Curtin University, Perth, Australia

The Western Australian Group for Evidence Informed Healthcare Practice: A JBI Centre of Excellence, Perth, Australia

Yifan Xue MBBS, MPH, PhD

JBI, Faculty of Health and Medical Sciences, University of Adelaide, South Australia, Australia

Hu Yan RN, PhD

Fudan University Centre for Evidence-based Nursing: A JBI Centre of Excellence, Shanghai, PR China

Past Contributors

Listed in alphabetical order (by surname).

Cassia Baldini Soares PhD, MPH, RN

Department of Collective Health Nursing, School of Nursing, University of São Paulo, São Paulo, SP, Brazil

The Brazilian Centre for Evidence-based Healthcare: A JBI Centre of Excellence, São Paulo, Brazil

Fiona J Bath-Hextall PhD

The Nottingham Centre for Evidence-Based Healthcare: A JBI Centre of Excellence, Nottingham, UK

Deborah Parker PhD

The New South Wales Centre for Evidence Based Health Care: A JBI Centre of Excellence, Sydney, Australia

Alan Pearson AM, RN, MSc, PhD, FRCNSA, FAAG, FRCN, FAAN

JBI, Faculty of Health and Medical Sciences, University of Adelaide, South Australia, Australia

Heath White Bbiotech, PhD, MTHlthSc

JBI, Faculty of Health and Medical Sciences, University of Adelaide, South Australia, Australia

For peer review only

Chapter 1: JBI Systematic Reviews

Edoardo Aromataris, Zachary Munn.

How to cite:

Aromataris E, Munn Z. Chapter 1: JBI Systematic Reviews. In: Aromataris E, Munn Z (Editors)*. JBI Manual for Evidence Synthesis.* JBI, 2020. Available from https://synthesismanual.jbi.global.. https://doi.org/10.46658/JBIMES-20-02

Chapter 1: Contents

- 1.1 Introduction to JBI Systematic reviews
- 1.2 Planning a JBI Review
- 1.3 Registering a review title and protocol
- 1.4 Publishing a JBI Systematic review
- 1.5 Systematic review standards
- 1.6 Disclosures and contributions
- 1.7 Chapter References

For peer review only

1.1 Introduction to JBI Systematic reviews

Systematic reviews aim to provide a comprehensive, unbiased synthesis of many relevant studies in a single document using rigorous and transparent methods. A systematic review aims to synthesize and summarize existing knowledge. It attempts to uncover "all" of the evidence relevant to a question.

Given the explosion of knowledge and access to a diverse range of knowledge sources over the past decade, it is now almost impossible for individual clinicians or clinical teams to stay abreast of knowledge in a given field. Systematic reviews (also referred to as research syntheses), conducted by review groups with specialized skills, set out to retrieve international evidence and to synthesize the results of this search into evidence to inform practice and policy. They follow a structured research process that requires rigorous methods to ensure that the results are both reliable and meaningful to end users.

Systematic reviews and meta-analyses began to appear in a variety of health fields in the 1970s and 1980s (Bastian et al. 2010). In the 1990s confusion arose between the terms 'systematic review' and 'meta-analysis' with the importance of using systematic approaches to reduce bias in reviews being distinguished as an issue separate from meta-analysis. Chalmers and Altman (1995) suggested that the term 'meta-analysis' be restricted to the process of statistical synthesis, that is meta-analysis may or may not be part of a systematic review. Growing interest in systematic reviews led to the emergence of international, interdisciplinary groups of scholars promoting and expanding upon systematic reviews (such as the JBI, Cochrane, The Campbell Collaboration etc.). Today the methodology of systematic reviewing still continues to evolve. JBI reviewers are encouraged to read the article by Aromataris and Pearson (2014) that provides an introductory overview regarding systematic reviews.

The quality of a systematic review depends heavily on the extent to which methods are followed to minimize the risk of error and bias during the review process. Such rigorous methods distinguish systematic reviews from traditional reviews of the literature. As such, explicit and exhaustive reporting of the methods used in the synthesis is a necessity and a hallmark of any well conducted systematic review. As a scientific enterprise, a systematic review will influence healthcare decisions and should be conducted with the same rigor expected of all research.

Currently, JBI has formal guidance for the following types of reviews:

1. Systematic reviews of experiences or meaningfulness
2. Systematic reviews of effectiveness
3. Systematic reviews of text and opinion/policy
4. Systematic reviews of prevalence and incidence
5. Systematic reviews of costs of a certain intervention, process, or procedure
6. Systematic reviews of etiology and risk
7. Systematic reviews of mixed methods
8. Systematic reviews of diagnostic test accuracy
9. Umbrella reviews
10. Scoping reviews

There is general acceptance of the following steps being required in a systematic review of any evidence type. These include the following:

1. Formulating a review question
2. Defining inclusion and exclusion criteria
3. Locating studies through searching
4. Selecting studies for inclusion
5. Assessing the quality of studies
6. Extracting data
7. Analyzing and synthesizing the relevant studies
8. Presenting and interpreting the results, potentially including a process to establish certainty in the body of evidence (through systems such as GRADE)

An essential step in the early development of a systematic review is the development of a review protocol. A protocol pre-defines the objectives and methods of the systematic review which allows transparency of the process which in turns allows the reader to see how the findings and recommendations were arrived at. It must be done prior to conducting the systematic review as it is important in restricting the presence of reporting bias. The protocol is a completely separate document to the systematic review report.

For peer review only

1.2 Planning a JBI Review

Prior to developing a protocol for your review, some preliminary investigation of the literature is recommended to determine if studies are available on the topic of interest. If you have a strong feeling that there are no studies available on your review topic, your energies may be better directed towards a different endeavor than conducting an ‘empty’ review.

In order to avoid duplication, reviewers are advised to register their review title (see Section 1.2). It is also recommended that reviewers search major electronic databases to determine that there have been no recently published systematic reviews on the same topic prior to registration of a review title. A search of the Cochrane Database, PubMed/MEDLINE, PROSPERO and DARE databases as well as our online journal, the *JBI Evidence Synthesis* will assist to establish whether or not a recent review report exists on the topic of interest. The results of this search should be mentioned in the background of the systematic review protocol and review. If a systematic review on the topic of interest has already been conducted, consider the following questions to establish if continuing with the review topic will be strategic.

- Is the date of last update longer than three years ago?

- Is it a high quality, well conducted systematic review?

- Do the methods reflect the specific criteria of interest for your topic?

- Is there a specific gap in terms of population or intervention outcome that has not been addressed in the identified review?

If a systematic review (or protocol) already exists on your topic, think carefully about conducting your review. To reduce duplication and a waste of human resources, it may be best not to conduct your review. However, there may be important reasons why you should still conduct your review. Your inclusion criteria may differ in terms of the population, context, interventions and even study types. Additionally, you may plan to use a different method for searching, critical appraisal and synthesis. In these cases, duplication may be appropriate. The other systematic review may also have some flaws in its conduct and reporting which warrants a new review.

Authors may also wish to consider the technical resources available to them. The conduct of a systematic review is greatly facilitated by access to extensive library and electronic databases and the use of citation management software as well as software designed specifically to facilitate the conduct of a systematic review such as JBI SUMARI.

When preparing to undertake a systematic review, consideration needs to be given to the human as well as the technical resources needed to complete the review. To maintain the required rigorous standards and alleviate risk of bias in the review process, a JBI review requires a minimum of two reviewers to conduct a systematic review. Authors should always consider the submission guidelines before submitting a manuscript to a journal. For example, the *JBI Evidence Synthesis* requires that at least one author has been trained in the JBI approach to systematic review by undertaking the Comprehensive Systematic Review Training Program, although it is ideal when all reviewers have undergone training. The skills and expertise required for a systematic review will vary depending on the nature of the review being undertaken and the methodology utilized. It is therefore recommended that a JBI systematic review is conducted by a team comprising of individuals that possess the skills and knowledge required to conduct the review to a standard acceptable for publication in an international scientific periodical.

Dependent upon the type of review being conducted, review teams should ideally consist of members with:

- Knowledge of general JBI systematic review methodology such as formulating a review question, defining inclusion criteria and critical appraisal.
- An information scientist or research librarian with specialised skills to develop and implement a comprehensive search strategy.
- Specific methodological expertise required for the type of review being undertaken, for example knowledge of the statistical methods to be used, experience in qualitative synthesis, or experience with economic analyses for economic evaluations.
- Knowledge of the topic area. Representation from clinical specialities and consumers is recommended where the review is being undertaken by systematic reviewers/methodologists rather than topic experts
- The ability to write a report in English to a publishable standard.

From the outset, the review team should consider expected contributions to the review project and eventual authorship. Some members of the review team may be better recognised in the acknowledgements of the published report rather than as authors. Conversely, part of the review team may be formally organised as a “Review Panel”, where some of the individuals with the attributes listed above provide formal advice and oversight throughout the conduct of the review including reviewing the draft protocol and final manuscript submissions or providing specific insight into the interpretation of data and formulating recommendations for practice and research for example. The names, contact details and areas of speciality of each member of the review panel should be included in both the protocol and the report.

For peer review only

1.3 Registering a review title and protocol

JBI Systematic review authors are encouraged to register their review title. This enables other reviewers to identify reviews that are currently underway and helps to avoid unnecessary duplication of research. A review title can be registered with JBI on completion of the online Systematic Review Title Registration Form. Once titles become registered with JBI, they are listed on the website.

JBI recommend that protocols of eligible review projects are registered with PROSPERO, the international prospective register of systematic reviews. If the protocol is registered, the final version of the systematic review should include the registration number provided by PROSPERO as well as the reference to the published protocol at the beginning of the 'Methods' section of the review report.

For peer review only

1.4 Publishing a JBI Systematic review

Authors should consider where they plan to submit their systematic review for publication from the outset. JBI systematic reviews are published in many international peer reviewed journals. JBI has two, multi-disciplinary international journals that frequently publish JBI systematic reviews; the *JBI Evidence Synthesis* and the *International Journal of Evidence Based Healthcare*. Both journals are published by Wolters Kluwer Health/Lippincott Williams and Wilkins. The target audience for JBI Systematic Reviews are academics and health professionals from across the health disciplines, including nurses, doctors, allied health professionals, mangers, administrators and decision makers in healthcare. The JBI journals accept submissions of all systematic and scoping review types. Once a topic has been identified and the title registered (optional), the completed protocol should be submitted for peer review to the *JBI Evidence Synthesis*.

For peer review only

1.5 Systematic review standards

Reporting standards similar to those produced for primary research designs (CONSORT, STROBE etc) have been created for systematic reviews. The PRISMA statement (Moher et al. 2009), or Preferred Reporting Items for Systematic Reviews and Meta-Analyses, provides a checklist for review authors on how to report a systematic review. JBI endorses the PRISMA statement. An extension to the PRISMA statement, PRISMA-P, outlines standards for systematic review protocols (Moher et al. 2015) and is similarly endorsed by JBI.

JBI reviewers should follow the guidance in this Manual and the *JBI Evidence Synthesis* author guidelines or other journal they are submitting to.

Other useful guidance also exists for the conduct of systematic reviews from other groups such as the Centre for Reviews and Dissemination (CRD), GRADE, Cochrane, Eppi-Centre, the Agency for Healthcare Research and Quality (AHRQ), reporting initiatives in the EQUATOR network, and the Institute of Medicine (IOM).

For peer review only

1.6 Disclosures and contributions

Transparency regarding contributions of individuals and organisations, conflicts of interest and sources of funding aligned to any published research also apply to JBI systematic reviews and should be presented under their own subsections.

Acknowledgements

The non-financial support or contribution of colleagues or institutions (for example, searching for evidence; providing access to unpublished data; feedback on review protocol; expert advice regarding the relevant existing literature) should be acknowledged and detailed. Acknowledgement should be reserved for individuals whose contribution does not constitute authorship.

Funding

Authors should provide details regarding any sources of funding for the review project. The role of all funders in the review process, if any, should be explicitly described. If the review is funded, then any potential conflicts of interest or intellectual bias of the funders should be specified in the review and the protocol.

Conflict of interests

All review authors should disclose any potential conflict of interest or any professional or intellectual bias. A statement should be included which declares the absence of any conflicts of interest and which describes a potential conflict of interest for authors for whom it has arisen. If consumers or other stakeholders assisted the authors with regards to the completed work, any potential conflict of interest or intellectual bias should be disclosed.

The review team should provide details regarding the management of any such conflicts or biases.

For peer review only

1.7 Chapter References

Aromataris, E. & Pearson, A. 2014, The Systematic Review: an overview. American Journal of Nursing, 114(3): 47-55.

Bastian H, Glasziou P, Chalmers I. Seventy-five trials and eleven systematic reviews a day: how will we ever keep up? PLoS Med 2010; 7(9):e1000326.

Chalmers I, Altman D. Systematic Reviews. BMJ Publishing Group, 1995.

Moher D, Liberati A, Tetzlaff J, Altman DG, The PRISMA Group (2009). Preferred Reporting Items for Systematic Reviews and Meta-Analyses: The PRISMA Statement. PLoS Med 6(7): e1000097. doi: 10.1371/journal.pmed1000097

Moher D, Shamseer L, Clarke M, Ghersi D, Liberati A, Petticrew M, Shekelle P, Stewart LA. Preferred Reporting Items for Systematic Review and Meta-Analysis Protocols (PRISMA-P) 2015 statement. Syst Rev. 2015;4(1):1. doi: 10.1186/2046-4053-4-1

For peer review only

Chapter 2: Systematic reviews of qualitative evidence

Craig Lockwood, Kylie Porritt, Zachary Munn, Leslie Rittenmeyer, Susan Salmond, Merete Bjerrum, Heather Loveday, Judith Carrier, Daphne Stannard.

How to cite:

Lockwood C, Porrit K, Munn Z, Rittenmeyer L, Salmond S, Bjerrum M, Loveday H, Carrier J, Stannard D. Chapter 2: Systematic reviews of qualitative evidence. In: Aromataris E, Munn Z (Editors). *JBI Manual for Evidence Synthesis.* JBI, 2020. Available from https://synthesismanual.jbi.global. https://doi.org/10.46658/JBIMES-20-03

Chapter 2: Contents

- 2.1 Introduction and purpose of this guidance
- 2.2 Introduction to qualitative evidence and evidence-based healthcare
- 2.3 Introduction to qualitative systematic reviews
- 2.4 The JBI Approach to qualitative synthesis
- 2.5 Core definitions in meta-aggregative reviews
- 2.6 Developing a qualitative review protocol
    - 2.6.1 Title of a qualitative review protocol
    - 2.6.2 Review question
    - 2.6.3 Introduction
    - 2.6.4 Inclusion criteria
        - 2.6.4.1 Types of participants
        - 2.6.4.2 Phenomena of interest
        - 2.6.4.3 Context
        - 2.6.4.4 Types of studies
        - 2.6.4.5 Example inclusion criteria
    - 2.6.5 Search strategy
    - 2.6.6 Assessment of methodological quality
    - 2.6.7 Data extraction
    - 2.6.8 Data synthesis
    - 2.6.9 Conflicts of interest and acknowledgements
- 2.7 Systematic review and synthesis of qualitative data
    - 2.7.1 Title
    - 2.7.2 Abstract
    - 2.7.3 ConQual 'Summary of Findings'
    - 2.7.4 Introduction
    - 2.7.5 Inclusion criteria
    - 2.7.6 Methods
        - 2.7.6.1 Search strategy
        - 2.7.6.2 Assessment of methodological quality
        - 2.7.6.3 Data extraction
        - 2.7.6.4 Data synthesis
    - 2.7.7 Results
        - 2.7.7.1 Study inclusion
        - 2.7.7.2 Methodological quality
        - 2.7.7.3 Characteristics of included studies
        - 2.7.7.4 Findings of the review
    - 2.7.8 Discussion
    - 2.7.9 Conclusions and Recommendations
    - 2.7.10 Conflicts and acknowledgements
    - 2.7.11 Review appendices
- 2.8 Chapter references
- Appendix 2.1: JBI Critical Appraisal Checklist for Qualitative Research
- Appendix 2.2: Discussion of JBI Qualitative critical appraisal criteria
- Appendix 2.3: JBI Qualitative data extraction tool

For peer review only

2.1 Introduction and purpose of this guidance

Methodological development for quantitative systematic reviews of effects has broad scientific consensus, however the same cannot be said across the field qualitative synthesis. In qualitative synthesis, the normative features ascribed to systematic reviews of quantitative data have been challenged, adopted, rejected, or transposed to different extents into analogous concepts and methods more attune to the nuances of the critical and interpretive research paradigms.

The purpose of this chapter is to provide the rationale, methodology and methods for meta aggregation as an approach to qualitative synthesis. Its developmental history is grounded in philosophic perspectives with the needs and expectations of evidence to inform health care decision-making. Meta aggregation is a method that mirrors the accepted conventions for systematic review whilst holding to the traditions and requirements of qualitative research (it aggregates findings in to a combined whole that is more than the sum of the individual findings in a way that is analogous with meta analysis).

For peer review only

2.2 Introduction to qualitative evidence and evidence-based healthcare

Introduction

This section provides an introductory perspective on qualitative synthesis, the relationship between evidence, qualitative evidence and health care practice and sets out a framework for considering the philosophic traditions associated with forms of research. A brief outline of some of the debates regarding qualitative synthesis is presented, although not with the intent of comprehensively addressing the significant variety of positions, but rather to assist in situating meta aggregation (the JBI approach to qualitative synthesis) as a methodology and where it sits within the wider debates.Importantly, key operational assumptions have been included in this section, as have the definitions of core terms for the process of extracting and synthesizing qualitative data. These definitions inform meta aggregation and represent a distinctive difference from other methods of qualitative synthesis that rely on the reviewer to re-interpret literature. The term meta aggregation is the formal name of the methodology, however, aggregative review, aggregative synthesis or meta synthesis are used interchangeably in this manual.

What is qualitative research?

Qualitative evidence or qualitative data allows researchers to analyze human experience and cultural and social phenomena (Jordan 2006). Qualitative evidence has its origins in research methods from the humanities and social sciences and seeks to analyze the complexity of human phenomena in naturalistic settings and from a holistic perspective (Ailinger 2003). The term 'qualitative' refers to various research methodologies including ethnography, phenomenology, qualitative inquiry, action research, discourse analysis and grounded theory. Research methods include interviews, whether group or individual and observation (either direct or indirect). Researchers who use qualitative methodologies seek a deeper understanding, aiming to "study things in their natural setting, attempting to make sense of, or interpret, phenomena in terms of the meanings people bring to them" (Denzin 2005).

In the healthcare or medical context, qualitative research:

"...seeks to understand and interpret personal experiences, behaviors, interactions, and social contexts to explain the phenomena of interest, such as the attitudes, beliefs, and perspectives of patients and clinicians; the interpersonal nature of caregiver and patient relationships; the illness experience; or the impact of human suffering". (Wong and Haynes 2004).

Qualitative evidence has a particular role in exploring and explaining why interventions are or are not effective from a person centered perspective, and address questions related to the usability, meaningfulness, feasibility and appropriateness of interventions. Similarly, qualitative evidence is able to explain and explore why an intervention is not adopted in spite of evidence of its effectiveness (Black 1994). The strength of qualitative research lies in its credibility (i.e. close proximity to the truth), using selected data collection strategies that "touch the core of what is going on rather than just skimming the surface" (Greenhalgh 1997).

Qualitative Evidence and Healthcare

Qualitative methods and data are increasing in usage in evidence-based healthcare research. Instead of quantifying or statistically portraying the data or findings, qualitative research focuses on individuals and gives voice to the patient/client or provider in the healthcare decision-making process. As an example, the question: 'What proportion of smokers have tried to give up?' leads to statistical answers while the question 'Why do people continue to smoke?', leads the researcher into exploring the ideas and concerns people who smoke tobacco may have about their smoking habits (Greenhalgh 1997).

Qualitative research is undertaken because it:

"...has an important role in evidence-based health care, in that it represents the human dimensions and experiences of the consumers of health care. This type of research does not answer questions concerning the effectiveness of health care; rather it provides important information about such things as the appropriateness of care and the impact of illness. It also provides a means of giving consumers a voice in the decision-making process through the documentation of their experiences, preferences, and priorities..." (Evans 2002).

Qualitative research plays a significant role in understanding how individuals and communities perceive health, manage their own health and make decisions related to health service usage. It can assist to understand the culture of communities, in relation to implementing changes and overcoming barriers. It can also inform planners and policy makers about the manner in which service users experience health as well as illness, and can be used to evaluate activities of health services such as health promotion and community development.

For peer review only

Acknowledgement of the contribution that qualitative research findings make in improving the quality and relevance of healthcare conditions is increasing. As an example, *Systematic reviews. CRD's guidance for undertaking reviews in health care* published by the Centre for Reviews and Dissemination at the University of York states that ‘There is growing recognition of the contribution that qualitative research can make to reviews of effectiveness’ as it helps to develop an understanding of the people, the practices and the policies behind the mechanisms and interventions (CRD 2009).

Qualitative evidence comprises data that is expressed in terms of the meaning or experiences of acts or events rather than in terms of a quantitative measurement. (Barbour 1999, Moffatt et al. 2006, Forman et al. 2008) Arguably one of the best features of its contribution to research inquiry lies in its stories and accounts of living and its richness of meanings within its words (Forman et al. 2008).

Philosophical perspectives, research methodologies and methods

A philosophical perspective encompasses our assumptions of the theory and the research methodologies that guide research. There are three prevailing philosophical or guiding paradigms in current western health care research. The first is the positivist – or empirico- analytical –paradigm, often associated with quantitative evidence (see Chapter 3) while the other two, the interpretive and critical paradigms, are largely associated with qualitative evidence. In the interpretive paradigm, theory is inductive and concerned with exposing implicit meaning; it aims at understanding. The critical paradigm, like the interpretive, is inductive, however it aims to emancipate knowledge and practice. Each paradigm is encompasses a diversity of research methodologies and methods (methods being the specific approach to data collection).

An outline of the key research methodologies and methods associated with the interpretive and critical paradigms is shown in Table 2.1.

Table 2.1: A summary of qualitative philosophy, methodologies and methods.

	**Methodologies**	**Data Collection Methods**
**Interpretivism**<br><br>*Seeks to understand. Sees knowledge in the possession of the people.*	**Phenomenology**<br><br>Seeks to understand people’s individual subjective experiences and interpretations of the world.<br><br>**Ethnography**<br><br>Seeks to understand the social meaning of activities, rituals and events in a culture.<br><br>**Grounded Theory**<br><br>Seeks to generate theory that is grounded in the real world. The data itself defines the boundaries and directs development of theory.	Interviews.<br><br>Focus groups Observations.<br><br>Field work. (Observations, Interviews) Interviews.Field observations. Purposeful interviews Textual analysis.
**Critical enquiry**<br><br>*Seeks to change.*	**Action research**<br><br>Involves researchers participating with the researched to effect change.<br><br>**Feminist research**<br><br>Seeks to create social change to benefit women.<br><br>**Discourse Analysis**<br><br>assumes that language socially and historically constructs how we think about and experience ourselves, and our relationships with others.	Participative group work Reflective Journals. (Quantitative methods can be used in addition to qualitative methods).<br><br>Qualitative in-depth interviews. Focus Groups. (Quantitative methods can be used in addition to qualitative methods).<br><br>Study of communications, written text and policies.

For peer review only

2.3 Introduction to qualitative systematic reviews

There is no hierarchy of evidence among methodologies for qualitative studies. A meta aggregative systematic review does not require any distinction between critical or interpretive studies. The units of analysis sought from qualitative papers are the findings, presented as themes, metaphors or concepts as identified by the researchers (not the reviewer). Accordingly, meta aggregative reviews include a range of methodological studies in order to capture the whole of a phenomenon of interest, rather than merely a one dimensional aspect. The rationale for this is that the traditions of the methodology employed in a study are considered to be embedded within the findings, rather than distinct to the findings. This implies that when a finding is extracted, the perspective or context that the study author intended for the finding is not lost, but is embedded in the extraction.

The synthesis of qualitative data

The perspectives of primary qualitative researchers has had a significant impact on development of methods for qualitative synthesis. It has been proposed that this may in part due to the fact that primary qualitative researchers conceive of paradigms as emblematic of their ability to situate not only themselves but also their work in relation to knowledge generation. As Chin and Jacobs (1987) assert, knowledge as subjective truth requires a researcher or author to explicitly state their chosen paradigm as it has implications for how a reader will understand the written word and how the methodology and methods will be read and understood.

This is no less appropriate in qualitative synthesis. Indeed, Sandelowski and Barroso (2007), although reluctant to create or promulgate rules for qualitative synthesis, posit that the first rule (if any should exist) is that the methods of synthesis should not violate the philosophic foundations (i.e. paradigm) of the approach used. It is evident then that while synthesis is a different process to primary research, the principles and processes of qualitative synthesis must be sensitive to the core assumptions of the critical and interpretive paradigms. The synthesis of qualitative data is also contested among qualitative researchers themselves, based on philosophical and methodological differences between the different qualitative research approaches (Sandelowski et al. 1997, Thorne et al. 2004)

Of the views that characterize the ongoing debate surrounding the meta-synthesis of qualitative evidence, one area of focus is the perceived degree of ‘interpretiveness” of the approach to data analysis. There has been extensive debate in the literature as to what constitutes an’interpretive’ review, and whether some qualitative synthesis approaches are more or less interpretive than others. These debates tend to focus on the synthesis component of the systematic review, and attempt to classify the whole of a review methodology on the basis of whether the synthesis component can be labelled as either ’inductive’ or ’deductive’. A further issue is whether qualitative synthesis methodologies should fit within the accepted conventions for systematic review or whether qualitative synthesis methodologies should be more reflective of primary qualitative methodologies. Approaches to qualitative synthesis that are more aligned with primary qualitative methodologies may not require reviewers to undertake comprehensive searching, appraisal to establish quality is not considered important, and data extraction and synthesis may be iterative and based upon the re-interpretation of published data.

For peer review only

2.4 The JBI Approach to qualitative synthesis

The JBI uses a meta-aggregative approach to the synthesis of qualitative evidence. Meta aggregation is sensitive to the nature and traditions of qualitative research while being predicated on the process of systematic review (Pearson 2004). The meta-aggregative approach is sensitive to the practicality and usability of the primary author's findings and does not seek to re-interpret those findings as some other methods of qualitative synthesis do. A strong feature of the meta-aggregative approach is that it seeks to enable generalizable **statements in the form of recommendations to guide practitioners and policy makers** (Hannes and Lockwood 2011). In this regard, meta aggregation contrasts with meta-ethnography or the critical interpretive approach to qualitative evidence synthesis, which have a focus on re-interpretation and theory generation rather than aggregation.

The JBI recognizes the usefulness of alternate interpretive approaches such as meta-ethnography, as well as narrative synthesis and thematic synthesis. By way of illustration:

- the usefulness of meta- ethnography lies in its ability to generate theoretical understandings that may or may not be suitable for testing empirically,
- narrative synthesis of text is useful in drawing together different types of research evidence (e.g. qualitative, quantitative, economic), and
- thematic synthesis is of use in drawing conclusions based on common elements across otherwise heterogeneous studies.

JBI considers, however, that these approaches do not seek to provide guidance for action and aim only to 'anticipate' what might be involved in analogous situations and to understand how things connect and interact. Meta-aggregation is the preferred JBI approach for developing recommendations for action. The JBI SUMARI software is designed to facilitate meta-aggregation, however it can also be used successfully in meta-ethnography and other interpretive processes as a data management tool.

The core assumptions detailed in subsequent sections of this Chapter include:

- The requirement for an *a priori* protocol that describes all steps in the review, decisions on how they will be undertaken and appends all templates that will be used during the review;
- Comprehensive and exhaustive searching, independent critical appraisal and standardised data extraction;
- Synthesis of findings that authentically represents the aggregation of data from primary studies;
- Presentation of a meta-aggregative schematic that represents the findings and their aggregation in to categories, and the aggregation of categories in to synthesized findings; and
- The development of recommendations for policy or practice with assigned grades of recommendation.

For peer review only

2.5 Core definitions in meta-aggregative reviews

The operational definitions that characterize meta-aggregation describe the data to be synthesized, and explain what each step looks like.

Finding:

- A finding is a verbatim extract of the author's analytic interpretation of their results or data.

In undertaking the synthesis component of a meta-aggregative review, each finding that is extracted from a paper is accompanied by an illustration.

An illustration is defined as

- A direct quotation of a participant's voice, field-work observation or other supporting data from the paper.

For each extracted finding, a level of credibility is allocated, and this is completed in JBI SUMARI as the data for the finding and its accompanying illustration are entered. Levels of credibility are described in Section 2.7 of this chapter.

Category:

A category is a brief description of a key concept arising from the aggregation of two or more like findings and is accompanied by an explanatory statement that conveys the whole, inclusive meaning of a group of similar findings.

When two or more findings are combined to form a category, a category description is also created. A category description is defined as:

- An explanatory statement that conveys the whole, inclusive meaning of a group of similar findings.

A category illustration is developed by the review team, it is an explanatory statement that conveys the whole inclusive meaning of a group of similar findings.

Synthesized finding:

A synthesized finding is an overarching description of a group of categorized findings. Synthesized findings are expressed as 'indicatory' statements that can be used to generate recommendations for policy or practice.

As with categories, a description is created for each synthesized finding. The description for a synthesized finding is defined as:

- An explanatory statement that conveys the whole, inclusive meaning of a group of similar categories

These core definitions are the basis of meta aggregation and represent a goodness of fit with systematic review that is much closer than many other qualitative approaches to synthesis.

For peer review only

2.6 Developing a qualitative review protocol

This section outlines the components of a systematic review protocol of qualitative evidence and provides guidance on the information that each component should contain. Specifically, it provides guidance on each of the following components: title, review objectives/questions, background, inclusion criteria, search strategy, critical appraisal, data extraction, data synthesis, narrative summary, references, and appendices.

For peer review only

2.6.1 Title of a qualitative review protocol

The title should be informative and give clear indication of the topic of the review. Titles should not be phrased as questions and there should be congruency between the title, review objectives/questions and inclusion criteria. The title of the protocol should be structured and reflective of the core elements of the PICo (see Section 2.6.2). The title should always include the phrase "…:a protocol for the synthesis of qualitative evidence" or "...: a qualitative systematic review protocol", for example, to allow easy identification of the type of document it represents. A JBI review requires at least two reviewers. The names of all reviewers, affiliations for each author including their JBI centre affiliations and email address for the corresponding author should be included.

For peer review only

2.6.2 Review question

Clarity in the review questions assists in developing a protocol and also ultimately, the conduct of the review proper. The review question/s guide and direct the development of the specific review criteria and facilitate more effective searching, and provides a structure for the development of the full review report. Although a range of mnemonics have been described for different types of review (and research) questions, for a JBI qualitative synthesis, the PICo mnemonic also be used to construct a clear and meaningful question for a JBI systematic review of qualitative evidence. The PICo mnemonic stands for the Population, the Phenomena of Interest and the Context. There is no need for an outcome statement in qualitative synthesis (see Chapter 3). The expression of the phenomena of interest represents the outcome, therefore a specific outcome section or statement is not recommended in meta aggregation.

The review question and PICo mnemonic can provide potential readers with a significant amount of information about the focus, scope and applicability of a review to their needs. It should be apparent if the review is examining meaning or lived experience or a specific phenomena of interest is to be examined. Similarly, including the context in the question assists readers to situate the review.

A qualitative review will have a primary question. If that question sufficiently addresses the review objectives, there is no need for secondary or sub questions. However, as per the illustrations below, some questions benefit from one or more sub questions that delve into particular attributes of context, population or phenomena of interest.

For example, the primary question (aligned directly to the objective) below relates to the nursing profession, however, the sub questions delve into the particular issues related to professionally trained nurses and student nurses as distinct sub populations (Rittenmeyer et al. 2012):

What are the experiences of lateral or horizontal violence in the profession of nursing?

- *What is the experience of lateral or horizontal violence for professional nurses?*
- *What is the experience of lateral or horizontal violence for student nurses?*

In this example, the PICo elements can be readily identified: the Population of interest are nurses, professional or student. The Phenomenon of interest is their experience with lateral or horizontal violence and the context, which has not been explicitly stated in the question in this case may be in tertiary care or in the health system of a particular country for example.

For peer review only

2.6.3 Introduction

Every systematic review requires a clear and meaningful introduction section. Given the international circulation of systematic reviews, it is important to state variations in local understandings of clinical practice (including 'usual practice'), health service management and client or patient experiences. The introduction should describe and situate the phenomena of interest under review, as well as the population and context. The introduction should cover the main elements of the topic under review. The purpose of the introduction is to:

- situate the PICo and put the inclusion criteria into context,
- provide context to the review
- define key terms and list operational definitions
- refer to existing international literature to support and inform the inclusion criteria,
- provide indication that the review question has not been addressed previously, and
- justify the rationale and conduct of the review.

The introduction should avoid synthesizing findings from multiple authors given this is exactly what your review will aim to achieve, it should however, provide some indication that there is evidence available that will be included in your review and inform your question.

As mentioned above, the introduction should include a statement that a preliminary search for existing systematic reviews on the topic has been conducted (state the sources searched e.g. *JBI Evidence Synthesis*, Cochrane Database, CINAHL, PubMed, PROSPERO where relevant). If there is an existing systematic review, it should be specified how the proposed review will differ.

The introduction should conclude with an overarching review objective that captures and aligns with the core elements/mnemonic of the inclusion criteria (e.g. PICo). The stated objective should clearly indicate what the review project is trying to achieve. Vancouver style referencing should be used throughout the protocol with superscript numbers without brackets used for in-text citations.

For peer review only

2.6.4 Inclusion criteria

This section of the protocol details the basis on which studies will be considered for inclusion into the systematic review and should be as clear and unambiguous as possible. The inclusion criteria for a review are not designed to applied independently of each other, therefore each should be presented as mutually exclusive criteria and repetition between elements of the PICo is not necessary.

For peer review only

2.6.4.1 Types of participants

There needs to be a clear and direct link between the review question, title and the participant characteristics in the inclusion criteria.

For example, the population characteristics for conservative treatment for men may consider:

- Age ranges (18-75)
- Sex (male)
- A diagnosis of prostate cancer (diagnosed within the last six months, either new, or recurrent disease)
- Staging of severity of prostate cancer (I-IV)

The population should be clearly described and avoid ambiguity that may confound study selection.

Specific exclusion based on any participant or population characteristics should also be articulated in this section. In this example, patients with secondary tumor or metastasized cancer will be excluded.

For peer review only

2.6.4.2 Phenomena of interest

There should be congruence between the review question, title and the phenomena of interest.

In the example of men diagnosed with prostate cancer the phenomena of interest are their experiences with receiving conservative treatment. Details of the treatment in this case should have been well defined in the background section, though maybe reiterated briefly here as a guide for the study selection phase of the review when these criteria will be applied.

For peer review only

2.6.4.3 Context

In a qualitative review, context will vary depending on the objective and question(s) of the review. Context may include but is not limited to consideration of:

- cultural or sub-cultural factors,
- geographic location,
- specific racial or gender based interests, or
- detail about the specific setting (such as acute care, primary health care, or the community).

There is no requirement for an outcome statement in qualitative reviews as the expressed phenomena of interest is the outcome.

For peer review only

2.6.4.4 Types of studies

There should be a match in this section between the methodology of the primary research studies to be considered for the review and the review question.

The JBI SUMARI software offers standardized text consisting of statements regarding the types of studies considered for inclusion in a meta aggregative review. Any of the following 3 options provide an appropriate structure for a qualitative review:

- Option 1: This review will consider studies that focus on qualitative data including, but not limited to, designs such as phenomenology, grounded theory, ethnography, action research and feminist research.
- Option 2: This review will consider interpretive studies that draw on the experiences of <insert text> with <insert text> including, but not limited to, designs such as phenomenology, grounded theory, ethnography, action research and feminist research.
- Option 3: This review will consider critical studies that explore <insert text> including, but not limited to, designs such as action research and feminist research.

As can be seen from the three set text options above, creating a protocol for an interpretive or critical or generalist systematic review depends on the nature of the question being addressed. Interpretive reviews are conducted to aggregate evidence related to social interactions that occur within health care, or seek to establish insights into social, emotional or experiential phenomena. Critical reviews might be conducted to explore issues such as power or change. A critical *and* interpretive review might be conducted to bring both elements together.

A narrow approach in terms of focusing solely on either interpretive or critical designs alone is not recommended unless there is a clear, rationale and theoretically informed requirement to do so. The international consensus is heavily in favor of inclusive reviews of literature across both the critical and interpretive paradigm.

For peer review only

2.6.4.5 Example inclusion criteria

How the PICo elements of a review question are presented in the inclusion criteria is illustrated below on this example taken from Rittenmeyer and colleagues (2012) addressing the objective of nurses experiences with lateral and horizontal violence (see Section 2.6.2).

Inclusion Criteria

Types of Participants

This review will consider studies that include licensed nurses and student nurses. For purposes of this review 'licensed nurse' refers to a nurse who holds a license to practice nursing at any level. Due to the ambiguity of nomenclature, different titles for licensed nurse will be considered, including but not limited to registered nurse, practical nurse, vocational nurse.

Phenomena of Interest

The phenomenon of interest for this review is the actual experience of horizontal/lateral violence.

Context

This review will consider any setting where licensed or student nurses practice.

Types of studies

This review will consider studies that focus on qualitative data including, but not limited to, designs such as phenomenology, grounded theory, ethnography, and action research. Descriptive qualitative studies that describe the experience or describe the effects of the experience will also be considered.

For peer review only

2.6.5 Search strategy

This section details how the reviewers plan to search for and locate relevant studies. The process describing searching has been standardized in JBI SUMARI and is illustrated below. A systematic review should consider papers published by both commercial and academic publishers as well as grey literature. Rather than compete with the published literature, grey literature has the potential to complement and communicate findings to a wider audience. Grey or Gray literature is also known as Deep or Hidden Web material may include: Theses and Dissertations, Reports, blogs, technical notes, non-independent research or other documents produced and published by government agencies, academic institutions and other groups that are not distributed or indexed by commercial publishers. Systematic literature searching for qualitative evidence presents particular challenges. Some databases lack detailed thesaurus terms either for qualitative research as a genre or for specific qualitative methods. Additionally, changes in thesaurus terms mean that reviewers need to be cognizant of the limitations in each database they may use. The help of an experienced research librarian/information scientist is recommended.

The time frame chosen for the search should be justified and any language restrictions stated (e.g. only studies published in English will be considered for inclusion). The databases to be searched must be identified listed, including the search platform used where necessary, along with a completed search strategy for one major databases which should be presented as Appendix I of the review protocol.

The search strategy is described as a three-phase process:

Phase one consists of two steps:

1. the identification of initial key words based on knowledge of the field to perform an initial search where the reviewer creates a logic grid of key words from titles and abstracts; and
2. the analysis of text words contained in the titles and abstracts of papers, and of the index terms used in a bibliographic database to describe relevant articles in order to build comprehensive and specific search strategy for each included database.

Phase two involves implementing database-specific searches for each database included in the protocol.

Phase three involves the review of the reference lists of all studies that are retrieved for appraisal to search for additional studies.

The process describing searching has been standardized in SUMARI as follows:

The search strategy will aim to find both published and unpublished studies. An initial limited search of M EDLINE and CINAHL has been undertaken followed by analysis of the text words contained in the title and abstract, and of the index terms used to describe article. This informed the development of a search strategy which will be tailored for each information source. A full search strategy for #name the relevant database# is detailed in Appendix 1. The reference list of all studies selected for critical appraisal will be screened for additional studies.

Information sources:

The databases to be searched include:

Insert databases here

The search for unpublished studies will include:

Insert sources here

This standardized text is editable, and includes fields for reviewers to specify content relevant to their available resources. As mentioned, reviewers are required to state the databases to be searched and, if including unpublished studies, what sources will be accessed. An additional paragraph that addresses whether hand searching will be conducted, which sources will be subject to hand searching (e.g. the searching of journals that are not indexed in electronic databases), should be added to the review protocol as part of Phase 2 if required. The search strategy should also describe all limitations to the scope of searching in terms of dates, resources to be accessed or languages. Each of these may vary depending on the nature of the topic being reviewed, or the resources available to the review team.

Limiting by date:

Limiting the search by date may be used where the focus of the review is on a more recent intervention or innovation. However, potentially relevant studies as well as seminal, early studies in the field may be missed if the limit set is too recent thus date limits should be used in an informed way, based on knowledge of key papers relevant to the review question that must be cited to provide evidence for the decisions made to limit the search.

Limiting by resources accessed:

For peer review only

Limiting the search to a small number of databases is a hot topic in systematic review searching. The validity of systematic reviews relies in part on access to an extensive range of electronic databases for literature searching. There is inadequate evidence to suggest a particular number of databases, or even to specify if any particular databases should be included. The comprehensiveness of searching and the documentation of the databases searched is a core component of the systematic review's credibility.

Limiting by language:

Limiting by language is a common practice in settings with lack of ready access to translators. The caveat associated with excluding papers based upon language is that important cultural contexts or findings may be missed. The exclusion of selected languages also means the review audit trail is incomplete. If limiting by language is required, it is preferable to search inclusively, and keep a record of numbers of studies per language group. This allows the reader to identify how many studies have been identified, but are not included, therefore promoting transparency in the process.

Alternatively, many papers in languages other than English are abstracted in English, from which reviewers may decide to retrieve the full paper and seek to collaborate with other entities regarding translation.

Therefore, literature searching should be based on the principle of comprehensiveness, with the widest reasonable range of databases that are considered appropriate to the focus of the review.

For peer review only

2.6.6 Assessment of methodological quality

Qualitative studies that are eligible for inclusion in the review must be assessed for methodological quality. There are a variety of checklists and tools available to assess research syntheses and systematic reviews. Most checklists use a series of criteria that can be scored as being “met” or “not met” or “unclear” and in some instances as “not applicable”. The decision as to whether or not to include a study can be made based on meeting a pre-determined proportion of all criteria, or on certain criteria being met. It is also possible to weight certain criteria differently. Decisions about a scoring system or any cut-off for exclusion should be made in advance and agreed upon by all reviewers before critical appraisal commences. The protocol, therefore, should detail how selected studies will be assessed for quality, e.g. use of a predetermined cut off score.

All included studies need to be critically appraised using the standard JBI critical appraisal instrument for qualitative research that is available in Appendix 2.1 of this chapter (further details regarding the appraisal questions can be found in Appendix 2.2). The assessment criteria are built into JBI SUMARI. The tool is designed to be used with two independent reviewers conducting the critical appraisal of each research synthesis selected. Reviewers are blinded to each other’s assessment and assessments can only be compared once initial appraisal of an article is completed by both reviewers. Where there is a lack of consensus, discussion between reviewers should occur. In some instances it may be appropriate to seek assistance from a third reviewer. The source of the JBI critical appraisal tool for research syntheses should be cited in the protocol.

NB: If the best available evidence for your question is text and opinion rather than qualitative research, the text and opinion studies should be analysed using the text and opinion module of SUMARI. Such reviews become a text and opinion review (see Chapter 4) rather than a qualitative review of evidence, and therefore the review title, question and criteria should be reviewed against the expectations of a text and opinion review.

For peer review only

2.6.7 Data extraction

Standardized data extraction tools promote the extraction of similar data across all of the included studies and are required for JBI systematic reviews. The protocol should detail what data the reviewers plan to extract from the included studies and the data extraction tool should be appended to the protocol (see Appendix 2.3). The set text from SUMARI describes this process:

Qualitative data will be extracted from papers included in the review using the standardized data extraction tool from JBI SUMARI by two independent reviewers. The data extracted will include specific details about the populations, context, culture, geographical location, study methods and the phenomena of interest relevant to the review question and specific objectives. Findings, and their illustrations, will be extracted and assigned a level of credibility.

For peer review only

2.6.8 Data synthesis

The protocol should also describe how the findings extracted from the included studies will be synthesized. Qualitative research findings should be pooled using JBI SUMARI as per the set text below:

Qualitative research findings will, where possible be pooled using JBI SUMARI with the meta-aggregation approach. This will involve the aggregation or synthesis of findings to generate a set of statements that represent that aggregation, through assembling the findings and categorizing these findings on the basis of similarity in meaning. These categories are then subjected to a synthesis in order to produce a single comprehensive set of synthesized findings that can be used as a basis for evidence-based practice. Only unequivocal and credible findings will be included in the aggregation. Not-supported findings will be presented separately. Where textual pooling is not possible the findings will be presented in narrative form.

For peer review only

2.6.9 Conflicts of interest and acknowledgements

Details of requirements in these sections are described in Section 1.6 of this Manual.

Conflicts of interest
A statement which either declares the absence of any conflicts of interest or which describes a specified or potential conflict of interest should be made by the reviewers in this section.

Acknowledgements
Any acknowledgements should be made in this section e.g. sources of external funding or the contribution of colleagues or institutions. It should also be noted if the systematic review is to count towards a degree award.

For peer review only

2.7 Systematic review and synthesis of qualitative data

This section provides guidance on the components that should comprise a JBI systematic review report of qualitative evidence and the information that each component should contain. It illustrates how each component of the review is managed by SUMARI and the outputs that can be expected if JBI SUMARI has been used by the reviewer(s). This section also provides a brief outline of how the systematic review should be formatted and the stylistic conventions that should be used to ensure the review meets the criteria for publication in the *JBI Evidence Synthesis*. Specifically, guidance is provided on the following components: layout of the report, inclusion criteria (i.e., PICo), search strategy, critical appraisal, data extraction, data synthesis, results, and conclusions. The section also presents a series of questions designed to prompt the reviewer to check that certain key information or requirements have been adequately addressed in the review.

For peer review only

2.7.1 Title

The title should be clear, explicit and reflect the core elements of the review. Titles should not be phrased as questions or conclusions and there should be congruency between the title, review objectives /questions and inclusion criteria. The title should include the phrase ‘A systematic review’.

For peer review only

2.7.2 Abstract

This section forms a structured abstract of the main features of the systematic review. It must be no longer than 500 words and should contain no abbreviations or references. The abstract must accurately reflect and summarize the systematic review with the main focus on the results of the review.

The abstract should report the essential elements of the review using the following sub-headings in this order:

- **Objective**: State an overarching review objective structured using the key components of the inclusion criteria (approximately one to two sentences).
- **Introduction**: Briefly describe what is already known on the topic and what this review will add to the evidence-base (approximately two to three sentences).
- **Inclusion criteria:** Summarize the inclusion criteria as it relates to the type of review being conducted. Present the information in one or two sentences – **NOT** under individual subheadings.
- **Methods:** List the key information sources searched (those that provided the majority of included studies), any limits placed on the scope of the search (e.g. language), and the date range, or the date of the last search. If the recommended JBI approach to critical appraisal, study selection, data extraction and data synthesis was used, simply state it as such (without naming the actual tool). Otherwise, briefly describe any notable deviations to the methodological approach taken (e.g. criteria used to exclude studies on the basis of methodological quality etc.).
- **Results**: The bulk of the abstract should be reserved to convey the main results of the review.
    - As a general rule, report the number and type of included studies and participants, as well as any pertinent study characteristics. Summarize the overall quality of the included studies and notable aspects of rigor for qualitative reviews).
    - Report the number of findings and categories and final synthesized findings. Depending how many are presented in the review, the synthesized findings may be presented here or abridged summarized statements.
- **Conclusions**: Provide a conclusion based on a general interpretation of the results considering, for example, the methodological quality of the included studies and any limitations of the review. Briefly convey key implications for practice and/or research.

For peer review only

2.7.3 ConQual 'Summary of Findings'

CONQual (Note: the output Summary of Findings table from the CONQual process should be presented after the review Abstract)

In ConQual (Munn et al. 2014), each paper is initially ranked from High to Very Low – qualitative papers are ranked as High, while text and opinion papers are ranked Low. From this starting point, each paper is then graded for Dependability, and then Credibility as per the schema below. ConQual Score Calculation:

1. **Initial Ranking** scale for qualitative studies
   a. High
   b. Moderate
   c. Low
   d. Very Low

Assign a pre-ranking of papers, using the following schema:

– High for qualitative studies

– Low for expert opinion

Dependability

The ranking per paper moves up or down (or stays the same) depending on the Dependability Score as follows:

4-5 'yes' responses, the paper remains unchanged

2-3 'yes' responses: move down 1 level

0-1 'yes' responses: move down 2 levels

The Dependability score is based on the following specific questions from the critical appraisal scores for included studies related to the appropriateness of the conduct of the research with research aims and purpose:

1. Is there congruity between the research methodology and the research question or objectives?
2. Is there congruity between the research methodology and the methods used to collect data?
3. Is there congruity between the research methodology and the representation and analysis of data?
4. Is there a statement locating the researcher culturally or theoretically?
5. Is the influence of the researcher on the research, and vice-versa, addressed?

Credibility

- Assign a level of credibility to the synthesised finding by cross checking how many findings of what type were included in the categories associate with the synthesized finding:

– **Unequivocal (U)** – relates to evidence beyond reasonable doubt which may include findings that are matter of fact, directly reported/observed and not open to challenge.

– **Credible (C)** – those that are, albeit interpretations, plausible in light of data and theoretical framework. They can be logically inferred from the data. Because the findings are interpretive they can be challenged.

– **Not Supported (NS)** – when 1 nor 2 apply and when most notably findings are not supported by the data

- Rank according to the following scoring rubric for each synthesised finding:

  All unequivocal findings: remains unchanged.

  mix of unequivocal/credible findings: downgraded one (-1).

  credible/not supported findings: downgraded three (-3).

  *table is modified from source

Please note: For JBI qualitative reviews not-supported findings should not be included in the meta-aggregative process.

With the ConQual Score established for each synthesised finding, the Summary of Findings table can now be completed. *Cite Munn et al. 2014* when integrating ConQual.

Summary of Findings Table

For peer review only

Systematic review title: insert title here<br>Population: describe population of interest<br>Phenomena of interest: insert the specific phenomena of interest<br>Context: Concise description of the key contextual factors					
Synthesised Finding	Type of research	Depen dability	Cred ibility	ConQua l Score	Com ments
Insert each synthesized finding, and complete the columns per synthesized finding, keeping the rows aligned					

For peer review only

2.7.4 Introduction

The introduction should be comprehensive and cover all the main elements of the topic under review. It should be presented in complete prose, avoid lists and use sub headings sparingly and to improve logical flow of content and readability. Reviewers will find that the background information provided with the protocol needs modification or extension following the conduct of the review proper; the introduction of the review should not be a duplicate of that presented in the published protocol. The introduction should detail any definitions important to the review. The background information in this section must be sufficient to put the inclusion criteria into context and clear indication why the review is important and the rationale for its conduct. The introduction should conclude with a statement that a preliminary search for previous systematic reviews on the topic was conducted (state the sources searched e.g. *JBI Evidence Synthesis*, Cochrane Database, CINAHL, PubMed, PROSPERO). If there is a previous systematic review on the topic, it should be specified how the proposed review differs.

The introduction should conclude with an overarching review objective that captures and aligns with the core elements/mnemonic of the inclusion criteria (e.g. PICo). The stated objective should clearly indicate what the review project is trying to achieve. Vancouver style referencing should be used throughout the review with superscript numbers without brackets used for in-text citations.

For peer review only

2.7.5 Inclusion criteria

This section of the review details the basis on which studies were considered for inclusion in the systematic review and should be as clear and unambiguous as possible.

Types of participants

The types of participants should be appropriate for the review objective(s) and question(s). The reasons for the inclusion or exclusion of participants should be explained in the background.

Phenomena of interest

There should be congruence between the review objective(s) and question(s) and the phenomena of interest. How the phenomena relate to the topic under review should be clear and detailed in the background section.

Context

In a qualitative review, context will vary depending on the objective of the review. Context may include, but is not limited to, consideration of cultural factors such as geographic location, specific racial or gender based interests, or detail about the specific setting (such as acute care, primary health care, or the community).

Types of studies

There should be a match in this section between the methodology of the primary research studies that were included in the review.

For peer review only

2.7.6 Methods

This section of the review report is reserved for the methods used to conduct the review and should be presented under the relevant subheadings (See Section 2.7.5 points 1-4), including any deviations from the method outlined in the *a priori* protocol. In empty reviews for example, this section should not refer to methods that were not performed.

Directly below the Methods heading provide the following information:

- State and appropriately cite the JBI methodology that was employed in the conduct of the review and synthesis.
- Refer to and cite the *a priori* protocol that was published, or accepted for publication (e.g. 'in press'), in the *JBI Evidence Synthesis*.
- If the protocol has been registered with PROSPERO, provide registration information including registration number (e.g. PROSPERO CRD42015425226).

For peer review only

2.7.6.1 Search strategy

This section details how the reviewers searched for relevant studies. Detailed search strategy for all the sources searched should be appended to the review including record of the dates the searches were conducted. A JBI review should consider papers published by commercial and academic publishers as well as grey literature. The time frame chosen for the search should be justified and any language restrictions stated (e.g. only studies published in English were considered for inclusion).

For peer review only

2.7.6.2 Assessment of methodological quality

This section should detail the approach to critical appraisal, not the assessment results, and should be consistent with the protocol. Any deviations from the protocol must be reported and explained. The report should detail the criteria that were considered when determining the methodological quality of papers considered for inclusion in the review. JBI tools (i.e. JBI-Qualitative Appraisal Instrument) should be used. Critical appraisal tools used ideally should be cited appropriately in the methods section. If a modified tool was used, the *a priori* protocol where it was first presented should be cited.

For peer review only

2.7.6.3 Data extraction

Standardized data extraction tools that promote extraction of similar data form all of the included studies and are recommended. The review should detail what data the reviewers extracted from the included studies. The *a priori* protocol or this Manual with the original data extraction tool can be cited to indicate the tool used. Data extraction in a meta aggregation is a multi phase process, with the general details of papers, including the citations details, the population, phenomena of interest, and context as well as methodology, methods, settings and cultural information retrieved from papers before moving to extraction of the findings. The approach and process used to extract findings from the results of the included studies should be presented with enough detail to be readily reproducible. Indicate what data were considered findings in the review (i.e. themes, metaphors, etc.) and the process by which findings were identified (i.e. repeated reading of text).

Extracting findings is both the second phase of data extraction, and the first step in data synthesis.

- A finding is defined as a verbatim extract of the authors analytic interpretation accompanied by either a participant voice, or fieldwork observations or other data.

Each finding extracted is to be accompanied by an illustration from the same text that informs the finding.

- An illustration may be either a direct quotation of participant voice, field-work observations or other supporting data

Levels of credibility

As a finding is extracted and its accompanying illustration entered in the JBI SUMARI software, a level of 'Credibility' is allocated based on the reviewers perception of the degree of support each illustration offers for the specific finding it is associated with.

There are 3 levels of credibility as described below, and reviewers should document in this section of their review report HOW the decision was made to allocate these levels, and what (if any) issues arose during the process, or whether there was good agreement between the review team members.

- **Unequivocal** (findings accompanied by an illustration that is beyond reasonable doubt and; therefore not open to challenge);
- **Credible** (findings accompanied by an illustration lacking clear association with it and therefore open to challenge)
- **Not Supported** (findings are not supported by the data).

For peer review only

2.7.6.4 Data synthesis

This section should detail the approach to data synthesis, not the results of the synthesis. The review should detail how the reviewers synthesized the data extracted from included studies and detail the meta-aggregative approach and how it was applied across all included studies. . Any deviations from the methods outlined in the protocol need to be clearly documented in the review to maintain transparency.

Data synthesis in a meta aggregative review requires the reviewers to undertake a 3 step process, beginning with:

1. Extraction of all findings from all included papers with an accompanying illustration and establishing a level of credibility for each finding;
2. Developing categories for findings that are sufficiently similar, with at least 2 findings per category;
3. Developing one or more synthesized findings of at least 2 categories.

Please note: Although findings which are not supported should be extracted from studies, they must be presented separately (see Section 2.7.11); they are not included in the meta-aggregation.

Reporting the methods of data synthesis requires reviewers to describe:

- what data was considered 'findings' in their review (i.e. was it limited to themes and metaphors, or did it include other analytic data from the papers that might have been an author observation rather than a thematic analysis);
- the process by which findings were identified (i.e. repeated reading of text, or selection of themes from the results section only;
- how findings were grouped in order to develop categories (i.e. was it based on similarity in wording, or concepts;
- how category descriptions were created (i.e. by single reviewer, or by consenus process between reviewers/review group members);
- how synthesized findings and their accompanying descriptions were created and finalized.

For peer review only

2.7.7 Results

This section of the review report has distinct sub-sections describing the process of study inclusion, the methodological quality of the eligible studies, detailed characteristics and description of the included studies and, importantly, the findings of the review and results of the synthesis processes.

For peer review only

2.7.7.1 Study inclusion

The opening to this section should allow the reader to clearly follow how the included studies were identified and selected for inclusion in the review. There should be a narrative description of the process accompanied by a flowchart (from PRISMA Statement); details to be reported include narrative summary of the numbers of studies identified, numbers screened, studies selected for retrieval and included /excluded and their reasons for exclusion, numbers appraised and included/excluded, numbers included in the qualitative synthesis.

For peer review only

2.7.7.2 Methodological quality

This section should focus on methodological quality as determined by the relevant critical appraisal checklist. There should be a narrative summary of the overall methodological quality of the included studies, which can be supported (optional) by a table showing the results of the critical appraisal (see Table 2.2 for example). Where only few studies are identified, or there are specific items of interest from included studies, these should be addressed in the narrative also, particularly where studies were deficient, or particularly good. Use of Unclear and not applicable should also be explained in the text.

Table 2.2. Critical appraisal results for included studies using the JBI-Qualitative Critical Appraisal Checklist

Study	Q1	Q2	Q3	Q4	Q5	Q6	Q7	Q8	Q9	Q10
Author(s) [ref]	Y	Y	Y	N	Y	U	Y	N	Y	U
Author(s) [ref]	Y	N	Y	Y	Y	U	Y	N/A	Y	Y

Y - Yes, N - No, U - Unclear, N/A - not applicable

If appraisal tools are not appended to the review report (citation only), the appraisal questions should be added as a footnote/caption to the table (Table 2.2) so readers can clearly interpret the information presented.

For peer review only

2.7.7.3 Characteristics of included studies

This section of the results should also include an overall description of the included studies (with reference to the table of included study characteristics in the appendices), with the main aim to provide some context to the results section and sufficient detail for the reader to confirm that the studies match the eligibility criteria for the review and to determine if the included studies are similar enough to combine in meta-synthesis. This includes the descriptive and demographic features (e.g. the country and setting of the study) of the included studies, methodology of included studies, total population size for combined included studies, geographic context of included studies and participant characteristics, characteristics of the interventions, or phenomena of interest as well as the main clinical characteristics, as they relate to the review objective and the inclusion criteria. Specific items/points of interest from individual studies may also be highlighted here and synthesized in narrative.

For peer review only

2.7.7.4 Findings of the review

Review findings or results are preferentially structured according to the phenomena of interest for reviews that include qualitative data. A meta-aggregative schematic/overview flowchart should constitute part of this section.

The meta-aggregative schematic table must be accompanied by sufficient narrative to explain the categories and synthesized findings, and the similarity of meaning informing each category and synthesized finding to the reader of the report.

Findings and illustrations (including their levels of credibility) should be located in an appendix, or may be incorporated into the body of the report. Not-supported findings must not be included in the meta-aggregative synthesis. There should be a logical and informative presentation of the findings, categories and synthesized findings using only credible and unequivocal findings.

Meta-Aggregative Overview Flowchart

Authors can choose to include a meta-aggregative overview flowchart. See below for an example:

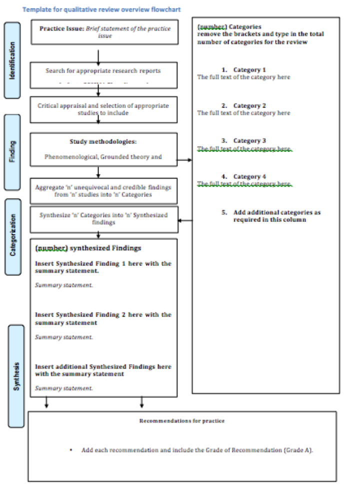

Figure 2.1: Meta-Aggregative Overview Flowchart (Davis et al. 2014)

For peer review only - http://bmjopen.bmj.com/site/about/guidelines.xhtml

2.7.8 Discussion

This section should very briefly summarize and then concentrate on the discussion of the the results of the synthesis as well as any limitations of the primary studies included in the review and of the review itself (i.e. language, access, time frame, study design, etc.). **DO NOT** repeat the results of the review. The results should be discussed in the context of current literature, practice and policy. It will also include a narrative discussion of the review results in comparison with other external literature, and against the broad directions established in the introduction of the review.

This section should also discuss the strength of the evidence (for each main outcome in reviews of effects); any limitations of the included studies (e.g. methodological quality, inconsistencies or errors in reporting, etc.); and any limitations or issues that arose during the conduct of the systematic review itself (e.g. limitations of the search; the impact of deviations from protocol, etc.).

The application and relevance of the findings to relevant stakeholders (e.g. healthcare providers, patients and policy makers) should also be discussed, and where applicable, an indication of whether the findings are generalizable to other populations or healthcare settings.

For peer review only

2.7.9 Conclusions and Recommendations

Conclusions

This section should begin with an overall conclusion based on the results. The conclusions drawn should match with the review objective/question.

Recommendations for practice

The recommendations for practice should be context specific and enable a reader to consider the applicability to practice. E.g. suggesting in a general sense that '…*more education should be provided*…' is not a useful contribution.

Instead, provide direction for a specific type of education on a specified topic for the specific participants. It should be stated how the findings of the review are proposed to impact on clinical practice in the area. If there is sufficient evidence to make specific recommendations for practice, then the appropriate JBI Grade of Recommendation should be assigned to each recommendation.

Recommendations for research

This section should include clear, detailed specific recommendations for future research based on gaps in knowledge identified from the results of the review. The implications for research should not be generic statements on a phenomena of interest without providing specific detail on:

- what phenomena should be investigated,
- the context in which it should be investigated, and
- the specific populations to be considered

By this stage in a systematic review, the international literature on the topic has been comprehensively reviewed, and authors therefore well placed to provide meaningful, researchable recommendations. While drafting implications for research, consider what information you would find helpful if you were planning to do further research on the topic.

For peer review only

2.7.10 Conflicts and acknowledgements

Details of requirements in these sections are described in Section 1.6. of this Manual.

Conflicts of interest

A statement which either declares the absence of any conflicts of interest or which describes a specified or potential conflict of interest should be made by the reviewers in this section.

Funding

Authors should provide details regarding any sources of funding for the review project. The role of all funders in the review process, if any, should be explicitly described. If the review is funded, then any potential conflicts of interest or intellectual bias of the funders should be specified in the review.

Acknowledgements

Any acknowledgements should be made in this section e.g. sources of external funding or the contribution of colleagues or institutions. It should also be noted if the systematic review is to count towards a degree award.

For peer review only

2.7.11 Review appendices

There are several required appendices for a JBI review:

Appendix 1: Search strategy

- A detailed and complete search strategy for all of the major databases and other sites and sources searched must be appended. Major databases that were searched must be identified, including the search platform used where necessary. All search filters with logic employed should be displayed, including the number of records returned.

Appendix 2: Data extraction instrument

- The data extraction instrument used must be appended i.e JBI SUMARI Data Extraction Form for Interpretive & Critical Research.

Appendix 3: List of excluded studies

- Studies excluded following examination of the full-text should be listed along with their reason for exclusion at that stage (i.e. a mismatch with the inclusion criteria). This may be as a separate appendix or itemized in some fashion within the one appendix with those studies excluded at the critical appraisal stage. Reasons for exclusion following appraisal should be provided for each study (these reasons should relate to the methodological quality of the study, not study eligibility).

Appendix 4: Table of included study characteristics

- A table of included studies is required to provide quick reference to important details extracted from of the studies included in the review.

Appendix 5: Findings and illustrations

- An appendix presenting findings and their supporting illustrations (Not-supported findings must be included in this appendix, but must have ‘Not-Supported’ in place of the illustration) with levels of credibility and their citation/reference should be provided if this material is not already presented in the body of the review report.

For peer review only

2.8 Chapter references

Ailinger, R. Contributions of qualitative evidence to evidence based practice in nursing. Revista Latino-americana de Enfermagem. 2003, 11(3): 275-279.

Barbour, R. The case for combining qualitative and quantitative approaches in health services research." Journal of Health Service Research Policy 1999, 4(1): 39-43.

Black, N. Why we need qualitative research. Journal of Epidemiological Community Health 1994, 48: 425-426.

Chin, P. Jacobs, M. Theory in Nursing: A Systematic Approach. St Louis, Mosbey, 1987.

CRD. Systematic Reviews. CRD's guidance for undertaking reviews in health care. Centre for Reviews and Dissemination, University of York, 2009.

Davis K, White S, Stephenson M. The influence of workplace culture on nurses' learning experiences: a systematic review protocol of qualitative evidence. The JBI Database of Systematic Reviews and Implementation Reports, 2014, (12):45 – 58.

Denzin N, Lincoln Y. Eds. Handbook of qualitative research. California, Thousand Oaks; SAGE publications, 2005.

Evans, D. Database searches for qualitative research. Journal of the Medical Libraries Association 2005, 13(3): 290-293.

Forman J, Damschroder L, Kowalski C, Krein S. Qualitative research methods: key features and insights gained from use in infection prevention research." American Journal of Infection Control 2008, 36(10): 764-771.

Greenhalgh T. Papers that go beyond numbers (qualitative research). BMJ 1997, 315(7110): 740-743.

Hannes K, Lockwood. C. Pragmatism as the philosophical foundation for the Joanna Briggs meta-aggregative approach to qualitative evidence synthesis. Journal of Advanced Nursing 2011, 67(7): 1632-1642.

Jordan Z, Pittman P. A short history of a big idea. Melbourne, The Joanna Briggs Institute, 2006.

Moffatt S, Mackintosh J, Howel D. Using quantitative and qualitative data in health services research - what happens when mixed method findings conflict? BMC Health Services Research 2006 6: 28.

Munn Z, Porritt K, Lockwood C, Aromataris E, Pearson A. Establishing confidence in the output of qualitative research synthesis: the ConQual approach. *BMC Medical Research Methodology*, 2014, 14: 108

Pearson, A. Balancing the evidence: incorporating the synthesis of qualitative data into systematic reviews. JBI Report 2004, 2(2): 45-65.

Rittenmeyer L, Block M, Mathaler M, Misner S, Moore E, Wegnere G, Fleefisch K A comprehensive systematic review on lateral/horizontal violence in the profession of nursing. JBI Database of Systematic Reviews and Implementation Reports 2012, 10(42): 17.

Sandelowski M, Barroso J. Handbook for Synthesizing Qualitative Research. New York, Springer Publishing Company, 2007.

Sandelowski M, Docherty, Emden C. Focus on qualitative methods. Qualitative meta synthesis: issues and techniques." Research in Nursing and Health 1997, 20: 365-371.

Thorne S, Kearney MH, Noblit G, Sandelowski M. Qualitative meta synthesis: reflections on methodological orientation and ideological agenda." Qualitative Health Research 2004, 14(10): 1342-1365.

Wong S, Haynes R. Developing optimal search strategies for detecting clinically relevant qualitative studies in Medline. Stud Health Technol Inform 2004, 107(1): 311-316.

For peer review only

Appendix 2.1: JBI Critical Appraisal Checklist for Qualitative Research

JBI Critical Appraisal Checklist for Qualitative Research

Reviewer____________________Date____________________

Author ____________________Year________Record Number______

	Yes	No	Unclear	Not applicable
1. Is there congruity between the stated philosophical perspective and the research methodology?	☐	☐	☐	☐
2. Is there congruity between the research methodology and the research question or objectives?	☐	☐	☐	☐
3. Is there congruity between the research methodology and the methods used to collect data?	☐	☐	☐	☐
4. Is there congruity between the research methodology and the representation and analysis of data?	☐	☐	☐	☐
5. Is there congruity between the research methodology and the interpretation of results?	☐	☐	☐	☐
6. Is there a statement locating the researcher culturally or theoretically?	☐	☐	☐	☐
7. Is the influence of the researcher on the research, and vice- versa, addressed?	☐	☐	☐	☐
8. Are participants, and their voices, adequately represented?	☐	☐	☐	☐
9. Is the research ethical according to current criteria or, for recent studies, and is there evidence of ethical approval by an appropriate body?	☐	☐	☐	☐
10. Do the conclusions drawn in the research report flow from the analysis, or interpretation, of the data?	☐	☐	☐	☐

Overall appraisal: Include ☐ Exclude ☐ Seek further info ☐

Comments (Including reason for exclusion)

For peer review only

Appendix 2.2: Discussion of JBI Qualitative critical appraisal criteria

1. Congruity between the stated philosophical perspective and the research methodology

Does the report clearly state the philosophical or theoretical premises on which the study is based? Does the report clearly state the methodological approach adopted on which the study is based? Is there congruence between the two? For example:

A report may state that the study adopted a critical perspective and participatory action research methodology was followed. Here there is congruence between a critical view (focusing on knowledge arising out of critique, action and reflection) and action research (an approach that focuses on firstly working with groups to reflect on issues or practices, then considering how they could be different; then acting to create a change; and finally identifying new knowledge arising out of the action taken). However, a report may state that the study adopted an interpretive perspective and used survey methodology. Here there is incongruence between an interpretive view (focusing on knowledge arising out of studying what phenomena mean to individuals or groups) and surveys (an approach that focuses on asking standard questions to a defined study population); a report may state that the study was qualitative or used qualitative methodology (such statements do not demonstrate rigor in design) or make no statement on philosophical orientation or methodology.

2. Congruity between the research methodology and the research question or objectives

Is the study methodology appropriate for addressing the research question? For example:
A report may state that the research question was to seek understandings of the meaning of pain in a group of people with rheumatoid arthritis and that a phenomenological approach was taken. Here, there is congruity between this question and the methodology. A report may state that the research question was to establish the effects of counselling on the severity of pain experience and that an ethnographic approach was pursued. A question that tries to establish cause-and effect cannot be addressed by using an ethnographic approach (as ethnography sets out to develop understandings of cultural practices) and thus, this would be incongruent.

3. Congruity between the research methodology and the methods used to collect data

Are the data collection methods appropriate to the methodology? For example:

A report may state that the study pursued a phenomenological approach and data was collected through phenomenological interviews. There is congruence between the methodology and data collection; a report may state that the study pursued a phenomenological approach and data was collected through a postal questionnaire. There is incongruence between the methodology and data collection here as phenomenology seeks to elicit rich descriptions of the experience of a phenomena that cannot be achieved through seeking written responses to standardized questions. There is congruity between the research methodology and the representation and analysis of data.

4. Congruity between the research methodology and the representation and analysis of data

Are the data analyzed and represented in ways that are congruent with the stated methodological position? For example:

A report may state that the study pursued a phenomenological approach to explore people's experience of grief by asking participants to describe their experiences of grief. If the text generated from asking these questions is searched to establish the meaning of grief to participants, and the meanings of all participants are included in the report findings, then this represents congruity; the same report may, however, focus only on those meanings that were common to all participants and discard single reported meanings. This would not be appropriate in phenomenological work.

5. There is congruence between the research methodology and the interpretation of results

Are the results interpreted in ways that are appropriate to the methodology? For example:

A report may state that the study pursued a phenomenological approach to explore people's experience of facial disfigurement and the results are used to inform practitioners about accommodating individual differences in care. There is congruence between the methodology and this approach to interpretation; a report may state that the study pursued a phenomenological approach to explore people's experience of facial disfigurement and the results are used to generate practice checklists for assessment. There is incongruence between the methodology and this approach to interpretation as phenomenology seeks to understand the meaning of a phenomenon for the study participants and cannot be interpreted to suggest that this can be generalized to total populations to a degree where standardized assessments will have relevance across a population.

6. Locating the researcher culturally or theoretically

Are the beliefs and values, and their potential influence on the study declared? For example:

For peer review only

The researcher plays a substantial role in the qualitative research process and it is important, in appraising evidence that is generated in this way, to know the researcher's cultural and theoretical orientation. A high quality report will include a statement that clarifies this.

7. Influence of the researcher on the research, and vice-versa, is addressed

Is the potential for the researcher to influence the study and for the potential of the research process itself to influence the researcher and her/his interpretations acknowledged and addressed? For example:

Is the relationship between the researcher and the study participants addressed? Does the researcher critically examine her/his own role and potential influence during data collection? Is it reported how the researcher responded to events that arose during the study?

8. Representation of participants and their voices

Generally, reports should provide illustrations from the data to show the basis of their conclusions and to ensure that participants are represented in the report.

9. Ethical approval by an appropriate body

A statement on the ethical approval process followed should be in the report.

10. Relationship of conclusions to analysis, or interpretation of the data

This criterion concerns the relationship between the findings reported and the views or words of study participants. In appraising a paper, appraisers seek to satisfy themselves that the conclusions drawn by the research are based on the data collected; data being the text generated through observation, interviews or other processes.

For peer review only

Appendix 2.3: JBI Qualitative data extraction tool

JBI QARI Data Extraction Tool for Qualitative Research

Reviewer ____________________ Date ____________________

Author ____________________ Year ____________________

Journal ____________________ Record Number ____________________

Study Description

Methodology

Method

Phenomena of interest

Setting

Geographical

Cultural

Participants

Data analysis

Authors conclusions

Comments

Complete Yes ☐ No ☐

Copyright © The JoAnna Briggs Institute 2014

Findings	Illustration form Publication (page number)	Evidence		
		Unequivocal	Credible	Unsupported

Extraction of findings complete Yes ☐ No ☐

Copyright © The JoAnna Briggs Institute 2014

For peer review only

Chapter 3: Systematic reviews of effectiveness

Catalin Tufanaru, Zachary Munn, Edoardo Aromataris, Jared Campbell, Lisa Hopp

How to cite:

Tufanaru C, Munn Z, Aromataris E, Campbell J, Hopp L. Chapter 3: Systematic reviews of effectiveness. In: Aromataris E, Munn Z (Editors). *JBI Manual for Evidence Synthesis.* JBI, 2020. Available from https://synthesismanual.jbi.global. https://doi.org/10.46658/JBIMES-20-04

Chapter 3: Contents

- 3.1 Introduction to quantitative evidence and evidence-based practice
- 3.2 Development of a protocol for a systematic review of effectiveness evidence
  - 3.2.1 Title of the systematic review protocol
  - 3.2.2 Review question(s)
  - 3.2.3 Introduction
  - 3.2.4 Inclusion criteria
    - 3.2.4.1 Population (types of participants)
    - 3.2.4.2 Intervention (types of interventions)
    - 3.2.4.3 Comparison (types of comparators)
    - 3.2.4.4 Outcomes
    - 3.2.4.5 Types of studies
  - 3.2.5 Search strategy
  - 3.2.6 Selection of studies
  - 3.2.7 Critical appraisal
  - 3.2.8 Data extraction
  - 3.2.9 Data synthesis
- 3.3 Meta-analysis
  - 3.3.1 Objectives of meta-analysis
  - 3.3.2 Statistical models for meta-analysis
  - 3.3.3 Effect sizes
  - 3.3.4 Considerations for the meta-analysis of dichotomous data
  - 3.3.5 Considerations for the meta-analysis of continuous data
  - 3.3.6 Meta-analysis: Statistical Methods
  - 3.3.7 Subgroups in meta-analysis
  - 3.3.8 Sensitivity analysis in meta-analysis
  - 3.3.9 Meta-regression
  - 3.3.10 Heterogeneity
    - 3.3.10.1 Standard chi-squared test (Cochran test)
    - 3.3.10.2 Quantification of the statistical heterogeneity: I squared
    - 3.3.10.3 Tau-squared for random effects model meta-analysis
  - 3.3.11 Publication bias
- 3.4 Systematic review of effectiveness
  - 3.4.1 Title
  - 3.4.2 Abstract
  - 3.4.3 GRADE 'Summary of Findings' table
  - 3.4.4 Introduction
  - 3.4.5 Review question(s)
  - 3.4.6 Inclusion criteria
  - 3.4.7 Methods
    - 3.4.7.1 Search strategy
    - 3.4.7.2 Study screening and selection
    - 3.4.7.3 Critical appraisal
    - 3.4.7.4 Data extraction
    - 3.4.7.5 Data synthesis
  - 3.4.8 Results
    - 3.4.8.1 Study inclusion
    - 3.4.8.2 Methodological quality
    - 3.4.8.3 Characteristics of included studies
    - 3.4.8.4 Results and meta-analysis
  - 3.4.9 Discussion
  - 3.4.10 Conclusions and recommendations
  - 3.4.11 Conflicts and acknowledgements
  - 3.4.12 Review Appendices
- 3.5 Chapter References
- Appendix 3.1: JBI Critical appraisal checklist for randomized controlled trials
- Appendix 3.2: Discussion of JBI appraisal criteria for randomized controlled trials
- Appendix 3.3: JBI Critical appraisal Checklist for Quasi-Experimental Studies (non-randomized experimental studies)
- Appendix 3.4: Discussion of JBI appraisal criteria for Quasi-Experimental Studies (non-randomized experimental studies)

For peer review only

3.1 Introduction to quantitative evidence and evidence-based practice

Quantitative evidence is generated by research based on traditional scientific methods that generate numerical data. The methods associated with quantitative research in healthcare have developed out of the study of natural and social sciences. It was suggested that quantitative evidence in medicine originated in eighteenth century Britain, when surgeons and physicians started using statistical methods to assess the effectiveness of therapies for scurvy, dropsy, fevers, palsies, syphilis, and different methods of amputation and lithotomy (Trohler 2000). Since these beginnings, quantitative research has expanded to encompass aspects other than effectiveness, such as incidence, prevalence, etiology of disease, psychometric properties, and measurement of physical characteristics, quality of life, and satisfaction with care.

JBI quantitative reviews focusing on evidence of effectiveness examine the extent to which an intervention, when used appropriately, achieves the intended effect. Evidence about the effects of interventions may come from three main categories of studies: experimental studies, quasi-experimental studies and observational studies. Ideally, evidence about the effectiveness of interventions should come from good quality randomized controlled trials (RCTs) that explore final clinical end points (or patient important outcomes) such as morbidity, mortality, and quality of life (not surrogate end points which may include laboratory tests for example) (Brignardello-Petersen et al 2015). Good empirical evidence exists to indicate that RCTs that explored final clinical end points frequently contradicted (refuted) clinical studies that explored surrogate end points and also the results of observational studies (Brignardello-Petersen et al 2015). Some authors have claimed that results from RCTs and observational studies provide consistent results. Thus, the issue of the agreement of the results from RCTs and observational studies remains controversial (Brignardello-Petersen et al 2015).

Although high quality RCTs exploring final clinical end points are considered the “reference standard” (Brignardello-Petersen et al 2015), reviewers should be aware that results from any single RCT cannot be considered as “final” because results from new RCTs may contradict results from previous RCTs (Brignardello-Petersen et al 2015).

Reviewers should be aware that there is no unique universally accepted terminology for the quantitative study designs. Also, there is no unique comprehensive set of descriptions for the different study designs considered here.

Experimental studies meet three conditions: manipulation, control and random assignment. Specifically, the researchers manipulate the intervention of interest and the control condition and they randomly allocate the participants to the intervention or control group (Shadish et al 2002). Random allocation refers to an authentically random process such as the toss of a coin or use of a table of random numbers (Shadish et al 2002). Randomized controlled trials with different designs (parallel design, cross-over design, cluster design) are examples of experimental studies. There are also existing experimental studies (the intervention of interest and the control condition are manipulated by the researchers) where the allocation may not use an authentically random process. For example, if investigators use alternate group allocation like even and odd dates, they cannot ensure that each participant has an equal chance of landing in either group. Experimental studies without authentic random allocation but using systematic alternate group allocation methods mentioned above are experimental studies with pseudo-randomization, or pseudo-RCTs. Quasi-experimental studies are studies where the intervention of interest and the control condition are controlled (manipulated) by the researchers, however, the allocation of participants is not a random, systematic or pseudo-random allocation (Shadish et al 2002). Frequently, participants self-select into groups or the researchers decide which persons should get the intervention and which persons should get the control (Shadish et al 2002).

Observational studies are studies where the intervention of interest and the control condition are not controlled (manipulated) by the researchers and where researchers only observe the presence or absence of the intervention of interest and of the outcome of interest. There are diverse types of observational studies, which can be broadly categorized into analytical observational studies (cohort studies, case-control studies, and analytical cross-sectional studies) and descriptive observational studies (case reports and case series). In a cohort study, investigators select participants based on presence or absence of exposure to an intervention of interest and compare prospectively for the occurrence of the outcome of interest. In a case-control study, researchers select “case” participants or those with the outcome of interest and “control” participants, without the outcome of interest, to compare groups for past exposure or absence of exposure to the intervention. In an analytical cross-sectional study, investigators select participants without reference to the intervention or the presence of the outcome of interest. They then simultaneously examine the groups for the presence or absence of exposure to the intervention of interest and the presence or absence of the outcome of interest. In case reports and case series researchers simply describe the characteristics of participants and the outcomes of interventions.

For peer review only - http://bmjopen.bmj.com/site/about/guidelines.xhtml

3.2 Development of a protocol for a systematic review of effectiveness evidence

An *a priori* systematic review protocol is important because it pre-defines the objectives and methods of the systematic review. A review protocol provides the plan or proposal for the systematic review. Any deviations from the review protocol should be discussed in the systematic review report.

The review protocol describes:

- the context and rationale for the review, including what is already known and uncertainties,
- the study selection criteria (inclusion/exclusion criteria),
- the outcome measures, interventions, and comparisons considered,
- the proposed search strategy for identifying relevant studies,
- the procedures for study selection,
- the critical appraisal process and instruments,
- the data extraction process and instruments,
- the process for resolving disagreement between reviewers in study selection, data extraction, and critical appraisal decisions, and
- the proposed approaches to synthesis

For peer review only

3.2.1 Title of the systematic review protocol

A clear, descriptive title is important to allow readers and users to readily identify the scope and relevance of the review. The clearer and more specific a title is, the more readily a reader will be able to make decisions about the potential relevance of the systematic review. The protocol title should accurately describe and reflect the content of the review protocol and include relevant information with regards the types of participants, types of interventions and comparators and the outcomes considered in the review. The title should be concise and should not be phrased as a question. The title of the review protocol should explicitly identify the publication as a protocol for a systematic review. The following convention is recommended: ‘a protocol for a systematic review’. Following the guidance mentioned, for systematic reviews of effectiveness we recommend the following convention: *'The effectiveness of [intervention] compared to [comparator] on [outcome]: a protocol for a systematic review'*.

For peer review only

3.2.2 Review question(s)

The review protocol should provide an explicit and clear statement of the review questions addressed in the review. The review questions should specify the focus of the review (effectiveness), the types of participants, types of interventions and comparators, and the types of outcomes considered. Usually, reviewers use the PICO mnemonic (population, intervention, comparator and outcome) to construct a clear and meaningful review objective/question regarding the quantitative evidence on effectiveness of interventions.

Examples of review questions: *'In community dwelling patients with stable, moderate-to-severe chronic obstructive pulmonary disease'*:

1. *What is the effect of inspiratory muscle training versus no specific training on dyspnea and functional ability?*
2. *What is the effect of inspiratory muscle training versus no specific training on inspiratory muscle strength and endurance?*
3. *What is the effect of inspiratory muscle training on hypoxemia and discomfort?*

There should be consistency between the review title and the review questions in terms of the focus of the review. Review authors are encouraged to read the article by Stern et al (2014) regarding the review questions and the inclusion criteria.

For peer review only

3.2.3 Introduction

The introduction of the review protocol should provide explicit and comprehensive information regarding the justification (rationale) for the conduct of the review in the context of what is already known. The introduction should be of sufficient length to discuss all of the elements of the proposed plan for the review; usually all the relevant information may be provided in approximately 1000 words. This section should be written in simple prose for non-expert readers. Usually, a systematic review is informed by international research and is conducted for an international readership, therefore, reviewers should include relevant international literature in this introductory section. There are exceptions, for example, where systematic reviews are conducted on a question relevant to a single country (for example, Australia or UK) or region (Africa) specific issues. However, with the exception of these reviews that use strict limitations on the inclusion criteria, a systematic review should include all relevant international literature. The introduction should provide sufficient details to justify the conduct of the review and the choice of inclusion criteria for the review (types of participants, types of interventions and comparators, the types of outcomes, and types of studies). The review protocol should provide all conceptual and operational definitions that are relevant for the review. It is the responsibility of the reviewers to ensure that their review is not a duplicate of an existing review. It is recommended that reviewers search major electronic databases to determine that there have been no recently published systematic reviews on the same topic. A search of the *JBI Evidence Synthesis*, Cochrane Database, MEDLINE, DARE, PROSPERO, EPISTEMONIKOS, and ACCESSSS will assist to establish whether or not a recent review exists on the topic of interest. Reviewers should report in the background section the details of this preliminary search. If systematic reviews on the topic of interest have already been conducted, reviewers should explain the differences between the existing reviews and the new proposal and provide an explicit justification for the need to conduct a new systematic review.

The introduction should conclude with an overarching review objective that captures and aligns with the core elements/mnemonic of the inclusion criteria (e.g. PICO). The stated objective should clearly indicate what the review project is trying to achieve. Example of a review objective: *'To synthesize the best available evidence related to using inspiratory muscle training to improve dyspnoea in patients with chronic obstructive pulmonary disease.'* This broad statement provides the general scope but must be further clarified with focused review questions.

The background section of the review protocol should provide information regarding:

- the importance of the topic (prevalence, incidence, morbidity, mortality, impact on quality of life; economic burden),
- concerns expressed by consumers, healthcare professionals, policy-makers,
- the specifics of diverse groups of patients (age, gender, ethnicity, severity of the disease, co-existing diseases) and settings,
- the intervention of interest and how it works,
- any uncertainties and conflicting reports regarding the effectiveness of the intervention of interest,
- other existing interventions with which the intervention of interest may be compared,
- the importance of different outcomes,
- how outcomes are measured (approaches, measurement instruments),
- the relevance of different research study designs in the examination of the topic of interest,
- relevant existing primary research studies,
- what is already known, including details about the existing systematic reviews, including meta-analyses, and
- the justification for the need for a new review and the objectives of the review project.

For peer review only

3.2.4 Inclusion criteria

The review protocol should provide explicit, unambiguous, inclusion criteria for the review. Inclusion criteria should be reasonable, sound (based on scientific arguments), and justified. These criteria will be used in the selection process, when it is decided if a study will be included or not in the review. Usually, it is enough to provide explicit inclusion criteria without specifying explicit exclusion criteria; it is implicitly assumed that exclusion is based on the criteria that are the opposite of those specified as inclusion criteria. However, sometimes, for clarity, in order to avoid any potential ambiguity, it is recommended to provide explicit exclusion criteria. Inclusion criteria for a review are not intended to be considered in isolation; in this regard they should be articulated so as to be as mutually exclusive as possible and not repeat information relevant to other aspects of the PICO.

Two categories of inclusion criteria should be considered: *inclusion criteria based on study characteristics* , and *inclusion criteria based on publication characteristics*. *Inclusion criteria based on study characteristics* are those related to the types of participants and settings, types of interventions, comparators, types and measurement of outcomes, and types of studies. *Inclusion criteria based on publication characteristics* are those related to publication date, language of publication, type of publication (published in commercial scientific databases; documents not published in commercial databases, for example, trials documents). Usually, reviewers use the PICO mnemonic (participants, intervention, comparator and outcome) to construct a clear and meaningful review objective/question regarding the quantitative evidence on effectiveness of interventions. The reviewer uses the same PICO framework to develop inclusion criteria based on study characteristics. The inclusion criteria must provide adequate details about the conceptual and operational definitions of each element to enable reviewers to make reliable decisions when making decisions to include studies.

For peer review only

3.2.4.1 Population (types of participants)

This section should specify the details about types of participants considered for the review, for example, age; gender; ethnicity; diagnosis; diagnostic criteria; stage or severity of the disease; co-existing diseases. What are the most important characteristics of the population? (e.g., age, disease/condition, severity of illness, setting, gender, etc.).

Consider the following example regarding COPD, describe the population (*patients with COPD*), the severity of illness (*moderate-to-severe*), trajectory of the disease (*stable*), with a specific setting (*communi ty dwelling*). Diagnostic criteria should be made clear to allow inclusion and exclusion; if reviewers anticipate subgroup analysis related to population characteristics, these subgroups should be reflected in the population inclusion criteria. For example, '*COPD includes patients with chronic bronchitis and emphysema but not asthma (fixed airway obstruction with forced expiratory volume in one second [FEV_1] less than <80% of predicted). According to the Global Initiative for Chronic Obstructive Lung Disease (GOLD) and the American Thoracic/European Respiratory Society Guidelines (ATS/ERS), the description of the severity of disease is as follows: stage II or moderate disease is an FEV_1 of 50-80% predicted; stage III or severe is an FEV_1 of 30-50% predicted and stage IV or very severe is an FEV_1 <30% predicted. Patients with reversible airway disease (improvement in FEV_1 >20% with fast acting bronchodilator) will be excluded because their response to training may relate more to changes in their airway obstruction than a training effect*.' Specific reference to population characteristics, either for inclusion or exclusion should be based on a clear, scientific justification rather than based on unsubstantiated clinical, theoretical or personal reasoning.

For peer review only

3.2.4.2 Intervention (types of interventions)

What is the intervention? This section should specify the details about the intervention of interest for the review, for example, the nature of intervention, frequency, intensity, timing, and details about those administering the intervention. The same kind of information should be specified for all comparators considered in the review. Where possible, the intervention should be described in detail, particularly if it is multifaceted. A more detailed analytical framework can be used to refer to these complexities. If the review is examining a class or group of interventions, a comprehensive list of identified examples should be provided for the reader. Reviewers should plan any subgroup analysis based on different modes, timing, etc. of the intervention during the protocol stage and account for them in the inclusion criteria. For example, '*inspiratory muscle training includes any mode (threshold loading, resistive, hyperpneic,) practiced at least daily for no less than 4 weeks'* allows the reviewers to consider different types of training but specifies the minimum training period.

For peer review only

3.2.4.3 Comparison (types of comparators)

What is the intervention being compared with? (e.g., placebo, standard care, another therapy or no treatment). This section should detail what the intervention of interest is being compared with. The reviewer may wish to examine the comparative effectiveness of two treatments with a specific, head-to-head comparison. In the example (See Section 3.2.4.3), the reviewers may have specified inspiratory muscle training compared to cardiovascular conditioning. This level of detail is important in determining study selection once searching is complete. Systematic reviews of effectiveness based on the inclusive definition of evidence adopted by the JBI often seek to answer broader questions about multifaceted interventions and comparing the intervention of interest with all existing alternative interventions (comparators).

For peer review only

3.2.4.4 Outcomes

The review protocol should list all the outcomes considered. There is an international initiative known as The COMET (Core Outcome Measures in Effectiveness Trials) initiative, involved in the development and application of agreed standardized sets of outcomes for trials on specific conditions. Details are provided on the COMET website (http://www.comet-initiative.org/). Reviewers are encouraged to check the available standardized sets of outcomes for trials relevant for their reviews.

Outcomes should be measurable and appropriate to the review objectives and questions. Usually, only a limited number of primary outcomes and a limited number of secondary outcomes are considered for a review. Sometimes, if justified, it is acceptable to include multiple primary and secondary outcomes. However, the appropriateness of the number and scope of outcomes depend on the specifics of the review objectives and review questions (Aromataris 2015). The relevance of each outcome to the review objective/questions should be justified in the background section. Both beneficial outcomes (positive effects) and harms (negative effects, such as adverse effects or side effects) should be considered as outcomes (Aromataris 2015). Essentially, primary outcomes are those outcomes that are the most important outcomes informing the review questions and the conclusions about the beneficial and harmful effects of the intervention of interest for a review (Aromataris 2015). Secondary outcomes are all other outcomes not specified as primary outcomes. A fundamental distinction is that between true endpoints and surrogate outcomes; true endpoints reflect the effects of treatment on aspects of patients' status considered the most important in terms of mortality and morbidity; surrogate outcomes are measured as "surrogates' for true endpoints, for reasons related to complexity, time, and costs of measurement of true endpoints (Tufanaru 2016). Examples of true endpoints are survival time in cancer and bone fractures in osteoporosis; examples of surrogate outcomes are time to progress from one stage to another stage in cancer and bone mineral density in osteoporosis (Tufanaru 2016).

It is recommended that whenever possible true endpoints should be used as primary outcomes, and that if surrogate outcomes are used as primary outcomes then an explicit justification should be provided for the use of a surrogate outcomes instead of true endpoints (Tufanaru 2016). It is expected that all outcomes specified *a priori* in the review protocol, will be explicitly addressed in the systematic review report, regardless of the existence or not of data from included studies on these outcomes (Aromataris 2015).

A further critical aspect refers to the measurement of the specified outcomes. It is recommended that reviewers present explicit information on available measurement instruments, including details about the validity and reliability properties of these instruments (Aromataris 2015).

As JBI endorses the use of the GRADE approach known as the 'Summary of findings' table, reviewers should be aware that the most important outcomes, that is, the primary outcomes specified in the review protocol should be addressed in the review report and should be explicitly presented in the GRADE Summary of findings' table. Details are provided in the GRADE Handbook (Schunnemann et al. 2013).

For peer review only

3.2.4.5 Types of studies

There are three approaches regarding choices for inclusion of studies based on their design in JBI systematic reviews. The first option is to clearly state in the protocol what study designs will be included (for example RCTs), and include only studies that are of this design in the review. This approach is transparent and at low risk of subjectivity during selection of studies. However, it runs the risk of leading to an empty review or a review with few included studies.

The second option is to consider using the hierarchy of study designs for including and excluding studies in the review. In this approach, authors may include other study designs if their preferential study designs are not located. If this is the case, there should be a statement about the primary study design of interest and the other types of studies that will be considered if primary study design of interest is not found. It is common to provide a statement that RCTs will be sought, and that in the absence of RCTs, other study designs will be included, such as quasi-experimental studies and observational studies. This is a pragmatic approach with the aim to include the best available evidence within a review.

The third option is to simply include all quantitative study designs (or all study designs up to a point of the hierarchy of evidence - for example experimental studies and cohort studies, both prospective and retrospective).. This inclusive approach is acceptable as it allows for examination of the totality of empirical evidence and may provide invaluable insights regarding the agreement or disagreement of the results from different study designs. Where feasible, JBI prefers and suggests reviewers consider option 3, the most inclusive approach. However, for many topics, this will present a great deal of information which may not be of use to best inform effectiveness.

For peer review only

3.2.5 Search strategy

This section of a review protocol should provide explicit and clear information regarding two different aspects of locating studies: *all information sources* that will be searched for the review, and the *strategies used for searching*. The aim of a systematic review is to identify all relevant studies, published or not, on a given topic. Searching should be based on the principle of comprehensiveness, with the widest reasonable collection of information sources that are considered appropriate to the review.

A systematic review of effectiveness aims to identify, at a minimum (see Section 3.2.4.5) all data derived from experimental trials (published or not) performed on a specific topic. Two recent international initiatives, one called 'All Trials' (http://www.alltrials.net/), and the other one called Restoring invisible and Abandoned Trials abbreviated RIAT (http://www.bmj.com/content/346/bmj.f2865) are fundamental in this regard.

The review protocol should list all information sources that will be used in the review: electronic bibliographic databases; search engines; trials registers; specific relevant journals; websites of relevant organizations; direct contact with researchers; direct contact with sponsors and funders of clinical trials; contact with regulatory agencies (for example, US FDA). The review protocol, ideally, should specify all the details (a line-by-line description) of the proposed search strategy used for each electronic bibliographic database considered for the review. As a minimum, all the details of the proposed search strategy for at least one major electronic bibliographic database (such as PubMed) should be provided in an appendix. The review protocol should specify the timeframe for search, and any language and date restrictions, with appropriate justifications. The reviewers should consider the potential consequences of language and date search restrictions. If possible, authors should always seek the advice of an expert research librarian when developing a search strategy. Involvement of a research librarian in the development of a search strategy should be acknowledged. For JBI systematic reviews, the search strategy is often described as a three-phase process beginning with the identification of initial key words that are used in a limited number of databases (for example, PubMed and CINAHL); followed by an analysis of the text words contained in the title, abstract and index terms used to describe relevant articles. The second phase consists of the use of database-specific searches for each database specified in the review protocol. The third phase includes the examination of the reference lists of all studies already retrieved with the explicit aim to identify additional relevant studies. The list of all databases that will be considered for database-specific searches should be provided. Usually, a comprehensive search for a review of effectiveness includes a search of relevant multiple bibliographic databases (for example, PubMed, CINAHL, EMBASE etc.), a search of trial registers, a search of relevant grey literature sources, and a hand-search of relevant journals. Reviewers should provide enough information in order to persuade readers that the sources of information considered are relevant and comprehensive and the search strategy is comprehensive and sound. Reviewers are encouraged to read the article by Aromataris and Riitano (2014) regarding searching for evidence.

For peer review only

3.2.6 Selection of studies

This section should describe the process of study inclusion for all stages of selection (based on title and abstract examination; based on full text examination) and the procedures for solving disagreements between reviewers. The software used for the management of the results of the search should be specified (e.g. Covidence, Endnote). Selection is performed based on inclusion criteria (See Section 3.2.4) pre-specified in the review protocol. In a systematic review study selection (both at title/abstract screening and full text screening) should be performed by two or more reviewers, independently. Any disagreements are solved by consensus or by the decision of a third reviewer. JBI reviewers are encouraged to read the article by Porritt et al (2014) regarding study selection and critical appraisal.

For peer review only

3.2.7 Critical appraisal

This section should describe the critical appraisal process and instruments that will be used in the review process and the procedures for solving disagreements between reviewers.

The goal of critical appraisal (assessment of risk of bias) is to assess the methodological quality of a study and to determine the extent to which a study has excluded or minimized the possibility of bias in its design, conduct and analysis. Bias refers to systematic errors in the design, conduct and analysis of quantitative studies that may impact the validity of inferences from these studies. Critical appraisal of the studies included in a systematic review is performed with the explicit goal of identifying the risk of diverse biases in these studies. JBI uses standardized critical appraisal tools for the assessment of risk of diverse biases encountered in quantitative studies. There are JBI standardized appraisal tools based on study design appropriate for JBI reviews of effectiveness (see Appendix 3.2 regarding the JBI standardized appraisal tools). JBI systematic reviews are required to use these JBI standardized appraisal tools.Reviewers should refer in the review protocol to the JBI standardized critical appraisal checklists and provide references for these checklists. It is not necessary to provide these checklists in appendices of the review protocol. If non-JBI appraisal tools are proposed then these tools should be briefly described and correctly referenced. In this case, an explicit justification for the use of non-JBI appraisal tools should be provided in the review protocol.

Two reviewers should perform independent critical appraisal of retrieved studies using the standardized critical appraisal checklists developed by JBI. The protocol should specify that any disagreements are solved by consensus or by the decision of a third reviewer. In experimental studies (randomized experimental studies and quasi-experimental studies) the most important biases are: selection bias, performance bias, attrition bias, detection bias, and reporting bias. In observational studies the most important biases are: selection bias, information bias, and confounding. The review protocol should specify that reviewers plan to report in narrative form and in tables the results of risk of bias (methodological quality) assessments for each aspect of methodological quality (randomization; blinding; measurement; statistical analysis etc.) for each individual study and the overall risk of bias of the entire set of included studies. The critical appraisal phase of the review should not be treated as a rapid ‘box ticking exercise’ on checklists, but rather as a complex, profound, critical, systematic, thorough examination of the risk of bias of each included study, a solid foundation for an appropriate synthesis of the results.

The review protocol should specify if and how the results of critical appraisal will be used for the exclusion of studies from the review. For example, if studies judged of low methodological quality will be excluded from the review, the details of the circumstances under which such decisions will be made and the explicit criteria or decision rules should be explicitly provided, including explanations for what is considered low methodological quality by reviewers. It is the decision of the review team if they want to exclude from the review studies judged of low methodological quality. Reviewers should explain and justify their criteria and decision rules. The decision as to whether or not to include a study can be made based on meeting a predetermined proportion of all criteria, or on certain criteria being met. It is also possible to weight the different criteria differently. The decisions about the scoring system and the cut-off for inclusion of a study in the review should be made in advance and be agreed upon by all participating reviewers before critical appraisal commences. The review protocol should specify if and how the results of critical appraisal will be used in the synthesis (narrative synthesis or meta-analysis) of the results. It is recommended that the results of critical appraisal should be used in the synthesis phase of the review, for the critical examination of the impact of methodological quality of studies on results (including subgroup analysis or sensitivity analysis). JBI reviewers are encouraged to read the article by Porritt et al (2014) regarding study selection and critical appraisal.

For peer review only

3.2.8 Data extraction

This section of the review protocol should specify the data extraction process and instruments that will be used in the review process, as well as the procedures for solving disagreements between reviewers. Complete and accurate data extraction is essential for a good quality systematic review. Reviewers should carefully consider all the relevant data that should be extracted for the review given the focus of the review, the review objectives/questions, and the inclusion criteria. Details regarding the publication and the study, the participants, settings, the interventions, the comparators, the outcome measures, study design, statistical analysis and results, and all other relevant data (funding; conflict of interest etc.) should be carefully and accurately extracted from all included studies. In a review assessing effectiveness, thorough extraction of details of the intervention is essential to allow for reproducibility of an intervention that is found to the effective (Munn et al. 2014). In a JBI systematic review data extraction is performed by two or more reviewers, independently, using the standardized data extraction form developed by JBI. Any disagreements about data extraction are solved by consensus or by the decision of a third reviewer. If non-JBI data extraction forms are used these should be briefly described and the justification for their use should be explicitly indicated. The review protocol should specify if authors of studies will be contacted by reviewers in order to clarify existing data, to request missing data or additional data. The review protocol should specify the pre-planned approach for the situations when there are multiple reports (publications) for the same study, and for missing data and for data conversion /transformation.

For peer review only

3.2.9 Data synthesis

This section should describe how the data will be combined and reported in the systematic review. Essentially, in a systematic review of effectiveness there are two synthesis options: statistical synthesis (meta-analysis) and narrative summary (narrative synthesis). Details of the statistical models and methods and effect estimates that will be calculate and measures of statistical heterogeneity should be included (See Section 3.3). Authors should ensure that the effect estimates that will be calculate correspond to the type of data (dichotomous and/or continuous) they have suggested will be collected in their protocol (see Section 3.2.4.4). The review protocol should also explicitly specify the pre-planned approaches that will be used for the examination of publication bias, including the use of funnel plots and the use of statistical tests for the examination of publication bias (see Section 3.3.11).

The review protocol should explicitly specify that reviewers plan to use the GRADE approach for the reporting of the strength of evidence, including the reporting of the summary of findings table of evidence. The use of GRADE approach is currently endorsed by JBI and JBI reviewers must use it regardless of the synthesis approach employed, meta-analysis or narrative synthesis.

For peer review only

3.3 Meta-analysis

Meta-analysis refers to the statistical synthesis of quantitative results from two or more studies. The review protocol should state that statistical meta-analysis of data will be conducted if appropriate and that if meta-analysis is not possible, narrative synthesis will be conducted as the primary mechanism of data synthesis. Narrative summary should be included to supplement the technical details provided on the process and results even if meta-analysis is performed and to provide synthesis of data not captured in statistical meta-analysis.

Meta-analysis should be reserved for the results of studies that are considered similar enough from a clinical and methodological point of view (homogeneous studies). If studies are heterogeneous from a clinical or methodological point of view, then it is uncertain if it is appropriate to synthesize the respective studies into meta-analysis. Any meta-analysis where studies are heterogeneous from a clinical or methodological point of view will require substantial justification by the authors. Clinical heterogeneity refers to differences between studies with regards the participants, interventions, comparators, settings, and outcomes. Methodological heterogeneity refers to the study design and the methodological quality of the studies (risk of bias). Studies that are similar with regards the participants, interventions, comparators, settings, outcomes, study design, and risk of bias may be combined in meta-analysis. The judgement that studies are homogeneous enough and that it is appropriate to combine the studies in meta-analysis should be based on the understanding of the review question, the characteristics of the studies, and the interpretability of the results. The decision should not be based just on statistical considerations regarding heterogeneity (Sutton et al 2000).

The review protocol should specify the appropriate possible, reasonable details regarding the anticipated (pre-planned) meta-analysis:

- Objectives of the meta-analysis,
- Meta-analysis model (fixed effects model or random effects model) and the justification,
- Effect size to be used (OR, RR, etc.),
- Meta-analysis method (Peto method etc.) and justification,
- Statistical testing procedures used for the exploration of statistical heterogeneity (such as Q Cochran test) and the rules used for the interpretation of the results,
- Statistical indicator used for the quantification of statistical heterogeneity (such as I^2) and the rules used for the interpretation of the results,
- Pre-planned sensitivity analyses and their justification, and
- Pre-planned subgroup analyses and their justification.

For peer review only

3.3.1 Objectives of meta-analysis

The objectives of meta-analysis should be pre-specified in the review protocol. There are different legitimate objectives for a meta-analysis: to improve statistical power to detect a treatment effect, to estimate a summary average effect, to identify subsets of studies (sub-groups) associated with a beneficial effect, and to explore if there are differences in the size or direction of the treatment effect associated with study-specific variables (Normand 1999).

For peer review only

3.3.2 Statistical models for meta-analysis

There are three categories of statistical models for meta-analysis: the fixed effects model, random effects model, and mixed effects models (Hedges 1992). Only the first two models are used in JBI SUMARI for meta-analysis and discussed here. Using the fixed-effect model we assume that the true effect size for all studies is identical and the effect sizes estimated in studies are different only due to errors in estimating the effect size (Borenstein et al 2010). In the random-effects model we assume a distribution of effects, not a common identical effect size, and we assume that the meta-analysis summary effect size is an estimate of the mean of a distribution of true effects, not a common shared effect size identical for all studies (Borenstein et al 2010).

The proposed statistical model for meta-analysis should be explicitly indicated in the review protocol. When considering statistical inference, meta-analysis using the fixed effects model is appropriate if the aim is to draw statistical conclusions only about the studies included in the meta-analysis, and that the random effects model is appropriate whenever statistical generalizations beyond the included studies are considered (Cooper and Hedges 1994). Commonly, review authors want to generalize the conclusions beyond the actual studies included in meta-analysis, therefore we suggest that the default model for meta-analysis in JBI reviews should be the random effects model. However, it has been recommended by statisticians that the fixed effects model is the appropriate model whenever the number of studies is small (less than five studies) (Cooper and Hedges 1994; Murad et al 2015, p.511). Further details about the fixed effects and random effects models for meta-analysis, including a flowchart for the decisions regarding the selection of the meta-analysis model are provided by Tufanaru et al (2015).

For peer review only

3.3.3 Effect sizes

In this section, effect sizes refer to quantitative indicators of the direction and magnitude of the effects of the interventions on outcomes. Common effect sizes reported in meta-analysis include the risk ratio (RR), risk difference (RD), odds ratio (OR), weighted mean difference (WMD), and standardized mean difference (SMD).

For peer review only

3.3.4 Considerations for the meta-analysis of dichotomous data

For meta-analyses, computation of the logarithm (log) of the RR or the log of OR, or the RD from each individual study may be used or the number of events and the total number of participants for each group. RR and RD may be computed for any experimental study (RCT) or quasi-experimental study or cohort studies. Odds ratios may be computed for any study design (experimental, quasi-experimental, cohort, case-control, or analytical cross-sectional studies). Fleiss (1994) discussed the statistical properties of the OR and concluded that the OR is the preferred effect size for the computation phase of the meta-analysis of binary data regardless of the study design of the studies. However, the OR is not easily interpretable. Therefore, reviewers should be careful in providing correct explicit interpretation of the odds ratios computed in meta-analysis. Reviewers should provide the results expressed using both absolute (RD) and relative (RR) effect sizes for meta-analysis of binary data. Reviewers should provide correct explicit interpretation of the computed effect sizes.

For peer review only

3.3.5 Considerations for the meta-analysis of continuous data

For the effect sizes related to differences in continuous data (WMD, SMD), the data regarding the mean response, the standard deviation, and the number of participants in each group are used. The difference in means is the difference between the mean response in the intervention group and the mean response in the control group. This may be the difference in the means between groups at the final measurement of outcomes, or it may be the difference between the means in their changes from baseline. The simple difference in means is also called the mean difference (MD) or the weighted mean difference (WMD). We will use the term the WMD in this chapter. The WMD is used in meta-analysis of continuous data if all studies included in meta-analyses measured the outcome using the same measurement instrument. For meta-analysis computation the difference in means from each individual study are used. The results are expressed in the natural (clinical) units used for the common measurement instrument. If WMD is used, reviewers should provide explanations regarding the interpretation of the results expressed in units used for the common measurement instrument. The minimum score and the maximum score that are possible on the measurement instrument should be specified together with their interpretation. Also, reviewers should specify what change (difference) is considered significant from a practical or clinical point of view. Reviewers should explain the interpretation of a negative or positive difference. The standardized mean difference (SMD) is a difference in means that is standardized by using information on the variability of data (standard deviation). There are three methods (formulas) that are commonly used for the computation of SMD: Cohen's d, Hedges' adjusted g, and Glass's delta. These three formulae use different standard deviations in their computation. Currently, the JBI SUMARI software offers capabilities for the computation of Cohen's d. The SMD is used in meta-analysis of continuous data if the studies measured the same outcome but with different measurement instruments. For meta-analysis computation the SMD from each individual study are used. The results are expressed in units of standard deviation. Reviewers should provide explanations regarding the interpretation of the results. In order to facilitate the interpretation of the results it is recommended that reviewer's convert the results into natural (clinical) units by multiplying the results expressed in units of standard deviation with the standard deviation of the scores from a study on a known measurement instrument. The instrument chosen may be the most commonly used instrument or the instrument which has the best psychometric properties. Reviewers should explain the interpretation of differences and justify what is considered a small or medium or large difference; explanations should be provided for negative or positive differences.

For peer review only

3.3.6 Meta-analysis: Statistical Methods

Different statistical methods are available for meta-analysis: Mantel-Haenszel method, Peto's method, DerSimonian and Laird method, and the inverse variance method. The Mantel-Haenszel method, the Peto's method, and the inverse variance method are methods used with the fixed effects model of meta-analysis (Deeks et al 2008). The DerSimonian and Laird method is used with the random effects model of meta-analysis (Deeks et al 2008).

The inverse variance method may be used with all types of ratios and differences for example the log odds ratio, log relative risk, risk difference, mean difference (weighted mean difference) and standardized mean difference (Petitti 2000; Deeks et al 2008). The Mantel–Haenszel method may be used with ratios, typically with odds ratio, but can be applied to rate ratio and risk ratio (Petitti 2000). The Peto's method is used with odds ratios (Petitti 2000). DerSimonian and Laird method may be used with all types of ratios (odds ratio, risk ratio) and difference (weighted mean difference) and standardized mean difference (Petitti 2000; Deeks et al 2008).

There are different statistical methods (formulae) used to compute a standardized mean difference for each study including the Hedges' method, the Cohen's method, and the Glass method. If a fixed effects model is used for meta-analysis of standardized mean differences then the inverse variance method of meta-analysis may be used. If a random effects model is used for meta-analysis of standardized mean differences then the DerSimonian and Laird method may be used.

When deciding what method for meta-analysis to be used statistical considerations are important. When studies have small sample sizes and the number of events is small in these studies the inverse variance method may not be appropriate; in these circumstances, it may be preferable to use the Mantel-Haenszel method (Deeks et al 2008). Peto's method may produce serious under-estimates when the odds ratio is far from unity (large treatment effects) (Sutton et al 2000). If the number of studies to be combined is small, but the within-study sample sizes per study are large, the inverse-weighted method should be used (Sutton et al 2000, p.69). If there are many studies to combine, but the within-study sample size in each study is small, the Mantel-Haenszel method is preferred (Sutton et al 2000).

For peer review only

3.3.7 Subgroups in meta-analysis

Subgroups refer to diverse grouping of studies based on specific characteristics of the studies such as study design. These characteristics may include the types of participants, types of comparators, and the outcomes. For example, it is possible to group all randomized experimental studies in one subgroup and all observational studies in another group; similarly reviewers may wish to group all studies with young participants in one subgroup and all studies with older participants in another subgroup. For these subgroups, it is possible to perform meta-analysis and to report the summary effects computed within subgroups. Also, it is possible to compare the summary effects computed in diverse subgroups. It is recommended that if subgroup analyses are performed these should be limited in number, should be pre-planned in the review protocol, and explanation and justification should be explicitly provided. These analyses should be carefully interpreted.

For peer review only

3.3.8 Sensitivity analysis in meta-analysis

As there are many decisions involved in meta-analyses it is important to perform a sensitivity analysis in order to explore the impact of different decisions on results. For example, one sensitivity analysis may explore the impact of using different meta-analysis models. Another sensitivity analysis may explore the impact of excluding or including studies in meta-analysis based on sample size, methodological quality, or variance. If results remain consistent across the different analyses, the results can be considered robust as even with different decisions they remain the same/similar. If the results differ across sensitivity analyses, this is an indication that the result may need to be interpreted with caution.

For peer review only

3.3.9 Meta-regression

Meta-regression analysis aims to examine if characteristics of studies are associated with the magnitude and direction of the effect in studies included in meta-analysis. However, given the strict statistical circumstances under which it is appropriate to perform meta-aggregation and also the advanced statistical skills required to use meta-regression software, we cannot recommend the common use of these methods in meta-analysis in JBI reviews of effectiveness.

For peer review only

3.3.10 Heterogeneity

There are different statistical approaches for investigating heterogeneity, included the standard chi-squared test, the I square statistic, and Tau squared.

For peer review only

3.3.10.1 Standard chi-squared test (Cochran test)

The standard chi-squared test (Cochran Q test) for statistical heterogeneity tests the statistical hypothesis that the true treatment effects (the effect size parameters) are the same in all the primary studies included in meta-analysis (Sutton et al 2000). This statistical test uses a test statistic Q that has a chi-squared distribution on k-1 degrees of freedom (k represents the number of studies) under the statistical hypothesis; the corresponding p-value for the test statistic is examined (Sutton et al 2000). The statistical power of the test is in most cases very low due to the small number of studies; heterogeneity may be present even if the Q statistic is not statistically significant at conventional levels of significance such as 0.05. A cut-off significance level of 0.10 rather than the usual 0.05 has been advocated (Sutton et al 2000). If results of the test are statistically significant ($p<0.05$) the statistical hypothesis that the true treatments effects (the effect size parameters) are the same in all the primary studies included in meta-analysis (the hypothesis of homogeneity) is rejected, therefore, it is considered that there is statistical heterogeneity. With a small number of studies (< 20), the Q test should be interpreted very cautiously (Huedo-Medina et al 2006). It is not appropriate to decide the meta-analysis model (fixed or random effects model) based on the results of the Chi squared statistical test (Q test) for heterogeneity.

For peer review only

3.3.10.2 Quantification of the statistical heterogeneity: I squared

The I square statistic (I^2) represents the percentage of the variability in effect estimates that is due to heterogeneity (Deeks et al 2008). I^2 is the proportion of observed dispersion of results from different studies included in a meta-analysis that is real, rather than spurious (Borenstein et al 2009). The I^2 index can be interpreted as the percentage of the total variability in a set of effect sizes due to true heterogeneity (between-studies variability) (Huedo-Medina et al 2006). If I^2 = 0%, this indicates that all variability in effect size estimates is due to sampling error within studies. If I^2= 50%, it indicates that half of the total variability among effect sizes is caused not by sampling error, but by true heterogeneity between studies (Huedo-Medina et al 2006). I^2 is a percentage and its values lie between 0% and 100% (Higgins et al 2003). A value of 0% indicates no observed heterogeneity, and larger values show increasing heterogeneity (Higgins et al 2003). One proposed suggestion was to consider as low, moderate, and high heterogeneity for I^2 values of 25%, 50%, and 75% (Higgins et al 2003). Another guide to interpretation was proposed: 0% to 40% might not be important; 30% to 60% may represent moderate heterogeneity; 50% to 90% may represent substantial heterogeneity; 75% to 100% considerable heterogeneity (Deeks et al 2008). Authors of the guide mention that careful interpretation of the value of I^2 depends on magnitude and direction of effects and strength of evidence for heterogeneity (Deeks et al 2008). With a small number of studies (< 20) and/or average sample size (N <80) the statistical power for I^2 procedures is less than the usually recommended minimum value of 0.8 (Huedo-Medina et al 2006).With a small number of studies (< 20), both the I^2 confidence interval and the Q test should be interpreted very cautiously (Huedo-Medina et al 2006).

For peer review only

3.3.10.3 Tau-squared for random effects model meta-analysis

In random-effects meta-analysis, the extent of variation among the effects observed in different studies (between-study variance) is referred to as tau-squared, ^{2}, or Tau^{2} (Deeks et al 2008). ^{2} is the variance of the effect size parameters across the population of studies and it reflects the variance of the true effect sizes. The square root of this number is referred to as tau (T). T^{2} and Tau reflect the amount of true heterogeneity. T^{2} represents the absolute value of the true variance (heterogeneity). T^{2} is the variance of the true effects while tau (T) is the estimated standard deviation of underlying true effects across studies (Deeks et al 2008). The summary meta-analysis effect and T as standard deviation may be reported in random-effects meta-analysis to describe the distribution of true effects (Borenstein et al 2009).

For peer review only

3.3.11 Publication bias

Publication bias occurs when published studies differ systematically from all conducted studies on a topic. Publication bias arises when studies with statistically significant results or positive results in a specific direction are more likely to be published compared to studies without statistically significant results or negative results. Reviewers should make all reasonable efforts to include in their systematic review all or most of all relevant studies, regardless of the nature of reports (published or unpublished. Publication bias can have a detrimental effect on the validity of systematic reviews (Deeks et al 2008). Funnel plots are a method of investigating the located studies in a meta-analysis for publication bias, they are scatter plots in which an effect estimate of each study is plotted against a measure of size or precision (i.e. standard error) (Deeks et al 2008). The largest studies should be closest to the ‘true’ value, with the smaller studies spread on either side; creating the shape of a funnel if publication bias is not present. If publication bias has had an effect on the studies available (and there are no other confounding factors) then the ‘funnel’ should be incomplete with an area missing (Deeks et al 2008). Generally the best way to minimise the impact of publication bias on a systematic review is the inclusion of trial registries and unpublished studies or grey literature (Lau et al 2006; Sterne et al 2011). Funnel plots suffer from numerous issues including low power, numerous alternative explanations for asymmetrical distribution of studies, and inaccurate researcher interpretations of plots (Lau et al 2006; Sterne et al 2011). However, they remain a useful and popular way of investigating publication bias (Deeks et al 2008). Potential reasons for funnel plot asymmetry other than publication bias include: poor methodological quality leading to exaggerated effects in smaller studies (which can be the result of poor methodological design, inadequate analysis, or fraud), true heterogeneity, artefactual causes (in some situations sampling variation can lead to an association between the two factors (effect estimate and measure of size or precision)) and chance (Sterne et al 2011). The visual inspection of funnel plots introduces great uncertainty and subjectivity. In a survey utilizing simulated plots, researchers had only 53% accuracy at identifying publication bias (Lau et al 2006). A very liberal minimum number of studies for the performance of a funnel plot to be justified is ten (Lau et al 2006).

Statistical tests for funnel plot asymmetry (also known as tests for publication bias) investigate the association between effect size estimate and measure of study size or precision. The most popular statistical tests for funnel plot asymmetry are Egger test, Begg test, and the Harbord test. These tests were developed based on the following assumptions: large studies are more likely to be published regardless of statistical significance; small studies are at the greatest risk for being lost; in small studies only the large effects are likely to be statistically significant therefore published small studies often show larger effect sizes compared to larger studies; small and unfavorable effects are more likely to be missing; small studies with large effect sizes are likely to be published (Jin et al 2015). Null statistical hypotheses for these tests reflect the hypothesis of symmetry of the plot, that is, the hypothesis of no publication bias. A finding of not statistically significant P-value for the asymmetry test does not exclude bias. These tests are known to have low power.

A statistical test for funnel plot asymmetry investigates whether the association between effect estimate and measure of study size or precision is larger than what can be expected to have occurred by chance (Sterne et al 2011). These tests are known to have low power and consequently a finding of no evidence of asymmetry does not serve to exclude bias (Sterne et al 2011).

The Begg’s Test was proposed by Begg and Mazumdar in 1994. It is used for dichotomous outcomes with intervention effects measured as odds ratios. It is an adjusted rank correlation test (Jin et al 2015). It explores the correlation between the effect estimates and their sampling variances (Jin et al 2015). It is a very popular test, however, it has low power; some statisticians do not recommend its use. It is “fairly powerful” for meta-analysis of 75 studies; it has “moderate power” for meta-analysis of 25 studies (Begg and Mazumdar 1994). It is considered that the test has “appropriate” type I error rate (Jin et al 2015).

The Egger’s test was proposed by Egger et al in 1997. It is used for continuous outcomes with intervention effects measured as mean differences. It is a “regression test”, that is, it uses a linear regression approach (Jin et al 2015). The standard normal deviate (estimated effect size/estimated standard error) is regressed against the estimate’s precision. It is a very popular test. It is considered that the test has “inappropriate” type I error rate when heterogeneity is present and the number of included studies is large (Jin et al 2015). The Egger test for funnel asymmetry is the most cited statistical test for publication bias.

The Harbord Test was proposed by Harbord et al in 2006. It is used for dichotomous outcomes with intervention effects measured as odds ratios. The test uses “a weighted regression model” (Jin et al 2015). It is considered that the test has “inappropriate” type I error rate when heterogeneity is present. It was contended that the Harbord Test has better error rate compared to Egger’s test in balanced trials with little or no heterogeneity (Jin et al 2015).

For peer review only - http://bmjopen.bmj.com/site/about/guidelines.xhtml

3.4 Systematic review of effectiveness

A systematic review report is important because it provides all the details regarding the conduct of the systematic review and the best available evidence to inform the question posed by the review. Essentially, the content of the sections of the review protocol and the review report are conceptually the same, particularly the background and the methods section. The review protocol specified the proposed plan for the review; the review report reports the conduct of the review, what was actually performed and the results of the review undertaking. All deviations from what was pre-planned in the review protocol should be explicitly reported and justified in the review report.

For peer review only

3.4.1 Title

A clear, descriptive title is important to assist readers and users to readily identify the scope and relevance of the review. The review report title should accurately describe and reflect the content of the review, and should not be phrased as a question. The review title should explicitly identify the publication as a report for a finalized systematic review. It is important to indicate in the review title the focus of the review on effectiveness; we recommend the following convention: *'The effectiveness of [intervention] compared to [comparator] on [outcome]: a systematic review'*. The title of the review should be as descriptive as possible and reflect all relevant information. Ideally, the review title should include in a concise way the relevant information with regards to the types of participants, types of interventions and comparators and the types of outcomes considered in the review.

For peer review only

3.4.2 Abstract

This section forms a structured abstract of the main features of the systematic review. It must be no longer than 500 words and should contain no abbreviations or references. The abstract must accurately reflect and summarize the systematic review with the main focus on the results of the review.

The abstract should report the essential elements of the review using the following sub-headings in this order:

- **Objective**: State an overarching review objective structured using the key components of the inclusion criteria (approximately one to two sentences).
- **Background**: Briefly describe what is already known on the topic and what this review will add to the evidence-base (approximately two to three sentences).
- **Inclusion criteria:** Summarize the inclusion criteria as it relates to the type of review being conducted. Present the information in one or two sentences – **NOT** under individual subheadings.
- **Methods:** List the key information sources searched (those that provided the majority of included studies), any limits placed on the scope of the search (e.g. language), and the date range, or the date of the last search. If the recommended JBI approach to critical appraisal, study selection, data extraction and data synthesis was used, simply state it as such (without naming the actual tool). Otherwise, briefly describe any notable deviations to the methodological approach taken (e.g. criteria used to exclude studies on the basis of methodological quality etc.).
- **Results**: The bulk of the abstract should be reserved to convey the main results of the review.
  - As a general rule, report the number and type of included studies and participants, as well as any pertinent study characteristics. Summarize the overall quality of the included studies and notable aspects of risk of bias.
  - Report the results for all main outcomes (not only those that were statistically significant or clinically important). If meta-analyses were conducted report the summary measures (estimated effect) and confidence intervals and ensure statistics are presented in a standard way. If a meta-analysis was proposed but not conducted, report the reason (e.g. clinical or methodological heterogeneity). Where possible, indicate the number of studies and participants for each main outcome. Describe the direction of the effect (e.g. lower, fewer, greater, more, etc.) in a way that is understandable to patients and health care professionals (i.e. which group was favored and the size of the effect) and indicate the measurement scale used, where applicable.
- **Conclusions**: Provide a conclusion based on a general interpretation of the results considering, for example, the methodological quality of the included studies and any limitations of the review. Briefly convey key implications for practice and/or research.

For peer review only

3.4.3 GRADE 'Summary of Findings' table

The use of the GRADE approach is currently endorsed by JBI and JBI reviewers must use it regardless of the synthesis approach employed, meta-analysis or narrative synthesis. The GRADE ‘Summary of Findings’ table should be presented immediately below the abstract. The GRADE ‘Summary of Findings’ table can be developed following the guidance in the GRADE handbook (Schunnemann et al. 2013). Links to resources and support for using GRADE are available via the JBI Adelaide GRADE Centre.

For peer review only

3.4.4 Introduction

The introduction of the review report should provide explicit and comprehensive information regarding the justification (rationale) for the conduct of the review in the context of what was already known. Ideally, this section of the review report should be a revised, expanded, version of the introductory section from the review protocol. See Section 3.2.3 from the review protocol for further information regarding the content of the introduction.

The introduction should conclude with an overarching review objective that captures and aligns with the core elements/mnemonic of the inclusion criteria (e.g. PICO). The stated objective should clearly indicate what the review project is trying to achieve. Vancouver style of referencing should be used throughout the protocol with superscript numbers without brackets, used for in-text citations.

For peer review only

3.4.5 Review question(s)

The review question(s) should be explicitly stated in unambiguous terms. See the Section 3.2.2 of this Chapter for further information regarding the objectives and questions of a review of effectiveness.

For peer review only

3.4.6 Inclusion criteria

This section should describe the inclusion criteria used for the review. Information should be provided regarding the types of participants, types of interventions, comparators, types of outcomes, and types of studies actually considered and included in the review. See Section 3.2.4 for further details regarding specification of inclusion criteria in the systematic review report.

For peer review only

3.4.7 Methods

This section of the review report is reserved for the methods used to conduct the review and should be presented under the relevant subheadings(See Sections 3.4.6.1 to Section 3.4.6.5), including any deviations from the method outlined in the *a priori* protocol. In empty reviews for example, this section should not refer to methods that were not performed.

Directly below the Methods heading provide the following information:

- State and appropriately cite the JBI methodology that was employed in the conduct of the review and synthesis.
- Refer to and cite the *a priori* protocol that was published, or accepted for publication (e.g. 'in press'), in the *JBI Evidence Synthesis*.
- If the protocol has been registered with PROSPERO, provide registration information including registration number (e.g. PROSPERO CRD42015425226).

For peer review only

3.4.7.1 Search strategy

The search strategy section of a review report should provide explicit and clear information regarding all information sources that were actually used in the review, and the actual strategies used for searching. The review report should provide details regarding all information sources that were used in the review: electronic bibliographic databases; trial registers; relevant journals; websites of relevant organizations; etc. The review report, ideally, should specify all the details (a line-by-line description) of the actual search strategy used for each electronic bibliographic database used for the review and should be provided in an appendix. The review report should specify the timeframe for search, the date of last search for each database, and any language and date restrictions, with appropriate justifications.

For peer review only

3.4.7.2 Study screening and selection

The review report should describe the actual process of study screening and for all stages of selection (based on title and abstract examination; based on full text examination) and the actual procedures used for solving disagreements between reviewers.

For peer review only

3.4.7.3 Critical appraisal

The review report should specify the critical appraisal process and instruments that were actually used in the review process and the procedures for solving disagreements between reviewers. The review report should describe how the results of critical appraisal were used for the exclusion of studies from the review, if this is the case. The details of the decisions processes and criteria used for exclusion of studies based on results of critical appraisal should be explicitly provided. All details about the scoring systems and the cut-off scores for inclusion of studies in the review should be described and justified.

For peer review only

3.4.7.4 Data extraction

The review report should specify the data extraction process and instruments that were used in the review process and the procedures for solving disagreements between reviewers.

For peer review only

3.4.7.5 Data synthesis

The review report should explicitly specify how the data were combined and reported. Essentially, the review report should provide the details about all preformed analyses and their justifications. The synthesis approaches by which studies were combined should be described in as much detail as is reasonably possible and to enable them to be reproduced.

If meta-analysis was performed, the review report should specify the details regarding the performed meta-analyses. The report should specify:

- the objectives of the meta-analysis
- the effect size used (OR, RR, etc.)
- the meta-analysis model (fixed effects model or random effects model) and the justification
- the meta-analysis method (Peto method etc.) and the justification
- the statistical testing procedures used for the exploration of statistical heterogeneity (such as Q Cochran test) and the rules used for the interpretation of the results
- the statistical indicator used for the quantification of statistical heterogeneity (such as I^2) and the rules used for the interpretation of the results
- the performed sensitivity analyses
- the performed subgroup analyses

For peer review only

3.4.8 Results

This section of the review report has distinct sub-sections describing the process of study inclusion, the methodological quality of the eligible studies, detailed characteristics and description of the included studies and, importantly, the findings of the review and results of the synthesis processes.

For peer review only

3.4.8.1 Study inclusion

This section should provide a narrative summary of the search results and selection process and results. The number of papers identified by the search strategy and the number of papers that were included and excluded should be stated.

A complete and accurate report should be provided regarding:

- the number of studies identified by the search in diverse sources;
- the number of studies excluded after the examination of title and abstract against inclusion criteria;
- the number of full text articles retrieved for examination;
- the number of studies excluded after full text examination against inclusion criteria;
- the number of critically appraised studies;
- the number of studies excluded after critical appraisal;
- the final total number of included studies.

A flowchart using the PRISMA template for the reporting of the selection process should be included.

Ideally, a list of all excluded studies, excluded after full text examination and after critical appraisal, with the explicit reasons for exclusion, should be provided in appendices to the review. As a minimum, at least the list of studies excluded after critical appraisal and the reasons for exclusion should be reported. If no studies were excluded after critical appraisal then the list of all studies excluded after full text examination including the explicit reasons for exclusion, should be provided in appendices to the review.

For peer review only

3.4.8.2 Methodological quality

The review report should report in a comprehensive manner, in narrative form and in tables, the results of risk of bias (methodological quality) assessments for each aspect of methodological quality (randomization; blinding; measurement; statistical analysis etc.) for each individual study and the overall risk of bias of the entire set of included studies. This section must provide an overarching statement of the quality of the included studies as a whole (i.e. low, moderate, high, etc.) and a narrative summary of the methodological quality of the included studies against each of the critical appraisal criteria, with a clear indication of the risks of bias present across the included studies (e.g. performance bias, detection bias etc.). Reporting can be supported (optional) by a table showing the results of the critical appraisal (see Table 3.1 for example). Where only few studies are identified, or there are specific items of interest from included studies, these should be addressed in the narrative also, particularly where studies were deficient, or particularly good. Use of 'Unclear' and 'Not Applicable' should also be explained in the text.

Table 3.1. Critical appraisal results for included studies using the JBI-Critical Appraisal Checklist for randomised controlled trials

Study	Q1	Q2	Q3	Q4	Q5	Q6	Q7	Q8	Q9	Q10
Author(s) ref	Y	Y	Y	N	Y	U	Y	N	Y	U
Author(s) ref	Y	N	Y	Y	Y	U	Y	N/A	Y	Y

Y - Yes, N - No, U - Unclear, N/A - not applicable

If appraisal tools are not appended to the review report (citation only), the appraisal questions should be added as a footnote/caption to the table (Table 3.1) so readers can clearly interpret the information presented.

For peer review only

3.4.8.3 Characteristics of included studies

This section should include a narrative summary of the details about the design and details of the included studies. Relevant characteristics of the included studies for which data were extracted and are needed to understand and interpret the results of the study should be synthesized in narrative. This includes the descriptive and demographic features (e.g. the country and setting of the study) of the included studies, as well as the main clinical characteristics, as they relate to the review objective and the inclusion criteria (e.g. PICOs). For example, in a review of effects, synthesize characteristics of the population, intervention, comparator, outcomes, and study design. Information on interventions should include treatment modalities and the amount, duration, frequency and intensity of the intervention any details related to the follow-up of the participants. Population characteristics should include the number of participants (i.e. study size) and demographic information such as age, gender and any information relevant to the specific review question (e.g. past medical history, diagnosis, co-morbidities).

Reviewers should provide an appendix of the review report summarized details of the included studies. The examination of the table of included studies should suffice to convince the readers that there is good match between the included studies and the inclusion criteria.

For peer review only

3.4.8.4 Results and meta-analysis

This section should be organized in a meaningful way based on the review objectives and questions and types of interventions, comparators, outcomes and types of studies. This section should provide comprehensive information regarding the results of all performed meta-analyses and additional analyses such as sensitivity analysis and sub-group analysis. Point estimates and interval estimates (confidence intervals) should be reported. Before presenting any meta-analysis results, the conduct of meta-analyses should be justified; reviewers should explicitly provide commentaries regarding the clinical, methodological, and statistical heterogeneity of the studies included in meta-analyses and the appropriateness of conducting meta-analyses. Summary results from meta-analyses should be reported as summary point estimates and interval estimates. The meta-analysis forest plots for all performed meta-analyses should be presented in this section. A narrative summary should complement the forest plots and provide additional commentaries and explanations for all performed meta-analyses (Munn et al 2014).

Reviewers should report the funnel plot for publication bias if such assessment was appropriate and performed. Reviewers should include the results of assessment of risk of publication bias, including the results of statistical tests for publication bias, if such tests were used.

Even if meta-analysis is performed, a narrative summary should be included to supplement the technical details provided on the process and results of meta-analysis and to provide synthesis of data not captured in statistical meta-analysis.

If meta-analysis is not performed, a narrative summary should be included. The narrative summary should provide an overall summary of the findings of the included studies and their biases, strengths and limitations. The essence of narrative summary is that the results are summarized in words and in tables without any statistical meta-analysis. Textual commentaries and tables are used in order to summarize the results from the included studies and to provide context information for these results, thus facilitating understanding of the summarized results.

For peer review only

3.4.9 Discussion

The aim of this section is to briefly summarize the main findings and then focus on the discussion of these results. Results should be discussed, compared and contrasted with what was already known from other sources, other than the review, usually at a minimum the literature mentioned in the background section, however, additional external literature may be discussed here in order to facilitate the understanding and positioning of the review results in a broader research and practice context. The applicability and generalizability of the review results should be discussed. The significance of the results should be discussed for individual studies and for meta-analyses. It is not enough to discuss the statistical significance of the results; the practical/clinical significance of the results should be discussed regardless of the statistical significance of the results. The minimum and maximum values for the scales of measurement or measurement instruments should be discussed and the values that are considered to represent the minimum important change from a clinical/practical point of view.

This section should provide a presentation of the limitations of included studies and the limitations of the review process. Limitations of each included study (limitations in the design and conduct of the research, including risk of bias) should be discussed. Also, the limitations of entire set of included studies should be discussed in terms of common limitations (including risk of bias). All limitations, issues and problems noted in the review process related to the search, selection of study, critical appraisal, data extraction, and data synthesis, should be discussed. The impact of the limitations of the studies and of the review process on the applicability and generalizability of the results should be considered.

For peer review only

3.4.10 Conclusions and recommendations

This section should include the overall conclusions of the review. The conclusions should provide direct answers to the review objectives/questions. These conclusions should be based only on the results of the review and directly inferred from the review results.

Recommendations for practice

This sub-section of Conclusions section should include the recommendations for practice inferred from the results of the review and inferred also based on the discussion of the generalizability of the results and the potential factors that may affect the applicability of results. Recommendations should be assigned a JBI Grade of Recommendation. Refer for the editorial by Munn 2015 for further discussion regarding the appropriateness of making recommendations in systematic reviews.

Recommendations for research

This sub-section of Conclusions should include the recommendations for future research inferred from the results of the review, specifically, inferred from the limitations, issues and problems noted in the review process related to the search, selection of study, critical appraisal, data extraction, and data synthesis.

For peer review only

3.4.11 Conflicts and acknowledgements

Details of requirements in these sections are described in Section 1.6. of this Manual.

Conflicts of interest

A statement which either declares the absence of any conflicts of interest or which describes a specified or potential conflict of interest should be made by the reviewers in this section.

Funding

Authors should provide details regarding any sources of funding for the review project. The role of all funders in the review process, if any, should be explicitly described. If the review is funded, then any potential conflicts of interest or intellectual bias of the funders should be specified in the review.

Acknowledgements

Any acknowledgements should be made in this section e.g. sources of external funding or the contribution of colleagues or institutions. It should also be noted if the systematic review is to count towards a degree award.

For peer review only

3.4.12 Review Appendices

There are several required appendices for a JBI review:

Appendix 1: Search strategy

- A detailed and complete search strategy for all of the major databases and other sites and sources searched must be appended. Major databases that were searched must be identified, including the search platform used where necessary. All search filters with logic employed should be displayed, including the number of records returned.

Appendix 2: Data extraction instrument

- The data extraction instrument used must be appended i.e JBI SUMARI Data Extraction Form.

Appendix 3: List of excluded studies

- Studies excluded following examination of the full-text should be listed along with their reason for exclusion at that stage (i.e. a mismatch with the inclusion criteria). This may be as a separate appendix or itemized in some fashion within the one appendix with those studies excluded at the critical appraisal stage. Reasons for exclusion following appraisal should be provided for each study (these reasons should relate to the methodological quality of the study, not study eligibility).

Appendix 4: Table of included study characteristics

- A table of included studies is required to provide quick reference to important details extracted from of the studies included in the review.

For peer review only

3.5 Chapter References

Aromataris E. Ins and outcomes. JBI Database System Rev Implement Rep. 2015; 13(4): 1-2.

Aromataris E, Riitano D. Constructing a search strategy and searching for evidence. A guide to the literature search for a systematic review. Am J Nurs. 2014; 114(5): 49-56.

Begg CB, Mazumdar M. Operating Characteristics of a Rank Correlation Test for Publication Bias. Biometrics. 1994; 50(4): 1088-1101.

Borenstein M, Hedges LV, Higgins JPT, Rothstein HR. A basic introduction to fixed-effect and random-effects models for meta-analysis. Res Synth Methods 2010; 1: 97–111.

Borenstein M, Hedges LV, Higgins JPT, Rothstein HR. Introduction to Meta-Analysis. Wiley, 2009.

Brignardello-Petersen R, Ioannidis JPA, Tomlinson G, Guyatt G. Surprising results of randomized trials. In Guyatt G, Rennie D, Meade MO, Cook DJ (editors). Users' Guide to the medical literature. A manual for evidence-based clinical practice. 3rd edition. New York: McGraw-Hill, 2015.

Cooper H, Hedges LV. Potentials and limitations of research synthesis. In: Cooper H, Hedges LV, editors. The handbook of research synthesis. New York: Russell Sage Foundation, 1994.

Deeks JJ, Higgins JPT, Altman DG (editors). Chapter 9: Analysing data and undertaking meta-analyses. In: Higgins JPT, Green S (editors). Cochrane Handbook for Systematic Reviews of Interventions. Chichester (UK): John Wiley & Sons, 2008.

Egger M, Davey Smith G, Schneider M, Minder C. Bias in meta-analysis detected by a simple, graphical test. BMJ.1997; 315:629–34.

Fleiss JL. Measures of effect size for categorical data. In: Harris Cooper Hedges LV, editors. The handbook of research synthesis. New York: Russell Sage Foundation, 1994.

Harbord RM, Egger M, Sterne JAC. A modified test for small-study effects in meta-analyses of controlled trials with binary endpoints. Statist. Med. 2006; 25:3443–3457.

Hedges LV. Meta-analysis. J Educ Behav Stat 1992; 17: 279– 96.

Huedo-Medina TB, Sánchez-Meca J, Marín-Martínez F, Botella J. Assessing heterogeneity in meta-analysis: Q statistic or I2 index? Psychol Methods. 2006; 11(2): 193-206.

Jin ZC, Zhou XH, He J. Statistical methods for dealing with publication bias in meta-analysis. Stat Med. 2015; 34(2): 343-60.

Lau J, Ioannidis JP, Terrin N, Schmid CH, Olkin I. The case of the misleading funnel plot. Bmj. 2006; 333 (7568): 597-600.

Munn Z, Tufanaru C, Aromataris E. JBI's systematic reviews: data extraction and synthesis. Am J Nurs. 2014; 114(7):49-54.

Munn Z. Implications for Practice: should recommendations be recommended in systematic reviews? JBI database of systematic reviews and implementation reports. 2015 Jan 1;13(7):1-3.

Murad MH, Montori VM, Ioannidis JPA, et al. Fixed-effects and random-effects models. In: Guyatt G, Rennie D, Meade MO, Cook DJ, editors. Users' guide to the medical literature.

A manual for evidence-based clinical practice. 3rd ed. New York: McGraw-Hill, 2015.

Normand SL. Meta-analysis: formulating, evaluating, combining, and reporting. Stat Med 1999; 18: 321–59.

Petitti DB. Meta-analysis, decision analysis, and cost-effectiveness analysis: methods for quantitative synthesis in medicine. 2nd ed. New York: Oxford University Press, 2000.

Porritt K, Gomersall J, Lockwood C. JBI's Systematic Reviews: Study selection and critical appraisal. Am J Nurs. 2014; 114(6):47-52.

Schünemann H, Broek J, Guyatt G, Oxman A (editors). GRADE Handbook. Handbook for grading the quality of evidence and the strength of recommendations using the GRADE approach. Updated October 2013.

Shadish WR, Cook TD, Campbell DT. Experimental and Quasi-experimental designs for generalized causal inference. Boston: Houghton Mifflin Company, 2002.

Stern C, Jordan Z, McArthur A. Developing the review question and inclusion criteria. Am J Nurs. 2014; 114(4): 53-6.

Sterne JA, Sutton AJ, Ioannidis JP et al. Recommendations for examining and interpreting funnel plot asymmetry in meta-analyses of randomised controlled trials. Bmj. 2011; 343: d4002.

For peer review only

Sutton AJ, Abrams KR, Jones DR, Sheldon TA, Song F. Methods for meta-analysis in medical research. New York: Wiley, 2000.

Trohler U. The 18th century British Origins of a critical approach. Edinburgh: Royal College of Physicians, 2000.

Tufanaru C. Surrogate outcomes. JBI Database System Rev Implement Rep. 2016; 14(11): 1-2.

Tufanaru C, Munn Z, Stephenson M, Aromataris E. Fixed or random effects meta-analysis? Common methodological issues in systematic reviews of effectiveness. Int J Evid Based Healthc. 2015; 13(3):196-207.

For peer review only

Appendix 3.1: JBI Critical appraisal checklist for randomized controlled trials

JBI Critical Appraisal Checklist for Randomized Controlled Trials

Reviewer____Date____________________________________

Author ____Year________________Record Number ________

	Yes	No	Unclear	NA
1. Was true randomization used for assignment of participants to treatment groups?				
2. Was allocation to treatment groups concealed?				
3. Were treatment groups similar at the baseline?				
4. Were participants blind to treatment assignment?				
5. Were those delivering treatment blind to treatment assignment?				
6. Were outcomes assessors blind to treatment assignment?				
7. Were treatment groups treated identically other than the intervention of interest?				
8. Was follow up complete and if not, were differences between groups in terms of their follow up adequately described and analyzed?				
9. Were participants analyzed in the groups to which they were randomized?				
10. Were outcomes measured in the same way for treatment groups?				
11. Were outcomes measured in a reliable way?				
12. Was appropriate statistical analysis used?				
13. Was the trial design appropriate, and any deviations from the standard RCT design (individual randomization, parallel groups) accounted for in the conduct and analysis of the trial?				

Overall appraisal: Include Exclude Seek further info

Comments (Including reason for exclusion)

____

____

____

For peer review only

Appendix 3.2: Discussion of JBI appraisal criteria for randomized controlled trials

Critical Appraisal Tool for RCTs (individual participants in parallel groups)

Answers: Yes, No, Unclear or Not Applicable

1. **1. Was true randomization used for assignment of participants to treatment groups?**

The differences between participants included in compared groups constitutes a threat to the internal validity of a study exploring causal relationships. If participants are not allocated to treatment and control groups by random assignment there is a risk that the allocation is influenced by the known characteristics of the participants and these differences between the groups may distort the comparability of the groups. A true random assignment of participants to the groups means that a procedure is used that allocates the participants to groups purely based on chance, not influenced by the known characteristics of the participants. Check the details about the randomization procedure used for allocation of the participants to study groups. Was a true chance (random) procedure used? For example, was a list of random numbers used? Was a computer-generated list of random numbers used?

1. **2. Was allocation to groups concealed?**

If those allocating participants to the compared groups are aware of which group is next in the allocation process, that is, treatment or control, there is a risk that they may deliberately and purposefully intervene in the allocation of patients by preferentially allocating patients to the treatment group or to the control group and therefore this may distort the implementation of allocation process indicated by the randomization and therefore the results of the study may be distorted. Concealment of allocation (allocation concealment) refers to procedures that prevent those allocating patients from knowing before allocation which treatment or control is next in the allocation process. Check the details about the procedure used for allocation concealment. Was an appropriate allocation concealment procedure used? For example, was central randomization used? Were sequentially numbered, opaque and sealed envelopes used? Were coded drug packs used?

1. **3. Were treatment groups similar at the baseline?**

The differences between participants included in compared groups constitute a threat to the internal validity of a study exploring causal relationships. If there are differences between participants included in compared groups there is a risk of selection bias. If there are differences between participants included in the compared groups maybe the 'effect' cannot be attributed to the potential 'cause' (the examined intervention or treatment), as maybe it is plausible that the 'effect' may be explained by the differences between participants, that is, by selection bias. Check the characteristics reported for participants. Are the participants from the compared groups similar with regards to the characteristics that may explain the effect even in the absence of the 'cause', for example, age, severity of the disease, stage of the disease, co-existing conditions and so on? Check the proportions of participants with specific relevant characteristics in the compared groups. Check the means of relevant measurements in the compared groups (pain scores; anxiety scores; etc.). *[Note: Do NOT only consider the P-value for the statistical testing of the differences between groups with regards to the baseline characteristics.]*

1. **4. Were participants blind to treatment assignment?**

If participants are aware of their allocation to the treatment group or to the control group there is the risk that they may behave differently and respond or react differently to the intervention of interest or to the control intervention respectively compared to the situations when they are not aware of treatment allocation and therefore the results of the study may be distorted. Blinding of participants is used in order to minimize this risk. Blinding of the participants refers to procedures that prevent participants from knowing which group they are allocated. If blinding of participants is used, participants are not aware if they are in the group receiving the treatment of interest or if they are in any other group receiving the control interventions. Check the details reported in the article about the blinding of participants with regards to treatment assignment. Was an appropriate blinding procedure used? For example, were identical capsules or syringes used? Were identical devices used? Be aware of different terms used, blinding is sometimes also called masking.

1. **5. Were those delivering treatment blind to treatment assignment?**

If those delivering treatment are aware of participants' allocation to the treatment group or to the control group there is the risk that they may behave differently with the participants from the treatment group and the participants from the control group, or that they may treat them differently, compared to the situations when they are not aware of treatment allocation and this may influence the implementation of the compared treatments and the results of the study may be distorted. Blinding of those delivering treatment is used in order to minimize this risk. Blinding of those delivering treatment refers to procedures that prevent those delivering treatment from knowing which group they are treating, that is those delivering treatment are not aware if they are treating the group receiving the treatment of interest or if they are treating any other group receiving the control interventions. Check the details reported in the article about the blinding of those delivering treatment with regards to treatment assignment. Is there any information in the article about those delivering the treatment? Were those delivering the treatment unaware of the assignments of participants to the compared groups?

For peer review only

1. **6. Were outcomes assessors blind to treatment assignment?**

If those assessing the outcomes are aware of participants' allocation to the treatment group or to the control group there is the risk that they may behave differently with the participants from the treatment group and the participants from the control group compared to the situations when they are not aware of treatment allocation and therefore there is the risk that the measurement of the outcomes may be distorted and the results of the study may be distorted. Blinding of outcomes assessors is used in order to minimize this risk. Check the details reported in the article about the blinding of outcomes assessors with regards to treatment assignment. Is there any information in the article about outcomes assessors? Were those assessing the treatment's effects on outcomes unaware of the assignments of participants to the compared groups?

1. **7. Were treatment groups treated identically other than the intervention of interest?**

In order to attribute the 'effect' to the 'cause' (the treatment or intervention of interest), assuming that there is no selection bias, there should be no other difference between the groups in terms of treatment or care received, other than the manipulated 'cause' (the treatment or intervention controlled by the researchers). If there are other exposures or treatments occurring at the same time with the 'cause' (the treatment or intervention of interest), other than the 'cause', then potentially the 'effect' cannot be attributed to the examined 'cause' (the investigated treatment), as it is plausible that the 'effect' may be explained by other exposures or treatments occurring at the same time with the 'cause' (the treatment of interest). Check the reported exposures or interventions received by the compared groups. Are there other exposures or treatments occurring at the same time with the 'cause'? Is it plausible that the 'effect' may be explained by other exposures or treatments occurring at the same time with the 'cause'? Is it clear that there is no other difference between the groups in terms of treatment or care received, other than the treatment or intervention of interest?

1.8. Was follow up complete and if not, were differences between groups in terms of their follow up adequately described and analyzed?

For this question, follow up refers to the time period from the moment of random allocation (random assignment or randomization) to compared groups to the end time of the trial. This critical appraisal question asks if there is complete knowledge (measurements, observations etc.) for the entire duration of the trial as previously defined (that is, from the moment of random allocation to the end time of the trial), for all randomly allocated participants. If there is incomplete follow up, that is incomplete knowledge about all randomly allocated participants, this is known in the methodological literature as the post-assignment attrition. As RCTs are not perfect, there is almost always post-assignment attrition, and the focus of this question is on the appropriate exploration of post-assignment attrition (description of loss to follow up, description of the reasons for loss to follow up, the estimation of the impact of loss to follow up on the effects etc.). If there are differences with regards to the loss to follow up between the compared groups in an RCT, these differences represent a threat to the internal validity of a randomized experimental study exploring causal effects, as these differences may provide a plausible alternative explanation for the observed 'effect' even in the absence of the 'cause' (the treatment or intervention of interest). When appraising an RCT, check if there were differences with regards to the loss to follow up between the compared groups. If follow up was incomplete (that is, there is incomplete information on all participants), examine the reported details about the strategies used in order to address incomplete follow up, such as descriptions of loss to follow up (absolute numbers; proportions; reasons for loss to follow up) and impact analyses (the analyses of the impact of loss to follow up on results). Was there a description of the incomplete follow up (number of participants and the specific reasons for loss to follow up)? It is important to note that with regards to loss to follow up, it is not enough to know the number of participants and the proportions of participants with incomplete data; the reasons for loss to follow up are essential in the analysis of risk of bias; even if the numbers and proportions of participants with incomplete data are similar or identical in compared groups, if the patterns of reasons for loss to follow up are different (for example, side effects caused by the intervention of interest, lost contact etc.), these may impose a risk of bias if not appropriately explored and considered in the analysis. If there are differences between groups with regards to the loss to follow up (numbers/proportions and reasons), was there an analysis of patterns of loss to follow up? If there are differences between the groups with regards to the loss to follow up, was there an analysis of the impact of the loss to follow up on the results? *[Note: Question 8 is NOT about intention-to-treat (ITT) analysis; question 9 is about ITT analysis.]*

1.9. Were participants analyzed in the groups to which they were randomized?

This question is about the intention-to-treat (ITT) analysis. There are different statistical analysis strategies available for the analysis of data from randomized controlled trials, such as intention-to-treat analysis (known also as intent to treat; abbreviated, ITT), per-protocol analysis, and as-treated analysis. In the ITT analysis the participants are analyzed in the groups to which they were randomized, regardless of whether they actually participated or not in those groups for the entire duration of the trial, received the experimental intervention or control intervention as planned or whether they were compliant or not with the planned experimental intervention or control intervention. The ITT analysis compares the outcomes for participants from the initial groups created by the initial random allocation of participants to those groups. Check if ITT was reported; check the details of the ITT. Were participants analyzed in the groups to which they were initially randomized, regardless of whether they actually participated in those groups, and regardless of whether they actually received the planned interventions? *[Note: The ITT analysis is a type of statistical analysis recommended in the Consolidated Standards of Reporting Trials (CONSORT) statement on best practices in trials reporting, and it is considered a marker of good methodological quality of the analysis of results of a randomized trial. The ITT is estimating the effect of offering the intervention, that is, the effect of instructing the participants to use or take the intervention; the ITT it is not estimating the effect of actually receiving the intervention of interest.]*

For peer review only

10. Were outcomes measured in the same way for treatment groups?

If the outcome (the 'effect') is not measured in the same way in the compared groups there is a threat to the internal validity of a study exploring a causal relationship as the differences in outcome measurements may be confused with an effect of the treatment (the 'cause'). Check if the outcomes were measured in the same way. Same instrument or scale used? Same measurement timing? Same measurement procedures and instructions?

11. Were outcomes measured in a reliable way?

Unreliability of outcome measurements is one threat that weakens the validity of inferences about the statistical relationship between the 'cause' and the 'effect' estimated in a study exploring causal effects. Unreliability of outcome measurements is one of the different plausible explanations for errors of statistical inference with regards to the existence and the magnitude of the effect determined by the treatment ('cause'). Check the details about the reliability of measurement such as the number of raters, training of raters, the intra-rater reliability, and the inter-raters reliability within the study (not as reported in external sources). This question is about the reliability of the measurement performed in the study, it is not about the validity of the measurement instruments/scales used in the study. *[Note: Two other important threats that weaken the validity of inferences about the statistical relationship between the 'cause' and the 'effect' are low statistical power and the violation of the assumptions of statistical tests. These other two threats are explored within Question 12).]*

12. Was appropriate statistical analysis used?

Inappropriate statistical analysis may cause errors of statistical inference with regards to the existence and the magnitude of the effect determined by the treatment ('cause'). Low statistical power and the violation of the assumptions of statistical tests are two important threats that weaken the validity of inferences about the statistical relationship between the 'cause' and the 'effect'. Check the following aspects: if the assumptions of statistical tests were respected; if appropriate statistical power analysis was performed; if appropriate effect sizes were used; if appropriate statistical procedures or methods were used given the number and type of dependent and independent variables, the number of study groups, the nature of the relationship between the groups (independent or dependent groups), and the objectives of statistical analysis (association between variables; prediction; survival analysis etc.).

13. Was the trial design appropriate for the topic, and any deviations from the standard RCT design accounted for in the conduct and analysis?

Certain RCT designs, such as the crossover RCT, should only be conducted when appropriate. Alternative designs may also present additional risks of bias if not accounted for in the design and analysis.

Crossover trials should only be conducted in people with a chronic, stable condition, where the intervention produces a short term effect (i.e. relief in symptoms). Crossover trials should ensure there is an appropriate period of washout between treatments.

Cluster RCTs randomize groups of individuals, forming 'clusters.' When we are assessing outcomes on an individual level in cluster trials, there are unit-of-analysis issues, as individuals within a cluster are correlated. This should be taken into account by the study authors when conducting analysis, and ideally authors will report the intra-cluster correlation coefficient.

Stepped-wedge RCTs may be appropriate when it is expected the intervention will do more good than harm, or due to logistical, practical or financial considerations in the roll out of a new treatment /intervention. Data analysis in these trials should be conducted appropriately, taking into account the effects of time.

For peer review only

Appendix 3.3: JBI Critical appraisal Checklist for Quasi-Experimental Studies (non-randomized experimental studies)

JBI Critical Appraisal Checklist for Quasi-Experimental Studies
(non-randomized experimental studies)

Reviewer____Date____

-

Author ____Year__________________Record Number ________

	Yes	No	Unclear	Not applicable
1. Is it clear in the study what is the 'cause' and what is the 'effect' (i.e. there is no confusion about which variable comes first)?				
2. Were the participants included in any comparisons similar?				
3. Were the participants included in any comparisons receiving similar treatment/care, other than the exposure or intervention of interest?				
4. Was there a control group?				
5. Were there multiple measurements of the outcome both pre and post the intervention/exposure?				
6. Was follow up complete and if not, were differences between groups in terms of their follow up adequately described and analyzed?				
7. Were the outcomes of participants included in any comparisons measured in the same way?				
8. Were outcomes measured in a reliable way?				
9. Was appropriate statistical analysis used?				

Overall appraisal: Include Exclude Seek further info

Comments (Including reason for exclusion)

____

____

____

For peer review only

Appendix 3.4: Discussion of JBI appraisal criteria for Quasi-Experimental Studies (non-randomized experimental studies)

Explanation for the critical appraisal tool for Quasi-Experimental Studies (experimental studies without random allocation)

Critical Appraisal Tool for Quasi-Experimental Studies (experimental studies without random allocation)

Answers: Yes, No, Unclear or Not Applicable

1. Is it clear in the study what is the 'cause' and what is the 'effect' (i.e. there is no confusion about which variable comes first)?

Ambiguity with regards to the temporal relationship of variables constitutes a threat to the internal validity of a study exploring causal relationships. The 'cause' (the independent variable, that is, the treatment or intervention of interest) should occur in time before the explored 'effect' (the dependent variable, which is the effect or outcome of interest). Check if it is clear which variable is manipulated as a potential cause. Check if it is clear which variable is measured as the effect of the potential cause. Is it clear that the 'cause' was manipulated before the occurrence of the 'effect'?

2. Were the participants included in any comparisons similar?

The differences between participants included in compared groups constitute a threat to the internal validity of a study exploring causal relationships. If there are differences between participants included in compared groups there is a risk of selection bias. If there are differences between participants included in the compared groups maybe the 'effect' cannot be attributed to the potential 'cause', as maybe it is plausible that the 'effect' may be explained by the differences between participants, that is, by selection bias. Check the characteristics reported for participants. Are the participants from the compared groups similar with regards to the characteristics that may explain the effect even in the absence of the 'cause', for example, age, severity of the disease, stage of the disease, co-existing conditions and so on? *[NOTE: In one single group pre-test/post-test studies where the patients are the same (the same one group) in any pre-post comparisons, the answer to this question should be 'yes.']*

3. Were the participants included in any comparisons receiving similar treatment/care, other than the exposure or intervention of interest?

In order to attribute the 'effect' to the 'cause' (the exposure or intervention of interest), assuming that there is no selection bias, there should be no other difference between the groups in terms of treatments or care received, other than the manipulated 'cause' (the intervention of interest). If there are other exposures or treatments occurring in the same time with the 'cause', other than the intervention of interest, then potentially the 'effect' cannot be attributed to the intervention of interest, as it is plausible that the 'effect' may be explained by other exposures or treatments, other than the intervention of interest, occurring in the same time with the intervention of interest. Check the reported exposures or interventions received by the compared groups. Are there other exposures or treatments occurring in the same time with the intervention of interest? Is it plausible that the 'effect' may be explained by other exposures or treatments occurring in the same time with the intervention of interest?

4. **Was there a control group?**

Control groups offer the conditions to explore what would have happened with groups exposed to other different treatments, other than to the potential 'cause' (the intervention of interest). The comparison of the treated group (the group exposed to the examined 'cause', that is, the group receiving the intervention of interest) with such other groups strengthens the examination of the causal plausibility. The validity of causal inferences is strengthened in studies with at least one independent control group compared to studies without an independent control group. Check if there are independent, separate groups, used as control groups in the study. *[Note: The control group should be an independent, separate control group, not the pre-test group in a single group pre-test post-test design.]*

5. Were there multiple measurements of the outcome both pre and post the intervention /exposure?

For peer review only

In order to show that there is a change in the outcome (the 'effect') as a result of the intervention /treatment (the 'cause') it is necessary to compare the results of measurement before and after the intervention/treatment. If there is no measurement before the treatment and only measurement after the treatment is available it is not known if there is a change after the treatment compared to before the treatment. If multiple measurements are collected before the intervention/treatment is implemented then it is possible to explore the plausibility of alternative explanations other than the proposed 'cause' (the intervention of interest) for the observed 'effect', such as the naturally occurring changes in the absence of the 'cause', and changes of high (or low) scores towards less extreme values even in the absence of the 'cause' (sometimes called regression to the mean). If multiple measurements are collected after the intervention/treatment is implemented it is possible to explore the changes of the 'effect' in time in each group and to compare these changes across the groups. Check if measurements were collected before the intervention of interest was implemented. Were there multiple pre-test measurements? Check if measurements were collected after the intervention of interest was implemented. Were there multiple post-test measurements?

6. Was follow up complete and if not, were differences between groups in terms of their follow up adequately described and analyzed?

If there are differences with regards to the loss to follow up between the compared groups these differences represent a threat to the internal validity of a study exploring causal effects as these differences may provide a plausible alternative explanation for the observed 'effect' even in the absence of the 'cause' (the treatment or exposure of interest). Check if there were differences with regards to the loss to follow up between the compared groups. If follow up was incomplete (that is, there is incomplete information on all participants), examine the reported details about the strategies used in order to address incomplete follow up, such as descriptions of loss to follow up (absolute numbers; proportions; reasons for loss to follow up; patterns of loss to follow up) and impact analyses (the analyses of the impact of loss to follow up on results). Was there a description of the incomplete follow up (number of participants and the specific reasons for loss to follow up)? If there are differences between groups with regards to the loss to follow up, was there an analysis of patterns of loss to follow up? If there are differences between the groups with regards to the loss to follow up, was there an analysis of the impact of the loss to follow up on the results?

7. Were the outcomes of participants included in any comparisons measured in the same way?

If the outcome (the 'effect') is not measured in the same way in the compared groups there is a threat to the internal validity of a study exploring a causal relationship as the differences in outcome measurements may be confused with an effect of the treatment or intervention of interest (the 'cause'). Check if the outcomes were measured in the same way. Same instrument or scale used? Same measurement timing? Same measurement procedures and instructions?

8. Were outcomes measured in a reliable way?

Unreliability of outcome measurements is one threat that weakens the validity of inferences about the statistical relationship between the 'cause' and the 'effect' estimated in a study exploring causal effects. Unreliability of outcome measurements is one of different plausible explanations for errors of statistical inference with regards to the existence and the magnitude of the effect determined by the treatment ('cause'). Check the details about the reliability of measurement such as the number of raters, training of raters, the intra-rater reliability, and the inter-raters reliability within the study (not to external sources). This question is about the reliability of the measurement performed in the study, it is not about the validity of the measurement instruments/scales used in the study. *[Note: Two other important threats that weaken the validity of inferences about the statistical relationship between the 'cause' and the 'effect' are low statistical power and the violation of the assumptions of statistical tests. These other threats are not explored within Question 8, these are explored within Question 9.]*

9. Was appropriate statistical analysis used?

Inappropriate statistical analysis may cause errors of statistical inference with regards to the existence and the magnitude of the effect determined by the treatment ('cause'). Low statistical power and the violation of the assumptions of statistical tests are two important threats that weakens the validity of inferences about the statistical relationship between the 'cause' and the 'effect'. Check the following aspects: if the assumptions of statistical tests were respected; if appropriate statistical power analysis was performed; if appropriate effect sizes were used; if appropriate statistical procedures or methods were used given the number and type of dependent and independent variables, the number of study groups, the nature of the relationship between the groups (independent or dependent groups), and the objectives of statistical analysis (association between variables; prediction; survival analysis etc.).

For peer review only - http://bmjopen.bmj.com/site/about/guidelines.xhtml

Chapter 4: Systematic reviews of text and opinion

Alexa McArthur, Jitka Klugarova, Hu Yan, Silvia Florescu

How to cite:

McArthur A, Klugarova J, Yan H, Florescu S. Chapter 4: Systematic reviews of text and opinion. In: Aromataris E, Munn Z (Editors). *JBI Manual for Evidence Synthesis*. JBI, 2020. Available from https://synthesismanual.jbi.global. https://doi.org/10.46658/JBIMES-20-05

Chapter 4: Contents

- 4.1 Text and opinion reviews and evidence-based practice
    - 4.1.1 Evidence, the practice gap and re-consideration of textual evidence
    - 4.1.2 The synthesis of text and opinion
- 4.2 Protocol development for reviews of textual, non-research evidence
    - 4.2.1 Title development and author information
    - 4.2.2 Protocol title
    - 4.2.3 Background
    - 4.2.4 Review objectives/questions
    - 4.2.5 Inclusion criteria
    - 4.2.6 Search strategy
    - 4.2.7 Assessment of methodological quality
    - 4.2.8 Textual data extraction
    - 4.2.9 Data synthesis
    - 4.2.10 References
- 4.3 The systematic review and synthesis of text and opinion data
    - 4.3.1 Title of systematic review
    - 4.3.2 Review authors
    - 4.3.3 Executive summary
    - 4.3.4 Main body of the report
        - 4.3.4.1 Background
        - 4.3.4.2 Review objectives/questions
        - 4.3.4.3 Inclusion criteria
        - 4.3.4.4 Search strategy
        - 4.3.4.5 Method of the review
            - 4.3.4.5.1 Assessment of methodological quality/critical appraisal
            - 4.3.4.5.2 Textual data extraction
            - 4.3.4.5.3 Textual data synthesis
        - 4.3.4.6 Results
            - 4.3.4.6.1 Description of publications
            - 4.3.4.6.2 Methodological quality
            - 4.3.4.6.3 Findings of the review
        - 4.3.4.7 Discussion
        - 4.3.4.8 Conclusions
        - 4.3.4.9 References
    - 4.3.5 Appendices
- 4.4 Chapter references
- Appendix 4.1: JBI Critical Appraisal Checklist for Text and Opinion Papers
- Appendix 4.2: Explanation of Text and Expert Opinion critical appraisal tool
- Appendix 4.3: Textual data extraction form for text and opinion publications

For peer review only

4.1 Text and opinion reviews and evidence-based practice

An evidence-based healthcare approach plays a major role in the clinical decision-making process. Every decision made by a healthcare professional should be based on the best available evidence, clinical experience and patient preferences. The best available evidence is usually understood as statistically proven results of primary or secondary quantitative study. Over the last three decades, results from qualitative studies have also been considered as scientific evidence. However, in the absence of evidence derived from rigorous primary research studies, what are the options? What is the best available evidence when quantitative and qualitative studies are missing?

Expert opinion has a role to play in evidence-based health care, as it can be used to either complement empirical evidence or, in the absence of research studies, stand alone as the best available evidence. While rightly claimed not to be a product of 'good' science, expert opinion is empirically derived and mediated through the cognitive processes of practitioners who have been typically trained in scientific method. This is not to say that the superior quality of evidence derived from rigorous research is to be denied; rather, that in its absence, it is not appropriate to discount expert opinion as non-evidence. (The Joanna Briggs Institute, 2014)

Text and opinion-based evidence (which may also be referred to as non-research evidence) is drawn from expert opinions, consensus, current discourse, comments, assumptions or assertions that appear in various journals, magazines, monographs and reports. (The Joanna Briggs Institute, 2014)(Sackett et al, 1996)(Tonelli, 2006)(Woolf, 2006) An important feature of using opinion in evidence based practice "is to be explicit when opinion is used so that readers understand the basis for the recommendations and can make their own judgment about validity." (W oolf 2000, p.364)   It is also important to highlight that one expert opinion is not as valid as a synthesis of the opinion of a group of experts, as displayed in the formation of consensus guidelines.

For peer review only

4.1.1 Evidence, the practice gap and re-consideration of textual evidence

Evidence based healthcare focuses on the need to use interventions that are supported by the most up-to-date evidence or knowledge. Many clinical aspects of healthcare cannot be fully explored by evidence derived from quantitative and/or qualitative research designs alone, since many areas of clinical care are supported by clinicians' tacit knowledge derived from their clinical experiences or the dominant healthcare discourse at the time of practice. (Jordan, Konno & Mu, 2011) It is clearly recognized that diverse knowledge/evidence types are required to inform practice, and for this reason comprehensive systematic review methods have been formulated to explore not only the evidence on the effectiveness of interventions ("knowing what" type of evidence), but also evidence related to subjective human experiences, culture, values, ethics, health policy, or the accepted discourse at the time of practice ("knowing how" type of evidence). (Jordan, Konno & Mu, 2011)

For peer review only

4.1.2 The synthesis of text and opinion

The synthesis of expert opinion findings within the systematic review process is not well recognized in mainstream evidence-based practice and it is acknowledged that efforts to appraise often conflicting opinions are tentative. However, in the absence of research studies, the use of a transparent systematic process to identify the best available evidence drawn from text and opinion can provide practical guidance to practitioners and policy makers. “Textual evidence should be understood as the narrative expression of clinical wisdom from health professionals.” (Jordan, Konno & Mu, 2011, p.19) It may also draw on the expertise of consumer representatives that are aligned with affiliated organizations.

For some clinical questions, there is an absence or paucity of quantitative and/or qualitative research studies, and in these situations, textual evidence can be promoted to understand narratively expressed experiences/tacit knowledge on a topic of interest. Textual evidence is, according to Mattingly (1991) and Worth (2008), the narrative expression of clinical wisdom from health professionals. Narrative knowledge does not fall into a conventional academic reasoning system of induction and deduction, but it is possible that health professionals can receive content-specific guidance and insights into how to improve their everyday practice based on the synthesis of textual evidence. This may also relate to the current discourse, or the verbal interchange of ideas that is grounded in language and in the context within which it occurs. Discourse in the professional and public domains is a source of knowledge that can be used to inform policy and clinical decision making.

When would you undertake a systematic review of text and opinion?

There are broadly three indications for undertaking a review of text and opinion.

1. As an adjunct to a quantitative or qualitative review where there are no research studies identified.
2. As an adjunct to a comprehensive systematic review, where the text and opinion component may provide supplemental evidence to the quantitative or qualitative reviews. (An example of this is a comprehensive systematic review conducted on the best evidence for assisted bathing of older adults with dementia (Konno et al, 2013))
3. As a stand alone review to investigate:

- People’s opinions/thoughts/conclusions
- Discourse analysis
- Policy analysis (an example of this is a systematic review looking at local and national policy and practice initiatives in relation to maternal mortality. McArthur & Lockwood, 2013))

The nature of textual or opinion based reviews is that they do not rely upon evidence in the form of primary research and, therefore, elements of the protocol will vary from reviews drawing on primary research as the types of papers of interest. However, the principals of developing a clearly documented protocol, incorporating a priori criteria and methods are – as for any systematic review – considered essential.

For peer review only - http://bmjopen.bmj.com/site/about/guidelines.xhtml

4.2 Protocol development for reviews of textual, non-research evidence

JBI systematic reviews of narrative, text and opinion-based evidence are conducted using the JBI System for the Unified Management, Assessment and Review of Information (SUMARI) which includes modules for reviews of different evidence types. The text and opinion module is designed to assist reviewers to appraise, extract and analyze and synthesize data from textual and expert opinion based evidence. To use the software, reviewers need to register through the JBI website and obtain a username and password.

Before developing their protocol, and to prevent review duplication, reviewers should search at least the Cochrane Library, *JBI Evidence Synthesis*, PubMed, and the PROSPERO database to establish whether a similar protocol or review has recently been published. Reviewers should also establish whether the size of the evidence base warrants conducting the review. The importance of this first step in the systematic review process cannot be overstated. This is for two reasons:

(i) because the development of the background section in the review, which includes providing the rationale for the review and definition of key concepts, helps reviewers to define the scope of the review and establishes its value (i.e. its contribution to the evidence base and potential offerings to decision makers);

(ii) because a priori setting of inclusion and exclusion criteria reduces the risk of introducing bias into the review, thereby promoting validity of its findings.

This guidance for developing the protocol for a JBI systematic review of text and opinion-based evidence is organized to meet the structure/template requirements for submission to the *JBI Evidence Synthesis*. An a-priori protocol must be developed before undertaking the review of text and opinion, and should contain the following sentence;

"The objectives, inclusion criteria and methods for this text and opinion review were specified in advance and documented in a protocol." (citation)

For submission to the *JBI Evidence Synthesis*, US English spelling and the Vancouver style referencing should be used.

For peer review only

4.2.1 Title development and author information

The protocol title should be informative and give clear indication of the topic of the systematic review. The title should always include the phrase "……: a systematic review protocol" to allow easy identification of the type of document it represents. All reviews using JBI methodologies require at least two reviewers in order to minimize reporting bias. The names of all reviewers, institutional affiliations and JBI center affiliations, and email address for the corresponding author must be included.

For peer review only

4.2.2 Protocol title

While a number of mnemonics have been discussed in the sections on quantitative and qualitative protocol development, and can be used for text and opinion, not all elements necessarily apply to every text or opinion-based review, and use of mnemonics should be considered a guide rather than a policy.

The following are examples of titles:

1. Competencies and skills to enable effective care of severely obese patients undergoing bariatric surgery across a multi-disciplinary healthcare perspective: a systematic review protocol.

2. Policies that promote age-friendly cities and enhance the wellbeing of inner city dwelling older adults: a systematic review protocol.

For peer review only

4.2.3 Background

The background should describe and situate the elements of the review, regardless of whether a particular mnemonic is used or not. The background should provide sufficient detail on each of the important elements of the review question to justify the conduct of the review and the choice of the various elements of the review. The author should particularly highlight the absence of scientific evidence, and verify the reasons for conducting a review of text and opinion.

JBI places significant emphasis on an extensive, comprehensive, clear and meaningful background section to every systematic review. Given the international circulation of systematic reviews, variations in local understandings of clinical practice, health service management and client or patient experiences need to be clearly stated. It is often as important to justify why elements are not to be included.

For peer review only

4.2.4 Review objectives/questions

The objectives guide and direct the development of the specific review criteria. Clarity in the objectives and specificity in the review questions assists in developing a protocol, facilitates more effective searching, and provides a structure for the development of the full review report. The review objectives must be stated in full. Conventionally, a statement of the overall objective is made and elements of the review are then listed as review questions. With reviews of text and opinion, consideration needs to be given to the phrasing of objectives and specific questions as causal relationships are not established through evidence of this nature, hence cause and effect type questions should be avoided.

The review objectives or questions should reflect key elements of the inclusion criteria (see below).

Questions to consider:

Does the background cover all the population, phenomenon of interest and the context for the systematic review? Are operational definitions provided? Do systematic reviews already exist on the topic? Why is this review important? Are the review objectives/questions clearly defined?

For peer review only

4.2.5 Inclusion criteria

Population/type of participants (P)

Describe the population, giving attention to whether specific characteristics of interest, such as age, gender, level of education or professional qualification are important to the question. These specific characteristics should be stated. Specific reference to population characteristics, either for inclusion or exclusion should be based on a clear justification rather than personal reasoning. The term population is used but not to imply that aspects of population pertinent to quantitative reviews such as sampling methods, sample sizes or homogeneity are either significant or appropriate in a review of text and opinion.

Pregnant and birthing women who received care from a skilled birth attendant within Cambodia, Thailand, Malaysia and Sri Lanka (McArthur & Lockwood, 2013)

Intervention / phenomena of interest (I)

Is there a specific intervention or phenomena of interest? As with other types of reviews, interventions may be broad areas of practice management, or specific, singular interventions. However, reviews of text or opinion may also reflect an interest in opinions around power, politics or other aspects of health care other than direct interventions, in which case, these should be described in detail.

The review will consider publications that describe: 1. The health system/service delivery structures and underlying policy; 2. The maternity care provided by a skilled birth attendant. (McArthur & Lockwood, 2013)

Context (Co) / Consequence (Co)

It is important to consider the context, or the consequences (impact) which will be the focus of the review, and must be specified within the inclusion criteria.

The use of a comparator, or a specific outcome statement is not required, as in a textual systematic review there are no measurable outcomes – only the outcomes (or consequences) as reported from the synthesis of expert opinion in the absence of non-research evidence.

Types of publications

Reviews of text and opinion consider publications reporting on expert opinion, which may be from standards for clinical care, consensus guidelines, expert consensus, narrative case reports, literature reviews including expert opinion, published discussion papers, conference proceedings, government policy reports or reports accessed from web pages of professional organizations.

This review considered government reports, expert opinion, discussion papers,

position papers, and other forms of text, published in the English language. Technical reports, statistical reports and epidemiological reports were excluded. (McArthur & Lockwood, 2013)

For peer review only

4.2.6 Search strategy

This section should flow naturally from the criteria that have been established to this point, and particularly from the objective and questions the review seeks to address. As reviews of opinion do not draw on published research as the principal designs of interest, the reference is to types of 'text' or 'narrative' publications, rather than types of 'studies'.

Searching for text and opinion evidence

As recommended in all JBI types of reviews, a three-step search strategy should be utilized, and detailed in this section of the protocol. A research librarian should be consulted to assist with development of a search strategy for textual evidence.

There are a range of databases that are relevant to finding expert opinion based literature. Examples include CINAHL, PubMed, CRD database from the NHS Centre for Reviews and Dissemination, University of York, PsycINFO, and National Guideline Clearing House. Grey literature searching is also of importance in a text and opinion review, depending on the clinical focus. Government websites, and contacting relevant organizations may also be beneficial in developing your search strategy. The search strategy of the published and unpublished literature also depends on the types of text specified in the inclusion criteria.

Search terms for text and opinion papers

Search filters are pre-tested strategies that identify articles based on criteria such as specified words in the title, abstract and keywords e.g. testimony, expert opinion. They can be of use to restrict the number of articles identified from the vast amount of literature in the major databases. Search filters look for sources according to relevance, not the quality of the article or citation itself. Quality judgments are performed separately and require skills in critical appraisal.

As with all types of systematic reviews conducted through JBI, the search strategy does need to reflect current international standards for best practice in literature searching. JBI SUMARI includes the following editable statement on searching:

The search strategy aims to find both published and unpublished studies. A three-step search strategy will be utilized in this review. An initial limited search of MEDLINE and CINAHL will be undertaken followed by analysis of the text words contained in the title and abstract, and of the index terms used to describe article. A second search using all identified keywords and index terms will then be undertaken across all included databases. Thirdly, the reference list of all identified reports and articles will be searched for additional studies. Studies published in #insert language(s)# will be considered for inclusion in this review. Studies published #insert dates# will be considered for inclusion in this review.

The databases to be searched include:

#insert text#

The search for unpublished studies will include:

#insert text#

Initial keywords to be used will be:

#insert text#

The protocol should also include a list of databases to be searched. If unpublished papers are to be included, the specific strategies to identify them are also described, and lists of key words per database are also recorded.

For peer review only

4.2.7 Assessment of methodological quality

Expert opinion – whether expressed by an individual, by a learned body or by a group of experts in the form of a consensus guideline – draws on the experience of practitioners. It may also draw on the expertise of consumer representatives that are aligned with affiliated organizations. However, the opinion of experts is more than just their practical experience; it is based on their understanding of the knowledge and experience; moreover it is the expression of these opinions in writing, and publishing in journals, magazines, webpages, etc. So, we should also consider the risk of “speech bias” according to the circumstances in which they expressed their opinions.

Thus, validity in this context relates to the soundness of opinion in terms of its logic and its ability to convince, the authority of the source and the quality of the opinion that renders it supportable. Although expert opinion is non-research evidence, it is empirically derived and mediated through the cognitive processes of practitioners who have typically been trained in scientific methods.

The focus then of appraisal is on authenticity: specifically, authenticity of the opinion, its source, the possible motivating factors and how alternate opinions are addressed. It is also focused on the assessment of credibility of the expert voice, and decision as to whether the arguments are logical. The items of appraisal are standardized for this type of literature, while the methods are the same as for appraisal of any type of literature. Standardized appraisal criteria require the primary and secondary reviewer to meet or electronically discuss the criteria to ensure a common understanding, then to apply them individually to each paper. Once both primary and secondary reviewers have conducted appraisal, any discrepancies in opinion are discussed and a mutual decision agreed upon. It is JBI policy that all systematic reviews need to be critically appraised using the JBI SUMARI critical appraisal checklist for text and opinion papers.

For peer review only

4.2.8 Textual data extraction

This section of the protocol should detail what textual data is to be extracted and the tool that will be used for extracting that data. JBI reviewers are required to use the text and opinion data extraction tool which can be found in Appendix I. This template data extraction tool may be adapted by reviewers as required. Textual data extraction serves the same purpose across evidence types - as in the previous modules that considered quantitative, qualitative and economic evidence, extraction aims to facilitate the accurate retrieval of important data that can be identified from many papers and summarized into a single document. An extraction is a summary of the main details of the publication and should be conducted after carefully reading the publication. Textual data extraction incorporates several fields relating to the type of text, its authors and participants, then the content of the paper in the form of conclusions.

Textual data extraction involves transferring conclusions from the original publication using an approach agreed upon and standardized for the specific review. Thus, an agreed format is essential to minimize error, provide an historical record of decisions made about the data in terms of the review, and to become the data set for categorization and synthesis. Specifically, the reviewer is seeking to extract the conclusions drawn by the author or speaker and the argument that supports the conclusion. The supporting argument is usually a quotation from the source document and is cited by page number with the conclusion if using JBI SUMARI. Many text and opinion based reports do not report conclusions explicitly. It is for this reason that reviewers are required to read and re-read each paper closely to identify the conclusions to be generated into JBI SUMARI. Conclusions should be extracted as verbatim statements from the author. It is recommended that double textual data extraction is performed independently by two reviewers. This aims to reduce errors in textual data extraction, assisted by using a standardized extraction tool.

For peer review only

4.2.9 Data synthesis

This section of the protocol should include details of how the extracted data will be synthesized. The aim of meta-aggregation is to: firstly, assemble conclusions; secondly, categorize these conclusions into categories based on similarity in meaning; and thirdly, to aggregate these to generate a set of statements that adequately represent that aggregation. These statements are referred to as synthesized findings and they can be used as a basis for evidence-based practice. In order to facilitate this process, as with ensuring a common understanding of the appraisal criteria and how they will be applied, reviewers need to discuss synthesis and work to common understandings on the assignment of categories, and assignment to synthesized findings.

JBI SUMARI describes a particular approach to the synthesis of textual papers. As with meta-aggregation in the qualitative module, synthesis is a three-step analytical process undertaken within the text and opinion module. Textual papers will, where possible be pooled using JBI SUMARI. This involves the aggregation or synthesis of conclusions to generate a set of statements that represent that aggregation, through assembling and categorizing these conclusions on the basis of similarity in meaning. These categories are then subjected to a meta-synthesis in order to produce a single comprehensive set of synthesized findings that can be used as a basis for evidence-based practice. Where textual pooling is not possible the conclusions are presented in narrative form.

The aim of synthesis is for the reviewer to establish synthesized findings by bringing together key conclusions drawn from all of the included papers. The units of extraction in this process are specific conclusions stated by the author/speaker and the text that demonstrate the argument or basis of the conclusion. Conclusions are principal opinion statements embedded in the paper and are identified by the reviewer after examining the text in the paper. It is for this reason that reviewers are required to read and re-read the paper closely to identify the conclusions to be entered into JBI SUMARI. Conclusions should be extracted as verbatim statements from the author.

Assessing Confidence

The final synthesized findings will be graded according to the ConQual approach for establishing confidence in the output of textual synthesis and presented in a Summary of Findings table. (Munn et al, 2014) The Summary of Findings table includes the major elements of the review and details how the ConQual score is developed. Included in the table is the title, population, phenomena of interest and context for the specific review. Each synthesized finding from the review is then presented along with the type of research informing it, a score for dependability, credibility, and the overall ConQual score.

For peer review only

4.2.10 References

Protocols are required to use the Vancouver referencing style. References should be numbered in the order in which they appear with superscript Arabic numerals in the order in which they appear in text. Full reference details should be listed in numerical order in the reference section.

More information about the Vancouver referencing style is detailed in: http://openjournals.net/files/Ref/VANCOUVER%20Reference%20guide.pdf

Appendices

Appendices should be placed at the end of the protocol and be numbered with Roman numerals in the order in which they appear in text. Following the PRISMA-P, JBI protocols will now include a completed search strategy for one database as Appendix 1. The critical appraisal tool should only be appended if modified in some way, otherwise it should be referenced. Data extraction tools should still be appended.

Does the protocol have any conflicts of interests and acknowledgments declared, appendices attached, and references in the Vancouver referencing style?

For peer review only

4.3 The systematic review and synthesis of text and opinion data

This section provides further guidance on components that should be included in the final report of a JBI text and opinion review, and information regarding each component as found in JBI SUMARI. Please refer to publication criteria for the *JBI Evidence Synthesis* for specific presentation requirements for systematic review reports. For further information please refer to the Author Guidelines of the journal.

All JBI systematic reviews are based on approved peer reviewed and published systematic review protocols. Deviations from approved protocols should be clearly justified in the report. JBI advocates approved peer reviewing of systematic review protocols as an essential part of a process to enhance the quality and transparency of systematic reviews.

Layout of the report

The systematic review protocol details how the review will be conducted, what outcomes are of interest and how the data will be presented. The systematic review report should be the follow up to an approved protocol – any deviations from the protocol need to be clearly detailed in the report, to maintain transparency. JBI SUMARI software provides a detailed framework for the necessary sections of a report.

The review report generated in JBI SUMARI can be exported as a word document which then requires editing. The tense needs to be changed from future to past tense, as the text used in the protocol is transferred into the subsequent report. All content must be reviewed and headings checked for relevancy, and references reviewed and finalized. Additional work will be required, to add narrative description and interpretation in the results section to develop the discussion, conclusions and inferences for research, practice and/or policy.

Briefly, a JBI review should contain the following sections:

Title

Reviewers

Executive summary (including Summary of Findings table; Keywords)

Background

Objectives/questions

Methods:

- *Inclusion criteria*
- *Search strategy*
- *Methodological quality assessment*
- *Data extraction and synthesis method*

Results

- *Description of studies (including PRISMA flow diagram)*
- *Methodological quality*
- *Findings of the review*

Discussion

Conclusion

- *Implications for practice*
- *Implications for research*

Conflict of interest

Acknowledgements

References

Appendices

- Appendix I: Search strategy
- Appendix II: Textual data extraction tool
- Appendix III: Articles excluded at full text appraisal with reasons
- Appendix IV: Table of characteristics of included studies

For peer review only

4.3.1 Title of systematic review

The title should be clear, explicit and reflect the core elements of the review. This should be the same as detailed in the protocol, with the phrase "……: a systematic review."

For peer review only

For peer review only - http://bmjopen.bmj.com/site/about/guidelines.xhtml

4.3.2 Review authors

The names and the JBI affiliation should be listed for each reviewer. An email address should be provided for the corresponding author.

For peer review only

4.3.3 Executive summary

The executive summary should be a comprehensive yet concise summary of the purpose, scope, methods, findings and implications of the systematic review. It should contain no abbreviations or references, be limited to 500 words and accurately reflect the review content. The executive summary includes the following headings:

Background

Objectives

Inclusion criteria

- **Participants**
- **Intervention or Phenomena of interest**
- **Context or Consequences**
- **Types of text and opinion papers**

Search strategy

Methodological quality

Data collection

Data synthesis

Results

Conclusions

Implications for practice

Implications for research

Keywords

Summary of findings table

Systematic reviews of text and opinion should consider presenting the final synthesized findings in a summary of findings table. This will be graded according to the ConQual approach for establishing confidence in the output of textual synthesis and presented in a Summary of Findings table. (Munn et al, 2014)

Table 4.1 ConQual summary of findings table

Table 1 ConQual summary of findings example

Systematic review title: the patient experience of high technology medical imaging: a systematic review of the qualitative evidence

Population: persons who had undergone high technology medical imaging

Phenomena of interest: the meaningfulness of a patients experience of undergoing diagnostic imaging using high technology

Context: male and female adult patients presenting to a medical imaging department

Synthesised finding	Type of research	Dependability	Credibility	ConQual score
People undergoing imaging often expect a health issue to be found during their scan, which can then	Qualitative	Downgrade 1 level*	Downgrade 1 level**	Low

* Downgraded one level due to common dependability issues across the included primary studies (the majority of studies had no statement locating the researcher and no acknowledgement of their influence on the research).
** Downgraded one level due to a mix of unequivocal and equivocal findings.

For peer review only

4.3.4 Main body of the report

The following sections should make up the main body of the report:

Background

Review objectives/questions

Inclusion criteria

Search strategy

Method of the review

Textual data extraction

Textual data synthesis

Results

Discussion

Conclusion

References / Appendices

We will go into more detail with each section.

For peer review only

4.3.4.1 Background

As discussed in the protocol section, JBI places significant emphasis on a comprehensive, clear and meaningful background section to every systematic review particularly given the international circulation of systematic reviews, variation in local understandings of clinical practice, health service management and client or patient experiences. It is recommended that all JBI systematic reviews should contain a sentence clearly indicating:

'*This review was conducted according to an a priori published protocol*.' (The reference should be inserted for the appropriate citation in *JBI Evidence Synthesis*).

This sentence should appear as the final line of the background/introduction section of the review report and complies with the recommendations for reporting of systematic reviews detailed in the PRISMA guidelines.

For peer review only

4.3.4.2 Review objectives/questions

As discussed previously in the protocol section, the objective(s) of the review should be clearly stated. Conventionally a statement of the overall objective should be made and elements of the review then listed as review questions.

For peer review only

4.3.4.3 Inclusion criteria

As detailed in the protocol, the inclusion criteria used to determine consideration for inclusion should be stated.

Types of participants

There should be details about the type of individuals targeted including characteristics (e.g. age range), condition/diagnosis or health care issue (e.g. administration of medication in rural areas and the setting[s] in which the individuals are being managed). Again the decisions about the types of participants should have been justified in the background.

Types of interventions / phenomena of interest

There should be a list of all the interventions or phenomena of interest examined. In some cases it may be appropriate to list categories of interventions. This section should be concise as the background section provides the opportunity to describe the main aspects.

Context / Consequence

It is important to consider the context, or the consequences (impact) that will be the focus of the review.

Types of publications

This section should flow from the background. There should be a statement about the target type of text and opinion, e.g. medicine, nursing, physical therapy, education, psychology, sociology, etc.

For peer review only

4.3.4.4 Search strategy

Developing a search strategy for opinion and text-based evidence

This section should document how the reviewers searched for relevant papers to include in the text and opinion review. The search strategy needs to be comprehensively reported. The search strategies for all of the databases searched should be presented sequentially in the single appendix. The timeframe chosen for the search should be justified and any language restrictions stated (e.g. only studies published in English were considered for inclusion). The databases that were searched must be listed along with the search dates. Any hand searching of relevant journals should be described by journal name and years searched. Author contact, if appropriate, should also be included with the results of that contact.

For peer review only

4.3.4.5 Method of the review

For peer review only

4.3.4.5.1 Assessment of methodological quality/critical appraisal

This section of the review should include the results of critical appraisal with the JBI critical appraisal checklist for text and opinion papers, embedded in the JBI SUMARI software. As discussed in the section on protocol development, it is JBI policy that textual evidence should be critically appraised using the text and opinion module. The primary and secondary reviewer should discuss each item of appraisal for each study design included in their review.

In particular, discussions should focus on what is considered acceptable to the needs of the review in terms of the characteristics of the text and opinion. The reviewers should be clear on what constitutes acceptable levels of information to allocate a positive appraisal compared with a negative, or response of 'unclear' or 'not applicable.' This discussion should take place before conducting the appraisal as each publication in a review should be assessed independently by both reviewers. The critical appraisal tool should be referenced accordingly.

Critical appraisal of text or expert opinion

The focus on limiting bias to establish validity in the appraisal of quantitative studies is not possible when dealing with text and opinion. In appraisal of text, the opinions being raised are vetted, the credibility of the source investigated, the motives for the opinion examined, and the global context in terms of alternate or complementary views are considered.

Validity in this context therefore relates to what is being said, the source and its credibility and logic; and consideration of the overt and covert motives at play. The explanation for the JBI SUMARI text and opinion critical appraisal tool is detailed further in Appendix I and II.

Has the JBI SUMARI text and opinion critical appraisal tool been referenced correctly in the review? Have the results of critical appraisal been discussed? Where there any differences of opinion between the reviewers?

For peer review only

4.3.4.5.2 Textual data extraction

This section of the review should include details of the types of data extracted for inclusion in the review. Data extraction begins with recording the type of text. Once data extraction of the background details is complete, the extraction becomes highly specific to the nature of the data of interest and the question being asked in the review. In JBI SUMARI, elements of data extraction are undertaken through the text and opinion analytical module, and the data extracted is automatically transferred to the exported report.

Extracting data from text and opinion

As detailed in the protocol section, this section of the review should include details of the types of data extracted for inclusion in the review. An extraction in JBI SUMARI includes fields relating to the type of text, its authors and participants, and the content of the paper. Textual data (conclusions) are extracted from papers included in the review using the standardized data extraction tool for text and opinion reviews. The data extracted will include specific details about the phenomena of interest, populations, and any outcomes of significance to the review question and specific objectives.

It is recommended that double textual data extraction is performed independently by two reviewers.

The specific fields and types of text to extract are as follows: (see Appendix III)

1. Types of text

The type of opinion that is being extracted, for example, an expert opinion, a consensus guideline, conference proceedings, policy reports or reports accessed from web pages of professional organizations.

2. Population represented

To whom the paper refers or relates.

3. Setting / Context (may be clinical, cultural or geographical)

Setting is the specific location where the opinion was written, for example, a nursing home, a hospital or a dementia specific ward in a sub-acute hospital. Some papers will have no setting at all.

The geographical context is the location of the author(s) – be as specific as possible, for example Poland, Austria, or rural New Zealand.

The cultural context is the cultural features in the publication setting, such as, but not limited to, time period (16th Century); ethnic groupings (indigenous Australians); age groupings (e.g. older people living in the community); or socio-economic groups (e.g. working class). When entering information it is important to be as specific as possible. This data should identify cultural features such as time period, employment, lifestyle, ethnicity, age, gender, and socio-economic class or context.

4. Stated allegiance/position

A short statement from the expert voice summarizing the main thrust of the publication.

5. Conclusion (with illustration from text and page number)

Use this field to describe the main finding/s of the publication. This includes an assessment of the clarity of the argument's presentation and logic. Is other evidence provided to support assumptions and conclusions? Is it based on clinical or life experience?

Levels of credibility (Unequivocal/Credible/Not Supported) can be assigned in this section (see further detail in data synthesis section)

6. Reviewer's conclusion

Use this field to summarize the strengths and weaknesses of the paper.

7. Notes

This section of the report should include any other notes the reviewer wants to make. It may also include techniques that have been used to analyze the data, e.g. named software program.

Has the NOTARI data extraction tool been appended to the review? Have all of the extracted findings been discussed and assigned levels of credibility in the review?

For peer review only

4.3.4.5.3 Textual data synthesis

As the process relates to textual findings rather than numeric data, the need for methodological homogeneity – so important in the meta-analysis of the results of quantitative studies – is not a consideration.

This section of the report should include how the findings were synthesized. Where meta-aggregation is possible, textual findings should be pooled using JBI SUMARI. The units of extraction in this process are specific conclusions stated by the author/speaker and the text that demonstrate the argument or basis of the conclusion. Conclusions are principal opinion statements embedded in the paper and are identified by the reviewer after examining the text in the paper; the conclusion is the claim or assertion of the author. It is for this reason that reviewers are required to read and re-read the paper closely to identify the conclusions to be entered into JBI SUMARI. Conclusions should be extracted as verbatim statements from the author.

The processes for categorization and formulating synthesized ndings mirror that of the JBI SUMARI qualitative module. For a more detailed discussion of synthesis, reviewers are encouraged to read the section on data synthesis for qualitative studies.

Data synthesis should involve the aggregation or synthesis of findings to generate a set of statements that represent that aggregation, through assembling the findings rated according to their credibility, and categorizing these findings on the basis of similarity in meaning. These categories should then be subjected to a meta-synthesis in order to produce a single comprehensive set of synthesized findings that can be used as a basis for evidence-based practice. Where textual pooling is not possible the findings can be presented in narrative form.

Prior to carrying out data synthesis, reviewers first need to establish, and then document:

- their own rules for setting up categories
- how to assign conclusions (findings) to categories
- how to aggregate categories into synthesized findings.

In JBI SUMARI, a reviewer can add conclusions to a study after an extraction is completed on that paper.

The JBI approach to synthesizing the conclusions of textual or non-research studies requires reviewers to consider the credibility (logic, authenticity) of each report as a source of guidance for practice; identify and extract the conclusions from papers included in the review; and to aggregate these conclusions as synthesized findings.

The most complex problem in synthesizing textual data is agreeing on and communicating techniques to compare the conclusions of each publication. The JBI approach uses the SUMARI analytical module for the meta-synthesis of opinion and text. This process involves categorizing and re-categorizing the conclusions of two or more studies to develop synthesized ndings. Reviewers should also document these decisions and their rationale in the systematic review report.

Many text- and opinion-based reports do not state conclusions explicitly. It is for this reason that reviewers are required to read and re-read each paper closely to identify the conclusions to be generated into JBI SUMARI.

Each conclusion/finding should be assigned a level of credibility, based on the congruency of the finding with supporting data from the paper where the finding was found. Textual evidence has three levels of credibility; thus, the reviewer is required to determine if, when comparing the Conclusion with the argument the Conclusion represents evidence that is:

Unequivocal - relates to evidence beyond reasonable doubt which may include conclusions that are matter of fact, directly reported/observed and not open to challenge

Credible - relates to those conclusions that are, albeit interpretations, plausible in light of the data and theoretical framework.

Not Supported - is when the findings are not supported by the data

In the systematic review report, it may be set out in the following way.

Papers were pooled using JBI SUMARI. This involved a three stage process:

1. Extraction of Level 1 author's conclusions from full text articles and rating each according to its assessed validity (unequivocal, credible, not supported).

2. Categories were developed and assigned (Level 2 conclusions) based on similarity of meaning of Level 1 conclusions.

3. A set of synthesized conclusions were developed (Level 3 conclusions) after subjecting the categories to meta-synthesis. This represents the meta-aggregation of Level 1 and Level 2 conclusions.

For peer review only - http://bmjopen.bmj.com/site/about/guidelines.xhtml

4. Recommendations for practice and research were developed from the meta-syntheses and graded according to JBI Grades of Recommendation.

Have all of the conclusions been extracted from the included papers? Do all of the conclusions have illustrations? Do all of the conclusions have levels of credibility assigned to them?

For peer review only

4.3.4.6 Results

For peer review only

For peer review only - http://bmjopen.bmj.com/site/about/guidelines.xhtml

4.3.4.6.1 Description of publications

The presentation of results should identify how many studies were identified and selected. There should be a narrative description of the search decision process accompanied by the search decision flowchart. This section should include the type and number of papers identified by the search and the number of studies that were included and excluded from the review. A flowchart of this is shown in Figure 4.1. A flowchart of search results

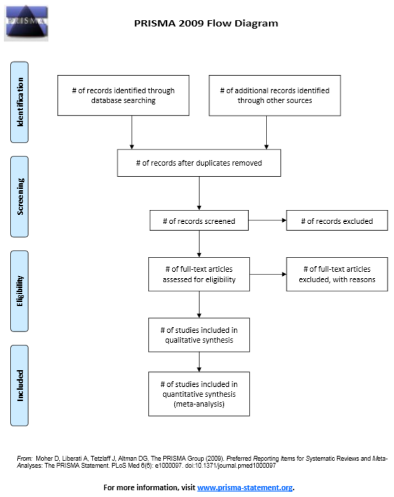

The results section should be framed in such a way that as a minimum, the following fields are described or given consideration by the reviewers in preparing their systematic review report:

Papers: Number of studies identified, number of retrieved papers, number of appraised papers, number of excluded papers and overview of reasons for exclusion, and number of included papers.

The results section then focuses on providing a detailed description of the results of the review. Where a systematic review has several foci, the results should be presented in a logical, structured way, relevant to the specific questions. The role of tables and appendices should not be overlooked. Adding extensive detail on studies in the results section may 'crowd' the findings, making them less accessible to readers, hence the use of tables, graphs and in text reference to specific appendices is encouraged.

For peer review only

4.3.4.6.2 Methodological quality

This section should focus on the methodological quality as determined by the JBI SUMARI text and opinion critical appraisal checklist. There should be a narrative summary of the overall methodological quality of the included studies, which can be supported by a table showing the overall results of the critical appraisal. (See Table 4.2 for example)

Table 1: Final methodological quality assessment table

Citation	Q1	Q2	Q3	Q4	Q5	Q6	Q7
Wright, K., Bauer, C., 2005	Y	Y	Y	Y	N	N	Y
McIntyre, T., Jones, D.B., 2005	Y	Y	N	Y	Y	Y	U

Y – Yes, N – No, U – Unclear

Table 4.2 Final methodological quality assessment table

For peer review only

4.3.4.6.3 Findings of the review

There is no standardized international approach to structuring how the findings of systematic reviews of textual or non-research evidence should be reported. The audience for the review should be considered when structuring and writing up the findings. Meta-Aggregative Flowcharts represent a specific item of analysis that can be incorporated into the results section of a review. However, the results are more than the Meta-Aggregative Flowcharts, and whether it is structured based on the intervention of interest, or some other structure, the content of this section needs to present the results with clarity using the available tools supported by textual descriptions.

Given there is no clear international standard or agreement on the structure or key components of this section of a review report, and the level of variation evident in published systematic reviews, the parameters described in this section should be considered as guidance for consideration rather than a prescription.

The results section then focuses on providing a detailed description of the results of the review. For clarity and consistency of presentation, JBI recommends that the reviewers, in discussion with their review panel give consideration to whether the ndings can be reported under the outcomes specified in the protocol.

Where a systematic review seeks to address multiple questions, the results may be structured in such a way that particular outcomes are presented under specific questions.

When all conclusions and supporting illustrative data have been identified, the reviewer needs to read all of the conclusions and identify similarities that can then be used to create categories of more than one finding.

Categorization is the first step in aggregating conclusions and moves from a focus on individual papers to consideration of all conclusions for all papers included in the review. Categorization is based on similarity in meaning as determined by the reviewers. Once categories have been established, they are read and re-read in light of the findings, their illustrations and in discussion between reviewers to establish synthesized findings. JBI SUMARI sorts the data into a meta-aggregative flowchart, when allocation of categories to synthesized findings (a set of statements that adequately represent the data) is completed. (see Figure 4.2) These statements can be used as a basis for evidence-based practice.

Synthesized conclusions

Conclusion	Category	Synthesised Finding
Bariatric surgeons should become members of IFSO or another adhering body and each surgeon, prior to the beginning of practice, should have taken an IFSO course or has attended an IFSO meeting. (C)	Organizational structure for a bariatric surgery service	Safe, effective and meaningful care for bariatric surgery patients may require a minimum set of competencies for managing a bariatric surgery unit and MDT. Managerial Competencies
Outcomes for the development of a bariatric surgery program, include nursing competency and site survey. (C)		
Patient and staff safety is a key consideration in bariatric surgical nursing care and nursing staff should be trained to use patient handling equipment. (C)		
The IFSO statement on bariatric surgeon qualifications states that bariatric surgery should only take place where there is adequate provision of equipment and staff. (C)		
A range of topics related to surgical management are recommended for educating nurse practitioners (C)	Required competencies for managing a MDT in bariatric surgery	
Bariatric surgery related management should be part of education for nurse practitioners caring for obese patients. (C)		
CNS collaboration may enhance provision of safe care and follow-up for bariatric surgery patients. (C)		
Core competencies for the CNS include expertise in clinical care, consultation, professional guidance, leadership, collaboration (C)		
Nurses can coordinate education within the multidisciplinary team and address key factors related to bariatric surgical care. (C)		
The bariatric program coordinator (CNS) requires leadership skills and clinical expertise. (C)		

Figure 4.2: Example of a meta-aggregative flowchart (Stephen et al, 2014)

For peer review only

4.3.4.7 Discussion

This section should provide a detailed discussion of issues arising from the conduct of the review, as well as a discussion of the findings of the review, and to demonstrate the significance of the review findings in relation to practice and research. Areas that may be addressed include:

- A summary of the major findings of the review
- Issues related to the quality of the research within the area of interest (such as poor indexing)
- Other issues of relevance
- Implications for practice and research, including recommendations for the future
- Potential limitations of the systematic review (such as a narrow search timeframe or other restrictions).

The discussion does not bring in new literature or findings that have not been reported in the results section but does seek to establish a line of argument based on the findings regarding the phenomenon of interest, or its impact on the outcomes identified in the protocol.

For peer review only

4.3.4.8 Conclusions

This section should begin with an overall conclusion based on the results. The conclusions drawn should match the review objective/question.

Implications for practice

Where evidence is of a sufficient level, appropriate recommendations should be made. The implications must be based on the documented results, not reviewer opinion. Recommendations must be clear, concise and unambiguous, and assigned a JBI Grade of Recommendation. This will be based on a consideration of the conclusions (whether a mixture of unequivocal, credible or not supported), and be reported as Grade A (a 'strong' recommendation) or Grade B (a 'weak' recommendation). (The Joanna Briggs Institute, 2014)

Implications for research

All implications for research must be derived from the results of the review, based on identified gaps, or on areas of weakness in the literature such as professional credibility of the authors. Implications for research should avoid generalized statements calling for further research, but should be linked to specific issues (such as longer follow up periods). Recommendations must be clear, concise and unambiguous.

For peer review only

4.3.4.9 References

For publication in the *JBI Evidence Synthesis*, all references should be listed in full using the Vancouver referencing style, in the order in which they appear in the review. Abbreviated journal titles must be used in accordance with the United States National Library of Medicine.

For peer review only

4.3.5 Appendices

Appendices should be numbered using Roman numerals in the order in which they have been referred to in the body of the text. While reviewers may choose to develop additional appendices for details that are unfeasible to present in the main body of the report, there are two required appendices for a JBI text and opinion review:

Appendix I: Search strategy

A detailed and complete search strategy for all of the major databases and other sites and sources searched must be appended. Major databases that were searched must be identified, including the search platform used where necessary. All search filters with logic employed should be displayed, including the number of records returned.

Appendix II: Data extraction instrument

The data extraction instrument used must be appended (see the template in Appendix 4.3)

Appendix III: Excluded studies

Insert table of excluded studies. Where studies have been excluded based on not meeting inclusion criteria or being of insufficient quality, these need to be listed separately.

Appendix IV: List of study findings / conclusions / characteristics of included studies

A table of included studies is crucial to allow a snapshot of the studies included in the review.

For peer review only

4.4 Chapter references

Jordan, Z, Konno, R, Mu, PF 2011, 'Synthesizing evidence from narrative, text and opinion.' *Synthesis Science in Healthcare Series: Book 3* Lipincott-Joanna Briggs Institute.

Konno, R, Stern, C, Gibb, H 2013, 'The best evidence for assisted bathing of older people with dementia: a comprehensive systematic review,' *JBI Database of Systematic Reviews and Implementation Reports,*1 1:123-212.

Mattingly, C 1991, 'The narrative nature of clinical reasoning,' *Am J Occup Ther,* 45:998-1005.

McArthur, A, Lockwood, C 2013, 'Maternal mortality in Cambodia, Thailand, Malaysia and Sri Lanka: a systematic review of local and national policy and practice initiatives,' *The JBI Database of Systematic Reviews and Implementation Reports,*11:115-86.

Moher, D, Liberati, A, Tetzlaff, J & Altman, DG 2009, 'Preferred reporting items for systematic reviews and meta-analyses: the PRISMA statement', *Ann Intern Med*, vol. 151, ed. 4, pp. 264-9.

Munn, Z, Porritt, K, Lockwood, C, Aromataris, E, Pearson, A 2014, 'Establishing confidence in the output of qualitative research synthesis: the ConQual approach,' *BMC Medical Research Methodology,.* 14;108.

Sackett, D, Rosenberg, J, Gray, J & Haynes R 1996, 'Evidence based medicine: what it is and what it isn' t,' *British Medical Journal,* 312:71-2.

Stephen, A, Bermano, G, Bruce, D, Kirkpatrick, P 2014, 'Competencies and skills to enable effective care of severely obese patients undergoing bariatric surgery across a multi-disciplinary healthcare perspective: a systematic review,' *The JBI Database of Systematic Reviews and Implementation Reports,* 12:321-97.

The Joanna Briggs Institute 2014, 'The Joanna Briggs Institute Reviewers' Manual: 2014 Edition.' Adelaide: *The Joanna Briggs Institute.*

The Joanna Briggs Institute Levels of Evidence and Grades of Recommendation Working Party 2014, *Supporting Document for the Joanna Briggs Institute Levels of Evidence and Grades of Recommendation:* The Joanna Briggs Institute, Adelaide, viewed 20 March 2017, https://jbi.global/about-jbi/jbi-approach-to-EBHC#tabbed-nav=Levels-of-Evidence

Tonelli, M 2006, 'Integrating evidence into clinical practice: an alternative to evidence-based approaches,' *Journal of Evaluation in Clinical Practice,* 12:248-56.

Woolf, S 2000, 'Evidence based medicine and practice guidelines: an overview,' *Cancer Control,* 7:362-7.

Worth, SE 2008, 'Storytelling and narrative knowing: An examination of the epistemic benefits of well-told stories,' *J Aesthetic Education*, 42:42-56.

For peer review only

Appendix 4.1: JBI Critical Appraisal Checklist for Text and Opinion Papers

JBI Critical Appraisal Checklist for Text and Opinion Papers

Reviewer_ Date_ _

Author _ Year_ _ _ _ _ _ _ _ _ _ _Record Number _ _ _ _

	Yes	No	Unclear	Not applicable
1. Is the source of the opinion clearly identified?	☐	☐	☐	☐
2. Does the source of opinion have standing in the field of expertise?	☐	☐	☐	☐
3. Are the interests of the relevant population the central focus of the opinion?	☐	☐	☐	☐
4. Is the stated position the result of an analytical process, and is there logic in the opinion expressed?	☐	☐	☐	☐
5. Is there reference to the extant literature?	☐	☐	☐	☐
6. Is any incongruence with the literature/sources logically defended?	☐	☐	☐	☐

Overall appraisal: Include ☐ Exclude ☐ Seek further info ☐

Comments (Including reason for exclusion)

Appendix 4.2: Explanation of Text and Expert Opinion critical appraisal tool

Answers: Yes, No, Unclear or Not/Applicable

1. Is the source of the opinion clearly identified?

Is there a named author? Unnamed editorial pieces in journals or newspapers, or magazines give broader licence for comment, however authorship should be identifiable.

2. Does the source of opinion have standing in the field of expertise?

The qualifications, current appointment and current affiliations with specific groups need to be stated in the publication and the reviewer needs to be satisfied that the author(s) has some standing within the field.

3. Are the interests of the relevant population the central focus of the opinion?

The aim of this question is to establish the author's purpose in writing the paper by considering the intended audience. If the review topic is related to a clinical intervention, or aspect of health care delivery, a focus on health outcomes will be pertinent to the review. However, if for example the review is focused on addressing an issue of inter-professional behaviour or power relations, a focus on the relevant groups is desired and applicable. Therefore this question should be answered in context with the purpose of the review.

4. Is the stated position the result of an analytical process, and is there logic in the opinion expressed?

In order to establish the clarity or otherwise of the rationale or basis for the opinion, give consideration to the direction of the main lines of argument. Questions to pose of each textual paper include: What are the main points in the conclusions or recommendations? What arguments does the author use to support the main points? Is the argument logical? Have important terms been clearly dened? Do the arguments support the main points?

5. Is there reference to the extant literature?

If there is reference to the extant literature, is it a non-biased, inclusive representation, or is it a non-critical description of content specifically supportive of the line of argument being put forward? These considerations will highlight the robustness of how cited literature was managed.

6. Is any incongruence with the literature/sources logically defended?

Is there any reference provided in the text to ascertain if the opinion expressed has wider support? Consider also if the author demonstrated awareness of alternate or dominant opinions in the literature and provided an informed defence of their position as it relates to other or similar discourses.

For peer review only

Appendix 4.3: Textual data extraction form for text and opinion publications

Publication:

Publication						
Type of text	Population represented	Setting/<br>Context<br>(may be clinical, cultural, or geographical)	Stated allegiance/<br>position	Conclusion<br>Illustration from text & page no.<br>Unequivocal/Credible/Unsupported<br>(U/C/Un)	Reviewer's Conclusion	Notes

peer review only

Chapter 5: Systematic reviews of prevalence and incidence

Zachary Munn, Sandeep Moola, Karolina Lisy, Dagmara Riitano, Catalin Tufanaru

How to cite:

Munn Z, Moola S, Lisy K, Riitano D, Tufanaru C. Chapter 5: Systematic reviews of prevalence and incidence. In: Aromataris E, Munn Z (Editors). *JBI Manual for Evidence Synthesis.* JBI, 2020. Available from https://synthesismanual.jbi.global. https://doi.org/10.46658/JBIMES-20-06

Chapter 5: Contents

- 5.1 Introduction to systematic reviews of prevalence and incidence
- 5.2 Study designs reporting prevalence and incidence data
- 5.3 Systematic reviews of prevalence and incidence
  - 5.3.1 Indications for systematic reviews of prevalence and incidence
- 5.4 Developing a review protocol
  - 5.4.1 Protocol and review title
  - 5.4.2 Review question
  - 5.4.3 Inclusion criteria
    - 5.4.3.1 Types of participants (population)
    - 5.4.3.2 Condition
    - 5.4.3.3 Context
    - 5.4.3.4 Types of studies
  - 5.4.4 Search strategy
  - 5.4.5 Assessment of methodological quality
  - 5.4.6 Data extraction
  - 5.4.7 Data synthesis
- 5.5 Conducting and reporting systematic reviews of prevalence and incidence data
  - 5.5.1 Executive summary
  - 5.5.2 Background
  - 5.5.3 Review questions
  - 5.5.4 Inclusion criteria
  - 5.5.5 Search strategy
  - 5.5.6 Assessment of methodological quality
  - 5.5.7 Data extraction
  - 5.5.8 Data synthesis and meta-analysis
    - 5.5.8.1 Narrative and non-statistical summary
    - 5.5.8.2 Meta-analysis
  - 5.5.9 Results
  - 5.5.10 Discussion
  - 5.5.11 Conclusions
- 5.6 Chapter references
- Appendix 5.1: Critical Appraisal Instrument for Studies Reporting Prevalence Data
- Appendix 5.2: Data extraction form for prevalence studies

For peer review only

5.1 Introduction to systematic reviews of prevalence and incidence

The amount of literature in the health and social fields has increased at an exponential rate over the last 30 years and each year hundreds of thousands of new articles are being published in social and health journals. This increase in research output has been paralleled with a growing focus on the need for healthcare decisions, policies and funding to be based on the best available evidence, with consideration of patient preferences, clinical expertise and available resources. This need to base health and social care policy on evidence from research is now well-accepted internationally and is seen as the ideal way to practice healthcare. However, in real world settings this is not always the case.

There are many barriers that inhibit the uptake of research evidence into practice, one of which is the difficulty for the practicing professional to keep up to date with the expanding body of literature. The systematic review of evidence has been proposed and is well now accepted as the best method to summarize the literature relating to a certain social or healthcare topic. The systematic review is a type of research design that provides a reliable summary of the literature to assist health professionals to keep up to date. Key features of a systematic review include the creation of an a priori protocol, clear inclusion criteria, a structured and systematic search process, critical appraisal of studies, and a formal process of data extraction followed by methods to synthesize, or combine, this data. In this way, systematic reviews extend beyond the subjective, narrative reporting characteristics of a traditional literature review to provide a comprehensive, rigorous and transparent synthesis of the literature on a certain topic.

Historically reviews have focused on the synthesis of evidence of effects, particularly trying to establish the effectiveness of various treatments on social and health outcomes. However, decisions made in social and health care require more information than can be provided by the simple question 'does this work?' As systematic review methodology has evolved so have the types of evidence that have been synthesized using this approach. There now exists methods and guidance for conducting reviews of various forms of evidence, including qualitative research, cost data, diagnostics, prognostics, harms and risk.

Whilst the steps included in the systematic review process are mirrored across the various types of evidence, there are important considerations that need to be taken into account when conducting a systematic review pertaining to the type of research to be synthesized. There are established methods for conducting meta-analyses of randomized controlled trials and some observational study designs. However, no clear guidance currently exists on synthesizing frequency data from incidence and prevalence estimates. This chapter seeks to fill this gap by outlining methods and guidance for an emerging type of systematic review, that of prevalence and incidence data. Prevalence and incidence data systematic reviews are becoming more important as policy makers realize the usefulness of syntheses of this type of information. Synthesis of this type of information has the potential to better inform social and healthcare professionals, policy makers and consumers in decisions made relating to a range of healthcare decisions, but particularly regarding the burden of healthcare both now and in to the future. This chapter provides comprehensive guidance to authors wishing to conduct systematic reviews on prevalence and incidence data.

For peer review only

5.2 Study designs reporting prevalence and incidence data

Now that the measures of prevalence and incidence of disease have been discussed, it is important to detail the kinds of studies that are useful in obtaining this kind of data. Whilst randomized controlled trials (RCTs) are the best study design to answer questions of the effectiveness of interventions due to their ability to determine causality, they are not ideally suited to provide data of rates and patterns of disease occurrence. Having said that, certain prevalence or incidence data can be gleaned from RCTs, as discussed further below.

To address issues regarding prevalence and incidence, epidemiological study designs, such as those classified under the term observational and descriptive studies, are required. Observational studies do not involve manipulation on the part of the researcher. These studies rely on the natural or 'ecological' events of exposures and disease, where the researcher simply observes certain characteristics of the sample population as they occur "naturally", and records the relevant data. Observational studies can therefore be distinguished from experimental or quasi-experimental studies such as RCTs and controlled clinical trials, where there is manipulation of the independent variable (or the intervention) by the researcher. Observational studies have a number of advantages over experimental study designs and are particularly valuable in instances where conduct of an RCT is unethical. It is certainly unethical to conduct an RCT to investigate the effects of a variable that is thought to be harmful, such as the effect of alcohol use during pregnancy for example, or the effect of asbestos exposure. Questions such as these can only be addressed using observational studies, where an exposure, behavior or event occurs and the researcher observes participants over time to investigate any outcomes.

Observational studies address questions such as: how many people have the disease? Who is getting the disease? Where is the disease occurring? This kind of information is particularly valuable for governments when making decisions regarding health policy and planning. Furthermore, observational studies can often be used to infer correlations between two variables, for example, between a variable such as an exposure, risk factor or protective factor, and a disease outcome. Data from observational studies can therefore be useful in enabling the formation of hypotheses regarding risk or preventive factors in disease development and progression. It is important to note that these studies are not able to determine causality; rather they are able only to infer correlations or relationships between variables.

Observational study designs include prospective and retrospective cohort studies, case-control studies, cross-sectional studies, case series and case reports, and can be broken down into the broad categories of analytical studies and descriptive studies. Generally, descriptive studies describe the occurrence /presence of an outcome only, whereas analytical studies describe the relationship between a variable and an outcome. Some observational studies may report both analytic and descriptive data particularly in the case of certain case-studies and cross-sectional studies. Due to the nature of observational study designs, they are more at risk of confounding factors and different sources of bias that are unavoidable, which will be discussed further below. Despite this, observational studies are essential in answering questions of prevalence and incidence.

For peer review only

5.3 Systematic reviews of prevalence and incidence

The systematic review of prevalence and incidence data is important in the description of the geographical distribution of a variable, variation between subgroups (such as gender) and informing health care planning and resource allocation. Pooling of such data is necessary to monitor trends in disease burden and emergence and to contribute to the design of further etiological studies. Systematic reviews are of particular relevance where individual studies are limited by small sample sizes. The systematic review of studies to answer questions of prevalence and incidence data still follow the same basic principles of systematic review of other types of data. A protocol must be written for the conduct of the systematic review, comprehensive searching must be performed and critical appraisal of retrieved studies must be carried out.

For peer review only

5.3.1 Indications for systematic reviews of prevalence and incidence

There are many reasons to do a review of these types of data, including the following:

- They assist in answering questions of global disease burden
- Help to measure global design burden (incidence data can be used to determine disability adjusted life years)
- In cases where it is not practical to do a large global survey
- Questions larger than a national scale
- Cumulative meta-analysis can show changes and trends over time, and can highlight emerging or decreasing diseases
- Assists policy makers and funding models
- Informs geographical distributions and comparisons of subgroups
- Inform healthcare professionals of diseases and symptoms of disease
- Can compare prevalence between groups
- Inform further research priorities

The methods outlined in this book relate to systematic reviews of prevalence and incidence data. The data that is to be synthesized is therefore proportional data; that is, proportions (often percentages) of a population experiencing the particular disease or condition. However, the methods can be applied more broadly than this and do not necessarily need to focus on a disease. There are examples of reviews that have been conducted addressing issues such as the prevalence and incidence of medication errors, (Miller, Robinson, Lubomski, Rinke, & Pronovost, 2007) claustrophobic reactions in magnetic resonance imaging, (Munn, Moola, Lisy, Riitano, & Murphy, 2014) barriers to adherence with treatment (Mills et al., 2006), and electronic health record adoption.(Rao et al., 2008)

For peer review only

5.4 Developing a review protocol

A systematic review protocol is important because it pre-defines the objectives and methods of the systematic review. It is a systematic approach to the conduct and report of the review that allows transparency of the process, which in turn allows the reader to see how the findings and recommendations were arrived at. The protocol details the criteria the reviewers will use to include and exclude studies, to identify what data is important and how it will be extracted and synthesized. A protocol provides the plan or proposal for the systematic review and as such is important in restricting the presence of reporting bias. Any deviations between the protocol and systematic review report should be discussed in the systematic review report.

This section outlines the components of a systematic review protocol of prevalence and incidence evidence and provides guidance on the information that each component should contain. Specifically, it provides guidance on each of the following components: title page, title development, background, review objectives/questions, inclusion criteria, search strategy, critical appraisal, data extraction, data synthesis, narrative summary, conflict of interest, acknowledgements, references, and appendices. This guidance is based on the JBI approach to systematic reviews of prevalence and incidence.

For peer review only

5.4.1 Protocol and review title

The title should be clear, explicit and reflect the core elements of the protocol. Titles should not be phrased as questions or conclusions and there should be congruency between the title, review objectives /questions and inclusion criteria. The title needs to include the phrase ‘A systematic review protocol’.

The title should give an indication on the type of data that will be reported (descriptive, analytical or a combination of both) by including the epidemiological indicator or a term that reflects the analysis that will be used to measure the variables of interest. Generally, measures of disease should appear in the title (prevalence, incidence).

The factors or events of interest (health condition or disease of interest) are defined by the time period, the place and the population at risk. Accordingly, the title should specify the defining characteristics of the population (i.e., gender, age) as well as the place and time of occurrence where relevant.

For example: ‘Prevalence and incidence of depression amongst adolescents: A systematic review protocol.’

For peer review only

5.4.2 Review question

The review question(s) must be clearly stated.

The overarching aim of reviews of prevalence and incidence data is to report on the frequency, distribution and determinants of specific factors, health states or conditions in a defined population.

Reviews that aim to describe the distribution of existing variables or seek to answer the question: how common is a particular disease or condition in a specific group of individuals?' are often classified as descriptive and will utilise measures of prevalence and incidence to answer such lines of enquiry.

The objective of these reviews is to describe the health issue (what), those affected by it (who) as well as the location (where) and the time period (when) in which it occurred.

Accordingly, the review question should outline the factor, disease, symptom or health condition of interest, the epidemiological indicator used to measure its frequency (prevalence, incidence), the population or groups at risk, as well as the context/location (e.g., limited to specific geographic areas) and time period (e.g., peaks at a particular season) where relevant.

For example: The objective of this review is to assess the prevalence and incidence of peri-natal depression among women in Australia.

Reviews focusing on how and why are predominantly analytic in nature. The objective of reviews of explanatory or analytic studies is to contribute to and improve our understanding of the causes of health-related events or outcomes by isolating the association between specific factors. This element is non-existent or lacking in studies that are purely descriptive. While studies that report prevalence and incidence only are broadly classified as descriptive and those that examine associations between exposures and outcomes are broadly classified as analytical a clear-cut distinction between analytical and descriptive study designs is not possible. Data generated from these studies can be measured and reported in different ways and the review question will indicate whether the review seeks to report data that is descriptive, analytical or a combination of both.

For peer review only

5.4.3 Inclusion criteria

This section of the protocol details the basis on which studies will be considered for inclusion into the systematic review and should be as clear and unambiguous as possible.

When determining the inclusion criteria, the CoCoPop mnemonic (**Co**ndition, **Co**ntext and **Po**pulation) can be used for reviews assessing prevalence and incidence data.

For peer review only

5.4.3.1 Types of participants (population)

The types of participants should be appropriate for the review objectives. The reasons for the inclusion or exclusion of participants should be explained in the background.

It is important that the population or study subjects are clearly defined and described in detail. This includes outlining the specific or defining characteristics of the population such as age, sex, race gender, educational status, individual behaviour, socio-demographic factors etc.

For example, we will include studies involving adult pregnant women aged 18 – 45 years at any trimester and up to delivery.

Exclusion criteria should also be outlined where relevant. For example, studies examining pregnancies with neural tube defects, intra-uterine growth retardation and early pregnancy loss; and those involving adolescent pregnancies and anaemic mothers.

For peer review only

5.4.3.2 Condition

This refers to the variable of interest and may refer to a health condition, disease, symptom, event or factor. It is important that the variable of interest is clearly stated and defined. For example, Malaria could be P. falciparum infection, P. vivax infection or disease due to malarial infection. This may include providing information on how the condition will be measured, diagnosed, or confirmed.

For peer review only

5.4.3.3 Context

Environmental factors can have a substantial impact on the prevalence or incidence of a condition. Accordingly, it is important that authors define the context or specific setting relevant to their review question. For example, this may include defining the geographic area or country, specific community or setting (inpatient vs outpatient) and the time period given that some conditions may peak at a particular season (e.g. the incidence of influenza in different seasons and years).

For peer review only

5.4.3.4 Types of studies

Reviews of prevalence and incidence are predominantly derived from observational studies. A cross-sectional study is the appropriate study design to determine the prevalence of a particular health problem. Cross-sectional surveys are typically used to estimate the point prevalence of common conditions of long duration and are generally not appropriate for rare or temporary diseases. As incidence is the number of new cases of a particular illness within a population over time study participants need to be followed up. Therefore, cohort studies that have a prospective or longitudinal design and follow up each subject over a suitable period of time are the best way to establish the incidence of a disease or the natural history of a condition. However many study designs may provide prevalence and incidence information.

For peer review only

5.4.4 Search strategy

This section details how the reviewers plan to search for relevant papers. A review should consider papers published in both commercial and in non-commercially operated databases (grey literature). The timeframe chosen for the search should be justified and any language restrictions stated (e.g. only studies published in English will be considered for inclusion). The databases to be searched must be listed along with the initial keywords to be used for the search. Appropriate databases to search should be included, including specification from the outset of the platform used to search a particular database.

Prevalence and incidence data are reported within the published, peer-reviewed literature and accordingly the standard JBI search strategy can be applied to locating this type of evidence.

There are also many and various sources of epidemiological data, within the grey literature, particularly for estimates of prevalence and incidence.

Some examples include:

- Administrative sources (clinical records, insurance data)
- Vital statistics data, government surveillance data and reports, Centers for Disease Control and Prevention data, population censuses and surveys (i.e., national or state health survey data), health care utilization records and disease registries (population-based disease registries established to record cases of certain serious diseases).
- Disease associations (e.g., American Diabetes Association).
- Medical books, grey literature and reports from experts

What sources are chosen will obviously depend on the specific research question and its scope. For example, estimating the worldwide prevalence of a common condition (chronic disease) will need to include many more sources than a review examining the prevalence of a condition within a specific regional setting.

For peer review only

5.4.5 Assessment of methodological quality

The protocol should detail the criteria considered when determining methodological quality of papers to include in the review. JBI tools should be used preferentially; if not clear reasoning should be provided. Critical appraisal tools must be appended to the protocol. For questions assessing incidence, the critical appraisal tool should be selected based on the type of study design retrieved from the search process. However, as prevalence data may be sourced from a number of study designs (including RCTs), a critical appraisal checklist specifically for prevalence studies has been developed. Critical appraisal must be conducted by two reviewers independently of each other. The reviewers should then meet to discuss the results of their critical appraisal for their final appraisal. If the two reviewers disagree on the final critical appraisal and this cannot be resolved through discussion, a third reviewer may be required.

For peer review only

5.4.6 Data extraction

Standardized data extraction tools allow the extraction of the same types of data across the included studies and are required for JBI systematic reviews. The protocol should detail what data the reviewers plan to extract from the included studies and the data extraction tool should be appended to the protocol.

The data extraction sheet should be adapted to suit the collection and stratification of the variables of interest from the included studies. It is important to extract data that reflects points of difference /heterogeneous characteristics between studies that affect the interpretation of the findings and synthesis of data.

Whether data synthesis can be performed will depend on the heterogeneity of the variables of interest across included studies. To facilitate such a comparison it is critical data extraction details the variables that will be extracted and compared.

The description of disease patterns often includes analysis of demographic, geographical, social, seasonal and other risk factors.

It is also likely to include the setting/location, dates of survey, definitions of conditions and populations, inclusion and exclusion criteria, mean age, sex, sample size, estimates of prevalence, incidence etc.

Gender categorization, while important for sexually transmitted diseases and other diseases with a large gender gap may not be important for numerous other diseases. Geographical distribution is important to describe diseases linked to environmental conditions but may not be useful for other diseases.

For peer review only

5.4.7 Data synthesis

The protocol should detail how the reviewers plan to synthesize data extracted from included studies. The types of data it is anticipated will be synthesized should be consistent with the methods used for data collection and the included study designs. Refer to the next section for more detail on synthesis of prevalence and incidence data.

For peer review only

5.5 Conducting and reporting systematic reviews of prevalence and incidence data

This section provides information on how to synthesize evidence relating to prevalence and incidence data. It provides guidance on the components that should comprise a systematic review of prevalence and incidence data and the information that each component should contain. Specifically, guidance is provided on the following components: layout of the report, inclusion criteria (i.e. CoCoPop), search strategy, critical appraisal, data extraction, data synthesis, results, and conclusions. The section also presents a series of questions designed to prompt the reviewer to check that certain key information or requirements have been adequately addressed in the review. This guidance is based on the JBI approach to systematic reviews of prevalence and incidence.

For peer review only

5.5.1 Executive summary

This section forms a structured abstract of the main features of the systematic review. It must be no longer than 500 words and should contain no abbreviations or references. The executive summary must accurately reflect and summarize the systematic review. The executive summary should include the following headings:

Background

This section should briefly describe the issue under review including the population, condition and context that are documented in the literature. The background should be an overview of the main issues. It should provide sufficient detail to justify why the review was conducted and the choice of the various elements such as the condition and context.

Objectives

The review objectives should be stated in full, as detailed in the protocol section.

Inclusion criteria:

Population:

The report should provide details about the types of participants included in the review. Useful details include: age range, gender, profession, etc. Information supporting the decisions about the types of participants should be explained in the background.

*Condition***:** This section should present all the conditions examined, as detailed in the protocol.

*Context***:** This section should present all the contexts examined, as detailed in the protocol.

Studies: As per the protocol section, the types of studies that were considered for the review should be included. There should be a statement about the target study type and whether or not this type was not found. The types of study identified by the search and those included should be detailed in the report.

Search strategy

A brief description of the search strategy should be included. This section should detail search activity (e. g. relevant databases searched, initial search terms or keywords, and any limitations) for the review, as predetermined in the protocol.

Methodological quality

Reviewer's should make mention of how the studies included in the review were appraised.

Data extraction

This section should include a brief description of the types of data collected and the instrument (as specified in the protocol) used to extract data.

Data synthesis

This section should include a brief description of how the data was synthesised –as a meta-analysis or as a narrative summary or in a graphical form or in a tabular form.

Results

This section should include a brief description of the findings of the review.

Conclusions

This section should include a brief description of the conclusions of the review.

Implications for practice

This section should include a brief description of how the findings and conclusions of the review may be applied in practice, as well as any implications that the findings may have on current practice.

Implications for research

This section should include a brief description of how the findings of the review may lead to further research in the area- such as gaps identified in the body of knowledge.

For peer review only

5.5.2 Background

The background section should be comprehensive and cover all the main elements of the topic under review. Many reviewers will find that the background provided with the protocol needs modification or extension following the conduct of the review proper. The background should detail any definitions important to the review. The information in the background section must be sufficient to put the inclusion criteria into context. The background section should conclude with a statement that a preliminary search for previous systematic reviews on the topic was conducted (state the databases searched e.g. JBI Library, Cochrane Library, CINAHL, PubMed, PROSPERO). If there is a previous systematic review on the topic, it should be specified how the proposed review differs. Vancouver style referencing should be used throughout the review with superscript numbers without brackets used for in-text citations. JBI places significant emphasis on a comprehensive, clear and meaningful background section to every systematic review particularly given the international circulation of systematic reviews, variation in local understandings of clinical practice, health service management and client or patient experiences. It is recommended that all JBI systematic reviews should contain a sentence clearly indicating:

"*The objectives, inclusion criteria and methods of analysis for this review were specified in advance and documented in a protocol*. Ref" (The reference should be to the appropriate citation in *JBI Evidence Synthesis*, and provide registration number in PROSPERO where applicable).

This sentence should appear as the final line of the background/introduction section of the review report and complies with the recommendations for reporting of systematic reviews detailed in the PRISMA guidelines.

For peer review only

5.5.3 Review questions

As discussed previously in the protocol section, the question(s) of the review should be clearly stated.

For peer review only

5.5.4 Inclusion criteria

As detailed in the protocol, this section of the review should detail the basis on which studies were considered for inclusion in the systematic review and should be as clear and unambiguous as possible. For a systematic review of prevalence and incidence studies, aspects include: Population, Condition and Context.

For peer review only

5.5.5 Search strategy

This section should detail how the reviewers searched for relevant papers. The databases that were searched must be listed along with the search dates. The detailed search strategies for all of the major databases and other sources searched should be appended to the review. The documentation of search strategies is a key element of the scientific validity of a systematic review. It enables readers to look at and evaluate the steps taken, decisions made to consider the comprehensiveness and exhaustiveness of the search strategy for each included database. A JBI review should consider papers published in both commercial (e.g. PubMed, Cochrane, JBI Library etc.) and in non-commercially operated databases (grey literature).

Each electronic database is likely to use a different system for indexing key words within their search engines. Hence the search strategy will be tailored to each particular database. These variations are important and need to be captured and included in the systematic review repo

For peer review only

5.5.6 Assessment of methodological quality

This section should detail the approach to critical appraisal, not the assessment results, and should be consistent with the protocol. Any deviations from the protocol must be reported and explained. The report should detail the criteria that were considered when determining the methodological quality of papers considered for inclusion in the review.

Critical appraisal tools must be appended to the review.

The primary and secondary reviewer should discuss each item of appraisal for each study design included in their review. The discussions should focus on what is considered acceptable to the needs of the review in terms of the specific study characteristics. The reviewers should be clear on what constitutes acceptable levels of information to allocate positive appraisal compared with a negative, or response of “unclear”. This discussion should take place before independently conducting the appraisal. The critical appraisal tool should be appended to the review.

For peer review only

5.5.7 Data extraction

This section of the review should include details of the types of data extracted from the included studies. If no data was available for particular outcomes, that should also be discussed. Standardized data extraction tools allow the extraction of the same types of data across the included studies and are recommended for JBI systematic reviews. The included studies may include several outcomes; however, the review should focus on extracting information related to the research questions and outcomes of interest. Information that may impact upon the generalizability of the review findings such as study methods, setting and population characteristics should also be extracted and reported. Population characteristics include factors such as age, past medical history, co-morbidities, complications or other potential confounders. JBI aims to reduce errors in data extraction by using two independent reviewers and a standardized data extraction instrument. The data extraction tool used must be appended to the review.

The data collection should include the following items and a brief description is provided for each item (A data extraction form has been appended (Appendix II) with the following items listed):

Study details

- Reviewer – Mostly includes details or ID of the primary reviewer.
- Study ID/Record Number - is a numeric code to identify the study from which the effect size estimate was obtained
- Date – the date when this data extraction form was filled
- Study title – the full title of the study
- Author - This is an alphabetic or character code which is usually the first few characters of the primary study author's name. This serves as an easy way to identify the study in the bibliography
- Year – the year of publication
- Journal – the journal in which the article was published

Study Method

- Aims of the study – as stated in the report
- Setting – may refer to hospital or community or aged care facility. May also refer to rural/urban etc.
- Study design – briefly describing the type of study design. For e.g. if it is a randomised controlled trial or quasi-randomised controlled trial
- Follow-up or study duration – any details on the duration of the study or follow-up of the participants
- Subject characteristics – Includes age, sex, country/location, sample size, diagnosis and other relevant characteristics.
- Dependent variable -
- Outcomes – the primary outcome measured and where relevant includes associated secondary outcomes.
- Outcome measurements – describe the scales or tools used to measure the outcomes. For e.g. a standardised pain scale to measure pain.
- Ethical approval – yes/no
- Method of data analysis

Results

- Prevalence n/N (%)
- Proportion and 95% Confidence Intervals
- Incidence n/N (%)
- Proportion and 95% Confidence Intervals and duration of recruitment or the study
- Authors' comments

Reviewer comments

For peer review only

5.5.8 Data synthesis and meta-analysis

The data synthesized within a systematic review are the results extracted from individual research studies relevant to the review question. As much as meta-analysis is preferred, it is not always possible in a systematic review if the included studies vary greatly from each other, either in terms of how they are conducted (different interventions), who they are performed on (different populations) or in their final result. When meta-analysis isn't possible, common alternatives for the synthesis of quantitative data in a systematic review include narrative summary of results, vote counting, and presenting data via tables. Before discussing meta-analysis, alternative methods to synthesis are discussed.

For peer review only

5.5.8.1 Narrative and non-statistical summary

A narrative summary is commonly used where meta-analysis is not possible. A narrative summary describes the included studies and provides conclusions about the evidence. With a narrative summary, readers may not be able to discern how evidence was weighted and whether conclusions are biased. It is therefore important that when summarizing findings in narrative form, there is a clear structure to the summary, with an emphasis on reporting the characteristics of included studies along with data extracted relevant to the review outcomes. (Lockwood & White, 2012) Narrative summary in systematic reviews should be rigorous and clear, and can utilize tables, graphs, and other diagrams to help convey how studies compare to each other and to assist in presentation of the data.(Lockwood & White, 2012) A narrative summary should include the presentation of the quantitative results reported in individual studies; where available, the point estimates (one value that represent or best estimate of effects) and the interval estimates (usually presented as 95% confidence intervals) for the effects should be provided. Due to the flexibility of narrative summary in terms of the amount of data that can be conveyed textually, a structure that applies to each sequence or reporting of results from each study should be discussed beforehand and applied by the systematic review authors. This will ensure that there is consistency across the results section of a review. If a structure is not followed there may be substantial variability in reporting of results causing the data to appear incomplete or unreliable.(Lockwood & White, 2012) Therefore adherence to this structure is critical; if studies do not provide the relevant information to comply with the structure this should be made clear in the summary.(Lockwood & White, 2012) Bear in mind that there is no prescriptive guidance on presenting a narrative summary and it is recommended that the context of the review be taken into consideration.

Tables where relevant should be included to aid in the presentation of the data. Mostly, these tables include individual studies with their raw data; for example, percentages, distribution of prevalence and incidence estimates and confidence intervals. The tables should also include other elements such as participant characteristics.

The various graphs that may be useful in presenting include but are not limited to forest plot (for meta-analysis), funnel plot (for publication bias), L'abbe plot (explores heterogeneity and is applicable for meta-analysis of studies with binary outcomes), Galbraith plot (assesses the extent of heterogeneity between studies in a meta-analysis), and cumulative plot (incidence and prevalence estimates). However, it should be noted that the interpretation of the graphs is quite subjective and therefore should be interpreted with caution.

For peer review only

5.5.8.2 Meta-analysis

A meta-analysis is a statistical process that essentially calculates effect sizes for individual studies, converts them to a common metric, and then combines them to obtain an average effect size. (Field, 2001) This statistical combination increases the power of the overall estimate from various small individual studies as a result of the overall increase in the sample size. In addition, meta-analysis also enables reviewers to explore the differences between individual studies. Meta-analysis should only be undertaken when the studies are sufficiently similar to combine; in the absence of this homogeneity, the conclusion from the meta-analysis may be invalid. The findings may also depend on the selection and quality of the studies included and the availability of relevant data.

Where meta-analysis is used, the statistical methods and the software used should be described. Prior to a meta-analysis to be undertaken, relevant data needs to extracted. If the data is heterogeneous and is presented as a narrative summary, sources of heterogeneity should be discussed (e.g. clinical, methodological or statistical) as well as on what basis it was determined inappropriate to combine the data statistically (such as differences in populations, study designs or clinical or statistical heterogeneity).

There are established methods for conducting meta-analyses of randomized controlled trials and some observational study designs. However, no clear guidance exists on synthesizing frequency data from incidence and prevalence estimates. This section provides this guidance.

Effect size

The effect size statistically describes the relationship between two variables and is represented by a square on forest plot. In traditional effectiveness reviews, this could be the impact of a new therapy on mortality rates or the effect of a new teaching method on exam scores. The effect size could be a single number such as for a prevalence study or a ratio such as a risk ratio. The effect size has been described as being the “currency of the systematic review” as the aim of the meta-analysis is to summarize the effect size of each included study to obtain a summary effect.(Borenstein M, Hedges LV, Higgins JPT, & Rothstein HR, 2009) The summary effect is shown as a diamond on a forest plot. When effect sizes are statistically combined, the methods used make certain assumptions.

Statistical combination of data

In meta-analysis, the results of similar, individual studies are combined to determine the overall effect. In meta-analysis, the effect-size and weight of each study are calculated. The effect size indicates the direction and magnitude of the results of a particular study (i.e. do the results favor the treatment or control, and if so, by how much), while the weight is indicative of how much information a study provides to the overall analysis when all studies are combined together.

It has been suggested that there are three important criteria for choosing a summary statistic for meta-analysis: consistency of effect across studies, (ii) mathematical properties, and (iii) ease of interpretation. (Deeks & Altman, 2001)

Consistency of effect is important because the aim of meta-analysis is to bring together the results of several studies into a single result.

The main mathematical property required by summary statistics is the availability of a reliable variance estimate. Consensus about the other two mathematical properties (reliance on which of the two outcome states [e.g. mortality/survival] is coded as the event and odds ratio being the only statistic which is unbounded) has not yet been reached.

Ease of interpretation

Essentially there are three popular approaches to conduct meta-analysis for all types of data: Hedge and Olkin technique, Rosenthal and Rubin technique and the Hunter and Schmidt technique. Hedge and Olkin developed both fixed- and random-effects models for pooling data, Rosenthal and Rubin developed a fixed-effects model only and Hunter and Schmidt developed a random-effects model.

Statistical assumptions in meta-analysis

Meta-analysis can be based on either one of two statistical assumptions – fixed or random effects. It is important to distinguish between fixed- and random-effects models when conducting meta-analysis, as it can lead to false assumptions about statistical significance of the pooled estimate.

The main difference between fixed and random effects models is in the calculation of standard errors associated with the combined effect size. Fixed effects models use only within-study variability in their error term because all other ‘unknowns’ in the model are assumed not to affect the effect size. In contrast, in random effects models it is necessary to account for the errors associated with sampling from populations that themselves have been sampled from a superpopulation. As such the error term contains two components: within-study variability and variability arising from differences between studies. (Field, 2001)

For peer review only

The fixed effects model assumes that there is one true effect for the population underlying the studies in the analysis and that all the differences in the data are due to sampling error or chance within each study and that there is no heterogeneity between the studies. A fixed effect model is statistically stringent and should be used when there is little heterogeneity, as determined by Chi-square or I2 tests. This model therefore assumes that the overall sample consists of samples that all belong to the same underlying population.(Kock, 2009) The between-study variability will be zero in this model as it assumes that the population effect size is identical for all studies. In an analysis based on a fixed effects model, inference is applicable or generalizable ("conditional") based on statistical justification only on the studies actually done.(Petitti, 2000) The fixed effects model assumes that there is little interest or value in generalizing the results to other studies.(Fleiss, 1993; Munn, Tufanaru, & Aromataris, 2014)

A random effects model allows more flexibility, assuming that there may be other factors influencing the data than error or chance, within and between studies. As a result, in an analysis based on a random effects model, inference relies on the assumption that the studies used in the analysis are a random sample of some hypothetical population of studies. (Munn, Tufanaru, et al., 2014; Petitti, 2000) For example, the effect size may be influenced in studies where the participants are more educated, older or healthier or if a more intense intervention is being used. The effect size is assumed to follow a normal distribution and consequently has a mean and variance. The random-effects model considers both between-study variability and within-study variability. The random-effects model enables generalizations beyond the population included in the studies.

There is no consensus about whether fixed or random effects models should be used in meta-analysis. In many cases when heterogeneity is absent, the two methods will give similar overall results. When heterogeneity is present, the random effects estimate provides a more conservative estimate of the overall effect size, and is less likely to detect significant differences. For this reason, random effects models are sometimes employed when heterogeneity is not severe; however, the random effects model does not actually analyze the heterogeneity away and should not be considered as a substitute for a thorough investigation into the reasons for heterogeneity. Additionally, random effects models give relatively more weight to the results of smaller studies – this may not be desirable because smaller studies are typically more prone to bias and are often lower quality than larger studies.

There are a number of meta-analytical techniques available – the selection of a particular technique is governed by three things: the study type, the nature of the data extracted and the assumptions underlying the meta-analysis.

Meta-analysis of prevalence and incidence data - Proportions

Prevalence and incidence data is often reported as a proportion. When pooling proportions for meta-analysis, a transformation of the data is required. There are two main ways to transform the data, the Freeman-Tukey transformation (arcsine square root transformation), and the Logit transformation, both of these are used to calculate the weighted summary proportion under the fixed and random effects model. The resultant meta-analysis will give pooled proportion with 95% CI both for the fixed effects model and the random effects model and in addition, will list the proportions (expressed as a percentage), with their 95% CI, found in the individual studies included in the meta-analysis. The results are then presented graphically in a forest plot. For all meta-analyses, prevalence estimates are transformed to logits to improve their statistical properties. These are then back-transformed to prevalence.

Converting proportions (p) to logits:(Sutton, Abrams, Jonas, Sheldon, & Song, 2000)

Logit = log(odds) = log(p/1?p).

Using the number of cases with an event (Nevent) and without an event (N-event), the variance of logit is given by

Var(logit)=1/Nevent +1N-event.

There are different models for performing the meta-analysis as mentioned above. Normally the reviewer is provided with a choice of using the Mantel-Haenszel model or the DerSimonian and Laird model. We recommend that the meta-analyses of the prevalence reported in the studies is grouped by a random-effects model and is presented with 95% confidence intervals (95% CI). Random effects model are used when there is sufficient information on standard errors. However, bear in mind that the random-effects model gives a conservative estimate with a wider confidence interval. The random effects model allows for between-study variation by assuming that the individual study prevalence estimates follow a normal distribution. The fixed model can be chosen but the reviewer should be aware of its underlying principles, particularly in relation to its assumption that there is one true effect, which may not hold for prevalence and incidence data.

Heterogeneity of the results is tested by the I-squared, Tau-squared, Cochran's Q test and Chi-squared ($p > 0.05$) tests. These tests of heterogeneity evaluate whether the differences in prevalence estimates across studies are higher than expected by chance. To identify the sources of heterogeneity across studies, subgroup analysis or meta-regression can be used to assess the contribution of each variable (i. e. year of study, geographic location, characteristic of countries, study population etc.) to the overall heterogeneity. Those variables significantly associated with the heterogeneity ($p < 0{\cdot}05$) can be included in a multivariate hierarchical model. A p value of <0·05 is considered statistically significant in all the analyses.

For peer review only - http://bmjopen.bmj.com/site/about/guidelines.xhtml

Below is an example of a table of studies that were combined in a meta-analysis. These studies reported on overall termination rates for scans in the general MRI population.

Study	Events	Sample	%
Dantendorfer 1997	2	297	0.673400673
Dewey 2007 (all)	1004	55734	1.801413859
Eshed 2007	59	4821	1.223812487
Lang et al (2010)*	336	34521	0.973320587
Nawaz 2009*	58	2630	2.205323194
Sarji 1998	18	3324	0.541516245
Wiebe 2004	14	1790	0.782122905

Figure: Meta-analysis of scan termination due to claustrophobia in general scan types

The figure above represents meta-analysis of proportion data using random effects model from the seven studies. There was significant heterogeneity present in the studies, with a Cochran Q test reaching statistical significance and an I2 value of 96.1%. The pooled proportion equaled 1.18% (95% CI 0.79 – 1.65). However, due to the significant heterogeneity, this value should be interpreted with caution.

There are limitations with conducting meta-analysis of frequency data, including: (Saha, Chant, & McGrath, 2008)

Heterogeneity of data: If the data from the included studies are heterogenous, then the standard errors or confidence intervals for a pooled effect estimate will not adequately reflect the variability of underlying data.

Inadequate reporting of frequency estimates: standard error (SE) for each estimate is required to weight the estimate when pooling the data. Standard errors can still be calculated if the data for the numerator, denominator and the duration of the study were available; however, these calculated SEs will not take into account various adjustments.

How to interpret effect sizes?

Once authors calculate effect sizes, they need to answer the question: What does the effect size mean?

An effect size is simply a number and its meaning and importance must be explained by the researcher. An effect size of any magnitude can mean different things depending on the research that produced it and the results of similar past studies. Therefore, it is the researcher’s responsibility to discuss the importance of their findings and this information requires comparing current effects to those obtained in previous work in the same research area. Confidence Intervals (CIs) are an important way to evaluate the precision of a study’s findings by providing a range of likely values around the obtained effect size.

Heterogeneity

When used in relation to meta-analysis, the term ‘heterogeneity’ refers to the amount of variation in characteristics of included studies. For example, if three studies are to be included in a meta-analysis, does each of the included studies have similar demographics, and assess the same intervention? While some variation between studies will always occur due to chance alone, heterogeneity is said to occur if there are significant differences between studies, and under these circumstances meta-analysis is not valid and should not be undertaken.

There are three types of heterogeneity; clinical, methodological, and statistical heterogeneity.(Higgins & Thompson, 2002) Differences in the characteristics of study populations and measurements represent clinical heterogeneity. Differences in study designs and methodological quality (risk of bias) represent methodological heterogeneity. Statistical heterogeneity is the variation of effects sizes between studies. Statistical heterogeneity may arise because of clinical heterogeneity, methodological heterogeneity, or simply by chance.

For peer review only

There is often heterogeneity amongst studies addressing prevalence and incidence. This is due to a number of reasons. Firstly, clinical heterogeneity may be present due to the measures used to determine the presence of a variable.(Webb et al., 2005) For example, different scales exist to measure depression, and depending on the scale used, a person may be classified as suffering from depression whilst using one scale and not suffering based on a different scale. Additionally, prevalence and incidence studies often look at specific populations at a specific point of time, and the scope of the study may be limited by state or national borders. Another consideration with the population is whether those considered at risk or eligible for the disease have been included.(Webb et al., 2005) For example, if look at the prevalence or incidence of breast cancer, have these studies reported the proportion out of the whole population, all females, only adult females, and so on. These different populations may contribute to clinical heterogeneity.

Methodological heterogeneity is also important to consider. Prevalence and incidence data can arise from various study designs with differing levels of methodological quality. This can also results in differences amongst studies.

But how does one tell whether or not differences are significant?

Firstly, the studies should be assessed carefully to determine whether there is clinical or methodological heterogeneity present. If conducting a meta-analysis, then a visual inspection of the meta-analysis output (e.g. the forest plot) is the first stage of assessing heterogeneity. If the results are scattered across the forest plot and none of the confidence intervals overlap, this is a good indicator of heterogeneity.

A formal statistical test of the similarity of studies is provided by test of homogeneity. The test calculates a probability (P value) from a Chi-square statistic calculated using estimates of the individual study weight, effect size and the overall effect size. Note, however, that this test suffers from lack of power – and will often fail to detect a significant difference when a difference actually exists – especially when there are relatively few studies included in the meta-analysis. Because of this low power, some review authors use a significance level of $P < 0.1$, rather than the conventional 0.05 value, in order to protect against the possibility of falsely stating that there is no heterogeneity present. Often when combining the results from a series of observational studies, this is the default significance level due to the increased heterogeneity associated inherent with the study design.

The I2 statistic is the percentage of observed total variation across due to heterogeneity and not due to chance. A value of 0% indicates no observed heterogeneity and larger values show increasing heterogeneity.

If there is statistically significant heterogeneity, a narrative synthesis or graphical representation is recommended.

Subgroup analysis (Analysis of subgroups or subsets):
Subgroup analysis is a means of investigating heterogeneous results and can be used to estimate the influence of various subsets including age group, gender, type of population and sampling strategy used to gather data (e.g. letter, phone, face-to-face). However, subgroups should be pre-specified a priori and should be few. Subgroup analysis may include by study design or by patient groups.

Meta-regression

Meta-regression investigates whether particular covariates explain any of the heterogeneity of treatment effects between studies. A meta-regression is either a linear or logistic regression and can be fixed-effect or random-effect model. The unit of analysis is a study and predictors in the regression are the study-level covariates.

Publication bias

The research that appears in the published literature may be systematically unrepresentative of the population of completed studies. 'File drawer' problem or 'Publication bias' is a term coined by Rosenthal to mean the number of statistically non-significant studies ($p > 0.05$) that remain unpublished.(Rosenthal & Rubin, 1982) A Funnel plot is used to detect publication bias. This is a scatter plot of effect estimate (x-axis) against inverse of its variance (y-axis). If there is no bias then the funnel will appear symmetric and inverted and if there is bias, the funnel will be asymmetric or skewed in shape.

For peer review only

5.5.9 Results

This section should allow the reader to clearly follow how the included studies were identified and selected for inclusion in the review. In addition, the number of papers excluded should also be stated. There should be a narrative description of the process accompanied by a flowchart of review process (from the PRISMA statement) detailing the flow from the search, through study selection, duplicates, full text retrieval, and any additions from 3rd search, appraisal, extraction and synthesis

Details of full-text articles retrieved for critical appraisal should be given. There should be separate appendices for details of included and excluded studies and for excluded studies; reasons should be stated on why they were excluded.

Description of studies

This section of the results should also include an overall description of the included studies (with reference to the table in the appendices), with the main aim to provide some context to the results section and sufficient detail for the reader to determine if the included studies are similar enough to combine in meta-analysis. Specific items/points of interest from individual studies may also be highlighted here. Additional details may include the assessment of methodological quality, characteristics of the participants and types of interventions and outcomes.

Where a systematic review has several foci, the results should be presented in a logical, structured way, relevant to the specific questions. The roles of tables and appendices should not be overlooked. Adding extensive detail on studies in the results section may crowd the findings, making them less accessible to readers, hence the use of tables and in text reference to specific appendices is encouraged.

Methodological quality

This section should focus on methodological quality as determined by the relevant critical appraisal checklist. There should be a narrative summary of the overall methodological quality of the included studies, which can be supported (optional) by a table showing the results of the critical appraisal (see Table 1 for example). Where only few studies are identified, or there are specific items of interest from included studies, these should be addressed in the narrative also, particularly where studies were deficient, or particularly good. i.e. with clear narrative regarding risk of bias/rigour of included studies. Use of N/A should also be justified in the text.

Table 1. Critical appraisal results for included studies using the JBI-Prevalence Critical Appraisal Checklist

Study	Q1	Q2	Q3	Q4	Q5	Q6	Q7	Q8	Q9	Q10
Author(s) ref	Y	Y	Y	N	Y	U	Y	N	Y	U

Y - Yes, N - No, U - Unclear

Findings of the review

Although there is no defined structure for this section, the findings of the review should flow logically from the review objection/question i.e. they must ultimately answer the question! Findings should be extracted and a narrative, tabular, graphical or meta-analysis should constitute part of this section.

With detail on the studies reported, the results section then focuses on providing a detailed description of the results of the review. For clarity and consistency of presentation, JBI recommends that the reviewer, in discussion with their review panel, give consideration to whether the specific review question used to structure the results section, or whether the findings can be reported under the conditions specified in the protocol. When a systematic review seeks to address multiple questions, the results may be structured in such a way that particular conditions are reported according to the specific questions.

Given there is no clear international standard or agreement on the structure or key components of this section of a review report, and the level of variation evidence in published systematic reviews, the advice here is general in nature. In general, findings are discussed textually and then supported with meta-graphs, tables, figures as appropriate. Graphs may be particularly useful for presenting prevalence and incidence data where meta-analysis is not possible.

The focus should be on presenting information in a clear and concise manner. Any large or complex diagrams/tables/figures should be included as appendices so as not to break the flow of text. Meta-view graphs represent a specific item of analysis that can be incorporated in to the results section of the review. However, the results are more than meta-view graphs, and whether this section is structured based on the intervention of interest, or some other structure, the content of this section needs to present the results with clarity.

Synthesis of Research Findings

For peer review only

It is important to combine the studies in an appropriate manner; otherwise the conclusions that are drawn will not be valid. Where possible study results should be pooled in statistical meta-analysis. Where statistical pooling is not possible the findings can be presented in narrative summary or graphical form, as previously discussed.

This section of the report should describe the data type , the required effects model used (random/fixed), the statistical method of meta-analysis required and the size of confidence limits to be included in the calculations. The method used will depend on the data type.

For peer review only

5.5.10 Discussion

This section should discuss the results of the synthesis as well as any limitations of the primary studies included in the review and of the review itself (i.e. language, access, timeframe, study design, etc.). The results should be discussed in the context of current literature, practice and policy.

The aim of this section is to minimise and discuss the main findings – including the strength of the evidence, for each main outcome. It should address the issues arising from the conduct of the review including limitations and issues arising from the findings of the review (such as search limitations). The discussion does not bring in new literature or information that has not been reported in the results section. The discussion does seek to establish a line of argument based on the findings regarding the effectiveness of an intervention, or its impact on the outcomes identified in the protocol. The application and relevance of the findings to relevant stakeholders (e.g. healthcare providers, patients and policy makers) should also be discussed in this section.

Points to consider this section include:

- Where any problems identified undertaking the search (perhaps there is little primary research on this topic or perhaps it is poorly indexed by the databases that were searched or perhaps the search was insufficient)?
- What limitations were found in the included primary research (e.g. were there inconsistencies or errors in reporting)?
- How do the review findings fit with what is currently known on the topic (from issues highlighted in the Background section)?
- Are the findings generalizable to other populations of participants/healthcare settings etc?

For peer review only

5.5.11 Conclusions

This section should begin with an overall conclusion based on the results. The conclusions drawn should match with the review objective/question.

Conclusion

The conclusion section of a systematic review should provide a general interpretation of the findings in the context of other evidence and provide a detailed discussion of issues arising from the findings of the review and demonstrate the significance of the review findings to practice and research. Ares that may be addressed include:

- A summary of the major findings of the review;
- Issues related to the quality of the research within the area of interest;
- Other issues of relevance; and
- Potential limitations of the systematic review.

Recommendations for practice

It should be stated how the findings of the review impact on clinical practice in the area. If there is sufficient evidence to make specific recommendations for practice, then the appropriate JBI Grades of Recommendation should be assigned to each recommendation based on the study design that led to the recommendation.

Recommendations for research

This section should include clear, specific recommendations for future research based on gaps in knowledge identified from the results of the review. Implications for research should avoid generalised statements calling for further research, but should be linked to specific issues.

For peer review only

5.6 Chapter references

Akl, E. A., Guyatt, G. H., Irani, J., Feldstein, D., Wasi, P., Shaw, E., . . . Schunemann, H. J. (2012). "Might" or "suggest"? No wording approach was clearly superior in conveying the strength of recommendation. *J Clin Epidemiol, 65*(3), 268-275. doi: 10.1016/j.jclinepi.2011.08.001

Andrews, J., Guyatt, G., Oxman, A. D., Alderson, P., Dahm, P., Falck-Ytter, Y., . . . Schunemann, H. J. (2013). GRADE guidelines: 14. Going from evidence to recommendations: the significance and presentation of recommendations. *J Clin Epidemiol, 66*(7), 719-725. doi: 10.1016/j.jclinepi.2012.03.013

Australian Bureau of Statistics. (2012). 4102.0 - Australian Social Trends, Jun 2012 Retrieved 13/11/13, from http://www.abs.gov.au/AUSSTATS/abs@.nsf/Lookup/4102.0Main+Features10Jun+2012#Viral

Borenstein M, Hedges LV, Higgins JPT, & Rothstein HR. (2009). *Introduction to meta-analysis* Chichester : John Wiley & Sons.

Centers for Disease Control. (1981). Kaposi's sarcoma and Pneumocystis pneumonia among homosexual men--New York City and California. *MMWR Morb Mortal Wkly Rep, 30*(25), 305-308.

Daniel, W. W. (1999). *Biostatistics: A Foundation for Analysis in the Health Sciences.*

edition (7th ed.). New York: John Wiley & Sons. .

Deeks, J. J., & Altman, D. G. (2001). Effect measures for meta-analysis of trials with binary outcomes. In M. Egger, G. D. Smith & D. G. Altman (Eds.), *Systematic Reviews in Health Care: Meta-Analysis in Context* (2nd ed., pp. 313-335). London: BMJ Publishing Group.

Field, A. (2001). Meta-analysis of correlation coefficients: a Monte Carlo comparison of fixed- and random-effects methods. *Psychol Methods, 6*(2), 161-180.

Fleiss, J. L. (1993). The statistical basis of meta-analysis. *Stat Methods Med Res, 2*, 121-145.

Guyatt, G., Drummond, R., Meade, M. O., & Cook, D. J. (2008). *Users' guides to the medical literature: a manual for evidence-based clinical practice* (2nd ed.). USA: McGraw Hill Companies: JAMA & Archives Journals.

Guyatt, G. H., Oxman, A. D., Kunz, R., Falck-Ytter, Y., Vist, G. E., Liberati, A., . . . Group, G. W. (2008). Going from evidence to recommendations. *BMJ, 336*(7652), 1049-1051. doi: 10.1136/bmj.39493.646875.AE

Hicks, N. (1997). Evidence based healthcare. *Bandolier, 4*(39), 8.

Higgins, J. P., & Thompson, S. G. (2002). Quantifying heterogeneity in a meta-analysis. *Stat Med, 21*(11), 1539-1558. doi: 10.1002/sim.1186

Hillier, S., Grimmer-Somers, K., Merlin, T., Middleton, P., Salisbury, J., Tooher, R., & Weston, A. (2011). FORM: an Australian method for formulating and grading recommendations in evidence-based clinical guidelines. *BMC Med Res Methodol, 11*, 23. doi: 10.1186/1471-2288-11-23

Kock, A. A. (2009). Guideline to Meta-Analysis: TIM Working Paper Series. *Technische Universität Berlin, 2*.

Last, J. (1995). *A dictionary of epidemiology*: Oxford University Press.

Lockwood, C., & White, S. (2012). *Synthesizing descriptive evidence*. Philadelphia: Lippincott Williams & Wilkins.

Lomotan, E. A., Michel, G., Lin, Z., & Shiffman, R. N. (2010). How "should" we write guideline recommendations? Interpretation of deontic terminology in clinical practice guidelines: survey of the health services community. *Qual Saf Health Care, 19*(6), 509-513. doi: 10.1136/qshc.2009.032565

Michie, S., & Johnston, M. (2004). Changing clinical behaviour by making guidelines specific. *BMJ, 328*(7435), 343-345. doi: 10.1136/bmj.328.7435.343

Michie, S., & Lester, K. (2005). Words matter: increasing the implementation of clinical guidelines. *Qual Saf Health Care, 14*(5), 367-370. doi: 10.1136/qshc.2005.014100

Miller, M. R., Robinson, K. A., Lubomski, L. H., Rinke, M. L., & Pronovost, P. J. (2007). Medication errors in paediatric care: a systematic review of epidemiology and an evaluation of evidence supporting reduction strategy recommendations. *Qual Saf Health Care, 16*(2), 116-126. doi: 10.1136/qshc.2006.019950

Mills, E. J., Nachega, J. B., Bangsberg, D. R., Singh, S., Rachlis, B., Wu, P., . . . Cooper, C. (2006). Adherence to HAART: a systematic review of developed and developing nation patient-reported barriers and facilitators. *PLoS Med, 3*(11), e438. doi: 10.1371/journal.pmed.0030438

Munn, Z., Moola, S., Lisy, K., Riitano, D., & Murphy, F. (2014). Claustrophobia in Magnetic Resonance Imaging: A Systematic Review and Meta-Analysis *Unpublished data - Submitted for publication*.

For peer review only

Munn, Z., Tufanaru, C., & Aromataris, E. (2014). Data extraction and synthesis in systematic reviews. *American Journal of Nursing*, In Press.

Naing, L., Winn, T., & Rusli, B. N. (2006). Practical issues in calculating the sample size for prevalence studies *Archives of Orofacial Sciences, 1*, 9-14.

Pearson, A., Robertson-Malt, S., & Rittenmeyer, L. *Synthesizing Qualitative Evidence*. Philadelphia Lippincott Williams & Wilkins

Pearson, A., Wiechula, R., Court, A., & Lockwood, C. (2005). The JBI model of evidence-based healthcare. *Int J Evid Based Healthc, 3*(8), 207-215. doi: 10.1111/j.1479-6988.2005.00026.x

Petitti, D. B. (2000). *Meta-analysis, decision analysis, and cost-effectiveness analysis : methods for quantitative synthesis in medicine* (2nd ed.). New York: Oxford University Press,.

Rao, S., Graubard, B., Schmid, C., Morton, S., Louis, T., Zaslavsky, A., & Finkelstein, D. (2008). Meta-analysis of survey data: application to health services research. *Health Services and Outcomes Research Methodology, 8*(2), 98-114. doi: 10.1007/s10742-008-0032-0

Rosenthal, R., & Rubin, D. (1982). Comparing effect sizes of independent studies. *Psychological Bulletin, 92*(500-504).

Sackett, D. L., Rosenberg, W. M., Gray, J. A., Haynes, R. B., & Richardson, W. S. (1996). Evidence based medicine: what it is and what it isn't. *BMJ, 312*(7023), 71-72.

Saha, S., Chant, D., & McGrath, J. (2008). Meta-analyses of the incidence and prevalence of schizophrenia: conceptual and methodological issues. *Int J Methods Psychiatr Res, 17*(1), 55-61. doi: 10.1002/mpr.240

Straus, S. E., Richardson, W. S., Glasziou, P., & Haynes, R. B. (2005). *Evidence-Based Medicine: How to Practice and Teach EBM* (3rd ed.): Elsevier Churchill Livingstone.

Sutton, A. J., Abrams, K. R., Jonas, M., Sheldon, T. A., & Song, F. (2000). *Methods for meta-analysis in medical research*. Chichester, New York: Wiley.

The Joanna Briggs Institute. (2008). *The Joanna Briggs Institute Reviewers' Manual 2008 Edition*. Adelaide: The Joanna Briggs Institute.

The Kirby Institute. (2012). HIV, viral hepatitis and sexually transmissible infections in Australia, Annual Surveillance Report 2012 (Vol. 2052). Sydney NSW The Kirby Institute, the University of New South Wales.

University of New Mexico. (2007). Gray literature and how to find it, from http://hsc.unm.edu/library/subject/graylit.shtml

Webb, P., Bain, C., & Pirozzo, S. (2005). *Essential epidemiology: an introduction for students and health professionals*. New York: Cambridge University Press.

Woolf, S., Schunemann, H. J., Eccles, M. P., Grimshaw, J. M., & Shekelle, P. (2012). Developing clinical practice guidelines: types of evidence and outcomes; values and economics, synthesis, grading, and presentation and deriving recommendations. *Implement Sci, 7*, 61. doi: 10.1186/1748-5908-7-61

For peer review only

Appendix 5.1: Critical Appraisal Instrument for Studies Reporting Prevalence Data

JBI Critical Appraisal Checklist for Studies Reporting Prevalence Data

Reviewer____Date__________________________
____

Author ____Year___________Record Number ________

	Yes	No	Unclear	Not applicable
1. Was the sample frame appropriate to address the target population?	?	?	?	?
2. Were study participants sampled in an appropriate way?	?	?	?	?
3. Was the sample size adequate?	?	?	?	?
4. Were the study subjects and the setting described in detail?	?	?	?	?
5. Was the data analysis conducted with sufficient coverage of the identified sample?	?	?	?	?
6. Were valid methods used for the identification of the condition?	?	?	?	?
7. Was the condition measured in a standard, reliable way for all participants?	?	?	?	?
8. Was there appropriate statistical analysis?	?	?	?	?
9. Was the response rate adequate, and if not, was the low response rate managed appropriately?	?	?	?	?

Overall appraisal: Include ? Exclude ? Seek further info ?

Comments (Including reason for exclusion)

© JBI 2021

Explanation of Prevalence Critical Appraisal

How to cite: Munn Z, Moola S, Lisy K, Riitano D, Tufanaru C. (2015) Methodological guidance for systematic reviews of observational epidemiological studies reporting prevalence and incidence data. Int J Evid Based Healthc. 2015; 13:147–153.

Prevalence Critical Appraisal Tool

Answers: Yes, No, Unclear or Not/Applicable

1. Was the sample frame appropriate to address the target population?

This question relies upon knowledge of the broader characteristics of the population of interest and the geographical area. If the study is of women with breast cancer, knowledge of at least the characteristics, demographics and medical history is needed. The term "target population" should not be taken to infer every individual from everywhere or with similar disease or exposure characteristics. Instead, give consideration to specific population characteristics in the study, including age range, gender, morbidities, medications, and other potentially influential factors. For example, a sample frame may not be appropriate to address the target population if a certain group has been used (such as those working for one organisation, or one profession) and the results then inferred to the target population (i.e. working adults). A sample frame may be appropriate when it includes almost all the members of the target population (i.e. a census, or a complete list of participants or complete registry data).

2. Were study participants recruited in an appropriate way?

Studies may report random sampling from a population, and the methods section should report how sampling was performed. Random probabilistic sampling from a defined subset of the population (sample frame) should be employed in most cases, however, random probabilistic sampling is not needed when everyone in the sampling frame will be included/ analysed. For example, reporting on all the data from a good census is appropriate as a good census will identify everybody. When using cluster sampling, such as a random sample of villages within a region, the methods need to be clearly stated as the precision of the final prevalence estimate incorporates the clustering effect. Convenience samples, such as a street survey or interviewing lots of people at a public gatherings are not considered to provide a representative sample of the base population.

For peer review only

3. Was the sample size adequate?

The larger the sample, the narrower will be the confidence interval around the prevalence estimate, making the results more precise. An adequate sample size is important to ensure good precision of the final estimate. Ideally we are looking for evidence that the authors conducted a sample size calculation to determine an adequate sample size. This will estimate how many subjects are needed to produce a reliable estimate of the measure(s) of interest. For conditions with a low prevalence, a larger sample size is needed. Also consider sample sizes for subgroup (or characteristics) analyses, and whether these are appropriate. Sometimes, the study will be large enough (as in large national surveys) whereby a sample size calculation is not required. In these cases, sample size can be considered adequate.

When there is no sample size calculation and it is not a large national survey, the reviewers may consider conducting their own sample size analysis using the following formula: (Naing et al. 2006, Daniel 1999)

n= Z2P(1-P)

d2

Where:

n= sample size

Z = Z statistic for a level of confidence

P = Expected prevalence or proportion (in proportion of one; if 20%, P = 0.2)

d = precision (in proportion of one; if 5%, d=0.05)

Ref:

Naing L, Winn T, Rusli BN. Practical issues in calculating the sample size for prevalence studies Archives of Orofacial Sciences. 2006;1:9-14.

Daniel WW. Biostatistics: A Foundation for Analysis in the Health Sciences.

Edition. 7th ed. New York: John Wiley & Sons. 1999.

4. Were the study subjects and setting described in detail?

Certain diseases or conditions vary in prevalence across different geographic regions and populations (e. g. Women vs. Men, sociodemographic variables between countries). The study sample should be described in sufficient detail so that other researchers can determine if it is comparable to the population of interest to them.

5. Was data analysis conducted with sufficient coverage of the identified sample?

Coverage bias can occur when not all subgroups of the identified sample respond at the same rate. For instance, you may have a very high response rate overall for your study, but the response rate for a certain subgroup (i.e. older adults) may be quite low.

6. Were valid methods used for the identification of the condition?

Here we are looking for measurement or classification bias. Many health problems are not easily diagnosed or defined and some measures may not be capable of including or excluding appropriate levels or stages of the health problem. If the outcomes were assessed based on existing definitions or diagnostic criteria, then the answer to this question is likely to be yes. If the outcomes were assessed using observer reported, or self-reported scales, the risk of over- or under-reporting is increased, and objectivity is compromised. Importantly, determine if the measurement tools used were validated instruments as this has a significant impact on outcome assessment validity.

7. Was the condition measured in a standard, reliable way for all participants?

Considerable judgment is required to determine the presence of some health outcomes. Having established the validity of the outcome measurement instrument (see item 6 of this scale), it is important to establish how the measurement was conducted. Were those involved in collecting data trained or educated in the use of the instrument/s? If there was more than one data collector, were they similar in terms of level of education, clinical or research experience, or level of responsibility in the piece of research being appraised? When there was more than one observer or collector, was there comparison of results from across the observers? Was the condition measured in the same way for all participants?

8. Was there appropriate statistical analysis?

For peer review only

Importantly, the numerator and denominator should be clearly reported, and percentages should be given with confidence intervals. The methods section should be detailed enough for reviewers to identify the analytical technique used and how specific variables were measured. Additionally, it is also important to assess the appropriateness of the analytical strategy in terms of the assumptions associated with the approach as differing methods of analysis are based on differing assumptions about the data and how it will respond.

9. Was the response rate adequate, and if not, was the low response rate managed appropriately?

A large number of dropouts, refusals or "not founds" amongst selected subjects may diminish a study's validity, as can a low response rates for survey studies. The authors should clearly discuss the response rate and any reasons for non-response and compare persons in the study to those not in the study, particularly with regards to their socio-demographic characteristics. If reasons for non-response appear to be unrelated to the outcome measured and the characteristics of non-responders are comparable to those who do respond in the study (addressed in question 5, coverage bias), the researchers may be able to justify a more modest response rate.

For peer review only

Appendix 5.2: Data extraction form for prevalence studies

Data extraction form for prevalence studies

Citation Details

Authors:

Title:

Journal:

Year:

Issue:

Volume:

Pages:

Generic Study details

Study design:

Country:

Setting/Context:

Year/ timeframe for data collection:

Participant Characteristics (study inclusion/exclusion information):

Condition and measurement method:

Description of main results (n/N):

For peer review only

Chapter 6: Systematic reviews of economic evidence

This chapter is currently being updated. In the meantime, please refer to the following pdf: https://drive.google.com/file/d/12OkTDHvZ9mGxGHKfsaIIOT563Bffom-t/view?usp=sharing

https://doi.org/10.46658/JBIMES-20-07

For peer review only

Chapter 7: Systematic reviews of etiology and risk

Sandeep Moola, Zachary Munn, Catalin Tufanaru, Edoardo Aromataris, Kim Sears, Raluca Sfetc, Marian Currie, Karolina Lisy, Rubab Qureshi, Patrick Mattis, Pei-Fan Mu

How to cite:

Moola S, Munn Z, Tufanaru C, Aromataris E, Sears K, Sfetcu R, Currie M, Lisy K, Qureshi R, Mattis P, Mu P. Chapter 7: Systematic reviews of etiology and risk. In: Aromataris E, Munn Z (Editors)*. JBI Manual for Evidence Synthesis.* JBI, 2020. Available from https://synthesismanual.jbi.global. https://doi.org/10.46658/JBIMES-20-08

Chapter 7: Contents

- 7.1 Introduction to etiological evidence and systematic reviews
- 7.2 Study designs for etiology and risk
  - 7.2.1 Observational Study Designs
    - 7.2.1.1 Cohort Studies
    - 7.2.1.2 Case-control studies
    - 7.2.1.3 Cross-sectional studies (Analytical)
  - 7.2.2. Descriptive study designs
- 7.3 The systematic review protocol and report
  - 7.3.1 Title of the systematic review
  - 7.3.2 Abstract
  - 7.3.3 Objective and review question
  - 7.3.4 Background
  - 7.3.5 Inclusion criteria
    - 7.3.5.1 Population (types of participants)
    - 7.3.5.2 Exposure of interest (Independent variable)
    - 7.3.5.3 Outcome (dependent variable)
    - 7.3.5.4 Types of studies
  - 7.3.6 Methods
    - 7.3.6.1 Search strategy
    - 7.3.6.2 Sources to search
    - 7.3.6.3 Assessment of methodological quality
      - 7.3.6.3.1 Confounding and confounders
      - 7.3.6.3.2 Types of bias in studies of etiology and risk
    - 7.3.6.4 Data extraction
    - 7.3.6.5 Data synthesis
      - 7.3.6.5.1 Meta-analysis of observational research
      - 7.3.6.5.2 The narrative synthesis of data
      - 7.3.6.5.3 The tabular synthesis of data
  - 7.3.7 Results
    - 7.3.7.1 Description of studies
    - 7.3.7.2 Methodological quality
    - 7.3.7.3 Findings of the review
  - 7.3.8 Discussion
  - 7.3.9 Conclusion and Recommendations
  - 7.3.10 Appendices
- 7.4 Chapter references
- Appendix 7.1 Critical appraisal checklist for cohort studies
- Appendix 7.2 Critical appraisal checklist for case-control studies
- Appendix 7.3 Critical appraisal checklists for case series
- Appendix 7.4 Critical appraisal checklist for case reports
- Appendix 7.5 Critical appraisal checklist for analytical cross-sectional studies

For peer review only

7.1 Introduction to etiological evidence and systematic reviews

In the epidemiological literature, terms such as risk, risk factors, and cause are inconsistently and imprecisely used, and as a result are often misinterpreted leading to incorrect research and policy recommendations.(Kraemer, Kazdin et al. 1997) Risk refers to the probability of an outcome within a population of subjects (e.g. risk of lung cancer among people exposed to asbestos)(Kraemer, Kazdin et al. 1997) and etiology refers to the cause or the causes (origin) of a certain disease. It is important to distinguish between etiology and risk factors. A risk factor refers to an individual characteristic or exposure that is associated with an increased likelihood of an outcome occurring. For example, are children in sub-Saharan Africa who are exposed to Plasmodium falciparum malaria at an increased risk of developing mental disorders?(Akpalu, Ae-Ngibise et al. 2012) Whereas a protective factor refers to a characteristic or exposure that is associated with the reduced likelihood of an adverse outcome. For example, are people who perform regular higher levels of physical activity less likely to develop lung cancer than those who perform little or no physical activity?(Cancer Australia 2014)

Risk factors are commonly referred to as modifiable, which means they may be controlled or modified in some way, or they may represent a characteristic over which an individual has no control, and therefore categorized as non-modifiable. Exposure to cigarette smoke (either actively or passively), elevated arsenic concentrations, or asbestos in the work or home environment are examples of exposure to modifiable factors – all can ultimately be avoided in most circumstances. Conversely, having a family history of the disease is also known to increase the likelihood of lung cancer development in an individual,(Cancer Australia 2014) and despite any efforts, these non-modifiable risk factors, though less common, are difficult to control or modify.

Systematic reviews of etiology and risk factors assess the relationship (association) between certain factors (whether genetic or environmental for example) and the development of a disease or condition or other health outcome. Systematic reviews underpin evidence-based healthcare. The process of conducting a systematic review is a scientific exercise, and as the results will influence health care decisions, it is required to have the same rigor expected of all research. The quality of a systematic review depends on the extent to which the methods minimize the risk of error and bias. There is currently no universally accepted methodology for conducting systematic reviews of etiology and risk. Systematic review and meta-analysis of studies related to etiology and risk can provide useful information for healthcare professionals and policymakers on the risk factors (and preventive or protective factors) of disease and where factors, other than direct intervention with therapy and treatment, may influence or impact on health outcomes. Systematic review of etiological studies is important in the public health domain for informing health care planning, resource allocation and strategies for disease prevention.

This chapter outlines and describes JBI's approach and guidance for synthesizing evidence related to etiology and risk and contributes to the emerging field of systematic review methodologies. The systematic review of studies to answer questions of etiology and risk still adheres to the same basic principles of systematic review of other types of data. An *a priori* protocol must precede and inform the conduct of the systematic review, comprehensive searching must be performed, and critical appraisal of retrieved studies must be carried out followed by data abstraction, analysis and synthesis. These steps will be further discussed in the following sections of this chapter. Additionally, reviewers should refer to two statements/checklists: one for transparent reporting of a systematic review of various research study designs (preferred reporting items for systematic reviews and meta-analyses (PRISMA))(Moher, Liberati et al. 2009) and one for Meta-Analyses Of Observational Studies in Epidemiology (MOOSE), which provides a checklist or guidance to report meta-analyses of observational studies in epidemiology, including background, search strategy, methods, results, discussion, and conclusion.(Stroup, Berlin et al. 2000)

A note on causation

British epidemiologist Sir Austin Bradford Hill proposed in 1965 a list of nine “viewpoints”, or “circumstances” or “aspects” that should be considered when exploring the likelihood of inferring causation from examined associations: strength of the association; consistency of the observed association; specificity of the association; temporal relationship of the association; biological gradient (dose-response); biological plausibility; coherence (cause-effect interpretation of data should not conflict with generally known facts regarding natural history and biology of the disease; experimental evidence; analogy).(Hill 1965) Sir Bradford Hill explicitly stated that none of the nine viewpoints can be used as “indisputable evidence” for or against the causal hypothesis and that these aspects are used to explore more or less likely alternative explanations to the proposed causal explanation for the observed association.[16]

A comprehensive modern discussion about causality (including a critical examination of Hill’s viewpoints) was provided by Rothman et al (2008).(Rothman, Greenland et al. 2008) It was contended that temporality is a *sine qua non* for causal explanations of observed associations; however, there is no other criterion other than temporality that is necessary or sufficient criterion for determining whether an observed association is causal.(Rothman, Greenland et al. 2008)

For peer review only

7.2 Study designs for etiology and risk

Commonly, epidemiological or observational studies are utilized to investigate etiology and risk. Observational studies aid in studying causal associations between an exposure and disease/health outcome (for example associations between occupational risk factors and lung cancer, or the adverse effects of a treatment in healthcare), although distinguishing true causality generally requires experimental research. Observational studies do not involve manipulation on the part of the researcher. These studies rely on the natural or 'ecological' events of exposures and disease, where the researcher simply observes certain characteristics of the sample population as they occur "naturally", and records the relevant data.(The Joanna Briggs Institute b 2014) In this way they can be distinguished from experimental or quasi-experimental studies (such as RCTs and controlled clinical trials) where there is researcher manipulation of the independent variable (the potential cause or the exposure).(The Joanna Briggs Institute b 2014)

For peer review only

7.2.1 Observational Study Designs

Observational study designs include prospective and retrospective cohort studies, case-control studies, cross-sectional studies, case series and case reports, and can be broken down into the broad categories of analytical studies and descriptive studies. Generally, descriptive studies describe the occurrence /presence of an outcome or exposure, whereas analytical studies describe the relationship between the exposure and an outcome. Due to the nature of observational study designs compared with experimental designs, they are more at risk of the influence of confounding factors and different sources of bias that are unavoidable, which will be discussed further below. Similar to the MOOSE statement,(Stroup, Berlin et al. 2000) reviewers should also refer to the Strengthening the reporting of observational studies in epidemiology (STROBE) statement, which is a checklist of items that need to be addressed in studies reporting on cohort, case-control, and cross sectional study designs and provides guidance on how to report observational research.(von Elm, Altman et al. 2007)

For peer review only

7.2.1.1 Cohort Studies

Cohort studies are the 'gold standard' of observational study designs and prospective cohort studies appear the highest on evidence hierarchies of observational study designs.(Thiese 2014) These longitudinal studies are typically used to analyse relationships between exposures and disease by comparing the outcomes between two groups over time, where individuals in one group are exposed to a common event or characteristic, such as a risk factor, and the other group are not. Sampling in cohort studies is based on the presence or absence of an exposure or characteristic, and participants are followed over time to observe development of any disease or health outcomes. A prospective cohort study begins with the exposure of interest, and participants are followed forward through time to observe any outcomes that may occur. Conversely, a retrospective cohort study generally begins after the outcomes of interest have already been recorded; a researcher may sift through patient records or data that is already available and groups patients according to exposures, and identifies any differences in outcomes. Cohort studies enable observations of a large number of people over a long period of time.

For peer review only

7.2.1.2 Case-control studies

Case-control studies select participants based on presence of disease or a specific condition, and look for prior exposures that may have led to the disease or outcome developing. In this study design, those with the disease/outcome (cases) are matched with comparable individuals who do not have the disease (controls), and both groups are studied to determine if any differences in characteristics or past exposures exist. Case control studies have an advantage over cohort studies, particularly when investigating rare diseases, because of fewer costs associated with recruiting participants (usually less). In addition, the issue of ‘drop out’ or ‘loss to follow up’ of participants as seen in cohort studies does not arise in case-control studies.

For peer review only

7.2.1.3 Cross-sectional studies (Analytical)

Cross-sectional studies are used to provide a snapshot of disease and other variables in a defined population at one point in time. Data can be used to infer relationships between a disease and other variables, however as the data is gathered simultaneously, chronological sequences of exposures and outcomes cannot be determined. Some cross-sectional studies are purely descriptive, in that they just describe the number of cases or number of events in a particular population at a point in time or over a period of time.

For peer review only

7.2.2. Descriptive study designs

Descriptive studies aim to collect information about a given individual or group and can be used to provide data on the distribution of disease. Examples of descriptive study designs are case reports and case series. In health care, these types of studies are typically used to describe the occurrence of disease or a risk factor. Case reports and case series are often used to report novel occurrences of a disease or a unique finding, and they can be particularly informative for rare or emerging diseases. There are guidelines to report case reports in terms of completeness, transparency and data analysis (The CARE Guidelines: Consensus-based Clinical Case Reporting Guideline Development),(Gagnier, Kienle et al. 2014) which the reviewers should refer to when including and reporting case reports in their systematic review reports.

For peer review only

7.3 The systematic review protocol and report

This section outlines the requirements and methods for systematic review protocols and systematic review reports addressing etiology and risk.

For peer review only

7.3.1 Title of the systematic review

The title should be clear, explicit and reflect the core elements of the question. It should be as informative and descriptive as is reasonable reflecting the scope and type of systematic review to be undertaken. The title should not be phrased as a question or conclusion and there should be congruency between the title, review objectives/questions and inclusion criteria. The title should include the phrase “A systematic review protocol” in a review protocol and “A systematic review” in a review report.

Although a range of mnemonics have been described for different types of review (and research) questions, if, for example the review aims to examine etiology of disease or risk of a health outcome, this should, as much as possible, be stated clearly in the title of the document. If specific exposure/s and/or patient outcomes are to be examined these should also be included in the title. For example: “Long-term topical corticosteroid use and risk of skin cancer: a systematic review protocol”.(Ratib, Burden-Teh et al. 2016) This example provides potential readers of the review with a clear indication of the population, the exposure (corticosteroid use), and the outcome (incidence of skin cancer) of interest, as well as that it is a systematic review protocol.

For peer review only

7.3.2 Abstract

This section forms a structured abstract of the main features of the systematic review. It must be no longer than 500 words and should contain no abbreviations or references. The abstract must accurately reflect and summarize the systematic review with the main focus on the results of the review.

The abstract should report the essential elements of the review using the following sub-headings in this order:

- **Objective**: State an overarching review objective structured using the key components of the inclusion criteria (approximately one to two sentences).
- **Background**: Briefly describe what is already known on the topic and what this review will add to the evidence-base (approximately two to three sentences).
- **Inclusion criteria:** Summarize the inclusion criteria as it relates to the type of review being conducted. Present the information in one or two sentences – **NOT** under individual subheadings.
- **Methods:** List the key information sources searched (those that provided the majority of included studies), any limits placed on the scope of the search (e.g. language), and the date range, or the date of the last search. If the recommended JBI approach to critical appraisal, study selection, data extraction and data synthesis was used, simply state it as such (without naming the actual tool). Otherwise, briefly describe any notable deviations to the methodological approach taken (e.g. criteria used to exclude studies on the basis of methodological quality etc.).
- **Results**: The bulk of the abstract should be reserved to convey the main results of the review. As a general rule, report the number and type of included studies and participants, as well as any pertinent study characteristics. Summarize the overall quality of the included studies and notable aspects of rigor for qualitative reviews).
  Report the number of findings and categories and final synthesized findings. Depending how many are presented in the review, the synthesized findings may be presented here or abridged summarized statements.
- **Conclusions**: Provide a conclusion based on a general interpretation of the results considering, for example, the methodological quality of the included studies and any limitations of the review. Briefly convey key implications for practice and/or research.

For peer review only

7.3.3 Objective and review question

The objective(s) of the review should be clearly stated. This should be followed by the specific review question(s). The overarching objectives of reviews of etiology and risk are to determine whether and to what degree a relationship exists between two or more quantifiable variables. Accordingly, the review question should outline the exposure, the population or groups at risk and the disease, symptom or health outcome of interest. The specific context/location (which may include any contextual factors such as geographical, or cultural elements relevant to the topic), and the duration of the exposure (e.g. pregnancy) may also be important to articulate if relevant.

An example of an objective for a systematic review of etiology and risk is:

- The objective of this review is to assess the epidemiological association between consumption of alcohol (as exposure of interest or risk factor) and lung cancer (as the outcome of interest).

A question that will align with this review objective is:

- Does the consumption of alcohol increase the incidence of lung cancer?

The exposure and outcome may be positively associated or the relationship may be negative e.g. as one increases the other decreases.

For peer review only

7.3.4 Background

The background section of the review protocol and systematic review should be comprehensive and consider the main elements of the topic under review. Many reviewers will find that the background provided with the protocol needs modification or extension following the conduct of the review proper. The background should detail any definitions important to the review. The information in the background section must be sufficient to put the review inclusion criteria into context and also highlight the importance and relevance of the topic for the reader and a clear basis for the rationale to pursue the review topic. The background section should conclude with a statement that a preliminary search for previous systematic reviews on the topic was conducted (state the sources searched e.g. *JBI Evidence Synthesis*, Cochrane Library, CINAHL, PubMed, PROSPERO). If there is a previous systematic review on the topic, it should be specified how the proposed review differs. All JBI systematic reviews should contain a sentence clearly stating:

"The objectives, inclusion criteria and methods of analysis for this review were specified in advance and documented in an a priori protocol. Ref" (Reference should be to the appropriate citation in the *JBI Evidence Synthesis*, and provide registration number in PROSPERO where applicable).

This sentence should appear as the final line of the background/introduction section of the review report and complies with the recommendations for reporting of systematic reviews detailed in the PRISMA guidelines.

For peer review only

7.3.5 Inclusion criteria

Specific inclusion criteria ensure that the included studies will meet these criteria and they represent an important and transparent plan for to the selection of studies for the review. The inclusion criteria are also critical when formulating a comprehensive search strategy to locate studies.

Authors will realize that the traditional PICO format (Population, Intervention, Comparator, Outcomes) commonly encountered and well aligned to systematic reviews(The Joanna Briggs Institute a 2014) assessing the effectiveness of interventions or therapies in health care does not readily align with questions relating to etiology and risk. Rather, a systematic review of etiology should include the following components, easily referred to as PEO:

- Population (types of participants)
- Exposure of interest (independent variable)
- Outcome (dependent variable)

For peer review only

7.3.5.1 Population (types of participants)

The types of participants should be appropriate for the review objective and question(s). The reasons for the inclusion of a participant group should be supported by information in the background and the rational for the review. Specific criteria for inclusion or exclusion of participants should be explained in this section. The inclusion and exclusion criteria need to reflect sound clinical and scientific reasoning and the need for an adequate degree of homogeneity amongst the samples in the studies.

For peer review only

7.3.5.2 Exposure of interest (Independent variable)

This refers to a particular risk factor or several risk factors (or protective factors) of interest. It should be clearly reported in this section what the exposure or risk factor (or protective factor) is, and how it may be measured/identified including the nature of the exposure and its intensity and/or the duration of exposure, if relevant. The exposure of interest may be modifiable, and relate to lifestyle habits such as alcohol consumption, smoking or may relate to the environment and occupation such as asbestos and air pollution or conversely, may be non-modifiable, such as family history of the disease.

For peer review only

7.3.5.3 Outcome (dependent variable)

It should be clearly reported in this section what the outcome (disease or condition) is, and how it may be measured/identified. Commonly, the outcome of reviews of etiology and risk is often the incidence or observed rate of a disease or condition. Outcomes should be presented in a non-directional expression; for example, the outcome should simply be stated as the incidence of lung cancer, not an increase in lung cancer, as the evidence may suggest that the exposure has no effect and does not increase risk (neutral factor) or may decrease the risk (protective factors). The review protocol should specify the important outcomes of interest relevant to the health issue and relevant to key stakeholders like the knowledge users, consumers, policy makers, consumers and the like.

For peer review only

7.3.5.4 Types of studies

Epidemiological observational studies of etiology relate individual characteristics, personal behaviours, environmental conditions, and treatments as ‘exposures’ that may modify risk of disease. These reviews will predominantly include observational studies such as prospective and retrospective cohort studies, case control studies and analytical cross-sectional studies. Randomized controlled trials may also report on the risk associated with an exposure and can be included. Prospective cohort studies usually provide stronger evidence than case-control studies when addressing etiological questions or issues.

For peer review only

7.3.6 Methods

This section of the review report is reserved for the methods used to conduct the review and should be presented under the relevant subheadings, including any deviations from the method outlined in the *a priori* protocol. In empty reviews for example, this section should not refer to methods that were not performed.

Directly below the Methods heading provide the following information:

- State and appropriately cite the JBI methodology that was employed in the conduct of the review and synthesis.
- Refer to and cite the *a priori* protocol that was published, or accepted for publication (e.g. 'in press'), in the *JBI Evidence Synthesis*.
- If the protocol has been registered with PROSPERO, provide registration information including registration number (e.g. PROSPERO CRD42015425226).

For peer review only

7.3.6.1 Search strategy

This section should state how the reviewers plan to search for relevant papers in a protocol and how they conducted the final search in a review report, clearly detailing how the review authors located the studies included in their review. Details of the databases and sources searched must be provided along with search strategies and the search dates. Databases and sources searched should be appropriate for the review question and include specification from the outset of the platform used to search a particular database. A JBI review should search for studies published by commercial and academic publishers as well as non-commercially published studies (grey literature). An example of a source of grey literature is Open Grey. Any limits applied to the search, for example limiting the range of years searched, should be justified and any language restrictions stated (e.g. only studies published in English will be considered for inclusion).

In the JBI review report, a detailed search strategy for all of the major databases searched should be appended and relevant details and dates of searching through other sources. The documentation of search strategies is a key element of the scientific validity of a systematic review. It enables readers to look at and evaluate the steps taken, decisions made to consider the comprehensiveness and exhaustiveness of the search strategy for each included database.

For peer review only

7.3.6.2 Sources to search

Appropriate databases to search should be included, the most common being Medline (PubMed) and EMBASE. Details should include specification from the outset of the platform used to search a particular database. Etiology and risk data are commonly reported within the published, peer-reviewed literature and accordingly the standard JBI three-step search strategy can be applied to locating this type of evidence. The search strategy should use both subject heading and text word searches. Initial search terms should be updated after searching the reference lists of relevant articles. The timeframe chosen for the search should be justified and any language restrictions stated (e.g. only studies published in English will be considered for inclusion).

A JBI review should consider papers both published and unpublished (grey) literature. Grey literature can often provide useful studies and estimates for reviews of etiology and risk factors.

Some examples include:

- Disease and health association websites. (e.g. American Diabetes Association)
- Bibliographic databases: Disease and allied health research database (e.g. Medline, EMBASE, PsycINFO, CINAHL, British Nursing Index (BNI), Web of Science, and Cochrane library. PhD theses etc.)
- Conference abstracts or proceedings (e.g. BIOSIS databases, American Society of Clinical Oncology (ASCO), Biological Abstracts/RRM, British Library Inside, British Library Direct Plus, ISI Proceedings)
- Web searching (e.g. Google Scholar, Science.gov, scricus.com)
- Administrative sources (clinical records, insurance data)
- Vital statistics data, government reports, Centers for Disease control and prevention data, population consensus and surveys.
- Medical books, grey literature and reports from experts.

For peer review only

7.3.6.3 Assessment of methodological quality

Assessment of methodological quality, or critical appraisal, is a process conducted in systematic reviews to establish the internal validity and risk of bias of studies that meet the review inclusion criteria.The JBI has developed a number of tools for assessing the quality of various quantitative study designs that are appropriate to use in systematic reviews assessing questions of etiology & risk. (See Appendix II).

The protocol should indicate which tool is going to be used that match the included study designs when determining methodological quality of papers to include in the review. JBI appraisal tools should be used preferentially; if not clear reasoning should be provided. Critical appraisal tools should be cited in the protocol and should be appended if the tools are modified in any way. Critical appraisal must be conducted by two reviewers independently of each other. The reviewers should then meet to discuss the results of their critical appraisal for their final appraisal. If the two reviewers disagree on the final critical appraisal and this cannot be resolved through discussion, a third reviewer may be required.

When detailing the 'Methods' of the review report, the section on appraisal should detail the approach to critical appraisal, not the assessment results, and should be consistent with the protocol. The approach to critical appraisal process should include information on what constitutes acceptable levels of information for appraisal and whether the decision to include or exclude studies following critical appraisal is based on meeting a predetermined proportion of criteria or weighing criteria differently. The authors of the review should state *a priori* in the review protocol the criteria used to determine the inclusion or exclusion of poor quality studies. The authors have to make explicit and agree on criteria to determine whether a study is of good, moderate or poor quality, and based on these criteria or a combination of criteria, the authors can decide whether to include only good quality studies or all studies irrespective of the quality. However, the importance of these criteria (e.g. selection, measurement bias, confounding) will vary with study type and problems specific to the review question.

The report should detail the criteria that were considered when determining the methodological quality of papers considered for inclusion in the review. In the systematic review, appraisal questions should be presented with the results, or appended.

For peer review only

7.3.6.3.1 Confounding and confounders

Confounding occurs when another factor other than primary factor of interest or being investigated, can directly influence the outcome being measured. To be classed as a confounding factor, it should not be a factor that appears in the casual pathway between and exposure and the outcome. Confounding bias is defined as “bias of the estimated effect of an exposure on an outcome due to the presence of common causes of the exposure and the outcome”.(Miquel 2014) (p.55) A confounder or confounding variable is a variable that can be used to decrease confounding bias when properly adjusted for.(Miquel 2014) (p.55)

Criteria for confounders are:(Rothman, Greenland et al. 2008) (p.132-134)

1. A confounding factor must be an extraneous risk factor for the disease; i.e. the confounder is a risk factor for the disease and the factor's association with disease arises from a causal pathway other than the one under study.
2. A confounding factor must be associated with the exposure under study in the source population (the population at risk from which the cases are derived).
3. A confounding factor must not be affected by the exposure or the disease. In particular, it cannot be an intermediate step in the causal path between the exposure and the disease. (For example, in the case of increased risk of lung cancer from high levels of red meat consumption, the confounding factor could possibly be the ‘cooking method’)(Cancer Australia 2014)

Confounding can be controlled in the design and analysis phases in the case of observational studies. The two approaches used for the control of confounding in the analysis of data are stratification and statistical modelling. In stratification, study participants are split into strata that are different groups based on levels of the potential confounding variable, for example age. Although this approach is a simple method, this approach is limited by the fact that only a certain a number of potential factors could be stratified. Hence, it is not a common approach to control for confounding in observational studies in the analysis phase. (Kahlert, Gribsholt et al. 2017) Statistical modelling (such as multiple logistic regression, conditional logistic regression, Cox proportional hazards regression, multivariable regression analysis) is used to estimate the strength of the relationship of interest while controlling for all of the potential confounders.(Webb and Bain 2011)

For peer review only

7.3.6.3.2 Types of bias in studies of etiology and risk

Bias is a particular concern when assessing the methodological quality of studies of etiology and risk. Bias refers to systematic errors in any type of study that result in an incorrect estimate of the association between putative risk or predictive factors and the study outcome(s). The taxonomy of bias is well covered in the Cochrane Handbook(Higgins and Green 2011) and in the Agency for Healthcare Research and Quality Methods Guide for Comparative Effectiveness Reviews.(Viswanathan, Ansari et al. 2008) If bias is suspected or reported, it is important to try and detect the direction of the bias, i.e. is it towards a change in the effect estimate of risk or not. Table 1 below shows the common types of bias that affect studies of etiology and risk.

Table 1: Common types of bias affecting studies of etiology and risk

**Type of bias**	**Definition**	**Check for**
Selection Bias	Systematic errors that result from procedures used to select study participants, from factors that influence participation in the study, or the ways in which data are collected or analyzed	**Sample**<br><br>e.g. inappropriate definition of the eligible population or use of an inappropriate sampling frame; oversampling of healthy volunteers; exclusion of those who cannot or do not access health care services /those from a CALD background/those who are illiterate; changes to population over time; attrition (general or greater in one group than another)/non-response related to survivorship and severity of illness or length of illness; institutional bias e.g. hospital patients are different form community living patients.<br><br>**Classification**<br><br>e.g. uneven diagnostic procedures; changes in procedures over time; observer bias; competing risks (e.g. attribution of cause of death); changes in guidelines/institutional policy outside the researchers' control and publication bias.
Information bias	Flawed measuring of independent and/or dependent variables/s that results in differential quality of information.	Inadequate detection; missing variables; misclassification; Hawthorne effect ; ecological fallacy; prestige/social desirability bias; recall bias; interviewer bias; reporting bias and missing data.

For peer review only

7.3.6.4 Data extraction

This section in the review report should include details of the types of data extracted from the included studies. Standardized data extraction tools allow the extraction of the same types of data across the included studies and are required for JBI systematic reviews. The protocol should detail what data the reviewers plan to extract from the included studies and the data extraction tool should be appended to the protocol.

The data extracted should include specific details about the participants, exposure of interest and outcomes of significance to the review question. Irrespective of the focus of the systematic review, additional data should be extracted, such as study methods, covariates and the sample size for each study included in the review. The methods of collection of exposure and outcome data (i.e. number of cigarettes or ppm of asbestos fibres or dust), which commonly include questionnaires, registries or interviews should also be stated.

Relative risk and other measures of association should be extracted, preferably those adjusted for the maximum number of covariates. Unadjusted results should be included only where no other data is provided. Epidemiological studies investigating the same association between an exposure and disease /condition provide different effect measures that may be too dissimilar to combine, which presents a challenge when combining studies in a meta-analysis. Each different study may report different measures of association, or estimates of effect, which most commonly include relative risks (RR), odds ratios (OR), hazard ratios (HR), standardized incidence ratios (SIR) or a standardized mortality ratios (SMR). An absolute risk reflects the observed or calculated probability of an outcome (disease) in a population exposed to a specific risk factor. A relative risk, which is the most common metric of risk, is simply the ratio of absolute risk in the group exposed to the risk factor of interest, to the absolute risk in a group (control) that is not exposed to the risk factor. An OR uses the odds of developing a disease in both groups to calculate a relative measure between two groups rather than the risk.

Where an absolute risk of the exposed group is presented relative to available existing data for a population group, this is referred to as a standardized ratio. Depending on whether incidence or mortality data is used will depend on whether the SIR or SMR is reported. Standardized mortality ratio refers to the ratio of observed and expected mortality, based on the age-sex-calendar period specific rates. Usually SMR greater than 1 implies higher than expected deaths and SMR less than 1 implies lower than expected deaths. Standardized incidence ratio is the ratio of the observed number of cases to the expected number of cases, based on the age-sex specific rates. A range of corrections, transformations and assumptions can be used to account for difference in the different types of data presented.

The following details are suggested at a minimum for extraction.

Study details
Author - This is an alphabetic or character code which is usually the first few characters of the primary study author's name. This serves as an easy way to identify the study in the bibliography
Year – the year of publication
Journal – the journal in which the article was published

Study method/characteristics
Study design – briefly describing the type of study design. For e.g. if it is a cohort study or a cross-sectional study.
Setting - may refer to hospital or community. May also refer to rural/urban etc.
Participants - – Includes age, sex, country/location, sample size, diagnosis and other relevant characteristics
Recruitment procedures utilized
Follow-up or study duration – any details on the duration of the study or follow-up of the participants
Exposure(s) of interest (Independent variable) – type, frequency, intensity, duration

Dependent variable (outcome)
Outcomes – the primary outcome measured and where relevant includes associated secondary outcomes.
Outcome measurements – describe the scales or tools used to measure the outcomes. For e.g. a standardized pain scale to measure pain.

Data analysis methods including statistical technique (e.g. regression), adjustment for confounding factors, etc.
Study results
Appropriate measures for effect size such as:

- Risk ratio
- Relative risk ratio
- Odds ratio

P value & 95% Confidence Intervals
Reviewer comments

For peer review only

7.3.6.5 Data synthesis

The protocol should detail how the reviewers plan to synthesize data extracted from included studies. The types of data it is anticipated will be synthesized should be consistent with the methods used for data collection and the included study designs. The review report should detail how the reviewers synthesized the data extracted from included studies and how it was applied consistently across all included studies.

As with all systematic reviews, there are various approaches to present the results, including a narrative, graphical or tabular summary, or meta-analysis (refer to the appropriate section below).(Munn, Tufanaru et al. 2014) There are some special considerations when conducting meta-analysis for questions related to etiology & risk.

For peer review only

7.3.6.5.1 Meta-analysis of observational research

A meta-analysis is a statistical procedure that combines the findings from multiple primary studies into a single overall summary estimate. A meta-analysis can be conducted to improve statistical power to detect a treatment effect, to estimate a summary average effect, to identify sub-groups associated with a negative outcome or a beneficial effect, and to explore differences in the size or direction of the treatment effect associated with study-specific variables. Interpretation of summary effect sizes from meta-analyses of epidemiological studies addressing etiological issues is difficult because of the differences in the factors controlled for in multivariate analyses from individual studies, and also because of poor reporting in the original studies with lack of adequate or complete details. For more information and guidance on meta-analysis, refer to Chapter 3 of this manual.

An overall effect size is reported in a meta-analysis. It is computed for each study and the findings are pooled together to draw overall inferences. There are many different types of effect size and it is possible to convert one effect size into another, so each really just offers a differently scaled measure of the strength of an effect or a relationship. Reviewers should be aware that there are different guidelines for the interpretation of practical significance of the effect sizes such as ORs and RRs.(Tufanaru C, Huang WJ et al. 2012) One proposed guide for interpretation of effect sizes suggests that a value of 2 for a risk estimate (such as a relative risk RR or an odds ratio OR) is considered the minimum significant value from a practical point of view; a value of 3 is considered moderate significant; a value of 4 is considered to indicate strong significance from a practical point of view.(Tufanaru C, Huang WJ et al. 2012)

Frequently primary published studies investigating risk of an exposure will design the study and present the available data at different levels of the exposure, or in different categories to reflect a ‘dose-response’ relationship between the exposure and outcome variable. Difficulties will naturally arise if different studies have used different exposure categories and have presented this data in a variety of different ways. A dose response relationship between an exposure and the outcome is most commonly investigated to strengthen the support for causal inference or causation.(Greenland and Longnecker 1992, Bekkering, Harris et al. 2008) Individual studies may present results in a stratified manner, either across different exposure groups or in different quantiles. For example, considering the risk of alcohol intake and lung cancer, the data may be presented as different exposure groups such as in glasses/week or in grams of alcohol. Irrespective of this, methods are available to combine the results of individual studies presenting such ‘trend’ data. Dependent on the type of data presented from such a dose response investigation, accepted methods exist to summarize the data to a consistent risk estimate which can then be subsequently used in meta-analysis.

Bekkering et al in a study on the usability of results in a meta-analysis reported that majority of usable results reported were odds, risk, or hazard ratios that compared one or more exposure categories with a baseline category.(Bekkering, Harris et al. 2008) They further suggest some advantages in reporting results in ORs, RRs and HRs, which include checking informally for nonlinear exposure effects, and easier interpretation of the magnitude of the association.(Bekkering, Harris et al. 2008) In case of nonlinear associations, there is a risk for conclusions from dose-response meta-analysis being misleading and it is suggested that linearity assumptions be checked for each study, when conducting dose-response meta-analysis.(Greenland and Longnecker 1992, Bekkering, Harris et al. 2008) Bekkering et al,(Bekkering, Harris et al. 2008) Chene and Thompson,(Chene and Thompson 1996) Greenland and Longnecker,(Greenland and Longnecker 1992) Hamling et al,(Hamling, Lee et al. 2008) and Orsini et al (Orsini, Bellocco et al. 2006) describe methods for conducting linear and non-linear dose-response meta-analyses. Essentially, for linear dose-response meta-analysis, the method involves estimation of a linear dose-response curve for each study when combining studies with different exposure category definitions. Further, it requires the numbers of cases and noncases (outcomes) and persons/person-years (person-time) and the effect estimates (RR or OR) with confidence intervals for at least three quantitative exposure categories.(Aromataris, Hopp L et al. 2011)

A note on heterogeneity (refer to Chapter 3 for more details)

Despite the impediment to meta-analysis that heterogeneity of the published data presents, be it for methodological, clinical or statistical reasons, meta-analysis of observational studies to inform etiology and risk is almost always possible and can offer a valid means to explore heterogeneity and its impact within a data set. A combined analysis of individual studies, beyond the outright aim of increased precision due to increased sample size, may be desirable as it allows the exploration of potential confounders and interactions and other modifying effects that may explain the heterogeneity among the included studies. It is suggested that the decision to conduct meta-analysis should not be just based on statistical considerations regarding heterogeneity but should be based on the review question, the characteristics of the studies, and the interpretability of the results.

For peer review only

7.3.6.5.2 The narrative synthesis of data

The results of all systematic reviews require some degree of narrative. Where a meta-analysis has been performed, that narrative may focus on synthesis of the characteristics of studies and their quality to explain and interpret the calculated effect estimates. In instances where meta-analysis has not been possible, the review authors will have to resort to narrative synthesis of the results of the included studies also. Narrative synthesis relies primarily on the use of words and text (tables are often included also, See Section 2.8.3) to summarise and explain the findings of a synthesis process. Its form may vary from the simple recounting and description of study characteristics, context, quality, and findings. The textual description of studies (individual or group of studies) and the thematic analysis methods are briefly presented below. Further exploration as well as worked examples for these approaches is provided by Lucas & co.(Lucas, Baird et al. 2007)

- Textual descriptions of individual studies. Summaries of individual studies can be structured to provide details of the setting, participants, exposure, and outcomes, along with any other factors of interest (e.g. the income level of the users, age of users, previous experiences, attrition, length of follow-up, sample size);
- Textual descriptions of groups of studies. Based on relevant criteria (e.g. types of participants) included studies can be sub-grouped. Subsequently, commentaries summarizing key aspects of the studies in relation to the sub-group within which they were included are produced. In a final step, the scope, differences and similarities among studies are used to draw conclusions across the studies.

Where a narrative synthesis is undertaken to describe the included studies and their conclusions, it is important to discern how the evidence was weighted and whether conclusions were biased. It is recommended that the characteristics of the studies and the data extracted are emphasised and tables, graphs, and other diagrams are made use of to compare data.(Lockwood and White 2012) The narrative summary will present quantitative data extracted from individual studies, as well as, where available, point estimates (a value that represents a best estimate of effects) and interval estimates (an estimated range of effects, presented as a 95% confidence interval).

Because a potentially large amount of data can be conveyed in a narrative summary, consistency can be ensured in the results section if all reviewers agree beforehand on a structure for the reporting of results. If a structure is not followed, the report of results may appear incomplete or unreliable.(Lockwood and White 2012) However, if included studies do not provide the relevant information to comply with a structure, it should be made clear in the summary. A textual combination of data is often used when the included studies are dissimilar in terms of patients, methods, or data.

For peer review only

7.3.6.5.3 The tabular synthesis of data

Tabulating the data begins with grouping the studies in discrete categories (e.g. based on types of participants, exposures, outcomes, country of origin, duration of the exposure, number of participants in each group, context, results and comments). When the analysis of the tables reveals the presence of dominant groups or clusters of characteristics groups of studies can be formed by which the subsequent synthesis can be organized; this technique is particularly useful when there are larger number of papers. Based on the type of data reported, a common results rubric can be tabulated as well (e.g. absolute difference, relative risk, odds ratio, favours exposure vs. favours no exposure column); this approach can serve as a first step in comparing the effects observed across the included studies.

Bellow you will find some suggested steps for tabulating information from studies included in a systematic review.(Khan, Kunz et al. 2003)

Suggested steps:

- Place features related to populations, exposures and outcomes in columns.
- Consider what subgroups of populations there are among included studies.
- Consider what subtypes of exposures there are.
- Consider the outcomes and their importance.
- Consider if studies need to be sub-classified according to study designs and quality.
- Populate the cells in the table with information from studies along rows in subgroups.
- Sort studies according to a feature that helps to understand their results (e.g. a characteristic of a population or exposure, rank order of quality, year of publication, etc.).

For peer review only

7.3.7 Results

The findings of the review should flow logically from the review objective/question i.e. they must ultimately answer the question! Findings should be extracted using JBI SUMARI and a narrative, tabular, graphical or meta-analysis should constitute part of this section. Reporting of results, as suggested by previous research, can include graphical summaries of study estimates and any combined estimate, a table listing descriptive information for each study, results of sensitivity testing and any subgroup analysis, and an indication of statistical uncertainty of findings.

This section should allow the reader to clearly follow how the included studies were identified and selected for inclusion in the review. In addition, the number of papers excluded should also be stated. There should be a narrative description of the process accompanied by a flowchart of the review process (from the PRISMA statement) detailing the flow from the search, through study selection, duplicates, full text retrieval, and any additions from 3rd search, appraisal, extraction and synthesis.

For peer review only

7.3.7.1 Description of studies

This section of the results should include an overall description of the included studies (with reference to the table in the appendices), with the main aim to provide some context to the results section and sufficient detail for the reader to determine if the included studies are similar enough to combine in meta-analysis. Specific items/points of interest from individual studies may also be highlighted here. Additional details may include the assessment of methodological quality, characteristics of the participants, location and types of exposures and outcomes. These can be presented in a narrative form, in a table or in both formats when studies vary in orientation and focus.

For peer review only

7.3.7.2 Methodological quality

This section should focus on methodological quality as determined by the relevant critical appraisal checklist. There should be a narrative summary of the overall methodological quality of the included studies, which can be supported (optional) by a table showing the results of the critical appraisal. Where only few studies are identified, or there are specific items of interest from included studies, these should be addressed in the narrative also, particularly where studies were deficient, or particularly good, i.e. with clear narrative regarding risk of bias/rigor of included studies. Use of N/A should also be justified in the text.

For peer review only

7.3.7.3 Findings of the review

This section should be organized in a meaningful way based on the review objectives and questions and types of exposures and outcomes and types of studies. This section should provide comprehensive information regarding the results of all performed meta-analyses and additional analyses such as sensitivity analysis and sub-group analysis. Point estimates and interval estimates (confidence intervals) should be reported. Before presenting any meta-analysis results, the conduct of meta-analyses should be justified; reviewers should explicitly provide commentaries regarding the clinical, methodological, and statistical heterogeneity of the studies included in meta-analyses and the appropriateness of conducting meta-analyses. Summary results from meta-analyses should be reported as summary point estimates and interval estimates. The meta-analysis forest plots for all performed meta-analyses should be presented in this section. A narrative summary should complement the forest plots and provide additional commentaries and explanations for all performed meta-analyses (Munn et al 2014).

Reviewers should report the funnel plot for publication bias if such assessment was appropriate and performed. Reviewers should include the results of assessment of risk of publication bias, including the results of statistical tests for publication bias, if such tests were used.

Even if meta-analysis is performed, a narrative summary should be included to supplement the technical details provided on the process and results of meta-analysis and to provide synthesis of data not captured in statistical meta-analysis.

If meta-analysis is not performed, a narrative summary should be included. The narrative summary should provide an overall summary of the findings of the included studies and their biases, strengths and limitations. The essence of narrative summary is that the results are summarized in words and in tables without any statistical meta-analysis. Textual commentaries and tables are used in order to summarize the results from the included studies and to provide context information for these results, thus facilitating understanding of the summarized results.

For peer review only

7.3.8 Discussion

This section should discuss the results of the synthesis as well as any limitations of the primary studies included in the review and of the review itself (i.e. language, access, timeframe, study design, etc.). The results should be discussed in the context of current literature, practice and policy.

The aim of this section is to explain and discuss the main findings – including the strength of the evidence, for each main outcome. It should address the issues arising from the conduct of the review including limitations and issues arising from the findings of the review (such as search limitations). The discussion does seek to establish a line of argument based on the findings regarding the exposure and its association with the outcomes identified in the protocol. The application and relevance of the findings to relevant stakeholders (e.g. healthcare providers, patients and policy makers) should also be discussed in this section.

Points to consider this section include:

- Where any problems identified undertaking the search (perhaps there is little primary research on this topic or perhaps it is poorly indexed by the databases that were searched or perhaps the search was insufficient)?
- What limitations were found in the included primary research (e.g. were there inconsistencies or errors in reporting)?
- How do the review findings fit with what is currently known on the topic (from issues highlighted in the Background section)?
- Are the findings generalizable to other populations of participants/healthcare settings etc.?

Suggested layout of Discussion section:

Paragraph 1 – Begin your discussion with the:

- Amount and weight of available evidence
- Any particular feature/s associated with future risk of disease/harm/outcome
- Limitations to establish the reliability of results of the included studies (e.g. biases, data issues)

Paragraph 2 – set in context.

- Set the results in context of other knowledge on the topic, i.e. compare your work with previous systematic reviews or current opinions and guidelines.

Paragraph 3 – outline strengths and weaknesses of the meta-analytic methods used.

- Strengths: e.g. multiple reviewers reduced inclusion bias; which moderating variables were identified and how they were managed e.g. study design; determined that the effect estimate was sufficiently large in practical as well as statistical terms; determined precision of the effect; determined heterogeneity of the participants to enable generalisation of findings; conducted sensitivity analyses to assess any changes in the pooled effect estimator.
- Weaknesses: bias e.g. included only English language publications, unable to access suitable grey literature; possibility of missing (explanatory) variable/s, some issues with interpretation of findings.

Paragraph 4 – discuss limitations to establish the reliability of result/s.

- Of your review (bias)

For peer review only

7.3.9 Conclusion and Recommendations

This section should begin with an overall conclusion based on the results. The conclusions drawn should match with the review objective/question.

The conclusion section of a systematic review should provide a general interpretation of the findings in the context of other evidence and provide a detailed discussion of issues arising from the findings of the review and demonstrate the significance of the review findings to practice and research. Areas that may be addressed include:

- A summary of the major findings of the review;
- Issues related to the quality of the research within the area of interest;
- Other issues of relevance; and
- Potential limitations of the systematic review.

Recommendations for practice

It should be stated how the findings of the review impact on public health issues and clinical practice in the area. If there is sufficient evidence to make specific recommendations for practice, then the appropriate JBI Grades of Recommendation should be assigned to each recommendation based on the study design that led to the recommendation.

Recommendations for research

This section should include clear, specific recommendations for future research based on gaps in knowledge identified from the results of the review. Recommendations for research should avoid generalised statements calling for further research, but should be linked to specific issues.

For peer review only

7.3.10 Appendices

Here are several required appendices for a JBI review:

- Appendix I: Search strategy
  - A detailed and complete search strategy for all of the major databases and other sites and sources searched must be appended. Major databases that were searched must be identified, including the search platform used where necessary. All search filters with logic employed should be displayed, including the number of records returned.
- Appendix II: Table of included studies
  - A table of included studies is crucial to allow a snapshot of the studies included in the review.
- Appendix III: List of excluded studies
  - At a minimum, a list of studies excluded at the full text selection stage, if any, must be appended and reasons for exclusion should be provided for each study.

For peer review only

7.4 Chapter references

Akpalu, B., K. Ae-Ngibise, F. Agbokey, G. Adjei and Y. Enuameh (2012). "Association between Plasmodium falciparum malaria and the mental health of children between five years and nineteen years in subSaharan Africa: A systematic review." JBI Database of Systematic Reviews and Implementation Reports **10**(28): 1-14.

Aromataris, E., Hopp L and M. Z. (2011). Synthesizing Evidence of Risk. Philadelphia, Lippincott Williams & Wilkins.

Bekkering, G. E., R. J. Harris, S. Thomas, A. M. Mayer, R. Beynon, A. R. Ness, R. M. Harbord, C. Bain, G. D. Smith and J. A. Sterne (2008). "How much of the data published in observational studies of the association between diet and prostate or bladder cancer is usable for meta-analysis?" Am J Epidemiol **167**(9): 1017-1026.

Cancer Australia (2014). Risk factors for Lung cancer: a systematic review. Surry Hills, NSW, Cancer Australia.

Chene, G. and S. G. Thompson (1996). "Methods for summarizing the risk associations of quantitative variables in epidemiologic studies in a consistent form." Am J Epidemiol **144**(6): 610-621.

DerSimonian, R. and N. Laird (1986). "Meta-analysis in clinical trials." Control Clin Trials **7**(3): 177-188.

DerSimonian, R. and N. Laird (2015). "Meta-analysis in clinical trials revisited." Contemp Clin Trials **45**(Pt A): 139-145.

Gagnier, J. J., G. Kienle, D. G. Altman, D. Moher, H. Sox and D. Riley (2014). "The CARE guidelines: consensus-based clinical case report guideline development." J Clin Epidemiol **67**(1): 46-51.

Greenland, S. (1987). "Quantitative methods in the review of epidemiologic literature." Epidemiol Rev **9**: 1-30.

Greenland, S. and M. P. Longnecker (1992). "Methods for trend estimation from summarized dose-response data, with applications to meta-analysis." Am J Epidemiol **135**(11): 1301-1309.

Hamling, J., P. Lee, R. Weitkunat and M. Ambuhl (2008). "Facilitating meta-analyses by deriving relative effect and precision estimates for alternative comparisons from a set of estimates presented by exposure level or disease category." Stat Med **27**(7): 954-970.

Higgins, J. and S. Green (2011). Cochrane Handbook for Systematic Reviews of Interventions The Cochrane Collaboration.

Higgins, J. P. T., S. G. Thompson, J. J. Deeks and D. G. Altman (2003). "Measuring inconsistency in meta-analyses." BMJ : British Medical Journal **327**(7414): 557-560.

Hill, A. B. (1965). "The Environment and Disease: Association or Causation?" Proceedings of the Royal Society of Medicine **58**(5): 295-300.

Kahlert, J., S. B. Gribsholt, H. Gammelager, O. M. Dekkers and G. Luta (2017). "Control of confounding in the analysis phase – an overview for clinicians." Clinical Epidemiology **9**: 195-204.

Khan, K. S., R. Kunz, J. Kleijnen and G. Antes (2003). "Five steps to conducting a systematic review." J R Soc Med **96**(3): 118-121.

Kraemer, H. C., A. E. Kazdin, D. R. Offord, R. C. Kessler, P. S. Jensen and D. J. Kupfer (1997). "Coming to terms with the terms of risk." Arch Gen Psychiatry **54**(4): 337-343.

Lockwood, C. and S. White (2012). Synthesizing descriptive evidence, Lippincott Williams & Wilkins /Joanna Briggs Institute.

Lucas, P. J., J. Baird, L. Arai, C. Law and H. M. Roberts (2007). "Worked examples of alternative methods for the synthesis of qualitative and quantitative research in systematic reviews." BMC Med Res Methodol **7**: 4.

Miquel, P. (2014). A Dictionary of Epidemiology, Oxford University Press.

Moher, D., A. Liberati, J. Tetzlaff, D. G. Altman and P. G. and the (2009). "Preferred reporting items for systematic reviews and meta-analyses: The prisma statement." Annals of Internal Medicine **151**(4): 264-269.

Munn, Z., C. Tufanaru and E. Aromataris (2014). "JBI's systematic reviews: data extraction and synthesis." Am J Nurs **114**(7): 49-54.

Orsini, N., R. Bellocco and S. Greenland (2006). "Generalized least squares for trend estimation of summarized dose-response data." Stata Journal **6**(1): 40-57.

For peer review only

Ratib, S., E. Burden-Teh, J. Leonardi-Bee, C. Harwood and F. Bath-Hextall (2016). "Long-term topical corticosteroid use and risk of skin cancer: a systematic review protocol." JBI Database of Systematic Reviews and Implementation Reports **14**(12): 64-73.

Rothman, K., S. Greenland and T. Lash (2008). Validity in Epidemiologic Studies. In Rothman KJ, Greenland S, Lash TL. Modern Epidemiology. Philadelphia, Lippincott Williams & Wilkins.

Rothman, K. J., S. Greenland, C. Poole and T. L. Lash (2008). Causation and Causal Inference. In Rothman KJ, Greenland S, Lash TL. Modern Epidemiology. . Philadelphia, Lippincott Williams & Wilkins.

Stroup, D. F., J. A. Berlin, S. C. Morton, I. Olkin, G. D. Williamson, D. Rennie, D. Moher, B. J. Becker, T. A. Sipe and S. B. Thacker (2000). "Meta-analysis of observational studies in epidemiology: a proposal for reporting. Meta-analysis Of Observational Studies in Epidemiology (MOOSE) group." Jama **283**(15): 2008-2012.

The Joanna Briggs Institute a (2014). The Joanna Briggs Institute Reviewers' Manual. Australia.

The Joanna Briggs Institute b (2014). Joanna Briggs Institute Reviewers' Manual: The Systematic Review of Prevalence and Incidence Data. Adelaide, Australia, The Joanna Briggs Institute.

Thiese, M. S. (2014). "Observational and interventional study design types; an overview." Biochemia Medica **24**(2): 199-210.

Tufanaru C, Huang WJ, Tsay S-F and C. S-S (2012). Statistics for Systematic Review Authors. Philadelphia, Lippincott Williams & Wilkins.

Viswanathan, M., M. T. Ansari, N. D. Berkman, S. Chang, L. Hartling, M. McPheeters, P. L. Santaguida, T. Shamliyan, K. Singh, A. Tsertsvadze and J. R. Treadwell (2008). AHRQ Methods for Effective Health Care Assessing the Risk of Bias of Individual Studies in Systematic Reviews of Health Care Interventions. Methods Guide for Effectiveness and Comparative Effectiveness Reviews. Rockville (MD), Agency for Healthcare Research and Quality (US).

von Elm, E., D. G. Altman, M. Egger, S. J. Pocock, P. C. Gotzsche and J. P. Vandenbroucke (2007). "Strengthening the Reporting of Observational Studies in Epidemiology (STROBE) statement: guidelines for reporting observational studies." BMJ **335**(7624): 806-808.

Warren, F., K. Abrams, S. Golder and A. Sutton (2012). "Systematic review of methods used in meta-analyses where a primary outcome is an adverse or unintended event." BMC Med Res Methodol **12**: 64.

Webb, P. and C. Bain (2011). Essential Epidemiology. Cambridge, Cambridge University

Wolf, F. (1986). Meta-Analysis: Quantitative methods for research synthesis. Beverly Hills, CA: Sage.

For peer review only

Appendix 7.1 Critical appraisal checklist for cohort studies

JBI Critical Appraisal Checklist for Cohort Studies

Reviewer____Date___________________________ ____________

Author ____Year___________Record Number _ ____________

	Yes	No	Unclear	Not applicable
1. Were the two groups similar and recruited from the same population?				
1. Were the exposures measured similarly to assign people to both exposed and unexposed groups?				
1. Was the exposure measured in a valid and reliable way?				
1. Were confounding factors identified?				
1. Were strategies to deal with confounding factors stated?				
1. Were the groups/participants free of the outcome at the start of the study (or at the moment of exposure)?				
1. Were the outcomes measured in a valid and reliable way?				
1. Was the follow up time reported and sufficient to be long enough for outcomes to occur?				
1. Was follow up complete, and if not, were the reasons to loss to follow up described and explored?				
1. Were strategies to address incomplete follow up utilized?				
1. Was appropriate statistical analysis used?				

Overall appraisal: Include Exclude Seek further info

Comments (Including reason for exclusion)

____ ____________

____ ____________

____ ____________

Explanation of cohort studies critical appraisal

For peer review only

How to cite: Moola S, Munn Z, Tufanaru C, Aromataris E, Sears K, Sfetcu R, Currie M, Lisy K, Qureshi R, Mattis P, Mu P. Chapter 7: Systematic reviews of etiology and risk. In: Aromataris E, Munn Z (Editors). JBI Manual for Evidence Synthesis. JBI, 2020. Available from https://synthesismanual.jbi.global. https://doi.org/10.46658/JBIMES-20-08

Cohort studies Critical Appraisal Tool

Answers: Yes, No, Unclear or Not/Applicable

1. Were the two groups similar and recruited from the same population?

Check the paper carefully for descriptions of participants to determine if patients within and across groups have similar characteristics in relation to exposure (e.g. risk factor under investigation). The two groups selected for comparison should be as similar as possible in all characteristics except for their exposure status, relevant to the study in question. The authors should provide clear inclusion and exclusion criteria that they developed prior to recruitment of the study participants.

2. Were the exposures measured similarly to assign people to both exposed and unexposed groups?

A high quality study at the level of cohort design should mention or describe how the exposures were measured. The exposure measures should be clearly defined and described in detail. This will enable reviewers to assess whether or not the participants received the exposure of interest.

3. Was the exposure measured in a valid and reliable way?

The study should clearly describe the method of measurement of exposure. Assessing validity requires that a 'gold standard' is available to which the measure can be compared. The validity of exposure measurement usually relates to whether a current measure is appropriate or whether a measure of past exposure is needed.

Reliability refers to the processes included in an epidemiological study to check repeatability of measurements of the exposures. These usually include intra-observer reliability and inter-observer reliability.

4. Were confounding factors identified?

Confounding has occurred where the estimated intervention exposure effect is biased by the presence of some difference between the comparison groups (apart from the exposure investigated/of interest). Typical confounders include baseline characteristics, prognostic factors, or concomitant exposures (e.g. smoking). A confounder is a difference between the comparison groups and it influences the direction of the study results. A high quality study at the level of cohort design will identify the potential confounders and measure them (where possible). This is difficult for studies where behavioral, attitudinal or lifestyle factors may impact on the results.

5. Were strategies to deal with confounding factors stated?

Strategies to deal with effects of confounding factors may be dealt within the study design or in data analysis. By matching or stratifying sampling of participants, effects of confounding factors can be adjusted for. When dealing with adjustment in data analysis, assess the statistics used in the study. Most will be some form of multivariate regression analysis to account for the confounding factors measured. Look out for a description of statistical methods as regression methods such as logistic regression are usually employed to deal with confounding factors/variables of interest.

6. Were the groups/participants free of the outcome at the start of the study (or at the moment of exposure)?

The participants should be free of the outcomes of interest at the start of the study. Refer to the 'methods' section in the paper for this information, which is usually found in descriptions of participant /sample recruitment, definitions of variables, and/or inclusion/exclusion criteria.

For peer review only

7. Were the outcomes measured in a valid and reliable way?

Read the methods section of the paper. If for e.g. lung cancer is assessed based on existing definitions or diagnostic criteria, then the answer to this question is likely to be yes. If lung cancer is assessed using observer reported, or self-reported scales, the risk of over- or under-reporting is increased, and objectivity is compromised. Importantly, determine if the measurement tools used were validated instruments as this has a significant impact on outcome assessment validity.

Having established the objectivity of the outcome measurement (e.g. lung cancer) instrument, it's important to establish how the measurement was conducted. Were those involved in collecting data trained or educated in the use of the instrument/s? (e.g. radiographers). If there was more than one data collector, were they similar in terms of level of education, clinical or research experience, or level of responsibility in the piece of research being appraised?

8. Was the follow up time reported and sufficient to be long enough for outcomes to occur?

The appropriate length of time for follow up will vary with the nature and characteristics of the population of interest and/or the intervention, disease or exposure. To estimate an appropriate duration of follow up, read across multiple papers and take note of the range for duration of follow up. The opinions of experts in clinical practice or clinical research may also assist in determining an appropriate duration of follow up. For example, a longer timeframe may be needed to examine the association between occupational exposure to asbestos and the risk of lung cancer. It is important, particularly in cohort studies that follow up is long enough to enable the outcomes. However, it should be remembered that the research question and outcomes being examined would probably dictate the follow up time

9. Was follow up complete, and if not, were the reasons to loss to follow up described and explored?

It is important in a cohort study that a greater percentage of people are followed up. As a general guideline, at least 80% of patients should be followed up. Generally a dropout rate of 5% or less is considered insignificant. A rate of 20% or greater is considered to significantly impact on the validity of the study. However, in observational studies conducted over a lengthy period of time a higher dropout rate is to be expected. A decision on whether to include or exclude a study because of a high dropout rate is a matter of judgement based on the reasons why people dropped out, and whether dropout rates were comparable in the exposed and unexposed groups.

Reporting of efforts to follow up participants that dropped out may be regarded as an indicator of a well conducted study. Look for clear and justifiable description of why people were left out, excluded, dropped out etc. If there is no clear description or a statement in this regards, this will be a 'No'.

10. Were strategies to address incomplete follow up utilized?

Some people may withdraw due to change in employment or some may die; however, it is important that their outcomes are assessed. Selection bias may occur as a result of incomplete follow up. Therefore, participants with unequal follow up periods must be taken into account in the analysis, which should be adjusted to allow for differences in length of follow up periods. This is usually done by calculating rates which use person-years at risk, i.e. considering time in the denominator.

11. Was appropriate statistical analysis used?

As with any consideration of statistical analysis, consideration should be given to whether there was a more appropriate alternate statistical method that could have been used. The methods section of cohort studies should be detailed enough for reviewers to identify which analytical techniques were used (in particular, regression or stratification) and how specific confounders were measured.

For studies utilizing regression analysis, it is useful to identify if the study identified which variables were included and how they related to the outcome. If stratification was the analytical approach used, were the strata of analysis defined by the specified variables? Additionally, it is also important to assess the appropriateness of the analytical strategy in terms of the assumptions associated with the approach as differing methods of analysis are based on differing assumptions about the data and how it will respond.

For peer review only - http://bmjopen.bmj.com/site/about/guidelines.xhtml

Appendix 7.2 Critical appraisal checklist for case-control studies

JBI Critical Appraisal Checklist for Case Control Studies

Reviewer____Date__________________________

Author ____Year___________Record Number__

	Yes	No	Unclear	Not applicable
1. Were the groups comparable other than the presence of disease in cases or the absence of disease in controls?				
1. Were cases and controls matched appropriately?				
1. Were the same criteria used for identification of cases and controls?				
1. Was exposure measured in a standard, valid and reliable way?				
1. Was exposure measured in the same way for cases and controls?				
1. Were confounding factors identified?				
1. Were strategies to deal with confounding factors stated?				
1. Were outcomes assessed in a standard, valid and reliable way for cases and controls?				
1. Was the exposure period of interest long enough to be meaningful?				
1. Was appropriate statistical analysis used?				

Overall appraisal: Include Exclude Seek further info

Comments (Including reason for exclusion)

For peer review only

Explanation of case control studies critical appraisal

How to cite: Moola S, Munn Z, Tufanaru C, Aromataris E, Sears K, Sfetcu R, Currie M, Lisy K, Qureshi R, Mattis P, Mu P. Chapter 7: Systematic reviews of etiology and risk. In: Aromataris E, Munn Z (Editors). *JBI Manual for Evidence Synthesis.* JBI, 2020. Available from https://synthesismanual.jbi.global. https://doi.org/10.46658/JBIMES-20-08

Case Control Studies Critical Appraisal Tool

Answers: Yes, No, Unclear or Not/Applicable

1. Were the groups comparable other than presence of disease in cases or absence of disease in controls?

The control group should be representative of the source population that produced the cases. This is usually done by individual matching; wherein controls are selected for each case on the basis of similarity with respect to certain characteristics other than the exposure of interest. Frequency or group matching is an alternative method. Selection bias may result if the groups are not comparable.

2. Were cases and controls matched appropriately?

As in item 1, the study should include clear definitions of the source population. Sources from which cases and controls were recruited should be carefully looked at. For example, cancer registries may be used to recruit participants in a study examining risk factors for lung cancer, which typify population-based case control studies. Study participants may be selected from the target population, the source population, or from a pool of eligible participants (such as in hospital-based case control studies).

3. Were the same criteria used for identification of cases and controls?

It is useful to determine if patients were included in the study based on either a specified diagnosis or definition. This is more likely to decrease the risk of bias. Characteristics are another useful approach to matching groups, and studies that did not use specified diagnostic methods or definitions should provide evidence on matching by key characteristics. A case should be defined clearly. It is also important that controls must fulfil all the eligibility criteria defined for the cases except for those relating to diagnosis of the disease.

4. Was exposure measured in a standard, valid and reliable way?

The study should clearly describe the method of measurement of exposure. Assessing validity requires that a 'gold standard' is available to which the measure can be compared. The validity of exposure measurement usually relates to whether a current measure is appropriate or whether a measure of past exposure is needed.

Case control studies may investigate many different ‘exposures’ that may or may not be associated with the condition. In these cases, reviewers should use the main exposure of interest for their review to answer this question when using this tool at the study level.

Reliability refers to the processes included in an epidemiological study to check repeatability of measurements of the exposures. These usually include intra-observer reliability and inter-observer reliability.

5. Was exposure measured in the same way for cases and controls?

As in item 4, the study should clearly describe the method of measurement of exposure. The exposure measures should be clearly defined and described in detail. Assessment of exposure or risk factors should have been carried out according to same procedures or protocols for both cases and controls.

6. Were confounding factors identified?

Confounding has occurred where the estimated intervention exposure effect is biased by the presence of some difference between the comparison groups (apart from the exposure investigated/of interest). Typical confounders include baseline characteristics, prognostic factors, or concomitant exposures (e.g. smoking). A confounder is a difference between the comparison groups and it influences the direction of the study results. A high quality study at the level of case control design will identify the potential confounders and measure them (where possible). This is difficult for studies where behavioral, attitudinal or lifestyle factors may impact on the results.

7. Were strategies to deal with confounding factors stated?

For peer review only

Strategies to deal with effects of confounding factors may be dealt within the study design or in data analysis. By matching or stratifying sampling of participants, effects of confounding factors can be adjusted for. When dealing with adjustment in data analysis, assess the statistics used in the study. Most will be some form of multivariate regression analysis to account for the confounding factors measured. Look out for a description of statistical methods as regression methods such as logistic regression are usually employed to deal with confounding factors/ variables of interest.

8. Were outcomes assessed in a standard, valid and reliable way for cases and controls?

Read the methods section of the paper. If for e.g. lung cancer is assessed based on existing definitions or diagnostic criteria, then the answer to this question is likely to be yes. If lung cancer is assessed using observer reported, or self-reported scales, the risk of over- or under-reporting is increased, and objectivity is compromised. Importantly, determine if the measurement tools used were validated instruments as this has a significant impact on outcome assessment validity.

Having established the objectivity of the outcome measurement (e.g. lung cancer) instrument, it's important to establish how the measurement was conducted. Were those involved in collecting data trained or educated in the use of the instrument/s? (e.g. radiographers). If there was more than one data collector, were they similar in terms of level of education, clinical or research experience, or level of responsibility in the piece of research being appraised?

9. Was the exposure period of interest long enough to be meaningful?

It is particularly important in a case control study that the exposure time was sufficient enough to show an association between the exposure and the outcome. It may be that the exposure period may be too short or too long to influence the outcome.

10. Was appropriate statistical analysis used?

As with any consideration of statistical analysis, consideration should be given to whether there was a more appropriate alternate statistical method that could have been used. The methods section should be detailed enough for reviewers to identify which analytical techniques were used (in particular, regression or stratification) and how specific confounders were measured.

For studies utilizing regression analysis, it is useful to identify if the study identified which variables were included and how they related to the outcome. If stratification was the analytical approach used, were the strata of analysis defined by the specified variables? Additionally, it is also important to assess the appropriateness of the analytical strategy in terms of the assumptions associated with the approach as differing methods of analysis are based on differing assumptions about the data and how it will respond.

For peer review only

Appendix 7.3 Critical appraisal checklists for case series

JBI Critical Appraisal Checklist for Case Series

Reviewer____Date____________________
____

-

-

Author ____Year__________Record Number _________

	Yes	No	Unclear	Not applicable
1. Were there clear criteria for inclusion in the case series?				
1. Was the condition measured in a standard, reliable way for all participants included in the case series?				
1. Were valid methods used for identification of the condition for all participants included in the case series?				
1. Did the case series have consecutive inclusion of participants?				
1. Did the case series have complete inclusion of participants?				
1. Was there clear reporting of the demographics of the participants in the study?				
1. Was there clear reporting of clinical information of the participants?				
1. Were the outcomes or follow up results of cases clearly reported?				
1. Was there clear reporting of the presenting site(s)/clinic(s) demographic information?				
1. Was statistical analysis appropriate?				

Overall appraisal: Include Exclude Seek further info

Comments (Including reason for exclusion)

____

____

____

Introduction to the Case Series Critical Appraisal Tool

For peer review only

How to cite: Moola S, Munn Z, Tufanaru C, Aromataris E, Sears K, Sfetcu R, Currie M, Lisy K, Qureshi R, Mattis P, Mu P. Chapter 7: Systematic reviews of etiology and risk. In: Aromataris E, Munn Z (Editors). *JBI Manual for Evidence Synthesis.* JBI, 2020. Available from https://synthesismanual.jbi.global. https://doi.org/10.46658/JBIMES-20-08

-

The definition of a case series varies across the medical literature, which has resulted in inconsistent use of this term (Appendix 1).[1-3] The gamut of case studies is wide, with some studies claiming to be a case series realistically being nothing more than a collection of case reports, with others more akin to cohort studies or even quasi-experimental before and after studies. This has created difficulty in assigning 'case series' a position in the hierarchy of evidence and identifying and appropriate critical appraisal tool.[1, 2]

Dekkers et al. define a case series as a study in which 'only patients with the outcome are sampled (either those who have an exposure or those who are selected without regard to exposure), which does not permit calculation of an absolute risk.'[1p.39] The outcome could be a disease or a disease related outcome. This is contrasted to cohort studies where sampling is based on exposure (or characteristic), and case- control studies where there is a comparison group without the disease.

The completeness of a case series contributes to its reliability.[1] Studies that indicate a consecutive and complete inclusion are more reliable than those that do not. For example, a case series that states 'we included all patients (24) with osteosarcoma who presented to our clinic between March 2005 and June 2006' is more reliable than a study that simply states 'we report a case series of 24 people with osteosarcoma.'

For the purposes of this checklist, we agree with the principles outlined in the Dekker et al. paper, and define case series as studies where only patients with a certain disease or disease-related outcome are sampled. Some of the items below relate to risk of bias, whilst others relate to ensuring adequate reporting and statistical analysis. A response of 'no' to any of the questions below negatively impacts the quality of a case series.

Tool Guidance

Answers: Yes, No, Unclear or Not/Applicable

1. Were there clear criteria for inclusion in the case series?

The authors should provide clear inclusion (and exclusion criteria where appropriate) for the study participants. The inclusion/exclusion criteria should be specified (e.g., risk, stage of disease progression) with sufficient detail and all the necessary information critical to the study.

2. Was the condition measured in a standard, reliable way for all participants included in the case series?

The study should clearly describe the method of measurement of the condition. This should be done in a standard (i.e. same way for all patients) and reliable (i.e. repeatable and reproducible results) way.

3. Were valid methods used for identification of the condition for all participants included in the case series?

Many health problems are not easily diagnosed or defined and some measures may not be capable of including or excluding appropriate levels or stages of the health problem. If the outcomes were assessed based on existing definitions or diagnostic criteria, then the answer to this question is likely to be yes. If the outcomes were assessed using observer reported, or self-reported scales, the risk of over- or under-reporting is increased, and objectivity is compromised. Importantly, determine if the measurement tools used were validated instruments as this has a significant impact on outcome assessment validity.

4. Did the case series have consecutive inclusion of participants?

Studies that indicate a consecutive inclusion are more reliable than those that do not. For example, a case series that states 'we included all patients (24) with osteosarcoma who presented to our clinic between March 2005 and June 2006' is more reliable than a study that simply states 'we report a case series of 24 people with osteosarcoma.'

5. Did the case series have complete inclusion of participants?

The completeness of a case series contributes to its reliability (1). Studies that indicate a complete inclusion are more reliable than those that do not. A stated above, a case series that states 'we included all patients (24) with osteosarcoma who presented to our clinic between March 2005 and June 2006' is more reliable than a study that simply states 'we report a case series of 24 people with osteosarcoma.'

6. Was there clear reporting of the demographics of the participants in the study?

For peer review only

The case series should clearly describe relevant participant's demographics such as the following information where relevant: participant's age, sex, education, geographic region, ethnicity, time period, education.

7. Was there clear reporting of clinical information of the participants?

There should be clear reporting of clinical information of the participants such as the following information where relevant: disease status, comorbidities, stage of disease, previous interventions/treatment, results of diagnostic tests, etc.

8. Were the outcomes or follow-up results of cases clearly reported?

The results of any intervention or treatment should be clearly reported in the case series. A good case study should clearly describe the clinical condition post-intervention in terms of the presence or lack of symptoms. The outcomes of management/treatment when presented as images or figures can help in conveying the information to the reader/clinician. It is important that adverse events are clearly documented and described, particularly a new or unique condition is being treated or when a new drug or treatment is used. In addition, unanticipated events, if any that may yield new or useful information should be identified and clearly described.

9. Was there clear reporting of the presenting site(s)/clinic(s) demographic information?

Certain diseases or conditions vary in prevalence across different geographic regions and populations (e.g. women vs. men, sociodemographic variables between countries). The study sample should be described in sufficient detail so that other researchers can determine if it is comparable to the population of interest to them.

10. Was statistical analysis appropriate?

As with any consideration of statistical analysis, consideration should be given to whether there was a more appropriate alternate statistical method that could have been used. The methods section of studies should be detailed enough for reviewers to identify which analytical techniques were used and whether these were suitable.

References

1 Dekkers OM, Egger M, Altman DG, Vandenbroucke JP. Distinguishing case series from cohort studies. Annals of Internal Medicine. 2012;156(1 Part 1):37-40.

2 Esene IN, Ngu J, El Zoghby M, Solaroglu I, Sikod AM, Kotb A et al. Case series and descriptive cohort studies in neurosurgery: the confusion and solution. Child's Nervous System. 2014;30 (8):1321-32.

3 Abu-Zidan FM, Abbas AK, Hefny AF. Clinical "case series": a concept analysis. African Health Sciences. 2012;12(4):557-62.

4 Straus SE, Richardson WS, Glasziou P, Haynes RB. Evidence-based medicine: How to practice and teach EBM. 3rd Edition ed: Elsevier 2005.

Appendix 1: Case series definitions:

'A report on a series of patients with an outcome of interest. No control group is involved.'(4) (p 279)

'A case series is a descriptive study involving a group of patients who all have the same disease or condition: the aim is to describe common and differing characteristics of a particular group of individuals' (Oxford Handbook of medical statistics)

'A group or series of case reports involving patients who were given similar treatment. Reports of case series usually contain detailed information about the individual patients. This includes demographic information (for example, age, gender, ethnic origin) and information on diagnosis, treatment, response to treatment, and follow-up after treatment.' Law K, Howick J. OCEBM Table of Evidence Glossary. 2013 [cited 2014 10th January]; Available from: http://www.cebm.net/index.aspx?o=1116

'A **case series** (also known as a clinical **series**) is a type of medical research study that tracks subjects with a known exposure, such as patients who have received a similar treatment, or examines their medical records for exposure and outcome.' Wikipedia

For peer review only

'A study which makes observations on a series of individuals, usually all receiving the same intervention, with no control group. Comments: At this stage it is unclear whether case series should be included in Cochrane systematic reviews, but we have left them in the list so that working groups can consider whether there are circumstances in which it would be appropriate to include them, and to assess risk of bias. A particular reason for including case series might be where they provide evidence relating to adverse effects of an intervention. Potential examples of risk of bias might be that if a case series does not [attempt to] recruit consecutive participants, this might introduce a risk of selection bias, while some case series could be at risk of detection bias, if the circumstances in which adverse effects are reported (or elicited) are not standardised.' http://bmg.cochrane.org/research-projectscochrane-risk-bias-tool

For peer review only

Appendix 7.4 Critical appraisal checklist for case reports

JBI Critical Appraisal Checklist for Case Reports

Reviewer____Date_________________________ _________

Author ____Year___________Record Number _________

	Yes	No	Unclear	Not applicable
1. Were patient's demographic characteristics clearly described?				
1. Was the patient's history clearly described and presented as a timeline?				
1. Was the current clinical condition of the patient on presentation clearly described?				
1. Were diagnostic tests or assessment methods and the results clearly described?				
1. Was the intervention(s) or treatment procedure(s) clearly described?				
1. Was the post-intervention clinical condition clearly described?				
1. Were adverse events (harms) or unanticipated events identified and described?				
1. Does the case report provide takeaway lessons?				

Overall appraisal: Include Exclude Seek further info

Comments (Including reason for exclusion)

____ ___________

____ ___________

____ ___________

Explanation of case reports critical appraisal

How to cite: Moola S, Munn Z, Tufanaru C, Aromataris E, Sears K, Sfetcu R, Currie M, Lisy K, Qureshi R, Mattis P, Mu P. Chapter 7: Systematic reviews of etiology and risk. In: Aromataris E, Munn Z (Editors). *JBI Manual for Evidence Synthesis.* JBI, 2020. Available from https://synthesismanual.jbi.global. https://doi.org/10.46658/JBIMES-20-08

Case Reports Critical Appraisal Tool

Answers: Yes, No, Unclear or Not/Applicable

1. Were patient's demographic characteristics clearly described?

For peer review only

Does the case report clearly describe patient's age, sex, race, medical history, diagnosis, prognosis, previous treatments, past and current diagnostic test results, and medications? The setting and context may also be described.

2. Was the patient's history clearly described and presented as a timeline?

A good case report will clearly describe the history of the patient, their medical, family and psychosocial history including relevant genetic information, as well as relevant past interventions and their outcomes. (CARE Checklist 2013)

3. Was the current clinical condition of the patient on presentation clearly described?

The current clinical condition of the patient should be described in detail including the uniqueness of the condition/disease, symptoms, frequency and severity. The case report should also be able to present whether differential diagnoses was considered.

4. Were diagnostic tests or methods and the results clearly described?

A reader of the case report should be provided sufficient information to understand how the patient was assessed. It is important that all appropriate tests are ordered to confirm a diagnosis and therefore the case report should provide a clear description of various diagnostic tests used (whether a gold standard or alternative diagnostic tests). Photographs or illustrations of diagnostic procedures, radiographs, or treatment procedures are usually presented when appropriate to convey a clear message to readers.

5. Was the intervention(s) or treatment procedure(s) clearly described?

It is important to clearly describe treatment or intervention procedures as other clinicians will be reading the paper and therefore may enable clear understanding of the treatment protocol. The report should describe the treatment/intervention protocol in detail; for e.g. in pharmacological management of dental anxiety - the type of drug, route of administration, drug dosage and frequency, and any side effects.

6. Was the post-intervention clinical condition clearly described?

A good case report should clearly describe the clinical condition post-intervention in terms of the presence or lack thereof symptoms. The outcomes of management/treatment when presented as images or figures would help in conveying the information to the reader/clinician.

7. Were adverse events (harms) or unanticipated events identified and described?

With any treatment/intervention/drug, there are bound to be some adverse events and in some cases, they may be severe. It is important that adverse events are clearly documented and described, particularly when a new or unique condition is being treated or when a new drug or treatment is used. In addition, unanticipated events, if any that may yield new or useful information should be identified and clearly described.

8. Does the case report provide takeaway lessons?

Case reports should summarize key lessons learned from a case in terms of the background of the condition/disease and clinical practice guidance for clinicians when presented with similar cases.

References:

Gagnier JJ, Kienle G, Altman DG, Moher D, Sox H, Riley D, CARE Group. The CARE Guidelines: ConsensusBased Clinical Case Reporting Guideline Development. Headache: The Journal of Head and Face Pain, 2013;53(10):1541-1547.

For peer review only

Appendix 7.5 Critical appraisal checklist for analytical cross-sectional studies

JBI Critical Appraisal Checklist for Analytical Cross Sectional Studies

Reviewer______________________________________Date________________________
____

-

-

Author ____Year__________Record Number ________

	Yes	No	Unclear	Not applicable
1. Were the criteria for inclusion in the sample clearly defined?				
1. Were the study subjects and the setting described in detail?				
1. Was the exposure measured in a valid and reliable way?				
1. Were objective, standard criteria used for measurement of the condition?				
1. Were confounding factors identified?				
1. Were strategies to deal with confounding factors stated?				
1. Were the outcomes measured in a valid and reliable way?				
1. Was appropriate statistical analysis used?				

Overall appraisal: Include Exclude Seek further info

Comments (Including reason for exclusion)

____
____

____
____

____
____

Explanation of analytical cross sectional studies critical appraisal

For peer review only

How to cite: Moola S, Munn Z, Tufanaru C, Aromataris E, Sears K, Sfetcu R, Currie M, Lisy K, Qureshi R, Mattis P, Mu P. Chapter 7: Systematic reviews of etiology and risk. In: Aromataris E, Munn Z (Editors). *JBI Manual for Evidence Synthesis.* JBI, 2020. Available from https://synthesismanual.jbi.global. https://doi.org/10.46658/JBIMES-20-08

Analytical cross sectional studies Critical Appraisal Tool

Answers: Yes, No, Unclear or Not/Applicable

1. Were the criteria for inclusion in the sample clearly defined?

The authors should provide clear inclusion and exclusion criteria that they developed prior to recruitment of the study participants. The inclusion/exclusion criteria should be specified (e.g., risk, stage of disease progression) with sufficient detail and all the necessary information critical to the study.

2. Were the study subjects and the setting described in detail?

The study sample should be described in sufficient detail so that other researchers can determine if it is comparable to the population of interest to them. The authors should provide a clear description of the population from which the study participants were selected or recruited, including demographics, location, and time period.

3. Was the exposure measured in a valid and reliable way?

The study should clearly describe the method of measurement of exposure. Assessing validity requires that a 'gold standard' is available to which the measure can be compared. The validity of exposure measurement usually relates to whether a current measure is appropriate or whether a measure of past exposure is needed.

Reliability refers to the processes included in an epidemiological study to check repeatability of measurements of the exposures. These usually include intra-observer reliability and inter-observer reliability.

4. Were objective, standard criteria used for measurement of the condition?

It is useful to determine if patients were included in the study based on either a specified diagnosis or definition. This is more likely to decrease the risk of bias. Characteristics are another useful approach to matching groups, and studies that did not use specified diagnostic methods or definitions should provide evidence on matching by key characteristics.

5. Were confounding factors identified?

Confounding has occurred where the estimated intervention exposure effect is biased by the presence of some difference between the comparison groups (apart from the exposure investigated/of interest). Typical confounders include baseline characteristics, prognostic factors, or concomitant exposures (e.g. smoking). A confounder is a difference between the comparison groups and it influences the direction of the study results. A high quality study at the level of cohort design will identify the potential confounders and measure them (where possible). This is difficult for studies where behavioral, attitudinal or lifestyle factors may impact on the results.

6. Were strategies to deal with confounding factors stated?

Strategies to deal with effects of confounding factors may be dealt within the study design or in data analysis. By matching or stratifying sampling of participants, effects of confounding factors can be adjusted for. When dealing with adjustment in data analysis, assess the statistics used in the study. Most will be some form of multivariate regression analysis to account for the confounding factors measured.

7. Were the outcomes measured in a valid and reliable way?

Read the methods section of the paper. If for e.g. lung cancer is assessed based on existing definitions or diagnostic criteria, then the answer to this question is likely to be yes. If lung cancer is assessed using observer reported, or self-reported scales, the risk of over- or under-reporting is increased, and objectivity is compromised. Importantly, determine if the measurement tools used were validated instruments as this has a significant impact on outcome assessment validity.

Having established the objectivity of the outcome measurement (e.g. lung cancer) instrument, it's important to establish how the measurement was conducted. Were those involved in collecting data trained or educated in the use of the instrument/s? (e.g. radiographers). If there was more than one data collector, were they similar in terms of level of education, clinical or research experience, or level of responsibility in the piece of research being appraised?

8. Was appropriate statistical analysis used?

For peer review only

As with any consideration of statistical analysis, consideration should be given to whether there was a more appropriate alternate statistical method that could have been used. The methods section should be detailed enough for reviewers to identify which analytical techniques were used (in particular, regression or stratification) and how specific confounders were measured.

For studies utilizing regression analysis, it is useful to identify if the study identified which variables were included and how they related to the outcome. If stratification was the analytical approach used, were the strata of analysis defined by the specified variables? Additionally, it is also important to assess the appropriateness of the analytical strategy in terms of the assumptions associated with the approach as differing methods of analysis are based on differing assumptions about the data and how it will respond.

For peer review only

Chapter 8: Mixed methods systematic reviews

Lucylynn Lizarondo[1], Cindy Stern[1], Judith Carrier, Christina Godfrey, Kendra Rieger, Susan Salmond, Joao Apostolo, Pamela Kirkpatrick, Heather Loveday

[1] Co-first authors

How to cite:

Lizarondo L, Stern C, Carrier J, Godfrey C, Rieger K, Salmond S, Apostolo J, Kirkpatrick P, Loveday H. Chapter 8: Mixed methods systematic reviews. In: Aromataris E, Munn Z (Editors). *JBI Manual for Evidence Synthesis.* JBI, 2020. Available from https://synthesismanual.jbi.global. https://doi.org/10.46658/JBIMES-20-09

Chapter 8: Contents

- 8.1 Introduction to mixed methods systematic reviews
- 8.2 Concepts and considerations for mixed methods systematic reviews
- 8.3 The JBI approach to mixed method systematic reviews
- 8.4 Developing a mixed methods review protocol
  - 8.4.1 MMSR questions that take a CONVERGENT INTEGRATED approach to synthesis and integration
  - 8.4.2 MMSR questions that take a CONVERGENT SEGREGATED approach to synthesis and integration
- 8.5 Conducting and reporting a JBI MMSR
  - 8.5.1 Mixed methods systematic review using a CONVERGENT INTEGRATED approach to synthesis and integration
  - 8.5.2 Mixed methods systematic review using a CONVERGENT SEGREGATED approach to synthesis and integration
- Chapter References
- Appendix 8.1 JBI Mixed Methods Data Extraction Form following a Convergent Integrated Approach

For peer review only

8.1 Introduction to mixed methods systematic reviews

Decision-makers who use systematic reviews increasingly argue for a more comprehensive synthesis of the evidence than that currently offered by single method reviews (Dixon-Woods et al., 2005) This is particularly evident in the areas of public health and social policy that deal with complex interventions. A range of methodologies are available that incorporate multiple study designs/types of data including integrative literature reviews (which can include both empirical and theoretical studies with limited formal methods on combining data) (Broome, 2000), comprehensive literature reviews/systematic reviews (where no formal combination or integration of data is undertaken) and mixed methods reviews (where data is combined and integrated together in a more formalized manner). Systematic reviews aim to provide unbiased syntheses of studies/evidence using rigorous and transparent methods as opposed to literature reviews that are largely subjective and unreproducible. Mixed methods systematic reviews (MMSR) can bring together the findings of effectiveness (quantitative evidence) and patient, family, staff or other's experience (qualitative evidence) to enhance their usefulness to decision-makers (Bressan et al., 2016). In addition to this movement for MMSR, there is an increasing focus on the different types of information that guideline developers need when making a decision, such as feasibility, priority, cost effectiveness, impact on equity, acceptability (Alonso-Coello et al., 2016) and patient values and preferences (Zhang et al., 2018). Integrating data in response to these different types of questions into a single synthesis would be incredibly useful for guideline development groups and decision makers.

Systematic reviews addressing questions of experience, (qualitative) and effectiveness (quantitative) have specific purposes but increasingly both perspectives are required to inform clinical, policy or organizational decisions. For example, although quantitative evidence suggests that the use of larval therapy is both clinically effective and cost effective for the debridement of wounds (Adela, 2017; Arabloo et al., 2016; Sun et al., 2014; Tian et al., 2013; Wilasrusmee et al., 2014), evidence from qualitative studies indicates that negative experiences and perceptions impact on the acceptability of the therapy. Some studies indicate feelings of distaste and disgust associated with maggots influence patients' decisions to reject the therapy or impact negatively on their experience of the therapy (McCaughan et al., 2015; Menon, 2012).

Mixed method systematic review methodology is an emerging field of enquiry; MMSR are also referred to as mixed methods research syntheses (Heyvaert et al., 2013), mixed studies reviews (Pluye & Hong, 2014) and mixed research syntheses (Sandelowski et al., 2006). While there is a degree of complexity in conducting MMSR, the core intention is to combine quantitative and qualitative data (from primary studies) or integrate quantitative evidence and qualitative evidence to create a breadth and depth of understanding that can confirm or dispute evidence and ultimately answer the review question/s posed.

Mixed methods reviews represent an important development for individuals engaged in evidence synthesis for healthcare as they attempt to increase the usefulness of their findings and the ability of those findings to inform policy and practice. Similarly, Sandelowski et al. 2013 suggest that the methodological inclusiveness characteristic of MMSR is particularly relevant to international organizations as this broad conceptualization of evidence increases accessibility and utility by a wider range of end users.

Through the development of a well-structured MMSR, the numerical data inherent in the positivist paradigm can support or endorse the equally important opinions and perspectives presented in interpretive and critical paradigms and vice versa. This has the potential to produce more informative conclusions than those derived from evidence presented in autonomous modes of synthesis, i.e. effectiveness systematic reviews and experiential systematic reviews.

Dependent on the nature of the review question (discussed in more depth in Section 8.3) MMSRs allow for:

- an examination of the degree of agreement between quantitative and qualitative data to validate or triangulate results/findings,
- identification of discrepancies within the available evidence,
- determination of whether the quantitative and qualitative data address different aspects of a phenomenon of interest, and
- one type of data that can explore, contextualize or explain the findings of the other type of data.

Although MMSR are gaining traction among healthcare professionals due to their usefulness and practicality, guidance regarding the methodology of combining quantitative and qualitative data is limited and largely at the theoretical stage (Hong et al., 2017).

For peer review only

8.2 Concepts and considerations for mixed methods systematic reviews

The universal steps involved in a systematic review (e.g. formulation of review question/s, establishing eligibility criteria, development of a search strategy, searching and retrieval of relevant studies, critical appraisal of included studies, data extraction, and synthesis) also apply to a MMSR. However, unique aspects regarding how data is combined and the overall integration of the evidence are additional factors that need to be considered.

To avoid confusion in describing a MMSR, it is important to firstly outline a number of core concepts related to this type of systematic review (Table 8.1). A review of the literature conducted by the authors informed the development of core concepts and the subsequent JBI MMSR approach that is detailed in Section 8.3.

Table 8.1: Summary of concepts related to MMSR

**Data**	Refers to the primary data obtained from quantitative studies, qualitative studies or mixed methods studies.
**Data transformation**	Refers to the process of transforming qualitative data into a quantitative format (‘quantitizing’) or quantitative data into a qualitative format (‘qualitizing’).
**Integration**	Refers to the combining of quantitative data with qualitative data following transformation OR of combining quantitative evidence and qualitative evidence without transformation.
**Synthesis**	Can either be a quantitative synthesis or a qualitative synthesis.<br><br>Quantitative synthesis refers to the process of combining extracted data from quantitative studies (including data from the quantitative component of a mixed methods study), resulting in the generation of quantitative evidence.<br><br>Qualitative synthesis refers to the process of combining extracted data from qualitative studies (including data from the qualitative component of a mixed methods study), resulting in the generation of qualitative evidence.
**Sequence of synthesis**	Refers to whether the quantitative synthesis and qualitative synthesis occurs *simultaneously* (i.e. convergent) or *consecutively* (i.e. sequential, where the results /findings from a synthesis of one type of evidence inform the synthesis of the other type of evidence).

A systematic review examining the different methods available to synthesize quantitative and qualitative data or integrate quantitative and qualitative evidence was undertaken by Hong et al 2017. The review included 459 reviews utilizing a number of different frameworks for integration; however, it identified two predominant frameworks to MMSR: the convergent design (where syntheses occur at the same time) and the sequential design (where syntheses occur one after another). The two frameworks identified in Hong et al.’s (2017) review concur with the seminal work undertaken by Sandelowski and colleagues (2006) who developed three basic designs for MMSR which were adapted from the primary mixed methods literature. They include the: (1) integrated design, (2) segregated design and (3) contingent design (Sandelowski et al., 2006).

1. The integrated design involves integration of transformed data referred to as *direct assimilation*, which rests on the assumption that quantitative and qualitative data can both address the same research question. As such they can be combined once data have been transformed in the same format (i.e. ‘quantitized’ or ‘qualitized’) (Sandelowski et al., 2006).
2. The segregated design involves integration of evidence through a method referred to as *configuration,* which is the arrangement of complementary evidence into a line of argument. Complementarity rests on the assumption that quantitative and qualitative evidence address different research questions that are related to the same phenomenon of interest. In other words, quantitative and qualitative evidence address different aspects or dimensions of a phenomenon of interest and therefore they can neither confirm nor refute each other but rather only complement each other. As such, the quantitative evidence and qualitative evidence cannot be directly combined and can only be organized into a coherent whole (Sandelowski et al., 2006).
3. The contingent design takes a cyclic approach in which synthesis is conducted in order to answer questions raised from the previous synthesis i.e. the results of each synthesis determines the next question to undertake until a comprehensive research synthesis that addresses the reviewers objectives is complete (Sandelowski et al., 2006). Table 8.2 provides a comparison of these frameworks.

Table 8.2: Comparison of frameworks identified by Hong et al. (2017) and Sandelowski et al. (2006).

For peer review only - http://bmjopen.bmj.com/site/about/guidelines.xhtml

Hong et al. (2017)	What is involved?	Sandelowski et al. (2006)
**Convergent data-based**	• Typically involves a broad review question that can be addressed by both quantitative studies and qualitative studies<br>• **Requires data transformation**<br>• Involves integration of transformed data	**Integrated**
**Convergent results-based: results are presented in the results section of the systematic review**<br><br>**Convergent parallel-results: results are presented in the discussion section of the systematic review**	• Typically involves an overall review question with sub-questions (some that can only be addressed by quantitative studies and others that can only be addressed by qualitative studies)<br>• Separate and simultaneous synthesis of quantitative data and qualitative data<br>• Involves integration of quantitative evidence and qualitative evidence<br>• **No data transformation**	**Segregated**
**Sequential**	• Synthesis of quantitative data and qualitative data are conducted sequentially based on results from the previous synthesis	**Contingent**

The three main considerations in undertaking an MMSR relate to:

1. the sequence in which the synthesis occurs,
2. how data is transformed, and
3. how transformed data or quantitative and qualitative evidence are integrated together.

Sequence of synthesis

As described above, the order of synthesis can be either convergent or sequential. The convergent design is the dominant approach used in MMSR (95% of reviews), with the sequential design only applied in a very small proportion of reviews (5%) (Hong et al., 2017). Consequently, this current MMSR guidance will focus exclusively on convergent approaches.

In the convergent approach the synthesis occurs simultaneously. This can occur at two different stages within the review; dependent on the type of convergent design utilized. In the first instance, synthesis occurs at the data level when quantitative, qualitative and mixed methods studies are extracted concurrently, data is transformed and then analyzed in a parallel manner.

In the second instance, quantitative evidence (from quantitative studies and data from the quantitative component of mixed methods studies) is synthesized separately as is qualitative evidence (from qualitative studies and data from the qualitative component of mixed methods studies) which are then integrated together.

Data transformation

In order for qualitative and quantitative data to be integrated and fully inform the topic, one approach is for the data to be transformed into a mutually compatible format (Voils et al., 2009). Data transformation can occur either by converting qualitative data into quantitative data (i.e. quantitizing) or by converting quantitative data into qualitative data (i.e. qualitizing). Quantitizing is a process in which qualitative data are assigned numerical values. Approaches described in the literature include content analysis, Bayesian analysis and Boolean analysis (Frantzen & Fetters, 2016). Qualitizing refers to quantitative data being converted into themes, categories, typologies or narratives (Frantzen & Fetters, 2016; Heyvaert et al., 2013; Sandelowski et al., 2006). This can be undertaken by thematic analysis, critical interpretative synthesis, meta-narrative synthesis and realist synthesis (Frantzen & Fetters, 2016). Both quantizing and qualitizing approaches are accepted in the literature; however, one is not recommended over the other with both having their strengths and weaknesses.

Integration of findings

For peer review only - http://bmjopen.bmj.com/site/about/guidelines.xhtml

Integration refers to how transformed data are merged or how quantitative and qualitative evidence are combined. The literature indicates there are various methods for undertaking integration; some of these are described below

A. Integration following data transformation

Quantitative approach: this type of integration is applied when qualitative data are quantitized. Commonly used approaches include content analysis and vote counting.

- In content analysis, themes or categories are developed *a priori* (i.e. before integration) and then all extracted data (i.e. quantitative data and quantitized qualitative data) are coded according to these categories or themes (Dixon-Woods, Agarwal, Jones, Young, & Sutton, 2005; Spilsbury et al., 2008). This is followed by creating tabulations of frequency counts to identify key findings (Dixon-Woods et al., 2005; Spilsbury et al., 2008).
- Vote counting involves two steps: first, the findings of the included studies are classified into those that yield positive results, those that yield negative results, and those that show no difference (i.e. not positive and not negative); second, the number of primary studies allocated to each classification are counted (Hayvaert et al., 2017; Hong et al., 2017). The classification which has the most number of counts is declared the 'winning category' and therefore provides the most convincing evidence according to the vote-counting approach (Hayvaert et al., 2017; Hong et al., 2017).

Qualitative approach: this type of integration is applied when quantitative data are qualitized; to date, the most common approach to such integration is thematic synthesis. In thematic synthesis, extracted data are coded, followed by grouping of codes which then make up a specific theme (Thomas & Harden, 2008). The descriptive themes might then lead to a conceptual framework. In some instances, a theoretical or conceptual framework is used to develop *a priori* set of themes on which to organize the codes identified from the analysis of extracted data.

B. Integration following quantitative and qualitative synthesis

Methods that are often used for integrating a quantitative evidence synthesis with a qualitative evidence synthesis are realist synthesis, narrative summary, thematic synthesis or framework synthesis.

- Realist synthesis is a theory-driven approach aimed at unpacking how an intervention works in a particular context or setting – '*what works for whom in what circumstances'* (Pawson et al., 2005).
- Narrative summary varies from a *'simple recounting and description of findings to more reflective accounts that include commentary and higher levels of abstraction to explain complex processes'* (Hayvaert et al., 2017) p.231.
- Thematic synthesis uses coding, groups similar codes and develops descriptive themes to generate an overall summary of findings (Hong et al., 2017; Thomas & Harden, 2008).
- Framework synthesis involves a preliminary identification of themes against which to map and configure the findings from the quantitative and qualitative studies (Carroll et al., 2011).

A summary of the methodological approaches for MMSR is provided in Table 8.3.

Table 8.3: Summary of methodological approaches for MMSR

Review design	Description	What is involved in the integration?	Methods for integration
Converg ent Integrat ed	Involves data transformation that allows reviewers to combine quantitative and qualitative data	Direct assimilation	• Content analysis<br>• Vote counting<br>• Thematic synthesis
Converg ent Segrega ted	Independent synthesis of quantitative data and qualitative data followed by the integration of the two types of evidence	Configuration	• Realist synthesis<br>• Narrative summary<br>• Thematic synthesis<br>• Framework synthesis
Sequent ial	Synthesis of one type of data occurs after, or is informed by, the synthesis of the other type of data	Direct assimilation or configuration or both	Integration of quantitative evidence and qualitative evidence may or may not occur

For peer review only

8.3 The JBI approach to mixed method systematic reviews

The JBI methodology for mixed methods systematic reviews aligns with the typology developed by Hong et al. 2017. That is, that the review approach can either be convergent (where the synthesis occurs simultaneously) or sequential (where the synthesis occurs consecutively). However, based on minimal usage of the sequential approach, this guidance for JBI mixed methods systematic reviews currently focuses exclusively on the convergent approach. The convergent design can be broken down into a series of methods that have been simplified into two groups – convergent integrated (involves data transformation that allows reviewers to combine quantitative and qualitative data) and convergent segregated (involves independent synthesis of quantitative data and qualitative data leading to the generation of quantitative evidence and qualitative evidence which are then integrated together). The nature/type of question(s) that is (are) posed in the systematic review dictates the approach the reviewer should follow for the synthesis.

Nature of the question

The reviewer needs to consider if the review question can be addressed by both quantitative and qualitative studies or if the focus of the review is on different aspects or dimensions of a particular phenomenon of interest. Here are two scenarios highlighting the different question(s) a reviewer may pose for a mixed methods systematic review.

Scenario 1

Consider the following question:

'What are the barriers and enablers to self-management in adolescents with asthma?' (Holley et al., 2017)

- Here the focus is on barriers and enablers, which can be addressed through qualitative research (e.g. through a phenomenological study of adolescents with asthma) as well as quantitative research (e.g. through a survey of adolescents with asthma conducted as part of a cross sectional study).

Scenario 2

Consider the following questions:

'What is the impact of mindfulness-based interventions on nurses?' and 'What do nurses perceive the benefits and challenges of mindfulness-based interventions to be?'

(Guillaumie, Boiral, & Champagne, 2017)

- Here both questions relate to a common phenomenon i.e. mindfulness-based interventions for nurses but they are addressing two different aspects associated with it – namely what impact these interventions have on nurses in terms of the effect of the interventions on outcomes such as stress and anxiety, and how nurses experience or perceive them. We know that questions of effectiveness are answered through quantitative research (e.g. through a randomized controlled trial comparing mindfulness-based interventions with standard interventions) and questions of experience/perception are answered through qualitative research (e.g. through an ethnographic study where the researcher undertakes fieldwork on a group of nurses receiving mindfulness-based interventions).

For peer review only - http://bmjopen.bmj.com/site/about/guidelines.xhtml

Following question development, the steps involved in quantitative and qualitative systematic reviews apply to mixed methods systematic reviews, such as development of eligibility criteria, literature searching and retrieval, critical appraisal and data extraction (please see Chapter 2 and Chapter 3 of the JBI Manual for Evidence Synthesis for further information). Hence, the guidance described in this section will focus on synthesis and the distinct features of a mixed method systematic review, that is, the integration of quantitative and qualitative evidence, and the transformation of quantitative and qualitative data. Ultimately which approach is utilized will depend on the nature of the question(s) posed, as outlined above.

Approaches to synthesis and integration

If the review question can be addressed by both quantitative and qualitative research designs the **conver gent integrated approach** should be followed; if the focus of the review is on different aspects or dimensions of a particular phenomenon of interest the **convergent segregated approach** is undertaken. Let's now take another look at our two examples to explain why.

Scenario 1

'What are the barriers and enablers to self-management in adolescents with asthma?' (Holley et al., 2017)

- Here the focus is on barriers and enablers, which can be addressed through qualitative research (e.g. through a phenomenological study of adolescents with asthma) as well as quantitative research (e.g. through a survey of adolescents with asthma conducted as part of a cross sectional study).

- Since this review question can be answered by both quantitative AND qualitative studies it would follow a **convergent integrated approach** to its synthesis and integration.

Scenario 2

What is the impact of mindfulness-based interventions on nurses?' and 'what do nurses perceive the benefits and challenges of mindfulness-based interventions to be?'

(Guillaumie et al., 2017)

- Here both questions relate to a common phenomenon i.e. mindfulness-based interventions for nurses but they are addressing two different aspects associated with it – namely what impact these interventions have on nurses in terms of the effect of the interventions on outcomes such as stress and anxiety and how nurses experience or perceive them. We know that questions of effectiveness are answered through quantitative research (e.g. through a randomized controlled trial comparing mindfulness-based interventions with standard interventions) and questions of experience/perception are answered through qualitative research (e.g. through an ethnographic study where the researcher undertakes fieldwork on a group of nurses receiving mindfulness-based interventions).

- Since this review focuses on different dimensions of a phenomenon it would follow a **convergent segregated approach** to its synthesis and integration.

For peer review only

The **convergent integrated approach, suggested for Scenario 1 above,** refers to a process of combining extracted data from quantitative studies (including data from the quantitative component of mixed methods studies) and qualitative studies (including data from the qualitative component of mixed methods studies), and involves data transformation. It is recommended that quantitative data be 'qualitized', as codifying quantitative data is less error-prone than attributing numerical values to qualitative data (The Joanna Briggs Institute, 2014). 'Qualitizing' involves extracting data from quantitative studies and translating or converting it into 'textual descriptions' to allow integration with qualitative data. 'Qualitizing' involves a narrative interpretation of the quantitative results.

At the simplest level, qualitized data might comprise describing a sample (or members of it) using word categories based on supplementary descriptive statistics such as average or percentage scores (Bazeley, 2012). The study by Cohen et al. 2003 (part of the review by Holley et al. 2017 outlined in Scenario 1 above) aimed to examine the perceptions of adolescents with asthma and their attitudes towards self-treatment. Qualitization identified: *29% of survey participants reported feeling embarrassed having an asthma attack while with friends* (Cohen et al., 2003). Qualitized data can also include profiling of the sample using cluster or factor analysis (Bazeley, 2012). Data with a temporal or longitudinal component (Bazeley, 2012), or those that examine associations and relationships using inferential statistics such as linear or logistic regression analysis also have narrative potential and can therefore be qualitized by identifying variables included in the analysis. For example the study by Kyngäs (2000) (also in Holley et al., 2017) identified factors that predict compliance with health regimens by adolescents with asthma using logistic regression. Transformation identified: *support from nurses as a significant factor in predicting compliance with health regimens by adolescents with asthma (OR =56.87, 95% 17.15-88.58)*. By qualitizing, the reviewer converts the 'quantities' into declarative stand-alone sentences, in a way that answers the review question.

These textual descriptions are then assembled and pooled with the qualitative data extracted directly from qualitative studies. Similar to the meta-aggregative approach for JBI qualitative reviews, reviewers are required to then undertake repeated, detailed examination of the assembled data to identify categories on the basis of similarity in meaning. A category will integrate two or more: qualitative data, 'qualitized' data or a combination of both. In some instances however, data may not have the same meaning as others and therefore cannot be combined to form a category. Where possible, categories are then aggregated to produce the overall finding(s) of the review. This process is illustrated in Figure 8.1.

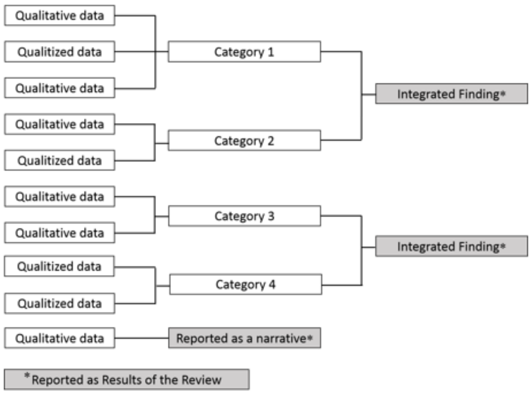

Figure 8.1: Convergent Integrated Approach. Qualitized findings are assembled into categories with qualitative findings extricated directly from qualitative studies based on similarity of meaning.

Using the example outlined above (Scenario 1), reviewers were able to determine six key barriers and/or enablers regarding self-management of asthma, which related to knowledge, lifestyle, beliefs and attitudes, relationships, intrapersonal characteristics and communication (Holley et al., 2017).

For peer review only - http://bmjopen.bmj.com/site/about/guidelines.xhtml

The **convergent segregated approach** consists of conducting a separate quantitative synthesis and qualitative synthesis, followed by integration of the results derived from each of the syntheses. By integrating the quantitative and qualitative synthesized findings, we are able to have a greater depth of understanding of the phenomena of interest compared to undertaking two separate component syntheses without formally linking the two sets of evidence. In Scenario 2 above, quantitative data is synthesized in the form of a meta-analysis (or a narrative summary if meta-analysis is not possible) to determine the effects of mindfulness-based interventions on nurses. Additionally, all the qualitative data is pooled (in the case of the JBI approach, through the process of meta-aggregation or narrative summary if deemed inappropriate) – refer to Chapter 2 of the JBI Manual for Evidence Synthesis for further information) to determine the experiences/perceptions of nurses receiving these interventions. There is no order to which synthesis is done first as they are independent; however, both must be completed before moving onto the next step, integration of quantitative evidence and qualitative evidence. This next step involves juxtaposing the synthesized quantitative results with the synthesized qualitative findings and organizing or linking the results and findings into a line or argument to produce an overall 'configured analysis.' This is where the reviewer considers how (and if) the results and findings complement each other by using one type of evidence to explore, contextualize or explain the findings of the other type of evidence. In this step, results and findings cannot be reduced but are organized into a coherent whole (Sandelowski, Voils, & Barroso, 2006). In this approach, the reviewer repeatedly compares the results of the quantitative synthesis with the findings of the qualitative synthesis, analyzing the intervention which had been investigated for effectiveness (quantitative) in light of the experiences of the participants (qualitative). The following questions act as a guide for this process:

- Are the results/findings from individual synthesis supportive or contradictory?
- Does the qualitative evidence explain why the intervention is or is not effective?
- Does the qualitative evidence help explain differences in the direction and size of effect across the included quantitative studies?
- Which aspects of the quantitative evidence are/are not explored in the qualitative studies?
- Which aspects of the qualitative evidence are/are not tested in the quantitative evidence?

In some instances, the reviewer may find that the results of quantitative studies are not complementary or have no relationship with the findings of the qualitative studies, or vice-versa. In some cases the reviewer may identify gaps where further research may be useful to explain the contradictory findings or when there is no relationship between the qualitative findings and quantitative findings.

In Scenario 2 (mindfulness-based interventions for nurses), results from statistical meta-analysis showed significant reductions in anxiety and depression following treatment, whereas the qualitative synthesis highlighted improvements in areas such as well-being and work performance. In this example the qualitative synthesis highlighted factors not considered or covered in the quantitative synthesis which led to stronger support of the intervention as well as recommendations for future research (Guillaumie et al., 2017).

This integration follows a formal, structured process which is reported in the results section of the review (i.e. it "marries" the results of separate syntheses). The JBI Framework for undertaking a mixed methods systematic reviewis outlined in Figure 8.2.

Regardless of the approach taken, the ability to undertake a mixed methods synthesis and integration will ultimately depend on the evidence located and subsequently included in the review. As in a quantitative review focussing on a question of effectiveness where the aim is to be able to conduct a meta-analysis (or similarly a meta-aggregation in a qualitative review), in a mixed methods systematic review there may not be sufficient evidence available, the data may be limited in its 'richness' or thickness of description or the evidence located may not be similar enough to combine or link together. In these situations, the authors may need to undertake a narrative synthesis instead, much like in a quantitative review when a meta-analysis is not possible.

The JBI System for the Unified Management, Assessment and Review of Information (JBI SUMARI) supports reviewer's to undertake a mixed methods systematic review using both the convergent integrated and the convergent segregated approaches.

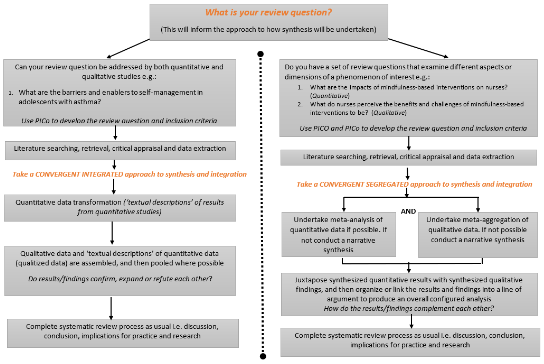

Figure 8.2: The JBI Framework for mixed methods systematic reviews

For peer review only - http://bmjopen.bmj.com/site/about/guidelines.xhtml

For peer review only

8.4 Developing a mixed methods review protocol

This section outlines the components of a MMSR protocol and provides guidance on the information that each section should address. Specifically, it provides guidance on each of the following components: title, introduction, review question(s), inclusion criteria, methods (search strategy, study selection, assessment of methodological quality, data extraction, data synthesis), references, and appendices.

As discussed in Section 8.3, JBI focuses exclusively on the convergent approach to mixed methods reviews and as such the nature of the question(s) posed dictates the approach reviewers take with their synthesis. While the main steps undertaken in a systematic review are universal, there are some elements between the two approaches the MMSR that will differ. For this reason, the following section is divided into the two approaches. Reviewers will need to be clear on the type of question(s) (and subsequently the type of synthesis) their proposed review is attempting to answer and follow the corresponding guidance provided below.

- 8.4.1 MMSR questions that take a CONVERGENT INTEGRATED approach to synthesis and integration
- 8.4.2 MMSR questions that take a CONVERGENT SEGREGATED approach to synthesis and integration

For peer review only

8.4.1 MMSR questions that take a CONVERGENT INTEGRATED approach to synthesis and integration

MMSR questions that take a CONVERGENT INTEGRATED approach to synthesis and integration

If the review question(s) can be addressed by both quantitative and qualitative studies, an integrated approach to synthesis and integration is undertaken. In this approach quantitative and qualitative data are synthesized/combined together through data transformation.

Protocol development

Commonly a review following this approach comprises one review question and primarily lends itself to the PICo criteria, where P is the population of interest, I is the Phenomena of interest and Co is the Context. However, where a review question does not fit the PICo approach, reviewers may consider using a different framework (e.g. PICO) to structure their question. The guidance for protocol development provided in Chapter 2 (Systematic reviews of qualitative evidence) of this online reviewer's manual can be followed however some additional considerations are needed for a MMSR and these are detailed below.

Title of a MMSR protocol

The title should be informative and give clear indication of the topic and population of the MMSR. Titles should not be phrased as questions and there should be congruency between the title, review question (s) and inclusion criteria. The title should always include the phrase "…: a mixed methods systematic review protocol" to allow easy identification of the type of document it represents. An example title may be:

Barriers and facilitators to asthma self-management in adolescents: a mixed methods systematic review protocol

Abstract

This section is a summary of the protocol in 300 words. The following headings should be included in the abstract - Objective, Introduction, Inclusion Criteria, Methods, Systematic review registration number (if applicable) and Keywords. The abstract should not contain abbreviations or references.

Introduction

As with all JBI systematic review protocols, the introduction to a MMSR should describe and situate the topic of interest under review. Definitions can assist to provide clarity. Explanation of how the review question can be answered by both quantitative and qualitative studies is required as is an explanation on how the review will add to the evidence base or inform clinical practice.

Additionally, a statement that a preliminary search of databases (with databases listed) has been undertaken and no existing or ongoing mixed method or individual systematic reviews on the topic have been identified should be provided. If other systematic reviews on the topic exist, indication on how the proposed systematic review will differ should be detailed. Finally, the introduction should conclude with an overarching review objective that captures and aligns with the core elements/mnemonic (i.e. PICo) of the inclusion criteria. The introduction should be of sufficient length to discuss all of the elements of the proposed plan for the review; usually all the relevant information may be provided in approximately 1000 words. This section should be written in simple prose for non-expert readers.

Review question(s)

Clarity in the review question(s) assists in developing a protocol and also ultimately, the conduct of the review. The review question(s) guide and direct the development of the specific review inclusion criteria and facilitate more effective searching, and provide a structure for the development of the full review. There should also be consistency between the review title and the review question(s). Typically for a MMSR that follows a convergent integrated approach to synthesis a broad review question is posed that can be addressed by both quantitative studies and qualitative studies. As such PICo should be used to develop the review question as well as the inclusion criteria. An example of a PICo question that may be posed by a MMSR is:

1. What are the barriers and facilitators to self-management in adolescents with asthma?

For peer review only - http://bmjopen.bmj.com/site/about/guidelines.xhtml

In the above example, adolescents with asthma (i.e. those managing their own asthma), healthcare professionals (i.e. those involved in supporting adolescents to self-manage their asthma) and policy makers (i.e. those that assist in deciding how asthma is managed at a population level) are the target audiences since the intention is to determine how adolescents with asthma can best manage their asthma.

Inclusion criteria

This section of the protocol details the basis on which studies will be considered for inclusion into the systematic review and should be as clear and unambiguous as possible. Inclusion criteria should be reasonable, sound and justified. These criteria will be used in the selection process, when it is decided if a study will be included or not in the review.

Population

There needs to be a clear and direct link between the review question, title and the participant characteristics in the inclusion criteria. This section should specify the details about the types of participants considered for the review. Consider what are the most important characteristics of the population? (e.g., age, disease/condition, severity of illness, setting, gender, ethnicity etc.).

For example:

This review will consider studies that include #describe population#

Phenomena of interest

A phenomenon of interest is the experience, event or process occurring that is under study. The level of detail ascribed to the phenomena may vary with the nature or complexity of the topic.

This review will consider studies that investigate #insert text#

Context

Context will vary depending on the question(s) of the review. Context may include, but is not limited to consideration of: cultural or sub-cultural factors, geographic location, specific racial or gender based interests, or detail about the specific setting (such as acute care, primary health care, or the community).

For example:

This review will consider studies that investigate #insert text#

Types of studies

This section should include the relevant information related to both quantitative and qualitative studies. The time frame chosen for the search should be justified and any language restrictions stated. For example:

This review will consider quantitative, qualitative and mixed methods studies. Quantitative studies will include #insert text#. Qualitative studies will include #insert text#. Mixed method studies will only be considered if data from the quantitative or qualitative components can be clearly extracted.

Studies published in #insert language(s)# will be included. Studies published from #database inception/or insert date# to the present will be included as #justify date range#

There should be a match in this section between the methodology of the primary research studies to be considered for the review and the review question.

Methods

Reference to the JBI methodology for MMSR should be provided. Additionally if the review title has been registered, the name of the registry (e.g. PROSPERO) and the registration number should be reported below the Methods heading. For example:

The proposed systematic review will be conducted in accordance with the JBI methodology for MMSR #insert a citation to the Chapter in the JBI Manual for Evidence Synthesis # **Note**: if the review title has been registered, report the name of the registry (e.g. PROSPERO) and the registration number.

Search strategy

For peer review only

This section of a review protocol should provide explicit and clear information regarding two different aspects of locating studies: all information sources that will be searched for the review, and the strategies used for searching. The aim of a systematic review is to identify all relevant studies, published or not, on a given topic. Searching should be based on the principle of comprehensiveness, with the widest reasonable collection of information sources that are considered appropriate to the review.

The databases to be searched must be listed, including the search platform used where necessary, along with a completed search strategy for one major database which should be presented as an Appendix.

This section is universal for example:

The search strategy will aim to locate both published and unpublished studies. An initial limited search of #MEDLINE and CINAHL #change as appropriate# was undertaken to identify articles on the topic. The text words contained in the titles and abstracts of relevant articles, and the index terms used to describe the articles were used to develop a full search strategy for #report the name of the relevant database# (see Appendix #). The search strategy, including all identified keywords and index terms will be adapted for each included information source. The reference list of all studies selected for critical appraisal will be screened for additional studies.

Information sources

This section is universal for example:

The databases to be searched include: #insert text#

The search for unpublished studies and gray literature will include: #insert text#

Study selection

This section should describe the process of reviewing the results of the search to see if they meet inclusion criteria and subsequently deciding which of the papers are to be retrieved.

This section is universal for example:

Following the search, all identified citations will be loaded into #insert the name of the bibliographic software or citation management system e.g. EndNote version/year (Clarivate Analytics, PA, USA)# and duplicates removed. Titles and abstracts will then be screened by two independent reviewers for assessment against the inclusion criteria for the review. Potentially relevant studies will be retrieved in full and their citation details imported into the JBI System for the Unified Management, Assessment and Review of Information (JBI SUMARI; JBI, Adelaide, Australia). The full text of selected citations will be assessed in detail against the inclusion criteria by two independent reviewers. Reasons for exclusion of full text studies that do not meet the inclusion criteria will be recorded and reported in the systematic review. Any disagreements that arise between the reviewers at each stage of the study selection process will be resolved through discussion, or with a third reviewer. The results of the search will be reported in full in the final review and presented in a Preferred Reporting Items for Systematic Reviews and Meta-analyses (PRISMA) flow diagram. #Insert reference to the PRISMA statement#

Assessment of methodological quality

This section should describe the critical appraisal process and instruments that will be used in the review process and the procedures for solving disagreements between reviewers.

Studies that are eligible for inclusion in the review must be assessed for methodological quality. The decision as to whether or not to include a study can be made based on meeting a pre-determined proportion of all criteria, or on certain criteria being met. It is also possible to weight certain criteria differently. Decisions about a scoring system or any cut-off for exclusion should be made in advance and agreed upon by all reviewers before critical appraisal commences.

All included studies need to be critically appraised using the standard JBI critical appraisal instruments (qualitative instrument available in Appendix 2.1 and quantitative instruments available in Appendices 3.1-3.4). For mixed methods studies the relevant JBI qualitative and quantitative tools can be used. The source of the JBI critical appraisal tool should be cited in the protocol.

The recommended set text should therefore include the relevant information related to both quantitative and qualitative studies, for example:

Quantitative studies (and quantitative component of mixed methods studies) selected for retrieval will be assessed by two independent reviewers for methodological validity prior to inclusion in the review using standardized critical appraisal instruments from JBI SUMARI. #Insert reference to appraisal tools#

Qualitative studies (and qualitative component of mixed methods studies) selected for retrieval will be assessed by two independent reviewers for methodological validity prior to inclusion in the review using the standardized critical appraisal instrument from JBI SUMARI (The Joanna Briggs Institute et al., 2017).

For peer review only

Authors of papers will be contacted to request missing or additional data for clarification, where required. Any disagreements that arise between the reviewers will be resolved through discussion, or with a third reviewer. The results of critical appraisal will be reported in narrative form and in a table.

Choose from one of the following two options:

- All studies, regardless of the results of their methodological quality, will undergo data extraction and synthesis (where possible). #Indicate how the results of the critical appraisal will be incorporated into the review#.
- Following critical appraisal, studies that do not meet a certain quality threshold will be excluded. This decision will be based on #list the decision rules#.

Data extraction

This section of the review protocol should specify the data extraction process and instruments that will be used in the review process, as well as the procedures for solving disagreements between reviewers.

For a MMSR that follows a convergent integrated approach, this section should specify what information from the quantitative and qualitative studies will be considered as constituting the findings.

- Quantitative studies typically include descriptive, or analytic studies that provide information about magnitude and statistical significance.
  - For descriptive studies, the extracted data might comprise an average or a percentage that profiles the sample or members of it.
  - For analytic studies, where the study examines a relationship between variables, data extraction should include ALL relationships RELEVANT to the review question, that is, both significant and non-significant results. Variables/outcomes not reaching statistical significance are important to report, as they may validate or highlight inconsistencies in the literature when integrated and pooled with other quantitative or qualitative findings.
- For qualitative studies, themes or subthemes relevant to the review question are extracted and supported with illustrations (i.e. a direct quotation from a participant, an observation or other supporting data from the paper) to preserve the context of the findings. Each finding should then be assigned a level of credibility based on the congruency of the finding with supporting data. There are three levels of credibility:
  - Unequivocal - relates to evidence beyond reasonable doubt which may include conclusions that are matter of fact, directly reported/observed and not open to challenge
  - Credible - relates to those conclusions that are, albeit interpretations, plausible in light of the data and theoretical framework.
  - Not Supported - is when the findings are not supported by the data

*'Not Supported' data are not included in the synthesis of data.

Example text that can be reported in this section is as follows:

Quantitative and qualitative data will be extracted from studies included in the review by two independent reviewers using the standardized JBI data extraction tool in JBI SUMARI (tool provided at Appendix 8.1) #modify if other software or processes will be used for your review#. #Cite the tool to be used or append the data extraction tool if an existing tool has been modified or a new tool developed. Any modifications to existing tools should be described in the text#. The data extracted will include specific details about the populations, study methods, phenomena of interest, context and outcomes of relevance to the review question(s). Specifically, quantitative data will comprise of data-based outcomes of descriptive and/or inferential statistical tests. In addition, qualitative data will comprise of themes or subthemes with corresponding illustrations, and will be assigned a level of credibility.

Any disagreements that arise between the reviewers will be resolved through discussion, or with a third reviewer. Authors of papers will be contacted to request missing or additional data, where required.

Data transformation

Following extraction, quantitative data are then transformed into qualitized data. This section of the review protocol should describe how the extracted quantitative data are converted into qualitized data to facilitate integration with data extracted from qualitative studies (and qualitative component of mixed methods studies). For example:

The quantitative data will then be converted into ‘qualitized data’. This will involve transformation into textual descriptions or narrative interpretation of the quantitative results so as to respond directly to the review question.

Data synthesis and integration

This section should describe how the data will be combined and reported in the systematic review. For a MMSR that follows a convergent integrated approach this should include the relevant information related to how qualitized data and data from qualitative studies will be integrated, for example:

For peer review only

This review will follow a convergent integrated approach according to the JBI methodology for mixed methods systematic reviews using JBI SUMARI. #Insert a citation to the methodology#. This will involve assembling the qualitized data with the qualitative data. Assembled data are categorized and pooled together based on similarity in meaning to produce a set of integrated findings in the form of line of action statements.

Please note: Due to the complexities associated with recommendations being derived from both streams of evidence and the impact of data transformation and/or integration on the grading process, an assessment of the certainty of the evidence using either the GRADE or ConQual approach is currently not recommended for JBI MMSR following either the integrated or segregated approach and requires further investigation.

Conflicts of interest and acknowledgements

Details of requirements in these sections are described in Section 1.6 of this Manual.

Conflicts of interest

A statement which either declares the absence of any conflicts of interest or which describes a specified or potential conflict of interest should be made by the reviewers in this section.

Acknowledgements

Any acknowledgements should be made in this section e.g. sources of external funding or the contribution of colleagues or institutions. It should also be noted if the systematic review contributes towards a degree award.

For peer review only

8.4.2 MMSR questions that take a CONVERGENT SEGREGATED approach to synthesis and integration

The convergent segregated approach adopted by JBI maintains a clear distinction between quantitative and qualitative evidence and requires individual syntheses to be conducted prior to the final integration of quantitative evidence and qualitative evidence.

Protocol development

Protocol development addresses both PICO and PICo criteria and is commonly comprised of separate review questions. As such the guidance for protocol development provided in Chapter 2 (Systematic reviews of qualitative evidence) and Chapter 3 (Systematic reviews of effectiveness) of this online reviewer's manual should be followed. Some additional considerations are needed for a MMSR and these are detailed below.

Title of a MMSR protocol

The title should be informative and give clear indication of the topic and population of the MMSR. Titles should not be phrased as questions and there should be congruency between the title, review question (s) and inclusion criteria. The title should always include the phrase "…: a mixed methods systematic review protocol" to allow easy identification of the type of document it represents. An example title may be:

Mindfulness-based interventions for nurses: a mixed methods systematic review protocol

Abstract

This section is a summary of the protocol in 300 words. The following headings should be included in the abstract - Objective, Introduction, Inclusion Criteria, Methods, Systematic review registration number (if applicable) and Keywords. The abstract should not contain abbreviations or references.

Introduction

As with all JBI systematic review protocols, the introduction to a MMSR should describe and situate the topic of interest under review. Definitions can assist to provide clarity. Where complex or multifaceted phenomena are being described, it may be important to detail the whole of the phenomenon for an international readership. Justification for the need to examine both quantitative and qualitative evidence in a single review is required as is an explanation on how the review will add to the evidence base or inform clinical practice.

Additionally, a statement that a preliminary search of databases (with databases listed) has been undertaken and no existing or ongoing mixed method or individual systematic reviews on the topic have been identified should be provided. If other systematic reviews on the topic exist, indication on how the proposed systematic review will differ should be detailed. Finally, the introduction should conclude with an overarching review objective that captures and aligns with the core elements/mnemonic (i.e. PICO /PICo) of the inclusion criteria. The introduction should be of sufficient length to discuss all of the elements of the proposed plan for the review; usually all the relevant information may be provided in approximately 1000 words. This section should be written in simple prose for non-expert readers.

Review question(s)

Clarity in the review questions assist in developing a protocol and also ultimately, the conduct of the review. The review question(s) guide and direct the development of the specific review criteria and facilitate more effective searching, and provides a structure for the development of the full review. There should also be consistency between the review title and the review questions.

For a MMSR that takes a convergent segregated approach to synthesis, the review question(s) should focus on different aspects or dimensions of a particular phenomenon of interest and will pose questions that specifically require the inclusion of two or more syntheses that are grounded in different approaches.

As such PICO and PICo mnemonics should be used to develop the review questions as well as the inclusion criteria. Examples of clearly articulated PICO/PICo questions that may be posed by a MMSR are:

1. What is the impact of mindfulness-based interventions on nurses?
2. What do nurses perceive the benefits and challenges of mindfulness-based interventions to be?

For peer review only

The overarching aim of a MMSR is to produce a final integrated synthesis incorporating quantitative and qualitative evidence that informs conclusions and recommendations for clinical practice and policy decision making. In the above example, healthcare professionals and policy makers involved in delivering and planning such interventions are the target audience since the intention is to determine effective and positively experienced interventions for nurses.

Inclusion criteria

This section of the protocol details the basis on which studies will be considered for inclusion into the systematic review and should be as clear and unambiguous as possible. Inclusion criteria should be reasonable, sound and justified. These criteria will be used in the selection process, when it is decided if a study will be included or not in the review.

Population

There needs to be a clear and direct link between the review question, title and the participant characteristics in the inclusion criteria. This section should specify the details about the types of participants considered for the review. Consider what are the most important characteristics of the population? (e.g., age, disease/condition, severity of illness, setting, gender, ethnicity etc.).

For a MMSR that follows a convergent segregated approach this section is universal (i.e. the population should be the same for both the quantitative and qualitative questions) for example:

The review will consider studies that include #describe population#

Intervention

Details about the intervention of interest should be specified, for example, the nature of intervention, frequency, intensity, timing, and details about those administering the intervention. The same kind of information should be specified for all comparators considered in the review. Where possible, the intervention should be described in detail, particularly if it is multifaceted.

The quantitative component of the review will consider studies that evaluate #insert text#.

Phenomena of interest

The qualitative component of this review will consider studies that investigate #insert text#

A phenomenon of interest is the experience, event or process occurring that is under study. The level of detail ascribed to the phenomena may vary with the nature or complexity of the topic. There should be congruence between the intervention and phenomena of interest.

Outcomes

This should address the quantitative component only, for example:

The quantitative component of this review will consider studies that include the following outcome measures: #insert text#

Outcomes should be measurable and appropriate to the review question(s). The relevance of each outcome to the review question(s) should be justified in the introduction section. Both beneficial outcomes and harms should be considered. The appropriateness of the number and scope of outcomes depend on the specifics of the review question(s).

Context

This should address the qualitative component only, for example:

The qualitative component of this review will consider studies that investigate #insert text#

Context will vary depending on the question(s) of the review. Context may include, but is not limited to consideration of: cultural or sub-cultural factors, geographic location, specific racial or gender based interests, or detail about the specific setting (such as acute care, primary health care, or the community).

Types of studies

This should address each of the syntheses included in the review. The time frame chosen for the search should be justified and any language restrictions stated. For example:

This review will consider quantitative, qualitative and mixed methods studies. Quantitative studies will include #insert text#. Qualitative studies will include #insert text#. Mixed method studies will only be considered if data from the quantitative or qualitative components can be clearly extracted.

For peer review only

Studies published in #insert language(s)# will be included. Studies published from #database inception/or insert date# to the present will be included as #justify date range#

There should be a match in this section between the methodology of the primary research studies to be considered for the review and the review question.

Methods

Reference to the JBI methodology for MMSR should be provided. Additionally, if the review title has been registered, the name of the registry (e.g. PROSPERO) and the registration number should be reported below the Methods heading. For example:

The proposed systematic review will be conducted in accordance with the JBI methodology for mixed methods systematic reviews #insert a citation to the Chapter in the JBI Manual for Evidence Synthesis #
Note: if the review title has been registered, report the name of the registry (e.g. PROSPERO) and the registration number.

Search strategy

This section of a review protocol should provide explicit and clear information regarding two different aspects of locating studies: all information sources that will be searched for the review, and the strategies used for searching. The aim of a systematic review is to identify all relevant studies, published or not, on a given topic. Searching should be based on the principle of comprehensiveness, with the widest reasonable collection of information sources that are considered appropriate to the review.

The databases to be searched must be listed, including the search platform used where necessary, along with a completed search strategy for one major databases which should be presented as an Appendix.

This section is universal, for example:

The search strategy will aim to locate both published and unpublished studies. An initial limited search of #MEDLINE and CINAHL #change as appropriate# was undertaken to identify articles on the topic. The text words contained in the titles and abstracts of relevant articles, and the index terms used to describe the articles were used to develop a full search strategy for #report the name of the relevant database# (see Appendix #). The search strategy, including all identified keywords and index terms will be adapted for each included information source. The reference list of all studies selected for critical appraisal will be screened for additional studies.

Depending on the review questions that are posed, authors may find that it is appropriate to search for all forms of evidence simultaneously with the one search strategy or they may develop separate search strategies for the different review questions. This decision will need to be made by the reviewers and consideration should be given to the review questions posed, the amount of literature available in the topic area and the searching expertise of the reviewers.

Information sources

This section is universal for example:

The databases to be searched include: #insert text#

The search for unpublished studies and gray literature will include: #insert text#

Where databases/registries/sources are specific to a particular design, the reviewers should clearly indicate such e.g.:

Cochrane Central Register of Controlled Trials (For quantitative studies only)

Study selection

This section should describe the process of reviewing the results of the search to see if they meet inclusion criteria and subsequently deciding which of the papers are to be retrieved.

For a MMSR that follows a convergent segregated approach this section is universal for example:

For peer review only

Following the search, all identified citations will be loaded into #insert the name of the bibliographic software or citation management system e.g. EndNote version/year (Clarivate Analytics, PA, USA)# and duplicates removed. Titles and abstracts will then be screened by two independent reviewers for assessment against the inclusion criteria for the review. Potentially relevant studies will be retrieved in full and their citation details imported into JBI's System for the Unified Management, Assessment and Review of Information (JBI SUMARI). The full text of selected citations will be assessed in detail against the inclusion criteria by two independent reviewers. Reasons for exclusion of full text studies that do not meet the inclusion criteria will be recorded and reported in the systematic review. Any disagreements that arise between the reviewers at each stage of the study selection process will be resolved through discussion, or with a third reviewer. The results of the search will be reported in full in the final report and presented in a Preferred Reporting Items for Systematic Reviews and Meta-analyses (PRISMA) flow diagram. #Insert reference to the PRISMA statement#

Assessment of methodological quality

This section should describe the critical appraisal process and instruments that will be used in the review process and the procedures for solving disagreements between reviewers.

Studies that are eligible for inclusion in the review must be assessed for methodological quality. The decision as to whether or not to include a study can be made based on meeting a pre-determined proportion of all criteria, or on certain criteria being met. It is also possible to weight certain criteria differently. Decisions about a scoring system or any cut-off for exclusion should be made in advance and agreed upon by all reviewers before critical appraisal commences.

All included studies need to be critically appraised using the standard JBI critical appraisal instruments (qualitative instrument available in Appendix 2.1 and quantitative instruments available in Appendices 3.1-3.4). The source of the JBI critical appraisal tool should be cited in the protocol.

The recommended set text should therefore address each of the syntheses included in the review, for example:

Quantitative studies (and quantitative component of mixed methods studies) selected for retrieval will be assessed by two independent reviewers for methodological validity prior to inclusion in the review using standardized critical appraisal instruments from JBI SUMARI. #Insert reference to appraisal tools#

Qualitative studies (and qualitative component of mixed methods studies) selected for retrieval will be assessed by two independent reviewers for methodological validity prior to inclusion in the review using the standardized critical appraisal instrument from JBI SUMARI (The Joanna Briggs Institute et al., 2017).

Authors of papers will be contacted to request missing or additional data for clarification, where required. Any disagreements that arise between the reviewers will be resolved through discussion, or with a third reviewer. The results of critical appraisal will be reported in narrative form and in a table.

Choose from one of the following two options:

- All studies, regardless of the results of their methodological quality, will undergo data extraction and synthesis (where possible). #Indicate how the results of the critical appraisal will be incorporated into the review#.
- Following critical appraisal, studies that do not meet a certain quality threshold will be excluded. This decision will be based on #list the decision rules#.

Data extraction

This section of the review protocol should specify the data extraction process and instruments that will be used in the review process, as well as the procedures for solving disagreements between reviewers.

For a MMSR that follows a convergent segregated approach this should address each of the syntheses included in the review, for example:

For the quantitative component, data will be extracted from quantitative and mixed methods (quantitative component only) studies included in the review by two independent reviewers using the standardized JBI data extraction tool in JBI SUMARI #modify if other software or processes will be used for your review#. #Cite the tool to be used or append the data extraction tool if an existing tool has been modified or a new tool developed# Any modifications to existing tools should be described in the text# The data extracted will include specific details about the populations, study methods, interventions, and outcomes of significance to the review objective.

For the qualitative component, data will be extracted from qualitative and mixed methods (qualitative component only) studies included in the review by two independent reviewers using the standardized JBI data extraction tool in JBI SUMARI #modify if other software or processes will be used for your review#. #Cite the tool to be used or append the data extraction tool if an existing tool has been modified or a new tool developed. Any modifications to existing tools should be described in the text#. The data extracted will include specific details about the population, context, culture, geographical location, study methods and the phenomena of interest relevant to the review objective. Findings, and their illustrations will be extracted and assigned a level of credibility.

For peer review only - http://bmjopen.bmj.com/site/about/guidelines.xhtml

Any disagreements that arise between the reviewers will be resolved through discussion, or with a third reviewer. Authors of papers will be contacted to request missing or additional data, where required.

Data synthesis and integration

This section should describe how the data will be synthesized, combined and reported in the systematic review. For a MMSR that follows a convergent segregated approach this should address the quantitative synthesis and the qualitative synthesis as well as how they will be integrated in the final synthesis, for example:

This review will follow a convergent segregated approach to synthesis and integration according to the JBI methodology for mixed methods systematic reviews using JBI SUMARI. #Insert a citation to the methodology#. This will involve separate quantitative and qualitative synthesis followed by integration of the resultant quantitative evidence and qualitative evidence.

Quantitative synthesis

Data will, where possible, be pooled with statistical meta-analysis using JBI SUMARI. Effect sizes will be expressed as either odds ratios (for dichotomous data) or weighted (or standardized) final post-intervention mean differences (for continuous data) and their 95% confidence intervals will be calculated for analysis #modify as appropriate#. Heterogeneity will be assessed statistically using the standard chi squared and I^2 tests. Statistical analyses will be performed using #insert model (random or fixed effects) #. #Cite the Tufanaru et al study# Subgroup analyses will be conducted where there is sufficient data to investigate #add text as appropriate#. Sensitivity analyses will be conducted to test decisions made regarding #add text as appropriate#. Where statistical pooling is not possible the findings will be presented in narrative form including tables and figures to aid in data presentation, where appropriate. A funnel plot will be generated #state software to use# to assess publication bias if there are 10 or more studies included in a meta-analysis#. Statistical tests for funnel plot asymmetry (Egger test, Begg test, Harbord test) will be performed where appropriate.

Qualitative synthesis

Qualitative research findings will, where possible be pooled using JBI SUMARI with the meta-aggregation approach. #Insert a citation to the methodology#. This will involve the aggregation or synthesis of findings to generate a set of statements that represent that aggregation, through assembling the findings and categorizing these findings based on similarity in meaning. These categories are then subjected to a synthesis to produce a comprehensive set of synthesized findings that can be used as a basis for evidence-based practice. Where textual pooling is not possible the findings will be presented in narrative form.

Integration of quantitative evidence and qualitative evidence

The findings of each single method synthesis included in this review will then be configured according to the JBI methodology for mixed methods systematic reviews. #Insert a citation to the methodology# This will involve quantitative evidence and qualitative evidence being juxtaposed and organized/linked into a line of argument to produce an overall configured analysis. Where configuration is not possible the findings will be presented in narrative form.

Please note: Due to the complexities associated with recommendations being derived from both streams of evidence and the impact of data transformation and/or integration on the grading process, an assessment of the certainty of the evidence using either the GRADE or ConQual approach is currently not recommended for JBI MMSR following either the integrated or segregated approach and requires further investigation.

Conflicts of interest and acknowledgements

Details of requirements in these sections are described in Section 1.6 of this Manual.

Conflicts of interest

A statement which either declares the absence of any conflicts of interest or which describes a specified or potential conflict of interest should be made by the reviewers in this section.

Acknowledgements

Any acknowledgements should be made in this section e.g. sources of external funding or the contribution of colleagues or institutions. It should also be noted if the systematic review contributes towards a degree award.

For peer review only

8.5 Conducting and reporting a JBI MMSR

This section provides further guidance on components that should be included in the final JBI MMSR, and information regarding each component as found in JBI SUMARI. The content of the sections of the review protocol (Section 8.3) and the review report are conceptually the same, particularly the introduction and the methods section. The review protocol specified the proposed plan for the review; the review reports on what was actually performed and the results of the review undertaken.

Please refer to publication criteria for the *JBI Evidence Synthesis* for specific submission requirements for systematic reviews.

As in Section 8.4, the following section is divided into the two approaches. Reviewers should follow the appropriate guidance provided below.

- 8.5.1 Mixed methods systematic review using a CONVERGENT INTEGRATED approach to synthesis and integration
- 8.5.2 Mixed methods systematic review using a CONVERGENT SEGREGATED approach to synthesis and integration

For peer review only

8.5.1 Mixed methods systematic review using a CONVERGENT INTEGRATED approach to synthesis and integration

Title of a mixed methods systematic review

The title should be informative and give clear indication of the topic of the MMSR. The title should always include the phrase "…: a mixed methods systematic review" to allow easy identification of the type of document it represents. An example title may be:

Barriers and facilitators to asthma self-management in adolescents: a mixed methods systematic review

Abstract

This section is a summary of the review in 500 words, stating the objective, methods, main findings and principal conclusions of the review. The abstract should not contain abbreviations or references.

The following headings should be included in the abstract.

Objective: State an overarching review objective structured using the key components of the inclusion criteria (approximately one to two sentences).

Introduction: Briefly describe what is already known on the topic and what this review will add to the evidence base (approximately two to three sentences).

Inclusion criteria: Summarize the inclusion criteria as it relates to the type of review being conducted. Present the information in one or two sentences – **NOT** under individual subheadings.

Methods: List the key information sources searched (those that provided the majority of included studies), any limits placed on the scope of the search (e.g. language), and the date range, or the date of the last search. State the recommended JBI approach to MMSR was followed e.g. study selection, critical appraisal, data extraction and data synthesis and integration. The method of synthesis and integration should be clearly reported (convergent integrated). Otherwise, briefly describe any notable deviations to the methodological approach taken (e.g. criteria used to exclude studies on the basis of methodological quality etc.).

Results: The bulk of the abstract should be reserved to convey the main results of the review.

As a general rule, report the number and type of included studies, and any pertinent study characteristics. Summarize the overall quality of the included studies.

Report the findings obtained from the integration of 'qualitized' data and qualitative data.

Conclusions: Provide a conclusion based on a general interpretation of the results considering, for example, the methodological quality of the included studies and any limitations of the review. Briefly convey key implications for practice and/or research.

Introduction

As with all JBI systematic reviews, the introduction to a MMSR should describe and situate the topic of interest under review. Definitions can assist to provide clarity. Where complex or multifaceted phenomena are being described, it may be important to detail the whole of the phenomenon for an international readership. Explanation of how the review question can be answered by both quantitative and qualitative studies is required as is an explanation on how the review will add to the evidence base or inform clinical practice.

Additionally a statement that a preliminary search of databases (with databases listed) has been undertaken and no existing or ongoing mixed method or systematic reviews on the topic have been identified should be provided. If other systematic reviews on the topic exist, indication on how the proposed systematic review differed should be detailed. Finally the introduction should conclude with an overarching review objective that captures and aligns with the core elements of the inclusion criteria. The introduction should be approximately 1000 words.

Review question(s)

The review question(s) should be explicitly stated in unambiguous terms. See Section 8.4 of this Chapter for further information regarding the question(s) of a MMSR.

For peer review only - http://bmjopen.bmj.com/site/about/guidelines.xhtml

Inclusion criteria

This section of the review details the basis on which studies were considered for inclusion in the systematic review and should be as clear and unambiguous as possible. Inclusion criteria should be reasonable, sound and justified and address the elements in the PICo question(s).

Population

This section should specify the details about types of participants considered for the review (e.g., age, disease/condition, severity of illness, setting, gender, ethnicity etc.). This section is universal for example:

The review considered studies that included #describe population#

Phenomena of Interest

A phenomenon of interest is the experience, event or process occurring that is under study. The level of detail ascribed to the phenomena may vary with the nature or complexity of the topic.

Like the protocol, details about the phenomena of interest should be adequately described. For example:

This review considered studies that investigated #insert text#

Context

Context may include, but is not limited to consideration of: cultural or sub-cultural factors, geographic location, specific racial or gender based interests, or detail about the specific setting (such as acute care, primary health care, or the community). Like the protocol, details regarding the context should be provided. For example:

This review considered studies that investigated #insert text#

Types of studies

This section should include the relevant information related to quantitative, qualitative and mixed methods studies. For example:

This review considered quantitative, qualitative and mixed methods studies. Quantitative studies included #insert text#. Qualitative studies included #insert text#. Mixed method studies where data from the quantitative or qualitative components could be clearly extracted were also considered.

There should be a congruence in this section between the methodology of the primary research studies that were considered for the review and the review question(s).

Methods

This section of the review is reserved for the methods used to conduct the review and should be presented under the relevant subheadings, including any deviations from the method outlined in the *a priori* protocol and a rationale.

Directly below the Methods heading provide the following information:

- State and appropriately cite the JBI methodology that was employed in the conduct of the review and synthesis.
- Refer to and cite the *a priori*protocol that was published, or accepted for publication (e.g. 'in press'), in the *JBI Evidence Synthesis*.
- If the protocol was registered with PROSPERO, provide registration information including registration number (e.g. PROSPERO CRD42015425226).

Search strategy

The search strategy section of a review should provide explicit and clear information regarding all information sources that were used in the review, and the actual strategies used for searching. The review should provide details regarding all information sources that were used in the review: electronic bibliographic databases (including the search platform used); gray literature sources; relevant journals; websites of relevant organizations; etc. The review should specify the timeframe for the search, the date of last search for each database, and any language and date restrictions, with appropriate justifications. For example:

For peer review only - http://bmjopen.bmj.com/site/about/guidelines.xhtml

The search strategy aimed to find both published and unpublished studies. A three-step search strategy was utilized in this review. First an initial limited search of #MEDLINE and CINAHL# change as appropriate# was undertaken followed by analysis of the text words contained in the title and abstract and the index terms used to describe the articles. The search strategy, including all identified keywords and index terms was adapted for each included information source and a second search was undertaken on #insert month and date searched#. The full search strategies are provided in Appendix #. Finally the reference list of all studies selected for critical appraisal will be screened for additional studies.

Information sources

The databases that were searched included: #insert databases with platforms as appropriate#.

Sources of unpublished studies and gray literature included #insert text, e.g. trial registers etc.#

Study selection

The review report should describe the actual process of study screening for all stages of selection (e.g. title and abstract examination; full text examination) and the actual procedures used for solving disagreements between reviewers. For example:

Following the search, all identified citations were collated and uploaded into #insert bibliographic software or citation management system (e.g. EndNote version/year (Clarivate Analytics, PA, USA))# and duplicates removed. Titles and abstracts were then screened by two reviewers for assessment against the inclusion criteria for the review. Studies that met the inclusion criteria were retrieved in full and their details imported into the JBI System for the unified Management Assessment and Review of Information package (JBI SUMARI). The full text of selected studies were retrieved and assessed in detail against the inclusion criteria by two independent reviewers. Full text studies that did not meet the inclusion criteria were excluded and reasons for exclusion are provided in #insert Appendix number#. Any disagreements that arose between the reviewers were resolved through discussion (OR There were no disagreements that arose between reviewers).

Assessment of methodological quality

The review should specify the critical appraisal process and instruments that were used in the review process and the procedures for solving disagreements between reviewers. The details of the decision processes and criteria used for exclusion of studies based on the results of critical appraisal should be explicitly provided. All details about the scoring systems and the cut-off scores (if applicable) for inclusion of studies in the review should be described and justified. For example:

Eligible studies were critically appraised by two independent reviewers for methodological quality using the #insert names of tools used and cite them.# Authors of papers were contacted to request missing or additional data for clarification, where required. Any disagreements that arose between the reviewers were resolved through discussion, or with a third reviewer.

Indicate what constituted acceptable levels of information for a study to receive a positive, negative or unclear response to a critical appraisal question and if applicable, the rationale and criteria for excluding studies on the basis of methodological quality.

Data extraction

The review should specify the data extraction process and instruments that were used in the review process and the procedures for solving disagreements between reviewers. As outlined in Section 8.4 of this Chapter, for a MMSR that follows a convergent integrated approach it needs to be specified what information from the quantitative and qualitative studies were considered as constituting the findings. For example:

Quantitative and qualitative data were extracted from included studies by two independent reviewers using the standardized JBI data extraction tool. #modify if other software or processes were used for your review#. #Cite the tool used or append the data extraction tool if an existing tool was modified or a new tool developed. Any modifications to existing tools should be described in the text#. The data extracted included specific details about the population, study methods, the phenomenon of interest, context and outcomes of relevance to the review question(s). In addition, qualitative data comprised of themes or subthemes with corresponding illustrations, which were assigned a level of credibility. Any disagreements that arose between the reviewers will be resolved through discussion, or with a third reviewer. Authors of #insert number of studies# were contacted for missing information or additional data.

Data transformation

The review should specify the data transformation process that was used to convert the extracted quantitative data into qualitized data to facilitate integration with data extracted from qualitative studies (and the qualitative component of mixed methods studies). For example:

For peer review only - http://bmjopen.bmj.com/site/about/guidelines.xhtml

The quantitative data was converted into 'qualitized data'. This involved transformation into textual descriptions or narrative interpretation of the quantitative results from experimental and observational studies (including the quantitative component of mixed methods studies), in a way that answered the review questions by repeated detailed examination.

Data synthesis and integration

The review should indicate that a convergent integrated approach was applied. The review should detail how the reviewers analyzed and integrated the data extracted from included quantitative, qualitative and mixed methods studies and detail the aggregative approach to integration. For example:

The convergent integrated approach according to the JBI methodology for mixed methods systematic review using JBI SUMARI was used in this review. #Insert a citation to the methodology#. This involved assembling the 'qualitized' data with the qualitative data. Assembled data were categorized and pooled together based on similarity in meaning to produce a set of integrated findings in the form of line of action statements.

Results

This section of the review has distinct sub-sections describing the study inclusion, the methodological quality of included studies, detailed characteristics and description of the included studies and, importantly, the findings of data transformation and the integration processes.

Study inclusion

This section should allow the reader to clearly follow how the included studies were identified and selected for inclusion in the review. There should be a narrative description of the process accompanied by a PRISMA flowchart; details to be reported include narrative summary of the numbers of studies identified, numbers screened, studies selected for retrieval and included/excluded and their reasons for exclusion, numbers appraised and included/excluded, and numbers included in the review.

Ideally, a list of all excluded studies, excluded after full text examination and after critical appraisal, with the explicit reasons for exclusion, should be provided in appendices to the review. As a minimum, at least the list of studies excluded after critical appraisal and the reasons for exclusion should be reported. If no studies were excluded after critical appraisal then the list of all studies excluded after full text examination including the explicit reasons for exclusion, should be provided in appendices to the review.

Methodological quality

This section should focus on methodological quality as determined by the relevant critical appraisal instrument. There should be a separate narrative summary for the overall methodological quality of the quantitative (and quantitative component of mixed methods studies) and qualitative studies (and qualitative component of mixed methods studies), which can be supported by tables showing the results of the critical appraisal (see Tables 8.4 and 8.5 for examples). Please note, not all quantitative study designs are shown below). Where only few studies are identified, or there are specific items of interest from included studies, these should be addressed in the narrative also, particularly where studies were deficient, or particularly good. Use of 'Unclear' and 'Not Applicable' should also be explained in the text.

Table 8.4: Critical appraisal results for included studies using the JBI Critical Appraisal Checklist for Randomized Controlled Trials (and RCT component of mixed methods studies)

Study	Q1	Q2	Q3	Q4	Q5	Q6	Q7	Q8	Q9	Q10	Q11	Q12	Q13
Author(s) ref	Y	Y	Y	N	Y	U	Y	N	Y	U	Y	Y	U
Author(s) ref	Y	N	Y	Y	Y	U	Y	N/A	Y	Y	Y	Y	U

Y - Yes, N - No, U - Unclear, N/A - not applicable

Table 8.5: Critical appraisal results for included studies using the JBI Qualitative Critical Appraisal Checklist (and qualitative component of mixed methods studies)

Study	Q1	Q2	Q3	Q4	Q5	Q6	Q7	Q8	Q9	Q10
Author(s) ref	Y	Y	Y	N	Y	U	Y	N	Y	U
Author(s) ref	Y	N	Y	Y	Y	U	Y	N/A	Y	Y

Y - Yes, N - No, U - Unclear, N/A - not applicable

If modified appraisal tools are not appended to the review, the appraisal questions should be added as a footnote/caption to the tables so readers can clearly interpret the information presented.

Characteristics of included studies

For peer review only

This section of the results should include an overall description of the included studies (with reference to the table of included study characteristics in the appendices), with the main aim to provide some context to the results section and sufficient detail for the reader to confirm that the studies match the eligibility criteria for the review. This includes the descriptive and demographic features (e.g. the country and setting of the study) of the included studies, methodology of included studies, geographic context of included studies, participant characteristics, and phenomena of interest, as they relate to the review question(s) and the inclusion criteria. Specific items/points of interest from individual studies may also be highlighted here and synthesized in a narrative.

Findings of the review

Review findings are structured according to the phenomena of interest and should describe all the identified integrated finding(s), the categories that form them and the underpinning qualitative and/or qualitized data. Integrated findings should be presented with an explanatory statement that conveys the inclusive meaning of a group of similar categories (i.e. line of action statements). This section should also provide a narrative of all the data that cannot be combined to form a category.

A schematic of the synthesis (See Figure 8.1) should constitute part of this section, which must be accompanied by sufficient narrative to explain the categories and integrated findings. *Where textual pooling was not possible the findings should be presented in narrative form.* The suggested structure for reporting findings:

#insert Integrated Finding# (where appropriate)

#Brief explanatory statement#

#insert underpinning Category 1#

#Report the qualitative and/or qualitized data underpinning the category#

#insert underpinning Category 2#

#Report the qualitative and/or qualitized data underpinning the category#

Please note: Due to the complexities associated with recommendations being derived from both streams of evidence and the impact of data transformation and/or integration on the grading process, an assessment of the certainty of the evidence using either the GRADE or ConQual approach is *currently* not recommended for JBI MMSR following either the integrated or segregated approach and requires further investigation.

Discussion

This section should provide a detailed discussion of issues arising from the conduct of the review, as well as a discussion of the findings of the review and of the significance of the review findings in relation to practice and research. The findings should be discussed in the context of current literature, practice and policy. It should also include a narrative discussion of the review results in comparison with other external literature, and against the broad directions established in the introduction of the review. The discussion does not bring in new findings that have not been reported in the results section but does seek to establish a line of argument based on the findings regarding the phenomenon of interest.

Conclusions and Recommendations

This section should include the overall conclusions of the review. The conclusions should provide direct answers to the review question(s). These conclusions should be based only on the results of the review and directly inferred from the review results.

Recommendations for practice

This sub-section of the Conclusion section should include the recommendations for practice inferred from the results of the integration of the 'qualitized' data and qualitative data. Recommendations should be assigned a JBI Grade of Recommendation.

Recommendations for research

This sub-section of the Conclusion should include the recommendations for future research inferred from the results of the integration of the 'qualitized' data and qualitative data, and issues and problems noted in the review process related to the search, selection of studies, critical appraisal, data extraction, and data synthesis.

For peer review only - http://bmjopen.bmj.com/site/about/guidelines.xhtml

Conflicts and acknowledgements

Details of requirements in these sections are described in Section 1.6 of this Manual.

Conflicts of interest

A statement which either declares the absence of any conflicts of interest or which describes a specified or potential conflict of interest should be made by the reviewers in this section.

Funding

Authors should provide details regarding any sources of funding for the review project. The role of all funders in the review process, if any, should be explicitly described. If the review is funded, then any potential conflicts of interest or intellectual bias of the funders should be specified in the review.

Acknowledgements

Any acknowledgements should be made in this section e.g. sources of external funding or the contribution of colleagues or institutions. It should also be noted if the systematic review contributes towards a degree award.

For peer review only

8.5.2 Mixed methods systematic review using a CONVERGENT SEGREGATED approach to synthesis and integration

Title of a mixed method systematic review

The title should be informative and give clear indication of the topic and population of the MMSR. Titles should not be phrased as questions and there should be congruency between the title, review question (s) and inclusion criteria. The title should always include the phrase "…: a mixed methods systematic review" to allow easy identification of the type of document it represents. An example title may be:

Mindfulness-based interventions for nurses: a mixed methods systematic review

Abstract

This section is a summary of the review in 500 words, stating the objective, methods, main findings and principal conclusions of the review. The abstract should not contain abbreviations or references.

The following headings should be included in the abstract.

Objective: State an overarching review objective structured using the key components of the inclusion criteria (approximately one to two sentences).

Introduction: Briefly describe what is already known on the topic and what this review will add to the evidence base (approximately two to three sentences).

Inclusion criteria: Summarize the inclusion criteria as it relates to the type of review being conducted. Present the information in one or two sentences – **NOT** under individual subheadings.

Methods: List the key information sources searched (those that provided the majority of included studies), any limits placed on the scope of the search (e.g. language), and the date range, or the date of the last search. State the recommended JBI approach to MMSR was followed e.g. study selection, critical appraisal, data extraction and data synthesis and integration. The method of synthesis and integration should be clearly reported (convergent segregated). Otherwise, briefly describe any notable deviations to the methodological approach taken (e.g. criteria used to exclude studies on the basis of methodological quality etc.).

Results: The bulk of the abstract should be reserved to convey the main results of the review. As a general rule, report the number and type of included studies, and any pertinent study characteristics. Summarize the overall quality of the included studies.

Report the results obtained from quantitative synthesis, and the findings from the qualitative synthesis. Key findings from the integration of quantitative evidence and qualitative evidence should also be presented.

Conclusions: Provide a conclusion based on a general interpretation of the results considering, for example, the methodological quality of the included studies and any limitations of the review. Briefly convey key implications for practice and/or research.

Introduction

As with all JBI systematic reviews, the introduction to a MMSR should describe and situate the topic of interest under review. Definitions can assist to provide clarity. Where complex or multifaceted phenomena are described, it may be important to detail the whole of the phenomenon for an international readership. Explanation of how the review question can be answered by both quantitative and qualitative studies is required as is an explanation on how the review will add to the evidence base or inform clinical practice.

Additionally, a statement that a preliminary search of databases (with databases listed) was undertaken and no existing or ongoing mixed method or individual systematic reviews on the topic were identified should be provided. If other systematic reviews on the topic existed, indication on how the proposed systematic review differed should be detailed. Finally the introduction should conclude with an overarching review objective that captures and aligns with the core elements of the inclusion criteria. The introduction should be approximately 1000 words.

Review question(s)

The review question(s) should be explicitly stated in unambiguous terms. See Section 8.4 of this Chapter for further information regarding the question(s) of a MMSR.

For peer review only

Inclusion criteria

This section of the review details the basis on which studies were considered for inclusion in the systematic review and should be as clear and unambiguous as possible. Inclusion criteria should be reasonable, sound and justified and address the elements in the PICO/PICo questions.

Population

This section should specify the details about types of participants considered for the review (e.g., age, disease/condition, severity of illness, setting, gender, ethnicity etc.). This section is universal, for example:

The review considered studies that included #describe population#

Intervention

Details about the intervention of interest should be specified, for example:

The quantitative component of the review considered studies that evaluated #insert text# Information about the comparator(s) should also be detailed here.

Phenomena of interest

The qualitative component of this review considered studies that investigated #insert text#

Like the protocol, details about the phenomena of interest should be adequately described.

Outcomes

This should address the quantitative component only, for example:

The quantitative component of this review considered studies that included the following outcome measures: #insert text#

Like the protocol, all outcomes should be adequately described including how they will be measured.

Context

This should address the qualitative component only, for example:

The qualitative component of this review considered studies that investigated #insert text#

Like the protocol, details regarding the context should be provided.

Types of studies

This should address each of the syntheses included in the review, for example:

This review considered quantitative, qualitative and mixed methods studies. Quantitative studies included #insert text#. Qualitative studies included #insert text#. Mixed method studies were considered if data from the quantitative or qualitative components could be clearly extracted.

There should be a congruence in this section between the methodology of the primary research studies to be considered for the review and the review question(s).

Methods

This section of the review is reserved for the methods used to conduct the review and should be presented under the relevant subheadings, including any deviations from the method outlined in the *a priori* protocol and a rationale.

Directly below the Methods heading provide the following information:

- State and appropriately cite the JBI methodology that was employed in the conduct of the review and synthesis.
- Refer to and cite the *a priori* protocol that was published, or accepted for publication (e.g. 'in press'), in the *JBI Evidence Synthesis*.
- If the protocol was registered with PROSPERO, provide registration information including registration number (e.g. PROSPERO CRD42015425226).

Search strategy

For peer review only - http://bmjopen.bmj.com/site/about/guidelines.xhtml

The search strategy section of a review should provide explicit and clear information regarding all information sources (electronic bibliographic databases; gray literature sources; relevant journals; websites of relevant organizations; etc.) that were used in the review, and the actual strategies used for searching (all should be provided in the appendix). The review should specify the timeframe for the search, the date of the last search for each database, and any language and date restrictions, with appropriate justifications. For example:

The search strategy aimed to find both published and unpublished studies. A three-step search strategy was utilized in this review. First an initial limited search of #MEDLINE and CINAHL# change as appropriate# was undertaken followed by analysis of the text words contained in the title and abstract and the index terms used to describe the articles. The search strategy, including all identified keywords and index terms was adapted for each included information source and a second search was undertaken on #insert month and date searched#. The full search strategies are provided in Appendix #. Finally the reference list of all studies selected for critical appraisal will be screened for additional studies.

Information sources

The databases that were searched included: #insert databases with platforms as appropriate#.

Sources of unpublished studies and gray literature included #insert text, e.g. trial registers etc.#

Where databases/registries/sources were specific to a particular design, the reviewers should clearly indicate such e.g.:

Cochrane Central Register of Controlled Trials (For quantitative studies only)

Study selection

The review should describe the actual process of study screening for all stages of selection (e.g. title and abstract examination; full text examination) and the procedures used for solving disagreements between reviewers. For example:

Following the search, all identified citations were collated and uploaded into #insert bibliographic software or citation management system (e.g. EndNote version/year (Clarivate Analytics, PA, USA)# and duplicates removed. Titles and abstracts were then screened by two reviewers for assessment against the inclusion criteria for the review. Studies that met the inclusion criteria were retrieved in full and their details imported into the JBI System for the Unified Management Assessment and Review of Information package (JBI SUMARI). The full text of selected studies were retrieved and assessed in detail against the inclusion criteria by two independent reviewers. Full text studies that did not meet the inclusion criteria were excluded and reasons for exclusion are provided in #insert Appendix number#. Any disagreements that arose between the reviewers were resolved through discussion (OR There were no disagreements that arose between reviewers).

Assessment of methodological quality

The review should specify the critical appraisal process, the instruments that were used and the procedures for solving disagreements between reviewers. The details of the decision processes and criteria used for exclusion of studies based on the results of critical appraisal should be explicitly provided. All details about the scoring systems and the cut-off scores (if applicable) for inclusion of studies in the review should be described and justified. For example:

Eligible studies were critically appraised by two independent reviewers for methodological quality using the #insert names of tools used and cite them.# Authors of papers were contacted to request missing or additional data for clarification, where required. Any disagreements that arose between the reviewers were resolved through discussion, or with a third reviewer.

Indicate what constituted acceptable levels of information for a study to receive a positive, negative or unclear response to a critical appraisal question and if applicable, the rationale and criteria for excluding studies on the basis of methodological quality.

Data extraction

The review should specify the data extraction process and instruments that were used in the review process and the procedures for solving disagreements between reviewers. For example:

For peer review only

Quantitative and qualitative data were extracted from included studies by two independent reviewers using the relevant JBI data extraction tool. #modify if other software or processes were used for your review#. #Cite the tool used or append the data extraction tool if an existing tool was modified or a new tool developed. Any modifications to existing tools should be described in the text#. For quantitative studies (and the quantitative component of mixed methods studies), data extracted included specific details about the populations, interventions, study methods and outcomes of significance to the review question. For qualitative studies (and the qualitative component of mixed methods studies), data extracted included specific details about the population, context, culture, geographical location, study methods and the phenomenon of interest relevant to the review question. Findings with their corresponding illustrations were also extracted and assigned a level of credibility. Any disagreements that arose between the reviewers will be resolved through discussion, or with a third reviewer. Authors of #insert number of studies# were contacted for missing information or additional data.

Data synthesis and integration

The review should indicate that a convergent segregated approach to synthesis and integration was applied. This section should also indicate the approach used to perform the quantitative synthesis (i.e. meta-analysis and/or narrative synthesis) and the qualitative synthesis (i.e. meta-aggregative or narrative synthesis). See Section 8.4 of this Chapter for further information. The approach to the integration of the quantitative and qualitative evidence should be described in as much detail as is reasonably possible. For example:

Quantitative evidence and qualitative evidence were integrated using configurative analysis. This involved constant comparison of the quantitative evidence and the qualitative evidence, followed by the analysis of interventions, which had been investigated in the quantitative studies, in line with the experiences of participants explored in the qualitative studies in order to organize/link the evidence into a line of argument. Where configuration was not possible the findings are presented in narrative form.

Results

This section of the review has distinct sub-sections describing study inclusion, the methodological quality of included studies, detailed characteristics and description of the included studies and, importantly, the findings of the individual syntheses and results of the integration of the quantitative evidence and qualitative evidence.

Study inclusion

This section should allow the reader to clearly follow how the included studies were identified and selected for inclusion in the review. There should be a narrative description of the process accompanied by a PRISMA flowchart; details to be reported include narrative summary of the numbers of studies identified, numbers screened, studies selected for retrieval and included/excluded and their reasons for exclusion, numbers appraised and included/excluded, and numbers included in the review. This section should report the number of studies which contributed to the quantitative component and the number of studies which contributed to the qualitative component.

Ideally, a list of all excluded studies, excluded after full text examination and after critical appraisal, with the explicit reasons for exclusion, should be provided in appendices to the review. As a minimum, at least the list of studies excluded after critical appraisal and the reasons for exclusion should be reported. If no studies were excluded after critical appraisal then the list of all studies excluded after full text examination including the explicit reasons for exclusion, should be provided in appendices to the review.

Methodological quality

This section should focus on methodological quality as determined by the relevant critical appraisal instrument. There should be a separate narrative summary for the overall methodological quality of the quantitative (and quantitative component of mixed methods studies) and qualitative (and qualitative component of mixed methods studies) studies, which can be supported by tables showing the results of the critical appraisal (see Tables 8.6 and 8.7 for examples). Please note, not all quantitative study designs are shown below). Where only few studies are identified, or there are specific items of interest from included studies, these should be addressed in the narrative also, particularly where studies were deficient, or particularly good. Use of 'Unclear' and 'Not Applicable' should also be explained in the text.

Table 8.6: Critical appraisal results for included studies using the JBI Critical Appraisal Checklist for Randomized Controlled Trials (and RCT component of mixed methods studies)

Study	Q1	Q2	Q3	Q4	Q5	Q6	Q7	Q8	Q9	Q10	Q11	Q12	Q13
Author(s) ref	Y	Y	Y	N	Y	U	Y	N	Y	U	Y	Y	Y
Author(s) ref	Y	N	Y	Y	Y	U	Y	N/A	Y	Y	Y	U	U

Y - Yes, N - No, U - Unclear, N/A - not applicable

Table 8.7: Critical appraisal results for included studies using the JBI Qualitative Critical Appraisal Checklist (and qualitative component of mixed methods studies)

For peer review only - http://bmjopen.bmj.com/site/about/guidelines.xhtml

Study	Q1	Q2	Q3	Q4	Q5	Q6	Q7	Q8	Q9	Q10
Author(s) ref	Y	Y	Y	N	Y	U	Y	N	Y	U
Author(s) ref	Y	N	Y	Y	Y	U	Y	N/A	Y	Y

Y - Yes, N - No, U - Unclear, N/A - not applicable

If modified appraisal tools are not appended to the review, the appraisal questions should be added as a footnote/caption to the tables so readers can clearly interpret the information presented.

Characteristics of included studies

This section of the results should include an overall description of the included studies (with reference to the table of included study characteristics in the appendices), with the main aim to provide some context to the results section and sufficient detail for the reader to confirm that the studies match the eligibility criteria for the review. This includes the descriptive and demographic features (e.g. the country and setting of the study) of the included studies, methodology of included studies, geographic context of included studies and participant characteristics, characteristics of the interventions, and phenomena of interest, as they relate to the review questions and the inclusion criteria. Specific items/points of interest from individual studies may also be highlighted here and synthesized in a narrative.

Findings of the review

Quantitative evidence

This section should be organized in a meaningful way based on the review question(s) and types of interventions and outcomes. This section should provide comprehensive information regarding the results of all performed meta-analyses and additional analyses (e.g. sub-group analysis). Summary results from meta-analyses should be reported as summary point estimates and interval estimates (confidence intervals) with consideration of any heterogeneity present. The meta-analysis forest plots should also be presented in this section. A narrative summary should complement the forest plots and provide additional commentaries and explanations for all performed meta-analyses (Munn, Tufanaru, & Aromataris, 2014).

If meta-analysis is not performed, a narrative summary should be included. The narrative summary should provide an overall summary of the findings of the included studies and their biases, strengths and limitations. Textual commentaries and tables are used in order to summarize the results from the included studies and to provide context information for these results, thus facilitating understanding of the summarized results.

Qualitative evidence

This section should be organized in a meaningful way based on the review question(s). A meta-aggregative schematic should constitute part of this section, which must be accompanied by sufficient narrative to explain the categories and synthesized findings. *Where textual pooling was not possible the findings should be presented in narrative form.*

Findings and illustrations should be located in an appendix, or may be incorporated into the body of the review. There should be a logical and informative presentation of the findings, categories and synthesized findings.

Integration of quantitative evidence and qualitative evidence

This section should provide a narrative summary that represents the configured analysis of the quantitative and qualitative evidence. This should include statements that address ALL of the following questions:

- Are the results/findings from individual syntheses supportive or contradictory?

For example, the quantitative evidence might show improvements in patient outcomes following exposure to the intervention. These results support the qualitative evidence, which might demonstrate patients' perceived benefits from taking part in the intervention. In this example, the quantitative evidence supports the qualitative evidence. In some instances, however, the results/findings from individual syntheses may be conflicting. For example, while the qualitative evidence might describe patients' perceived benefits from the treatment, the quantitative evidence might fail to demonstrate a reduction of patient symptoms following the intervention.

- Does the qualitative evidence explain why the intervention is/is not effective?

For example, findings from the qualitative evidence might reveal that patients perceived the intervention of interest as a pleasant experience and that it contributed to their sense of well-being. This can then be used to explain and support why compliance to the intervention was high and why the majority of patients actively engaged with their health practitioners, which would be useful for explaining the effectiveness of the intervention.

For peer review only

- Does the qualitative evidence explain differences in the direction and size of effect across the included quantitative studies?

For example, results from the quantitative evidence might show differences in the effects of the intervention which might have been explored in the qualitative studies e.g. it is possible that some results in the quantitative evidence are better understood when the results from the qualitative evidence are taken into account?

- Which aspects of the quantitative evidence were/were not explored in the qualitative studies?

For example, the reviewer might indicate that some outcomes measured in the quantitative studies (e.g. health-related quality of life, family relationships, anxiety) were not explored in the qualitative studies and can therefore be investigated in future qualitative studies.

- Which aspects of the qualitative evidence were/were not tested in the quantitative studies?

For example, findings from the qualitative evidence might indicate some perceived positive effects (e.g. improved mood) from the intervention which might not have been measured in the quantitative studies; this would have implications for future trials.

All of the questions above should be answered, however dependent on the evidence included in the review it is acknowledged that some responses will be more detailed than others.

Please note: Due to the complexities associated with recommendations being derived from both streams of evidence and the impact of data transformation and/or integration on the grading process, an assessment of the certainty of the evidence using either the GRADE or ConQual approach is *currently* not recommended for JBI MMSR following either the integrated or segregated approach and requires further investigation.

Discussion

This section should provide a detailed discussion of the findings of the review and of the significance of the review findings in relation to practice and research as well as a discussion of issues arising from the conduct of the review. The findings should be discussed in the context of current literature, practice and policy. It should also include a narrative discussion of the review results in comparison with other external literature, and against the broad directions established in the introduction of the review. The discussion does not bring in new findings that have not been reported in the results section but does seek to establish a line of argument based on the findings regarding the intervention and phenomenon of interest.

Conclusions and Recommendations

This section should include the overall conclusions of the review. The conclusions should provide direct answers to the review question(s). These conclusions should be based only on the results of the review and directly inferred from the review results.

Recommendations for practice

This sub-section of the Conclusions section should include the recommendations for practice inferred from the results of the integration of the quantitative evidence and qualitative evidence. Recommendations should be assigned a JBI Grade of Recommendation.

Recommendations for research

This should include the recommendations for future research inferred from the results of the integration of the quantitative evidence and qualitative evidence, specifically, inferred from the gaps identified during the configurative analysis, and issues and problems noted in the review process related to the search, selection of studies, critical appraisal, data extraction, and data synthesis.

Conflicts and acknowledgements

Details of requirements in these sections are described in Section 1.6 of this Manual.

Conflicts of interest

A statement which either declares the absence of any conflicts of interest or which describes a specified or potential conflict of interest should be made by the reviewers in this section.

Funding

For peer review only - http://bmjopen.bmj.com/site/about/guidelines.xhtml

Authors should provide details regarding any sources of funding for the review project. The role of all funders in the review process, if any, should be explicitly described. If the review is funded, then any potential conflicts of interest or intellectual bias of the funders should be specified in the review.

Acknowledgements

Any acknowledgements should be made in this section e.g. sources of external funding or the contribution of colleagues or institutions. It should also be noted if the systematic review contributes towards a degree award.

For peer review only

Chapter References

Abela, G. (2017). Benefits of maggot debridement therapy on leg ulcers: a literature review. *British Journal of Community* Nursing, 22(Sup6), S149-S19.

Alonso-Coello, P., Schunemann, H. J., Moberg, J., Brignardello-Petersen, R., Akl, E. A., Davoli, M., Treweek, S., Mustafa, R. A., Rada, G., Rosenbaum, S., Morelli, A., Guyatt, G. H., & Oxman, A. D. (2016). GRADE Evidence to Decision (EtD) frameworks: a systematic and transparent approach to making well informed healthcare choices. 1: Introduction. *BMJ, 353*, i2016. doi: 10.1136/bmj.i2016

Arabloo, J., Grey, S., Mobinizadeh, M., Olyaeemanesh, A., Hamouzadeh, P., &Khamisabadi, K. (2016) Safety, effectiveness and economic aspects of maggot debridement therapy for wound healing. *Medical Journal of the Islam Republic of Iran, 30*, 319.

Bazeley, P. (2012). Integrative Analysis Strategies for Mixed Data Sources. *American Behavioral Scientist, 56*(6), 814-828.

Bressan, V., Bagnasco, A., Aleo, G., Timmins, F., Barisone, M., Bianchi, M., Pellegrini, R., & Sasso, L. (2016). Mixed-methods research in nursing - a critical review. *Journal of Clinical Nursing, 26*(19-20), 2878-2890. doi: 10.1111/jocn.13631

Broome, M. (2000). Chapter 13 Integrative literature reviews for the development of concepts. In Rodgers, B. L. & K. A. Knafl. (Eds.). *Concept Development in Nursing. Foundations, Techniques and Applications.*

Carroll, C., Booth, A., & Cooper, K. (2011). A worked example of "best fit" framework synthesis: A systeamtic review of views concerning the taking of some potential chemopreventive agents. *BMC Medical Research Methodology, 11*, 29.

Dixon-Woods, M., Agarwal, S., Jones, D., Young, B., & Sutton, A. (2005). Synthesising qualitative and quantitative evidence: a review of possible methods. *J Health Serv Res Policy, 10*(1), 45-53. doi: 10.1177/135581960501000110

Frantzen, K. K., & Fetters, M. D. (2016). Meta-integration for synthesizing data in a systematic mixed studies review: insights from research on autism spectrum disorder. *Quality & Quantity, 50*(5), 2251-2277. doi: 10.1007/s11135-015-0261-6

Guillaumie, L., Boiral, O., & Champagne, J. (2017). A mixed-methods systematic review of the effects of mindfulness on nurses. *J Adv Nurs, 73*(5), 1017-1034. doi: 10.1111/jan.13176

Hayvaert, M., Hannes, K., & Onghena, P. (2017). Data synthesis for integrated MMRS literature reviews *Using mixed methods research synthesis for literature reviews* (pp. 217-245). Los Angeles, USA: SAGE Publications.

Heyvaert, M., Maes, B., & Onghena, P. (2013). Mixed methods research synthesis: definition, framework, and potential. *Quality and Quantity, 47*(2), 659-676.

Holley, S., Morris, R., Knibb, R., Latter, S., Liossi, C., Mitchell, F., & Roberts, G. (2017). Barriers and facilitators to asthma self-management in adolescents: A systematic review of qualitative and quantitative studies. *Pediatr Pulmonol, 52*(4), 430-442. doi: 10.1002/ppul.23556

Hong, Q. N., Pluye, P., Bujold, M., & Wassef, M. (2017). Convergent and sequential synthesis designs: implications for conducting and reporting systematic reviews of qualitative and quantitative evidence. *Syst ematic Reviews,* 6(1), 61.

McCaughan, D., Cullum, N., & Dumville, J. (2015). Patients' perceptions and experiences of venous leg ulceration and their attitudes to larval therapy: an in-depth qualitative study. *Health Expectations, 18*(4), 527-41.

Menon, J. (2012). Maggot therapy: a literature review of methods and patient experience. *British Journal of Nursing, 21*(5), S38-42.

Munn, Z., Tufanaru, C., & Aromataris, E. (2014). JBI's systematic reviews: data extraction and synthesis. *American Journal of Nursing, 114*(7), 49-54.

Pawson, R., Greenhalgh, T., Harvey, G., & Walshe, K. (2005). Realist review – a new method of systematic review designed for complex policy interventions. *Journal of Health Services Research and Policy, 10 Suppl 1* 21-34.

Pluye, P., & Hong, Q. N. (2014). Combining the power of stories and the power of numbers: mixed methods research and mixed studies reviews. *Annu Rev Public Health, 35*, 29-45. doi: 10.1146/annurev-publhealth-032013-182440

Sandelowski, M., Leeman, J., Knafl, K., & Crandell, J. L. (2013). Text-in-context: a method for extracting findings in mixed-methods mixed research synthesis studies. *J Adv Nurs, 69*(6), 1428-1437. doi: 10.1111/jan.12000

For peer review only

Sandelowski, M., Voils, C. I., & Barroso, J. (2006). Defining and Designing Mixed Research Synthesis Studies. *Res Sch, 13*(1), 29.

Spilsbury, K., Cullum, N., Dumville, J., O'Meara, S., Petherick, E., & Thompson, C. (2008). Exploring patient perceptions of larval therapy as a potential treatment for venous leg ulceration. *Health Expect, 11*(2), 148-159. doi: 10.1111/j.1369-7625.2008.00491.x

Sun, X., Jiang, K., Chen, J., Wu, L., Lu, H., Wang, A., & Wang, J. (2014). A systematic review of maggot debridement therapy for chronically infected wounds and ulcers. *International Journal of Infectious Diseases, 25,* 32-7.

The Joanna Briggs Institute. (2014). Joanna Briggs Institute Reviewers' Manual: 2014 edition / Supplement Methodology for JBI Mixed Methods Systematic Reviews. Adelaide, Australia.

The Joanna Briggs Institute, Lockwood, C., Porrit, K., Munn, Z., Rittenmeyer, L., Salmond, S., Bjerrum, M., Loveday, H., Carrier, J., & Stannard, D. (2017). Chapter 2: Systematic reviews of qualitative evidence. In E. Aromataris & Z. Munn. (Eds.), *Joanna Briggs Institute Reviewer's Manual*

Thomas, J., & Harden, A. (2008). Methods for the thematic synthesis of qualitative research in systematic reviews. *BMC Medical Research Methodology, 8*, 45.

Tian, X., Liang, X. M., Song, G. M., Zhao, Y., & Yang, X. L. (2013). Maggot debridement therapy for the treatment of diabetic foot ulcers: a meta-analysis. *Journal of Wound Care, 22*(9), 462-9.

Voils, C. I., Hassselblad, V., Crandell, J. L., Chang, Y., Lee, E., & Sandelowski, M. (2009). A Bayesian method for the synthesis of evidence from qualitative and quantitative reports: the example of antiretroviral medication adherence. *Journal of health services research & policy, 14*(4), 226-233. doi: 10.1258/jhsrp.2009.008186

Wilasrusmee, C., Marjareonrungrung, M., Eamkong, S., Attia, J., Poprom, N., Jirasisrithum, S., & Thakkinstian, A. (2014). Maggot therapy for chronic ulcer: a retrospective cohort and a meta-analysis. *Asian Journal of Surgery, 37*(3): 138-47.

Zhang, Y., Alonso-Coello, P., Guyatt, G. H., Yepes-Nuñez, J. J., Akl, E. A., Hazlewood, G., Pardo-Hernandez, H., Etxeandia-Ikobaltzeta, I., Qaseem, A., Williams, J. W. Jr., Tugwell, P., Flottorp, S., Chang, Y., Zhang, Y., Mustafa, R. A., Rojas, M. X., & Schünemann, H. J. (2018). GRADE Guidelines: 19. Assessing the certainty of evidence in the importance of outcomes or values and preferences— Risk of bias and indirectness. *Journal of Clinical Epidemiology*. doi: 10.1016/j.jclinepi.2018.01.013

For peer review only

Appendix 8.1 JBI Mixed Methods Data Extraction Form following a Convergent Integrated Approach

Note: This form should only be used for reviews that follow a convergent integrated approach, i.e. integration of qualitative data and 'qualitized' data following data transformation. For reviews that follow a convergent segregated approach, reviewers should use separate data extraction forms: the JBI quantitative data extraction tool and the JBI qualitative data extraction tool.

Reviewer: ______________________ Date: ______________________

Author(s) of the publication: ______________________ Year ______________________

Journal ______________________ Record Number ______________________

Type of study

- ☐ Quantitative study
- ☐ Qualitative study
- ☐ Mixed methods study

Methodology: (e.g. randomized controlled trial, phenomenology)

Number of participants:

Characteristics of participants

Phenomena of interest

Setting and other context-related information (e.g. cultural, geographical)

Outcomes or findings of significance to the review objectives

For a quantitative study, for example

**Results**

For peer review only

· 29% of survey participants reported feeling embarrassed having an asthma attack with friends; only 39% disclosed their asthma to friends<br><br>· 32% were embarrassed about taking asthma medication in front of friends; only 38% reported taking asthma pump when going out<br><br>Reference: (Cohen et al., 2003)

For a qualitative study, for example:

**Themes or Subtheme**	**Illustration (a direct quotation from a participant, an observation or other supporting data from the paper)**
Parental support	'I can take my medicines by myself, but my parents remind me of taking the medicines and they fill prescriptions at the pharmacy. I always talk to the pediatrician or asthma nurse together with my parents.' (page 834, Koster et al., 2015)

Author's conclusion

Reviewer's Comments

For peer review only

Chapter 9: Diagnostic test accuracy systematic reviews

Jared M. Campbell, Miloslav Klugar, Sandrine Ding, Dennis P. Carmody, Sasja J. Hakonsen, Yuri T. Jadotte, Sarahlouise White, Zachary Munn.

How to cite:

Campbell JM, Kulgar M, Ding S, Carmody DP, Hakonsen SJ, Jadotte YT, White S, Munn Z. Chapter 9: Diagnostic test accuracy systematic reviews. In: Aromataris E, Munn Z (Editors). *JBI Manual for Evidence Synthesis.* JBI, 2020. Available from https://synthesismanual.jbi.global. https://doi.org/10.46658/JBIMES-20-10

Chapter 9: Contents

- 9.1 Background
  - 9.1.1 Measures of diagnostic test accuracy
    - 9.1.1.1 Sensitivity and specificity
    - 9.1.1.2 Predictive values
    - 9.1.1.3 Likelihood ratios
    - 9.1.1.4 ROC analyses
- 9.2 Protocol and title development
  - 9.2.1Title
  - 9.2.2 Review question or objective
  - 9.2.3 Inclusion/exclusion criteria
    - 9.2.3.1 Population
    - 9.2.3.2 Index test
    - 9.2.3.3 Reference test
    - 9.2.3.4 Diagnosis of interest
    - 9.2.3.5 Types of studies
  - 9.2.4 Search strategy
  - 9.2.5 Assessment of methodological quality
  - 9.2.6 Data extraction
  - 9.2.7 Data synthesis
    - 9.2.7.1 Graphic representation
    - 9.2.7.2 Meta-analysis
      - 9.2.7.2.1 Context
      - 9.2.7.2.2 Models
      - 9.2.7.2.3 Heterogeneity
  - 9.2.8 References
- 9.3 Searching for studies of diagnostic test accuracy
- 9.4 The systematic review of studies of diagnostic test accuracy
  - 9.4.1 Executive summary
  - 9.4.2 Summary of findings table
  - 9.4.3 Background
  - 9.4.4 Methods
  - 9.4.5 Results
  - 9.4.6 Discussion
  - 9.4.7 References
  - 9.4.8 Appendices
- 9.5 Chaper references
- Appendix 9.1 Critical appraisal checklist
- Appendix 9.2 Data extraction instrument
- Appendix 9.3 Meta-analysis equations and models
- Appendix 9.4 Examples of databases

For peer review only

9.1 Background

Diagnostic tests are used by clinicians to identify the presence or absence of a condition in a patient for the purpose of developing an appropriate treatment plan (White et al. 2011). They can include imaging and biochemical technologies, pathological and psychological investigation, and signs and symptoms observed during history taking and clinical evaluations (Deeks. 2001). New diagnostic tests are continuously developed, driven by demands for improvements in speed, cost, ease of performance, patient safety and accuracy (White et al. 2011). Consequently there are often several tests available for the diagnosis of a particular condition. This highlights the importance of clinicians and other healthcare practitioners having access to high level evidence on the accuracy of the diagnostic tests they use or are considering using. The end goal of diagnostic tests is that they result in improved outcomes in areas that are important to the patient. Systematic reviews that investigate whether diagnostic tests improve outcomes are reviews of effectiveness, however, and should be carried out using the methodology from the chapter on effectiveness. Primary studies that investigate the accuracy of diagnostic tests are termed diagnostic test accuracy (DTA) studies, and it is the systematic review of these which will be the focus of this chapter.

Diagnostic test accuracy studies compare a diagnostic test of interest (the 'index test') to an existing diagnostic test (the 'reference test'), which is known to be the best test currently available for accurately identifying the presence or absence of the condition of interest. The outcomes of the two tests are then compared with one another in order to evaluate the accuracy of the index test. There are two main types of studies of DTA. The first is the diagnostic case- control design, also sometimes called the 'two gate design'. In this study design people with the condition (cases) come from one population (i.e. a health care centre for people known to have the condition), while people without the condition come from another. Although this design gives an indication of the maximum accuracy of the test, the results will generally give an exaggerated indication of the test's accuracy in practice (Leeflang et al. 2013).

The second study design is cross-sectional, and involves all patients suspected of having the condition of interest undergoing the index test and the reference test. Those who test positive for the condition by the reference test can be considered to be the cases, whereas those who test negative are the controls.

This study design is held to reflect actual practice better and is more likely to provide a valid estimate of diagnostic accuracy (Leeflang et al. 2013).

Systematic reviews of diagnostic test accuracy provide a summary of test performance based on all available evidence, evaluate the quality of published studies, and account for variation in findings between studies (Deeks. 2001; Leeflang et al. 2013). Estimates of test accuracy frequently vary between studies, often due to differences in how test positivity is defined, study design, patient characteristics and positioning of the test in the diagnostic pathway (Leeflang et al. 2013). Furthermore, DTA studies have unique design characteristics which require different criteria for critical appraisal compared to other sources of quantitative evidence, and report a pair of related summary statistics ('sensitivity and specificity', as discussed below) rather than a single statistic such as an odds ratio. Consequently systematic reviews of DTA studies require different statistical methods for meta-analytical pooling, and different approaches for narrative synthesis (Leeflang et al. 2014).

Diagnostic accuracy is predominantly represented by two measures, sensitivity and specificity; however sometimes other measures, including predictive values, odds-ratios, likelihood ratios, and summary receiver operating characteristic (ROC) curves, are used (Leeflang et al. 2014). Sensitivity refers to the probability of a person with the condition of interest having a positive result (also known as the true positive proportion [TPP]), while specificity is the probability of a person without the condition of interest having a negative result (also known as the true negative proportion [TNP]) (Leeflang et al. 2014). It should be noted that these definitions refer to the clinical situation, and other definitions of sensitivity and specificity exist that are used in different contexts (Sackett and Haynes. 2002). Sensitivity and specificity have been identified as essential measures of diagnostic accuracy (Leeflang et al. 2013; Leeflang et al. 2014; Habbema et al. 2009; Leeflang et al. 2013b).

For peer review only

9.1.1 Measures of diagnostic test accuracy

Several pairs of measures are used to determine how well a diagnostic test performs relative to the known proportions of individuals with and without the disorder. Diagnostic accuracy is critical in the evaluation of medical diagnostic tests (Leeflang et al. 2013b). Methods to summarize the results of diagnostic studies are available for both binary and continuous data (Lau et al. 1997; Whiting et al. 2004). Measures of overall accuracy are affected by the prevalence of the disorder (Leeflang et al. 2009). In addition, estimates may vary greatly between studies due to differences in the criteria used to declare a test positive, patient characteristics, and study design (Leeflang et al. 2013).

For peer review only

9.1.1.1 Sensitivity and specificity

The most commonly used measures are sensitivity and specificity. Sensitivity is the probability that a person with the condition of interest will have a positive result, while specificity is the probability of a person without the condition having a negative result (Altman and Bland 1994).

Specifically, sensitivity can be calculated as $\frac{True\ positives}{(True\ positives + False\ negatives)}$ while specificity can be calculated as $\frac{True\ negatives}{(True\ negatives + False\ positives)}$

The definitions of what are counted as true positive and negative results are given in Table 9.1.

Table 9.1: Classification of patient test results by condition status

Index Test Outcome	Reference positive	reference negative	Total
Index test positive (T+)	True positives (TP)	False positives (FP)	Test positives (TP+FP)
Index test negative (T-)	False negatives (FN)	True Negatives (TN)	Test negatives (FN+TN)
Total	Reference positives (TP+FN)	Reference negatives (FP+TN)	N (TP+FP+FN+TN)

Sensitivity and specificity co-vary with the decision threshold used to identify the disorder (Lalkhen and McCluskey. 2008).

In Table 9.2 example data is presented from Mulligan et al. 2011, who investigated the diagnostic test accuracy of the Lachman test performed in prone position for the diagnosis of torn anterior cruciate ligament (ACL).

Table 9.2: Results from Mulligan et al. 2011

Prone Lachman	Reference Positive	Reference Negative	Total
Positive	16	1	17
Negative	7	28	35
Total	23	29	52

For this study the sensitivity can be calculated as $\frac{16}{16+7} = 0.70$ while the specificity is $\frac{28}{28+1} = 0.97$

For peer review only

9.1.1.2 Predictive values

While sensitivity and specificity measure the accuracy of a diagnostic test, they do not provide the probability of the diagnostic value of the result of the test. Predictive values provide the proportion of patients who are correctly diagnosed (Altman and Bland. 1994b). The positive predictive value $PPV = \frac{TP}{(TP+FP)}$ is the proportion of individuals with positive test results who were correctly diagnosed, while the negative predictive value $NPV = \frac{TN}{TN+FN}$ is the proportion of individuals with negative test results who were correctly diagnosed. From the example presented in Table 9.2 PPV is $\frac{16}{16+1} = 0.94$ while the NPV is $\frac{28}{28+7} = 0.8$ As prevalence does influence predictive values, it is important to account for the prevalence of the disorder in the population under study, given that the higher the prevalence the higher the PPV (Brenner and Gefeller. 1997).

For peer review only

9.1.1.3 Likelihood ratios

Likelihood ratios assess the probability or likelihood that the test result obtained would be expected in a person with the condition, compared to the probability or likelihood that the same result would be seen in a person without the condition (Deeks. 2001). The positive likelihood ratio $LR+=\frac{sensitivity}{(1-specificity)}=\frac{TP}{(TP+FN)}\div\frac{FP}{(FP+TN)}$ expresses how many times more likely people with the condition are to receive a positive test result compared to those who do not have the condition, while the negative likelihood ratio $LR-=\frac{(1-sensitivity)}{(specificity)}=\frac{FN}{(TP+FN)}\div\frac{TN}{(FP+TN)}$ expresses how likely it is that people with the condition will receive a negative test result compared to those who do not have the condition.

From the example presented in Table 9.2, LR+ is $\frac{0.70}{1-0.97}=23.33$ while LR- is $\frac{1-0.70}{0.97}=0.31$.

The initial assessment of the likelihood of a disorder, that is the *a priori* probability, is modified by the results of the diagnostic test for a *posteriori* probability (the probability actually observed). A suggestion on the limited use of likelihood ratios is that their interpretation requires a calculator to convert between probabilities and odds of the disorder (McGee. 2002).

For peer review only

9.1.1.4 ROC analyses

Receiver Operating Characteristic (ROC) curve analysis is useful for evaluating the performance of diagnostic tests that classify individuals into categories of those with and those without a condition (Zou et al. 2007; Metz et al. 1978). The data obtained from a diagnostic test will often exist on a scale (i.e. blood pressure, hormone concentration), and a decision will need to be made on whether a certain test value indicates that the condition is present (positive test) or not (negative test). Where this 'line' is drawn is termed the decision or positivity threshold. For example, a blood pressure cut-off value for hypertension is 135/80.

The choice of a decision threshold will have a large effect on the sensitivity and specificity of a test. While setting a low threshold will result in a large proportion of true positives being correctly identified as positive, it will also decrease the rate of true negatives. In other words, a lower threshold increases sensitivity but decreases specificity. The inverse is also true for high thresholds. As sensitivity and specificity depend on the selection of a decision threshold, ROC analysis is used to plot the sensitivity (y-axis) against 1-specificity (x-axis) as the threshold value changes (Macaskill et al. 2010). This gives a visual representation of the relationship between sensitivity and specificity of a diagnostic test as the threshold value changes. This can be measured quantitatively by assessing the area under the curve (AUC) (Hanley and McNeil. 1982). The AUC for a perfect test is 1.0, and a test with no differentiation between disorder and no disorder has an AUC of 0.5 (Lalkhen and McCluskey. 2008).

Figure 9.1 shows an ROC curve from Erol et al. 2014 with an AUC of 0.81 (95%CI 0.80 to 0.82).

The diagonal line shows the baseline result of a test with no differential power (AUC=0.5).

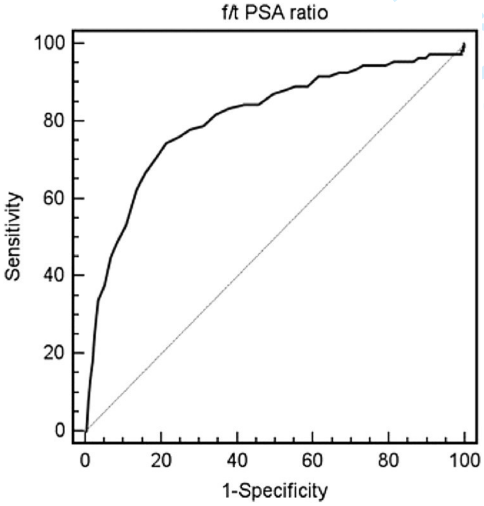

Figure 9.1: ROC graph for the use of prostate specific antigen free/total ratios for the diagnosis of prostate cancer

For peer review only

9.2 Protocol and title development

For peer review only

9.2.1Title

The title should be clear, explicit and reflect the core elements of the review. This creates the best chance of correct indexing by databases and easy identification by interested readers. The title should explicitly state that it is on 'diagnostic test accuracy' and include the phrase '…: a systematic review protocol'. Titles should not be phrased as questions or conclusions and there should be congruency between the title, review objectives/questions and inclusion criteria.

The title should include each of the elements of the PIRD acronym (discussed below), and approximately follow the form of: "The accuracy of INDEX relative to REFERENCE for the diagnosis of DIAGNOSIS in POPULATION : a systematic review protocol of diagnostic test accuracy"

Example titles are:

- "The accuracy of laboratory testing relative to viral culture for the diagnosis of influenza A (H1N1) 'swine flu' in people presenting with suspected flu: a systematic review protocol of diagnostic test accuracy"
- "The accuracy of endoscopic retrograde cholangiopancreatography relative to confirmed bile stone extraction for the diagnosis of common bile duct stones in in patients with clinical symptoms of common bile duct stones: a systematic review protocol of diagnostic test accuracy"
- "The accuracy of automated semen analysis for the diagnosis of infertility in the male partner of an infertile couple relative to laboratory technician analysis: a systematic review protocol of diagnostic test accuracy"

For peer review only

9.2.2 Review question or objective

Developing a good review question/objective is an important step in undertaking a quality systematic review as it sets out the key components of the review (i.e. population, index test, reference test, objectives).

An example of a well written review objective is “To determine the diagnostic accuracy of currently available laboratory tests for swine flu (H1N1) using viral culture as a reference test amongst people presenting with suspected flu” which could alternatively be phrased as the question “What is the diagnostic accuracy of currently available laboratory tests for swine flu (H1N1) compared to viral culture as a reference test amongst people presenting with suspected flu?”

For peer review only

9.2.3 Inclusion/exclusion criteria

The mnemonic PIRD is recommended for setting the inclusion criteria for systematic reviews of diagnostic test accuracy:

- Population
- Index test
- Reference test
- Diagnosis of interest

For peer review only

9.2.3.1 Population

The types of participants should be appropriate for the review objectives and reflect who will undergo the diagnostic test in clinical practice. If test results are extrapolated to other populations, this may result in an inaccurate estimation of test accuracy and should therefore be avoided. The reasons for the inclusion or exclusion of participants should be explained in the Background section and be based on clear scientific justifications. Population characteristics that may be relevant to outline in detail include disease stage, symptoms, age, sex, race, educational status, etc. An example ‘population’ is “individuals presenting with flu symptoms”.

For peer review only

9.2.3.2 Index test

The index test(s) is the diagnostic test whose accuracy is being investigated in the review. Sometimes multiple iterations of a specific test will exist, and it should be specified at the protocol stage what criteria will be used to determine if the tests are similar enough to combine in meta- analysis. The criteria by which the index test results will be categorized as being positive or negative (the threshold value) can also be specified at the protocol stage. It may be appropriate for reviewers to specify who carries out or interprets the test, the conditions under which the test is conducted (i.e. laboratory, clinical), and specific details regarding how the test will be conducted. An example of 'index test' is "currently available laboratory tests (PCR test)".

For peer review only

9.2.3.3 Reference test

The reference test is the ‘gold standard’ test to which the results of the index test will be compared. It should be the best test currently available for the diagnosis of the condition of interest. The same standards for describing the index test should be followed for describing the reference test in the protocol; the details of what criteria will be used to determine which tests ‘count’ as being the reference test, and how results will be categorized as positive or negative should be specified. Systematic reviews of diagnostic test accuracy must specify a reference test. An example ‘reference test’ is “viral culture”.

For peer review only

9.2.3.4 Diagnosis of interest

This item relates to what diagnosis is being investigated in the systematic review. This may be a disease, injury, disability or any other pathological condition. In some cases (i.e. where the index or reference tests are only used for one purpose or the 'population' specifies "patients suspected of…") this factor may seem redundant. However, as a general rule it is useful to explicitly specify the diagnosis of interest. An example 'diagnosis of interest' is "swine flu (H1N1)"

For peer review only

9.2.3.5 Types of studies

In this section the types of studies which will be considered for inclusion in the review are described. As detailed above, diagnostic studies generally use cross-sectional or case-control study designs. It should be noted however that restricting database searches by study design may result in studies which contain accuracy data being missed.

For peer review only

9.2.4 Search strategy

This section should detail how the reviewers will search for relevant papers. The documentation of search strategies is a key element of the scientific validity of a systematic review. It enables readers to look at and evaluate the steps taken and decisions made to consider the comprehensiveness and exhaustiveness of the search strategy for each included database. Initial keywords should be specified in the protocol along with the databases to be searched. A three-stage search strategy is recommended including an initial search of the select databases using the pre-specified keywords to identify additional relevant keywords and index terms, a second thorough search across all included databases, and then a final review of the reference lists of included studies in order to identify any studies that may have been missed. A completed search strategy for one database should be attached to the protocol as Appendix 1. If searching is restricted to a specific date range, then that should be specified in the protocol, as well as any language restrictions which may be applied. For further information on searching refer to the 'Searching for studies of diagnostic test accuracy' section.

For peer review only

9.2.5 Assessment of methodological quality

Assessing the quality of diagnostic studies being considered for inclusion is a vital part of the systematic review process. Methodological quality relates to the risk of bias resulting from the design and conduct of the study. The quality of a diagnostic study is determined by its design, the methods by which the study sample is recruited, the conduct of tests involved, blinding in the process of interpreting tests, and the completeness of the study report. The process of critical appraisal examines the methodology of a study against pre-defined criteria, with the aim of considering individual sources of risk of bias and is used to evaluate the extent to which the results of a study should be believed or to be deemed valid after rigorous assessment (Reitsma et al. 2009).

Table 9.3 is modified and expanded from “Synthesizing evidence of diagnostic accuracy” (White et al. 2011; Reitsma et al. 2009) and highlights the major types of bias that can occur in diagnostic accuracy studies as a result of flawed or incomplete reporting. Attempts such as those by the Standards for Reporting of Diagnostic Accuracy (STARD) initiative (Bossuyt et al. 2003; Meyer et al. 2003), have been made to improve reporting, methodological quality and to aid primary researchers to address and avoid sources of bias.

Table 9.3: Types of bias in studies of diagnostic test accuracy

	**Type of bias**	**When does it occur?**	**Impact on accuracy**	**Preventative measures**
**Patients/Subjects**	Spectrum bias	When included patients do not represent the intended spectrum of severity for the target condition or alternative conditions	Depends on which end of the disease spectrum the included patients represent	Ensure that the included patients represent a broad sample of those that the test is intended for use with in clinical practice
	Selection bias	When eligible patients are not enrolled consecutively or randomly	Usually leads to overestimation of accuracy	Consider all eligible patients and enroll either consecutively or randomly
**Index test**	Information bias	When the index results are interpreted with knowledge of the reference test results, or with more (or less) information than in practice	Usually leads to overestimation of accuracy, unless less clinical information is provided than in practice, which may result in an under estimation of accuracy	Index test results should be interpreted without knowledge of the reference test results, or with more (or less) information than in practice
**Reference test**	Misclassification bias	When the reference test does not correctly classify patients with the target condition	Depends on whether both the reference and index test make the same mistakes	Ensure that the reference correctly classifies patients within the target condition
	Partial verification bias	When a non-random set of patients does not undergo the reference test	Usually leads to overestimation of sensitivity, effect on specificity varies	Ensure that all patients undergo both the reference and index tests
	Differential verification bias	When a non-random set of patients is verified with a second or third reference test, especially when this selection depends on the index test result	Usually leads to overestimation of accuracy	Ensure that all patients undergo both the reference and index tests
	Incorporation bias	When the index test is incorporated in a (composite) reference test	Usually leads to overestimation of accuracy	Ensure that the reference and test are performed separately
	Disease/Condition progression bias	When the patients’ condition changes between administering the index and reference test	Under- or Over-estimation of accuracy, depending on the change in the patients’ condition	Perform the reference and index with minimal delay. Ideally at the same time where practical
	Information bias	When the reference test data is interpreted with the knowledge of the index test results	Usually leads to overestimation of accuracy	Interpret the reference and index data independently

For peer review only

**Data analysis**	Excluded data	When uninterpretable or intermediate test results and withdrawals are not included in the analysis	Usually leads to overestimation of accuracy	Ensure that all patients who entered the study are accounted for and that all uninterpretable or intermediate test results are explained

The most widely used tool for examining diagnostic accuracy is the QUADAS 2 which was released in 2011 following the revision of the original QUADAS (Quality Assessment of Diagnostic Accuracy Studies) tool (Whiting et al. 2011). JBI encourages the use of QUADAS 2, and this chapter includes a checklist which incorporates the "signaling questions" from QUADAS 2 (Appendix I). It should be noted that QUADAS 2 includes questions regarding the level of concern that reviewers have for the applicability of the study under consideration to the research question. For JBI DTA systematic reviews, a primary research study should not proceed to critical appraisal if there is concern that the study does not match the inclusion criteria and research question. As such, this element of QUADAS2 is not addressed in the below checklist (Domains

1, 2, 3, 4).

Domain 1: Patient selection

In this section the risk of selection bias is assessed by how patients were selected for the study.

- Was a consecutive or random sample of patients enrolled?
- Was a case-control design avoided?
- Did the study avoid inappropriate exclusions?

Domain 2: Index tests

In this section consideration is on whether the conduct and interpretation of the index test being investigated could have introduced bias.

- Were the index test results interpreted without knowledge of the results of the reference standard?
- If a threshold was used, was it pre-specified?

Domain 3: Reference standard/test

The focus of this section is to determine if and the extent that the way in which the reference test was conducted and interpreted could introduce bias into the study.

- Is the reference standard likely to correctly classify the target condition?
- Were the reference standard results interpreted without knowledge of the results of the index test?

Domain 4: Flow and timing

The aim of this section is to determine the risk of bias attributable to the order in which the index and reference tests were conducted in the study. If there is a long time delay between conduct of the two tests, the status of the patient may change and therefore impact the results of the later test. In addition, if the later test is conducted with knowledge of the results of the previous test, interpretation of the results may be impacted.

- Was there an appropriate interval between the index test and reference standard?
- Did all patients receive the same reference standard?
- Were all patients included in the analysis?

The primary and secondary reviewer should discuss each item of appraisal for each study design included in their review. In particular, discussions should focus on what is considered acceptable for the review in terms of the specific study characteristics. The reviewers should be clear on what constitutes acceptable levels of information to allocate a positive appraisal compared with a negative, or a response of "unclear".

For peer review only

This discussion should take place before independently conducting the appraisal. The weight placed on specific critical appraisal questions will vary between reviews and it is up to the reviewers to set what criteria will result in the inclusion/exclusion of a study. Many reviewers select a set of questions which must be answered “Yes” or the review will be excluded. It is important that these criteria be applied consistently across studies. Formerly systematic review protocols published in *JBI Evidence Synthesis* ap pended the appraisal tool which would be used to their protocols. Instead Campbell et al. 2015 which describes the appraisal process and tool should be cited in the relevant section of the protocol method.

For peer review only

9.2.6 Data extraction

Data extraction is the process of sourcing and recording relevant results and details from the primary research studies included in the systematic review. Standardized data extraction tools facilitate the extraction of the same types of data across all of the included studies and are required for JBI systematic reviews. Reviewers should practice using the data extraction tool so they are consistently applied. The protocol should detail what data the reviewers will extract from the included studies and the data extraction tool should be attached in the appendices. Among the most important detail to extract is the decision threshold used.

As well as recording the final results of the study it is important to extract the details that inform generalizability and context of the primary studies. The STARD (Standards for Reporting of Diagnostic Accuracy) checklist and flow diagram provides detailed guidance on what studies of DTA to report and the majority of items are incorporated into the standard data extraction template that is appended to this chapter (Appendix II) (Gatsonis. 2003). You can download the STARD checklist and STARD flow diagram: http://www.stard-statement.org/

To reduce errors in data extraction it is recommended that two independent reviewers extract data and use the standardized instrument.

Studies of diagnostic test accuracy that comply with the STARD statement should include a 2×2 table that classifies patient test results and disease status as shown below (Table 9.4):

Table 9.4: Condition status (reference test results)

Index test<br>Outcome	Condition positive	Condition negative	Total
Index test positive	True positives (a)	False positives (b)	Test positives (a + b)
Index test negative	False negatives (c)	True negatives (d)	Test negatives (c + d)
Total	Disease/condition positives (a + c)	Disease/condition negatives (b + d)	N (a + b + c + d)

This should essentially include all quantitative data that is needed for the extraction.

For peer review only

9.2.7 Data synthesis

Finally, the protocol should describe how the outcome data of the primary studies will be combined and reported, i.e. meta-analysis, narrative synthesis, graphical representation, etc. Options for summarizing and presenting data are discussed further below.

For peer review only

9.2.7.1 Graphic representation

Results of diagnostic test accuracy systematic reviews can be graphically represented through two different major ways.

As for systematic reviews of effectiveness, forest plots can be performed. In the case of diagnostic test accuracy, two forest plots are presented side by side: one for sensitivity and the other for specificity. These graphs thus show the means and confidence intervals for sensitivity/specificity for each of the selected primary studies. These values are also listed in numerical form. Moreover, the number of true positives, false positives, true negatives and false negatives are also reported, as well as, where appropriate, any covariates (for instance the type of diagnostic test used). Figure 9.2 shows a paired forest plot made using mock data (Campbell et al. 2015).

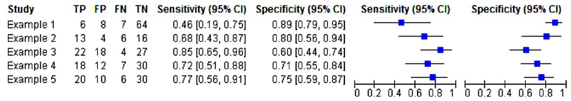

Figure 9.2: An example paired forest plot generated using mock data in RevMan5

Numerical values for sensitivity and specificity are presented alongside graphical representations where the boxes mark the values and the horizontal lines show the confidence intervals.

It is also possible to create Summary ROC (SROC) curves. They are graphs with 1-specificity on the x-axis and sensitivity on the y-axis, in which each primary study contributes to a unique point defined by its sensitivity and specificity for a given threshold. If several thresholds are reported in a single primary study, only one sensitivity/specificity pair for that study can be plotted on the SROC graph. Point size may vary according to sample size. To indicate more precisely the precision of the estimates, point height is proportional to the number of diseased patients, while point width is proportional to the number of control patients.

Following a rigorous meta-analysis, a curve can be added in the graph. A Summary ROC curve represents the expected ROC curve at many different positivity threshold levels. If the same positivity threshold has been used in each of the primary studies, it is appropriate to calculate and plot the summary sensitivity and specificity, and their confidence region. A prediction region can also be provided, corresponding to the area where the true sensitivity/specificity of a future study should be found in 95% of the cases. Figure 9.3 shows a SROC curve from made using mock data in RevMan5 (Campbell et al. 2015).

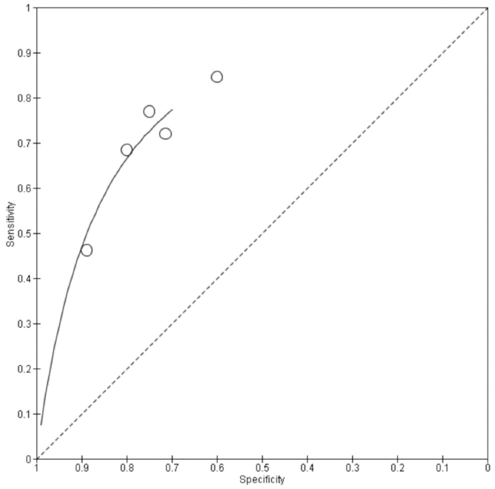

Figure 9.3: An SROC curve generated using mock data in RevMan5. Sensitivity is shown on the y-axis, the x-axis shows inverted specificity (Campbell et al. 2015)

For peer review only

9.2.7.2 Meta-analysis

Meta-analysis of data from studies of diagnostic test accuracy is more complicated than most other forms of meta-analysis (principally due to the paired nature of the main outcome measures sensitivity /specificity). As such the early involvement of a statistician is advisable.

For peer review only

9.2.7.2.1 Context

Authors of diagnostic test accuracy systematic reviews need to define the kind of meta-analysis to perform. Questions to consider are:

- Should we estimate summary sensitivity and specificity?
- Should we compute a summary ROC curve?

The answer to these questions lies in the kind of data available and more exactly whether the diagnostic threshold is the same across the selected primary studies. Sometimes retrieved studies may rigorously use the same diagnostic threshold, but, on other occasions variations in the threshold may exist. This is often the case when there is no explicit numerical cut-off-point or when the index test is based on an observer's judgement.

The basic strategy is as follows:

- When the same threshold is used through the primary studies, then:
  - estimate the summary sensitivity/specificity.
- When different thresholds are used, then:
  - produce a SROC curve; and
  - estimate the summary sensitivity/specificity for each threshold provided in the articles.

If a study has referred to sensitivity/specificity values for several threshold, it can contribute to several estimations of summary sensitivity/specificity.

For peer review only

9.2.7.2.2 Models

Methods for performing meta-analyses regarding diagnostic tests are still being debated in the literature and new statistical developments are underway (Eusebi et al. 2014). Three main models exist. The first one corresponds to a fixed effect model whereas the other two are random effect models. These last two are based on a hierarchical model, taking into account the variability present within studies and between studies. Exact mathematical details for each model discussed are provided in Appendix III.

- The Moses-Littenberg model (Littenberg et al. 1990; Moses et al. 1993) has been extensively used for meta-analyses of DTA (Holling et al. 2012).

However, it is principally a fixed effect model, whereas for many such analyses a random effect model is required. It allows the performance of SROC curves in an exploratory approach. As a fixed effect model, it does not take into account and does not consider the variability between studies.

Due to its evident simplicity (it notably does not integrate the inter-study variability), this model can, in some circumstances, produce very different SROC curves compared to the hierarchical model described below (Harbord et al. 2008). The Cochrane Collaboration recommends careful use of this model which should be limited to preliminary analyses. Confidence intervals in statistics estimates or investigations of heterogeneity should not be studied with this model. 19

- The Bivariate model (Reitsma et al. 2009) estimates the summary parameters: sensitivity and specificity across primary studies. It is presented in the Cochrane Handbook (Macaskill et al. 2010) and in the article of Leeflang et al. 2014 as a method of choice.

In this method, following Chu & Cole et al. 2006, the within study variability is modelled through binomial distributions, one for sensitivity and the other for specificity. These distributions are treated jointly since estimates of sensitivity and specificity, within each study, are non-independent.

- To deal with variability in positivity cutpoint values, Rutter and Gatsonis. 2001 developed the hierarchal SROC (HSROC) model. It produces a SROC in which each study provides only one pair of values for sensitivity and specificity. It is presented in the Cochrane Handbook (Macaskill et al. 2010) and in the article by Leeflang et al. 2014 as a method of choice to obtain SROC curves.

For peer review only

9.2.7.2.3 Heterogeneity

Systematic reviews of DTA frequently find heterogeneity between studies (Macaskill et al. 2010). This can be due to differences in study populations, procedures followed for carrying out the test (index or reference), and the conditions or context of testing.

Additionally, heterogeneity can be the result of differences in how studies have been conducted or their data analyzed which have biased the results (for example, one study may have included all test results in the final analysis, whereas another may exclude inconclusive outcomes, thereby making the test appear more accurate than it actually is). As such the presence of heterogeneity between studies should be carefully investigated. Displaying data graphically through paired forest plots or SROC curves can help to identify the presence or absence of heterogeneity (albeit subjectively), as large differences between studies, if present, will be recognizable. If there are differences in the diagnostic threshold between studies, then paired forest plots should not be used to estimate heterogeneity as variability should exist due to the interdependence of sensitivity and specificity. In these cases heterogeneity can be estimated by judging how well studies fit with the SROC curve (and not by how scattered they are). The Chi-square test or Fisher's exact test can be used for more objective assessments of heterogeneity, however their power has been noted to be low (Dinnes et al. 2005). The I^2 test is not routinely used in DTA systematic reviews as it does not account for the influence that differing positivity thresholds can have.

Where heterogeneity is found, its cause should be carefully investigated by comparing the characteristics of the differing studies. If this comparison suggests that the heterogeneity is due to the existence of specific risks of bias in some studies, then meta-analysis should be restricted to studies which do not possess the identified risks (as with all systematic reviews, efforts should be made to identify potential subgroup analyzes and the intention to carry them out declared a priori in the protocol) (White et al. 2011). Unfortunately, subgroup analysis carries its own difficulties, as when subgroups contain a low number of studies, they are prone to heterogeneity (White et al. 2011). The use of random effects models of meta-analysis (discussed above) can help to identify heterogeneity by adding a covariate into the model. The covariate, either categorical or continuous, is accordingly assumed to be the heterogeneity source. These values are not easily interpreted, however, as they show variation in parameters expressed on log odds scales (Leeflang et al. 2013). When the extent of heterogeneity cannot be explained, reviewers should refrain from meta-analysis and instead conduct a narrative synthesis.

For peer review only

9.2.8 References

The protocol should include all references in full, using the Vancouver referencing style, in the order in which they appear.

Appendices

The protocol should include a full search strategy for one database and the data extraction tool appended as appendices. These tools must match the criteria specified in the Inclusion Criteria and critical Appraisal sections. Appendices should be numbered using Roman numerals.

For peer review only

9.3 Searching for studies of diagnostic test accuracy

The aim of the search strategy is to generate a list of studies from the literature which is as comprehensive as possible and which may be suitable for answering the research question posed by the systematic review. The literature encompasses several types of published and unpublished material (grey literature), including journal articles, dissertations, editorials, conference proceedings and reports. Methods by which these sources can be found vary from searching electronic databases to hand searching journals and conference proceedings, checking reference lists of relevant publications, tracking citations of relevant studies and contacting experts (White et al. 2011; de Vet et al. 2008).

The timeframe chosen for the search should be justified and any language restrictions stated

(e.g. if only studies published in English are considered for inclusion).

The search strategy for a JBI systematic review should be conducted in three phases:

Stage 1: Identification of keywords and search terms

A limited search should be undertaken in major databases (such as MEDLINE) using preliminary search terms. The aim of this stage is to locate some papers relevant to the review and determine whether those papers can provide any additional keywords, indexing terms, or subject headings that may help in the search for similar papers. This is done by analyzing words contained in the title, keywords, abstract and indexing list.

Stage 2: Conducting the search across the specified databases

The second phase is to construct database-specific searches (see Appendix IV for general and subject specific databases) for each database included in the protocol. This may involve making slight modifications in how the search terms are entered as each database may have differences in how articles are indexed and terms used to describe articles.

Stage 3: Reference list searching

The final phase of searching involves the review of the reference lists of all studies included in the systematic review for additional studies. Additionally, researchers who are experts in the field of interest may also be considered as a potential source of articles and/or unpublished data.

Unpublished data

The comprehensiveness of searching and the documentation of the databases is a core component of the credibility of a systematic review. In addition to databases of commercially published research, there are several online sources of grey or unpublished literature that should be considered. Grey or gray literature is also known as Deep or Hidden Web material and refers to papers that have not been commercially published and includes: theses and dissertations, reports, blogs, technical notes, non-independent research or other documents produced and published by government agencies, academic institutions and other groups that are not distributed or indexed by commercial publishers. Rather than compete with the published literature, grey literature has the potential to complement and communicate findings to a wider audience, as well as to reduce publication bias. However, an important thing to remember is that the group of databases should be tailored to the particular review topic (White et al. 2011). Examples of sources of grey literature are included in Appendix IV.

Search strategies

Search strategies for identifying diagnostic studies should not be restricted to a particular study design and are predominantly focused on terms for the diagnostic test(s) of interest (index test) and the clinical disorder or disease stage the test is seeking to detect (target condition). If further restriction of search results is required, we recommend exploring the use of additional topic- specific terms first before a methodology search filter for diagnostic test accuracy studies is considered (de Vet et al. 2008). If methodology-specific terms are used to filter the search, examples which have been shown to have good sensitivity include false positive, false negative, sensitivity, specificity, diagnos*, detect*, accura* (Beynon et al. 2013). The terms used will need to be tailored to the database searched, and multiple terms linked with “OR” will be necessary.

Minimizing publication bias

For peer review only

Identifying as many relevant studies as possible and documenting the search for studies with sufficient detail so that it can be reproduced is a key feature that distinguishes a systematic review from a traditional narrative review, and should help to minimize bias and assist in achieving more reliable estimates of diagnostic accuracy. It is important to ensure that the process of identifying studies is as thorough and unbiased as possible, and to be aware of the range of potential biases which might need to be addressed through a variety of search methods. Although the importance of publication bias in diagnostic studies is not yet fully explored, recent research indicates that to achieve as comprehensive a search as possible and thereby minimizing the risk of bias, it is advisable to search several electronic databases and use other methods to retrieve studies (such as checking reference lists, citation searches, hand searching, contacting experts, etc.) (de Vet et al. 2008).

A basic Boolean strategy for searching bibliographic databases is to list synonyms for each element of the PIRD and combine them using “OR” within column and “AND” between columns (Table 9.5).

Table 9.5: Structure of a logic grid

Population		Index		Reference		Diagnosis
Flu symptoms<br>OR<br>Influenza symptoms<br>OR<br>Influenza-like	AND	Laboratory testing<br>OR<br>PCR assay<br>OR<br>PCR test	AND	Viral culture<br>OR<br>Viral test<br>OR<br>Viral assay	AND	Swine flu<br>OR<br>Swine influenza<br>OR<br>H1N1

Depending on the topic area, the number of articles retrieved by such searches may be very large. Methodological filters consisting of text words and database indexing terms have been developed in the hope of improving the searches by increasing their precision when these filters are added to the search terms for the disease and diagnostic test. On the other hand, using filters to identify records for diagnostic reviews may miss relevant studies while at the same time not making a big difference to the number of studies that have to be assessed for inclusion. A systematic review published in 2013 by Beynon et al. assessed the performance of 70 filters (reported in 19 studies) for identifying diagnostic studies in the two main bibliographic databases in health, MEDLINE and EMBASE. The results showed that search filters do not perform consistently, and should not be used as the only approach in formal searches to inform systematic reviews of diagnostic studies. None of the filters reached their minimum criteria of a sensitivity greater than 90% and a precision above 10%. The findings support the current recommendation in the Cochrane Handbook for Systematic Reviews of Diagnostic Test Accuracy, that the combination of methodological filter search terms with terms for the index test and target condition should not be used as the only approach when conducting formal searches to inform systematic reviews of DTA.

Studies on diagnostic accuracy are often based on routinely collected data rather than pre- registered trials, so publication bias may be more prevalent in diagnostic research than in therapeutic research. Searching for studies in languages other than English and for studies that are difficult to locate (grey literature), such as conference proceedings, various types of reports, ongoing studies etc., may be necessary to gain a more complete overview and to get an idea about the size and direction of any publication bias in diagnostic research (White et al. 2011).

Subject-specific databases

In addition to MEDLINE and EMBASE, which are generally considered to be major international general healthcare databases, many countries and regions produce electronic bibliographic databases that concentrate on the literature produced in these regions, and which often include journals and other literature not indexed elsewhere. Access to many of these databases is available free of charge on the internet. Others are only available by subscription or on a ‘pay- as-you-go’ basis. Indexing complexity and consistency varies, as does the sophistication of the search interface, but they can be an important source of additional studies from journal articles not indexed in other international databases such as MEDLINE or EMBASE. When supplementing a search of MEDLINE and EMBASE with databases from other regions, where the prevalence of the target condition of interest in the population may be markedly different, it may be particularly important for authors of DTA reviews to consider possible sources of publication bias.

The subject-specific databases to search in addition to MEDLINE, EMBASE and Cochrane Register of Diagnostic Test Accuracy Studies, will be influenced by the topic of the review, and access to specific databases. Examples of subject-specific databases are included in Appendix IV.

Dissertations and theses databases

For peer review only

Some studies have found that dissertations and theses are more likely to be published in full if results are positive and that, on average, dissertations that remain unpublished have lower effect sizes than published literature (White at al. 2011). It is not yet known whether dissertations in diagnostic test accuracy follow a similar publication pattern, but to minimize possible effects of publication bias authors may wish to consider searching for dissertations and theses. These are not normally indexed in general bibliographic databases such as MEDLINE or EMBASE but there are exceptions, such as CINAHL which indexes nursing dissertations and PsycINFO which indexes dissertations relevant to psychology and psychiatry. Some example databases of theses are included in Appendix IV.

Grey literature databases

As discussed above, the inclusion of grey or unpublished literature is important for minimizing bias in a systematic review as grey literature has been found to be more likely to contain intervention studies reporting non-significant results than those published in healthcare journals. Examples of databases covering grey literature sources are included in Appendix IV.

For peer review only

9.4 The systematic review of studies of diagnostic test accuracy

This section provides information on how to synthesize and write the results of a properly carried out systematic review of diagnostic test accuracy. Additionally, it includes a brief outline of how the systematic review report should be formatted and the stylistic conventions that should be used to ensure the review meets the publication criteria of the *JBI Evidence Synthesis*.

Title

The title should be clear and explicit, and reflect the core elements of the review. As per the advice for the protocol title it should state that it is on 'diagnostic test accuracy' and include the phrase '…: a systematic review' as well as make reference to each of the elements of the PIRD. Titles should not be phrased as questions or conclusions and there should be congruency between the title, review objectives /questions and inclusion criteria.

Reviewers

Each reviewer should have their affiliations listed, including affiliations with a JBI collaborating centre if applicable. An email address should be provided for the corresponding author.

For peer review only

9.4.1 Executive summary

The executive summary is a structured abstract that reflects and summarizes the main features of the systematic review. Maximum word length is 500 words, abbreviations and references should not be used.

The executive summary should include the following headings:

Background

This section should briefly describe and justify the choice of condition and tests under review, as well as provide sufficient detail to justify why the review was conducted.

Objectives/questions

The review objectives or questions should be stated in full, as detailed in the protocol section.

Inclusion criteria

Population: This section should provide the details of the population as described in the protocol

Index test: This section should provide the details of the index test as described in the protocol, including which iterations of the index test are included and how positive or negative outcomes are specified, i.e. the threshold values.

Reference test: This section should provide the details of the reference test as described in the protocol, including which iterations of the index test are included and how positive or negative outcomes were specified.

Diagnosis of interest: This section should state the disease/illness/injury/disability that is being investigated by the diagnostic test and the formal definition, if any, by which it is described.

Types of studies

Detail the study types which are eligible for inclusion in the systematic review as per the protocol

– not the study types which are ultimately found and included. These will be diagnostic case- control and /or diagnostic cross-sectional.

Search strategy

Write a brief description of the systematic review's search strategy (e.g. relevant databases searched, initial search terms or keywords, and any limitations) as specified in the protocol.

Methodological quality

Describe the method or criteria that are used to appraise the included studies.

Data extraction

This section should include a brief description of the types of data extracted and the tool (as specified in the protocol) that is used.

Data synthesis

A brief description of how the data is synthesized.

Results

A brief description of the findings of the review.

Conclusions

A brief description of the conclusions of the review.

Implications for practice

A brief description of any implications that the findings may have for current practice.

Implications for research

A brief description of the implications that the review has for the direction of future research.

For peer review only

9.4.2 Summary of findings table

Systematic reviews of diagnostic studies should be accompanied by a summary of findings table, which should include the question being investigated, the index test, the reference test, the population, the estimates rate of true positives, false negatives, true negatives and false positives and the absolute difference between the index and reference tests for these values per 1000 patients, the sample size as well as the number of studies which contributed to the sample, the GRADE (Grading of Recommendations Assessment, Development and Evaluation) quality of evidence for each finding, and any comments (including decisions as to why the reviewers assigned the final GRADE ranking) (Schünemann et al. 2013). These Summary of Findings tables can be created using the software program Guideline Development Tool (GDT, http://www.guidelinedevelopment.org/) and should appear in the Executive Summary section in JBI systematic reviews, following Implications for Research.

To determine a GRADE ranking of the evidence, the GRADE approach begins by assigning a starting level of quality to findings. For studies of diagnostic test accuracy, cross-sectional or cohort studies are considered to provide ‘high quality’ evidence, whereas for other quantitative studies they are ‘low’. There are two other levels in the GRADE systems, with four levels in total. These are high, moderate, low, and very low (Schünemann et al. 2013; Gopalakrishna et al. 2014; Atkins et al. 2004).

Different factors are then considered that lead to downgrading the GRADE ranking. These are: Risk of bias (as determined by critical appraisal; -1 if serious risk of bias, -2 if very serious risk of bias), Inconsistency or heterogeneity of evidence (-1 if serious inconsistency, -2 if very serious inconsistency), Indirectness of evidence (-1 if serious, -2 if very serious), Imprecision of results (-1 if wide confidence interval, -2 if very wide confidence interval) and Publication bias (-1 if likely, -2 if very likely) (Schünemann et al. 2013; Gopalakrishna et al. 2014; Atkins et al. 2004).

For other review types there are factors which can increase the GRADE quality of evidence (i.e. large magnitude of effect, dose response, all plausible confounding factors would reduce the demonstrated effect, or create a spurious effect where results suggest no effect). However, no such factors have been endorsed for studies of diagnostic test accuracy. For further guidance on the GRADE approach visit the GRADE working group website.

Table 9.6: Summary of Findings template

Test result	Number of results per 1000 patients tested (95% CI)		Number of participants (Studies)	Quality of the evidence (GRADE)	Comments
	Prevalence 0%				
	[index test]	[comparator test]			
True positives (patients with [target condition])					
	TP absolute difference: 0 more				
False negatives (patients incorrectly classified as not having [target condition])					
	FN absolute difference: 0 more				
True negatives (patients without [target condition])					
	TP absolute difference: 0 more				
False positives (patients incorrectly classified as having [target condition])					
	FP absolute difference: 0 more				

JBI endorses GDT for the development of Summary of Findings tables. All Summary of Findings tables created for JBI DTA reviews must use the GDT software.

For peer review only

When developing a Summary of Findings table within GDT, there are different format options for exporting the table. JBI reviews must use the Summary of Findings table (layer one) option (Table 9.6).

For peer review only

9.4.3 Background

The background section of the systematic review report should cover all the main elements of the topic under review. The Background section prepared for the protocol generally makes a good starting point; however it will often need an extension or modification following the review. The Background should detail any definitions important to the review. The information in the Background section must be sufficient to put the inclusion criteria in context. Reasons for investigating the index test, as well as the choice of reference test should be a particular area of focus.

At the conclusion of the Background section there should be a statement that a preliminary search for previous systematic reviews on the topic has been conducted (state the databases searched, e.g. *JBI Evidence Synthesis*, Cochrane Library, CINAHL, PubMed). If a previous systematic review has been found, it should be specified how the conducted review is different from the previous one. JBI places significant emphasis on a comprehensive, clear and meaningful background section to every systematic review, particularly given the international circulation of systematic reviews, variation in local understandings of clinical practice, health service management and client or patient experiences. It is recommended that all JBI systematic reviews contain a sentence clearly indicating:

“The objectives, inclusion criteria and methods of analysis for this review were specified in advance and documented in an a priori published protocol. Ref” (the reference should be to the appropriate citation in *JBI Evidence Synthesis*).

This sentence should appear as the final line of the background/introduction section of the systematic review report and complies with the recommendations for reporting of systematic reviews detailed in the PRISMA guidelines.

For peer review only

9.4.4 Methods

Review objectives and review questions

The review objectives should be the same as stated in the protocol (aside from tense adjustments). As discussed previously they should be followed by the specific questions.

Inclusion criteria

The inclusion criteria should be the same as described in the protocol (PIRD: population, index test, reference test, diagnosis of interest). They should be as clear and as unambiguous as possible.

Search strategy

This section should report on how the reviewers searched for relevant papers. The databases that were searched must be listed along with the search dates. This should be the same as described in the protocol. A detailed search strategy for all of the major databases and other sources searched should be appended to the review. The documentation of search strategies is a key element of the scientific validity of a systematic review as it enables readers to look at and evaluate the search strategy.

Assessment of methodological quality

This section should detail the methodology followed for critical appraisal in the systematic review, including the criteria used to determine the inclusion or exclusion of studies. The process described should be the same as that specified in the protocol, with reasons for deviation given. The critical appraisal tool should be appended to the review.

Data extraction

This section should detail the types of data extracted from the included studies, which should be the same as those specified in the protocol. The data extraction tool used to facilitate this process should be appended to the review.

Data synthesis

This section details the data synthesis approach, as opposed to the results of the synthesis itself. The protocol should have specified which methods of synthesis (narrative, graphical, tabular, meta-analysis) would be considered and under which circumstances. This section should detail the actual method used along with why it has been chosen (i.e. if narrative synthesis is chosen over meta-analysis due to the presence of heterogeneity, this should be explained along with the factors that are causing the studies to be heterogeneous). If a meta-analysis is performed, the statistical software should be specified.

For peer review only

9.4.5 Results

The results section should begin with a summary of the process followed from the search to the final selection of studies for extraction and synthesis, including how many articles have been included or excluded at each stage. This should be accompanied by a flow chart conforming to the PRISMA statement (Figure 9.4) (Liberati et al. 2009).40 Lists of included and excluded studies should be included as separate appendices in the systematic review report. It is important that all studies excluded at and from the 'full text review' stage should have their reason for exclusion given as a part of this list.

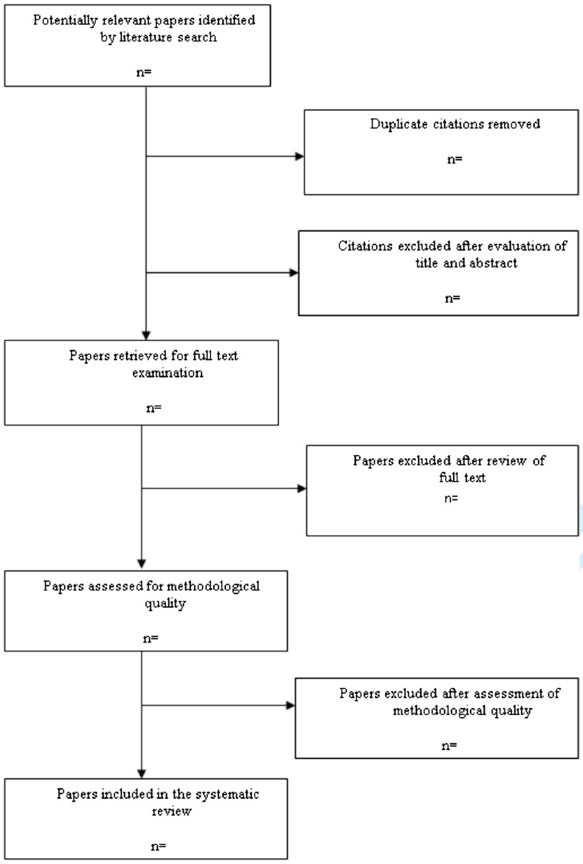

Figure 9.4: Flowchart detailing identification and selection of studies for inclusion in the review

Description of studies

To provide a context for the findings of the review, the results section should also include an overall description of the included studies. This should provide sufficient detail for the readers to assess how similar the studies are to one another, with a view to informing the appropriateness of meta-analysis. Specific items of interest from the studies may also be highlighted here. These may include: characteristics of the participants, the settings in which the tests have been conducted and specific study designs used. Tables are the most appropriate form for presenting this data, and the use of appendices should also be considered. The presence of extensive detail on study characteristics may obscure the actual findings, and make them less accessible to the reader.

Methodological quality

This section should detail the methodological quality of the included studies, as determined by the critical appraisal checklist used. It should include a narrative summary of the overall methodological quality of the included studies, which may be directly supported by a table showing the results of the critical appraisal (see Table 9.7 for example; if this table is not included in the results it should be included in the appendix). If any studies have been excluded due to critical appraisal, this is an appropriate area to provide justification.

Table 9.7: Critical appraisal results for included studies using the JBI critical appraisal checklist

For peer review only

Study	Q1	Q2	Q3	Q4	Q5	Q6	Q7	Q8	Q9	Q10
Author(s) ref	Y	Y	Y	N	Y	U	Y	N	Y	U

Y - Yes, N - No, U - Unclear

Findings of the review

There is no accepted standard for the structure for reporting the findings of systematic reviews; however it is recommended that findings be presented in the same order as the relevant review questions in order to create a logical flow. Again, the use of tables and appendices should be considered in order to avoid obscuring important details with an excess of less important items. As a general rule, findings are discussed textually and then supported with meta-graphs, tables and figures as appropriate.

For peer review only

9.4.6 Discussion

The discussion section should focus on considering the results in light of the review objectives, as well as how the review findings will influence the course of diagnosis in the area of the review. Specifically, the effects of the review findings on the field of diagnostics related to the test(s) under review, as well as their influence on patients and other relevant issues, should be considered.

Conclusion

The discussion should also include a final overview of the results that address any issues arising from the review's conduct, including any limitations as well as issues arising from the results of the review. Recommendations for practice and research should also be made.

Recommendations for practice

Recommendations for practice should be detailed, specific and based on documented results, not reviewer opinion. Where the results of the review do not support any specific recommendation for practice this should be noted.

Recommendations for research

Recommendations for research should be derived from the results of the review and based on identified gaps in the literature or methodological weakness. Generalized statements calling for further research should be avoided in favor of the identification of specific issues. Where the findings of a review suggest that no further research be performed (saturation may be apparent, or a test may have been identified as containing an unacceptable risk), this should be noted as a recommendation.

For peer review only

9.4.7 References

All references should be listed in full using the Vancouver referencing style, in the order in which they appear in the review. The references should be appropriate in content and volume and include background references and studies from the initial search.

For peer review only

9.4.8 Appendices

Appendices should be numbered using Roman numerals in the order in which they are referred to in the body of the text. There are several required appendices for a JBI review:

Appendix I: Search strategy

A detailed and complete search strategy for all of the major databases and other sites and sources searched must be appended. Major databases that were searched must be identified, including the search platform used where necessary. All search filters with logic employed should be displayed, including the number of records returned.

Appendix II: Data extraction template

The data extraction template used must be appended. Appendix III: Table of included studies

A table of included studies is crucial to allow a snapshot of the studies included in the review. Appendix IV: List of excluded studies

At a minimum, a list of studies excluded at the critical appraisal stage must be appended and reasons for exclusion be provided for each study (these reasons should relate to the methodological quality of the study, not study selection). Studies excluded following examination of the full-text may also be listed along with their reason for exclusion at that stage (i.e. a mismatch with the inclusion criteria). This may be as a separate appendix or itemized in some fashion within the one appendix.

Other appendices should be included in the order that they were referred to in the review.

For peer review only

9.5 Chaper references

Altman DG, Bland JM. Diagnostic tests 1: Sensitivity and specificity. Bmj. 1994;308(6943):1552.

Altman DG, Bland JM. Diagnostic tests 2: Predictive values. Bmj. 1994b;309(6947):102.

Atkins D, Best D, Briss PA et al. Grading quality of evidence and strength of recommendations. Bmj. 2004;328(7454):1490.

Beynon R, Leeflang MM, McDonald S et al. Search strategies to identify diagnostic accuracy studies in MEDLINE and EMBASE. The Cochrane database of systematic reviews. 2013;9:MR000022.

Bossuyt P, Reitsma, J., Bruns, D., Gatsonis, C., Gatsonis, C., Irwig, L., Lijmer, J., Moher, D., Rennie, D. & De Vet, H. Towards complete and accurate reporting of studies of diagnostic accuracy: The STARD initiative. Croat Med J. 2003;138:40-4.

Brenner H, Gefeller O. Variation of sensitivity, specificity, likelihood ratios and predictive values with disease prevalence. Stat Med. 1997;16(9):981-91.

Campbell JM, Kluger M, Ding S, Carmody DP, Hakonsen SJ, Jadotte YT, White S, Munn Z. Diagnostic test accuracy: Methods for Systematic Review and Meta-analysis. IJEBH. 2015.

Chu H, Cole SR. Bivariate meta-analysis of sensitivity and specificity with sparse data: a generalized linear mixed model approach. J Clin Epidemiol. 2006;59(12):1331-2; author reply 2-3.

de Vet H, Eisinga A, Riphagen I, Aertgeerts B, Pewsner D. Chapter 7: Searching for Studies. In: Deeks J, Bossuyt P, Gatsonis C, eds. Cochrane Handbook for Systematic Reviews of Diagnostic Test Accuracy: The Cochrane Collaboration 2008.

Deeks JJ. Systematic reviews in health care: Systematic reviews of evaluations of diagnostic and screening tests. Bmj. 2001;323(7305):157-62.

Dinnes J, Deeks J, Kirby J, Roderick P. A methodological review of how heterogeneity has been examined in systematic reviews of diagnostic test accuracy. Health Technol Assess. 2005;9(12):1-113, iii.

Erol B, Gulpinar MT, Bozdogan G et al. The cutoff level of free/total prostate specific antigen (f/t PSA) ratios in the diagnosis of prostate cancer: A validation study on a Turkish patient population in different age categories. Kaohsiung J Med Sci. 2014;30(11):545-50.

Eusebi P, Reitsma JB, Vermunt JK. Latent class bivariate model for the meta-analysis of diagnostic test accuracy studies. BMC Med Res Methodol. 2014;14:88.

Gatsonis C. Do we need a checklist for reporting the results of diagnostic test evaluations? The STARD proposal. Acad Radiol. 2003;10(6):599-600.

Gopalakrishna G, Mustafa RA, Davenport C et al. Applying Grading of Recommendations Assessment, Development and Evaluation (GRADE) to diagnostic tests was challenging but doable. J Clin Epidemiol. 2014;67(7):760-8.

Habbema J, Eijkemans R, Krijnen P, Knottnerus J. Analysis of data on the accuracy of diagnostic tests. In: Knottnerus J, Buntinx F, eds. The Evidence Base of Clinical Diagnosis: Theory and methods of diagnostic research. 2nd ed. London: BMJ Publishing Group 2009:118-45.

Hanley JA, McNeil BJ. The meaning and use of the area under a receiver operating characteristic (ROC) curve. Radiology. 1982;143(1):29-36.

Harbord RM, Whiting P, Sterne JAC et al. An empirical comparison of methods for meta-analysis of diagnostic accuracy showed hierarchical models are necessary. J Clin Epidemiol. 2008;61(11):1095-103.

Holling H, Bohning W, Bohning D. Meta-analysis of diagnostic studies based upon SROC-curves: a mixed model approach using the Lehmann family. Stat Model. 2012;12(4):347-75.

Lalkhen AG, McCluskey A. Clinical tests: sensitivity and specificity. Continuing Education in Anaesthesia, Critical Care & Pain. 2008;8(6):221-3.

Lau J, Ioannidis JP, Schmid CH. Quantitative synthesis in systematic reviews. Ann Intern Med. 1997;127 (9):820-6.

Leeflang MM, Bossuyt PM, Irwig L. Diagnostic test accuracy may vary with prevalence: implications for evidence-based diagnosis. J Clin Epidemiol. 2009;62(1):5-12.

Leeflang MM, Rutjes AW, Reitsma JB, Hooft L, Bossuyt PM. Variation of a test's sensitivity and specificity with disease prevalence. CMAJ. 2013b;185(11):E537-44.

Leeflang MM, Deeks JJ, Takwoingi Y, Macaskill P. Cochrane diagnostic test accuracy reviews. Syst Rev. 2013;2:82.

Leeflang MM. Systematic reviews and meta-analyses of diagnostic test accuracy. Clin Microbiol Infect.

For peer review only - http://bmjopen.bmj.com/site/about/guidelines.xhtml

2014;20(2):105-13

Liberati A, Altman DG, Tetzlaff J et al. The PRISMA statement for reporting systematic reviews and meta-analyses of studies that evaluate healthcare interventions: explanation and elaboration. Bmj. 2009;339: b2700.

Littenberg B, Moses LE, Rabinowitz D. Estimating Diagnostic-Accuracy from Multiple Conflicting

Reports - a New Meta-Analytic Method. Med Decis Making. 1990;38(2):A415-A.

Macaskill P, Gatsonis C, Deeks J, Harbord R, Takwoingi Y. Chapter 10: Analysing and presenting results. In: Deeks J, Bossuyt P, Gatsonis C, eds. Cochrane Handbook for Systematic Reviews of Diagnostic Test Accuracy: The Cochrane Collaboration 2010.

McGee S. Simplifying likelihood ratios. J Gen Intern Med. 2002;17(8):647-50.

Metz CE. Basic principles of ROC analysis. Semin Nucl Med. 1978;8(4):283-98.

Meyer G. Guidelines for reporting information in studies of diagnostic accuracy: The STARD initiative. J Pers Assess. 2003;81:191-3.

Moses LE, Shapiro D, Littenberg B. Combining Independent Studies of a Diagnostic-Test into a Summary Roc Curve - Data-Analytic Approaches and Some Additional Considerations. Stat Med.1993;12 (14):1293-316.

Mulligan EP, Harwell JL, Robertson WJ. Reliability and diagnostic accuracy of the Lachman test performed in a prone position. J Orthop Sports Phys Ther. 2011;41(10):749-57.

Sackett DL, Haynes RB. The architecture of diagnostic research. Bmj. 2002;324(7336):539-41.

Schünemann H BJ, Guyatt G, Oxman A, editors. 7. The GRADE approach for diagnostic tests and strategies. In: The GRADE Working Group, ed. GRADE handbook for grading quality of evidence and strength of recommendations 2013.

Reitsma J, Whiting, P., Vlassov, V., Leeflang, M. & Deeks, J. Chapter 9: Assessing methodological quality. . In: J D, ed. Cochrane Handbook for Systematic Reviews of Diagnostic Test Accuracy. York: The Cochrane Collaboration. 2009.

White S, Schultz, T., Enuameh, YAK., ed. Synthesizing evidence of diagnostic accuracy. Philadelphia, USA: Lippincott Williams and Williams 2011.

Whiting P, Rutjes AW, Dinnes J, Reitsma J, Bossuyt PM, Kleijnen J. Development and validation of methods for assessing the quality of diagnostic accuracy studies. Health Technol Assess. 2004;8(25):iii, 1-234.

Whiting P, Rutjes, AWS., Westwood,ME., Mallett, S., Deeks, JJ., Reitsma,JB., Leeflang, MMG., Sterne, JAC., Bossuyt, PMM., the QUADAS-2 Group. QUADAS-2: a revised tool for the quality assessment of diagnostic accuracy studies. Ann Intern Med. 2011;155::529e36.

Zou KH, O'Malley AJ, Mauri L. Receiver-operating characteristic analysis for evaluating diagnostic tests and predictive models. Circulation. 2007;115(5):654-7.

For peer review only

Appendix 9.1 Critical appraisal checklist

JBI Critical Appraisal Checklist for Diagnostic Test Accuracy Studies

Reviewer____Date____________________________

Author ____Year_________Record Number______

	Yes	No	Unclear	Not applicable
1. Was a consecutive or random sample of patients enrolled?	☐	☐	☐	☐
2. Was a case-control design avoided?	☐	☐	☐	☐
3. Did the study avoid inappropriate exclusions?	☐	☐	☐	☐
4. Were the index test results interpreted without knowledge of the results of the reference standard?	☐	☐	☐	☐
5. If a threshold was used, was it pre-specified?	☐	☐	☐	☐
6. Is the reference standard likely to correctly classify the target condition?	☐	☐	☐	☐
7. Were the reference standard results interpreted without knowledge of the results of the index test?	☐	☐	☐	☐
8. Was there an appropriate interval between index test and reference standard?	☐	☐	☐	☐
9. Did all patients receive the same reference standard?	☐	☐	☐	☐
10. Were all patients included in the analysis?	☐	☐	☐	☐

Overall appraisal: Include ☐ Exclude ☐ Seek further info ☐

Comments (Including reason for exclusion)

____

____

____

Explanation of diagnostic test accuracy studies critical appraisal

Diagnostic Test Accuracy Studies Critical Appraisal Tool

Answers: Yes, No, Unclear or Not/Applicable

Patient selection

1. Was a consecutive or random sample of patients enrolled?

Studies should state or describe their method of enrolment. If it is claimed that a random sample was chosen the method of randomization should be stated (and appropriate). It is acceptable if studies do not say 'consecutive' but instead describe consecutive enrolment; i.e. 'all patients from …. till …. were included'.

2. Was a case-control design avoided?

Case control studies are described in detail in the reviewers manual. In essence, if a study design involves recruiting participants who are already known by other means to have the diagnosis of interest and investigating whether the test of interest correctly identifies them as such, the answer is 'No'.

3. Did the study avoid inappropriate exclusions?

If patients are excluded for reasons that would likely influence the conduct, interpretation or results of the test, this may bias the results. Examples include: excluding patients on which the test is difficult to conduct, excluding patients with borderline results, excluding patients with clear clinical indicators of the diagnosis of interest.

Index test

For peer review only

4. Were the index test results interpreted without knowledge of the results of the reference standard?

The results of the index test should be interpreted by someone who is blind to the results of the reference test. The reference test may not have been conducted at the point that the index test is carried out, if so the answer to this question will be 'Yes'. If the person who interprets the index test also interpreted the reference test then it is assumed that this question will be answered 'No' unless there are other factors in play (for instance, the interpretation of the results may be separate from their collection, in which case the interpreter may be blinded to patient identity and past reference test results).

5. If a threshold was used, was it pre-specified?

Diagnostic thresholds may be chosen based on what gives the optimum accuracy from the data, or they may be pre-specified. When no diagnostic threshold is applied (i.e. the results of a test is based on the observation of a specific characteristic which is either there or not) this question will be answered NA.

Reference test

6. Is the reference standard likely to correctly classify the target condition?

The reference test should be the gold standard for the diagnosis of the condition of interest. Additionally, the reporting of the study should describe its conduct in sufficient detail that the reviewers can be confident that it has been correctly and competently implemented.

7. Were the reference standard results interpreted without knowledge of the results of the index test?

The points made for criteria 4 apply equally here. The results of the reference test should be interpreted by someone who is blind to the results of the index test. The index test may not have been conducted at the point that the reference test is carried out, if so the answer to this question will be 'Yes'. If the person who interprets the reference test also interpreted the index test then it is assumed that this question will be answered 'No' unless there are other factors in play (for instance, the interpretation of the results may be separate from their collection, in which case the interpreter may be blinded to patient identity and past index test results).

Flow and timing

8. Was there an appropriate interval between the index test and the reference standard?

The index test and the reference test should be carried out close enough together that the status of the patient could not have meaningfully changed. The maximum acceptable time will vary based on characteristics of the population and condition of interest.

9. Did all patients receive the same reference standard?

The reference standard by which patients are classed as having or not having the condition of interest should be the same for all patients. If the results of the index test influence how or whether the reference test is used (i.e. where an apparent false negative may be detected the study design may call for a 'double check') this may result in biased estimates of sensitivity and specificity. Additionally, in some studies two parallel reference tests may be used (on different patients) and the results then pooled. In either case the results should be 'No'.

10. Were all patients included in the analysis?

Loses to follow up should be explained and there cause and frequency should be considered in whether they are likely to have had an effect on the results (Subjectivity may exist in this context, overall low tolerance should be applied in deciding to answer 'No' to this question, but a single withdrawal from a large cohort should not necessarily force a negative response). However, if a patients' results being difficult to interpret results in their data being excluded from the analysis this will result in an exaggerated estimate of DTA, and this question should definitely be answered 'No'.

This tool is based on and largely informed and taken from the QUADAS-2 approach.

Whiting, Penny F., et al. "QUADAS-2: a revised tool for the quality assessment of diagnostic accuracy studies." Annals of internal medicine 155.8 (2011): 529-536.

For peer review only - http://bmjopen.bmj.com/site/about/guidelines.xhtml

Appendix 9.2 Data extraction instrument

Author/Date	
Inclusion/exclusion criteria: i.e. presenting symptoms, results from previous tests	Inclusion:<br>Exclusion:
Sample size	
Participant demographics (i.e. age, sex, spectrum of presenting symptoms, comorbidity, current treatments, recruitment centres)	
Study methodology (consecutive or random;<br>retrospective or prospective)	
Period that study was carried out (beginning and end date)	
Index test description (including criteria for positive test)	
Reference test description (including criteria for positive test)	
Geographical location of data collection	
Setting of data collection	
Persons executing and interpreting index tests<br>(numbers, training, and expertise)	
Persons executing and interpreting reference test	
Index/reference time interval (and treatments carried out in between)	
Distribution of severity of disease in those with target condition	
Other diagnoses in those without target condition	
Adverse events from index test	
Adverse events from reference test	

Index test results<br>Threshold=	Condition positive	Condition negative	Total
Index test positive (T+)			
Index test negative (T-)			
Total			

For peer review only

Appendix 9.3 Meta-analysis equations and models

The Moses-Littenberg model

The models are explained below and their formulas are issued from Macaskill et al. 2010, unless otherwise specified.

The method underlying the Moses-Littenberg model is based on a linear regression describing the variation of the test accuracy in function of the positivity threshold. It can be written as:

$$D = \propto + \beta S + error$$

The test accuracy is defined by the logit of the diagnostic odds ratio (D) following:

$$D = logit(sensitivity) - logit(1 - specificity)$$

The estimation of the positivity threshold (S) is:

$$S = logit(sensitivity) + logit(1 - specificity)$$

The linear regression model, describing the variation of the test accuracy in function of the positivity threshold, can be written as:

$$D = \propto + \beta S + error$$

This equation provides, through least squares method, values for which are then used to estimate sensitivity values for chosen specificities, with:

$$E(sensitivity) = 1/[1 + \exp(-\frac{[\propto + (1+\beta) logit(1 - specificity)]}{1-\beta})]$$

Usually, the chosen values of specificities are the one issued from the literature.

The bivariate model

The number of test positives in study i is defined according to:

$y_{Ai} \sim \text{Binomial}(n_{Ai}, \pi_{Ai})$ for sensitivity, and the number testing negative following:

$y_{Bi} \sim \text{Binomial}(n_{Bi}, \pi_{Bi})$ for specificity, with n_{Ai} and n_{Bi} respectively the number of diseased/control subjects in the study i and π_{Ai} and π_{Bi} respectively the probability of a positive/negative test in the respective group of the study i.

For the variability between studies, the logit-transformed sensitivity is treated with a normal distribution characterised by a mean μ_A and a variance σ_A^2. Similarly, the normal distribution of the logit-transformed specificity is defined by the mean μ_B and a variance σ_B^2. The correlation between these two components is integrated in a bivariate normal model, written as:

$\begin{pmatrix}\mu_{A,i}\\ \mu_{B,i}\end{pmatrix} \sim \text{Normal}\left(\begin{pmatrix}\mu_A\\ \mu_B\end{pmatrix}, \Sigma\right)$ where $\Sigma = \begin{pmatrix}\sigma_A^2 & \sigma_{AB}\\ \sigma_{AB} & \sigma_B^2\end{pmatrix}$. The term σ_{AB} expresses the covariance between logit sensitivity and specificity.

The HSROC Model

The model of Rutter and Gatsonis is based on hierarchical regression to estimate variations at the within studies level as well as at the between studies one.

For peer review only

At the within studies level, binomial distributions are assumed for the number of positive individuals in the diseased (y_{i1}) and control groups (y_{i2}) of study i. They are written as:

$y_{ij} \sim \text{Binomial}(n_{ij}, \pi_{ij})$ with n_{ij} the sample size of tested individuals and π_{ij} the probability of a positive test. Accordingly, the probability of a positive test is determined simultaneously for diseased and control groups, following:

$$\text{logit}(\pi_{ij}) = (\theta_i + \alpha_i dis_{ij})\exp(-\beta dis_{ij})$$

with θ_i modelling the positivity threshold, α_i the diagnostic accuracy of study i, $\mathbf{dis_{ij}}$ the status (diseased vs. control) for a patient of the ith study, and β called the scaled parameter permitting variation of accuracy with threshold. This engenders ROC curve with potential asymmetry. β is considered as a fixed effect.

The between-studies variability is treated with two normal distributions. One for the threshold, characterised by a mean Θ (capital theta) and a variance σ_β^2, and the other for diagnostic accuracy with parameters of mean Λ (capital lambda) and variance σ_α^2.

Based on the above-mentioned estimated parameters, a SROC curve can be plotted at given values of specificity.

$$sensitivity = 1/\{1 + \exp[-(\Lambda e^{-0.5\beta} + logit(1 - sensitivity)e^{-\beta})]\}$$

This equation is issued from Macaskill et al. 2004.

As expected, when $\beta = 0$, the SROC curve will be symmetric.

For peer review only

Appendix 9.4 Examples of databases

Databases of published literature

Nursing and allied health

- *Allied and Complementary Medicine (AMED):*

(http://www.ebscohost.com/academic/AMED-The-Allied-and-Complementary-Medicine- Database)

- *British Nursing Index (BNI):*

(www.bniplus.co.uk/)

- *Cumulative Index to Nursing and Allied Health (CINAHL):*

(www.cinahl.com/)

Primary care

- *Essential Evidence Plus (formerly Patient Oriented Evidence that Matters (InfoPOEMs)):*

(www.essentialevidenceplus.com/)

Social science, psychology and psychiatry

- *Applied Social Sciences Index and Abstracts (ASSIA):*

(http://www.proquest.com/products-services/ASSIA-Applied-Social-Sciences-Index-and- Abstracts.html)

- *PsycINFO:*

(www.apa.org/psycinfo/)

- *Sociological Abstracts:*

(http://proquest.libguides.com/SocAbs)

Biology and chemistry

- *Biological Abstracts / BIOSIS Previews:*

(http://thomsonreuters.com/biosis-previews/)

- *Chemical Abstracts:*

(www.cas.org/)

- *Database of the International Federation of Clinical Chemistry and Laboratory Medicine*

- Committee for Evidence-based Laboratory Medicine (IFCC C-EBLM database) (contact j.watine@ch-rodez.fr)

International health

- *Global Health:*

Available via: (www.cabi.org)

In addition to subject-specific databases, general search engines include:

For peer review only

- *Google Scholar (free on the internet):*

(scholar.google.com/advanced_scholar_search)

- *Turning Research into Practice (TRIP) database (evidence-based healthcare resource)*

(free on the internet): (www.tripdatabase.com/)

"Citation searching"

Citation searching is an important and effective adjunct to database searching and hand searching. Information about these citation indexes is available at: Cochrane handbook

- *Science Citation Index:*

scientific.thomson.com/products/sci/

- *Social Sciences Citation Index:*

scientific.thomson.com/products/ssci/

- *Web of Science:*

scientific.thomson.com/products/wos/

- *Web of Knowledge:*

isiwebofknowledge.com/

- *Scopus:*

http://www.elsevier.com/online-tools/scopus

Theses specific databases

- *ProQuest Dissertations & Theses Database:*

www.proquest.co.uk/products_pq/descriptions/pqdt.shtml

- *Dissertation Abstracts Online (DIALOG)*

- *Index to Theses in Great Britain and Ireland*

www.theses.com/

- *DissOnline: indexes 50,000 German dissertations:*

www.dissonline.de/

Grey literature databases

- *MedNar*

mednar.com/mednar

- *OpenSIGLE*

http://www.greynet.org/opensiglerepository.html

- *National Technical Information Service (NTIS)*

www.ntis.gov/

- *WorldWideScience.org*

worldwidescience.org/index

- *Open Grey*

http://www.opengrey.eu/

For peer review only

Chapter 10: Umbrella reviews

Edoardo Aromataris, Ritin Fernandez, Christina Godfrey, Cheryl Holly, Hanan Khalil, Patraporn Tungpunkom

How to cite:

Aromataris E, Fernandez R, Godfrey C, Holly C, Khalil H, Tungpunkom P. Chapter 10: Umbrella Reviews. In: Aromataris E, Munn Z (Editors). *JBI Manual for Evidence Synthesis.* JBI, 2020. Available from https://synthesismanual.jbi.global. https://doi.org/10.46658/JBIMES-20-11

Chapter 10: Contents

- 10.1 Umbrella reviews and evidence-based practice
  - 10.1.1 - Why an umbrella review?
  - 10.1.2 - Not just effectiveness - JBI umbrella reviews
- 10.2 Development of an Umbrella review protocol
  - 10.2.1 Title and author information
  - 10.2.2 Developing the title and question
  - 10.2.3 Introduction
  - 10.2.4 Review question(s)
  - 10.2.5 Inclusion criteria
  - 10.2.6 Search Strategy
  - 10.2.7 Study Selection
  - 10.2.8 Assessment of methodological quality
  - 10.2.9 Data collection
  - 10.2.10 Data summary
- 10.3 Umbrella Review and Summary of the evidence of research syntheses
  - 10.3.1 Title of the Umbrella Review
  - 10.3.2 Review Authors
  - 10.3.3 Abstract
  - 10.3.4 Introduction
  - 10.3.5 Review question(s)
  - 10.3.6 Inclusion criteria
  - 10.3.7 Methods
    - 10.3.7.1 Search strategy
    - 10.3.7.2 Study screening and selection
    - 10.3.7.3 Assessment of methodological quality/critical appraisal
    - 10.3.7.4 Data collection
    - 10.3.7.5 Data Summary
  - 10.3.8 Results
    - 10.3.8.1 Study inclusion
    - 10.3.8.2 Methodological quality
    - 10.3.8.3 Characteristics of included studies
    - 10.3.8.4 Findings of the review
  - 10.3.9 Summary of Evidence
  - 10.3.10 Discussion
  - 10.3.11 Conclusions and recommendations
  - 10.3.12 Conflicts and acknowledgements
  - 10.3.13 Review Appendices
- 10.4 Chapter references
- Appendix 10.1 JBI Critical Appraisal Checklist for Systematic reviews and Research Syntheses
- Appendix 10.2. Discussion of JBI Critical Appraisal Checklist for systematic reviews and research syntheses
- Appendix 10.3 JBI Data Extraction Form for Review for Systematic Reviews and Research Syntheses

For peer review only

10.1 Umbrella reviews and evidence-based practice

The volume of literature pertinent to healthcare is growing at an increasing rate with thousands of studies published annually. Systematic reviews in healthcare have evolved in large part out of the recognition that this overwhelming amount of research evidence makes it difficult for decision makers to utilize the best available evidence to inform their decision making. Systematic reviews involve a rigorous scientific approach to an existing body of research evidence in an attempt to identify original research, critically appraise eligible studies and summarize and synthesize the results of high quality research ultimately informing in a single manuscript.

A number of country-specific organizations, including the Agency for Healthcare Research and Quality (AHRQ) in the USA, the National Institute for Healthcare Excellence (NICE) in the UK, and international organizations such as Cochrane and JBI have dedicated themselves to the production of systematic reviews to inform healthcare policy and practice. In doing so, these organizations have contributed to the growing number of systematic reviews that have been published in recent years. Consequently, the number of systematic reviews published is, as with the bulk of scientific literature, also increasing at a phenomenal rate and now risks compounding the problem already faced by healthcare decision makers in sorting through multitudes of evidence to inform their questions. Bastian et al (2010) recently estimated that 11 systematic reviews were published every day! Still, decision making can be challenging for healthcare practitioners and policy makers, even with systematic reviews readily available. The purpose of this chapter is to provide guidance on a method of review that can address these issues. Called an Umbrella Review, this method of review is essentially an overview of existing systematic reviews.

For peer review only

10.1.1 - Why an umbrella review?

Considering the large numbers of systematic reviews and research syntheses available to inform many topics in health care, systematic reviews of existing reviews are now being undertaken to compare and contrast published reviews and to provide an overall examination of a body of information that is available for a given topic (Hartling et al. 2012).

Conduct of an Umbrella Review offers the possibility of addressing a broad scope of issues related to a topic of interest. The wide picture obtainable from the conduct of an Umbrella Review is also ideal in highlighting if the evidence base around a topic or question is consistent or if contradictory or discrepant findings exist, and in exploring and detailing the reasons why. Investigation of the evidence with an Umbrella Review allows assessment and consideration of whether reviewers addressing similar review questions independently observe similar results and arrive at generally similar conclusions. Reviews of systematic reviews are referred to by several different names in scientific literature as: umbrella reviews, overviews of reviews, reviews of reviews, a summary of systematic reviews and also a synthesis of reviews. In essence however they all have the same defining feature: a systematic review is the main and often sole “study type” that is considered for inclusion (Becker and Oxman 2011; Hartling et al. 2012; Smith et al, 2011).

For JBI syntheses of existing systematic reviews, the term “Umbrella Review” will be used. JBI Umbrella Reviews are designed to incorporate all types of syntheses of research evidence, including systematic reviews in their various forms (effectiveness, meta-aggregative, integrative, etc.) and meta-analyses.

Beyond the impetus for Umbrella Reviews which is driven by the sheer volume of systematic reviews being published, the need for “fast” evidence in reduced timeframes has also reinforced the attractiveness of undertaking such a review. Decision makers are increasingly required to make evidence informed policy decisions and often require evidence in short timeframes – as a result, “rapid reviews” are also appearing in research literature. Rapid reviews are essentially a streamlined approach to evidence synthesis in health care that attempt to accommodate an evidence informed decision as quickly as possible (Kangura et al, 2012). While the conduct of a rapid review may impinge on, or result in, undesirable modification of some of the processes required of a well- conducted systematic review, this may be alleviated to some extent by considering if any existing systematic reviews on the topic of interest are already available.

Using existing systematic reviews also reinforces the necessity for some measure of efficiency in scientific undertakings today. In short, if current, multiple, good quality, systematic reviews exist about a given topic or question, any reviewer should reconsider the need to conduct yet another review addressing the same issue. Rather, these may be the basis to conduct an Umbrella Review and summarize or synthesize the findings of systematic reviews already available.

For peer review only

10.1.2 - Not just effectiveness - JBI umbrella reviews

Similar to Cochrane, the JBI has historically focused on reviews that inform the effectiveness of an intervention or therapy; however the emphasis on “best available” evidence in JBI reviews of effectiveness has not been confined solely to randomized controlled trials and other experimental studies that occupy the uppermost levels of the evidence hierarchy.

JBI Umbrella Reviews are intended to compile evidence from multiple research syntheses. Any review author will recognize the advantage of having a good understanding of study design and research methodologies, whether quantitative or qualitative in nature. Similarly, it is recommended that reviewers intending or attempting to undertake a JBI Umbrella Review should have a good understanding of systematic reviews and the diversity and methodological nuances among the various types of reviews (and different organizations and authors that conduct them) before conducting an Umbrella Review themselves.

The reasons for conducting a JBI Umbrella Review are manifold. The principal reason is to summarize evidence from many research syntheses (Becker and Oxman 2011). These may include analyses of evidence of different interventions for the same problem or condition, or evidence from more than one research synthesis investigating the same intervention and condition but addressing and reporting on different outcomes. Similarly, a researcher or reviewer may wish to summarize more than one research synthesis for different conditions, problems or populations.3 The principle focus of a JBI Umbrella Review is to provide a summary of existing research syntheses related to a given topic or question and not to re-synthesize, for example, the results of existing reviews or syntheses with meta-analysis or meta-synthesis.

A reviewer familiar with the JBI methodology for the conduct of systematic review will appreciate that many questions that are asked in health care practice do not lend themselves directly to experimentation or gathering of numerical data to establish the answer regarding what the effectiveness or outcomes of a particular intervention. Rather, the questions are more of *how and why* interventions do or do not work, and how recipients of the intervention may experience them.

As a result, many JBI syntheses are of original qualitative research and apply a meta-aggregative approach to synthesis of qualitative data (see Chapter 2). Similarly, JBI Umbrella Reviews may find they inevitably ask questions that direct the reviewer predominantly to existing qualitative reviews. As with the combinations of PICO elements to organize the conduct an Umbrella Review mentioned above, the common denominator or feature across such multiple qualitative syntheses may be the population or subpopulation of interest, coupled with the context of the review question.

For peer review only

10.2 Development of an Umbrella review protocol

For peer review only

10.2.1 Title and author information

The title should be informative and give clear indication of the topic of the Umbrella Review. The title of a JBI Umbrella Review should always include the phrase "…:an Umbrella Review" to allow easy identification of the type of document it represents. The names of all reviewers, affiliations for each author including their JBI centre affiliations and email address for the corresponding author should be included.

For peer review only

10.2.2 Developing the title and question

Although the Umbrella Review may aim to examine existing research syntheses for different types of interventions or phenomena of interest with the same condition, or different outcomes for the same intervention or phenomena of interest, the PICO and PICo mnemonic should be used to generate a clear and meaningful title and question. Ideally, the title for a quantitative Umbrella Review may incorporate some of the PICO elements, including the Population, the Intervention, the Comparison and Outcome, and the PICo elements if considering a question or topic that lends itself to qualitative data, including the Population, the Phenomena of Interest and Context. If a JBI Umbrella Review intends to review both quantitative and qualitative systematic reviews, both the intervention and phenomena of interest need to be clearly specified in the protocol (see below). The title of the Umbrella Review protocol must be concise enough to reflect the interventions or the phenomena of interest as a whole; however, it should also be as descriptive as possible. If the Umbrella Review is examining an intervention used across different patient conditions or different interventions with the same patient condition, this should be further delineated in the inclusion criteria section. The PICO or PICo mnemonic can provide potential readers with a significant amount of information about the focus, scope and applicability of the Umbrella Review to their needs. The following are examples of Umbrella Review titles:

1. “Non-pharmacological management for aggressive behaviors in dementia: an Umbrella Review protocol”

2. “The experiences of caregivers who are living with and caring for persons with dementia: an Umbrella Review protocol”

As an illustration of the use of the PICO elements to aid in articulating the title of an Umbrella Review, note that in example 1, the population (dementia), the intervention (non-pharmacological management), and the outcome (aggressive behaviors) are clearly evident. In this example this appears as the title of an Umbrella Review that lends itself to the inclusion of systematic reviews of randomized controlled trials to inform the effectiveness of an intervention or therapy, or potentially a broader investigation of research syntheses, that not only explore effectiveness of interventions but also the experiences of patients that received these therapies and their acceptability. Such an approach to this Umbrella Review will provide a comprehensive picture of the available evidence on the topic.

Similarly, example 2, provides readers with a clear indication of the population (caregivers of persons dementia), the phenomena of interest (experiences of caregiving), and the context (living with and caring for) as well as the fact that it is Umbrella Review protocol of qualitative evidence.

For peer review only

10.2.3 Introduction

The introduction should be comprehensive and cover all the main elements of the topic under review. It should cover the extant knowledge addressing the question of the Umbrella Review. The reason for undertaking the Umbrella Review should be clearly stated together with the target audience and what the Umbrella Review is intended to inform.

The suggested length for the introduction of the review protocol is approximately 1000 words. The background should detail any definitions important to the topic of interest. The information in the introduction section must also be sufficient to put the inclusion criteria into context, including an indication that there are existing systematic reviews or research syntheses available on the topic, hence supporting the rationale to conduct an Umbrella Review. The introduction should conclude a statement that a preliminary search for existing Umbrella Reviews on the topic has been/will be conducted (state the databases searched or search platforms utilized e.g. Cochrane Library, CINAHL, PubMed, EPPI, Epistomonikos and PROSPERO where relevant). If there is an existing Umbrella Review or overview of systematic reviews available on the topic, a justification that specifies how the proposed review will differ from those already conducted and identified should be detailed.

The introduction should conclude with an overarching review objective that captures and aligns with the core elements/mnemonic of the inclusion criteria (e.g. PICO). The stated objective should clearly indicate what the review project is trying to achieve. The objective(s) may be broad and will be aligned to specific review question(s). For example, using the first title introduced above, the objectives or aims may be: To examine non-pharmacological interventions for the management of aggressive behaviors in elderly patients with dementia.

Vancouver style of referencing should be used throughout the protocol with superscript numbers without brackets, used for in-text citations

For peer review only

10.2.4 Review question(s)

The review question(s) must be clearly stated. The review question(s) should be consistent with the title and direct the development of the specific inclusion criteria from clearly identifiable PICO. For example, using the first title introduced above, the objectives or aims of this review would be: To examine non-pharmacological interventions for the management of aggressive behaviors in elderly patients with dementia.

An example of the corresponding questions for this review would be:

1. What are effective non-pharmacological interventions to manage aggressive behavior in elderly patients with dementia?; and

2. What are the experiences of dementia patients and their caregivers with the use of non-pharmacological interventions to manage aggressive behavior?

For peer review only

10.2.5 Inclusion criteria

For the purposes of an Umbrella Review, the term "studies" refers exclusively to syntheses of research evidence including systematic reviews and meta-analyses. The "Inclusion criteria" of the protocol detail the basis on which studies will be considered for inclusion into the Umbrella Review and should be clearly defined.

These criteria provide a guide for the reader to clearly understand what is proposed by the reviewers and, more importantly a guide for the reviewers themselves to base decisions about the studies to be included in the Umbrella Review.

Types of participants

Important characteristics of participants should be detailed, including age and other qualifying criteria that make them appropriate for the objectives of the Umbrella Review and match the review question. In the example question above these characteristics include elderly people with dementia. Umbrella Reviews that aim to encompass multiple population groups should define each group clearly. Justification for the inclusion or exclusion of participants should be explained. In many cases, defining characteristics of the participants for a review may also include details of the setting of interest such as acute care, primary health care, or the community.

Interventions/phenomena of interest

The interventions or phenomena of interest for an Umbrella Review should be defined in detail and should be congruent with the review objective and intervention(s) or the phenomena of interest. Umbrella Reviews that aim to address multiple interventions and treatments should define each potential intervention of interest clearly.

Outcomes

Outcomes of interest should be predefined in Umbrella Reviews that lend themselves to quantitative evidence. Outcomes should be relevant to the question of the Umbrella Review and also the important outcomes for the participant group of the review. Surrogate outcomes should be explained and presented where there is a clear association with patient relevant outcomes. To provide a balanced overview of the evidence base related to a particular topic and fully inform decision-making, an Umbrella Review should attempt to report both beneficial and adverse outcomes.

Context

Context will vary depending on the objective(s)/question(s) of the review. The context should be clearly defined and may include but is not limited to consideration of cultural factors such as geographic location, specific racial or gender based interests. In some cases, context may also encompass details about the specific setting (such as acute care, primary health care, or the community).

Types of studies

As mentioned at the outset, the unit of analysis for an Umbrella Review is a completed research synthesis; therefore, the types of studies included in an Umbrella Review are exclusively syntheses of existing research from systematic reviews (using internationally accepted methodologies) and meta-analyses. Research syntheses included in an Umbrella Review should represent syntheses of empirical research evidence. Due to the enormous range of "review" types and articles available in the literature (Grant and Booth, 2009), authors of Umbrella reviews will have to stipulate clearly which review types should be included *a priori* in the protocol. Reviews that incorporate theoretical studies or text and opinion as their primary source of evidence should not be included in a JBI Umbrella Review and should be listed as an explicit exclusion criterion in the protocol.

For peer review only

10.2.6 Search Strategy

The search for an Umbrella Review should aim to identify all research syntheses relevant to the review question. The protocol should provide a detailed strategy for locating research syntheses including the key terms to be used and the resources to be searched. Predefined search filters for reviews for various databases already exist and they are worthwhile investigating when developing the search strategy for the review. An example is the "systematic[sb]" search filter for PubMed. As many databases do not have a predefined search filter for review articles, in these cases, it is preferable to search with key terms such as "systematic" or "meta- analysis" across the title or abstract fields. Most authors will use these terms in the title of their publications to clearly identify the type of publication. Authors of JBI systematic reviews will be familiar with the recommendation to identify the document as a systematic review in the manuscript title to maximize the likelihood that it will be retrieved and read.

The search terms used should be broad enough to capture all relevant reviews. A three- phase search process should be used. First, initial keywords are identified followed by analysis of the text words contained in the title and abstract, and of the index terms to describe relevant reviews. The additional terms i.e., meta-analysis or systematic review need to be included in the key terms for searching. Second, database-specific search filters for each bibliographic citation database stipulated in the protocol are constructed, and finally the reference list of all included reviews should also be searched.

The search for systematic reviews rarely needs to extend prior to 1990 as there were very few systematic reviews published prior to that time (Smith et al, 2012). Essentially searching for the research syntheses conducted within the last five to ten years will yield original/primary research conducted 30+ years prior that has been included in the located reviews and research syntheses. As well as biomedical citation databases such as Medline, PubMed, EMBASE, and CINAHL, other sources to search include the major repositories of systematic reviews such as the *JBI Evidence Synthesis*, the *Cochrane Database of Systematic Reviews*, DARE and the PROSPERO register. The federated search engine Epistemonikos (www.epistemonikos.org/) that specifically targets research syntheses is also worthwhile using, particularly for initial searches. The databases searched for an Umbrella Review will depend on the review questions and objectives, for example, PEDro is a database indexing reviews relevant to physiotherapy, OTseeker, indexing reviews relevant to Occupational Therapy while BEME and the EPPI Centre Evidence Library are repositories of reviews relevant to education. Due to limitations of available resources, most JBI Umbrella Reviews will inevitably focus on including studies published in the English language. Where a review team has capacity, the search should ideally attempt to identify research syntheses published in any language and may expand the search to include databases that index languages other than English.

A comprehensive search for a JBI Umbrella Review should also encompass a search for gray literature or reports that are not commercially published. As decision makers are increasingly required to base their decisions on available evidence, more and more research syntheses are being commissioned by practitioners and health care policy makers in governments globally; as a result many reports available via government or organizational websites are syntheses of research evidence and may be eligible for inclusion in a JBI Umbrella Review. A JBI Umbrella Review should include a search of at least two or three relevant sources for "gray" reports.

For peer review only

10.2.7 Study Selection

The Umbrella review protocol should describe the process of study selection for all stages of selection (based on title and abstract examination; based on full text examination) and the procedures for solving disagreements between reviewers. Selection is performed based on inclusion criteria pre-specified in the review protocol. For any systematic review, study selection (both at title/abstract screening and full text screening) is performed by two or more reviewers, independently. Any disagreements are solved by consensus or by the decision of a third reviewer.

For peer review only

10.2.8 Assessment of methodological quality

Research syntheses that are eligible for inclusion in a JBI Umbrella Review must be assessed for methodological quality. Ideally, only high quality systematic reviews should be included in an Umbrella Review. There are a variety of checklists and tools available to assess research syntheses and systematic reviews. Most checklists use a series of criteria that can be scored as being "met" or "not met" or "unclear" and in some instances as "not applicable". The decision as to whether or not to include a study can be made based on meeting a pre-determined proportion of all criteria, or on certain criteria being met. It is also possible to weight certain criteria differently. Decisions about a scoring system or any cut-off for exclusion should be made in advance and agreed upon by all reviewers before critical appraisal commences. The protocol, therefore, should detail how selected research syntheses will be assessed for quality, e.g. use of a predetermined cut off score.

It is the JBI policy that all systematic reviews need to be critically appraised using the standard JBI critical appraisal instrument for Systematic reviews and Research Syntheses that is available in Appendix 10.1 of this chapter (further details regarding the appraisal questions can be found in Appendix 10.2). For a JBI Umbrella Review the assessment criteria are available for selection in the JBI SUMARI software. The tool is designed to be used with two independent reviewers conducting the critical appraisal of each research synthesis selected. Reviewers are blinded to each other's assessment and assessments can only be compared once initial appraisal of an article is completed by both reviewers. Where there is a lack of consensus, discussion between reviewers should occur. In some instances it may be appropriate to seek assistance from a third reviewer. The source of the JBI critical appraisal tool for research syntheses should be cited in the protocol (Aromataris et al., 2015).

For peer review only

10.2.9 Data collection

Data collection is the procedure for extracting relevant details and data from the included systematic reviews and meta-analyses for the Umbrella Review. To avoid risk of bias, the standardized JBI data extraction tools (see Appendix 10.3 of this chapter) should be used to extract the data from the included reviews. Reviewers should have discussed and piloted its use prior to launching into extraction of data for the Umbrella Review to maximize consistency and the likelihood that the relevant results are being identified and detailed sufficiently for the purposes of reporting in the Umbrella Review. Without some discussion and piloting, reviewers may interpret fields in the tool or their relevance to the Umbrella Review questions slightly differently; differences unearthed at the completion of extraction for the review will invariably create more, unnecessary work for the review team. Any additions or modifications to the data extraction tool that are demanded by the nature of review question should be reviewed through by all reviewers and discussed in detail before extracting the data independently. Any additions or modifications should be identified and submitted with the review protocol and approved for publication in the *JBI Evidence Synthesis* prior to use by any reviewer.

Guided by the data extraction tool, information regarding the citation details, the objectives of the included review, the participants, the setting and context, the number of databases sourced and searched, the date range of database searching, the date range of included studies that inform each outcome of interest, the number/types of studies/country of origin of primary research studies in the included research synthesis, the instrument used to appraise the primary studies in the research synthesis and the rating of their quality, the outcomes reported by the included reviews that are relevant to the Umbrella Review question, and the type of review and the method of synthesis/analysis employed to synthesize the evidence as well as any comments or notes the Umbrella review authors may have regarding any included study.

Importantly, specific details of the factor or issue of interest to the Umbrella Review; for example the range of interventions, phenomena of interest, population details or outcome differences should be extracted in detail with the key findings/results. Extraction for a JBI Umbrella Review should be conducted independently by two reviewers to further minimize the risk of error. The protocol must therefore describe how data will be extracted and include the JBI data extraction instruments for systematic reviews in the appendices of the protocol. Extraction and presentation of data for a JBI Umbrella Review should be limited to the results and findings presented by the included research syntheses; in this regard it is not recommended that the researchers conducting the Umbrella Review retrieve primary studies (original research) in an included systematic review, for example, to access extra data. It is unlikely that authors of a JBI Umbrella review will need to contact the authors of an included research synthesis as is often the norm when undertaking a JBI Systematic Review (see other Chapters of this Manual).

For peer review only

10.2.10 Data summary

The aim of the JBI Umbrella review is to present a summary of existing research syntheses relevant to a particular topic or question and not any further "meta-analysis" of the results of these publications. To this end, the results of all included studies should be presented to the reader to allow for a ready and easily interpretable overview of the findings.

In the Umbrella Review protocol the means by which the results of the reviews will be presented should be described in as much as detail as possible. Tabular presentation of findings is recommended when overall effect estimates extracted from systematic reviews or other similar numerical data are presented. Where quantitative data is being presented, the number of studies that inform the outcome, the number of participants (from included studies) and the heterogeneity of the results of included reviews should also be reported (Smith et al, 2011). Where the results of qualitative systematic reviews are included in the Umbrella Review, the final or overall synthesized findings from included reviews should be presented, also in tabular format and with enough relevant contextual information alongside each synthesized finding to ensure each is interpretable to the reader of the Umbrella Review. Clear indication of any overlaps of original research studies in each of the included research syntheses must also be presented in the JBI Umbrella Review. For example, if one study is included in multiple syntheses this must be indicated.

The Principles from Grading of Recommendations Assessment, Development, and Evaluation (GRADE) should be used for an overall assessment of the quality of evidence for each intervention or phenomena of interest. The GRADE concept is based on an assessment of the following criteria: quality of primary studies, design of primary studies, consistency and directness (Guyat et al, 2008).

For peer review only

10.3 Umbrella Review and Summary of the evidence of research syntheses

This section provides further guidance on components that should be included in the final report of an Umbrella Review and information that each component should contain. It illustrates how each component of the review is managed in JBI SUMARI. This section also provides a brief outline of the format and stylistic conventions for Umbrella Reviews to ensure the review meets publication criteria for the *JBI Evidence Synthesis*. For further information please refer to the Author Guidelines of the journal. Specifically, guidance is provided on the following components: outline of the report, inclusion criteria (i.e. PICO), search strategy, critical appraisal, data extraction, data synthesis, results and conclusions. All JBI Umbrella Reviews should be based on a peer reviewed Umbrella Review protocol that has been accepted for publication in the *JBI Evidence Synthesis*. Deviations from a published review protocol are rare and must be clearly detailed and justified in the methods section of the report where they occur.

For peer review only

10.3.1 Title of the Umbrella Review

The title should be clear, explicit and reflect the core elements of the review. Titles should not be phrased as questions or conclusions and there should be congruency between the title, review objectives /questions and inclusion criteria. The title should include the phrase: “An Umbrella Review“. Conventional wisdom suggests that the title should not be more than 12-14 words for ease of understanding. See the informative examples above in Section 10.2.1.

For peer review only

10.3.2 Review Authors

Each reviewer should have fist and last name listed. Affiliations for each author need to be stated, including the JBI affiliation of each reviewer. A valid email address must be provided for the corresponding author.

For peer review only

10.3.3 Abstract

This section forms a structured abstract of the main features of the systematic review. It must be no longer than 500 words and should contain no abbreviations or references. The abstract must accurately reflect and summarize the systematic review with the main focus on the results of the review.

The abstract should report the essential elements of the review using the following sub-headings in this order:

- **Objective**: State an overarching review objective structured using the key components of the inclusion criteria (approximately one to two sentences).
- **Introduction**: Briefly describe what the issue is under review and what is already known on the topic (approximately two to three sentences).
- **Inclusion criteria:** Summarize the inclusion criteria as it relates to the type of review being conducted. Present the information in one or two sentences – **NOT** under individual subheadings.
- **Methods:** List the key information sources searched (those that provided the majority of included studies), any limits placed on the scope of the search (e.g. language), and the date range, or the date of the last search. If the recommended JBI approach to critical appraisal, study selection, data extraction and data synthesis was used, simply state it as such (without naming the actual tool). Otherwise, briefly describe any notable deviations to the methodological approach taken (e.g. criteria used to exclude studies on the basis of methodological quality etc.).
- **Results**: The bulk of the abstract should be reserved to convey the main results of the review.
    - As a general rule, report the number and type of included studies and participants, as well as any pertinent study characteristics. Summarize the overall quality of the included studies and notable aspects of risk of bias.
    - Report the results for all main outcomes (not only those that were statistically significant or clinically important). If meta-analyses were conducted report the summary measures (estimated effect) and confidence intervals and ensure statistics are presented in a standard way. If a meta-analysis was proposed but not conducted, report the reason (e.g. clinical or methodological heterogeneity). Where possible, indicate the number of studies and participants for each main outcome. Describe the direction of the effect (e.g. lower, fewer, greater, more, etc.) in a way that is understandable to patients and health care professionals (i.e. which group was favored and the size of the effect) and indicate the measurement scale used, where applicable.
- **Conclusions**: Articulate brief overall conclusions based on the Umbrella Review findings should be articulated, including a clear answer to the question(s)/objective(s) of the Umbrella Review. Briefly convey key implications for practice and/or research.

For peer review only

10.3.4 Introduction

The introduction should be comprehensive and cover all of the main elements of the topic under review, as well as appropriate information about pathophysiology, diagnosis, prognosis, prevalence or incidence or other detail important to the review and why the topic or question of interest lends itself to an Umbrella Review for example, addressing a range of interventions relevant to a particular diagnosis. The primary objective of the Umbrella Review should be evident in the introduction as it situates the justification and importance of the question(s) posed. While many of these details will already have been addressed in "Introduction" of the protocol, many reviewers will find that the background information provided with the protocol needs modification or extension following the conduct of the review proper. The introduction should conclude with a statement that a preliminary search for previous Umbrella Reviews on the topic was conducted (state the sources searched e.g. *JBI Evidence Synthesis*, The Cochrane Library, Campbell Collection etc).

The introduction should conclude with an overarching review objective that captures and aligns with the core elements/mnemonic of the inclusion criteria (e.g. PICO). The stated objective should clearly indicate what the review project is trying to achieve. The objective(s) may be broad and will be aligned to specific review question(s). The objectives or aims of an example review may be: To examine non-pharmacological interventions for the management of aggressive behaviors in elderly patients with dementia. The Vancouver style referencing should be used throughout the review with superscript numbers without brackets used for in-text citations.

For peer review only

10.3.5 Review question(s)

The primary questions of the review should be stated. It can be followed by specific sub-questions that relate to differing comparisons contained in the Umbrella Review, such as, participant groups, interventions or outcome measures or a more in depth understanding of a particular phenomenon of interest. See example above in Section 10.2.4.

For peer review only

10.3.6 Inclusion criteria

This section of the review details the basis on which systematic reviews and/or meta-analyses were considered for inclusion in the Umbrella Review and should be as transparent and unambiguous as possible. The inclusion criteria for an Umbrella Review will depend on the question(s) asked. As a guiding principle, they should follow the norm for any JBI systematic review, where a question of effectiveness of an intervention(s) or therapy, for example, will stipulate a PICO (Population, Intervention, Comparator, Outcome), or an Umbrella Review that addresses a question that would lend itself to inclusion of qualitative systematic reviews that include a PICo (Population, Phenomena of interest and Context). Umbrella reviews that address multiple questions and evidence types may stipulate both PICO and PICo elements.

Types of participants

The types of participants should be related to the review objectives. The reasons for the inclusion or exclusion of participants detailed in this section should be explained to the reader of the Umbrella Review in the background section of the report.

Interventions/phenomena of interest

There should be congruence between the review objective and the outcomes of interventions under review and/or the phenomena of interest. Interventions may be focused, for example, to only pharmacological management or may be broad, including both pharmacology and other interventions (e. g. diet, exercise, surgery). Relationships should be clearly detailed in the background section. It is beneficial to use definitions where appropriate for the purposes of clarity.

Context/setting

In an Umbrella Review, the context or setting will vary depending on the objective of the review. Context may include but is not limited to consideration of cultural factors such as geographic location, racial or gender based interests. The setting details important features of the study location, such as acute care, primary health care or the community.

Outcomes

Outcomes for Umbrella Reviews should be described and defined and relevant to the question posed by the review. If outcomes are measured in a particular way, this should be included in the description (e.g., measurement of quality of life using the SF-36 questionnaire).

Types of studies

While it is clear that an Umbrella Review will include only existing research syntheses and systematic reviews, there should be a match in this section between the methodology of the systematic review to be considered for inclusion in the Umbrella Review and its primary objective. For example, an Umbrella Review that aims to assess the effectiveness of a range of interventions for aggressive behaviors in elderly dementia patients may limit itself to including systematic reviews that assessed effectiveness by including only randomized controlled trials and other experimental study designs.

For peer review only

10.3.7 Methods

This section of the review report is reserved for the methods used to conduct the review and should be presented under the relevant subheadings (See Sections 10.3.7.1 - 10.3.7.5), including any deviations from the method outlined in the *a priori* protocol. In empty reviews for example, this section should not refer to methods that were not performed.

Directly below the Methods heading provide the following information:

- State and appropriately cite the JBI methodology that was employed in the conduct of the review and synthesis.
- Refer to and cite the *a priori* protocol that was published, or accepted for publication (e.g. 'in press'), in the *JBI Evidence Synthesis*.
- If the protocol has been registered with PROSPERO, provide registration information including registration number (e.g. PROSPERO CRD42015425226).

For peer review only

10.3.7.1 Search strategy

This section should document how the reviewers searched for relevant papers to include in the Umbrella Review. The search strategy needs to be comprehensively reported and as a minimum, a detailed search strategy for all major bibliographic citation databases and other sources that were searched should be appended to the review. Ideally the search strategies for all of the databases searched should be presented sequentially in the single appendix. Clear documentation of the search strategy(ies) is a key element of the scientific validity of an Umbrella Review. A JBI Umbrella Review should consider papers published both commercially and in non-commercially in the gray literature. The timeframe chosen for the search should be justified and any language restrictions stated (e.g. only studies published in English were considered for inclusion). The databases that were searched must be listed along with the search dates. Any hand searching of relevant journals should be described by journal name and years searched. Author contact, if appropriate, should also be included with the results of that contact.

For peer review only

10.3.7.2 Study screening and selection

The review report should describe the actual process of study screening and for all stages of selection (based on title and abstract examination; based on full text examination) and the actual procedures used for solving disagreements between reviewers.

For peer review only

10.3.7.3 Assessment of methodological quality/critical appraisal

This section should detail the approach to critical appraisal, not the assessment results, and should be consistent with the details in the published JBI Umbrella Review protocol. Any deviations from the protocol must be reported and explained in this section of the review report. The JBI critical appraisal instrument for Systematic Reviews and Research Syntheses embedded in the JBI SUMARI software (See Appendices 10.1 and 10.2) must be used and either cited or appended to the review report.

For peer review only

10.3.7.4 Data collection

Standardized data extraction tools maximize the consistent extraction of accurate data across the included studies and are required for JBI Umbrella Reviews. The review should detail what data the reviewers extracted from the included systematic reviews and the JBI data extraction tool for Systematic Reviews and Research Syntheses must be appended to the review report (see Appendix 10.3). As mentioned , individual study level data should not be reported in an Umbrella Review (except where an outcome is only informed by one included study); the focus of reporting should be the results and findings of the included syntheses. Using the JBI extraction tool, at a minimum, details and data relevant to the items listed below should be extracted where the information is available. The majority of this information will appear in the Table of Included Study Characteristics to be appended to the review report, while other important details extracted, particularly relevant to the findings of the review (see below) will appear in the body of the review report:

Author/year

The citation details of included studies should be consistently referred to throughout the document. The citation details should include the name of the first author (Vancouver reference) and year of publication.

Objective(s)

A clear description of the objective of the included research synthesis should be stated.

Participants (characteristics/total number)

The defining characteristics of the participants in studies included in the research syntheses should be detailed, for example this may include diagnostic criteria, or age or ethnicity. The total number of participants that inform the outcomes relevant to the Umbrella Review question from all studies included studies should be presented also.

Setting/context

Details of the setting of interest such as acute care, primary health care, or the community or a particular geographical location should be included. For some Umbrella Reviews, particularly those that draw upon qualitative research syntheses, the context that underpins the review question will be important to clearly reveal to the reader and may include but is not limited to consideration of cultural factors such as geographic location and specific racial or gender based interests.

Interventions/phenomena of interest

Clear, succinct details of the interventions or phenomena of interest should be described, including the type of intervention, the frequency and/or intensity of the intervention for example. A statement of the phenomena of interest is also required where applicable.

Number of databases/sources searched

The number of sources searched should be reported. Though this will have been considered during critical appraisal of the research synthesis, reporting to the reader of the review will allow rapid and easy comparison between differences of included reviews and also consideration of potential for publication bias in the event no formal analysis has been conducted. Where possible the names of databases and sources should be listed (i.e. if <5-10). The search range of each database should also be included.

Date range of included studies

The date range spanning from the earliest study that informs the included research synthesis to the latest should be reported. This is important information that allows for consideration of the currency of the evidence base not necessarily reflected in the year of publication of the research synthesis. If this is not readily identifiable in the table of study characteristics provided by the included synthesis, it should be discer nable by scanning the date range of publications through the results section of the included review.

Number of Studies/Type of Studies/Country of origin of included studies

Summary descriptive details of the included studies in the research synthesis should be reported. This includes the number of studies in the included research synthesis, the types of study designs included in the research synthesis, for example randomized controlled trials, prospective cohort study, phenomenology, ethnography etc., and also the country of origin of the included studies. The latter is important to allow the reader of the review to consider the external validity and generalizability of the results presented.

Appraisal instrument and rating

The instrument or tool used to assess risk of bias, rigor or study quality should be reported along with some summary estimate of the quality of primary studies in the included research synthesis. For example, for Umbrella Reviews that use the Jadad Scale, a mean score for quality may be reported whereas for checklist appraisals, reporting of cut-off score or any ranking of quality should be reported. An example of the latter would be exclusion of studies that score <3/10, and inclusion of four moderate quality studies (4-6/10) and two high quality studies (7-10/10).

For peer review only

Type of Review/Method of analysis

The type of research synthesis as stated by the authors of the included review should be detailed. The method of analysis or synthesis used by the included research synthesis should be reported. For example, this may include random effects meta-analysis, fixed effect meta-analysis, meta- aggregative synthesis or meta-ethnography.

Outcome(s)

Included here should be the outcomes of interest to the Umbrella Review question reported on by the research synthesis, i.e. the names or labels of the outcomes (see below for presentation of results).

Results/findings

The relevant findings or results presented by the included research syntheses must be extracted. For quantitative reviews, this will ideally be an effect estimate or measure from a presented meta- analysis. Measures of heterogeneity should also be extracted where applicable. In the absence of this a statement indicating the key result relevant to an outcome may be inserted in the required field. For qualitative syntheses, the key synthesized finding should be extracted.

Comments

There should be provision to extract and present in the table of included study characteristics any relevant details or comments on the included research synthesis by the authors of the Umbrella Review. These comments may be relevant details regarding the included research synthesis, for example, the congruence between the review results and conclusions, and for highlighting any potential methodological differences between the individual included reviews.

For peer review only

10.3.7.5 Data Summary

This section should detail the approach to the presentation of findings and the results from included research syntheses, not the results of this process. The types of data detailed in this section should be consistent with the methods used for data collection and the included study designs.

For peer review only

10.3.8 Results

This section of the review report has distinct sub-sections describing the process of study inclusion, the methodological quality of the eligible studies, detailed characteristics and description of the included studies and, importantly, the findings of the review and results of the synthesis processes.

For peer review only

10.3.8.1 Study inclusion

This section should provide a narrative summary of the search results and selection process and results. The number of papers identified by the search strategy and the number of papers that were included and excluded should be stated.

A complete and accurate report should be provided regarding:

- the number of studies identified by the search in diverse sources;
- the number of studies excluded after the examination of title and abstract against inclusion criteria;
- the number of full text articles retrieved for examination;
- the number of studies excluded after full text examination against inclusion criteria;
- the number of critically appraised studies;
- the number of studies excluded after critical appraisal;
- the final total number of included studies.

A flowchart using the PRISMA template for the reporting of the selection process should be included (Moher et al, 2009).

Ideally, a list of all excluded studies, excluded after full text examination and after critical appraisal, with the explicit reasons for exclusion, should be provided in appendices to the review. As a minimum, at least the list of studies excluded after critical appraisal and the reasons for exclusion should be reported. If no studies were excluded after critical appraisal then the list of all studies excluded after full text examination including the explicit reasons for exclusion, should be provided in appendices to the review.

For peer review only

10.3.8.2 Methodological quality

This section should focus on methodological quality as determined by the JBI critical appraisal checklist for Systematic Reviews and Research Syntheses (see Appendices 10.1 and 10.2). There should be a narrative summary of the overall methodological quality of the included studies, which can be supported (optional) by a table showing the overall results of the critical appraisal (see Table 10.1 for example). Where only few studies are identified, or there are specific items of interest from included studies, these should be addressed in the narrative also, particularly where studies were deficient, or particularly good. i. e. with clear narrative regarding risk of bias/rigor of included studies. Use of N/A should also be justified in the text. Importantly, in a JBI Umbrella Review, it is important to present to the reader with clear indication of the quality of the included original research studies in each of the systematic reviews or research syntheses that are included in the Umbrella Review. This will have an impact on the interpretation and implications for practice and research and must be noted with clarity to the reader of the review in the body of the report. This detail will appear in the appended Table of Included Study Characteristics (see above)

Table 10.1: Critical appraisal results for included studies

Study	Q1	Q2	Q3	Q4	Q5	Q6	Q7	Q8	Q9	Q10
Author(s) ref	Y	Y	Y	N	Y	U	Y	N	Y	U

Y - Yes, N - No, U - Unclear

For peer review only

10.3.8.3 Characteristics of included studies

This section of the results should also include an overall description of the included studies (with reference to the detailed table of included study characteristics in the appendices). The main aim is to provide context to the results section and sufficient descriptive detail for the reader to support the inclusion of the systematic reviews in the Umbrella Review, the relevance of included systematic review to the Umbrella Review question and the evidence base they offer to the question. Specific items/points of interest/outcomes from individual reviews may also be highlighted here. A summary table of included studies should be appended to the report that will be populated from the appropriate extraction fields form the extraction tool (See Appendix 10.3).

For peer review only

10.3.8.4 Findings of the review

The findings of the review and presentation of the results should flow logically from the review objection /question i.e. they must ultimately answer the questions posed. The findings and key results extracted from the included research syntheses should constitute part of this section and may include presentation of quantitative and qualitative data. Both quantitative and qualitative findings presented in the JBI Umbrella Review report should be presented in a tabular format with supporting text.

Quantitative tabulation of results presented in this section must include clear presentation of the name of the intervention, the study or citation details that inform the intervention, the number of studies and individual participants that inform the outcome measure, the calculated effect estimate where possible or the main finding of the study related to the intervention and relevant outcome, as well as any details of measures of heterogeneity about the effect estimate(s). An example of the table of findings is below in Table 10.2 for one outcome. In this example it is for 'aggressive behaviors', if other outcomes were included, the final three columns of the table would be repeated for each. Tabular presentation must be accompanied by a clear and detailed description of the interventions addressed.

Table 10.2: Tabular presentation of quantitative findings for an Umbrella Review

Interventions/ phenomena of interest	Author/year	Number of studies/Participants	Results/Findings	Heterogeneity
**Staff training programs**	Kynoch, et al, 2009	1	No difference in Patient aggression between staff training and control group.	N/A
**Physical restraint**	Kynoch, et al, 2009	1	Not calculated	
**Music therapy**	Kynoch, et al, 2009	2	Not calculated	
**Multiple interventions**	Kynoch, et al, 2009	2	Not calculated	
**Bright light**	Forbes	5 /343 participants	Agitation at 1 year follow-up<br>-2.00 (-11.71,7.71)	N/A

Qualitative findings should also be tabulated in this section of the Umbrella Review report. A description of the phenomenon of interest alongside the key synthesized findings extracted from each included qualitative meta- synthesis or systematic review should be presented. Individual findings and illustrations that would be the norm for presentation in a JBI meta-aggregative review would not be presented in a JBI Umbrella Review presenting qualitative data. To facilitate interpretability and clarity of the findings in this section of the review, adequate contextual and descriptive detail should also be presented.

An example of tabular presentation of qualitative findings in a JBI Umbrella Review is presented in Table 10.3. In this table the synthesized finding presented must be an accurate, verbatim replication of the finding from the source review. The descriptive information in the final column may constitute the Umbrella Review authors' own words to provide the necessary detail for interpretability. Depending on the review, it is likely that an individual table would be presented for each included qualitative synthesis; otherwise, further rows could be added to the example table. This tabular presentation must be accompanied by further descriptive detail of the phenomena of interest to the review in the text.

Table 10.3: Tabular presentation of qualitative findings for an Umbrella Review

Phenomena of interest/context	Synthesized finding	Details of strategies
**Nurse's perspective of therapeutic interventions to manage verbal and behavioural aggression among patients with dementia or brain injury**	Entering the patient's world:<br>Nurses are encouraged to become a part of the patient's world and to strive for mutual understanding and action. Patient negativity is downplayed and interactions are shaped in thoughtfully creative ways	**Normalization:** Getting to know the patient behind the aggression and normalizing their experiences. Aggression is seen as part of the condition
		**Person-centered care:** During care the person's needs, experiences, and feelings remain a focal point instead of procedures
		**Nurse-patient mutuality:** Nurses are encouraged to mutually work through problems rather than always doing things to and for patients
		**Downplaying negativity:** Nurses are urged to develop a positive interaction style with potentially aggressive patients and to disregard or overlook negative behavior
		**Thoughtful creativity:** Nurses are urged to be reflective, use their imaginations, and be flexible when providing care
	Utilitarian care:<br>Nurses are inflexibly committed to rules and routines; focused on duty, responsibility, being in charge, and streamlining care	**Inflexible routines**: When inflexible routines are maintained, patients tend to be seen as troublesome,<br>and their behaviors are viewed as meaningless

For peer review only - http://bmjopen.bmj.com/site/about/guidelines.xhtml

10.3.9 Summary of Evidence

In line with the objectives of a JBI Umbrella Review to present an accurate and informative overview of the findings of research syntheses that inform a broad topic or question, all JBI Umbrella Reviews should conclude the results section of the report with a final and easily interpretable table that presents the overall "Summary of Evidence".

For quantitative findings, a final table should be presented that names the intervention, identifies the included research synthesis and provides a simple, visual indication of the results. Visual indication should follow a simple "stop-light" indicator, where green indicates the intervention is beneficial (effective), amber that there is no difference in the investigated comparison, and red that the results suggest the intervention is detrimental or less effective than the comparator. Actual details and effect estimates are presented in the findings of the review (see above). Table 10.4 presents an example for "aggressive behavior". Further outcomes reported in an Umbrella Review could be added in columns to the right. Where a study does not report on an outcome, the indicator square should be left blank.

Table 10.4: Summary of Evidence from quantitative research syntheses in a JBI Umbrella Review

Interventions/ phenomena of interest	Author/year	Aggressive behavior
Bright light	Forbes 2009	
Staff training programs	Kynoch, et al, 2009	
Physical restraint	Kynoch, et al, 2009	
Music therapy	Kynoch, et al, 2009	
Multiple interventions	Kynoch, et al, 2009	

Similarly, Umbrella Reviews that include qualitative syntheses should also conclude the results section with a clear summary of the overall findings of the included research syntheses.

In the final summary table, the key synthesized findings should be presented for the reader; for other contextual details the main findings can be referred to (see above). Similar to a summary presentation of qualitative findings, visual indicators of the finding should be included where possible. In the example provided in Table 10.5, those perspectives (see phenomenon) that are beneficial or facilitative are highlighted in green, while those that are inhibitory are highlighted in red.

Table 10.5: Summary of Evidence from qualitative research syntheses in a JBI Umbrella Review

Phenomena of interest	Authors	Synthesized finding
Nurse's perspective of optimal therapeutic interventions	Finfgeld-Connett et al.	Entering the patient's world - becoming part of the patient's world characterised by:<br>• Normalization<br>• Person-centered care<br>• Nurse-patient mutuality<br>• Downplaying negativity<br>• Thoughtful creativity
		Utilitarian care – characterized by:<br>• Being inflexibly committed to rules and routines<br>• Focused on duty, responsibility, being in charge and streamlining care

For peer review only - http://bmjopen.bmj.com/site/about/guidelines.xhtml

10.3.10 Discussion

This section should discuss the results of the review as well as any limitations of the systematic reviews or research syntheses included in the Umbrella Review and of the review itself (i.e. language, access, timeframe, study design, etc.). The results should be discussed in the context of current literature, practice and policy. Umbrella Reviews are subject to many of the limitations of any systematic review including that potentially relevant studies have been omitted and that some systematic error occurred during the selection, appraisal or data extraction processes. Similarly, Umbrella Reviews are ultimately dependent on the reporting of the included research syntheses which may limit reporting of desirable details of interventions for example in the Umbrella Review report. Inherent bias exists in the reporting of an Umbrella Review as one round of appraisal and extraction, where errors may arise, has already been performed in the conduct of the included systematic review or meta-analysis. Umbrella Reviews will also always be limited by the coverage of existing systematic reviews or research syntheses. For example, if an existing intervention or phenomena of interest is yet to be addressed in a systematic review, an Umbrella Review will never identify it.

For peer review only

10.3.11 Conclusions and recommendations

This section should include the overall conclusions of the review. The conclusions should provide direct answers to the review objectives/questions. These conclusions should be based only on the results of the review and directly inferred from the review results.

Recommendations for practice

This sub-section of Conclusions section should include the recommendations for practice inferred from the results of the review and inferred also based on the discussion of the generalizability of the results and the potential factors that may affect the applicability of results. It should be stated how the findings of the review impact on clinical practice or policy in the area. Where there is sufficient evidence to make specific recommendations for practice, these should be clearly articulated Recommendations should be assigned a JBI Grade of Recommendation.

Recommendations for research

This sub-section should include clear, specific recommendations for future research based on gaps in knowledge identified from the results of the review. Umbrella Review authors may find they are able to make comment both on the future conduct of research syntheses and systematic reviews as well as to provide comment on the primary research conducted in the area of interest.

For peer review only

10.3.12 Conflicts and acknowledgements

Details of requirements in these sections are described in Section 1.6. of this Manual.

Conflicts of interest

A statement which either declares the absence of any conflicts of interest or which describes a specified or potential conflict of interest should be made by the reviewers in this section.

Funding

Authors should provide details regarding any sources of funding for the review project. The role of all funders in the review process, if any, should be explicitly described. If the review is funded, then any potential conflicts of interest or intellectual bias of the funders should be specified in the review.

Acknowledgements

Any acknowledgements should be made in this section e.g. sources of external funding or the contribution of colleagues or institutions. It should also be noted if the systematic review is to count towards a degree award.

For peer review only

10.3.13 Review Appendices

Appendix 1: Search strategy

- A detailed and complete search strategy for all of the major databases and other sites and sources searched must be appended. Major databases that were searched must be identified, including the search platform used where necessary. All search filters with logic employed should be displayed, including the number of records returned.

Appendix 2: Data extraction instrument

- The data extraction instrument used must be appended i.e JBI Data Extraction Form.

Appendix 3: List of excluded studies

- Studies excluded following examination of the full-text should be listed along with their reason for exclusion at that stage (i.e. a mismatch with the inclusion criteria). This may be as a separate appendix or itemized in some fashion within the one appendix with those studies excluded at the critical appraisal stage. Reasons for exclusion following appraisal should be provided for each study (these reasons should relate to the methodological quality of the study, not study eligibility).

Appendix 4: Table of included study characteristics

- A table of included studies is required to provide quick reference to important details extracted from of the studies included in the review.

For peer review only

10.4 Chapter references

Aromataris, E., Fernandez, R., Godfrey, C., Holly, C., Kahlil, H. & Tungpunkom, P. 2015 Summarizing systematic reviews: methodological development, conduct and reporting of an Umbrella review approach International Journal of Evidence based Healthcare, 13(3):132-140.

Bastian H, Glasziou P, Chalmers I. Seventy-five trials and eleven systematic reviews a day: how will we ever keep up? PLoS Med 2010; 7(9):e1000326.

Becker LA, Oxman AD. Chapter 22: Overviews of reviews. In: Higgins JPT, Green S (editors), Cochrane Handbook for Systematic Reviews of Interventions Version 5.1.0 (updated March 2011). The Cochrane Collaboration, 2011. Available from www.cochrane-handbook.org.

Grant M, Booth A. A typology of reviews: an analysis of 14 review types and associated methodologies. Health Information and Libraries Journal; 2009 26:91-108 10.1111/j.1471-1842.2009.00848.x

Guyatt GH, Oxman AD, Vist G, Kunz R, Falck-Ytter Y, Alonso-Coello P, Schünemann HJ, for the GRADE Working Group. Rating quality of evidence and strength of recommendations GRADE: an emerging consensus on rating quality of evidence and strength of recommendations. BMJ; 2008; 336:924-926.

Hartling L, Chisholm A, Thomson D, Dryden DM. A descriptive analysis of overviews of reviews published between 2000 and 2011. PLoS One 2012; 7(11)e49667.

Khangura S, Konnyu K, Cushman R, Grimshaw J, Moher M. Evidence summaries: the evolution of a rapid review approach. Systematic Reviews 2012; 1:10.

Moher D, Liberati A, Tetzlaff J, Altman DG, The PRISMA Group (2009). Preferred Reporting Items for Systematic Reviews andMeta-Analyses: The PRISMA Statement. PloS Med 6(6):e1000097. Doi:10.1371 /journal.pmed1000097

Smith V, Devane D, Begley C, Clarke M Methodology in conducting a systematic review of systematic reviews of healthcare interventions. BMC Medical Research Methodology; 211; 11:15. doi:10.1186/1471-2288-11-15

For peer review only

Appendix 10.1 JBI Critical Appraisal Checklist for Systematic reviews and Research Syntheses

For peer review only

JBI Critical Appraisal Checklist for Systematic Reviews and Research Syntheses

Reviewer ______________________________ Date ________________

Author ______________________________ Year ________ Record Number

		Yes	No	Unclear	Not applicable
1.	Is the review question clearly and explicitly stated?	☐	☐	☐	☐
2.	Were the inclusion criteria appropriate for the review question?	☐	☐	☐	☐
3.	Was the search strategy appropriate?	☐	☐	☐	☐
4.	Were the sources and resources used to search for studies adequate?	☐	☐	☐	☐
5.	Were the criteria for appraising studies appropriate?	☐	☐	☐	☐
6.	Was critical appraisal conducted by two or more reviewers independently?	☐	☐	☐	☐
7.	Were there methods to minimize errors in data extraction?	☐	☐	☐	☐
8.	Were the methods used to combine studies appropriate?	☐	☐	☐	☐
9.	Was the likelihood of publication bias assessed?	☐	☐	☐	☐
10.	Were recommendations for policy and/or practice supported by the reported data?	☐	☐	☐	☐
11.	Were the specific directives for new research appropriate?	☐	☐	☐	☐

Overall appraisal: Include ☐ Exclude ☐ Seek further info ☐

For peer review only

Appendix 10.2. Discussion of JBI Critical Appraisal Checklist for systematic reviews and research syntheses

This appraisal instrument can be found in the JBI SUMARI software.

Review authors should discuss each item in the appraisal instrument for each study included in their review. In particular, discussions should focus on what is considered acceptable to the aims of the review in terms of the specific study characteristics. When appraising systematic reviews this discussion may include issues such as what represents an adequate search strategy or appropriate methods of synthesis. The reviewers should be clear on what constitutes acceptable levels of information to allocate a positive appraisal compared with a negative, or response of "unclear". This discussion should ideally take place before the reviewers independently conduct the appraisal.

Within Umbrella reviews, quantitative or qualitative systematic reviews may be incorporated, as well as meta-analyses of existing research. The individual checklist is available in Appendix 10.1.

There are 11 questions to guide the appraisal of systematic reviews or meta-analyses. Each question should be answered as "yes", "no", or "unclear". Not applicable "NA" is also provided as an option and may be appropriate in rare instances.

1. Is the review question clearly and explicitly stated?

The review question is an essential step in the systematic review process. A well-articulated question defines the scope of the review and aids in the development of the search strategy to locate the relevant evidence. An explicitly stated question, formulated around its PICO (Population, Intervention, Comparator, Outcome) elements aids both the review team in the conduct of the review and the reader in determining if they review has achieved its objectives. Ideally the review question should be articulated in a published protocol; however this will not always be the case with many reviews that are located.

2. Were the inclusion criteria appropriate for the review question?

The inclusion criteria should be identifiable from and match the review question. The necessary elements of the PICO should be explicit and clearly defined. The inclusion criteria should be detailed and the included reviews should clearly be eligible when matched against the stated inclusion criteria. Appraisers of meta-analyses will find that inclusion criteria may encompass criteria around the ability to conduct statistical analyses which would not be the norm for a systematic review. The types of included studies should be relevant to the review question, for example, an Umbrella Review aiming to summarize a range of effective non-pharmacological interventions for aggressive behaviors amongst elderly patients with dementia will limit itself to including systematic reviews and meta-analyses that synthesize quantitative studies assessing the various interventions; qualitative or economic reviews would not be included.

3. Was the search strategy appropriate?

A systematic review should provide evidence of the search strategy that has been used to locate the evidence. This may be found in the methods section of the review report in some cases, or as an appendix that may be provided as supplementary information to the review publication. A systematic review should present a clear search strategy that addresses each of the identifiable PICO components of the review question. Some reviews may also provide a description of the approach to searching and how the terms that were ultimately used were derived, though due to limits on word counts in journals this may be more the norm in online only publications. There should be evidence of logical and relevant keywords and terms and also evidence that Subject Headings and Indexing terms have been used in the conduct of the search. Limits on the search and their potential impact should also be considered; for example, if a date limit was used, was this appropriate and/or justified? If only English language studies were included, will the language bias have an impact on the review? The response to these considerations will depend, in part, on the review question.

4. Were the sources and resources used to search for studies adequate?

A systematic review should attempt to identify "all" the available evidence and as such there should be evidence of a comprehensive search strategy. Multiple electronic databases should be searched including major bibliographic citation databases such as MEDLINE and CINAHL. Ideally, other databases that are relevant to the review question should also be searched, for example, a systematic review with a question about a physical therapy intervention should also look to search the PEDro database, whilst a review focusing on an educational intervention should also search the ERIC. Reviews of effectiveness should aim to search trial registries. A comprehensive search is the ideal way to minimize publication bias. As a result, a well conducted systematic review should also attempt to search for gray literature, or "unpublished" studies; this may involve searching websites relevant to the review question or thesis repositories.

5. Were the criteria for appraising studies appropriate?

For peer review only

The systematic review should present a clear statement that critical appraisal was conducted and provide details of the items that were used to assess the included studies. This may be presented in “Methods of the review”, as an appendix of supplementary information, or as a reference to a source that can be located. The tools or instruments used should be appropriate for the review question asked and the type of research conducted. For example, a systematic review of effectiveness should present a tool or instrument that addresses aspects of validity for experimental studies and randomized controlled trials such as randomization and blinding – if the review includes observational research to answer the same question, a different tool would be more appropriate. Similarly, a review assessing diagnostic test accuracy may refer to the recognized QUADAS tool (Whiting et al, 2003).

6. Was critical appraisal conducted by two or more reviewers independently?

Critical appraisal or some similar assessment of the quality of the literature included in a systematic review is essential. A key characteristic to minimize bias or systematic error in the conduct of a systematic review is to have the critical appraisal of the included studies completed by members of the review team independently and in duplicate. The systematic review should present a clear statement that critical appraisal was conducted by at least two reviewers working independently from each other and conferring where necessary to reach a decision regarding study quality and eligibility on the basis of quality.

7. Were there methods to minimize errors in data extraction?

Efforts made by review authors during data extraction can also minimize bias or systematic errors in the conduct of a systematic review. Strategies to minimize bias may include conducting all data extraction in duplicate and independently, using specific tools or instruments to guide data extraction and some evidence of piloting or training around their use.

8. Were the methods used to combine studies appropriate?

A synthesis of the evidence is a key feature of a systematic review. The synthesis that is presented should be appropriate for the review question and the stated type of systematic review and evidence it refers to. If a meta-analysis has been conducted this needs to be reviewed carefully. Was it appropriate to combine the studies? Have the reviewers assessed heterogeneity statistically and provided some explanation for heterogeneity that may be present? Often, where heterogeneous studies are included in the systematic review, narrative synthesis will be an appropriate method for presenting the results of multiple studies. If a qualitative review, are the methods that have been used to synthesize findings congruent with the stated methodology of the review? Is there adequate descriptive and explanatory information to support the final synthesized findings that have been constructed from the findings sourced from the original research?

9. Was the likelihood of publication bias assessed?

As mentioned, a comprehensive search strategy is the best means by which a review author may alleviate the impact of publication bias on the results of the review. Reviews may also present statistical tests such as Egger’s test or funnel plots to also assess the potential presence of publication bias and its potential impact on the results of the review. This question should be considered N/A for JBI qualitative reviews.

10. Were recommendations for policy and/or practice supported by the reported data?

Whilst the first nine questions specifically look to identify potential bias in the conduct of a systematic review, the final questions are more indicators of review quality rather than validity. Ideally a review should present recommendations for policy and practice. Where these recommendations are made there should be a clear link to the results of the review. Is there evidence that the strength of the findings and the quality of the research have been considered in the formulation of review recommendations?

11. Were the specific directives for new research appropriate?

The systematic review process is recognized for its ability to identify gaps in the research, or knowledge base, around a particular topic. Most systematic review authors will provide some indication, often in the discussion section of the report, of future research direction. Where evidence is scarce or sample sizes that support overall estimates of effect are small and effect estimates are imprecise, repeating similar research to those identified by the review may be necessary and appropriate. In other instances, the case for new research questions to investigate the topic may be warranted.

P Whiting, AWS Rutjes, JB Reitsma, PMM Bossuyt, J Kleijnen. The development of QUADAS: a tool for the quality assessment of studies of diagnostic accuracy included in systematic reviews BMC Medical Research Methodology 2003, 3:2

For peer review only

Appendix 10.3 JBI Data Extraction Form for Review for Systematic Reviews and Research Syntheses

Study Details	
Author/year	
objectives	
Participants (characteristics/ total number)	
Setting/context	
Description of Interventions/ phenomena of interest	
Search Details	
Sources searched	
Range (years) of included studies	
Number of studies included /	
Types of studies included	
Country of origin of included studies	
Appraisal	
Appraisal instruments used	
Appraisal rating	
Analysis	
Method of analysis	
Outcome assessed	
Results/Findings	
Significance/direction	
Heterogeneity	
Comments	

Chapter 11: Scoping reviews

Micah DJ Peters, Christina Godfrey, Patricia McInerney, Zachary Munn, Andrea C. Tricco, Hanan Khalil

How to cite:

Peters MDJ, Godfrey C, McInerney P, Munn Z, Tricco AC, Khalil, H. Chapter 11: Scoping Reviews (2020 version). In: Aromataris E, Munn Z (Editors). *JBI Manual for Evidence Synthesis*, JBI, 2020. Available from https://synthesismanual.jbi.global. https://doi.org/10.46658/JBIMES-20-12

Updated from:

Peters MDJ, Godfrey C, McInerney P, Baldini Soares C, Khalil H, Parker D. Chapter 11: Scoping Reviews. In: Aromataris E, Munn Z (Editors). *Joanna Briggs Institute Reviewer's Manual,* JBI, 2017

For peer review only

Chapter 11: Contents

- 11.1 Introduction to Scoping reviews
  - 11.1.1 Why a scoping review?
  - 11.1.2 Scoping reviews compared to other types of review
  - 11.1.3 The scoping review framework
- 11.2 Development of a scoping review protocol
  - 11.2.1 Title
  - 11.2.2 Developing the title and question
  - 11.2.3 Introduction
  - 11.2.4 Inclusion criteria
  - 11.2.5 Search Strategy
  - 11.2.6 Source of evidence selection
  - 11.2.7 Data extraction
  - 11.2.8 Analysis of the evidence
  - 11.2.9 Presentation of the results
- 11.3 The scoping review and summary of the evidence
  - 11.3.1 Title of the scoping review
  - 11.3.2 Review authors
  - 11.3.3 Abstract
  - 11.3.4 Introduction
  - 11.3.5 Review question(s)
  - 11.3.6 Inclusion Criteria
  - 11.3.7 Methods
    - 11.3.7.1 Search strategy
    - 11.3.7.2 Source of evidence screening and selection
    - 11.3.7.3 Data extraction
    - 11.3.7.4 Analysis and Presentation of results
  - 11.3.8 Results
    - 11.3.8.1 Search results
    - 11.3.8.2 Inclusion of sources of evidence
    - 11.3.8.3 Review findings
  - 11.3.9 Discussion
  - 11.3.10 Conclusions and recommendations
  - 11.3.11 Conflicts and acknowledgements
  - 11.3.12 References
  - 11.3.13 Review appendices
- 11.4 Chapter references
- Appendix 11.1 JBI template source of evidence details, characteristics and results extraction instrument
- Appendix 11.2 PRISMA ScR Extension Fillable Checklist

For peer review only - http://bmjopen.bmj.com/site/about/guidelines.xhtml

11.1 Introduction to Scoping reviews

Evidence-based healthcare is an expanding field. Together with the continual increase in the availability of primary research, the conduct of reviews has also increased and evolved. Different forms of evidence and different review objectives and questions have led to the development of new approaches that are designed to more effectively and rigorously synthesize the evidence. In 2009, Grant and Booth identified 14 different types of reviews (Grant & Booth 2009), whilst in 2016 Tricco and colleagues identified 25 knowledge synthesis methods (Tricco et al. 2016c). Scoping reviews, which have also been called "mapping reviews" or "scoping studies" are one type of review (Ehrich et al. 2002; Anderson et al. 2008). Arksey and O'Malley proposed an original framework for conducting scoping reviews (Arksey & O'Malley 2005). This framework was then advanced and extended by Levac and colleagues (2010). Scoping review methodology was then further refined, and corresponding guidance developed by a working group from JBI and the JBI Collaboration (JBIC) (Peters et al. 2015, 2017). The guidance from this group explicitly addressed the need for this type of knowledge synthesis to be rigorously conducted, transparent and trustworthy. Peters et al. (2015, 2017) used the label 'systematic scoping review' in their original guidance for conduct and reporting of these types of reviews (Peters et al. 2015, 2017). In this current update, the nomenclature has been refined to simply 'scoping reviews' in acknowledgement that all types of knowledge synthesis should be systematic in their conduct, and that this is the most common term used for these types of reviews (Tricco et al. 2016b). In 2018, the Preferred Reporting Items for Systematic Reviews (PRISMA) Statement was extended to Scoping Reviews – the PRISMA-ScR (Tricco et al. 2018). The PRISMA-ScR was developed by a number of experts in scoping reviews and evidence synthesis, including members of the JBI/JBIC working group, to be consistent with the JBI scoping review methodology (Peters et al. 2017). Following the PRISMA-ScR and meetings of the scoping review methodology group, an updated version of the JBI scoping review methodology is now available.

For peer review only

11.1.1 Why a scoping review?

There are a number of reasons why a scoping review might be conducted. Unlike other reviews that tend to address relatively precise questions (such as a systematic review of the effectiveness of an intervention assessed using a predefined set of outcomes), scoping reviews can be used to map the key concepts that underpin a field of research, as well as to clarify working definitions, and/or the conceptual boundaries of a topic (Arksey & O'Malley 2005). A scoping review may address one of these aims or all of them. A scoping review of scoping reviews found that the three most common reasons for conducting a scoping review were to explore the breadth or extent of the literature, map and summarize the evidence, and inform future research (Tricco et al. 2016b). The indications for scoping reviews are listed below: (Munn et al. 2018a)

- As a precursor to a systematic review.
- To identify the types of available evidence in a given field.
- To identify and analyse knowledge gaps.
- To clarify key concepts/ definitions in the literature.
- To examine how research is conducted on a certain topic or field.
- To identify key characteristics or factors related to a concept.

Scoping reviews undertaken with the objective of providing a 'map' of the available evidence can be undertaken as a preliminary exercise prior to the conduct of a systematic review (Anderson et al. 2008). Scoping reviews are useful for examining emerging evidence when it is still unclear what other, more specific questions can be posed for evidence syntheses and valuably addressed. For example, while there are few studies on the sustainability of knowledge translation interventions in the area of chronic disease management, a scoping review has provided the foundation for a future systematic review to investigate the impact of sustainable knowledge translation interventions on health outcomes (Tricco et al. 2016a).

Authors deciding between the systematic review or scoping review approach should carefully consider the indications discussed above and determine exactly what question they are asking and what purpose they are trying to achieve with their review (Munn et al. 2018a). It is important for authors to clearly articulate *why* they are undertaking a scoping review; i.e. why is it necessary to identify and map the evidence in a given field? What will mapping the evidence achieve in terms of the objective of the review? Perhaps the most important consideration is whether or not the authors wish to use the results of their review as the basis for a trustworthy clinical guideline, to answer a clinically meaningful question, or provide evidence to inform practice or policy (Munn et al. 2018a). If so, then a systematic review approach is best. If the authors have a question addressing the feasibility, appropriateness, meaningfulness or effectiveness of a certain treatment or practice, then a systematic review is likely the most valid approach (Pearson 2004, 2005). A diverse suite of approaches to conducting systematic reviews to answer different types of clinical questions (i.e. effectiveness, prognosis, risk, etc) exist (Munn et al. 2018b). However, authors do not always wish to ask single or precise clinical questions and may be more interested in the identification of certain characteristics/concepts in sources of evidence, and in the mapping, reporting or discussion of these characteristics/concepts. In these cases, a scoping review is the better choice.

Unlike a systematic review, scoping reviews do not tend to produce and report results that have been synthesized from multiple evidence sources following a formal process of methodological appraisal to determine the quality of the evidence. Rather, scoping reviews aim to provide an overview or map of the evidence. Due to this, an assessment of methodological limitations or risk of bias of the evidence included within a scoping review is generally not performed (unless there is a specific requirement due to the nature of the scoping review aim) (Khalil et al. 2016; Peters et al. 2015). Given this assessment of bias is not conducted, the implications/recommendations for practice (from a clinical or policy making point of view) that arise from a scoping review are quite different compared to those of a systematic review. In some cases, there may be no need to articulate implications for practice and if there is a need to do so, these implications may be limited in terms of providing guidance from a clinical or policy making point of view. Conversely, when we compare this to systematic reviews, the provision of implications for practice is a key feature of systematic reviews and is recommended in reporting guidelines for systematic reviews (Liberati et al. 2009). To put it simply, systematic reviews normally inform the development of trustworthy clinical guidelines and recommendations. Scoping reviews are not conducted for this reason but rather to provide an overview of the evidence or to answer questions regarding the nature and diversity of the evidence/knowledge available

Davis and colleagues (2009) explain how, as useful tools for evidence reconnaissance, scoping reviews can be used to provide a broad overview of a topic. For instance, a scoping review that seeks to develop a "concept map" may aim to explore how, by whom and for what purpose a particular term is used in a given field (Anderson et al. 2008). Another example includes where scoping reviews have been performed to establish a comprehensive understanding of how scoping reviews have been conducted and reported (Pham et al 2014; Tricco et al. 2016b). Scoping review methodology was used to identify papers and guidelines that had either utilized or described scoping review methods and/or assessed the quality of reporting for scoping reviews (Tricco et al. 2016b). The review by Tricco et al (2016b) illustrates how the number of scoping reviews has steadily increased since 2012, that there was variation in terms of how they were conducted and reported, and that standardized reporting guidelines were absent.

For peer review only - http://bmjopen.bmj.com/site/about/guidelines.xhtml

Scoping reviews may also be used to develop “policy maps” by identifying and mapping evidence from policy documents and reports that guide practice in a particular field (Anderson et al. 2008). For example, a scoping review might include the objective of mapping research papers and policy documents that concern models of transition for young people to adult health services to provide evidence for best practice transitional care for children with complex health needs (Watson et al. 2011).The value of scoping reviews to evidence-based healthcare and practice lies in the examination of a broader area to identify gaps in the research knowledge base (Crilly et al. 2009, clarify key concepts (de Chavez et al. 2005), and report on the types of evidence that address and inform practice in the field (Decaria et al. 2012).

Due to the range of reasons why a scoping review may be conducted, it is important that reviewers clearly describe the rationale behind their particular scoping review within both the protocol and the review. This gives readers a clearer understanding of the importance of the topic and why a particular type of scoping review is being conducted.

For peer review only

11.1.2 Scoping reviews compared to other types of review

The synthesis of evidence in the form of the systematic review is at the center of evidence-based practice (Pearson et al. 2005).

Systematic reviews traditionally bring together evidence from quantitative literature to answer questions on the effectiveness of a specific intervention for a particular condition. Beyond effectiveness, JBI is also interested in the context of care delivery, its cost-effectiveness, as well as patient, carer and healthcare provider preferences. These foci are explored in terms of the appropriateness, meaningfulness, and feasibility of healthcare practices and delivery. These sorts of questions are most commonly answered by consideration of other forms of primary evidence found in qualitative and economic research studies. The results of well-designed research studies of any methodology are regarded by JBI as potential sources of credible evidence to inform healthcare practice and policy. To match this broader and more inclusive view of evidence, JBI has developed a number of methodologies and methods for the synthesis of evidence to support healthcare decision-making for a number of review types (Munn et al. 2018b).

All JBI knowledge syntheses – including scoping reviews – begin with the development of an *a priori* protocol with inclusion and exclusion criteria that relate clearly to the review question/s. A typical systematic review aims to answer a specific question (or series of questions) based on very precise inclusion criteria, for example, a systematic review may pose the following precise question based upon the PICO (Population, Intervention, Comparator, and Outcome) elements of its inclusion criteria (Marshall-Webb et al. 2018):

> What is the effectiveness of Nissen fundoplication in comparison to anterior partial fundoplication (90 degree, 120 degree and 180 degree) and posterior 270 degree fundoplication in terms of symptom control of gastro-esophageal reflux disease, and what are the side effects of these surgical interventions?

It is clear from this question that only certain types of experimental evidence and data would be relevant and that the review will be very specific in terms of the population, intervention, comparator and outcomes against which it will determine effectiveness.

A scoping review will have a broader "scope" with correspondingly less restrictive inclusion criteria. The following question based upon the PCC (Population, Concept and Context; see Section 11.2) elements of the inclusion criteria may be posed (Kao et al. 2017a):

> "What quality of life questionnaires are available for pediatric patients following tonsillectomies with or without adenoidectomies for chronic infections or sleep disordered breathing?"

The 'population' in this question is clearly specified (pediatric patients who have had tonsillectomies with or without adenoidectomies). The 'concept' in this example is also clear; the questionaries used to assess quality of life for pediatric patients after a tonsillectomy performed for the purposes of treating either chronic infection or sleep disordered breathing. While not explicit, the 'context' in this case is quite 'open' in the sense that the quality of life instrument may be used in any setting (primary health care, acute care, or even specialist psychological care or counselling).

An especially important point is that the scoping review may draw upon data from any source of evidence and research methodology, and is not restricted to quantitative studies (or any other study design) alone. This however is not prescriptive; reviewers may decide that particular study designs are beyond the scope of their review or not be appropriate or useful for consideration. For example, the protocol of the above example scoping review specifies that while any type of quantitative study design may be eligible for inclusion, as only psychometrically validated *quantitative* questionnaires were sought, qualitative and gray literature was not considered for inclusion;   In this example however, reports from published randomized controlled trials were considered side by side with observational studies (Kao et al. 2017a). Because of the broad nature of scoping review questions, they are particularly useful for bringing together evidence from disparate or heterogeneous sources.

It is important to highlight the distinction between scoping reviews and "mixed methods" systematic reviews that also rely on evidence from different study designs (Lizarondo et al. 2017). While the aim of a scoping review is to determine what kind of evidence (quantitative and/or qualitative etc.) is available on the topic and to represent this evidence by mapping or charting the data, mixed methods systematic reviews are designed to answer a question or questions based on the synthesis of evidence from for example qualitative, and quantitative research.

When contrasting systematic reviews, scoping reviews and traditional literature reviews, the following table (Table 11.1) from Munn et al. 2018 may be useful (as are the comparisons available in Tricco 2018):

Table 11.1: Defining characteristics of traditional literature reviews, scoping reviews and systematic reviews

For peer review only - http://bmjopen.bmj.com/site/about/guidelines.xhtml

	Traditional Literature Reviews	Scoping reviews	Systematic reviews
A priori review protocol	No	Yes (some)	Yes
PROSPERO registration of the review protocol	No	No*	Yes
Explicit, transparent, peer reviewed search strategy	No	Yes	Yes
Standardized data extraction forms	No	Yes	Yes
Mandatory Critical Appraisal (Risk of Bias Assessment)	No	No**	Yes
Synthesis of findings from individual studies and the generation of 'summary' findings***	No	No	Yes

*Current situation; this may change in time, and we suggest registration/publication of scoping review protocols is critical. Examples of databases where scoping reviews may be registered are: is "Open Science Framework (https://osf.io/) " and "Figshare (https://figshare.com/). **Critical appraisal is not mandatory, however, reviewers may decide to assess and report the risk of bias in scoping reviews depending on the purpose of the review. ***The use of statistical meta-analysis (for effectiveness, prevalence or incidence, diagnostic accuracy, aetiology or risk, prognostic or psychometric data), or meta-synthesis (experiential or expert opinion data) or both in mixed methods reviews is typically not conducted in a scoping review.

While recommendations or implications for research, including for primary research, other scoping reviews, or systematic reviews, may be generated from the results of a scoping review – especially those conducted with the objective of being precursors to systematic reviews (Anderson et al. 2008). Recommendations for practice are difficult due to the fact that a formal assessment of methodological quality of the included sources of evidence of a scoping review is generally not performed. In addition, a formal synthesis is not normally conducted in a scoping review (at least not in the same way for systematic reviews) and as such the methodology is not naturally aligned to establishing practice or policy recommendations. However, if recommendations for practice or policy are developed, it is expected that they will clearly flow from the objectives of the scoping review (Munn et al. 2018 a, b).

For peer review only

11.1.3 The scoping review framework

The framework originally proposed by Arksey and O'Malley (2005) has been influential in the conduct of scoping reviews. Their framework has been further enhanced by the work of Levac and colleagues (2010) (see Table 11.2). Levac and colleagues (2010) provide more explicit detail regarding what occurs at each stage of the review process and this enhancement increases both the clarity and rigor of the review process. Both of these frameworks have underpinned the development of the JBI approach to the conduct of scoping reviews (Peters et al. 2015).

Table 11.2: Scoping review frameworks

	**Arksey and O' Malley framework (2005, p. 22-23)**	**Enhancements proposed by Levac et al. (2010, p. 4-8)**	***Enhancements proposed by Peters et al (2015, 2017, 2020).**
1.	Identifying the research question	Clarifying and linking the purpose and research question	Defining and aligning the objective/s and question/s
2.	Identifying relevant studies	Balancing feasibility with breadth and comprehensiveness of the scoping process	Developing and aligning the inclusion criteria with the objective/s and question/s
3.	Study selection	Using an iterative team appro ach to selecting studies and extracting data	Describing the planned approach to evidence searching, selection, data extraction, and presentation of the evidence.
4.	Charting the data	Incorporating a numerical summary and qualitative thematic analysis	Searching for the evidence
5.	Collating, summarizing and reporting the results	Identifying the implications of the study findings for policy, practice or research	Selecting the evidence
6.	Consultation (optional)	Adopting consultation as a required component of scoping study methodology	Extracting the evidence
7.			Analysis of the evidence
8.			Presentation of the results
9.			Summarizing the evidence in relation to the purpose of the review, making conclusions and noting any implications of the findings

*Consultation of information scientists, stakeholders and/or experts throughout, including in the topic prioritization, planning, execution and dissemination

For peer review only

11.2 Development of a scoping review protocol

As with all well-conducted systematic reviews, an *a priori* protocol must be developed before undertaking the scoping review. A scoping review protocol is important, as it pre-defines the objectives, methods, and reporting of the review and allows for transparency of the process. The protocol should detail the criteria that the reviewers intend to use to include and exclude sources of evidence and to identify what data is relevant, and how the data will be extracted and presented. The protocol provides the plan for the scoping review and is important in limiting the occurrence of reporting bias. Any deviations of the scoping review from the protocol should be clearly highlighted and explained in the scoping review.

Prospective scoping reviewers should be aware that an extension of the PRISMA statement called the PRISMA-ScR is now available (Tricco et al. 2018). Appendix 11.2 to this chapter contains a fillable checklist for authors to check whether their scoping review conforms to this reporting standard. The JBI approach to conducting and reporting scoping reviews described here is congruent with the PRISMA-ScR checklist. Reviewers should also be aware that PROSPERO (the international prospective register of systematic reviews administered by the University of York's Centre for Reviews and Dissemination) states that scoping reviews (and literature reviews) are currently ineligible for registration in the database (Centre for Reviews and Dissemination, n.d. 'inclusion criteria', para. 5). Although this may change in the future, scoping reviews can be registered with the Open Science Framework (https://osf.io/) or Figshare (https://figshare.com/) in the meantime, or their protocols published in some journals, such as the *JBI Evidence Synthesis*.

For peer review only

11.2.1 Title

The title should be informative and give clear indication of the topic of the scoping review. The title of a scoping review should always include the phrase "…:a scoping review" to allow easy identification of the type of document it represents.

For peer review only

11.2.2 Developing the title and question

Title of the scoping review protocol

The title of the protocol (and the subsequent review) should be informative and give a clear indication of the topic of the scoping review. It is recommended that the title should always include the phrase "…: a scoping review" to allow easy identification of the type of document it represents. Correspondingly, protocols should also be identified as such. Titles should not be phrased as questions. This is a simple example of a scoping review protocol title by Kao et al. 2017a:

> "Pediatric tonsillectomy quality of life assessment instruments: a scoping review protocol"

A range of mnemonics for different types of review (and research) questions have been suggested. The "PCC" mnemonic is recommended as a guide to construct a clear and meaningful title for a scoping review. The PCC mnemonic stands for the Population, Concept, and Context. There is no need for explicit outcomes, interventions or phenomena of interest to be stated for a scoping review; however elements of each of these may be implicit in the concept under examination.

The title of the protocol (and subsequent review) should be structured to reflect the core elements of the PCC. Using the PCC mnemonic helps to construct a title that provides potential readers with important information about the focus and scope of the review, and its applicability to their needs. For example, if the review aims to map a range of quality of life instruments (concept) for pediatric patients (population) (Kao et al. 2017a) this should be stated in the title. Including the context in the title (if the context is a central focus of the review) can further help readers to position the review when they are searching for evidence related to their own particular information and/or decision-making needs.

As discussed in further depth below, there should be congruence between the title, review question/s, and inclusion criteria.

Scoping review question(s)

The scoping review question guides and directs the development of the specific inclusion criteria for the scoping review. Clarity of the review question assists in developing the protocol, facilitates effectiveness in the literature search, and provides a clear structure for the development of the scoping review. As with the title, the question should incorporate the PCC elements. A scoping review will generally have one primary question, e.g.

> "What quality of life questionnaires are available for pediatric patients following tonsillectomies with or without adenoidectomies for chronic infections or sleep disordered breathing?"

If that question sufficiently addresses the PCC and adequately corresponds with the objective of the review, sub-questions will not be needed. However, some scoping review questions benefit from one or more sub-questions that delve into particular attributes of Context, Population or Concept. Sub-questions can be useful in outlining how the evidence is likely to be mapped. For example, the primary question above relates to the types of quality of life questionnaires; however, the further sub-questions could be posed to delve into potential particular issues relating to other important details, such as the population (or participants) of interest. For example:

> "What are the ages of the pediatric patients where quality of life questionnaires have been or could be used within the sources of evidence identified for the primary review question?"

Likewise, a sub-question may help to justify mapping the evidence by context, e.g.

> "In what geographical (i.e. countries) and clinical (i.e. primary care, acute care, etc.) contexts have the quality of life instruments included for the primary review question been used?"

For peer review only

11.2.3 Introduction

The introduction should be comprehensive and cover all the main elements of the topic under review. Due to scoping reviews being essentially exploratory, it is not expected that the background covers all the extant knowledge in the area under review. The reason for undertaking the scoping review should be clearly stated together with what the scoping review is intended to inform. The rationale of conducting a scoping review should be clearly articulated and stated in this section before stating the aim.

The suggested length for the introduction section of the scoping review protocol is approximately 1,000 words. This section should detail any definitions important to the topic of interest. The information in the introduction must also be sufficient to put the inclusion criteria in context, including an indication of whether or not there are existing scoping reviews, systematic reviews, research syntheses, and/or primary research papers available on the topic, hence supporting the rationale to conduct the scoping review. While the inclusion criteria section of the protocol (explained below) should contain clear details of each of the Population, Concept and Context elements, the introduction must provide sufficient detail in terms of the rationale for each element. Explaining for example, why only primary care settings are of interest in terms of the context of the review question above.

The introduction should conclude with a statement that a preliminary search for existing scoping reviews (and ideally systematic reviews too) on the topic has been conducted. The date of the search(es) and journals and databases searched and search platforms utilized must be stated,

e.g. JBI Evidence Synthesis, Cochrane Database of Systematic Reviews, Cumulative Index to Nursing and Allied Health Literature (CINAHL), PubMed, Evidence for Policy and Practice Information (EPPI), and Epistemonikos, where relevant. If existing scoping reviews or systematic reviews are available on the topic, a justification that specifies how the proposed review will differ from those already conducted should be detailed. This is so that readers can easily establish what new knowledge or insight the proposed review will contain in relation to existing evidence syntheses.

The introduction should conclude with an overarching review objective that captures and aligns with the core elements/mnemonic of the inclusion criteria (e.g. PCC). The objective of the scoping review should indicate what the scoping review project is trying to achieve. The objective may be broad and will guide the scope of the enquiry. For the title example above, the objective has been phrased:

> “The objective of this scoping review is to investigate quality of life questionnaires available for pediatric patients following tonsillectomies with or without adenoidectomies for chronic infection or sleep-disordered breathing.”

For peer review only

11.2.4 Inclusion criteria

The “inclusion criteria” of the protocol details the basis on which sources will be considered for inclusion in the scoping review and should be clearly defined. These criteria provide a guide for the reader to clearly understand what is proposed by the reviewers and, more importantly, a guide for the reviewers themselves on which to base decisions about the sources to be included in the scoping review. As explained in Section 11.2.2, as for other review types, there must be clear congruence between the tile, question/s, and inclusion criteria of a scoping review.

Types of participants

Important characteristics of participants should be detailed, including age and other qualifying criteria that make them appropriate for the objectives of the scoping review and for the review question.

In some circumstances, participants *per se* are not a relevant inclusion criterion. For example, for a scoping review that is focused upon mapping the types and details of research designs that have been used in a particular field, it may not be useful or within scope to detail the types of participants involved in that research.

Concept

The core concept examined by the scoping review should be clearly articulated to guide the scope and breadth of the inquiry. This may include details that pertain to elements that would be detailed in a standard systematic review, such as the “interventions”, and/ or “phenomena of interest”, and/or “outcomes” (as relevant for the particular scoping review).

For example, the overarching concept of interest for the above scoping review is quality of life questionnaires that are used following tonsillectomies with or without adenoidectomies for chronic infection or sleep-disordered breathing.

Further elements of this overarching concept may be of importance to this review. For example, the format (e.g. paper or web-based) and contents (i.e. assessment domains) of the included instruments. The validity and reliability (i.e. if and how they have been psychometrically tested) may also be of interest for mapping.

Outcomes may also be a component of a scoping review’s “Concept”. If outcomes of interest are to be explained, they should be linked closely to the objective and purpose for undertaking the scoping review. For example, this scoping review could also identify and map the outcomes of quality of life assessments and/or the outcomes of the psychometric testing of the tools themselves.

Context

The “Context” element of a scoping review will vary depending on the objective/s and question/s of the review. The context should be clearly defined and may include, but is not limited to, consideration of cultural factors, such as geographic location and/or specific social, cultural, or gender-based interests. In some cases, context may also encompass details about the specific setting (such as acute care, primary health care or the community). Reviewers may choose to limit the context of their review to a particular country or health system or healthcare setting, depending on the topic and objectives.

The context of the review in the example provided above has not been stated explicitly (i.e. it could be described to be ‘open’) as sources of evidence pertaining to any contextual setting would be eligible for inclusion. However, a context could be imposed to refine the scope of the review in different ways. For example; only within middle-high income countries or only within primary care settings.

T*ypes of evidence sources*

For the purposes of a scoping review, the “source” of information can include any existing literature, e.g. primary research studies, systematic reviews, meta-analyses, letters, guidelines, websites, blogs, etc. Reviewers may wish to leave the source of information “open” to allow for the inclusion of any and all types of evidence. Otherwise, the reviewers may wish to impose limits on the types of sources they wish to include. This may be done on the basis of having some knowledge of the types of sources that would be most useful and appropriate for a particular topic. For example, the scoping review example on quality of life questionnaires available for pediatric patients following tonsillectomies with or without adenoidectomies for chronic infection or sleep-disordered breathing sought quantitative studies, specifically; experimental and epidemiological study designs including randomized controlled trials, non-randomized controlled trials, quasi-experimental, before and after studies, prospective and retrospective cohort studies, case-control studies, and analytical cross-sectional studies. Qualitative studies, reviews, and conference abstracts were excluded.

For peer review only - http://bmjopen.bmj.com/site/about/guidelines.xhtml

11.2.5 Search Strategy

The search strategy for a scoping review should ideally aim to be as comprehensive as possible within the constraints of time and resources in order to identify both published and unpublished (gray or difficult to locate literature) primary sources of evidence, as well as reviews. Any limitations in terms of the breadth and comprehensiveness of the search strategy should be detailed and justified. As recommended in all JBI types of reviews, a three-step search strategy is to be utilized. Each step must be clearly stated in this section of the protocol. The first step is an initial limited search of at least two appropriate online databases relevant to the topic. The databases MEDLINE (PubMed or Ovid) and CINAHL would be appropriate for a scoping review on quality of life assessment tools. This initial search is then followed by an analysis of the text words contained in the title and abstract of retrieved papers, and of the index terms used to describe the articles. A second search using all identified keywords and index terms should then be undertaken across all included databases. Thirdly, the reference list of identified reports and articles should be searched for additional sources. This third stage may examine the reference lists of all identified sources or examine solely the reference lists of the sources that have been selected from full-text and/or included in the review. In any case, it should be clearly stated which group of sources will be examined. A statement should be included of the reviewers' intent to contact authors of primary sources or reviews for further information, if this is relevant. A search for gray (i.e., difficult to locate or unpublished) material might be necessary, and guidance exists on these search strategies. Finally, a complete search strategy for at least one major database should be included as an appendix to the protocol. McGowan et al. (2016) developed an evidence-based guideline for Peer Review of Electronic Search Strategies (PRESS) for systematic reviews, health technology assessments, and other evidence syntheses and recommended the main search to be done by a librarian and peer-reviewed by another librarian.

Reviewers should include the languages that will be considered for inclusion in the review as well as the timeframe, with an appropriate and clear justification for choices. Our strong recommendation is that there are no restrictions on source inclusion by language unless there are clear reasons for language restrictions (such as for feasibility reasons).

As the review question might be broad, authors may find that it is appropriate to search for all sources of evidence (e.g. primary studies and text/opinion articles) simultaneously with the one search strategy. This also depends on the relevance of the evidence sources to the topic under review and its objectives. This approach will lead to a greater sensitivity in the search, which is desirable for scoping reviews.

The search for a scoping review may be quite iterative as reviewers become more familiar with the evidence base, additional keywords and sources, and potentially useful search terms may be discovered and incorporated into the search strategy. If this is the case, it is of the utmost importance that the entire search strategy and results are transparent and auditable. The input of a research librarian or information scientist can be invaluable in designing and refining the search.

For peer review only

11.2.6 Source of evidence selection

The scoping review protocol should describe the process of source selection for all stages of selection (based on title and abstract examination; based on full-text examination) and the procedures for solving disagreements between reviewers. Selection is performed based on inclusion criteria pre-specified in the review protocol. For any scoping review, source selection (both at title/abstract screening and full-text screening) is performed by two or more reviewers, independently. Any disagreements are solved by consensus or by the decision of a third reviewer.

There should be a narrative description of the process accompanied by a flowchart of review process (from the PRISMA-ScR statement) detailing the flow from the search, through source selection, duplicates, full-text retrieval, and any additions from third search, data extraction and presentation of the evidence. The software used for the management of the results of the search should be specified (e.g. Covidence, Endnote, JBI SUMARI). Details of full-text articles retrieved should be given. There should be separate appendices for details of included and a brief mention of the excluded sources, and for excluded sources; reasons should be stated on why they were excluded. We recommend some pilot testing of source selectors prior to embarking on source selection across a team. This will allow the review group to refine their guidance or source selection tool (if one is being used). One framework for pilot testing is described below:

- Random sample of 25 titles/abstracts is selected
- The entire team screens these using the eligibility criteria and definitions/elaboration document
- Team meets to discuss discrepancies and make modifications to the eligibility criteria and definitions/elaboration document
- Team only starts screening when 75% (or greater) agreement is achieved

For peer review only

11.2.7 Data extraction

In scoping reviews, the data extraction process may be referred to as "data charting". This process provides the reader with a logical and descriptive summary of the results that aligns with the objective/s and question/s of the scoping review.

A draft charting table or form should be developed and piloted at the protocol stage to record the key information of the source, such as author, reference, and results or findings relevant to the review question/s. This may be further refined at the review stage and the charting table updated accordingly. Some key information that reviewers might choose to chart are:

1. Author(s)
2. Year of publication
3. Origin/country of origin (where the source was published or conducted)
4. Aims/purpose
5. Population and sample size within the source of evidence (if applicable)
6. Methodology / methods
7. Intervention type, comparator and details of these (e.g. duration of the intervention) (if applicable). Duration of the intervention (if applicable)
8. Outcomes and details of these (e.g. how measured) (if applicable)
9. Key findings that relate to the scoping review question/s.

A template data extraction instrument for source details, characteristics and results extraction is provided in Appendix 11.1 of this chapter, which can be adapted by reviewers to use in their own scoping review protocols and reviews with citation to the JBI methodology guidance for scoping reviews.

For ease of reference and tracking, it is suggested that reviewers keep careful records to identify each source. As reviewers chart each source, it may become apparent that additional unforeseen data can be usefully charted. Charting the results can therefore be an iterative process whereby the charting table is continually updated. It is suggested that the review team become familiar with the source results and trial the extraction form on two or three sources to ensure all relevant results are extracted. This pilot step should be done by at least two members of the review team. This approach is favored by other authors on the conduct of scoping reviews (Arksey & O'Malley 2005; Armstrong et al. 2011; Valaitis et al. 2012). If this approach is not feasible, other approaches (such as one reviewer extracting and another verifying the data) can be considered. The most important thing is authors are transparent and clear in their methods regarding what and how they have extracted data. Once again, pilot testing is recommended.

For peer review only

11.2.8 Analysis of the evidence

There are many ways in which data can be analyzed and presented in scoping reviews. Whilst the next section discusses innovative ways to present the results in scoping reviews, this section discusses analysis of data extracted in scoping reviews.

It is important to point out that scoping reviews do not synthesize the results/outcomes of included sources of evidence as this is more appropriately done within the conduct of a systematic review. In some situations scoping review authors may choose to extract results and descriptively (rather than analytically) map them. For example, a scoping review may extract the results from included sources and map these but not attempt to assess certainty in these results or synthesize these in such a way as we would in systematic reviews.

For many scoping reviews, simple frequency counts of concepts, populations, characteristics or other fields of data will be all that is required. However, other scoping review authors may choose to perform more in-depth analyses, such as descriptive qualitative content analysis, including basic coding of data. This may result in scoping review results providing a summary of data coded to a particular category (i.e. coding and classifying interventions/strategies/behaviors to a behavioral change model or theory). For example, a scoping review on characteristics of indigenous primary health care service models (Harfield et al. 2018) performed content analysis techniques using NVivo as a way to code characteristics into overall categories. Principles of framework synthesis (where you may chart and sort findings/data from papers against an *a priori* identified framework) may also be useful in some scoping reviews (Davy et al. 2016; Carroll 2013; Glegg et al. 2018). It is important to note that qualitative content analysis in scoping reviews is generally descriptive in nature and reviewers should not undertake thematic analysis/synthesis (i.e. JBI's meta-aggregative approach or meta-ethnographic approaches) as this would be beyond the scope of a scoping review and would more appropriately fit within the objectives of a systematic review of qualitative evidence/ qualitative evidence synthesis.

In terms of quantitative data, scoping review authors may choose to investigate the occurrence of concepts, characteristics, populations etc with more advanced methods than simple frequency counts. Whilst this in-depth type of analysis is not normally required in scoping reviews, in other scoping reviews (depending on the aim), review authors may consider some form of more advanced analysis depending on the nature and purpose of their review. It is unlikely that a meta-analysis or interpretive qualitative analysis will be required in scoping reviews.

The way data is analysed in scoping reviews is largely dependent on the purpose of the review and the author's own judgement. The most important consideration regarding analysis is that the authors are transparent and explicit in the approach they have taken, including justifying their approach and clearly reporting any analyses, and as much as possible planned and stipulated a priori.

For peer review only

11.2.9 Presentation of the results

At the time of protocol development, the reviewers should provide some plan for the presentation of results – for example, a draft chart, figure or table (Lockwood et al. 2019). It is recommended that the authors do plan carefully how they intend to present the data extracted from the sources of evidence. Planning at this stage is very useful for an initial sense of what sorts of data might be identified and how best to present that data in relation to the scoping review's objective and question/s. This may be further refined during the review process as the reviewers increase their awareness and consideration of the contents of all of their included sources.

The ultimate purpose of charting the data is to identify, characterize, and summarize research evidence on a topic, including identification of research gaps (Nyanchoka et al. 2019).The results of a scoping review may be presented as a map of the data extracted from the included papers in a diagrammatic or tabular form, and/or in a descriptive format that aligns with the objective/s and scope of the review. The elements of the PCC inclusion criteria may be useful to guide how the data should be mapped most appropriately. In the scoping review example described above, because the objective was to map quality of life questionnaires used for pediatric patients following tonsillectomies with or without adenoidectomies for chronic infection or sleep-disordered breathing, the data may be usefully mapped by a tabular presentation of how the different components of the PCC includes as shown below. Other examples of presenting data from a scoping review can be found below (Table 11.3).

Table 11.3: Example tabular presentation of data for a scoping review

Parameter	Results
**Numbers of publications**	1. Total number of sources of evidence<br>2. Total numbers between 2000 until 2016 (5 Sept)<br>3. Number of publications every year
**Types of studies**	1. Randomized controlled trials<br>2. Non-randomized controlled trials<br>3. Quasi-experimental studies<br>4. Before-and-after studies<br>5. Prospective cohort studies<br>6. Retrospective cohort studies<br>7. Case-control studies<br>8. Cross-sectional studies<br>9. Other quantitative studies
**Population/s identified**	1. Children 0-4<br>2. Children 5-7<br>3. Children 8-10<br>4. Children 11-13<br>5. Children 14-16<br>6. Children 17-18<br>7. Parent/s and/or caregivers<br>8. Health Care professionals<br>9. Not applicable<br>10. Services<br>11. Others (not classified in any of the above)
**Quality of life domains**	1. Physical<br>2. Emotional<br>3. Social<br>4. School/ learning/ education<br>5. Behaviour<br>6. Mental health<br>7. General health<br>8. Family<br>9. Speech<br>10. Other (not classified in any of the above)
**Format/ number of items**	1. Paper-based<br>2. Web-based<br>3. Mobile/tablet (e.g. App)<br>4. Others

For peer review only

The tables and charts may also show results as: distribution of sources of evidence by year or period of publication (depends on each case), countries of origin, area of intervention (clinical, policy, educational, etc.) and research methods. A descriptive summary should accompany the tabulated and/or charted results and should describe how the results relate to the review objective/s and question/s.

The results can also be classified under main conceptual categories, such as: “intervention type”, “population” (and sample size, if it is the case), “duration of intervention”, “aims”, “methodology adopted”, “key findings” (evidence established), and “gaps in the research”. For each category reported, a clear explanation should be provided.

The examples below show various formats of charting the evidence depending on the scoping review question. In the first example (Figure 11.1), the authors aimed to clarify if intense sweeteners are effective tools to lower sugar consumption and maintain a healthy weight or, on the contrary, if these compounds promote weight gain (Mosdøl et al. 2018). This will result in identifying gaps where new systematic reviews or primary research are needed, including which hypotheses, types of intense sweeteners and outcomes that need further assessment.

In the second example (Figure 11.2), the authors were interested to map the types of family involvements in intensive care units and identify their level of involvement from passive to active (Olding et al. 2016. In this case, the authors used conventional content analysis to develop codes inductively through immersion with the text, deriving codes from the data itself rather than coding with preconceived categories.

In the third example (Figure 11.3), the authors used relational analysis to present their results. With this technique, all data from eligible sources were used to identify examples of an Integrated Knowledge Translation (IKT) approach or strategy, enabler, barrier, and outcome. This approach allowed gaps in the IKT literature to be identified (Gagliardi et al. 2015). These data were added to the IKT approaches or strategies, enablers, barriers, and outcomes identified in sources referenced in the background of this manuscript and then compiled in a summary of IKT conditions, influencing factors, and outcomes. This approach made clear what was known and not known about IKT interventions. To further understand knowledge gaps, the authors identified relationships between the characteristics of IKT strategies, contextual factors, and outcomes by categorizing IKT as used in eligible sources of evidence.

The fourth example (Figure 11.4) is derived from a scoping review by Pham et al. 2014. The authors provided an example of a bubble chart for results presentation. This method is frequently used in the engineering sector but could also be employed in many other disciplines. The size of each ‘bubble’ is representative of the number of sources of evidence published in each year.

	Evidence used					Intense sweeteners considered								Comparator					Outcomes presented						
	Primary studies																								
Reference	Human, observational	Human, experimental	Animal, experimental	Cell-cultures	Other reviews	Unspecified or grouped	Acesulfame K	Aspartame	Cyclamate	Saccharine	Sucralose	Stevia	Other	Sugar, other saccharides	Water	Intake levels	Nothing/placebo	Unclear	Body weight	Clinical outcomes	Energy/food intake	Appetite/hunger	Hormone secretion	Intestinal glucose absorption	Microbiome
Bellisle 2007 [31]	x	x	x			x								x	x	x	x		x		x	x			
Mattes 2009 [3]	x	x	x			x	x	x		x	x			x	x		x		x		x	x	x		
Yang 2010 [2]	x	x	x			x	x	x		x	x			x	x		x		x		x	x			
EFSA 2011 [32]	x	x			x	x		x						x					x						
Pepino 2011 [33]	x	x	x	x		x	x	x		x	x	x		x		x	x	x	x	x	x		x	x	x
Sylvetsky 2011 [34]	x	x	x	x		x		x		x	x			x	x	x			x		x	x	x		
Andersen 2012 [35]	x	x			x	x								x		x		x	x		x				
Brown 2012 [36]	x	x	x			x	x	x		x	x	x	x	x	x	x	x	x					x	x	x
Raben 2012 [37]	x	x			x	x	x	x		x	x	x		x	x				x	x	x	x	x		
Swithers 2013 [38]	x	x				x		x			x	x		x	x				x	x			x		
Araurjo 2014 [39]	x	x	x		x	x	x	x		x	x			x		x	x	x	x	x	x		x	x	x
Ferreira 2014 [40]	x	x	x			x		x						x	x	x	x		x		x	x			
Freswick 2014 [41]	x	x				x	x	x			x			x	x		x		x		x				
Gardner 2014 [42]	x	x				x		x						x	x	x	x	x	x	x	x				
Bellisle 2015 [43]	x	x				x								x	x		x					x			
Bruke 2015 [44]	x	x	x			x	x	x			x		x					x					x	x	x
Fernstrom 2015 [45]	x	x	x			x	x	x		x	x	x		x	x	x	x		x			x			
Pepino 2015 [46]	x	x	x	x	x	x	x	x		x	x	x		x	x	x	x			x			x	x	x
Roberts 2015 [47]	x	x				x								x	x				x	x	x	x	x		
Swithers 2015 [48]	x	x	x		x	x		x		x	x			x	x		x	x	x				x		x
Fowler 2016 [49]	x	x	x			x	x	x	x	x	x			x		x	x		x		x				
Glendinning 2016 [50]			x		x	x		x						x			x		x		x		x		x
Nettleton 2016 [51]	x	x	x			x		x		x	x				x	x	x		x	x	x	x	x	x	x
Peters 2016 [52]	x	x	x		x	x		x						x	x	x			x		x	x			
Shearer 2016 [53]	x	x	x		x	x		x		x	x				x	x	x	x	x				x	x	x
Swithers 2016 [54]	x	x	x		x	x				x				x	x				x				x		x

Figure 11.1: Example of data presentation (artificial sweeteners and weight loss/ gain). (Mosdøl et al. 2018)

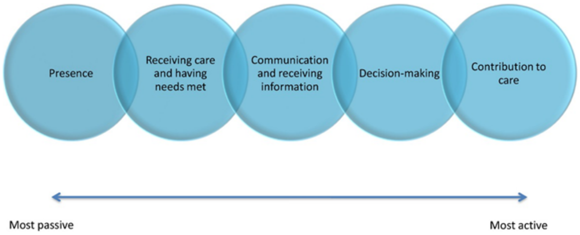

Figure 11:2: Example of data presentation (types of family involvements in intensive care units and level of involvement from passive to active). (Olding et al. 2016)

For peer review only

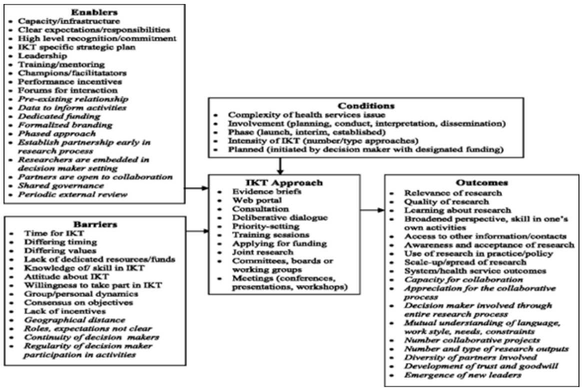

Figure 11.3: Example of data presentation (IKT approaches or strategies, enablers, barriers, and outcomes). (Gagliardi et al. 2015)

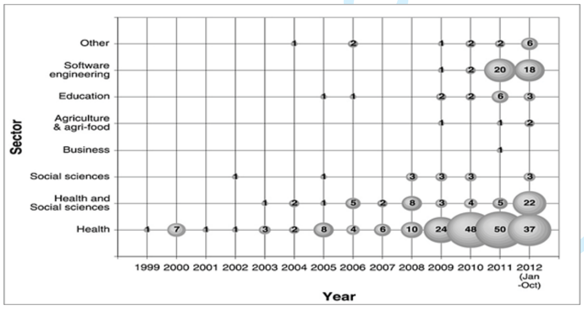

Figure 11:4: Example of data presentation (sources of evidence published by year) (Pham et al 2014)

11.3 The scoping review and summary of the evidence

This section provides further guidance on the components that should comprise the final report of a scoping review and the information that each component should contain. It illustrates how each component of the review is to be managed in the scoping reviews analytical module of JBI's System for the Unified Management, Assessment and Review of Information (SUMARI) software. For authors submitting to *JBI Evidence Synthesis*, please refer closely to the author guidelines available on the *JBI Evidence Synthesis* website.

Specifically, guidance is provided on the following components: outline of the review, inclusion criteria (i.e. PCC), search strategy, extraction, presenting and summarizing the results, and any potential implications of the findings for research and practice. For a traditional systematic review, while deviations from a published review protocol are rare, due to the more iterative nature of a scoping review, some changes may be necessary. These must still be clearly detailed and justified in the methods section of the scoping review if and when they occur.

Please note that more detailed guidance for the conduct of scoping reviews is outlined in the protocol section of this chapter.

For peer review only

11.3.1 Title of the scoping review

The title should be clear, explicit and reflect the core elements of the review. Titles should not be phrased as questions or conclusions and there should be congruence between the title, review objective/question /s, and inclusion criteria. The title should include the phrase: "….: a scoping review". The title should not be more than 25 words for ease of understanding (see example above in Section 11.2.2).

For peer review only

11.3.2 Review authors

Affiliations for each author need to be stated, including the JBI affiliation of each reviewer (if relevant). A valid email address must be provided as contact details for the corresponding author.

For peer review only

11.3.3 Abstract

This section forms a structured abstract of the main features of the scoping review. The abstract should accurately reflect and summarize the review with the main focus on the results of the review. Refer to the author guidelines of the journal you plan to submit for journal related guidance.

The abstract should report the essential elements of the review using the following sub-headings in this order:

- **Objective**: State an overarching review objective structured using the key components of the inclusion criteria (approximately one to two sentences).
- **Introduction**: Briefly describe what the issue is under review and what is already known on the topic (approximately two to three sentences).
- **Inclusion criteria:** Summarize the inclusion criteria as it relates to the type of review being conducted. Present the information in one or two sentences – **NOT** under individual subheadings.
- **Methods:** List the key information sources searched (those that provided the majority of included sources of evidence), any limits placed on the scope of the search (e.g. language), and the date range, or the date of the last search. If the recommended JBI approach (i.e. this chapter) to source selection, data extraction, and the presentation of the data was used. (Alternatively, briefly describe any notable deviations to the methodological approach taken).
- **Results**: The bulk of the abstract should be reserved to convey the main results of the review.
    - As a general rule, report the number and type of included sources and participants, as well as any pertinent source characteristics.
    - Report the main findings and results that have been charted in relation to the review's objective and question/s.
- **Conclusions**: Articulate brief overall conclusions based on the scoping review findings. This should be articulated in a way that directly responds to the objective and question/s of the scoping review. Briefly convey key implications for practice and/or research (if made).

For peer review only

11.3.4 Introduction

The introduction should be comprehensive and cover all of the main elements of the topic under review, as well as important information and why the topic or question of interest lends itself to a scoping review with a clear rationale for conducting the scoping review. The primary objective of the scoping review should be evident in this section as the introduction situates the justification and importance of the question/s posed. While many of these details will already have been addressed in the "Introduction" section of the protocol, reviewers should find that the background information provided with the protocol needs modification or extension following the conduct of the scoping review which now introduces the results of the review project. The introduction should conclude with a statement that a preliminary search for previous scoping reviews (and ideally, systematic reviews) on the topic aligning to the same concept was conducted (state the sources searched e.g. JBI Evidence Synthesis, The Cochrane Database of Systematic Reviews, Campbell Library, etc.).

The introduction should conclude with an overarching review objective that captures and aligns with the core elements/mnemonic of the inclusion criteria (e.g. PCC).

For peer review only

11.3.5 Review question(s)

The primary questions(s) addressed by the scoping review should be stated. It can be followed by sub-questions that relate to differing conceptual foci contained in the scoping review, such as, participant groups, interventions or outcome measures or a more in depth understanding of a particular phenomenon of interest or concept. (See example above in Section 11.2.2)

For peer review only

11.3.6 Inclusion Criteria

This section of the scoping review specifies the basis upon which sources were considered for inclusion in the scoping review. This section should necessarily be as transparent and unambiguous as possible. The inclusion criteria for a scoping review will be contingent on the question/s posed. The PCC should be stipulated (Population, Concept, and Context).

Types of participants

The types of participants in the sources of evidence sought for inclusion should be related to the objectives of the scoping review. The reasons for the inclusion or exclusion of particular participants detailed in this section should be explained clearly in the introduction section of the scoping review.

Concept

The core concept examined by the scoping review should be clearly articulated to guide the scope and breadth of the inquiry. This may include details that pertain to the “interventions” and/or “phenomena of interest” that would be explained in greater detail in a systematic review.

Outcomes may also be a component of a scoping review’s “Concept”. If outcomes of interest are to be explained, they should be linked closely to the objective and the purpose for undertaking the scoping review.

Context

Context will vary depending on the objective/s and question/s of the review. The context should be clearly defined and may include, but is not limited to, consideration of cultural factors, such as geographic location and/or specific racial or gender-based interests. In some cases, context may also encompass details about the specific setting (such as acute care, primary health care or the community).

Types of sources of evidence

The types of sources of evidence to be included in the scoping review should be explained. 'Sources of evidence ' can include any existing literature, e.g. primary research studies, systematic reviews, meta-analyses, letters, guidelines, etc. The source of information may be left “open” to allow for the inclusion of any, and all sources of evidence and rationale for this should be provided. Otherwise, any limits imposed on the types of studies should be detailed and explained. For example, some sources of evidence such as text and opinion papers and letters would not be particularly appropriate or useful in order to meet the objectives and answer the question(s) of particular scoping reviews.

For peer review only

11.3.7 Methods

This section of the review report is reserved for the methods used to conduct the review and should be presented under the relevant subheadings (See Sections 11.3.7.1 - 11.3.7.4), including any deviations from the method outlined in the *a priori* protocol. A reference to the published protocol should be clearly included and cited in this section. In empty reviews for example, this section should not refer to methods that were not performed.

Directly below the Methods heading provide the following information:

- State and appropriately cite the JBI methodology that was employed in the conduct of the review and synthesis.
- Refer to and cite the *a priori* protocol that was published, or accepted for publication (e.g. 'in press'), in the *JBI Evidence Synthesis*.

An example:

"The objectives, inclusion criteria and methods for this scoping review were specified in advance and documented in a protocol." (citation)

For peer review only

11.3.7.1 Search strategy

This section documents how the reviewers searched for relevant sources of information for inclusion in the scoping review. The search strategy must be comprehensively reported and the detailed search strategy for all of the major bibliographic citation databases and other sources that have been searched should be appended to the review. The individual search strategies for every database searched should be presented in sequence and in a consistent format in an Appendix. Clear documentation of the search strategy is a vital component of the scientific validity of any scoping review with justification of the dates of the search included in the protocol. A scoping review should ideally consider sources of evidence (primary studies, textual papers and reviews) both published and unpublished (gray literature). The time frame (start and end dates) chosen for the search should be clearly justified and any language restrictions specified (e.g. “only sources of evidence published in English were considered for inclusion”). Any hand searching of particular relevant journals should be detailed with the journal names and years examined. Author contact, for example, to request access to known but unavailable sources of evidence should also be included along with the outcomes of that contact.

For peer review only

11.3.7.2 Source of evidence screening and selection

The review should describe the actual process of source of evidence screening and for all stages of selection (based on title and abstract examination; based on full-text examination) and the actual procedures used for solving disagreements between reviewers.

For peer review only

11.3.7.3 Data extraction

Extraction of results for a scoping review should include extraction of all data relevant to inform the scoping review objective/s and question/s. Charting table or forms may be used (see Appendix 11.1 for a template tool). A descriptive summary of the main results organized based on the review inclusion criteria must be included. Examples of extraction fields are identified below.

Author/year

Citation details should be consistent throughout the document. The citation details include the name of the first author (Vancouver referencing style) and year of publication.

Objective/s

A clear description of the objective of the paper should be stated.

Participants (characteristics/total number)

The defining characteristics of the participants in included sources should be provided. This includes demographic details and total numbers.

Concept

Data from included sources of evidence in relation to the concept should be extracted and mapped. The concept examined by the scoping review will vary depending on the review, and should be clearly articulated to guide the scope and breadth of the inquiry. This may include details that pertain to the "interventions" and/or "phenomena of interest" that would be explained in greater detail in a systematic review. Outcomes may also be a component of a scoping review's "Concept". If outcomes of interest are to be explained, they should be linked closely to the objective and the purpose for undertaking the scoping review.

Context

Details of the context, such as location of care (acute, primary health care, community, long term care, etc.) or a particular geographical location, should be described. Cultural, social, ethnic, or gender factors may be relevant.

For peer review only

11.3.7.4 Analysis and Presentation of results

The authors should clearly articulate the method(s) used to present the results of the review. These may be a map of the data extracted from the included papers in a diagrammatic or tabular form, and/or in a descriptive format that responds to the questions of the review.

The tables and charts may also show results as: distribution of sources of evidence by year or period of publication (depends on each case), countries of origin, area of intervention (clinical, policy, educational, etc.) and research methods. A descriptive summary should accompany the tabulated and/or charted results and should describe how the results relate to the review objective/s and question/s.

The results can also be classified under main conceptual categories, such as: “intervention type”, “population” (and sample size, if it is the case), “duration of intervention”, “aims”, “methodology adopted”, “key findings” (evidence established), and “gaps in the research”. For each category reported, a clear explanation should be provided.

For peer review only

11.3.8 Results

For peer review only

11.3.8.1 Search results

The presentation of results section should identify how many sources of evidence were identified and selected. There should be a narrative description of the search decision process accompanied by the source of evidence identification and inclusion decision flowchart (see Figure 11.1). This flowchart has been adapted from the PRISMA flowchart developed by Moher et al. (2009). The flow chart should clearly detail the review decision process, indicating the results from the search, removal of duplicate citations, source selection, full retrieval and additions from a third search, and final summary presentation.

The narrative summary should logically describe the aims or purposes of the reviewed sources, concepts adopted and results that relate to the review question/s.

The results may be classified under main conceptual categories such as: “intervention type”, “population” (and sample size, if it is the case), “duration of intervention”, “aims”, “methodology adopted”, “key findings” (evidence established) and “gaps in the research”. For each category, a clear explanation should be provided.

For peer review only

11.3.8.2 Inclusion of sources of evidence

This section should include an overall description of the included sources with reference to the detailed Table of Included Source of Evidence Characteristics in the appendices (the template data extraction tool in Appendix 11.1 can be readily modified by reviewers to suit this purpose). The aim of this section is to provide detail to support the inclusion of each source (paper, study, report, etc.) in the scoping review. For each source, identify the relevance to the scoping review objective and evidence for the review question. Specific results from sources may be highlighted. A summary table of included sources of evidence should be provided in the appendices of the scoping review.

For peer review only

11.3.8.3 Review findings

Presentation of the results may map out the reviewed material in logical, diagrammatic or tabular form, and/or in a descriptive format that aligns specifically with the objective and scope of the review. The tables and charts may show results as: distribution of sources by year or period of publication (depends on each case), countries of origin, area of intervention (clinical, policy, educational, etc.), and research methods.

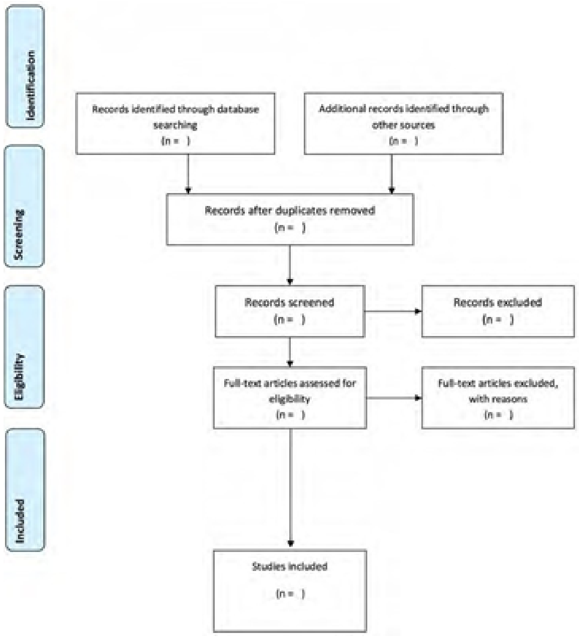

Figure 11.5: Flow diagram for the scoping review process adapted from the PRISMA statement by Moher and colleagues (2009)

11.3.9 Discussion

This section should discuss the results of the review as well as any limitations of the sources included in the scoping review; it should not repeat the results of the review. Results should be discussed in the context of current literature, practice and policy. Scoping reviews are subject to the limitations of any review, relevant sources of information may be omitted and the review is dependent on information on the review question being available. In a scoping review no rating of the quality of evidence is provided, therefore implications for practice or policy cannot be graded.

For peer review only

11.3.10 Conclusions and recommendations

Conclusions

This section should begin with an overall conclusion based on the results. The conclusions drawn should match the review objective/s and question/s.

Implications of the findings for research

This sub-section of the conclusions should include clear, specific implications for future research based on gaps in knowledge identified from the results of the review. Authors may be able to make comments about the future conduct of systematic reviews that may be appropriate, or primary research in the area of interest.

Implications of the findings for practice

If implications for practice are made (note, scoping reviews do not tend to include implications for practice) this sub-section of the conclusions should refer and align to results from the scoping review that can be used to inform practice. It may not be possible to develop implications for practice from the results of a scoping review as no assessment of methodological quality and formal synthesis takes place as part of a scoping review. As such this section may be omitted.

For peer review only

11.3.11 Conflicts and acknowledgements

Details of requirements in these sections are described in Section 1.6. of this Manual.

Conflicts of interest

A statement which either declares the absence of any conflicts of interest or which describes a specified or potential conflict of interest should be made by the authors in this section.

Funding

Authors should provide details regarding any sources of funding for the review project. The role of all funders in the review process, if any, should be explicitly described. If the review is funded, then any potential conflicts of interest or intellectual bias of the funders should be specified in the review. Sources of funding of included sources in the scoping review may also be stated.

Acknowledgements

Any acknowledgements should be made in this section. Acknowledgements should be reserved to individuals who have contributed to the manuscript yet whose contribution does not constitute authorship. Details of the contribution should be included, for example conceptualization, review of draft and feedback. It should also be noted if the scoping review is to count towards a degree award.

For peer review only

11.3.12 References

For publication in the *JBI Evidence Synthesis*, all references should be listed in full using the Vancouver referencing style, in the order in which they appear in the review. Abbreviated journal titles must be used in accordance with the United States National Library of Medicine (2016).

For peer review only

11.3.13 Review appendices

Appendices should be numbered using Roman numerals in the order in which they have been referred to in the body of the text. While reviewers may choose to develop additional appendices for details that are unfeasible to present in the main body of the report, there are three required appendices for a JBI scoping review:

Appendix I: Search strategy

A detailed search strategy for all sources searched must be appended.

Appendix II: Sources excluded following full-text review

A list of sources excluded following full-text review with primary reasons for exclusion

Appendix III: Data extraction instrument

The data extraction instrument used must be appended (see the template in Appendix 11.1)

For peer review only

11.4 Chapter references

Anderson, S, Allen, P, Peckham, S & Goodwin, N 2008, 'Asking the right questions: scoping studies in the commissioning of research on the organisation and delivery of health services', *Health Res Policy Syst*, vol. 6, no. 7.

Arksey, H & O'Malley, L 2005, 'Scoping studies: towards a methodological framework', *Int J Soc Res Methodol*, vol. 8, no. 1, pp.19-32.

Armstrong, R, Hall, BJ, Doyle, J & Waters, E 2011, 'Cochrane update. "Scoping the scope" of a Cochrane review', *J Public Health,* vol. 33, no. 1, pp. 147-50.

Carroll, C., Booth, A., Leaviss, J. and Rick, J., 2013. "Best fit" framework synthesis: refining the method. *B MC medical research methodology*, *13*(1), p.37.

Centre for Reviews and Dissemination n.d., '*PROSPERO International prospective register of systematic reviews'*, CRD The University of York, York, viewed 17 March 2017, <https://www.crd.york.ac.uk /PROSPERO/#aboutpage>.

Crilly, T, Jashapara, A & Ferlie, E 2009, *'Research utilisation and knowledge mobilisation: a scoping review of the literature*'. London: Department of Management, King's College London.

Davis, K, Drey, N & Gould, D 2009, 'What are scoping studies? A review of the nursing literature', *Int J Nurs Stud*, vol. 46, no. 10, pp.1386-400.

Davy, C., Harfield, S., McArthur, A., Munn, Z. and Brown, A., 2016. Access to primary health care services for Indigenous peoples: a framework synthesis. *International journal for equity in health*, 15(1), p. 163.

de Chavez, AC, Backett-Milburn, K, Parry, O & Platt, S 2005, 'Understanding and researching wellbeing: its usage in different disciplines and potential for health research and health promotion', *Health Educ J*, vol. 64, no.1, pp. 70-87.

Decaria, J, Sharp, C & Petrella, R 2012, 'Scoping review report: obesity in older adults', *Int J Obes (Lond)* , vol. 36, no. 9, pp. 1141-50.

Ehrich, K, Freeman, GK, Richards, SC, Robinson, IC, & Shepperd, S 2002, 'How to do a scoping exercise: continuity of care', *Res Pol Plan*, vol. 20, no. 1, pp. 25-9.

Gagliardi, AR, Berta, W, Kothari, A, Boyko, J & Urquhart, R 2015, 'Integrated knowledge translation (IKT) in health care: a scoping review', *Implement Sci*, vol. 11, no. 1, p. 38.

Glegg, S.M.N. and Levac, D.E., 2018. Barriers, facilitators and interventions to support virtual reality implementation in rehabilitation: A scoping review. *PM&R*, *10*(11), pp.1237-1251.

Grant, MJ & Booth, A 2009, 'A typology of reviews: an analysis of 14 review types and associated methodologies', Health Info Libr J, vol.26, no. 2, pp. 91-108.

Harfield, S.G., Davy, C., McArthur, A., Munn, Z., Brown, A. and Brown, N., 2018. Characteristics of Indigenous primary health care service delivery models: a systematic scoping review. *Globalization and health*, 14(1), p.12.

For peer review only - http://bmjopen.bmj.com/site/about/guidelines.xhtml

Joanna Briggs Institute Levels of Evidence and Grades of Recommendation Working Party, The 2014, *Supporting Document for the Joanna Briggs Institute Levels of Evidence and Grades of Recommendation*, JBI, Adelaide, viewed 20 March 2017, <https://joannabriggs.org/sites/default/files/2019-05/JBI%20Levels%20of%20Evidence%20Supporting%20Documents-v2.pdf>.

Kao, SS, Peters, MDJ & Ooi, E 2017a, 'Pediatric tonsillectomy quality of life assessment instruments: a scoping review protocol', *JBI Database System Rev Implement Rep,* vol. 15, no. 5, pp. 1222-7.

Kao, SS, Peters, MDJ, Dharmawardana, N, Stew, B & Ooi, EH 2017b, 'Pediatric tonsillectomy quality of life assessment instruments: a scoping review', *Laryngoscope,* vol. 127, no. 10, pp. 2399-406.

Khalil, H, Peters, M, Godfrey, CM, McInerney, P, Soares, CB & Parker, D 2016, 'An evidencebased approach to scoping reviews', *Worldviews Evid Based Nurs*, vol. 13, no. 2, pp.118-23.

Levac, D, Colquhoun, H & O'Brien, KK 2010, 'Scoping studies: advancing the methodology', *Implement Sci*, vol. 5, no. 69, pp. 1-9.

Liberati, A, Altman, DG, Tetzlaff, J, Mulrow, C, Gøtzsche, PC, Ioannidis, JP, Clarke, M, Devereaux, PJ, Kleijnen, J & Moher, D 2009, 'The PRISMA statement for reporting systematic reviews and meta-analyses of studies that evaluate health care interventions: explanation and elaboration', *PLoS Med*, vol. 6, no. 7, e1000100.

Lizarondo L, Stern C, Carrier J, Godfrey C, Rieger K, Salmond S, Apostolo J, Kirkpatrick P, Loveday H. Chapter 8: Mixed methods systematic reviews. In: Aromataris E, Munn Z (Editors). *Joanna Briggs Institute Reviewer's Manual*. The Joanna Briggs Institute, 2017. Available from https://reviewersmanual.joannabriggs.org/

Lockwood, C, dos Santos, KB and Pap, R 2019, 'Practical guidance for knowledge synthesis: scoping review methods'. *Asian Nurs Res*, vol. 13, no. 5, pp. 287-94.

Marshall-Webb, M, Peters, MDJ, Bright, T & Watson, DI 2018, 'Effectiveness of Nissen fundoplication versus anterior and posterior partial fundoplications for treatment of gastro-esophageal reflux disease: a systematic review protocol', *JBI Database System Rev Implement Rep,* vol. 16, no. 5, pp. 1095-102.

McGowan, J, Sampson, M, Salzwedel, DM, Cogo, E, Foerster, V & Lefebvre, C 2016, 'PRESS peer review of electronic search strategies: 2015 guideline statement', *J Clin Epidemiol*, vol. 75, pp. 40-6.

Moher, D, Liberati, A, Tetzlaff, J & Altman, DG; the PRISMA Group 2009, 'Preferred reporting items for systematic reviews and meta-analyses: the PRISMA statement', *Ann Intern Med*, vol. 151, ed. 4, pp. 264-9.

Mosdøl, A, Vist, GE, Svendsen, C, Dirven, H, Lillegaard, ITL, Mathisen, GH & Husøy, T 2018, 'Hypotheses and evidence related to intense sweeteners and effects on appetite and body weight changes: a scoping review of reviews', *PloS One*, vol. 13, no. 7, e0199558.

Munn, Z, Peters, MD, Stern, C, Tufanaru, C, McArthur, A & Aromataris, E 2018a, 'Systematic review or scoping review? Guidance for authors when choosing between a systematic or scoping review approach', *BMC Med Res Methodol*, vol. 18, no. 1, pp. 143.

For peer review only

Munn, Z, Stern, C, Aromataris, E, Lockwood, C & Jordan, Z 2018b, 'What kind of systematic review should I conduct? A proposed typology and guidance for systematic reviewers in the medical and health sciences', *BMC Med Res Methodol*, vol. 18, no. 1, pp.5-14.

Nyanchoka, L, Tudur-Smith, C, Iversen, V, Tricco, AC & Porcher, R 2019, 'A scoping review describes methods used to identify, prioritize and display gaps in health research', *J Clin Epidemiol*, vol. 109, pp. 99-110.

Olding, M, McMillan, SE, Reeves, S, Schmitt, MH, Puntillo, K & Kitto, S 2016. 'Patient and family involvement in adult critical and intensive care settings: a scoping review', *Health Expect*, vol. 19, no. 6, pp. 1183-202.

Pearson A 2004, 'Balancing the evidence: incorporating the synthesis of qualitative data into systematic reviews 2004', *JBI Reports,* vol. 2, pp.45-64.

Pearson, A, Wiechula, R, Court, A & Lockwood, C 2005, 'The JBI model of evidence-based healthcare', *I nt J Evid Based Healthc*, vol. 3 no. 8, pp. 207-15.

Peters, MDJ, Godfrey, C, Kahlil, H, McInerney, P, Baldini Soares, C & Parker, D 2015, 'Guidance for conducting systematic scoping reviews'. *Int J Evid Based Healthc,* vol. 13, ed. 3, pp. 141-46.

Peters, MDJ 2016, 'In no uncertain terms: the importance of a defined objective in scoping reviews', *JBI Database System Rev Implement Rep,* vol. 14, ed. 2, pp. 1-4.

Peters MDJ, Godfrey C, McInerney P, Baldini Soares C, Khalil H, Parker D 2017, Chapter 11: Scoping Reviews. In: Aromataris E, Munn Z (Editors). Joanna Briggs Institute Reviewer's Manual. The Joanna Briggs Institute, 2017

Pham, MT, Raji, A, Greig, JD, Sargeant, JM, Papadopoulos, A & McEwen, SA 2014, 'A scoping review of scoping reviews: advancing the approach and enhancing the consistency', *Res Synth Methods,* vol. 5, ed. 4, pp. 371-85.

Tricco, AC, Ashoor, HM, Cardoso, R, MacDonald, H, Cogo, E, Kastner, M, Perrier, L, McKibbon, A, Grimshaw, JM & Straus, SE 2016a, 'Sustainability of knowledge translation interventions in healthcare decision-making: a scoping review' *Implement Sci*, ed. 11, vol. 1, p.55.

Tricco, AC, Lillie, E, Zarin, W, O'Brien, K, Colquhoun, H, Kastner, M, Levac, D, Ng, C, Pearson Sharpe, J, Wilson, K, Kenny, M, Warren, R, Wilson, C, Stelfox, HT & Straus, SE 2016b, 'A scoping review on the conduct and reporting of scoping reviews', *BMC Med Res Methodol*, vol. 16, pp. 15.

Tricco, A. C., Soobiah, C., Antony, J., Cogo, E., MacDonald, H., Lillie, E., Tran, J., D'Souza, J., Hui, W., Perrier, L., Welch, V., Horsley, T., Straus, S. E., & Kastner, M. 2016c, 'A scoping review identifies multiple emerging knowledge synthesis methods, but few studies operationalize the method', *J Clin Epi*, *73* , 19–28. https://doi.org/10.1016/j.jclinepi.2015.08.030

Tricco, A.C., Lillie, E., Zarin, W., O'Brien, K.K., Colquhoun, H., Levac, D., Moher, D., Peters, M.D., Horsley, T., Weeks, L. and Hempel, S., 2018. PRISMA extension for scoping reviews (PRISMA-ScR): checklist and explanation. *Annals of internal medicine*, *169*(7), pp.467-473.

National Library of Medicine n.d*., Construction of the National Library of Medicine title abbreviations*, National Library of Medicine, viewed 17 March 2017, <https://www.nlm.nih.gov/tsd/cataloging /contructitleabbre.html>.

For peer review only

Valaitis, R, Martin-Misener, R, Wong, ST, MacDonald, M, Meagher-Stewart, D, Austin, P, Kaczorowksi, J, O-Mara, L, Savage, R, Strengthening Primary Health Care through Public Health and Primary Care Collaboration Team 2012, 'Methods, strategies and technologies used to conduct a scoping literature review of collaboration between primary care and public health', *Prim Health Care Res Dev*, vol. 13, no. 3, pp. 219-36.

Watson, R, Parr, JR, Joyce, C, May, C & Le Couteur, AS 2011, 'Models of transitional care for young people with complex health needs: a scoping review', *Child Care Health Dev,* vol. 37, no. 6, pp. 780-91.

For peer review only

Appendix 11.1 JBI template source of evidence details, characteristics and results extraction instrument

**Scoping Review Details**	
Scoping Review title:	
Review objective/s:	
Review question/s:	
**Inclusion/Exclusion Criteria**	
Population	
Concept	
Context	
Types of evidence source	
**Evidence source Details and Characteristics**	
Citation details (e.g. author/s, date, title, journal, volume, issue, pages)	
Country	
Context	
Participants (details e.g. age/sex and number)	
**Details/Results extracted from source of evidence** (in relation to the concept of the scoping review)	
E.g. Quality of Life Domains assessed	
E.g. Number of items in tool	
E.g. details of psychometric validation of tool	

For peer review only

Appendix 11.2 PRISMA ScR Extension Fillable Checklist

The below checklists can be downloaded for review authors to refer to when reporting scoping reviews to ensure they are in line with the PRISMA scoping reviews extension.

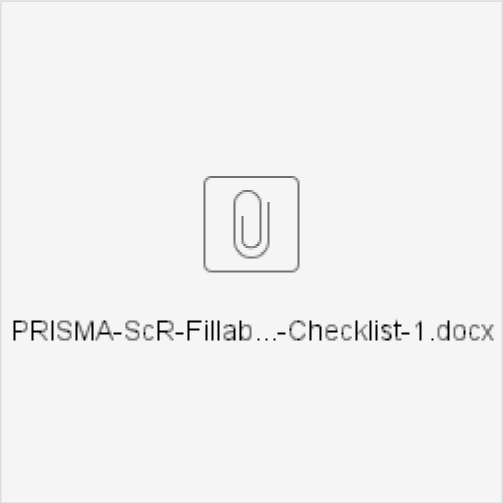

Prisma ScR Fillable Checklist 1 word doc

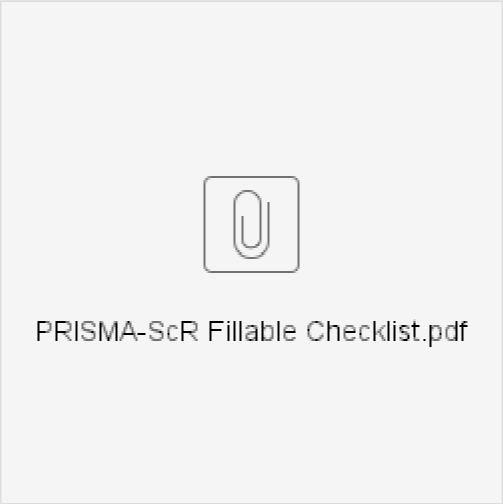

Prisma ScR Fillable Checklist 1 pdf

peer review only

Chapter 12: Systematic reviews of measurement properties

Matthew Stephenson, Dagmara Riitano, Sally Wilson, Jo Leonardi-Bee, Cedric Mabire, Kay Cooper, Dina Monteiro da Cruz, Maria Teresa Moreno-Casbas, Samuel Lapkin.

Acknowledgements: **Michelle Block**

How to cite:

Stephenson M, Riitano D, Wilson S, Leonardi-Bee J, Mabire C, Cooper K, Monteiro da Cruz D, Moreno-Casbas MT, Lapkin S. Chapter 12: Systematic reviews of measurement properties. In: Aromataris E, Munn Z (Editors). *JBI Manual for Evidence Synthesis.* JBI, 2020. Available from https://synthesismanual.jbi.global. https://doi.org/110.46658/JBIMES-20-13

Chapter 12: Contents

- 12.1 Measurement properties of instruments and evidence-based practice
    - 12.1.1 Rationale for a systematic review of measurement properties
- 12.2 Protocol development
    - 12.2.1 Title
    - 12.2.2 Review question(s)
    - 12.2.3 Introduction
    - 12.2.4 Keywords
    - 12.2.5 Inclusion criteria
    - 12.2.6 Search strategy
    - 12.2.7 Study selection
    - 12.2.8 Assessment of methodological quality
    - 12.2.9 Data extraction
    - 12.2.10 Data synthesis
    - 12.2.11 Assessing certainty in the findings (i.e. GRADE approach)
- 12.3 The systematic review
    - 12.3.1 Title
    - 12.3.2 Abstract
    - 12.3.3 Keywords
    - 12.3.4 Summary of findings
    - 12.3.5 Review question(s)
    - 12.3.6 Introduction
    - 12.3.7 Inclusion criteria
    - 12.3.8 Methods
    - 12.3.9 Results
    - 12.3.10 Discussion
    - 12.3.11 Conclusions
- 12.4 Glossary of terms
- 12.5 Chapter references
- Appendix 12.1 Data extraction / characteristics of included studies template
- Appendix 12.2 Table of results template

For peer review only

12.1 Measurement properties of instruments and evidence-based practice

A measurement is the quantity of an attribute and is used to produce data for quantitative studies. An instrument is the “device” used to collect a measurement. The term ‘instrument’ is broad and can include a questionnaire (e.g. patient-reported outcome such as quality of life), observation (e.g. the result of a clinical examination), scale (e.g. a visual analogue scale), laboratory test (e.g. blood test) or image (e.g. ultrasound or other medical imaging) (Polit & Beck, 2014). Measurements can be subjective or objective and data collected in any setting, in every age of client, carer or healthcare professional. Further, instruments may be either unidimensional, measuring one construct e.g. attitude, or multidimensional, measuring complex constructs such as family centered care for example. Instruments may consist of subscales that measure different aspects of the overall construct. It is important to ensure the instrument, and therefore the measurement it provides, measures what it is supposed to measure, is consistent, and is responsive to changes over time.

Psychometrics and clinimetrics are the construction and validation of measurement instruments, and the assessment of these instruments as valid and reliable forms of measurement (Ginty, 2013). The study of instruments that consist of items of equal weighting (e.g. items of a questionnaire that each make up 1 point of the total score) is known as psychometrics. Clinimetrics is an associated term used to describe the study of instruments where items may be major or minor; or present or absent, such as the revised Jones criteria for rheumatic fever (Gewitz et al., 2015). It has been suggested that clinimetrics does not constitute a separate approach, but rather is a subset of psychometrics (Streiner, 2003). As both types of instruments are important in clinical practice and research, we will refer to measurement properties throughout this chapter that incorporate both psychometric and clinimetric characteristics.

For peer review only

12.1.1 Rationale for a systematic review of measurement properties

Systematic reviews synthesize the best available evidence and are the keystone of evidence-based practice (Aromataris & Pearson, 2014). A well-conducted systematic review provides a robust, transparent, rigorous method of answering a research question and there are several reasons why a research question relating to the measurement properties of instruments may be asked.

Clinicians and researchers frequently want an instrument to measure a particular attribute that best suits their context. This may require synthesis of published and unpublished results of psychometric testing of instruments claiming to measure the specific attribute e.g. caregiver burden (Whalen & Buchholz, 2009) to find the best instrument for their purpose. Alternatively, there may be a need to establish the relevance and applicability of a specific instrument prior to implementing research findings into practice or using the instrument in research. Conducting a systematic review of the measurement properties of instruments may provide clinicians and researchers with the gold standard instrument and identify settings or contexts in which instruments should or should not be used. Alternatively, a systematic review of measurement properties may identify a gap in knowledge demonstrating where a reliable, valid instrument needs to be developed.

There are many measurement properties to consider when seeking answers to the above questions. However, the predominant domains into which these properties fit are validity (measure what it is supposed to measure), reliability (consistency), and responsiveness (ability to detect change over time). Several publications are available to assist systematic reviewers to identify the domains and items that should be considered when assessing the quality of studies reporting measurement properties of instruments and how best to synthesize them (Francis et al., 2016; Mokkink et al., 2018a; Polit & Yang, 2016; Prinsen et al., 2018).

An international panel of experts in health measurement properties used a consensus approach to develop a taxonomy, terminology, and definitions of measurement properties. The panel also sought consensus on the relevancy of evaluating each property when appraising an instrument. The resultant list, COnsensus-based Standards for the selection of health status Measurement INstruments (COSMIN), formed standards which can be used for the selection of health measurement instruments, peer reviewing a manuscript, designing or reporting a study on measurement properties, or for educational purposes (Mokkink et al., 2010b). A subsequent checklist applying a four-point scale was developed to enable quantifying the overall methodological quality of a study on measurement properties (Terwee et al., 2012), which was updated in 2018 (Mokkink et al., 2018a). The COSMIN initiative has also developed guidelines for systematic reviews of measurement properties for patient-reported outcome measures (PROMs) (Prinsen et al., 2018).

A similar taxonomy was developed (Polit, 2015) and subsequent debate regarding how to organize the measurement properties in domains and what measurements of error should be included has followed (Mokkink et al., 2016; Polit, 2016). Authors acknowledge that the work in this area is evolving. An alternative checklist was created to operationalize measurement characteristics of instruments measuring patient reported outcomes (Francis et al., 2016). The authors viewed the four domain, 119 item COSMIN checklist as too complex and offered a checklist of six domains with a total of 18 items, that could be used by both those with measurement theory expertise and less experienced clinicians (Francis et al., 2016). However, members of the COSMIN initiative have identified shortcomings of this reduced checklist, which may introduce bias in the ratings. One argument is that the criteria presented in the shortened checklist are not detailed enough to provide a transparent and systematic rating of the quality of an instrument (Terwee et al., 2016b). While both checklists by the COSMIN initiative and Francis et al. (2016) were established using patient reported outcomes, they have utility for assessing measurement properties of other instruments. There are several other critical appraisal tools developed for appraisal of studies of measurement properties, but the COSMIN checklist remains the benchmark in this field (Rosenkoetter & Tate, 2017).

JBI has been a leader in synthesizing findings from multiple studies within the framework of evidence-based healthcare. JBI has published guidelines for systematic reviews of many research designs, therefore, it is timely to consider the guidelines available for conducting systematic reviews of measurement properties and provide guidance to systematic reviewers who work within the JBI framework. There are some similarities between systematic reviews of measurement properties and diagnostic test accuracy systematic reviews (see JBI Manual for Evidence Synthesis – Chapter 9), however diagnostic test accuracy reviews specifically focus on the comparison of two tests (index test and reference test) to establish accuracy in identifying the presence or absence of a condition. On the other hand, systematic reviews of measurement properties are used to establish validity, reliability, and responsiveness of one or more instruments that may be used to measure a wide variety of outcomes.

This chapter outlines and describes guidance for synthesizing evidence related to the measurement properties of instruments and contributes to the emerging field of systematic review methodologies. The systematic review of studies to answer questions of validity, reliability, and responsiveness adheres to the same basic principles of systematic reviews of other types of data. An a priori protocol must precede and inform the conduct of the systematic review, comprehensive searching must be performed, and critical appraisal of eligible studies must be carried out by two independent reviewers, followed by data extraction and synthesis. These steps will be further discussed in the following sections of this chapter. Additionally, reviewers should refer to two statements/checklists: one for transparent reporting of a systematic review of various research study designs (Preferred Reporting Items for Systematic Reviews and Meta-analyses (PRISMA)) (Moher et al., 2009) and one for the COSMIN guidance for systematic reviews of patient-reported outcome measures, which provides guidance on standards for methodological quality of studies reporting measurement properties (Mokkink et al., 2018a; Prinsen et al., 2018).

For peer review only - http://bmjopen.bmj.com/site/about/guidelines.xhtml

12.2 Protocol development

An *a-priori* protocol must be developed before undertaking a JBI systematic review of measurement properties. The purpose of the protocol is to provide the rationale for the review, define the scope of the review and key concepts, establish explicit inclusion and exclusion criteria to minimize the risk of introducing bias in the review, and detail the methods to be used when conducting the review. Systematic review protocols should be publicly accessible e.g. published in a journal and/or registered on PROSPERO (https://www.crd.york.ac.uk/prospero/).

This section outlines the components of a systematic review protocol of measurement properties and provides guidance on the information that each component should contain. The guidance for developing the protocol for a JBI systematic review of measurement properties is organized to meet the structure/template requirements for submission to *JBI Evidence Synthesis*. For further details regarding formatting and submission requirements please see the journal website here.

For peer review only

12.2.1 Title

The title should be informative and give clear indication of the topic of the systematic review. The title of the protocol should always include the phrase "a systematic review protocol" and should also explicitly state that it is on "measurement properties", to allow easy identification of the type of systematic review it represents. Titles should not be phrased as questions or conclusions and there should be congruency between the title, review objectives/questions and inclusion criteria.

The following are examples of titles:

Screening instruments for frailty in palliative care settings: a systematic review protocol of measurement properties
Self-report instruments to identify anxiety in pregnancy: a systematic review protocol of measurement properties
Instruments for measuring functional performance following stroke: a systematic review protocol of measurement properties
The Confusion Assessment Method for detecting delirium in medical and surgical inpatients: a systematic review protocol of measurement properties

For peer review only

12.2.2 Review question(s)

The specific review question(s) must be clearly stated, as the questions guide and direct the development of the specific review criteria. Clarity and specificity in the review questions assists in developing a protocol, facilitates more effective searching, and provides a structure for the development of the full review. The review question(s) should be consistent with the title and direct the development of the specific inclusion criteria from a clearly identifiable PICO.

For peer review only

12.2.3 Introduction

Emphasis should be placed on developing a clear and meaningful introduction section for the systematic review protocol, which is comprehensive and covers all the main elements of the topic under review. The introduction should aim to situate the context of the review for an international readership and key terms important to the topic of interest should be clearly defined. The information in the introduction section must also be sufficient to put the inclusion criteria into context. Where possible, refer to existing international literature to support and inform the inclusion criteria. In addition, the introduction section should provide justification for the conduct of the review and indicate how the proposed review will make a unique and important contribution. The suggested length for the introduction section of the review protocol is approximately 1000 words.

The introduction section should include a statement that a preliminary search for existing systematic reviews on the topic has been conducted (state the databases searched e.g. Cochrane Library, *JBI Evidence Synthesis*, COSMIN Database of Systematic Reviews, PubMed, and PROSPERO). If there is an existing systematic review available on the topic, a justification that specifies how the proposed review will differ from those already conducted should be detailed. The introduction should conclude with an overarching review objective that captures and aligns with the core elements /mnemonic (i.e. PICO) of the inclusion criteria.

For peer review only

12.2.4 Keywords

List a maximum of five keywords in alphabetical order, separated by a semi-colon and a space. Note: these are for the purposes of meta-data and indexing, and not related to the search strategy.

For peer review only

For peer review only - http://bmjopen.bmj.com/site/about/guidelines.xhtml

12.2.5 Inclusion criteria

This section of the protocol details the basis on which studies will be considered for inclusion into the systematic review and should be as clear and unambiguous as possible. The mnemonic PICO is recommended for setting the inclusion criteria for systematic reviews of measurement properties:

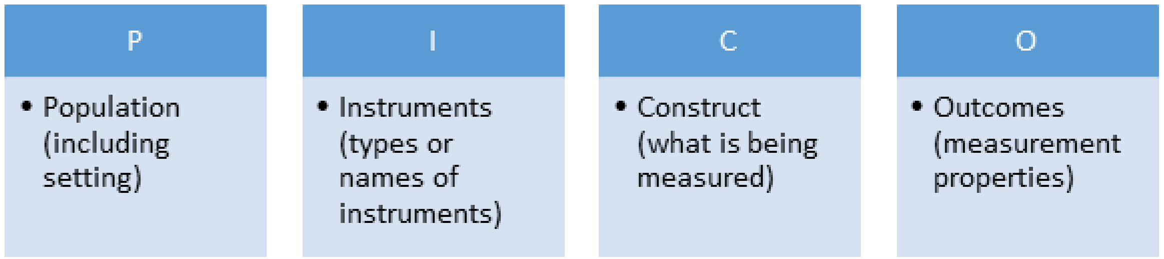

Population

Important characteristics of the population should be detailed, including age and other qualifying criteria (e.g. disease status, occupation, etc.) that make them appropriate for the objectives of the systematic review and match the review question. The reasons for the inclusion or exclusion of participants should be explained in the introduction and be based on clear justifications. When assessing the measurement properties of instruments, the setting in which the instrument is used is often important. Hence, defining characteristics of the population for a review should also include details of the setting of interest, such as acute care, primary health care, or the community.

Instruments and Construct

The term 'instrument' refers to a specific named instrument, for example the Beck Depression Inventory, while the term 'construct' refers more broadly to what is being measured, in this case, depression. Reviewers may choose to nominate specific named instruments that they wish to assess. Alternatively, if reviewers wish to assess all or commonly used instruments measuring that construct they may specify the type(s) of instruments that are of interest for the review (e.g. patient-reported, clinician-reported, performance-based, etc.). For clarity, the construct of interest must always be included even if specific instruments of interest are named, as some instruments may measure more than the construct of interest.

The example titles presented above include three cases in which a construct is referred to i.e. "screening instruments for frailty", "self-report instruments to identify anxiety", and "instruments for measuring functional performance". The fourth example is where a specific named instrument, the 'Confusion Assessment Method', is specified.

In principle, a modified measurement instrument should be treated as a new instrument. Studies utilizing a modified version of an instrument can be included in the review, although it is important to extract and report details of the modifications made.

Outcomes (measurement properties)

'Outcomes' for systematic reviews of measurement properties refer to the psychometric properties of the measurement instruments assessed in the review. Sufficient detail regarding the measurement properties of interest must be provided, with definitions. It may also be useful to provide examples of how the measurement properties of interest are commonly assessed and reported e.g. internal consistency (assessed by Cronbach's alpha), criterion validity (assessed by area under the curve [AUC]). See section 12.4 Glossary of Terms for definitions of measurement properties.

This section should focus on describing the relevant measurement properties which are of interest to the systematic review. As a minimum, measures of reliability and validity should be included. Measures of reliability could include internal consistency and measurement error (Mokkink et al., 2018b). Measures of validity could include content validity, face validity, structural validity, hypothesis testing, cross-cultural validity, and criterion validity (Mokkink et al., 2018b). Measures of responsiveness may be included if the research question of the systematic review is concerned with detecting changes in the construct over time.

Reliability measures are typically reported as either Cronbach's alpha coefficients, intra-class correlation coefficients (ICC), weighted or un-weighted Kappa statistics, standard error of measurement (SEM), limits of agreement (LoA), smallest detectable change (SDC), concordance correlation coefficients, or goodness of fit statistics.

The statistics that are reported for validity measures depend on which measure of validity is being reported. Content validity is evaluated by relevant items for the construct (e.g. Content Validity Index (Lynn, 1986)), purpose, target population, the comprehensiveness of the instrument, and floor or ceiling effects (if available). Construct validity is evaluated by factor analysis and measures are comparative fit index (CFI), Tucker-Lewis index (TLI), root mean square error of approximation (RMSEA) and standardized root mean residuals (SRMR). The differential item functioning (DIF) could be reported for cross-cultural validity. Hypothesis testing measures are typically reported as either absolute or relative differences or correlations between two instruments or two groups of participants. Criterion validity measures are typically reported as either correlations, area under Receiver Operating Curves (ROC), or as sensitivity and specificity.

peer review only

Responsiveness measures are typically reported as either absolute or relative correlations or differences of the change scores, area under the Receiver Operating Curve (ROC), or sensitivity and specificity.

Types of studies

In this section, the types of studies which will be considered for inclusion in the review are described. Any quantitative study design may be eligible for inclusion in a systematic review of measurement properties, however it is suggested to prioritize studies that focus on the development and/or validation of measurement instruments. For example, it is recommended to exclude studies that only use the measurement instrument as an outcome measure. The reason for this is that identifying all studies that have simply used a particular instrument as an outcome measure would require an extended search strategy and significantly increase the work involved at the study selection/full-text screening stage. Furthermore, data on measurement properties in studies that only use the instrument as an outcome measure are likely to be of limited value to the systematic review.

It is recommended to exclude studies that duplicate validation data of an instrument in a previous study i.e. do not present new measurement property data.

For peer review only

12.2.6 Search strategy

For a systematic review of psychometric properties, the specific aim of the search strategy is to locate studies that have described the development or validation process of a measurement instrument or those that have evaluated a measurement instrument's psychometric properties. Research has found that a poor search strategy is a common and major methodological weakness of systematic reviews of measurement instruments (Mokkink et al., 2009; Terwee et al., 2016a), which can threaten the validity of a reviews' findings and its role as a reliable source of evidence-based guidance for choosing appropriate instruments for use in research or clinical practice (Mokkink et al., 2009; Prinsen et al., 2018). Accordingly, the development of a comprehensive search that locates all the relevant and available literature on the topic of interest is fundamental to the conduct of a complete and high quality review. Unfortunately, locating studies that have reported or evaluated the psychometric properties of a measurement instrument can be challenging due to the poor indexing of such studies; the heterogeneity in terminology used to describe measurement properties; and poor reporting by authors, who frequently omit commonly used measurement property terms from the titles and abstracts of their published studies (Terwee et al., 2009).

Search filters

The COSMIN initiative have developed two validated search filters to aid researchers in finding studies of measurement properties in PubMed (a sensitive search and a precise search) (Terwee et al., 2009). COSMIN suggest researchers conducting systematic reviews of psychometric properties use the sensitive search filter, as it was designed to retrieve a high number of relevant articles (sensitivity: 97.4%; precision: 4.4%) (Terwee et al., 2009). Although the precise search filter is more specific, it is more likely to miss relevant studies (sensitivity of 93.1%; precision of 9.4%) (Terwee et al., 2009). The sensitive search filter has been translated for use in EMBASE, for MEDLINE using OVID, and two translations have been developed for CINAHL; however, none of translations have been validated. All search filters can be found on the COSMIN website (https://www.cosmin.nl/tools/pubmed-search-filters/). The search filters contain a combination of search terms (free text and index terms) that capture relevant measurement properties and should be used together with search terms defined by the review team for the population, construct and/or instrument/type of instrument of interest (Terwee et al., 2009). A search filter for use with PubMed and Ovid to find studies evaluating patient-reported outcome measures (PROMs) has also been developed by the Patient-Reported Outcomes Measurement Group (PROM Group), at the University of Oxford (University of Oxford), and can be used for the 'type of instrument' concept in the search strategy.

Search strategy development

The development of a search strategy involves identifying key search terms and synonyms for each major concept in the inclusion criteria mnemonic for psychometric reviews (Population; Instrument name/Type of Instrument; Construct, Outcomes [measurement properties]). The selection of search terms for each concept (i.e. search string) should be developed using an iterative process that involves adjusting terms and performing test searches. The final search should contain a combination of both free text words and index terms (e.g. MeSH) to improve the sensitivity of the search, i.e. its ability to recall relevant studies. The type and number of key search terms to be included will depend on the topic of interest and the size of its evidence base. For example, the population or construct of interest may involve very specific or very broad domains of interest, which will impact on the number of relevant records retrieved (sensitivity/specificity of the search). How the search strings for the major concepts (Population, Instrument name/Type of instrument, Construct, Measurement properties) are combined also depends on the type of systematic review of measurement instruments being conducted (Mokkink et al., 2018b). In their user manual, COSMIN provide a schematic that shows how search strategies for different types of reviews of patient-related outcome research should be constructed (Mokkink et al., 2018b). For example, if a systematic reviews seeks to evaluate all PROMS (validated or not), search terms related to the 'Measurement properties' concept should not be used in the search strategy (Mokkink et al., 2018b).

As a wide variety of terms related to the 'Type of instrument' concept exist and are not always reported in the abstract, these terms should typically be excluded from the search to avoid the high risk that relevant studies will be missed; however, if the systematic review seeks to locate PROMS, the PROMS filter can be included in the 'Type of instrument' concept in the search strategy. If possible, reviewers should select their search terms and develop their strategy in consultation with an expert research librarian or information specialist, whose contribution should be acknowledged (with permission) in any related publications. When considering possible search terms, reviewers may wish to familiarize themselves with the different types of instruments available, which differ in content and in their intended purpose or application. The PROM Group (University of Oxford) has classified measurement instruments into seven major categories with examples (disease-specific; population-specific; dimension-specific; generic; individualized; summary items; and utility measures).

The JBI recommend a three-phase search process that should be undertaken in the development of a comprehensive search strategy:

- **Phase one** involves conducting an initial limited search in a selected database (e.g. PubMed) to find articles on the topic of interest. The keywords (i.e. text words) used in the titles and abstracts of these articles, and the index terms used to describe and categorize them, should be identified, and subsequently, used to develop a full search strategy. The search strategy must be adapted and individualized for every selected database as each one uses its own controlled vocabulary (i.e. index terms).
- **Phase two** involves running the database-specific searches in each of the bibliographic databases and information sources selected and reported in the protocol.
- **Phase three** involves scanning the reference lists of all studies selected for critical appraisal to identify any additional relevant studies.

Information sources

The review protocol should list all the information sources that will be searched for the systematic review: electronic bibliographic databases; search engines; relevant websites; references lists of eligible studies; pre-selected journals, and direct contact with experts in the field who may help to identify measures under development or articles reporting on instruments that assess the construct of interest. The search should be conducted across a comprehensive range of relevant and content-specific (i.e. construct or population of interest) information sources appropriate for the topic and the type of studies being sought. The search should include MEDLINE and EMBASE at a minimum and should include sources of both published and gray or unpublished literature. Examples of other commonly searched databases include CINAHL, PsycINFO, Web of Science, Scopus, Dissertations and Theses Global (gray literature), and WorldCat (gray literature). The PROMS Group has also collated a list of information sources specific to PROMS (e.g. organizations and research groups; journals; royal colleges and relevant links) on their website (University of Oxford) and the Health and Psychosocial Instruments (HaPI) database contains information on behavioral measurement instruments from journal abstracts covering the health and psychosocial sciences. Databases should be searched from the date of inception until the present time unless a valid justification for placing a limit of the publication date can be provided.

Reporting a search strategy

For peer review only - http://bmjopen.bmj.com/site/about/guidelines.xhtml

It is important that the search strategy used to find eligible studies is reported in a detailed and transparent manner, such that other researchers could repeat it, if required. The review protocol should describe in detail the proposed search strategy (three phase approach), the complete list of information sources to be searched, the timeframe for the search, and any language and date restrictions with appropriate justifications. The full search strategy for at least one major electronic database (such as PubMed) should be provided in an appendix and should report the name of the information source and the platform or service provider, for example, CINAHL (via Ovid); all search terms (both free text and index terms) and how they are to be combined using Boolean logic (if applicable); the use of database specific truncation and wild cards; all limits placed on the search (e.g. publication date, publication type, etc.); and the number of records retrieved by the search.

For peer review only

12.2.7 Study selection

This section should describe the process of study inclusion for all stages of selection (based on title and abstract examination; based on full text examination) and the procedures for solving disagreements between reviewers. The software used for the management of the results of the search and study selection/grouping should be specified (e.g. Covidence, Endnote). Selection is performed based on inclusion criteria (See Section 12.2.4) pre-specified in the review protocol. In a systematic review, study selection (both at title/abstract screening and full text screening) ideally should be performed by two or more reviewers, independently. Any disagreements are solved by consensus or by the decision of a third reviewer. Reviewers are encouraged to read the article by Porritt et al. (2014) regarding study selection and critical appraisal.

For peer review only

12.2.8 Assessment of methodological quality

Studies that are eligible for inclusion in a systematic review of measurement properties must be assessed for methodological quality. Two reviewers should conduct independent appraisals and then reach consensus on study ratings. A third reviewer may be consulted where necessary. We recommend using and citing the COSMIN Risk of Bias checklist (Mokkink et al., 2018a). The COSMIN Risk of Bias checklist can be downloaded from the COSMIN website: https://www.cosmin.nl/tools/guideline-conducting-systematic-review-outcome-measures.

The COSMIN Risk of Bias checklist is modular, containing 10 boxes with standards for PROM development and nine measurement properties (see Table below). The PROM development box only needs to be completed for studies that report on the development of a measurement instrument. Likewise, each measurement property box only needs to be completed if a particular study has assessed that property. It may be useful for reviewers to create a table following the template below to identify which boxes need completing for each study.

Instrument	Included Studies	Outcomes									
		PROM Development	Content Validity	Structural validity	Internal consistency	Cross-cultural validity /measurement invariance	Reliability	Measurement error	Criterion validity	Hypotheses testing for construct validity	Responsiveness
Instrument A	Study 1	x	x		x	x	x				
	Study 2		x		x	x					
	Study 3		x		x		x				
Instrument B	Study 4	x	x	x	x						
	Study 5		x	x	x					x	
	Study 6		x	x	x	x	x				
Instrument C	Study 7		x		x						x
	Study 8		x		x			x			

Each criteria in the checklist is rated as either very good, adequate, doubtful, or inadequate. Studies then receive an overall rating for methodological quality, taking the lowest rating of any criteria (i.e. the worst score counts). A spreadsheet is also available for download via the COSMIN website which can be used to enter and organize the appraisal results (see 'help organizing your COSMIN Risk of Bias ratings' within the guideline for systematic reviews section: https://www.cosmin.nl/tools/guideline-conducting-systematic-review-outcome-measures/).

For peer review only

12.2.9 Data extraction

A data extraction template should be used to extract relevant data from the included studies. A suggested data extraction template is presented below (also in Appendix 12.1), which may be modified to suit the specific data for the systematic review. It is recommended that data extraction be conducted independently by two reviewers to minimize errors.

Information on which instrument(s) the study has assessed should be extracted, including specific information about sub-scales if only parts of a larger instrument are utilized. In addition, it is important to detail the construct assessed as some instruments may be used to assess multiple constructs. The country and language in which an instrument is administered should be extracted, as a translated version of an instrument may be considered as a separate instrument to the original language version and may have different psychometric properties. The mode of administration for an instrument (e.g. online, paper-based, etc.) is important to extract to facilitate comparison between studies. As previously mentioned in the inclusion criteria section (12.2.5), the setting in which an instrument is used is often important and information on the setting/context as well as the participants (e.g. study inclusion/exclusion information and numbers of participants) in the study should be extracted.

The results that should be extracted are data on the measurement properties of interest. If a large number of measurement properties are reported in included studies, reviewers may choose to create a separate table to enter the measurement property data (see Appendix 12.2).

Study	Instrument(s) assessed	Construct assessed	Country/ language	Mode of admin	Setting/ Context	Participants	Results (measurement properties)	Comments

Instrument feasibility and interpretability

In addition to the data on the measurement properties of interest, it is important to extract data (where possible) in relation to the feasibility and interpretability of instruments. This information is important to consider when selecting the most suitable instrument for a specific purpose. Feasibility and interpretability are not formal measurement properties and can only be assessed descriptively, not quantitatively evaluated. While the measurement properties of an instrument may be robust, an instrument may not be usable for a given context or population. Reviewers should consider feasibility and interpretability when making recommendations or suggestions about the suitability of particular measurement instruments.

Feasibility allows a researcher to use judgment in selecting the best instrument for a given research context and/or population beyond the strength of the measurement properties. For example, if a researcher is administering a compendium of instruments in a study, it may not be feasible to use lengthy instruments. Instead, the researcher may need to choose instruments that are psychometrically sound while not adding to the burden of participating in the study. Therefore, especially when there are multiple instruments measuring the same construct, it is essential to examine specific feasibility characteristics to determine the practicability of an instrument. The table below includes the key characteristics relating to feasibility that reviewers should aim to identify and extract for each measurement instrument of interest.

Feasibility Characteristic	Interpretation of Feasibility Characteristic
Number of Questions/Length	How many questions are included in the instrument?
Type of Questions	Likert scale numerical, Likert scale word, etc.
Who Completes Instrument	Is the instrument completed by the researcher or the participant?
Time to Complete Instrument	How long does it take to complete the instrument?
Time to Complete Scoring of Instrument	How long does it take to score the completed instrument? How complex is the scoring?
Literacy level	What grade level is the instrument written at?
Language Translation	Is this instrument available in other languages? If so, which languages?
Ease of Administration	How easy it is to administer the instrument? Does it take a great deal of explanation?
Cost of Instrument	Is the instrument free to use or is there a cost to use/score the instrument?
Availability of Instrument	Where can the instrument be located? Is the instrument copyrighted?

Interpretability refers to the qualitative meaning that can be assigned to a measurement instrument's quantitative score or change score (Mokkink et al., 2010a). Ideally, a measurement instrument will provide an outcome that is easily understood and can be used to judge the clinical meaningfulness of the result. The interpretation of single scores can be enhanced by reporting information on the distribution of scores in a study population, which may reveal clustering of scores and indicate floor and ceiling effects. Change scores can be more easily interpreted by reporting minimally important change (MIC) values. The COSMIN methodology provides further examples of information to extract regarding interpretability of measurement instruments (Mokkink et al., 2018b).

For peer review only - http://bmjopen.bmj.com/site/about/guidelines.xhtml

12.2.10 Data synthesis

The purpose of data synthesis of measurement properties is to evaluate whether the measurement properties for specific instruments are adequate for the intended use of the instrument. Data for each measurement property for each instrument of interest should be synthesized and evaluated.

Homogeneity of the study characteristics

The result with regard to measurement properties can only be generalized to populations that are similar to the study sample in which the measurement properties have been evaluated. This implies that when a measurement property has been evaluated in different studies we need to consider the (dis)similarities in populations and settings in the various studies, and use this to inform whether it is reasonable to combine the results from the studies. A further complexity is that we need also to consider the language version of the instrument that is used, and the form of administration (for example, online versus paper based).

There are two options for data synthesis of each measurement property: meta-analysis or narrative synthesis.

Meta-analysis

Statistical methods exist for pooling parameters related to measurement property data, for example, Cronbach's alpha coefficient, correlation coefficients (intra-class, Spearman, Pearson), standard error of measurement (SEMs) and minimal important change (MIC) values. Correlations may be pooled using the correlation coefficients directly or using z-transformed coefficients (Shadish & Haddock, 1994). Pooling should only be performed if there are several studies available that are sufficiently similar to be able to combine their results.

Some heterogeneity between the study estimates should be expected due to differences in participants and study characteristics. Thus, a DerSimonian and Laird random effects model should be used in the meta-analysis (DerSimonian & Laird, 1986). Heterogeneity between the studies should be quantified using the I^2 statistic, and reasons for heterogeneity should be explored using subgroup and/or sensitivity analyses. In particular, sensitivity analyses excluding studies of poor methodological quality should be performed to assess whether the pooled estimates are strongly influenced by the results of these studies.

While meta-analysis of data is encouraged where appropriate, useful published examples of meta-analysis using measurement property data are limited and there is a lack of standardized statistical methods. More research is needed on the methodology of statistical pooling of the data from studies on measurement properties. Some example systematic reviews with meta-analysis that may be worth consulting include Anderson et al. (2019) (correlation coefficients for internal consistency, reliability, construct validity, criterion validity), Bai et al. (2018) (Cronbach's alpha for internal consistency, ICC for test-retest reliability, Pearson correlation for hypotheses testing), Chamorro et al. (2017) (LoA for reliability, ICC for criterion validity), Chiarotto et al. (2016) (correlation coefficients for construct validity), and Collins et al. (2016) (standardized response mean (SRM) for responsiveness).

Narrative synthesis

Measurement property data that is not suitable to pool in meta-analysis should be combined using narrative synthesis. The narrative synthesis should take into consideration the following characteristics when reporting the findings of the studies: the methodological quality of the studies, consistency of the results, and homogeneity of the studies.

Evaluation of the measurement instrument(s)

Once the data has been statistically pooled or narratively synthesized, the evidence for each measurement property for each instrument of interest should be compared to accepted criteria for adequate measurement properties. It is recommended to use the 'criteria for good measurement properties' suggested by COSMIN (Prinsen et al. (2018) – Table 1; Mokkink et al. (2018b) – Table 4). Using these criteria, each measurement property can be rated as either sufficient, insufficient, or indeterminate. This overall rating is important in determining whether a measurement instrument is adequate for use for particular populations and contexts.

For peer review only

12.2.11 Assessing certainty in the findings (i.e. GRADE approach)

The Grading of Recommendations Assessment, Development, and Evaluation (GRADE) approach should be used to grade the quality of the pooled or summarized evidence (Schünemann et al., 2013). The starting point for evidence of measurement property data is assumed to be high, with the quality of evidence subsequently downgraded due to risk of bias, inconsistency, imprecision, and indirectness. Work in this area is evolving, however COSMIN currently recommend to not consider publication bias in the GRADE assessment for measurement property data, as this is difficult to assess given the lack of registries for these types of studies (Mokkink et al., 2018b; Prinsen et al., 2018). Further details on how to apply GRADE in systematic reviews of measurement properties can be found in the COSMIN user manual (Mokkink et al., 2018b).

For peer review only

12.3 The systematic review

This section provides further guidance on components that should be included in the final report of a systematic review of measurement properties, and information that each component should contain. This section also provides a brief outline of the format and stylistic conventions for systematic reviews to ensure they meet the formatting criteria for the *JBI Evidence Synthesis*. For further information please refer to the Author Guidelines of the journal.

All JBI systematic reviews of measurement properties should be based on a protocol that is publicly accessible (e.g. published or accepted for publication in the *JBI Evidence Synthesis* and/or registered in PROSPERO). Where deviations from a published protocol occur, these must be clearly detailed and justified in the methods section of the systematic review.

For peer review only

12.3.1 Title

The title should be clear and explicit, and reflect the core elements of the review. As per the advice for the protocol, the title should state that it is “a systematic review of measurement properties”. Titles should not be phrased as questions or conclusions and there should be congruency between the title, review objectives/questions and inclusion criteria.

For peer review only

12.3.2 Abstract

The structured abstract must accurately reflect and summarize the main features of the systematic review for the reader, in particular the results of the review. The abstract must be 500 words or less and should not contain abbreviations (unless they are universally understood, e.g. IVF) or references. The following sub-headings should be used to structure the abstract: Objective, Introduction, Inclusion Criteria, Methods, Results, and Conclusions. Further details regarding the extent of information to include under these headings is provided below.

Objective

State the overarching review objective in full, as described in the protocol section.

Introduction

Describe very briefly the issue under review and what this review will add to the evidence base

Inclusion criteria

Summarize the inclusion criteria in one or two sentences, including details of Population (and setting), Instruments, Construct, and Outcomes (measurement properties of interest).

Methods

List the key sources searched, relevant limits placed on the scope of the search, and the month and year of the search. Briefly describe the approach to critical appraisal, study selection, data extraction and data synthesis.

Results

This should be the principal focus of the Abstract. Provide important details of the results, including the number of studies located and included, the overall methodological quality and, most importantly, the key findings.

Conclusions

Articulate brief overall conclusions based on the systematic review findings, including a clear answer to the question(s) of the review.

For peer review only

12.3.3 Keywords

List a maximum of five keywords in alphabetical order, separated by a semi-colon and a space. Note: these are for the purposes of meta-data and indexing, and not related to the search strategy.

For peer review only

12.3.4 Summary of findings

A Summary of Findings table should be presented following the abstract. Summary of findings should be reported for each measurement property for each instrument. The summary of findings may be set out as per the example template below. The table should report, for each measurement property for each instrument, the total number of participants and studies that have contributed data and the pooled result or summary of results. Furthermore, the overall rating for each measurement property for each instrument should be presented, according to the 'criteria for good measurement properties' suggested by COSMIN, i.e. sufficient, insufficient, or indeterminate (Prinsen et al. (2018) – Table 1; Mokkink et al. (2018b) – Table 4). The quality of the evidence is to be reported, assessed according to the GRADE approach (see section 12.2.11).

Summary of Findings

Measurement property/instrument	Number of participants (studies)	Pooled result or summary	Overall rating	Quality of the evidence (GRADE)
**Internal consistency**				
Instrument A				
Instrument B				
**Reliability**				
Instrument A				
Instrument B				
**Measurement error**				
Instrument A				
Instrument B				
**Content validity**				
Instrument A				
Instrument B				
**Structural validity**				
Instrument A				
Instrument B				
**Hypotheses testing**				
Instrument A				
Instrument B				
**Cross-cultural validity**				
Instrument A				
Instrument B				
**Criterion validity**				
Instrument A				
Instrument B				
**Responsiveness**				
Instrument A				
Instrument B				

For peer review only

12.3.5 Review question(s)

As discussed previously in the protocol section, the question(s) of the review should be clearly stated. The review question(s) should be the same as stated in the protocol.

For peer review only

12.3.6 Introduction

The introduction section of the systematic review should be comprehensive and cover all the main elements of the topic under review. The introduction section prepared for the protocol generally makes a good starting point; however, it should not duplicate the introduction in the protocol and will need extension or modification following the review.

The introduction should conclude with the overarching review objective that aligns with the core elements/mnemonic of the inclusion criteria, as it situates the justification and importance of the question(s) posed. The Vancouver style referencing should be used throughout the review with superscript numbers without brackets used for in-text citations.

For peer review only

12.3.7 Inclusion criteria

As detailed in the protocol, the inclusion criteria used to determine consideration for inclusion should be stated. The inclusion criteria should be as clear and unambiguous as possible. As for the protocol, the inclusion criteria should be presented under the headings:

- Population
- Instrument(s)
- Construct
- Outcomes
- Types of studies

For peer review only

12.3.8 Methods

This section of the systematic review is reserved for the methods used to conduct the review and should be presented under the relevant subheadings, including any deviations from the method outlined in the *a priori* protocol.

Directly below the Methods heading provide the following information:

- State and appropriately cite the JBI methodology that was employed in the conduct of the review and synthesis.

Refer to and cite the *a priori* protocol that was published, or has been accepted for publication (e.g. 'in press') in the *JBI Evidence Synthesis* and/or if the protocol has been registered with PROSPERO, provide registration information including registration number (e.g. PROSPERO CRD42015425226).

12.3.8.1 Search strategy

This section should detail how the reviewers searched for relevant papers. The information sources that were searched must be listed along with the search dates. A detailed search strategy for all major databases searched must be appended to the review. The documentation of search strategies is a key element of the scientific validity of a systematic review. It enables readers to examine and evaluate the steps taken, decisions made to consider the comprehensiveness and exhaustiveness of the search strategy for each included database. A JBI review should consider papers published in both commercial (e.g. PubMed, CINAHL, EMBASE) and in non-commercially operated databases (grey/gray literature).

Each electronic database is likely to use a different system for indexing key words within their search engines. Hence, the search strategy will be tailored to each particular database. These variations are important and need to be captured and included in the systematic review.

12.3.8.2 Study selection

The review should describe the actual process of study screening and all the stages of selection (based on title and abstract examination; on full text examination etc.) and the actual procedures used for solving disagreements between reviewers.

12.3.8.3 Assessment of methodological quality

This section should detail the approach to critical appraisal, not the assessment results, and should be consistent with the protocol. Any deviations from the protocol must be reported and explained. The report should detail the criteria that were used when determining the methodological quality of papers considered for inclusion in the review. If the COSMIN Risk of Bias checklist (Mokkink et al., 2018a) was used as recommended, this should be appropriately cited. It is not necessary to append the COSMIN checklist, but the reference should be provided in a footnote to the table of methodological quality results.

12.3.8.4 Data extraction

This section of the review should include details of the types of data extracted from the included studies. If no data was available for particular measurement properties, that should also be reported. Standardized data extraction tools allow the extraction of the same types of data across the included studies and are recommended for JBI systematic reviews. Information that may impact upon the generalizability of the review findings such as study methods, setting and population characteristics etc. should also be extracted. This information is reported in the characteristics of included studies table (Appendix 12.1) and an overall description of key characteristics reported in the description of included studies section (see 12.3.9.3). Population characteristics include factors such as age, past medical history, co-morbidities, complications or other potential confounders. JBI aims to reduce errors in data extraction by using two independent reviewers. The data extraction tool used must be cited, with this Chapter cited as the reference. Authors should only append the data extraction tool if the cited tool was modified or a new tool developed. Any modifications to existing tools should be described in the text.

12.3.8.5 Data synthesis

The data synthesized within a systematic review are the results extracted from research studies relevant to the review question. This section should report if meta-analysis was conducted and, if so, the methods that were utilized. Indices that were unable to be pooled in meta-analysis, should be combined in narrative synthesis, making use of tables to aid in data presentation. The overall rating for each measurement property for each instrument of interest should be reported, according to established criteria for adequate measurement properties.

For peer review only

12.3.9 Results

12.3.9.1 Study inclusion

This section should provide a narrative summary of the search and selection process results. The number of papers identified by the search strategy and the number of papers that were included and excluded at each stage of the study selection process should be reported.

A complete and accurate report should be provided regarding:

- The number of studies identified by the search in diverse sources;
- the number of studies excluded after the examination of title and abstract against inclusion criteria;
- the number of full text articles retrieved for examination;
- the number of studies excluded after full text examination against inclusion criteria;
- the number of critically appraised studies;
- the number of studies excluded after critical appraisal; and
- the total number of included studies.

A figure using the PRISMA flowchart for the reporting of the selection process should be included (Moher et al., 2009). A list of all excluded studies, (i. e. those excluded on full text examination and after critical appraisal), with the explicit reasons for exclusion, should be provided in appendices to the review and referred to in the text.

12.3.9.2 Methodological quality

This section should include the results from the assessment of methodological quality/critical appraisal. It is recommended to use the COSMIN Risk of Bias checklist (Mokkink et al., 2018a). Criteria should be assessed and results presented according to the COSMIN guidance (Mokkink et al., 2018a; Mokkink et al., 2018b; Terwee et al., 2018).

12.3.9.3 Description of included studies

This section of the results should include an overall description of the included studies. The main aim is to provide context to the results section and sufficient descriptive detail for the reader to support the inclusion of the studies in the review, the relevance of included studies to the review question, and the evidence base they offer to the question. A characteristics of included studies table should be appended to the review that has been populated from the appropriate extraction fields from the extraction tool (see Appendix 12.1 for an example characteristics of included studies template).

12.3.9.4 Review findings

The findings of the review and presentation of the results should flow logically from the review objective/question(s). Given there is no clear international standard or agreement on the structure or key components of this section of a review, and the level of variation evident in published systematic reviews, the advice here is general in nature. Typically, findings are discussed textually and then supported with meta-analysis, tables, or figures as appropriate.

The focus should be on presenting information in a clear and concise manner. Any large or complex diagrams/tables/figures should be included as appendices so as not to break the flow of text. Meta-view graphs represent a specific item of analysis that can be incorporated in the results section of the review. However, the results are more than meta-view graphs, and whether this section is structured based on the measurement properties or instruments of interest, or some other structure, the content of this section needs to present the results with clarity.

While there is no standard format for this section, it is recommended to utilize one or more tables to organize the results (see Appendix 2 for an example template). If the number of studies permit, then separate tables of results can be used which relate to i) reliability; ii) validity, and iii) responsiveness (if applicable). Depending on the number of instruments evaluated, separate tables may be presented for each instrument. It is important that if at least one measurement property for each instrument under consideration is not reported within the studies included in the review, then this is reported within the text and the table of results.

For peer review only - http://bmjopen.bmj.com/site/about/guidelines.xhtml

12.3.10 Discussion

The aim of this section is to summarize and discuss (not repeat in detail) the main findings, including the strength of the evidence for each measurement property/instrument. This section should discuss any limitations of the primary studies included in the review and of the review itself (i.e. language restriction, access, timeframe, study design, etc.). The results should be discussed in the context of current literature, practice and policy.

The discussion should not bring in new findings that have not been reported in the results section. It should seek to establish a line of argument based on the findings regarding the suitability of particular measurement instruments for measuring a desired construct in a specific population and setting. The application and relevance of the findings to relevant stakeholders (e.g. healthcare providers, patients and policy makers) should also be discussed in this section.

Points to consider for the discussion include:

- Were any problems identified undertaking the search (perhaps there is little primary research on this topic or perhaps it is poorly indexed by the databases that were searched or perhaps the search was insufficient)?
- What limitations were found in the included primary research (e.g. were there inconsistencies or errors in reporting)?
- How do the review findings fit with what is currently known on the topic (from issues highlighted in the Introduction section)?

Are the findings generalizable to other populations of participants/healthcare settings etc.?

For peer review only

12.3.11 Conclusions

This section should include the overall conclusions of the review. The conclusions should provide direct answers to the review questions. These conclusions should be based only on the results of the review and directly inferred from the review results.

Recommendations for practice

This sub-section of the Conclusions section should include the recommendations for practice inferred from the results of the review and based on the discussion of the generalizability of the results and the potential factors that may affect the applicability of the results. It should be stated how the findings of the review impact on clinical practice or policy in the area. Where there is sufficient evidence to make specific recommendations for practice, these should be clearly articulated. Recommendations should be assigned a JBI Grade of Recommendation (Munn, 2015).

Recommendations for research

This sub-section should include clear, specific recommendations for future research based on gaps in knowledge identified from the results of the review. Recommendations for research should avoid generalized statements calling for further research, but should be linked to specific issues. Recommendations for research may include the development of new measurement instruments (if existing instruments are found to be inadequate) or further rigorous validation studies conducted in specific populations/contexts.

For peer review only

12.4 Glossary of terms

Term	Definition
Area under the curve (AUC)	In a receiver operating characteristic (ROC) curve analysis, an index of the performance of a diagnostic or screening measure in relation to diagnostic accuracy, summarized in a single value that typically ranges from 0.50 (no better than random classification) to 1.0 (perfect classification) (Polit & Yang, 2016); a measure of criterion validity or responsiveness.
Ceiling effect	The effect of having scores restricted at the upper end of a score continuum which limits discrimination at the upper end of the measurement, constrains true variability and restricts the amount of upward change possible (Polit & Yang, 2016); a measure of content validity.
Clinimetrics	The study of instruments where items may be major or minor; or present or absent (Gewitz et al., 2015).
Comparative fit index (CFI)	A statistic used to evaluate the goodness of fit of a proposed model to the data (e.g. in a confirmatory factor analysis or item response theory analysis) involving the comparison of the proposed model with a null model; a value greater than 0.95 is often considered as indicative of good fit (Polit & Yang, 2016); a measure of construct validity.
Construct validity	The degree to which evidence about a measure's scores in relation to other scores supports the inference that a construct has been appropriately represented; the degree to which a measure captures the focal construct (Polit & Yang, 2016).
Content validity	The degree to which a multi-item instrument has an appropriate set of relevant items reflecting the full content of the construct domain being measured (Polit & Yang, 2016); incorporates face validity.
Content validity index (CVI)	An index summarizing the degree to which a panel of experts agrees on an instrument's content validity (i.e. the relevance, comprehensiveness and balance of items comprising a scale) (Polit & Yang, 2016). There are item-level and scale-level CVI.
Criterion validity	The extent to which scores on a measure are an adequate reflection of (or predictor of) a criterion (i.e. 'gold standard' measure) (Polit & Yang, 2016).
Cronbach's alpha coefficients (Coefficient alpha)	An index of internal consistency that indicates the degree to which the items on a multi-item scale are measuring the same underlying construct (Polit & Yang, 2016); a measure of reliability.
Cross cultural validity	The degree to which the items on a translated or culturally adapted scale perform adequately and equivalently, individually and in the aggregate, in relation to their performance on the original instrument; an aspect of construct validity (Polit & Yang, 2016).
Differential item functioning (DIF)	The extent to which an item functions differently for one group or culture than for another despite the groups being equivalent with respect to the underlying latent trait (Polit & Yang, 2016); a measure of cross-cultural validity.
Face validity	The extent to which an instrument looks as though it is a measure of the target construct (Polit & Yang, 2016). An aspect of content validity.
Factor analysis	A statistical procedure for disentangling complex interrelationships among items and identifying the items that 'go together' as a unified dimension; A measure of construct validity (Polit & Yang, 2016).
Floor effect	The effect of having scores restricted at the lower end of a score continuum which limits the ability of the measure to discriminate at the lower end of the measurement, constrains true variability and limits the amount of downward change possible (Polit & Yang, 2016); a measure of content validity.
Goodness of fit index (GFI)	A statistic used to evaluate the goodness of fit of a proposed model to the data (e.g. In confirmatory factor analysis); a value greater than .90 is often considered as an adequate fit (Polit & Yang, 2016); a measure of reliability.
Internal consistency	The degree to which the subparts of a composite scale (i.e. the items) are interrelated and are all measuring the same attribute or dimension; a measure of reliability (Polit & Yang, 2016).
Inter-rater reliability	The variation between two or more raters who measure the same group of subjects.
Intra-class correlation coefficients (ICC)	Estimates the proportion of total variance in a set of scores that is attributable to true differences among the people or objects being measured (e.g. the test-retest reliability); a measure of reliability (Polit & Yang, 2016).
Intra-rater reliability	The variation of data measured by a single rater across two or more occasions.
Kappa	A statistical index of chance-corrected agreement or consistency between two nominal (or ordinal) measurements; often used to assess interrater or intra-rater reliability (Polit & Yang, 2016).
Limits of agreement (LOA)	An estimate of the range of differences in two sets of scores that could be considered random measurement error, typically with 95% confidence; graphically portrayed on Bland-Altman plots (Polit & Yang, 2016); a measure of reliability.

For peer review only

Measurement error	The systematic and random error of a person's score on a measure , reflecting factors other that the construct being measured and resulting in an observed score that is different from a hypothetical true score; a measurement property within the reliability domain (Polit & Yang, 2016).
Measurement properties	Instruments that incorporate psychometric or clinimetric characteristics.
Non-normed fit index (NNFI)	Also known as Tucker-Lewis index (TLI)-see below.
Psychometrics	The study of instruments that consist of items of equal weighting.
Reliability	The degree to which a measurement is free from measurement error; the extent to which scores for people who have not changed are the same for repeated measurements; statistically, the proportion of total variance in a set of scores that is attributable to true differences among those being measured (Polit & Yang, 2016).
Responsiveness	The ability of a measure to detect change over time in a construct that has changed, commensurate with the amount of change that has occurred (Polit & Yang, 2016).
Root mean square error of approximation (RMSEA)	An index used to evaluate how well a hypothesized model fits the data (e.g. in confirmatory factor analysis or item response theory modelling); an RMSEA of less than .06 is considered an indicator of adequate fit (Polit & Yang, 2016); a measure of construct validity.
Sensitivity	The ability of a screening or diagnostic instrument to correctly identify a 'case' (i.e. to correctly diagnose a condition) (Polit & Yang, 2016); a measure of criterion validity or responsiveness.
Smallest detectable change (SDC)	An index that estimates the threshold for a 'real' change in scores (i.e. a change that, with 95% confidence, is beyond measurement error); the SDC is a change score that falls outside the limits of agreement on a Bland-Altman plot (Polit & Yang, 2016); a measure of reliability.
Specificity	The ability of a screening or diagnostic instrument to correctly identify non-cases for a condition (Polit & Yang, 2016); a measure of criterion validity or responsiveness.
Standard error of measurement (SEM)	An index that quantifies the amount of 'typical' error on a measure and indicates the precision of individual scores (Polit & Yang, 2016); a measure of reliability.
Standardized root mean square residual (SRMR)	An index used to evaluate how well a hypothesized model fits the data (e.g. In a confirmatory factor analysis); an SRMR of less than 0.08 is considered an indicator of adequate fit (Polit & Yang, 2016); a measure of construct validity.
Structural validity	The extent to which an instrument captures the hypothesized dimensionality of the broad construct; an aspect of construct validity (Polit & Yang, 2016).
Test-retest reliability	The variation in measurements using an instrument on the same subject under the same conditions.
Tucker-Lewis index (TLI)	Also known as non-normed fit index (NNFI). A statistic used to evaluate the goodness of fit of a proposed model to the data (e.g. In confirmatory factor analysis) involving the comparison of the proposed model with a null model; a value greater than 0.95 is often considered as indicative of a good fit (Polit & Yang, 2016); a measure of construct validity.
Validity	In a measurement context, the degree to which an instrument is measuring the construct it purports to measure (Polit & Yang, 2016).

For peer review only

12.5 Chapter references

Anderson, D. B., Mathieson, S., Eyles, J., Maher, C. G., Van Gelder, J. M., Tomkins-Lane, C. C., . . . Ferreira, M. L. (2019). Measurement properties of walking outcome measures for neurogenic claudication: a systematic review and meta analysis. *Spine J*. doi: 10.1016/j.spinee.2019.04.004

Aromataris, E., & Pearson, A. (2014). The systematic review: an overview. *Am J Nurs, 114*(3), 53-58. doi: 10.1097/01.NAJ.0000444496.24228.2c

Bai, Z., Shu, T., Lu, J., & Niu, W. (2018). Measurement Properties of the Functional Rating Index: A Systematic Review and Meta-analysis. *Spine (Phila Pa 1976), 43*(22), E1340-E1349. doi: 10.1097/brs.0000000000002690

Chamorro, C., Armijo-Olivo, S., De la Fuente, C., Fuentes, J., & Javier Chirosa, L. (2017). Absolute Reliability and Concurrent Validity of Hand Held Dynamometry and Isokinetic Dynamometry in the Hip, Knee and Ankle Joint: Systematic Review and Meta-analysis. *Open Med (Wars), 12*, 359-375. doi: 10.1515/med-2017-0052

Chiarotto, A., Maxwell, L. J., Terwee, C. B., Wells, G. A., Tugwell, P., & Ostelo, R. W. (2016). Roland-Morris Disability Questionnaire and Oswestry Disability Index: Which Has Better Measurement Properties for Measuring Physical Functioning in Nonspecific Low Back Pain? Systematic Review and Meta-Analysis. *Phys Ther, 96*(10), 1620-1637. doi: 10.2522/ptj.20150420

Collins, N. J., Prinsen, C. A., Christensen, R., Bartels, E. M., Terwee, C. B., & Roos, E. M. (2016). Knee Injury and Osteoarthritis Outcome Score (KOOS): systematic review and meta-analysis of measurement properties. *Osteoarthritis Cartilage, 24*(8), 1317-1329. doi: 10.1016/j.joca.2016.03.010

DerSimonian, R., & Laird, N. (1986). Meta-analysis in clinical trials. *Control Clin Trials, 7*(3), 177-188.

Francis, O. D., McPheeters, M. L., Noud, M., Penson, D. F., & Feurer, I. D. (2016). Checklist to operationalize measurement characteristics of patient-reported outcome measures. *BMC Systematic Reviews, 5*(129), 1-11. doi: 10.1186/s13643-016-0307-4

Gewitz, M. H., Baltimore, R. S., Tani, L. Y., Sable, C. A., Shulman, S. T., Carapetis, J., . . . Kaplan, E. L. (2015). Revision of the Jones Criteria for the Diagnosis of Acute Rheumatic Fever in the Era of Doppler Echocardiography. *Circulation, 131*(20), 1806-1818. doi: org/10.1161/CIR.0000000000000205

Ginty, A. T. (2013). Psychometric Properties. In M. D. Gellman & J. R. Turner (Eds.), *Encyclopedia of Behavioral Medicine* (pp. 1563-1564). New York, NY: Springer New York.

Lynn, M. R. (1986). Determination and quantification of content validity. *Nurs Res, 35*(6), 382-385.

Moher, D., Liberati, A., Tetzlaff, J., & Altman, D. (2009). The PRISMA Group Preferred Reporting Items for Systematic Reviews and Meta-Analyses: The PRISMA Statement. *PLoS Med, 6*(7), 1-6. Retrieved from doi:10.1371/journal.pmed1000097

Mokkink, L. B., de Vet, H. C. W., Prinsen, C. A. C., Patrick, D. L., Alonso, J., Bouter, L. M., & Terwee, C. B. (2018a). COSMIN Risk of Bias checklist for systematic reviews of Patient-Reported Outcome Measures. *Qual Life Res, 27*(5), 1171-1179. doi: 10.1007/s11136-017-1765-4

Mokkink, L. B., Prinsen, C. A. C., Patrick, D. L., Alonso, J., Bouter, L. M., de Vet, H. C. W., & Terwee, C. B. (2018b). *COSMIN methodology for systematic reviews of Patient-Reported Outcome Measures (PROMs)*. Retrieved from www.cosmin.nl

Mokkink, L. B., Terwee, C. B., Patrick, D. L., Alonso, J., Stratford, P. W., Knol, D. L., . . . de Vet, H. C. (2010a). The COSMIN study reached international consensus on taxonomy, terminology, and definitions of measurement properties for health-related patient-reported outcomes. *J Clin Epidemiol, 63*(7), 737-745. doi: 10.1016/j.jclinepi.2010.02.006

Mokkink, L. B., Terwee, C. B., Patrick, D. L., Alonso, J., Stratford, P. W., Knol, D. L., . . . de Vet, H. C. W. (2010b). The COSMIN checklist for assessing the methodological quality of studies on measurement properties of health status measurement instruments: an international Delphi study. *Q uality of Life Research, 19*(4), 539-549. doi: 10.1007/s11136-010-9606-8

Mokkink, L. B., Terwee, C. B., Prinsen, C. A. C., & de Vet, H. C. W. (2016). Taxonomy of measurement properties: A commentary on Polit (2015). *Inter national Journal of Nursing Studies, 53*, 399-400. doi: http://dx.doi.org/10.1016/j.ijnurstu.2015.08.010

Mokkink, L. B., Terwee, C. B., Stratford, P. W., Alonso, J., Patrick, D. L., Riphagen, I., . . . de Vet, H. C. (2009). Evaluation of the methodological quality of systematic reviews of health status measurement instruments. *Quality of Life Research, 18*(3), 313-333. doi: 10.1007/s11136-009-9451-9

Munn, Z. (2015). Implications for Practice: should recommendations be recommended in systematic reviews? *JBI Database of Systematic Reviews and Implementation Reports, 13*(7), 1-3.

Polit, D. F. (2015). Assessing measurement in health: Beyond reliability and validity. *International Journal of Nursing Studies, 52*(11), 1746-1753. doi: http://dx.doi.org/10.1016/j.ijnurstu.2015.07.002

Polit, D. F. (2016). Taxonomy of measurement properties: A response to the commentary by Mokkink et al. (2015). *International Journal of Nursing Studies, 53*, 401-402. doi: http://dx.doi.org/10.1016/j.ijnurstu.2015.10.009

Polit, D. F., & Beck, C. (2014). *Essentials of nursing research: Appraising evidence for nursing practice* (8 ed.). Philadelphia: Wolters Kluwer Health | Lippincott Williams & Wilkins.

Polit, D. F., & Yang, F. (2016). *Measurement and the measurement of change: A primer for health professionals*. Philadelphia: Lippincott Williams and Wilkins.

Porritt, K., Gomersall, J., & Lockwood, C. (2014). JBI's Systematic Reviews: Study selection and critical appraisal. *Am J Nurs, 114*(6), 47-52. doi: 10.1097/01.Naj.0000450430.97383.64

Prinsen, C. A. C., Mokkink, L. B., Bouter, L. M., Alonso, J., Patrick, D. L., de Vet, H. C. W., & Terwee, C. B. (2018). COSMIN guideline for systematic reviews of patient-reported outcome measures. *Qual Life Res, 27*(5), 1147-1157. doi: 10.1007/s11136-018-1798-3

For peer review only

Rosenkoetter, U., & Tate, R. L. (2017). Assessing Features of Psychometric Assessment Instruments: A Comparison of the COSMIN Checklist with Other Critical Appraisal Tools. *Brain Impairment, 19*(1), 103-118. doi: 10.1017/BrImp.2017.29

Schünemann, H., Broek, J., Guyatt, G., & Oxman, A., (editors). (2013). GRADE handbook for grading quality of evidence and strength of recommendations. Updated October 2013.

Shadish, W. R., & Haddock, C. K. (1994). Combining estimates of effect size *The handbook of research synthesis.* (pp. 261-281). New York, NY, US: Russell Sage Foundation.

Streiner, D. L. (2003). Clinimetrics vs. psychometrics: an unnecessary distinction. *J Clin Epidemiol, 56*(12), 1142-1145; discussion 1146-1149.

Terwee, C., Prinsen, C., Garotti, M. R., Suman, A., De Vet, H., & Mokkink, L. (2016a). The quality of systematic reviews of health-related outcome measurement instruments. *Quality of Life Research, 25*(4), 767-779.

Terwee, C. B., de Vet, H. C. W., Prinsen, C. A. C., & Mokkink, L. B. (2016b). Comment on “Checklist to operationalize measurement characteristics of patient-reported outcome measures”.   Retrieved 16 Apr, 2017, from http://www.cosmin.nl/images/upload/files/Letter%20Francis.pdf

Terwee, C. B., Jansma, E. P., Riphagen, I. I., & de Vet, H. C. (2009). Development of a methodological PubMed search filter for finding studies on measurement properties of measurement instruments. *Quality of Life Research, 18*(8), 1115-1123.

Terwee, C. B., Mokkink, L. B., Knol, D. L., Ostelo, R. W., Bouter, L. M., & de Vet, H. C. (2012). Rating the methodological quality in systematic reviews of studies on measurement properties: a scoring system for the COSMIN checklist. *Quality of Life Research, 21*(4), 651-657. doi: 10.1007/s11136-011-9960-1

Terwee, C. B., Prinsen, C. A. C., Chiarotto, A., Westerman, M. J., Patrick, D. L., Alonso, J., . . . Mokkink, L. B. (2018). COSMIN methodology for evaluating the content validity of patient-reported outcome measures: a Delphi study. *Qual Life Res, 27*(5), 1159-1170. doi: 10.1007/s11136-018-1829-0

University of Oxford. Patient-Reported Outcomes Measurement Group.   Retrieved Accessed 15 October 2018, from http://phi.uhce.ox.ac.uk/home.php

Whalen, K., & Buchholz, S. W. (2009). The reliability, validity and feasibility of tools used to screen for caregiver burden: a systematic review. *JBI Library of Systematic Reviews, 7*(32), 1373-1430.

For peer review only

Appendix 12.1 Data extraction / characteristics of included studies template

Study	Instrument(s) assessed	Construct assessed	Country/ language	Mode of admin	Setting/ Context	Participants	Results (measurement properties)	Comments

For peer review only

Appendix 12.2 Table of results template

Study	Instrument(s) assessed	Reliability			Validity					Responsiveness
		Internal consistency	Reliability	Measurement error	Content validity (face validity)	Construct validity			Criterion validity	
						Structural validity	Hypotheses testing	Cross-cultural validity		
Author et al.[1]	Instrument A									
	Instrument B									
Author et al.[2]	Instrument A									
	Instrument B									

For peer review only

Archived at the Flinders Academic Commons:
http://dspace.flinders.edu.au/dspace/

The **AACODS** checklist is designed to enable evaluation and critical appraisal of grey literature.

The Fourth International Conference on Grey Literature held in Washington, DC, in October 1999 **defined grey literature** as: "that which is produced on all levels of government, academics, business and industry in print and electronic formats, but which is not controlled by commercial publishers."
Grey literature includes theses or dissertations (reviewed by examiners who are subject specialists); conference papers (often peer-reviewed or presented by those with specialist knowledge) and various types of reports from those working in the field. All of these fall into the **"expert opinion"**
Critical appraisal is "the process of carefully and systematically examining research to judge its trustworthiness, and its relevance and value in a particular context" (Burls 2009)

Grey (unpublished) studies and RCTs should be appraised using the same tools as their black (published) counterparts.

**AACODS**		**YES**	**NO**	**?**
**Authority**	Identifying who is responsible for the intellectual content.<br><br>**Individual author:**<br>• Associated with a reputable organisation?<br>• Professional qualifications or considerable experience?<br>• Produced/published other work (grey/black) in the field?<br>• Recognised expert, identified in other sources?<br>• Cited by others? (use Google Scholar as a quick check)<br>• Higher degree student under "expert" supervision?<br><br>**Organisation or group**:<br>• Is the organisation reputable? (e.g. W.H.O)<br>• Is the organisation an authority in the field?<br><br>**In all cases:**<br>• Does the item have a detailed reference list or bibliography?			
**Accuracy**	• Does the item have a clearly stated aim or brief?<br>• Is so, is this met?<br>• Does it have a stated methodology?<br>• If so, is it adhered to?<br>• Has it been peer-reviewed?<br>• Has it been edited by a reputable authority?<br>• Supported by authoritative, documented references or credible sources?<br>• Is it representative of work in the field?<br>• If No, is it a valid counterbalance?<br>• Is any data collection explicit and appropriate for the research?<br>• If item is secondary material (e.g. a policy brief of a technical report) refer to<br>• the original. Is it an accurate, unbiased interpretation or analysis?			

For peer review only

Archived at the Flinders Academic Commons:
http://dspace.flinders.edu.au/dspace/

**Coverage**	All items have parameters which define their content coverage. These limits might mean that a work refers to a particular population group, or that it excluded certain types of publication. A report could be designed to answer a particular question, or be based on statistics from a particular survey.<br><br>• Are any limits clearly stated?			
**Objectivity**	It is important to identify bias, particularly if it is unstated or unacknowledged.<br><br>• Opinion, expert or otherwise, is still opinion: is the author's standpoint clear?<br>• Does the work seem to be balanced in presentation?			
**Date**	For the item to inform your research, it needs to have a date that confirms relevance<br><br>• Does the item have a clearly stated date related to content? No easily discernible date is a strong concern.<br>• If no date is given, but can be closely ascertained, is there a valid reason for its absence?<br>• Check the bibliography: have key contemporary material been included?			
**Significance**	This is a value judgment of the item, in the context of the relevant research area<br><br>• Is the item meaningful? (this incorporates feasibility, utility and relevance)<br>• Does it add context?<br>• Does it enrich or add something unique to the research?<br>• Does it strengthen or refute a current position?<br>• Would the research area be lesser without it?<br>• Is it integral, representative, typical?<br>• Does it have impact? (in the sense of influencing the work or behaviour of others)			

For peer review only

Burls, A. 2009, *What is critical appraisal*?, Bandolier, viewed 4 November 2009.
<http://www.medicine.ox.ac.uk/bandolier/painres/download/whatis/What_is_critical_appraisal.pdf>

Jess Tyndall,
Flinders University,
Nov 2010

What does social cognition look like in everyday social functioning in Huntington's disease? - A protocol for a scoping review to explore and synthesise knowledge about social cognition alongside day-to-day social functioning of people with Huntington's disease

Corresponding Author:

Alexandra Fisher, email: alf944@bham.ac.uk

Authors

Alexandra Fisher [1,2,3], Anna Lavis [1], Sheila Greenfield [1], Hugh Rickards [1,2,3]

Keywords

Social cognition, social interaction, Huntington's disease, synthesis, literature

Abstract

Introduction

Social cognition is problematic in Huntington's disease (HD). Despite the observations of clinicians and families, there is minimal empirical literature about how it presents in daily life and the impact on social functioning. This protocol forms the basis of a scoping review to synthesize both the quantitative knowledge and qualitative experiences of the HD community so that a visual and narrative map can address what is known and what is not known for the benefit of the community and clinicians and academics alike.

Methods and analyses

An umbrella scoping review of previous work and a scoping review of newer studies of social cognition and social functioning will be undertaken. The electronic databases PubMed, Medline, Psych info, Web of Science, Scopus, Embase and CINAHL will be searched to identify eligible studies from starting from 2003 to March 2023. A grey literature search and grey data search will also be undertaken. Quality appraisal of the included documents will use the Critical Appraisal Skills Programme and AACODS (Authority, Accuracy, Coverage, Objectivity, Date, Significance) checklists. A data charting table will be used for data extraction, with analysis of qualitative data using the framework method. The review findings will be presented in a visual form and in a narrative summary.

Ethics and dissemination

Ethical review is not usually required as scoping reviews are produced via secondary data analysis, however this protocol includes the use of grey data from a charity web forum and so in line with best practice for internet mediated research ethical review was sought and approved. Review findings will be shared with service users and disseminated through a peer-reviewed publications, conference presentations and hosted via the website of the patient association charity the Huntington's Disease Association (HDA).

Strengths and limitations of this protocol

- This mixed scoping review will amalgamate the Preferred Reporting Items for Systematic Reviews and Meta analyses (PRISMA) extended guidelines for the conduct of scoping reviews as well as the Joanna Briggs Institute (JBI) method for mixed reviews to ensure transparency and rigour.

For peer review only

- Service user and community involvement in the design and conduct of the study which informs this review will ensure that the findings are supported by their experiences, further helping to identify evidence gaps and inform clinical and research practice.
- The use of grey literature and grey data will be searched to ensure that the review reflects the challenges faced by the HD community not currently captured in the scientific literature. A search strategy has been designed to try and reduce the issues associated with the inherent replicability problems by their inclusion.
- Whilst every effort has been made to create a protocol which is inclusive and comprehensive, there are barriers to this defined by the student authors resources i.e. the exclusion of non-English Language papers

Introduction

Most of our everyday behaviour is motivated by social and emotional goals (1) and social cognition is how we process that social information.

Huntington's disease (HD) is an inherited, neurodegenerative disease which impacts communication, cognition, movement, behaviour, and neuropsychiatric function. Increasingly, problems with social cognition and its component skills are now recognised and thought to manifest earlier than many of the latter symptoms (2, 3).

Social cognition in HD

Kennedy & Adolphs wrote a seminal review of neurological and psychiatric disease (4) conceptualising social cognition as the structures and processes underlying social behaviour and it is this definition that underpins the predominant extent of knowledge in HD with studies based on brain scanning but also tests in mostly controlled rather than everyday settings. Highlighting the setting is important because it is in everyday social interaction that the interrelated processes of social cognition and behaviour combine to produce the social skills which are then translated into social functioning which Kennedy & Adolphs define as the embedding of the behaviour over time and in context.

If we take this definition as the lens through which we view social functioning in HD and consider its potential contribution to the quality of various activities of daily living in which social interaction is key, the literature suggests that it is present within crises in intimate and family relationships (5, 6, 7), and work life (8, 9, 10) but may ultimately lead to institutionalization (11). Furthermore, this problematic social functioning has also been observed by family members and clinicians alike (12).

However despite what we know about social cognition and social functioning, the probable relationship between them has not been brought together to form a cohesive picture of what socially related activity looks like in HD. Addressing this disparity would allow for clinicians to draw on this understanding by supporting people with HD and their families through education and tackling these issues collectively, and for researchers to develop more HD specific measures vital to be able to deal with gaps in our knowledge and target therapeutic interventions - all areas which both the HD scientific community (13) and neuroscience community note they need (14). As part of the consultation with the PPI group of the Huntington's Disease Association (HDA) for England and Wales for the ethnographic study (of which this protocol is part), they too reflected it's inherent and much needed value.

Why a scoping review?

The motivations for a scoping review are varied (15) but are usually intended to provide an overview and synthesis of a wide body of literature to clarify concepts and identify gaps steering the audience

For peer review only

towards more specific questions which then might be addressed in a more focused review or which may be used towards a change in practice or policy (16).

The benefit of scoping reviews in HD seems to have recently taken hold through the synthesis of literature to aid the definition of irritability (17) but also the 'lived experience' of the illness (18) following genetic testing and these were driven by the mismatch in the literature and what is seen in clinical practice.

Similarly and with direct relevance to this protocol, Gibson and Springers' (19) motivations came from clinical curiosity and they produced a scoping review looking at reasons for behaviour behind the person's 'decreased participation in social activities in comparison to previous social engagement' (which they termed social withdrawal), what the outcome of this was and what interventions had been tried. Their results noted that the heterogeneity of studies and differences in concepts under the 'umbrella' of social functioning led to the meanings and outcomes of studies becoming blurred and so they made a number of recommendations which will be factored into this review. They were also keen to stress that whilst there are commonalities in social behaviour in HD, future authors should not dismiss the complexities at an individual level which may be fuelling the withdrawal.

Whilst there are already a number of systematic reviews (12, 20) and a meta-analysis (2) on social cognition and its proposed underlying components in HD (3), these now need updating as new knowledge is available. Furthermore, the authors of these reviews recognised that they had not incorporated the associated qualitative and mixed methods on the multiple domains such as work and home life related to social behaviour, interaction, and functioning which represent the extension of social cognition in HD; and likewise, the grey literature and grey data which may also refer to these experiences. It is the intention of this multiple method scoping review to incorporate the potential for rich detail from these latter sources.

What do we mean by grey literature & grey data?

Grey literature is defined as non-commercially published and unpublished literature from organisations in which publishing is not their primary activity and in the case of health sciences this means that it has not been subject to usual peer review (21).

Grey data refers to user generated information which is web based such as online forums.

What is the added value in incorporating grey literature and data in this area?

As Popes et al (22) state, the value of broadening the scope of synthesis and including diverse evidence does not mean abandoning rigour but rather judging the evidence in context and answering specific questions rather than intrinsically defining all evidence as lower quality than others.

Paez (23) also suggested the benefits of including grey literature in a systematic review reduced the potential for publication bias, identified current trends and like Pope et al considered context which Adams et al (24) recommend should now extend to not only 'written' literature such as journals but into the sphere of digital content.

This latter point by Adams is particularly pertinent in the HD area as whilst the study of social cognition has only relatively recently taken traction, the exploration of everyday social behaviour and functioning in HD has been muted with incongruity between might what appear in the (evidence-based) literature, clinical practice and the discussions in online communities. As Coulson and colleagues (25) who looked at the support offered in a HD online community, noted, "participation in an online support group allows a greater degree of anonymity than face-to-face groups. Such

For peer review only - http://bmjopen.bmj.com/site/about/guidelines.xhtml

anonymity may facilitate self-disclosure and help individuals in discussing sensitive issues more easily or to give opinions with less fear of embarrassment or judgement than in more traditional face-to-face groups". It is the contention of the authors of this protocol, in line with Coulson's arguments and our own clinical experiences, that given social functioning in HD maybe fraught with tensions and stigma, investigating this grey data will allow access to stories which illuminate this further.

The challenges of using grey literature and data

Whilst there is much about grey literature and grey data to be positive about, caution and strategies are needed to limit the potential for what is termed as scope creep but also transparency to enable a credible replicability vital for the perceived rigour of the review.

Scope creep is a term often used in industry but also recognised in research (26) when a project is ill defined and therefore in practice, those who advocate for the inclusion of grey literature and grey data recommend a structured and constrained search with boundaries formed by the aims of the review. A tight set of criteria is also needed to shore up the credible replicability referred to earlier. Replicability is a term used by Hoffecker (21) who proffers it as an alternative to that of the usual reproducibility aimed for in a standard systematic review, arguing that due to the nature of grey information (particularly grey data) reproducibility is a concept that is almost impossible to capture due to the evolving nature of its source i.e. an internet forum versus a static record within a database.

The aims of this review are:

1) to explore and summarise the literature related to social cognition in HD,

2) to explore and summarise the literature related to social behaviour and functioning in HD

3) to draw together the findings using both a mixture of both visual and narrative synthesis in line with current best methodological practice (27, 28).

Plan

Method

In Grant and Booth's typography of review methods (29), the authors note that the type of review determines the choice of the review. To fulfil the aims of this review, the following methodological approaches will be used:

1) *to explore and summarise the literature related to social cognition* an umbrella scoping review will be undertaken. As there is already a large amount of systematic work which has reviewed quantitative studies of social cognition in HD, for pragmatic reasons an umbrella style scoping review of these will be presented. An umbrella review is the systematic review method for the combination of results from independent systematic reviews. Therefore, as an umbrella scoping review, unlike a standard umbrella review, the aim will be to produce a synthesis of the data to date rather than a methodological critique of their quality. The guidance for the PRISMA protocol for umbrella reviews and Joanna Briggs Umbrella review methodology will be consulted (30).

Any quantitative papers on social cognition which have been published since the dates of the original systematic reviews will be assessed in the style of the umbrella scoping review. To provide consistency with the quantitative studies in the original systematic reviews (and meta-analysis), a limit for inclusion will be the use of a comparator in their design.

Mixed methods designs on social cognition will not be subject to this latter exclusion. Search terms and key words for the umbrella scoping review will include those from the individual systematic reviews as well as descriptors relevant to the broader domains of social behaviour and functioning.

2) *to explore and summarise the literature related to social behaviour and functioning in HD*, guidance which includes the using PRISMA extended protocol for scoping reviews (31) plus the Joanna Briggs Institute (JBI) method for mixed reviews (32). Search terms and keywords from the umbrella scoping review will be used.

This section of the review will also include both grey literature and grey data. For grey literature its appraisal will refer to the most recent best practice guidelines (33).

For grey data its use and analysis is considered more challenging (34) and at the current time as there are no specific guidelines, its scope will follow the same cautious approach applied to grey literature. In practical terms this means focussing the search of grey literature/grey data to key source material which will be defined in the inclusion/exclusion criteria. Furthermore, in grey data searches, a key consideration is the ownership of the material and therefore its stewardship (35). Whilst the data is publicly available, there is still the question of its potentially sensitive nature and therefore for ethical reasons and for pragmatic purposes, the data extraction from the source of the grey data will be presented as anonymised and in broad narrative concepts in line with Coulson's use of Cutrona & Suhr's coding system for online bulletin boards. Guidance was also taken from the British Psychological Society's framework on internet mediated research (36) and ethical approval sought and granted through the primary author's University Ethics Review Board (ERN_21-1028A).

Aim 3) *show the organisation of findings using both a mixture of both visual and narrative synthesis in line with current best methodological practice.* See analysis and synthesis plan below.

Eligibility and inclusion criteria

The review will consider sources of English language evidence which report on adults who are over the age of 18 who are gene positive, presymptomatic or have manifest HD.

Exclusion criteria

As the protocol forms part of a PhD project, resources and timelines will limit the scope of the literature search and for this reason non-English language evidence will be excluded.

Information sources

Search strategy

The searches will be conducted in 5 stages

1) The umbrella scoping review of pre-existing systematic reviews starting with Elamin (20)
2) A further (non-umbrella) scoping review of quantitative and mixed methods literature on social cognition, social behaviour, and social functioning to the current date. The timeline for the search will run from the first paper annotated in Elamin's review (2003) up to the current date (March 2023). The databases to be used will be Pubmed, Medline, Psychinfo, Web of Science, Scopus, Embase and Cinahl.
3) A scoping review of the qualitative literature concerning social cognition, social behaviour and social functioning. This will match the dates of the pre-existing systematic reviews and run up to the current date. The databases to be used will be the same as for point 3.

4) A grey literature scope using Grey Guide, Google Scholar and Researchgate. The timeline for the search will be the same as at points 1-3.
5) A grey data scope using a UK HD charity affiliated online network. In line with the agreed ethics approval and to reinforce anonymity but not abandon the replicability and therefore rigour of the review, the criteria for this element of the review will not be published. Readers of the review may request the inclusion/exclusion criteria and key words for the grey data search by writing to the author.

Search terms

Key words for the umbrella scoping review, scoping reviews and grey literature scope:

Huntington* AND
Social cogniti* / Social function / Social behaviour / Social interaction / Social judgement / Social psychology / Social understanding / Social skills / Social competence / Social communication / Social decision making
Social withdrawal / avoidance / disengage
Social quality of life
Socialization
Relationships / interpersonal relationships
Social networks / social connections
Work
Sex
Non-verbal communication
Body language
Facial expression
Recognition
Theory of mind (TOM) / simulation theory
Empath* / empath* communication
Mentalizing / mental state
Emotion / emotional recognition
Inference
Functional skills

Data extraction and data summary

Qualitative, quantitative, and mixed method studies:

In line with the recommended PRISMA guidelines (31) particularly to show the conduct (and therefore rigour) of a review, data relevant to the aims of the review will be extracted from the data sources and 'charted' using an extraction form to summarise the sources' main findings. Whilst this is not a systematic review and therefore methodological quality of the sources is not the main aim, quality cannot be disregarded and in order to optimise the organisation of the synthesis (and thus quality) the Critical Appraisal Skills Programme (CASP) tool forms matched to the method of the paper (i.e. qualitative) will be used as the extraction forms (37).

Grey literature:

Review of the grey literature will use the AACODS (Authority, Accuracy, Coverage, Objectivity, Date and Significance) checklist designed by Jess Tyndall of Flinders University in 2010 and accepted as the baseline of good review practice (33).

For peer review only

Analysis and synthesis

As Popay (28) notes, in a review, analysis and synthesis co-exist in that evidence synthesis involves some form of analysis e.g. the finding of common themes across studies or the "interpretation of results from multiple studies, with the aim of producing new knowledge/findings".

Popay goes on to state that these different types of synthesis can be located along a continuum from quantitative approaches to qualitative approaches and as recommended in best practice literature the narrative synthesis is the bridge between all of these with other synthesis approaches used in an iterative way depending on the heterogeneity of research designs and findings.

Therefore - after the data extraction, the data from each phase of the search strategy will be entered into their own matrix (using Excel) which will allow for the collation and therefore review of key themes. This framework method which comes from the family of content and thematic analysis methods (38) will lead to the production of the following -

1) An umbrella scoping review of social cognition will be presented through a textual description (alongside a tabular form) in clustered groupings (illustrating types of studies) with relationships between and across studies in a narrative and visual form.
2) A scoping review of social behaviour and functioning which will include the qualitative and grey literature as well as the grey data will also be presented in a tabular form with the main concepts (and relationships) described in a narrative and visual form.
3) A final synthesis will be in a narrative form but also illustrated with a concepts map (39).

Public and service user involvement

This protocol is part of a wider PhD study on social cognition in HD using ethnography as a method. As the engagement of the HD community was of primary importance, the PPI group of the HDA was approached to comment on the method to see if it would be possible and acceptable but also whether the outcomes would be of value to them.

Ethics and dissemination

The PhD study which forms the background to this protocol has received ethical approval from the University of Birmingham's (UOB) STEM ERB with a further addendum specific to this protocol also approved for the internet mediated search of grey data.

The HD community will continue to be involved throughout the life cycle of the study with regular updates at HDA family events and participants will receive personal updates and summaries as requested. The final study will form a standalone piece on the HDA's website.

Discussion

Social cognition is a set of fundamental skills which are thought to enable successful social interaction. People with HD are known to have both problems with the brain structures associated with social cognition and when tested show this. The tests often take place in controlled settings. HD scientists and neuroscientists want to know more about what happens with social cognition in real life.

This protocol represents the plan for the weaving together of the information we already know from controlled settings alongside of additional observations from the scientific literature on socially related activity in HD as well as evidence from grey literature and grey data. This is intended to form a holistic map of the state of our knowledge of social cognition in HD so that gaps in that knowledge can be targeted; but also, that the knowledge from the review can inform clinical practice.

For peer review only

Author affiliations

1. Institute of Applied Heath Research, University of Birmingham, UK
2. Neuropsychiatry, Department of Clinical & Experimental Medicine, University of Birmingham, UK
3. West Midlands Huntington's Disease Service, Birmingham and Solihull Mental Health Foundation Trust, Birmingham, UK

Contributors: The final version was read and approved by all authors (AF, AL, SG and HR)

Funding: The protocol has no associated funding

Competing interests: AF, AL, SG and HR have no conflicting interests to declare

Correspondence to: alf944@bham.ac.uk

1. Henry JD. Clinical assessment of social cognitive function in neurological disorders. Nature Reviews Neurology. 2016;12(1):28-40.
2. Bora E, Velakoulis D, Walterfang M. Social cognition in Huntington's disease: A meta-analysis. Behav Brain Res. 2016;297:131-40.
3. Henley SM, Novak MJ, Frost C, King J, Tabrizi SJ, Warren JD. Emotion recognition in Huntington's disease: a systematic review. Neuroscience & Biobehavioral Reviews. 2012;36(1):237-53.
4. Kennedy DP, Adolphs R. The social brain in psychiatric and neurological disorders. Trends Cogn Sci. 2012;16(11):559-72.
5. Aubeeluck A. Huntington's disease. Part 3: family aspects of HD. British Journal of Nursing. 2008;17(5):328-32.
6. Vamos M, Hambridge J, Edwards M, Conaghan J. The impact of Huntington's disease on family life. Psychosomatics. 2007;48(5):400-4.
7. Williams JK, Hamilton R, Nehl C, McGonigal-Kenney M, Schutte DL, Sparbel K, et al. "No one else sees the difference:" family members' perceptions of changes in persons with preclinical Huntington disease. American Journal of Medical Genetics Part B: Neuropsychiatric Genetics. 2007;144(5):636-41.
8. Brossman B, Williams JK, Downing N, Mills JA, Paulsen JS, Investigators P-H, et al. Development of the Huntington disease work function scale. Journal of occupational and environmental medicine/American College of Occupational and Environmental Medicine. 2012;54(10):1300.
9. Downing NR, Williams JK, Paulsen JS. Couples' attributions for work function changes in prodromal Huntington disease. Journal of genetic counseling. 2010;19(4):343-52.
10. Van Der Zwaan KF, Mentink MDC, Jacobs M, Roos RAC, De Bot ST. Huntington's disease influences employment before and during clinical manifestation: A systematic review. Parkinsonism & Related Disorders. 2022;96:100-8.
11. Fisher F, Andrews S, Churchyard A, Mathers S. Home or Residential Care? The Role of Behavioral and Psychosocial Factors in Determining Discharge Outcomes for Inpatients with Huntington's Disease. Journal of Huntington's disease. 2012;1(2):187-93.
12. Mason SL, Schaepers M, Barker RA. Problems with Social Cognition and Decision-Making in Huntington's Disease: Why Is it Important? Brain Sciences. 2021;11(7):838.
13. Stout JC, Glikmann-Johnston Y, Andrews SC. Cognitive assessment strategies in Huntington's disease research. Journal of neuroscience methods. 2016;265:19-24.

For peer review only

14. Hadley LV, Naylor G, Hamilton AFdC. A review of theories and methods in the science of face-to-face social interaction. Nature Reviews Psychology. 2022;1(1):42-54.
15. Arksey H, O'Malley L. Scoping studies: towards a methodological framework. International journal of social research methodology. 2005;8(1):19-32.
16. Munn Z, Peters MD, Stern C, Tufanaru C, McArthur A, Aromataris E. Systematic review or scoping review? Guidance for authors when choosing between a systematic or scoping review approach. BMC medical research methodology. 2018;18(1):1-7.
17. Simpson J, Dale M, Theed R, Gunn S, Zarotti N, Eccles FJ. Validity of irritability in Huntington's disease: A scoping review. Cortex. 2019;120:353-74.
18. Mahmood S, Law S, Bombard Y. "I have to start learning how to live with becoming sick": A scoping review of the lived experiences of people with Huntington's disease. Clinical Genetics. 2022;101(1):3-19.
19. Gibson JS, Springer K. Social Withdrawal in Huntington's Disease: A Scoping Review. Journal of Huntington's Disease. 2022(Preprint):1-8.
20. Elamin M, Pender N, Hardiman O, Abrahams S. Social cognition in neurodegenerative disorders: a systematic review. Journal of Neurology, Neurosurgery & Psychiatry. 2012;83(11):1071-9.
21. Hoffecker L. Grey Literature Searching for Systematic Reviews in the Health Sciences. The Serials Librarian. 2020;79(3-4):252-60.
22. Pope C. Synthesizing qualitative and quantitative health evidence : a guide to methods / Catherine Pope, Nicholas Mays and Jennie Popay. Mays N, Popay J, editors: Maidenhead, England ; New York, NY : Open University Press, McGraw Hill Education, 2007.; 2007.
23. Paez A. Gray literature: An important resource in systematic reviews. Journal of Evidence-Based Medicine. 2017;10(3):233-40.
24. Adams J, Hillier-Brown FC, Moore HJ, Lake AA, Araujo-Soares V, White M, et al. Searching and synthesising 'grey literature'and 'grey information'in public health: critical reflections on three case studies. Systematic reviews. 2016;5(1):1-11.
25. Coulson NS, Buchanan H, Aubeeluck A. Social support in cyberspace: a content analysis of communication within a Huntington's disease online support group. Patient education and counseling. 2007;68(2):173-8.
26. Booth A, Sutton A, Clowes M, Martyn-St James M. Systematic approaches to a successful literature review. 2021.
27. Lisy K, Porritt K. Narrative synthesis: considerations and challenges. JBI Evidence Implementation. 2016;14(4):201.
28. Popay J, Roberts H, Sowden A, Petticrew M, Arai L, Rodgers M, et al. Guidance on the conduct of narrative synthesis in systematic reviews. A product from the ESRC methods programme Version. 2006;1(1):b92.
29. Grant MJ, Booth A. A typology of reviews: an analysis of 14 review types and associated methodologies. Health information & libraries journal. 2009;26(2):91-108.
30. Aromataris E, Fernandez R, Godfrey CM, Holly C, Khalil H, Tungpunkom P. Summarizing systematic reviews: methodological development, conduct and reporting of an umbrella review approach. JBI Evidence Implementation. 2015;13(3):132-40.
31. Tricco AC, Lillie E, Zarin W, O'Brien KK, Colquhoun H, Levac D, et al. PRISMA extension for scoping reviews (PRISMA-ScR): checklist and explanation. Annals of internal medicine. 2018;169(7):467-73.
32. Stern C, Lizarondo L, Carrier J, Godfrey C, Rieger K, Salmond S, et al. Methodological guidance for the conduct of mixed methods systematic reviews. JBI evidence synthesis. 2020;18(10):2108-18.
33. Chang S. Health Libraries Inc. presentation:'Health librarians and grey literature-what do we need to know?'. Health Inform. 2014;23(2):45-6.
34. Savić D. When is 'grey'too 'grey'?-A case of grey data. The Grey Journal. 2019;15(2):71-6.

For peer review only

35. Borgman CL. Open data, grey data, and stewardship: Universities at the privacy frontier. Berkeley Technology Law Journal. 2018;33(2):365-412.
36. Hewson C. British Psychological Society - Ethics Guidelines for Internet Mediated Research www.bps.org.uk2021 [Available from: https://www.bps.org.uk/sites/www.bps.org.uk/files/Policy/Policy%20-%20Files/Ethics%20Guidelines%20for%20Internet-mediated%20Research.pdf.
37. Long HA, French DP, Brooks JM. Optimising the value of the critical appraisal skills programme (CASP) tool for quality appraisal in qualitative evidence synthesis. Research Methods in Medicine & Health Sciences. 2020;1(1):31-42.
38. Hughes SE, Aiyegbusi OL, Lasserson DS, Collis P, Rivera SC, McMullan C, et al. Protocol for a scoping review exploring the use of patient-reported outcomes in adult social care. BMJ open. 2021;11(4):e045206.
39. Ligita T, Nurjannah I, Wicking K, Harvey N, Francis K. From textual to visual: the use of concept mapping as an analytical tool in a grounded theory study. Qualitative Research. 2022;22(1):126-42.

For peer review only

Annals of Internal Medicine RESEARCH AND REPORTING METHODS

PRISMA Extension for Scoping Reviews (PRISMA-ScR): Checklist and Explanation

Andrea C. Tricco, PhD, MSc; Erin Lillie, MSc; Wasifa Zarin, MPH; Kelly K. O'Brien, PhD, BScPT; Heather Colquhoun, PhD; Danielle Levac, PhD, MSc, BScPT; David Moher, PhD, MSc; Micah D.J. Peters, PhD, MA(Q); Tanya Horsley, PhD; Laura Weeks, PhD; Susanne Hempel, PhD; Elie A. Akl, MD, PhD, MPH; Christine Chang, MD, MPH; Jessie McGowan, PhD; Lesley Stewart, PhD, MSc; Lisa Hartling, PhD, MSc, BScPT; Adrian Aldcroft, BA(Hons), BEd; Michael G. Wilson, PhD; Chantelle Garritty, MSc; Simon Lewin, PhD; Christina M. Godfrey, PhD, RN; Marilyn T. Macdonald, PhD, MSN; Etienne V. Langlois, PhD; Karla Soares-Weiser, MD, PhD; Jo Moriarty, MA; Tammy Clifford, PhD, MSc; Özge Tunçalp, MD, PhD, MPH; and Sharon E. Straus, MD, MSc

Scoping reviews, a type of knowledge synthesis, follow a systematic approach to map evidence on a topic and identify main concepts, theories, sources, and knowledge gaps. Although more scoping reviews are being done, their methodological and reporting quality need improvement. This document presents the PRISMA-ScR (Preferred Reporting Items for Systematic reviews and Meta-Analyses extension for Scoping Reviews) checklist and explanation. The checklist was developed by a 24-member expert panel and 2 research leads following published guidance from the EQUATOR (Enhancing the QUAlity and Transparency Of health Research) Network. The final checklist contains 20 essential reporting items and 2 optional items. The authors provide a rationale and an example of good reporting for each item. The intent of the PRISMA-ScR is to help readers (including researchers, publishers, commissioners, policymakers, health care providers, guideline developers, and patients or consumers) develop a greater understanding of relevant terminology, core concepts, and key items to report for scoping reviews.

Ann Intern Med. 2018;169:467-473. doi:10.7326/M18-0850 **Annals.org**
For author affiliations, see end of text.
This article was published at Annals.org on 4 September 2018.

For peer review only

Scoping reviews can be conducted to meet various objectives. They may examine the extent (that is, size), range (variety), and nature (characteristics) of the evidence on a topic or question; determine the value of undertaking a systematic review; summarize findings from a body of knowledge that is heterogeneous in methods or discipline; or identify gaps in the literature to aid the planning and commissioning of future research (1, 2). A recent scoping review by members of our team suggested that although the number of scoping reviews in the literature is increasing steadily, methodological and reporting quality needs to improve in order to facilitate complete and transparent reporting (1). Results from a survey on scoping review terminology, definitions, and methods showed a lack of consensus on how to conduct and report scoping reviews (3).

The Joanna Briggs Institute (JBI) published a guidance document for the conduct of scoping reviews (4) (updated in 2017 [5]) based on earlier work by Arksey and O'Malley (6) and Levac and colleagues (7). However, a reporting guideline for scoping reviews currently does not exist.

Reporting guidelines outline a minimum set of items to include in research reports and have been shown to increase methodological transparency and uptake of research findings (8, 9). Although a reporting guideline exists for systematic reviews—the PRISMA (Preferred Reporting Items for Systematic reviews and Meta-Analyses) statement (10)—scoping reviews serve a different purpose (11). Systematic reviews are useful for answering clearly defined questions (for example, "Does this intervention improve specified outcomes when compared with a given comparator in this population?"), whereas scoping reviews are useful for answering much broader questions (such as "What is the nature of the evidence for this intervention?" or "What is known about this concept?"). Given the difference in objectives, and therefore in the methodological approach (such as presence vs. absence of a risk-of-bias assessment or meta-analysis), scoping reviews should have different essential reporting items from systematic reviews. Consequently, some PRISMA items may not be appropriate, whereas other important considerations may be missing (12–14). It was decided that a PRISMA extension for scoping reviews was needed to provide reporting guidance for this specific type of knowledge synthesis. This extension is also intended to apply to evidence maps (15, 16), which share similarities with scoping reviews and involve a systematic search of a body of literature to identify knowledge gaps, with a visual representation of results (such as a figure or graph).

METHODS

The PRISMA-ScR (PRISMA extension for Scoping Reviews) was developed according to published guidance by the EQUATOR (Enhancing the QUAlity and Transparency Of health Research) Network for the development of reporting guidelines (9). The St. Michael's Hospital Research Ethics Board granted research ethics approval for this study on 15 August 2016.

See also:

Editorial comment 502

Web-Only
Appendix: Explanation and Elaboration
Supplement

For peer review only - http://bmjopen.bmj.com/site/about/guidelines.xhtml

Protocol, Advisory Board, and Expert Panel

Our protocol was drafted by the research team and revised as necessary by the advisory board before being listed as a reporting guideline on the EQUATOR (17) and PRISMA (18) Web sites. The research team included 2 leads (A.C.T. and S.E.S.) and 2 research coordinators (E.L. and W.Z.), none of whom participated in the scoring exercises, and a 4-member advisory board (K.K.O., H.C., D.L., and D.M.) with extensive experience doing scoping reviews or developing reporting guidelines. We aimed to form an expert panel of approximately 30 members that would be representative of different geography and stakeholder types and research experiences, including persons with experience in the conduct, dissemination, or uptake of scoping reviews.

Survey Development and Round 1 of Delphi

The initial step in developing the Delphi survey via Qualtrics (an online survey platform) (19) involved identifying potential modifications to the original 27-item PRISMA checklist. The modifications were based on a research program carried out by members of the advisory board to better understand scoping review practices (1, 3, 20) and included a broader research question and literature search strategy, optional risk-of-bias assessment and consultation exercise (whereby relevant stakeholders contribute to the work, as described by Arksey and O'Malley [6]), and a qualitative analysis. For round 1 of scoring, we prepared a draft of the PRISMA-ScR (**Supplement**, available at Annals.org) and asked expert panel members to rate their agreement with each of the proposed reporting items using a 7-point Likert scale (1 = "entirely disagree," 2 = "mostly disagree," 3 = "somewhat disagree," 4 = "neutral," 5 = "somewhat agree," 6 = "mostly agree," and 7 = "entirely agree"). Each survey item included an optional text box where respondents could provide comments. The research team calibrated the survey for content and clarity before administering it and sent biweekly reminders to optimize participation.

Survey Analysis

To be conservative, a threshold for 85% agreement was established a priori for each of the reporting items to indicate consensus among the expert panel. This rule required that at least 85% of the panel mostly or entirely agreed (values of 6 or 7 on the Likert scale) with the inclusion of the item in the PRISMA-ScR. If agreement was less than 85%, it was considered to be discrepant. This standard was used for all 3 rounds of scoring to inform the final checklist. For ease and consistency with how the survey questions were worded, we did not include a provision for agreement on exclusion (that is, 85% of answers corresponding to values of 1 or 2 on the Likert scale). All comments were summarized to help explain the scorings and identify any issues. For the analysis, the results were stratified by group (in-person meeting vs. online, hereafter e-Delphi) because discrepant items could differ between groups.

In-Person Group (Round 2 of Delphi)

The Chatham House rule (21) was established at the beginning of the meeting, whereby participants were free to use information that is shared but were not permitted to reveal the identity or affiliation of the speaker. Expert panel members were given their individual results; the overall group distribution, median, and interquartile range; a summary of the JBI methodological guidance (4); and preliminary feedback from the e-Delphi group. These data were used to generate and inform the discussion about each discrepant item from round 1. Two researchers (A.C.T. and S.E.S.) facilitated the discussion using a modified nominal group technique (22) to reach consensus. Panel members were subsequently asked to rescore the discrepant items using sli.do (23), a live audience-response system in a format that resembled the round 1 survey. For items that failed to meet the threshold for consensus, working groups were assembled. The meeting was audio-recorded and transcribed using TranscribeMe (24), and 3 note-takers independently documented the main discussion points. The transcript was annotated to complement a master summary of the discussion points, which was compiled using the 3 note-takers' files.

E-Delphi Group (Round 2 of Delphi)

Those who could not attend the in-person meeting participated via an online discussion exercise using Conceptboard (25), a visual collaboration platform that allows users to provide feedback on "whiteboards" in real time. The discrepant items from round 1 were presented as a single whiteboard, and questions (for example, "After reviewing your survey results with respect to this item, please share why you rated this item the way you did") were assigned to participants as tasks to facilitate the discussion. E-Delphi panel members received the same materials as in-person participants and were encouraged to respond to others' comments and interact through a chat feature. The second round of scoring was done in Qualtrics using a similar format as in round 1. A summary of the Conceptboard discussion, as well as the annotated meeting transcript and master summary document, were shared so that participants could learn about the perspectives of the in-person group before rescoring.

Working Groups and Round 3 of Delphi

To enable panel-wide dialogue and refine the checklist items before the final round of scoring, working groups were created and collaborated by teleconference and e-mail. Their task was to discuss the discrepant items in terms of the key issues and considerations (relating to both concepts and wording) that had been raised in earlier stages across both groups. To harmonize the data from the 2 groups, a third round of scoring exercise was administered using Qualtrics (19). In this step, suggested modifications (in terms of both concepts and wording) from all previous stages were incorporated into the items that had failed to reach consensus in the first 2 rounds across both groups, and the full panel scored this updated list.

For peer review only - http://bmjopen.bmj.com/site/about/guidelines.xhtml

Figure. Methods flow chart.

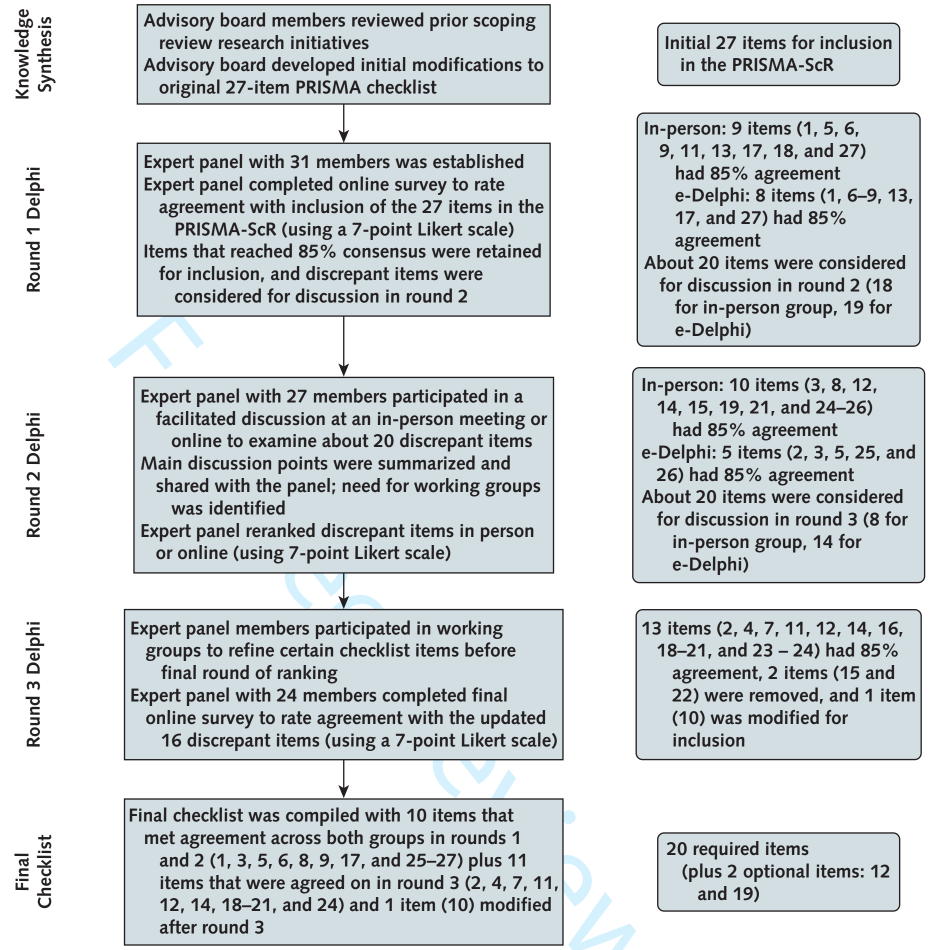

PRISMA = Preferred Reporting Items for Systematic reviews and Meta-Analyses; PRISMA-ScR = PRISMA extension for Scoping Reviews.

Interactive Workshop (Testing)

A workshop led by the lead investigator (A.C.T.) and facilitated by members of the advisory board and expert panel (S.E.S., C.M.G., C.G., T.H., M.T.M., and M.D.J.P.) was held as part of the Global Evidence Summit in Cape Town, South Africa, in September 2017. Participants (researchers, scientists, policymakers, managers, and students) tested the checklist by applying the PRISMA-ScR to a scoping review on a health-related topic (26).

Role of the Funding Source

This work was supported by a grant from the Canadian Institutes of Health Research. The funding source had no role in designing the study; collecting, analyzing, or interpreting the data; writing the manuscript; or deciding to submit it for publication.

RESULTS

Expert Panel

A total of 37 persons were invited to participate, of whom 31 completed round 1 and 24 completed all 3 rounds of scoring. The **Figure** presents results of the modified Delphi, including the number of items that met agreement at each stage.

Round 1 of Delphi

For the in-person group, which involved 16 participants, 9 of 27 items reached agreement. For the discrepant items, agreement ranged from 56% for item 15 (risk of bias) to 81% for items 3 (rationale), 16 (additional analyses), 20 (results of individual sources), and 23 (additional analyses). For the e-Delphi group, which involved 15 participants, 8 of 27 items met the 85% agreement threshold. For the discrepant items, agreement ranged from 40% for item 12 (risk of bias) to 80% for items 3 (rationale), 25 (limitations), and 26 (conclusions).

In-Person Meeting and Round 2 of Delphi

The 16 panel members who attended the in-person meeting in Toronto on 29 November 2016 were largely from North America, although a few were from Australia, Lebanon, or the United Kingdom. Of the 18

For peer review only - http://bmjopen.bmj.com/site/about/guidelines.xhtml

discrepant items from round 1, the panel decided to rescore 11 of the items after facilitated discussion. All, except item 7 (information sources), reached the 85% threshold for agreement in the rescoring exercise. For the remaining 7 of the 18 discrepant items, the group believed that notable changes were required, which formed the basis of action by the working groups.

E-Delphi Discussion and Round 2 of Delphi

Fifteen panel members were invited to participate in the online discussion exercise, from countries that included Canada, the United Kingdom, Switzerland, Norway, and South Africa. Of these, 50% (7 of 14 panelists) participated in at least 1 discussion on Conceptboard (25) and 1 dropped out. Eleven participants completed the second scoring exercise of the 19 discrepant items, whereby 5 items reached 85% agreement.

Working Groups and Round 3 of Delphi

The 6 working groups (with 1 call per group) ranged in size from 3 to 8 participants, with an average of 5 per group. Round 3 of the Delphi did not include the 11 items that reached consensus during round 1 or 2 across both the in-person and e-Delphi groups. The survey focused on the remaining 16 items that failed to reach consensus across both groups to ensure that one group's decisions did not take precedence over the other group.

A total of 27 persons were invited to participate in round 3 of the Delphi—16 from the in-person group and 11 from the e-Delphi group. Overall, 24 of 27 participants completed the final round of scoring, and 3 withdrew (2 from the in-person group and 1 from the e-Delphi). Two of the 16 applicable items—10 (data collection process) and 15 (risk of bias across studies)—failed to meet the 85% agreement threshold. Item 15 was subsequently removed from the checklist (along with its companion item, 22), whereas item 10 was retained but revised to exclude the optional consultation exercise described by Arksey and O'Malley (6) and Levac and colleagues (7), which was the source of the disagreement. Participants decided that the consultation exercise could be considered a knowledge translation activity, which could be done for any type of knowledge synthesis.

Interactive Workshop (Testing)

A total of 30 participants attended an interactive workshop at the Global Evidence Summit in September 2017 in Cape Town, South Africa, where minor revisions were suggested for wording of the items.

PRISMA-ScR Checklist

The **Table** presents the final checklist, which includes 20 items plus 2 optional items. It consists of 10 items that reached agreement in rounds 1 and 2 (items 1, 3, 5, 6, 8, 9, 17, 25, 26, and 27) and 9 that were agreed on in round 3 (items 2, 4, 7, 11, 14, 18, 20, 21, and 24), along with 1 item (item 10) that was modified for inclusion after the final round. Five items from the original PRISMA checklist were deemed not relevant. They were item 13 (summary measures), which reached agreement as not applicable for scoping reviews in round 1, and the following 4 items: item 15 (risk of bias across studies) and item 22 (risk of bias across study results, companion to item 15), which were excluded after round 3, along with item 16 (additional analyses) and item 23 (additional analyses results, companion to item 16), which reached agreement as not applicable for scoping reviews in round 3. The **Figure** illustrates this process. In addition, because scoping reviews can include many types of evidence (such as documents, blogs, Web sites, studies, interviews, and opinions) and do not examine the risk of bias of included sources, items 12 (risk of bias in individual studies) and 19 (risk of bias within study results) from the original PRISMA are treated as optional in the PRISMA-ScR.

PRISMA-ScR Explanation and Elaboration

The **Appendix** (available at Annals.org) elaborates on the PRISMA-ScR checklist items. It defines each item and gives examples of good reporting from existing scoping reviews to provide authors with additional guidance on how to use the PRISMA-ScR.

DISCUSSION

The PRISMA-ScR is intended to provide guidance on the reporting of scoping reviews. To develop this PRISMA extension, the original PRISMA statement was adapted and the following revisions were made: 5 items were removed (because they were deemed not relevant to scoping reviews), 2 items were deemed optional, and the wording was modified for all items. This reporting guideline is consistent with the JBI guidance for scoping reviews, which highlights the importance of methodological rigor in the conduct of scoping reviews. It is hoped that the PRISMA-ScR will improve the reporting of scoping reviews and increase their relevance for decision making and that adherence to our reporting guideline will be evaluated in the future, which will be critical to measure its impact.

The PRISMA-ScR will be housed on the EQUATOR Network's Web site and the Knowledge Translation Program Web site of St. Michael's Hospital (27). To promote its uptake, a 1-minute YouTube video will be created outlining how to operationalize each item, webinars for organizations that do scoping reviews will be offered, and a 1-page tip sheet for each item will be created. In the future, an automated e-mail system may be considered, whereby authors would be sent the PRISMA-ScR upon registering their scoping review, as well as an online tool similar to Penelope, which verifies manuscripts for completeness and provides feedback to authors (28). The PRISMA-ScR will be shared widely within our networks, including the Alliance for Health Policy and Systems Research, the World Health Organization (29), and the Global Evidence Synthesis Initiative (30). Finally, ongoing feedback and suggestions to improve uptake of the PRISMA-ScR will be collected via an online form on the Web site for the Knowledge Translation Program of St. Michael's Hospital (27).

For peer review only - http://bmjopen.bmj.com/site/about/guidelines.xhtml

Table. PRISMA-ScR Checklist

Section	Item	PRISMA-ScR Checklist Item
**Title**	1	Identify the report as a scoping review.
**Abstract**		
Structured summary	2	Provide a structured summary that includes (as applicable) background, objectives, eligibility criteria, sources of evidence, charting methods, results, and conclusions that relate to the review questions and objectives.
**Introduction**		
Rationale	3	Describe the rationale for the review in the context of what is already known. Explain why the review questions/objectives lend themselves to a scoping review approach.
Objectives	4	Provide an explicit statement of the questions and objectives being addressed with reference to their key elements (e.g., population or participants, concepts, and context) or other relevant key elements used to conceptualize the review questions and/or objectives.
**Methods**		
Protocol and registration	5	Indicate whether a review protocol exists; state if and where it can be accessed (e.g., a Web address); and if available, provide registration information, including the registration number.
Eligibility criteria	6	Specify characteristics of the sources of evidence used as eligibility criteria (e.g., years considered, language, and publication status), and provide a rationale.
Information sources*	7	Describe all information sources in the search (e.g., databases with dates of coverage and contact with authors to identify additional sources), as well as the date the most recent search was executed.
Search	8	Present the full electronic search strategy for at least 1 database, including any limits used, such that it could be repeated.
Selection of sources of evidence†	9	State the process for selecting sources of evidence (i.e., screening and eligibility) included in the scoping review.
Data charting process‡	10	Describe the methods of charting data from the included sources of evidence (e.g., calibrated forms or forms that have been tested by the team before their use, and whether data charting was done independently or in duplicate) and any processes for obtaining and confirming data from investigators.
Data items	11	List and define all variables for which data were sought and any assumptions and simplifications made.
Critical appraisal of individual sources of evidence§	12	If done, provide a rationale for conducting a critical appraisal of included sources of evidence; describe the methods used and how this information was used in any data synthesis (if appropriate).
Summary measures	13	Not applicable for scoping reviews.
Synthesis of results	14	Describe the methods of handling and summarizing the data that were charted.
Risk of bias across studies	15	Not applicable for scoping reviews.
Additional analyses	16	Not applicable for scoping reviews.
**Results**		
Selection of sources of evidence	17	Give numbers of sources of evidence screened, assessed for eligibility, and included in the review, with reasons for exclusions at each stage, ideally using a flow diagram.
Characteristics of sources of evidence	18	For each source of evidence, present characteristics for which data were charted and provide the citations.
Critical appraisal within sources of evidence	19	If done, present data on critical appraisal of included sources of evidence (see item 12).
Results of individual sources of evidence	20	For each included source of evidence, present the relevant data that were charted that relate to the review questions and objectives.
Synthesis of results	21	Summarize and/or present the charting results as they relate to the review questions and objectives.
Risk of bias across studies	22	Not applicable for scoping reviews.
Additional analyses	23	Not applicable for scoping reviews.
**Discussion**		
Summary of evidence	24	Summarize the main results (including an overview of concepts, themes, and types of evidence available), link to the review questions and objectives, and consider the relevance to key groups.
Limitations	25	Discuss the limitations of the scoping review process.
Conclusions	26	Provide a general interpretation of the results with respect to the review questions and objectives, as well as potential implications and/or next steps.
**Funding**	27	Describe sources of funding for the included sources of evidence, as well as sources of funding for the scoping review. Describe the role of the funders of the scoping review.

JBI = Joanna Briggs Institute; PRISMA-ScR = Preferred Reporting Items for Systematic reviews and Meta-Analyses extension for Scoping Reviews.
* Where *sources of evidence* (see second footnote) are compiled from, such as bibliographic databases, social media platforms, and Web sites.
† A more inclusive/heterogeneous term used to account for the different types of evidence or data sources (e.g., quantitative and/or qualitative research, expert opinion, and policy documents) that may be eligible in a scoping review as opposed to only studies. This is not to be confused with *information sources* (see first footnote).
‡ The frameworks by Arksey and O'Malley (6) and Levac and colleagues (7) and the JBI guidance (4, 5) refer to the process of data extraction in a scoping review as data charting.
§ The process of systematically examining research evidence to assess its validity, results, and relevance before using it to inform a decision. This term is used for items 12 and 19 instead of "risk of bias" (which is more applicable to systematic reviews of interventions) to include and acknowledge the various sources of evidence that may be used in a scoping review (e.g., quantitative and/or qualitative research, expert opinion, and policy documents).

For peer review only - http://bmjopen.bmj.com/site/about/guidelines.xhtml

From St. Michael's Hospital and University of Toronto, Toronto, Ontario, Canada (A.C.T., S.E.S.); St. Michael's Hospital, Toronto, Ontario, Canada (E.L., W.Z.); University of Toronto, Toronto, Ontario, Canada (K.K.O., H.C.); Northeastern University, Boston, Massachusetts (D.L.); Ottawa Hospital Research Institute, Ottawa, Ontario, Canada (D.M., C.G.); University of South Australia and University of Adelaide, Adelaide, South Australia, Australia (M.D.J.P.); Royal College of Physicians and Surgeons of Canada, Ottawa, Ontario, Canada (T.H.); Canadian Agency for Drugs and Technologies in Health, Ottawa, Ontario, Canada (L.W., T.C.); RAND Corporation, Santa Monica, California (S.H.); American University of Beirut, Beirut, Lebanon (E.A.A.); Agency for Healthcare Research and Quality, Rockville, Maryland (C.C.); University of Ottawa, Ottawa, Ontario, Canada (J.M.); University of York, York, United Kingdom (L.S.); University of Alberta, Edmonton, Alberta, Canada (L.H.); BMJ Open, London, United Kingdom (A.A.); McMaster University, Hamilton, Ontario, Canada (M.G.W.); Norwegian Institute of Public Health, Oslo, Norway, and South African Medical Research Council, Cape Town, South Africa (S.L.); Queen's University, Kingston, Ontario, Canada (C.M.G.); Dalhousie University, Halifax, Nova Scotia, Canada (M.T.M.); World Health Organization, Geneva, Switzerland (E.V.L., Ö.T.); Cochrane, London, United Kingdom (K.S.); and King's College London, London, United Kingdom (J.M.).

Note: Dr. Tricco affirms that the manuscript is an honest, accurate, and transparent account of the study being reported; that no important aspects of the study have been omitted; and that any discrepancies from the study as planned (and, if relevant, registered) have been explained.

Acknowledgment: The authors thank Susan Le for supporting the coordination of the project and formatting the manuscript; Anna Lambrinos and Dr. Mai Pham for participating in round 1 of scoring and attending the in-person meeting; Dr. Lisa O'Malley for participating in round 1 of scoring and the e-Delphi round 2 of scoring; Dr. Peter Griffiths and Dr. Charles Shey Wiysonge for participating in round 1 of scoring and providing feedback on Conceptboard; Dr. Jill Manthorpe and Dr. Mary Ann McColl for participating in round 1 of scoring; Assem M. Khamis for assisting with the identification of examples for the Explanation and Elaboration document; and Melissa Chen, Jessica Comilang, and Meghan Storey for providing administrative support for the in-person meeting.

Financial Support: By Knowledge Synthesis grant KRS 144046 from the Canadian Institutes of Health Research. Dr. Tricco is funded by a Tier 2 Canada Research Chair in Knowledge Synthesis. Dr. O'Brien was supported by a Canadian Institutes of Health Research New Investigator Award. Dr. Straus is funded by a Tier 1 Canada Research Chair in Knowledge Translation.

Disclosures: Mr. Aldcroft reports that he is the editor of BMJ Open. Dr. Lewin reports that he is the joint coordinating editor for the Cochrane Effective Practice and Organisation of Care (EPOC) Group. Dr. Straus reports that she is an associate editor of ACP Journal Club. Authors not named here have disclosed no conflicts of interest. Disclosures can also be viewed at www.acponline.org/authors/icmje/ConflictOfInterestForms.do?msNum=M18-0850.

Reproducible Research Statement: *Study protocol:* Available at the EQUATOR and PRISMA Web sites (www.equator-network.org/library/reporting-guidelines-under-development/#55 and www.prisma-statement.org/Extensions/InDevelopment.aspx). *Statistical code:* Not applicable. *Data set:* Available from Dr. Tricco (e-mail, triccoa@smh.ca).

Corresponding Author: Andrea C. Tricco, PhD, MSc, Knowledge Translation Program, Li Ka Shing Knowledge Institute, St. Michael's Hospital, 209 Victoria Street, East Building, Toronto, Ontario M5B 1W8, Canada; e-mail, triccoa@smh.ca.

Current author addresses and author contributions are available at Annals.org.

References

1. **Tricco AC, Lillie E, Zarin W, O'Brien K, Colquhoun H, Kastner M, et al.** A scoping review on the conduct and reporting of scoping reviews. BMC Med Res Methodol. 2016;16:15. [PMID: 26857112] doi:10.1186/s12874-016-0116-4
2. **Canadian Institutes of Health Research.** A guide to knowledge synthesis: a knowledge synthesis chapter. 2010. Accessed at www.cihr-irsc.gc.ca/e/41382.html on 10 January 2018.
3. **Colquhoun HL, Levac D, O'Brien KK, Straus S, Tricco AC, Perrier L, et al.** Scoping reviews: time for clarity in definition, methods, and reporting. J Clin Epidemiol. 2014;67:1291-4. [PMID: 25034198] doi:10.1016/j.jclinepi.2014.03.013
4. **Peters MD, Godfrey CM, Khalil H, McInerney P, Parker D, Soares CB.** Guidance for conducting systematic scoping reviews. Int J Evid Based Healthc. 2015;13:141-6. [PMID: 26134548] doi:10.1097/XEB.0000000000000050
5. **Peters MDJ, Godfrey C, McInerney P, Baldini Soares C, Khalil H, Parker D.** Scoping reviews. In: Aromataris E, Munn Z, eds. Joanna Briggs Institute Reviewer's Manual. Adelaide, Australia: Joanna Briggs Inst; 2017.
6. **Arksey H, O'Malley L.** Scoping studies: towards a methodological framework. Int J Soc Res Methodol. 2005;8:19-32.
7. **Levac D, Colquhoun H, O'Brien KK.** Scoping studies: advancing the methodology. Implement Sci. 2010;5:69. [PMID: 20854677] doi:10.1186/1748-5908-5-69
8. **Altman DG, Simera I.** Using reporting guidelines effectively to ensure good reporting of health research. In: Moher D, Altman D, Schulz K, Simera I, Wager E, eds. Guidelines for Reporting Health Research: A User's Manual. Hoboken, NJ: J Wiley; 2014:32-40.
9. **Moher D, Schulz KF, Simera I, Altman DG.** Guidance for developers of health research reporting guidelines. PLoS Med. 2010;7:e1000217. [PMID: 20169112] doi:10.1371/journal.pmed.1000217
10. **Moher D, Liberati A, Tetzlaff J, Altman DG; PRISMA Group.** Preferred reporting items for systematic reviews and meta-analyses: the PRISMA statement. Ann Intern Med. 2009;151:264-9. [PMID: 19622511] doi:10.7326/0003-4819151-4-200908180-00135
11. **Tricco AC, Zarin W, Ghassemi M, Nincic V, Lillie E, Page MJ, et al.** Same family, different species: methodological conduct and quality varies according to purpose for five types of knowledge synthesis. J Clin Epidemiol. 2018;96:133-42. [PMID: 29103958] doi:10.1016/j.jclinepi.2017.10.014
12. **McInnes MD, Bossuyt PM.** Pitfalls of systematic reviews and meta-analyses in imaging research. Radiology. 2015;277:13-21. [PMID: 26402491] doi:10.1148/radiol.2015142779
13. **Macaskill P, Gatsonis C, Deeks JJ, Harbord RM, Takwoingi Y.** Analysing and presenting results. In: Deeks JJ, Bossuyt PM, Gatsonis C, eds. Cochrane Handbook for Systematic Reviews of Diagnostic Test Accuracy. Version 1.0. London: The Cochrane Collaboration; 2010. Accessed at https://methods.cochrane.org/sites/methods.cochrane.org.sdt/files/public/uploads/Chapter%2010%20-%20Version%201.0.pdf on 3 August 2018.

For peer review only - http://bmjopen.bmj.com/site/about/guidelines.xhtml

14. **Whiting PF, Rutjes AW, Westwood ME, Mallett S, Deeks JJ, Reitsma JB, et al; QUADAS-2 Group.** QUADAS-2: a revised tool for the quality assessment of diagnostic accuracy studies. Ann Intern Med. 2011;155:529-36. [PMID: 22007046] doi:10.7326/0003-4819-155-8-201110180-00009
15. **Schmucker C, Motschall E, Antes G, Meerpohl JJ.** [Methods of evidence mapping. A systematic review]. Bundesgesundheitsblatt Gesundheitsforschung Gesundheitsschutz. 2013;56:1390-7. [PMID: 23978984] doi:10.1007/s00103-013-1818-y
16. **Miake-Lye IM, Hempel S, Shanman R, Shekelle PG.** What is an evidence map? A systematic review of published evidence maps and their definitions, methods, and products. Syst Rev. 2016;5:28. [PMID: 26864942] doi:10.1186/s13643-016-0204-x
17. **EQUATOR Network.** Preferred Reporting Items for Systematic Reviews and Meta-Analysis extension for Scoping Reviews (PRISMA-ScR). 2017. Accessed at www.equator-network.org/library/reporting-guidelines-under-development/#55 on 10 January 2018.
18. **PRISMA.** Extensions in development: Preferred Reporting Items for Systematic Reviews and Meta-Analyses (PRISMA). 2015. Accessed at www.prisma-statement.org/Extensions/InDevelopment.aspx on 10 January 2018.
19. Qualtrics. 2018. Accessed at www.qualtrics.com/uk on 10 January 2018.
20. **O'Brien KK, Colquhoun H, Levac D, Baxter L, Tricco AC, Straus S, et al.** Advancing scoping study methodology: a web-based survey and consultation of perceptions on terminology, definition and methodological steps. BMC Health Serv Res. 2016;16:305. [PMID: 27461419] doi:10.1186/s12913-016-1579-z
21. **The Royal Institute of International Affairs.** Chatham House Rule. 2018. Accessed at www.chathamhouse.org/chatham-house-rule on 14 June 2018.
22. **Jones J, Hunter D.** Consensus methods for medical and health services research. BMJ. 1995;311:376-80. [PMID: 7640549]
23. Sli.do. 2012. Accessed at www.sli.do on 10 January 2018.
24. TranscribeMe. 2018. Accessed at https://transcribeme.com on 27 February 2018.
25. Conceptboard. 2018. Accessed at https://conceptboard.com on 14 June 2018.
26. **Lourida I, Abbott RA, Rogers M, Lang IA, Stein K, Kent B, et al.** Dissemination and implementation research in dementia care: a systematic scoping review and evidence map. BMC Geriatr. 2017;17:147. [PMID: 28709402] doi:10.1186/s12877-017-0528-y
27. Knowledge Translation Program. 2016. Accessed at https://knowledgetranslation.net on 10 January 2018.
28. Penelope. 2017. Accessed at www.penelope.ai on 28 February 2018.
29. **World Health Organization.** Alliance for Health Policy and Systems Research. 2018. Accessed at www.who.int/alliance-hpsr/en on 27 February 2018.
30. Global Evidence Synthesis Initiative. 2016. Accessed at www.gesiinitiative.com on 27 February 2018.

For peer review

NEW PEER REVIEWERS

Sign up to become a peer reviewer for *Annals of Internal Medicine* by going to www.annals.org/aim/pages/reviewers and selecting "Reviewer Information" to register as a new reviewer. Note that *Annals* reviewers whose reviews are returned on time and are judged satisfactory by the Editors may receive up to 3 Category 1 CME credits per review (maximum, 15 credits in a calendar year).

For peer review only - http://bmjopen.bmj.com/site/about/guidelines.xhtml

Current Author Addresses: Drs. Tricco and Straus, Ms. Lillie, and Ms. Zarin: Knowledge Translation Program, Li Ka Shing Knowledge Institute, St. Michael's Hospital, 209 Victoria Street, East Building, Toronto, Ontario M5B 1T8, Canada.
Dr. O'Brien: Department of Physical Therapy, University of Toronto, 160-500 University Avenue, Toronto, Ontario M5G 1V7, Canada.
Dr. Colquhoun: Department of Occupational Science and Occupational Therapy, University of Toronto, 160-500 University Avenue, Toronto, Ontario M5G 1V7, Canada.
Dr. Levac: Department of Physical Therapy, Movement and Rehabilitation Science, Bouvé College of Health Sciences, Northeastern University, 360 Huntington Avenue, Boston, MA 02115.
Dr. Moher and Ms. Garritty: Centre for Journalology, Ottawa Hospital Research Institute, The Ottawa Hospital, 501 Smyth Road, PO Box 201B, Ottawa, Ontario K1H 8L6, Canada.
Dr. Peters: Rosemary Bryant AO Research Centre, Sansom Institute for Health Research, Division of Health Sciences, University of South Australia, Adelaide, South Australia 5000, Australia.
Dr. Horsley: The Royal College of Physicians and Surgeons, 774 Echo Drive, Ottawa, Ontario K1S 5N8, Canada.
Drs. Weeks and Clifford: CADTH (Canadian Agency for Drugs and Technologies in Health), 865 Carling Avenue, Suite 600, Ottawa, Ontario K1S 5S8, Canada.
Dr. Hempel: RAND Corporation, 1776 Main Street, Santa Monica, CA 90401.
Dr. Akl: Department of Internal Medicine, Faculty of Medicine, Gefinor Center, Block B, 4th Floor, American University of Beirut, Riad El-Solh, Beirut, Lebanon.
Dr. Chang: Agency for Healthcare Research and Quality, 5600 Fishers Lane, Rockville, MD 20857.
Dr. McGowan: Department of Medicine, University of Ottawa, Roger Guindon Hall, 451 Smyth Road, Ottawa, Ontario K1H 8M5, Canada.
Dr. Stewart: Centre for Reviews and Dissemination, University of York, Heslington, York YO10 5DD, United Kingdom.
Dr. Hartling: Department of Pediatrics, Faculty of Medicine and Dentistry, University of Alberta, 11405-87 Avenue, Edmonton, Alberta T6G 1C9, Canada.
Mr. Aldcroft: BMJ Open Editorial Office, BMA House, Tavistock Square, London WC1H 9JR, United Kingdom.
Dr. Wilson: Department of Health Research Methods, Evidence, and Impact, McMaster University, 1280 Main Street West, Hamilton, Ontario L8S 4K1, Canada.
Dr. Lewin: Norwegian Institute of Public Health, PO Box 4404 Nydalen, N-0403 Oslo, Norway.
Dr. Godfrey: Queen's Collaboration for Health Care Quality: A JBI Centre of Excellence, Queen's University School of Nursing, 992 University Avenue, Barrie Street, Kingston, Ontario K7L 3N6, Canada.
Dr. Macdonald: School of Nursing, Dalhousie University, PO Box 15000, 5869 University Avenue, Halifax, Nova Scotia B3H 4R2, Canada.
Dr. Langlois: Alliance for Health Policy and Systems Research, World Health Organization, Avenue Appia 20, 1211 Geneva, Switzerland.
Dr. Soares-Weiser: Cochrane Editorial Unit, Cochrane, St. Albans House, 57-59 Haymarket, London SW1Y 4QX, United Kingdom.
Ms. Moriarty: Social Care Workforce Research Unit, King's College London, Strand, London WC2R 2LS, United Kingdom.
Dr. Tunçalp: Department of Reproductive Health and Research, World Health Organization, Avenue Appia 20, 1211 Geneva, Switzerland.

Author Contributions: Conception and design: A.C. Tricco, W. Zarin, K.K. O'Brien, D. Moher, M.D.J. Peters, L. Stewart, S.E. Straus.
Analysis and interpretation of the data: A.C. Tricco, E. Lillie, W. Zarin, K.K. O'Brien, H. Colquhoun, D. Moher, M.D.J. Peters, T. Horsley, S. Hempel, E.A. Akl, C. Chang, J. McGowan, L. Stewart, L. Hartling, A. Aldcroft, M.G. Wilson, C. Garritty, S. Lewin, C.M. Godfrey, M.T. Macdonald, K. Soares-Weiser, Ö. Tunçalp, S.E. Straus.
Drafting of the article: A.C. Tricco, E. Lillie, W. Zarin, K.K. O'Brien, D. Levac, D. Moher, M.D.J. Peters, T. Horsley, C. Chang, J. McGowan, A. Aldcroft, C. Garritty.
Critical revision of the article for important intellectual content: A.C. Tricco, E. Lillie, W. Zarin, K.K. O'Brien, D. Moher, M.D.J. Peters, T. Horsley, L. Weeks, S. Hempel, E.A. Akl, J. McGowan, L. Stewart, L. Hartling, A. Aldcroft, M.G. Wilson, C. Garritty, S. Lewin, C.M. Godfrey, E.V. Langlois, T. Clifford, Ö. Tunçalp, S.E. Straus.
Final approval of the article: A.C. Tricco, E. Lillie, W. Zarin, K.K. O'Brien, H. Colquhoun, D. Levac, D. Moher, M.D.J. Peters, T. Horsley, L. Weeks, S. Hempel, E.A. Akl, C. Chang, J. McGowan, L. Stewart, L. Hartling, A. Aldcroft, M.G. Wilson, C. Garritty, S. Lewin, C.M. Godfrey, M.T. Macdonald, E.V. Langlois, K. Soares-Weiser, J. Moriarty, T. Clifford, Ö. Tunçalp, S.E. Straus.
Obtaining of funding: A.C. Tricco, K.K. O'Brien, S.E. Straus.
Administrative, technical, or logistic support: E. Lillie, W. Zarin, S.E. Straus.
Collection and assembly of data: E. Lillie, W. Zarin, K.K. O'Brien, H. Colquhoun, T. Horsley, L. Weeks, S. Hempel, E.A. Akl, L. Hartling, A. Aldcroft, C. Garritty, M.T. Macdonald, K. Soares-Weiser, T. Clifford, Ö. Tunçalp.

Appendix: PRISMA Extension for Scoping Reviews (PRISMA-ScR): Explanation and Elaboration

This explanation and elaboration document presents key examples from the literature with explanations for best practices for reporting each PRISMA-ScR item.

Title and Abstract

Item 1: Title

Identify the report as a scoping review.

Example

Screening of cognitive impairment in the dialysis population: a scoping review. (31)

Explanation and Elaboration. To easily identify the article as a scoping review, the title should include the term "scoping review." The JBI guidance states that the title should reflect the key elements that inform the eligibility criteria of the scoping review, such as the population, concept, and context (4, 5).

For peer review only - http://bmjopen.bmj.com/site/about/guidelines.xhtml

Item 2: Structured Summary

Provide a structured summary that includes (as applicable) background, objectives, eligibility criteria, sources of evidence, charting methods, results, and conclusions that relate to the review questions and objectives.

Example

Background. Among circumpolar populations, recent research has documented a significant increase in risk factors which are commonly associated with chronic disease, notably obesity.

Objective. The present study undertakes a scoping review of research on obesity in the circumpolar Inuit to determine the extent obesity research has been undertaken, how well all subpopulations and geographic areas are represented, the methodologies used and whether they are sufficient in describing risk factors, and the prevalence and health outcomes associated with obesity.

Design. Online databases were used to identify papers published 1992–2011, from which we selected 38 publications from Canada, the United States, and Greenland that used obesity as a primary or secondary outcome variable in 30 or more non-pregnant Inuit . . . participants aged 2 years or older.

Results. The majority of publications (92%) reported cross-sectional studies while 8% examined retrospective cohorts. All but one of the studies collected measured data. Overall 84% of the publications examined obesity in adults. Those examining obesity in children focused on early childhood or adolescence. While most (66%) reported 1 or more anthropometric indices, none incorporated direct measures of adiposity. Evaluated using a customized quality assessment instrument, 26% of studies achieved an "A" quality ranking, while 18 and 39% achieved quality rankings of "B" and "C", respectively.

Conclusions. While the quality of studies is generally high, research on obesity among Inuit would benefit from careful selection of methods and reference standards, direct measures of adiposity in adults and children, studies of preadolescent children, and prospective cohort studies linking early childhood exposures with obesity outcomes throughout childhood and adolescence. (32)

Explanation and Elaboration. The structured summary should concisely describe the aims, methods, findings, and conclusions of the scoping review so that they can be easily identified by knowledge users (such as policymakers, health care providers, health care managers, and patients or consumers), funding agencies, and researchers (33–35). The summary will be the only information available to some readers, who will rely on it to decide whether to read the full text, so details should be clearly reported. It is also useful for the purposes of literature searching retrieval (33–35).

The summary elements are listed "as applicable" to indicate that authors should include only those that are relevant to their scoping review and the journal requirements, such as the background or context of the issue under study, objectives or purpose of the review, eligibility criteria, selection process for sources of evidence, eligible sources of evidence, data charting methods, main findings or results, and conclusions as they relate to the review questions and objectives. Where applicable, authors should include information about other elements that are not listed (such as funding and registration number). In contrast with the original PRISMA structured summary item, "synthesis methods" has been replaced with "charting methods" (the more appropriate terminology and approach for scoping reviews) and "limitations" is omitted. Although the limitations associated with the conduct of the scoping review should be outlined, risk of bias and methodological quality are generally not appraised.

Introduction

Item 3: Rationale

Describe the rationale for the review in the context of what is already known. Explain why the review questions or objectives lend themselves to a scoping review approach.

Example

The support of the social environment is equally important: parents, peers, teachers, community-members, and friends. Parents, in particular, greatly influence participation at school, at home and in the community. They undertake many actions to improve their children's participation in daily life. Understanding the actions of parents and also their challenges and needs will contribute to how society can support these parents and thereby enable the participation of children with physical disabilities. Pediatric rehabilitation, aiming for optimal participation, could benefit from this understanding to improve Family-centered services (FCS). In FCS, the family is seen as an expert on the child's abilities and needs, and professionals work in partnership with the family. Pediatric rehabilitation considers FCS as a way to increase participation of children with a physical disability in daily life.

However, it is unclear what kind of information is available in literature about what parents live through, do, and what kind of problems and needs they have in supporting their child's participation? For these reasons, a scoping review was conducted in order to systematically map the research done in this area, as well as to identify any existing gaps in knowledge. (36)

For peer review only - http://bmjopen.bmj.com/site/about/guidelines.xhtml

Explanation and Elaboration. The background of a scoping review should be comprehensive and cover the main topic elements, important definitions, and existing knowledge in the field (4, 5). When reporting the rationale, researchers should situate their work appropriately (that is, in the context of what is already known on the topic or research question) and clearly explain why they chose the scoping review method given the many types of knowledge synthesis available (11).

Item 4: Objectives

Provide an explicit statement of the questions and objectives being addressed with reference to their key elements (for example, population or participants, concepts, and context) or other relevant key elements used to conceptualize the review questions or objectives.

Example

. . . a scoping review was conducted in order to systematically map the research done in this area, as well as to identify any existing gaps in knowledge. . . . The following research question was formulated: What is known from the literature about parents' action, challenges, and needs while enabling participation of their children with a physical disability? (36)

Explanation and Elaboration. Authors should include a clear and explicit statement of the overall objectives and research questions that they will address in their scoping review. These should be articulated in terms of the key elements, which relate to the review's eligibility criteria. The language of this item ("other relevant key elements used to conceptualize the review questions . . .") aims to be inclusive of the many approaches authors can use to develop research questions, including (but not limited to) the PICO (population, intervention, comparison, outcome) (37), SPICE (setting, population/perspective, intervention, comparison, evaluation) (38), or PCC (population, concept, context) (5, 39) frameworks. Regardless of how the objectives and questions were conceptualized, the main components guiding the inquiry should be clearly stated (6, 7).

Methods

Item 5: Protocol and Registration

Indicate whether a review protocol exists; state if and where it can be accessed (for example, a Web address); and if available, provide registration information, including the registration number.

Example

Our protocol was drafted using the Preferred Reporting Items for Systematic Reviews and Meta-analysis Protocols (PRISMA-P . . .), which was revised by the research team and members of Health Canada, and was disseminated through our programme's Twitter account (@KT-Canada) and newsletter to solicit additional feedback. The final protocol was registered prospectively with the Open Science Framework on 6 September 2016 (https://osf.io/kv9hu/). (40)

Explanation and Elaboration. A protocol should be developed a priori, and it is important to include information about the protocol in the scoping review (4, 5). To ensure transparency and reduce duplication of work, the protocol should ideally be registered (for example, with the Open Science Framework [41]), and authors may wish to publish it in a journal (such as Systematic Reviews [42], JBI Database of Systematic Reviews and Implementation Reports [43], or BMJ Open [44]). If the protocol is not publicly available, details about how to access it (for example, on request from the corresponding author) should be provided. If the scoping review is an update of an existing review, the original scoping review should be cited.

Item 6: Eligibility Criteria

Specify characteristics of the sources of evidence used as eligibility criteria (for example, years considered, language, and publication status), and provide a rationale.

Example

. . . to be included in the review, papers needed to measure or focus on specific dimensions of treatment burden, developed in the conceptual framework (e.g. financial, medication, administrative, lifestyle, healthcare and time/travel). Peer-reviewed journal papers were included if they were: published between the period of 2000–2016, written in English, involved human participants and described a measure for burden of treatment, e.g. including single measurements, measuring and/or incorporating one or two dimensions of burden of treatment. Quantitative, qualitative and mixed-method studies were included in order to consider different aspects of measuring treatment burden. Papers were excluded if they did not fit into the conceptual framework of the study, focused on a communicable chronic condition, for example human immunodeficiency virus infection and acquired immune deficiency syndrome (HIV/AIDS) or substance abuse. Papers talking about carer burden, in addition to patient burden of treatment, were also included. (45)

Explanation and Elaboration. Inclusion criteria should be provided to allow the reader to understand the types of evidence sources that will be included in the review. The rationale for each inclusion criterion should be clearly described. If limitations were in place

For peer review only - http://bmjopen.bmj.com/site/about/guidelines.xhtml

by year, language, publication status, or other characteristics, the authors should specify these and provide a rationale for each.

Item 7: Information Sources

Describe all information sources in the search (for example, databases with dates of coverage and contact with authors to identify additional sources), as well as the date the most recent search was executed.

Example

To identify potentially relevant documents, the following bibliographic databases were searched from 2004 to June 2015: MEDLINE, EMBASE, LexisNexis Academic, the Legal Scholarship Network, Justis, LegalTrac, QuickLaw, and HeinOnline. The search strategies were drafted by an experienced librarian [name] and further refined through team discussion. The final search strategy for MEDLINE can be found in Additional file 3. The final search results were exported into EndNote, and duplicates were removed by a library technician. The electronic database search was supplemented by searching the Canadian Medical Protective Association website (https://www.cmpa-acpm.ca/en) and scanning relevant reviews. (46)

Explanation and Elaboration. A comprehensive literature search should be done for a scoping review, and it may include both published and difficult to locate or unpublished (sometimes called "gray") literature (4, 5). Not all scoping reviews will include gray literature (depending on the specific research question and objectives), but if done, the information sources should be reported. The date of the most recent literature search is important to include because it allows the reader to judge how current the scoping review is. Details should be provided if the search was supplemented through various approaches, such as contacting authors to identify additional relevant material, hand-searching key journals, and scanning reference lists of included or relevant sources of evidence.

Item 8: Search

Present the full electronic search strategy for at least 1 database, including any limits used, such that it could be repeated.

Example

The final search strategy for MEDLINE can be found in Additional file 3. . . .

. . . Medline Search Strategy (Literature Search performed: June 15, 2015)

1. Obstetrics/
2. "Obstetrics and Gynecology Department, Hospital"/
3. exp Obstetric Surgical Procedures/
4. obstetric$.tw,hw.
5. exp Obstetric Labor Complications/
6. exp "Dilatation and Curettage"/
7. exp Hysterectomy/
8. Sterilization, Tubal/
9. Salpingostomy/
10. exp Pregnancy Complications/
11. cerebral palsy/
12. Asphyxia Neonatorum/
13. (abortion$ or cervical cerclage or colpotomy or culdoscop$ or fetoscop$ or hysteroscop$ or hysterotomy).tw.
14. (paracervical block$ or obstetric$ anesthe$ or obstetric$ anaesthe$).tw.
15. (Cesarean or Episiotom$ or obstetric$ abstraction$ or fetal version).tw.
16. ((induc$ or augmentation or premature or preterm or preterm or obstructed) adj (labour or labor)).tw.
17. (Abruptio Placentae or breech or Cephalopelvic Disproportion or premature rupture of fetal membrane$ or prom or fetal membranes premature rupture or Dystocia or Uterine Inertia or Chorioamnionitis or Placenta Accreta or Placenta Previa or Postpartum Hemorrhage or Uterine Inversion or Uterine Rupture or Vasa Previa).tw.
18. (Fetal Death or Fetal Resorption or Stillbirth or perinatal death or peri-natal death or Maternal Death or Birth Injuri$ or obstetric$ paralys$).tw.
19. (pre-eclampsia or dilatation or Curettage or Vacuum aspiration).tw.
20. (asphyxia neonatorum or cerebral palsy or birth asphyxia or fetal pulmonary embolism or dystocia).tw.
21. exp Dystocia/ or exp Pregnancy Complications, Cardiovascular/
22. or/1-21
23. exp Medical Errors/
24. ae.fs.
25. (error$ or advers$ or mistake$ or negligence).tw.
26. or/23-25
27. 22 and 26
28. exp Malpractice/
29. Expert Testimony/
30. (reforms or tort reform$ or damage award limit$ or lawsuit$ or immunity provision$).tw.
31. (immunity provision$ or immunity clause$ or fault compensation or Malpractice or expert witness$).tw.
32. (statutes adj2 limitations).tw.
33. lj.fs.
34. exp Jurisprudence/
35. or/28-34
36. 27 and 35
37. Limit 36 to yr = 2004-current
38. Limit 37 to English (46)

For peer review only

Explanation and Elaboration. The literature search strategy should be reported in a manner that allows easy replication by others and should be presented in its entirety in the text, a table, or an appendix. Additional details to report include the person who did the literature search (for example, an experienced librarian or information specialist) and whether it was peer-reviewed by another librarian using the Peer Review of Electronic Search Strategies (PRESS) checklist, a set of recommendations for librarians and other information specialists to use when evaluating electronic search strategies (47). The full search strategy should be provided for at least 1 electronic database, and if gray literature was searched as part of the scoping review, a detailed account of the approach should be documented. For example, "Grey Matters: a practical tool for searching health-related grey literature" (created by the Canadian Agency for Drugs and Technologies in Health) outlines an approach for searching gray literature (48). Any search limitations, such as language, date of publication, and study design filters, should be clearly documented with a rationale provided.

Item 9: Selection of Sources of Evidence

State the process for selecting sources of evidence (that is, screening and eligibility) included in the scoping review.

Example

To increase consistency among reviewers, all reviewers screened the same 50 publications, discussed the results and amended the screening and data extraction manual before beginning screening for this review. Nine reviewers working in pairs sequentially evaluated the titles, abstracts and then full text of all publications identified by our searches for potentially relevant publications. . . . We resolved disagreements on study selection and data extraction by consensus and discussion with other reviewers if needed. (49)

Explanation and Elaboration. A narrative description of the selection process for included sources of evidence should be provided. When reporting this item, authors should include information about the process for developing the form that was used to guide the selection of sources of evidence (that is, how the items were selected and which software was used), calibration exercises or pilot-testing (testing the form among some or all team members to refine it and ensure that all relevant data were captured), full screening process (how many reviewers participated and whether they screened independently and compared answers or 1 or more researchers screened and 1 or more researchers verified the screening for accuracy), and how inconsistencies or disagreements were resolved (for example, by involvement of a third party). Calibration exercise details should include the number of persons who tested the form (using x number of citations and full-text articles), the process for resolving inconsistencies, and key changes that were made and why. The **Box** shows further details on the calibration exercise. If applicable, all processes for obtaining and confirming data from investigators should be described.

Item 10: Data Charting Process

Describe the methods of charting data from the included sources of evidence (for example, calibrated forms or forms that have been tested by the team before their use, and whether data charting was done independently or in duplicate) and any processes for obtaining and confirming data from investigators.

Examples

A data-charting form was jointly developed by two reviewers to determine which variables to extract. The two reviewers independently charted the data, discussed the results and continuously updated the data-charting form in an iterative process. (50)

Data from eligible studies were [charted] using a standardized data abstraction tool designed for this study. The tool captured the relevant information on key study characteristics and detailed information on all metrics used to estimate/describe [child] growth based on at least two data points per child/group (even though our tool can accommodate metrics based on cross-sectional analyses) anywhere in the article, including metrics that were mentioned in the narrative yet for which results were not shown. . . .

Two reviewers independently [charted] data from each eligible article. Any disagreements were resolved through discussion between the two reviewers or further adjudication by a third reviewer. Data [charting] was implemented using REDCap, a customizable informatics systems-based web software. (51)

Explanation and Elaboration. In the frameworks by Arksey and O'Malley (6) and Levac and colleagues (7), as well as the JBI guidance (4, 5), the process of data extraction in a scoping review is called "data charting" and involves the use of a clear and comprehensive data charting form to extract the relevant information from the included sources of evidence. When reporting this step, authors should include information about the process for developing the charting form (that is, how the items were selected and which software was used), calibration (testing the form among some or all team members to refine it and ensure that all relevant data

For peer review only - http://bmjopen.bmj.com/site/about/guidelines.xhtml

Box. Implementation of calibration exercises (items 9 and 10).

To ensure systematic and reproducible study selection, data charting, and (optional) critical appraisal processes, it is recommended that scoping review authors start with calibration exercise(s) using standardized forms, accompanied by explanation and elaboration documents, to establish agreement among the team.

When multiple reviewers with varying knowledge and experience with scoping reviews are involved with the study selection, data charting, or optional critical appraisal steps, the potential for errors and interrater disagreements can be substantial. To prevent errors and ensure high interrater agreement, it is recommended to start each of the aforementioned steps with a calibration exercise (also known as a pilot test).

To begin, a standardized form, along with an explanation and elaboration document, is developed for each of the study selection, data charting, and (if applicable) critical appraisal steps. If desired, the forms can be tested internally by the review leads using a seminal paper to assess their appropriateness and comprehensiveness prior to the calibration exercise with the full review team. When ready, the form is tested by all members of the review team. For example, during the study selection phase, a calibration exercise can be carried out using a random sample of 50–100 citations for title and abstract screening. The entire team will screen these citations. The discrepancies between the reviewers on the team are calculated, and a roundtable discussion is held to clarify any issues. Refinements to the standardized form and the accompanying explanation and elaboration document can be made as needed. A second calibration exercise may be done if a predetermined level of agreement (typically 70%–80%) is not reached or if reviewers express the need for more training. Once satisfactory interrater agreement is reached, reviewers can move on to screening either in duplicate (i.e., 2 reviewers independently) or following any other variations (e.g., 1 reviewer and 1 verifier), as specified in the scoping review protocol. A similar approach can be used for the second phase of study selection (i.e., full-text screening), data charting, and (if applicable) critical appraisal processes.

were captured), full data charting process (how many reviewers participated and whether they charted independently and compared answers or 1 or more researchers charted and 1 or more researchers verified the data for accuracy), and how inconsistencies or disagreements were resolved (for example, through discussion or involvement of a third party). Calibration exercise details should include the number of persons who tested the form (using x number of included sources), as well as the process for resolving inconsistencies, and key changes that were made and why (Box). If the charting process was iterative (that is, the form was continually updated), authors should describe the main revisions with a rationale, to increase transparency of reporting. If applicable, the processes used for obtaining and confirming data from investigators of the included sources of evidence should be described.

Item 11: Data Items

List and define all variables for which data were sought and any assumptions and simplifications made.

Example

We abstracted data on article characteristics (e.g., country of origin, funder), engagement characteristics and contextual factors (e.g., type of knowledge user, country income level, type of engagement activity, frequency and intensity of engagement, use of a framework to inform the intervention), barriers and facilitators to engagement, and results of any formal assessment of engagement (e.g., attitudes, beliefs, knowledge, benefits, unintended consequences). (52)

Explanation and Elaboration. The specific data items (whether qualitative or quantitative) collected for the scoping review will vary according to the review's focus. If any items involve interpretation, this should be reported. The final version of the charting form, including clear definitions of each item, should be included (if possible) in the scoping review as an appendix or supplementary file.

Item 12 (Optional): Critical Appraisal of Individual Sources of Evidence

If done, provide a rationale for conducting a critical appraisal of included sources of evidence; describe the methods used and how this information was used in any data synthesis (if appropriate).

Example

. . . an in-depth assessment of the conduct of the knowledge synthesis approaches underlying the NMA [network meta-analysis] is lacking. As such, we aimed to explore the characteristics and methodological quality of knowledge synthesis approaches of NMAs. We also aimed to assess the statistical methods applied using the Analysis subdomain of the ISPOR checklist. . . .

The quality of the knowledge synthesis methods was appraised using the AMSTAR tool. The AMSTAR tool was created and validated to assess the methodological quality of systematic reviews of RCTs. The tool measures overall quality, where a score of 8 or higher is considered high quality, 4 to 7 is moderate quality, and 0 to 3 is low quality. Information for quality assessment was incorporated into the data extraction form, which was pilot-tested on a random sample of seven included articles that ranged from low to high quality.

To appraise the validity of the analytical methods applied, we used the 6-item Analysis subdomain of the ISPOR checklist for NMAs. To ensure high inter-rater agreement, a workshop on the tool was held with the team and two pilot-tests were conducted on a random sample of seven included NMAs. Each pilot-test consisted of a facilitated team meeting for feedback and discussion on discrepant items. Upon completion of the pilot-tests, pairs of reviewers (A.A.V., W.Z., J.A., S.S., P.R., C.D., J.E.) independently assessed the first 215 included articles. The remaining 241 included articles were assessed by one reviewer (M.P.) and verified by a second reviewer (A.V., S.S.). All discrepancies were resolved by a third reviewer (W.Z., A.A.V.). ISPOR items that were not applicable to open loop networks (related terms include without a closed-loop, star-shaped network, and tree-shaped network) were scored as "not

For peer review only - http://bmjopen.bmj.com/site/about/guidelines.xhtml

applicable." Items related to heterogeneity were also not applicable to NMAs that used a fixed-effect model and provided a rationale for selecting this model. (53)

Explanation and Elaboration. For both this item and item 19, we adopted the term "critical appraisal" (instead of "risk of bias") to include and acknowledge the various sources of evidence that may be used in a scoping review (such as quantitative and qualitative research, policy documents, and expert opinions). A key difference between scoping reviews and systematic reviews is that the former are generally conducted to provide an overview of the existing evidence regardless of methodological quality or risk of bias (4, 5). Therefore, the included sources of evidence are typically not critically appraised for scoping reviews. When individual sources of evidence are assessed for methodological quality or risk of bias, authors must provide a clear explanation of how the appraisal aligns with the review objectives (that is, a rationale), along with a description of the methodological approach (such as tools used and process followed, including number of reviewers, calibration, and so forth) and how the findings were used. This is an optional step, so authors are expected to report the rationale and methods *only if* an appraisal was done.

Item 13 (Not Applicable): Summary Measures

This item from the original PRISMA is not applicable for scoping reviews because a meta-analysis is not done (that is, summary measures are not relevant).

Item 14: Synthesis of Results

Describe the methods of handling and summarizing the data that were charted.

Example

We grouped the studies by the types of behavior they analyzed, and summarized the type of settings, populations and study designs for each group, along with the measures used and broad findings. Where we identified a systematic review, we counted the number of studies included in the review that potentially met our inclusion criteria and noted how many studies had been missed by our search. (54)

Explanation and Elaboration. The aim of the synthesis is to present the range of evidence that was identified to answer the review question or meet the objectives of the scoping review (4, 5). Authors should clearly describe how the evidence will be presented: in a narrative format, table, or visual representation, including a map or diagram.

Item 15 (Not Applicable): Risk of Bias Across Studies

This item from the original PRISMA is not applicable for scoping reviews because the scoping review method is not intended to be used to critically appraise (or appraise the risk of bias of) a cumulative body of evidence.

Item 16 (Not Applicable): Additional Analyses

This item from the original PRISMA is not applicable for scoping reviews because additional analyses, including sensitivity or subgroup analyses and meta-regression, are not done.

Results

Item 17: Selection of Sources of Evidence

Give numbers of sources of evidence screened, assessed for eligibility, and included in the review, with reasons for exclusions at each stage, ideally using a flow diagram.

Example

After duplicates were removed, a total of 883 citations were identified from searches of electronic databases and review article references. Based on the title and the abstract, 699 were excluded, with 184 full text articles to be retrieved and assessed for eligibility. Of these, 144 were excluded for the following reasons: 23 did not directly quantify the effects of climate change, 53 did not directly quantify effects on human health, and 67 were not considered to be original quantitative research (e.g., review articles, commentaries). We excluded 1 study because we were unable to retrieve it. The remaining 40 studies were considered eligible for this review. (55)

Explanation and Elaboration. The results of the literature search should be reported, including numbers of citations screened, duplicates removed, and full-text documents screened. Consistent with the original PRISMA statement (10), we recommend including a flow diagram that details the reasons for exclusion at the full-text level of screening at a minimum (**Appendix Figure 1**).

Item 18: Characteristics of Sources of Evidence

For each source of evidence, present characteristics for which data were charted and provide the citations.

Example

The modules [of the e-recovery interventions] are described in Table 2, together with a description of aim, target group and setting for each intervention. . . .

The studies' place of origin, aims, design, methods, measures and outcomes, and main findings related to

For peer review only - http://bmjopen.bmj.com/site/about/guidelines.xhtml

each intervention are presented in Table 3 Study characteristics. The number of studies available per interventions varied from one to six. (56)

Explanation and Elaboration. The characteristics of interest of each source of evidence should be presented, along with their references. An overall summary can be reported in the text, with characteristics for the individual sources of evidence provided in tables and appendixes, as appropriate.

Item 19 (Optional): Critical Appraisal Within Sources of Evidence

If done, present data on critical appraisal of included sources of evidence (see item 12).

Example

See **Appendix Figure 2** (53).

Explanation and Elaboration. As explained in item 12, although critical appraisal of individual sources of evidence falls outside the realm of scoping review methodology (4, 5), it may be done if relevant to the scoping review objectives. If done, authors must report the data (that is, the critical appraisal findings) for each included source of evidence in a manner that corresponds to the approach described in the methods (see item 12). Because this step is optional, authors are expected to report the results *only if* an appraisal was done.

Item 20: Results of Individual Sources of Evidence

For each included source of evidence, present the relevant data that were charted that relate to the review questions and objectives.

Example

See **Appendix Figure 3** (57).

Explanation and Elaboration. As noted in item 11, the specific data items that were charted will vary according to the specific questions and objectives of the scoping review and may not necessarily include the outcome results of the included sources of evidence. Depending on the number of sources included in the scoping review, the relevant data from each source can be provided in an appendix or supplementary file.

Item 21: Synthesis of Results

Summarize or present the charting results as they relate to the review questions and objectives.

Example

Active Travel and Physical Activity

Ninety-two studies examined associations between active travel and physical activity [references]. The majority were from the UK (*n* = 24) and USA (*n* = 19), followed by Australia (*n* = 12), Canada (*n* = 7), Denmark (*n* = 6) and New Zealand (*n* = 5). Other countries with less than five studies included: Norway, Netherlands, Belgium, Switzerland, Spain, Portugal, Estonia, Germany, Sweden, and Ireland. The majority were conducted among children (70%, *n* = 64), including 8 studies that included only children under 10 years old. Only two studies reported analyses of only adults over 65 [references].

The vast majority of studies used cross-sectional analyses—only six studies out of 92 (6.5%) reported results from longitudinal or pre/post analysis to examine associations between active travel and physical activity [references]. Just over half of the studies (*n* = 48) used objective measures (e.g., accelerometer, pedometer) to assess physical activity.

Overall, most studies (*n* = 72; 78%, representing 75% of the children's and 86% of the adults' studies) reported a positive association between active travel and physical activity; however many of these (*n* = 32) reported mixed results overall (e.g., when using more than one measure of physical activity, or in subanalyses such as for gender). Of the 20 studies that reported no association, 12 used objective measures to assess physical activity. The average numbers of participants in these 20 studies were much lower than in studies which did report an association. This may be indicative of insufficient power to find associations. (54)

Explanation and Elaboration. Results may be presented as a "map" of the data in the form of a diagram or table (**Appendix Figure 4**) or in a descriptive format, whichever aligns best with the review's objectives (4, 5).

Item 22 (Not Applicable): Risk of Bias Across Studies

This item is not applicable for scoping reviews. See explanation for item 15.

Item 23 (Not Applicable): Additional Analyses

This item is not applicable for scoping reviews. See explanation for item 16.

Discussion and Funding

Item 24: Summary of Evidence

Summarize the main results (including an overview of concepts, themes, and types of evidence available), link to the review questions and objectives, and consider the relevance to key groups.

For peer review only - http://bmjopen.bmj.com/site/about/guidelines.xhtml

Example

In this scoping review we identified 88 primary studies addressing dissemination and implementation research across various settings of dementia care published between 1998 and 2015. Our findings indicate a paucity of research focusing specifically on dissemination of knowledge within dementia care and a limited number of studies on implementation in this area. We also found that training and educating professionals, developing stakeholder interrelationships, and using evaluative and iterative strategies are frequently employed to introduce and promote change in practice. However, although important and feasible, these strategies only partly address what is repeatedly highlighted in the evidence base: that organisational factors are reported as the main barrier to implementation of knowledge within dementia care. Moreover, included studies clearly support an increased effort to improve the quality of dementia care provided in residential settings in the last decade. (26)

Explanation and Elaboration. The main findings should be summarized and linked to the review questions and objectives (6, 7). The data charting results can be elaborated for or tailored to each relevant knowledge user group, such as policymakers, health care providers, and patients or consumers.

Item 25: Limitations

Discuss the limitations of the scoping review process.

Example

Our scoping review has some limitations. To make our review more feasible, we were only able to include a random sample of rapid reviews from websites of rapid review producers. Further adding to this issue is that many rapid reviews contain proprietary information and are not publicly available. As such, our results are only likely generalizable to rapid reviews that are publicly available. Furthermore, this scoping review was an enormous undertaking and our results are only up to date as of May 2013. (58)

Explanation and Elaboration. Because a critical appraisal is optional for scoping reviews, reporting of this item should focus on limitations of the scoping review process (vs. limitations of the included sources of evidence). Any deviations from guidance (for example, the JBI methods guidance [4, 5]) or the scoping review protocol should be noted, along with a rationale and a reflection on the potential effect on the results.

Item 26: Conclusions

Provide a general interpretation of the results with respect to the review questions and objectives, as well as potential implications or next steps.

Example

The lack of evidence to support physiotherapy interventions for this population appears to pose a challenge to physiotherapists. The aim of this scoping review was to identify gaps in the literature which may guide a future systematic review. However, the lack of evidence found means that undertaking a systematic review is not appropriate or necessary.

Evidence is insufficient to guide the nature of the physiotherapy intervention. There is also limited evidence to describe the experiences of patients, next of kin, or physiotherapists working with this population. The consideration of the attitudes towards an intervention could be considered a vital component of a complex intervention and it is suggested that they should be an integral part of the implementation of that intervention. This advocates high quality research being needed to determine what physiotherapy techniques may be of benefit for this population and to help guide physiotherapists as how to deliver this. (59)

Explanation and Elaboration. The charting results should be discussed in relation to current literature, practice, and policy (4, 5). The potential implications of the scoping review should be discussed. Recommendations for future research, including a more focused systematic review that builds from the scoping review results, can be mentioned if appropriate. Most scoping reviews aim to summarize what has been done previously and carry out data charting and do not perform a formal appraisal or synthesis (1). As such, recommendations for practice and policy will not be relevant for most scoping reviews (4, 5). The interpretation of results should link to the review questions and objectives, as initially specified.

Item 27: Funding

Describe sources of funding for the included sources of evidence, as well as sources of funding for the scoping review. Describe the role of the funders of the scoping review.

Example

This paper was funded by Stichting Innovatie Alliantie (PRO-3-36) (http://www.regieorgaan-sia.nl) and Zuyd University of Applied Sciences. The funders had no role in study design, data collection and analysis, decision to publish, or preparation of the manuscript. (50)

For peer review only - http://bmjopen.bmj.com/site/about/guidelines.xhtml

Explanation and Elaboration. The source of funding for included sources of evidence should be documented, along with the source of funding for the scoping review. In addition, the role of the scoping review funding organization should be described. For any type of research, many journals require the contract number for the source of funding, which can be provided, if applicable.

Web-Only References

31. **San A, Hiremagalur B, Muircroft W, Grealish L.** Screening of cognitive impairment in the dialysis population: a scoping review. Dement Geriatr Cogn Disord. 2017;44:182-95. [PMID: 28869959] doi:10.1159/000479679
32. **Galloway T, Blackett H, Chatwood S, Jeppesen C, Kandola K, Linton J, et al.** Obesity studies in the circumpolar Inuit: a scoping review. Int J Circumpolar Health. 2012;71:18698. [PMID: 22765938] doi:10.3402/ijch.v71i0.18698
33. **Beller EM, Glasziou PP, Altman DG, Hopewell S, Bastian H, Chalmers I, et al; PRISMA for Abstracts Group.** PRISMA for Abstracts: reporting systematic reviews in journal and conference abstracts. PLoS Med. 2013;10:e1001419. [PMID: 23585737] doi:10.1371/journal.pmed.1001419
34. **Hopewell S, Clarke M, Moher D, Wager E, Middleton P, Altman DG, et al; CONSORT Group.** CONSORT for reporting randomized controlled trials in journal and conference abstracts: explanation and elaboration. PLoS Med. 2008;5:e20. [PMID: 18215107] doi:10.1371/journal.pmed.0050020
35. **Haynes RB, Mulrow CD, Huth EJ, Altman DG, Gardner MJ.** More informative abstracts revisited. Ann Intern Med. 1990;113:69-76. [PMID: 2190518]
36. **Piškur B, Beurskens AJ, Jongmans MJ, Ketelaar M, Norton M, Frings CA, et al.** Parents' actions, challenges, and needs while enabling participation of children with a physical disability: a scoping review. BMC Pediatr. 2012;12:177. [PMID: 23137074] doi:10.1186/1471-2431-12-177
37. **Richardson WS, Wilson MC, Nishikawa J, Hayward RS.** The well-built clinical question: a key to evidence-based decisions [Editorial]. ACP J Club. 1995;123:A12-3. [PMID: 7582737]
38. **Andrew B.** Clear and present questions: formulating questions for evidence based practice. Library Hi Tech. 2006;24:355-68.
39. **Joanna Briggs Institute.** The Joanna Briggs Institute Reviewers' Manual 2015: Methodology for JBI Scoping Reviews. Adelaide, Australia: Joanna Briggs Institute; 2015. Accessed at http://joannabriggs.org/assets/docs/sumari/Reviewers-Manual_Methodology-for-JBI-Scoping-Reviews_2015_v2.pdf on 3 August 2018.
40. **Tricco AC, Zarin W, Lillie E, Pham B, Straus SE.** Utility of social media and crowd-sourced data for pharmacovigilance: a scoping review protocol. BMJ Open. 2017;7:e013474. [PMID: 28104709] doi:10.1136/bmjopen-2016-013474
41. Open Science Framework. 2011. Accessed at https://osf.io on 10 January 2018.
42. **BioMed Central.** Systematic Reviews. 2018. Accessed at https://systematicreviewsjournal.biomedcentral.com on 10 January 2018.
43. JBI Database of Systematic Reviews and Implementation Reports. 2018. Accessed at http://journals.lww.com/jbisrir/pages/default.aspx on 10 January 2018.
44. BMJ Open. Accessed at http://bmjopen.bmj.com on 1 March 2018.
45. **Sav A, Salehi A, Mair FS, McMillan SS.** Measuring the burden of treatment for chronic disease: implications of a scoping review of the literature. BMC Med Res Methodol. 2017;17:140. [PMID: 28899342] doi:10.1186/s12874-017-0411-8
46. **Cardoso R, Zarin W, Nincic V, Barber SL, Gulmezoglu AM, Wilson C, et al.** Evaluative reports on medical malpractice policies in obstetrics: a rapid scoping review. Syst Rev. 2017;6:181. [PMID: 28874176] doi:10.1186/s13643-017-0569-5
47. **McGowan J, Sampson M, Salzwedel DM, Cogo E, Foerster V, Lefebvre C.** PRESS peer review of electronic search strategies: 2015 guideline statement. J Clin Epidemiol. 2016;75:40-6. [PMID: 27005575] doi:10.1016/j.jclinepi.2016.01.021
48. **Canadian Agency for Drugs and Technologies in Health.** Grey Matters: a practical tool for searching health-related grey literature. 2015. Accessed at https://cadth.ca/resources/finding-evidence/grey-matters on 10 January 2018.
49. **Duffett M, Choong K, Hartling L, Menon K, Thabane L, Cook DJ.** Randomized controlled trials in pediatric critical care: a scoping review. Crit Care. 2013;17:R256. [PMID: 24168782] doi:10.1186/cc13083
50. **Lenzen SA, Daniëls R, van Bokhoven MA, van der Weijden T, Beurskens A.** Disentangling self-management goal setting and action planning: a scoping review. PLoS One. 2017;12:e0188822. [PMID: 29176800] doi:10.1371/journal.pone.0188822
51. **Leung M, Perumal N, Mesfin E, Krishna A, Yang S, Johnson W, et al.** Metrics of early childhood growth in recent epidemiological research: a scoping review. PLoS One. 2018;13:e0194565. [PMID: 29558499] doi:10.1371/journal.pone.0194565
52. **Tricco AC, Zarin W, Rios P, Nincic V, Khan PA, Ghassemi M, et al.** Engaging policy-makers, heath system managers, and policy analysts in the knowledge synthesis process: a scoping review. Implement Sci. 2018;13:31. [PMID: 29433543] doi:10.1186/s13012-018-0717-x
53. **Zarin W, Veroniki AA, Nincic V, Vafaei A, Reynen E, Motiwala SS, et al.** Characteristics and knowledge synthesis approach for 456 network meta-analyses: a scoping review. BMC Med. 2017;15:3. [PMID: 28052774] doi:10.1186/s12916-016-0764-6
54. **Hutchinson J, Prady SL, Smith MA, White PC, Graham HM.** A scoping review of observational studies examining relationships between environmental behaviors and health behaviors. Int J Environ Res Public Health. 2015;12:4833-58. [PMID: 25950651] doi:10.3390/ijerph120504833
55. **Hosking J, Campbell-Lendrum D.** How well does climate change and human health research match the demands of policymakers? A scoping review. Environ Health Perspect. 2012;120:1076-82. [PMID: 22504669] doi:10.1289/ehp.1104093
56. **Strand M, Gammon D, Ruland CM.** Transitions from biomedical to recovery-oriented practices in mental health: a scoping review to explore the role of Internet-based interventions. BMC Health Serv Res. 2017;17:257. [PMID: 28388907] doi:10.1186/s12913-017-2176-5
57. **Constand MK, MacDermid JC, Dal Bello-Haas V, Law M.** Scoping review of patient-centered care approaches in healthcare. BMC Health Serv Res. 2014;14:271. [PMID: 24947822] doi:10.1186/1472-6963-14-271
58. **Tricco AC, Antony J, Zarin W, Strifler L, Ghassemi M, Ivory J, et al.** A scoping review of rapid review methods. BMC Med. 2015;13:224. [PMID: 26377409] doi:10.1186/s12916-015-0465-6
59. **Hall AJ, Lang IA, Endacott R, Hall A, Goodwin VA.** Physiotherapy interventions for people with dementia and a hip fracture—a scoping review of the literature. Physiotherapy. 2017;103:361-8. [PMID: 28843451] doi:10.1016/j.physio.2017.01.001

For peer review only - http://bmjopen.bmj.com/site/about/guidelines.xhtml

Appendix Figure 1. Example of item 17, selection of sources of evidence.

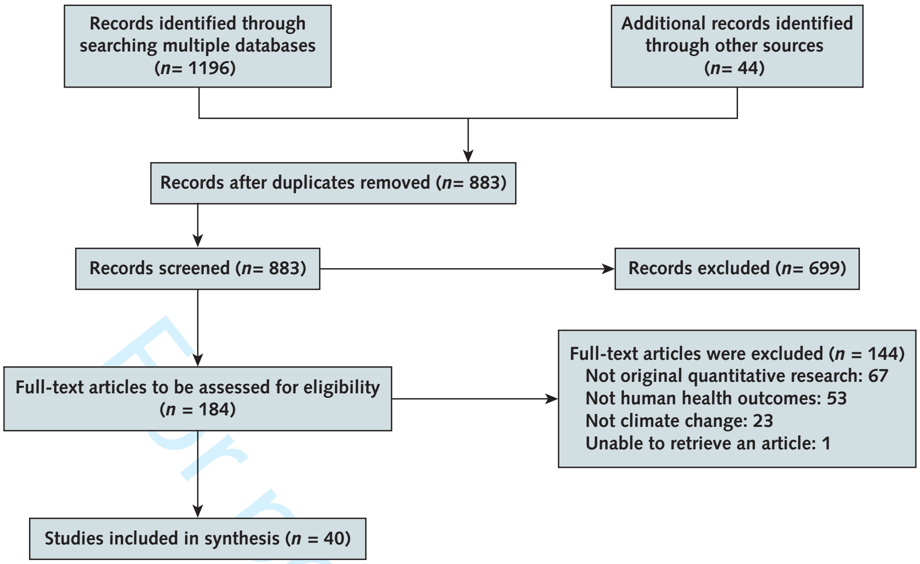

Appendix Figure 2. Example of item 19, critical appraisal within sources of evidence.

First Author	Year	A Priori Design	Duplicate Screening and Data Extraction	Comprehensive Search	Gray Literature	Include/Exclude List	Study Characteristics	Quality Appraisal	Conclusion	Pooling Methods	Publication Bias	Conflict of Interest	AMSTAR Score	Overall Quality
Abdullah	2008	Unclear	Unclear	No	No	No	Yes	Yes	No	Yes	Yes	Yes	5	Moderate
Akshintala	2013	Unclear	Yes	Yes	Unclear	No	Yes	Yes	No	Yes	No	Yes	6	Moderate
Alberton	2012	Unclear	Yes	Yes	Yes	No	Yes	No	No	Yes	No	No	5	Moderate

From the same example presented in item 12. Adapted from reference 53. AMSTAR = A Measurement Tool to Assess Systematic Reviews.

For peer review only

For peer review only - http://bmjopen.bmj.com/site/about/guidelines.xhtml

Appendix Figure 3. Example of item 20, results of individual sources of evidence.

Author	Article Type	Population	Patient-Centered Care Approach Identified
Ballweg [7]	Review article	Neonatal Intensive Care Unit	Developmentally Supportive, Family-Centered Care Model
Berger [8]	Review article	Psychiatry	The Tidal Model
Bickler [9]	Review article	Surgery	Patient-Focused Care Model
Boltz [10]	Review article	Geriatrics	Nurses Improving Care for Health System Elders
Booth & MacBride [11]	Review article	Generic	Patient-Centered Clinical Method
Briggs [12]	Review article	Palliative Care/	National Consensus Project for Quality Palliative Care
		Physical Therapy/	Hypothesis Oriented Algorithm for Clinicians
		End of Life Care	Framework for Rehabilitation of Neurodegenerative Diseases Framework for Assessment in Oncology Rehabilitation Models of Practice in Palliative Care
Browne et al. [13]	Review article	Nursing	Decentralization
Cox [14]	Review article	Psychiatry	Biopsychosocial Model

Scoping review included articles. Adapted from reference 57.

Appendix Figure 4. Example of item 21, synthesis of results.

Reference, First Author, Date	Country National or Regional	Date of Collection, Survey/Recruitment	Analysis	Analyzed, *n*	Sample Age, *y*	Data Collection Method (of Behavior Analyzed Together)*	Environmental Behavior	Health Behavior	Category for this Review
				Active Travel and Physical Activity					
[26] Abbott 2009	Australia Regional	2006 Healthy Kids Queensland (HKQ)	Cross-sectional	2076	Children 6–16	Travel: questionnaire Physical activity: pedometer	Active travel to school	Physical activity: steps	AT/PA
[27] Alexander 2005	United Kingdom Regional	2004 Four schools	Cross-sectional	92	13–14	Transport: questionnaire Physical activity: accelerometer	Active travel to school	Moderate to vigorous physical activity	AT/PA
[28] Anderson 2009	Denmark Regional	1983 Danish Youth and Sports Study	Cross-sectional	1249	15–19	Questionnaire	Active travel to school	Leisure-time physical activity	AT/PA

Description of included studies in the scoping review of bivariate analyses of health and environmental behaviors. Adapted from reference 54. AT/PA = active transportation and physical activity.

For peer review only

For peer review only - http://bmjopen.bmj.com/site/about/guidelines.xhtml

BMJ Open

What does social cognition look like in everyday social functioning in Huntington's disease? - A protocol for a scoping review to explore and synthesise knowledge about social cognition alongside day-to-day social functioning of people with Huntington's disease

Journal:	*BMJ Open*
Manuscript ID	bmjopen-2023-073655.R1
Article Type:	Protocol
Date Submitted by the Author:	20-Jun-2023
Complete List of Authors:	Fisher, Alexandra; University of Birmingham, Institute of Applied Health; Birmingham and Solihull Mental Health NHS Foundation Trust, West Midlands HD Service, Neuropsychiatry<br>Lavis, Anna; University of Birmingham, Institute of Applied Health Research<br>Greenfield, Sheila; University of Birmingham, Institute of Applied Health Research<br>Rickards, Hugh; University of Birmingham College of Medical and Dental Sciences, Clinical & Experimental Medicine; Birmingham and Solihull Mental Health NHS Foundation Trust, West Midlands HD Service, Neuropsychiatry
**Primary Subject Heading**:	Neurology
Secondary Subject Heading:	Research methods
Keywords:	Social Cognition, Social Interaction, NEUROLOGY, Literature, PSYCHIATRY

SCHOLARONE™
Manuscripts

What does social cognition look like in everyday social functioning in Huntington's disease? - A protocol for a scoping review to explore and synthesise knowledge about social cognition alongside day-to-day social functioning of people with Huntington's disease

Corresponding Author:

Alexandra Fisher, email: alf944@bham.ac.uk

Authors

Alexandra Fisher [1,2,3], Anna Lavis [1], Sheila Greenfield [1], Hugh Rickards [1,2,3]

Keywords

Social cognition, social interaction, Huntington's disease, synthesis, literature

Abstract

Introduction

Social cognition is problematic in Huntington's disease (HD). Despite the observations of clinicians and families, there is minimal empirical literature about how it presents in daily life and the impact on social functioning. This protocol forms the basis of a scoping review to synthesize both the quantitative knowledge and qualitative experiences of the HD community so that a visual and narrative map can address what is known and what is not known for the benefit of the community and clinicians and academics alike.

Methods and analyses

An umbrella scoping review of previous work and a scoping review of newer studies of social cognition and social functioning will be undertaken. The electronic databases PubMed, Medline, Psych info, Web of Science, Scopus, Embase and CINAHL will be searched to identify eligible studies from starting from 2003 to June 2023. A grey literature search and grey data search will also be undertaken. Quality appraisal of the included documents will use the Critical Appraisal Skills Programme and AACODS (Authority, Accuracy, Coverage, Objectivity, Date, Significance) checklists. A data charting table will be used for data extraction, with analysis of qualitative data using the framework method. The review findings will be presented in a visual form and in a narrative summary.

Ethics and dissemination

Ethical review is not usually required as scoping reviews are produced via secondary data analysis, however this protocol includes the use of grey data from a charity web forum and so in line with best practice for internet mediated research ethical review was sought and approved (STEM Ethical Review Committee, University of Birmingham – ERN_21-1028A). Review findings will be shared with service users and disseminated through a peer-reviewed publications, conference presentations and hosted via the website of the patient association charity the Huntington's Disease Association (HDA).

Strengths and limitations of this protocol

- This mixed scoping review will amalgamate the Preferred Reporting Items for Systematic Reviews and Meta analyses (PRISMA) extended guidelines for the conduct of scoping reviews as well as the Joanna Briggs Institute (JBI) method for mixed reviews to ensure transparency and rigour.

For peer review only

- Service user and community involvement in the design and conduct of the study which informs this review will ensure that the findings are supported by their experiences, further helping to identify evidence gaps and inform clinical and research practice.
- Grey literature and grey data will be searched to ensure that the review reflects the challenges faced by the HD community not currently captured in the scientific literature. A search strategy has been designed to try and reduce the issues associated with the inherent replicability problems by their inclusion.
- Whilst every effort has been made to create a protocol which is inclusive and comprehensive, there are barriers to this defined by the student authors resources i.e. the exclusion of non-English Language papers

Introduction

Most of our everyday behaviour is motivated by social and emotional goals (1). Social cognition with its underlying structures and processes is how we make sense of that social information.

Social cognition in Huntingtons disease (HD)

Huntington's disease (HD) is an inherited, neurodegenerative disease which impacts communication, cognition, movement, behaviour, and neuropsychiatric function.

HD is presently defined as a movement disorder and its clinical diagnosis in gene positive individuals is made following the confirmation of motor onset. However, problems with social cognition and its component skills are now recognised as manifesting earlier in the disease than motor onset (2, 3).

Social cognitive reviews concerning HD have concluded that the processes thought to underlie social cognition such as emotional recognition (ER) expand from problems in recognition of specific emotional valences in restricted sensory modalities to multiple valences and modalities as the disease progresses (4). Cognitive and affective theory of mind (TOM) are also impacted.

Nonetheless the extent of this latter knowledge comes from studies predominantly based on brain scanning and tests in controlled rather than everyday settings. Highlighting the setting is important because it is in everyday social interaction that the interrelated processes of social cognition and behaviour combine to produce the social skills which are then translated into social functioning which Kennedy & Adolph (5) define as the embedding of the behaviour over time and in context. Social functioning may include "occupation, handling of finances and domestic responsibility which encompasses abilities of performing and understanding social negotiations"(6).

Using this definition as the lens through which we view social functioning in HD, we can see how it contributes to the quality of various activities of daily living in which social interaction is key. Mason (7) highlights in particular shared decision making in HD as crucial to understand given the nature of the illness and the implications for family and friends. Problematic social functioning in HD seems to be present within crises in intimate (8) and family relationships (9), early difficulties in work life (10, 11) and may ultimately lead to institutionalization (12). Furthermore, these challenges have also been observed by family members and clinicians alike (7).

Despite what we know about social cognition and social functioning, the probable relationship between them has not been brought together to form a cohesive picture of what socially related activity looks like in HD. Addressing this disparity through a synthesis of existing knowledge would allow for clinicians to draw on this understanding by supporting people with HD and their families

For peer review only - http://bmjopen.bmj.com/site/about/guidelines.xhtml

through education and tackling these issues collectively with policy makers, for researchers to develop more HD sensitive measures vital to be able to deal with gaps in our knowledge, to develop and target earlier therapeutic interventions - all areas which the HD scientific community (13) (14) and neuroscience community note they need.

This protocol (and the review which will result from it) form part of the corresponding author's ethnographic study of social cognition in HD. Ethnography is a qualitative method set in the everyday community of people's lives. As part of the consultation with the Patient Participation Involvement (PPI) group of the Huntington's Disease Association (HDA) for England and Wales for the ethnographic study, the group reflected the value in knowing more about what social cognition looks like in the everyday. Due to its size, the review will be published in several parts in narrative and tabular form. However, the main outcome for the review will be its accessibility to all parties interested in the results and a visual synthesis with key points will available via the HDA's website.

Why a scoping review?

The motivations for a scoping review are varied (15) but are usually intended to provide an overview and synthesis of a wide body of literature to clarify concepts and identify gaps steering the audience towards more specific questions which then might be addressed in a more focused review or which may be used towards a change in practice or policy (16).

The benefit of scoping reviews in HD seems to have recently taken hold through the synthesis of literature to aid the definition of irritability (17) but also the 'lived experience' of the illness (18) following genetic testing and these were driven by the mismatch in the literature and what is seen in clinical practice.

Similarly and with direct relevance to this protocol, Gibson and Springer's (19) motivations came from clinical curiosity and they produced a scoping review looking at reasons for behaviour behind the person's 'decreased participation in social activities in comparison to previous social engagement' (which they termed social withdrawal), what the outcome of this was and what interventions had been tried. Their results noted that the heterogeneity of studies and differences in concepts under the 'umbrella' of social functioning led to the meanings and outcomes of studies becoming blurred and so they made a number of recommendations which will be factored into this review. They were also keen to stress that whilst there are commonalities in social behaviour in HD, future authors should not dismiss the complexities at an individual level which may be fuelling the withdrawal.

Whilst there are already a number of systematic reviews (7, 20) and a meta-analysis (4) on social cognition and its proposed underlying components in HD (3), these now need updating as new knowledge is available. Furthermore, the authors of these reviews recognised that they had not incorporated the associated qualitative and mixed methods on the multiple domains such as work and home life related to social behaviour, interaction, and functioning which represent the extension of social cognition in HD; and likewise, the grey literature and grey data which may also refer to these experiences. It is the intention of this multiple method scoping review to incorporate the potential for rich detail from these latter sources.

What do we mean by grey literature & grey data?

Grey literature is defined as non-commercially published and unpublished literature from organisations in which publishing is not their primary activity and in the case of health sciences this means that it has not been subject to usual peer review (21).

For peer review only

Grey data refers to user generated information which is web based such as online forums.

What is the added value in incorporating grey literature and data in this area?

As Pope et al (22) state, the value of broadening the scope of synthesis and including diverse evidence does not mean abandoning rigour but rather judging the evidence in context and answering specific questions rather than intrinsically defining all evidence as lower quality than others.

Paez (23) also suggested the benefits of including grey literature in a systematic review reduced the potential for publication bias, identified current trends and like Pope et al considered context which Adams et al (24) recommend should now extend to not only 'written' literature such as journals but into the sphere of digital content.

This latter point by Adams is particularly pertinent in the HD area as whilst the study of social cognition has only relatively recently taken traction, the exploration of everyday social behaviour and functioning in HD has been muted with incongruity between might what appear in the (evidence-based) literature, clinical practice and the discussions in online communities. As Coulson and colleagues (25) who looked at the support offered in a HD online community, noted, "participation in an online support group allows a greater degree of anonymity than face-to-face groups. Such anonymity may facilitate self-disclosure and help individuals in discussing sensitive issues more easily or to give opinions with less fear of embarrassment or judgement than in more traditional face-to-face groups". It is the contention of the authors of this protocol, in line with Coulson's arguments and our own clinical experiences, that given social functioning in HD maybe fraught with tensions and stigma, investigating this grey data will allow access to stories which illuminate this further.

The challenges of using grey literature and data

Whilst there is much about grey literature and grey data to be positive about, caution and strategies are needed to limit the potential for what is termed as scope creep but also transparency to enable a credible replicability vital for the perceived rigour of the review.

Scope creep is a term often used in industry but also recognised in research (26) when a project is ill defined and therefore in practice, those who advocate for the inclusion of grey literature and grey data recommend a structured and constrained search with boundaries formed by the aims of the review. A tight set of criteria is also needed to shore up the credible replicability referred to earlier. Replicability is a term used by Hoffecker (21) who proffers it as an alternative to that of the usual reproducibility aimed for in a standard systematic review, arguing that due to the nature of grey information (particularly grey data) reproducibility is a concept that is almost impossible to capture due to the evolving nature of its source i.e. an internet forum versus a static record within a database.

The aims of this review are:

1) to explore and summarise the literature related to social cognition in HD,

2) to explore and summarise the literature related to social behaviour and functioning in HD

3) to draw together the findings using both a mixture of both visual and narrative synthesis in line with current best methodological practice (27, 28).

Plan

Method

To fulfil the aims of this review, the following methodological approaches will be used:

For peer review only

1) *to explore and summarise the literature related to social cognition* an umbrella scoping review will be undertaken. As there is already a large amount of systematic work which has reviewed quantitative studies of social cognition in HD, for pragmatic reasons an umbrella style scoping review of these will be presented. An umbrella review is the systematic review method for the combination of results from independent systematic reviews. Therefore, as an umbrella scoping review, unlike a standard umbrella review, the aim will be to produce a synthesis of the data to date rather than a methodological critique of their quality. The guidance for the PRISMA protocol for umbrella reviews and Joanna Briggs Umbrella review methodology will be consulted (29).

Any quantitative papers on social cognition which have been published since the dates of the original systematic reviews will be assessed in the style of the umbrella scoping review. To provide consistency with the quantitative studies in the original systematic reviews (and meta-analysis), a limit for inclusion will be the use of a comparator in their design and have used tests of social cognition with HD cohorts.

Mixed methods designs on social cognition will not be subject to this latter exclusion. Search terms and key words for the umbrella scoping review will include those from the individual systematic reviews as well as descriptors relevant to the broader domains of social behaviour and functioning (please refer to Table 1).

2) *to explore and summarise the literature related to social behaviour and functioning in HD*, guidance which includes the using PRISMA extended protocol for scoping reviews (30) plus the Joanna Briggs Institute (JBI) method for mixed reviews (31). Search terms and keywords from the umbrella scoping review will be used.

This section of the review will also include both grey literature and grey data. For grey literature its appraisal will refer to the most recent best practice guidelines (32).

For grey data its use and analysis is considered more challenging (33) and at the current time as there are no specific guidelines, its scope will follow the same cautious approach applied to grey literature. In practical terms this means focussing the search of grey literature/grey data to key source material which will be defined in the inclusion/exclusion criteria. Furthermore, in grey data searches, a key consideration is the ownership of the material and therefore its stewardship (34). Whilst the data is publicly available, there is still the question of its potentially sensitive nature and therefore for ethical reasons and for pragmatic purposes, the data extraction from the source of the grey data will be presented as anonymised and in broad narrative concepts in line with Coulson's use of Cutrona & Suhr's coding system for online bulletin boards. Guidance was also taken from the British Psychological Society's framework on internet mediated research (35) and ethical approval sought and granted through the primary author's University Ethics Review Board (ERN_21-1028A).

Aim 3) *show the organisation of findings using both a mixture of both visual and narrative synthesis in line with current best methodological practice.* See analysis and synthesis plan below.

Table 1: Search terms and key words
Huntington* AND<br>Social cogniti* / Social function / Social behaviour / Social interaction / Social judgement / Social psychology / Social understanding / Social skills / Social competence / Social communication / Social decision making<br>Social withdrawal / avoidance / disengage<br>Relationships / interpersonal relationships

For peer review only

Social networks / social connections<br>Work<br>Sex*<br>Non-verbal communication<br>Body language<br>Facial expression<br>Recognition<br>Theory of mind (TOM) / simulation theory<br>Empath* / empath* communication<br>Mentalizing / mental state<br>Emotion*<br>Inference / Intuition<br>Function / Skills

Information sources

Search strategy

The searches will be conducted in 5 stages (please see supplementary files for full strategy)

1) The umbrella scoping review of pre-existing systematic reviews starting with Elamin (20)
2) A further (non-umbrella) scoping review of quantitative and mixed methods literature on social cognition, social behaviour, and social functioning to the current date. The timeline for the search will run from the first paper annotated in Elamin's review (2003) up to the current date (June 2023). The databases to be used will be Pubmed, Medline, Psychinfo, Web of Science, Scopus, Embase and Cinahl.
3) A scoping review of the qualitative literature concerning social cognition, social behaviour and social functioning. This will match the dates of the pre-existing systematic reviews and run up to the current date. The databases to be used will be the same as for point 3.
4) A grey literature scope using Grey Guide, Google Scholar and Researchgate. The timeline for the search will be the same as at points 1-3.
5) A grey data scope using a UK HD charity affiliated online network. In line with the agreed ethics approval and to reinforce anonymity but not abandon the replicability and therefore rigour of the review, the criteria for this element of the review will not be published. Readers of the review may request the inclusion/exclusion criteria and key words for the grey data search by writing to the author. Inclusion criteria will use similar parameters as per the scoping review for social functioning (see Table 2) i.e. comment on relationships.

Table 2: Summary of eligibility and inclusion/exclusion criteria<br><br>*Inclusion*<br><br>All elements of the review will consider sources of English language evidence which report on adults who are over the age of 18 who are gene positive, presymptomatic or have manifest HD.<br><br>Social cognitive reviews will be defined as: reviews which has used a systematic review approach and included all quantitative and mixed methods studies which have a comparator group and have the primary outcome using tests of social cognition in people with HD (i.e. tests of emotional recognition and/or tests of TOM)

For peer review only

The social cognitive review date search will start from 2003 which marks the earliest known social cognitive review by Elamin et al. This will be the start date for all elements of the total review. The final date for all elements of the total review will be up to and including June 2023

The updated social cognition scoping section will use the latter parameters for inclusion (quantitative, mixed methods, comparator group and social cognitive tests)

The scoping review for social functioning and behaviour will use the definition of social functioning as defined by Kennedy and Adolphs (see earlier comments)

The scoping review for social functioning and behaviour will include any qualitative, mixed or quantitative paper which comments on functioning in people with HD which takes place in a social context and that comments on social interaction or activity which has social consequences.

Any disagreement on inclusion will be through consultation between the primary author and supervisor HR

Search terms and key words can be found at Table 1. These were formed following a review of thesaurus terms and abstracts using two major databases (Medline and Web of Science) using the combination of words – social cognition AND Huntington*

Exclusion

As the protocol forms part of a PhD project, resources and timelines will limit the scope of the literature search and for this reason non-English language evidence will be excluded.

Data extraction and data summary

Qualitative, quantitative, and mixed method studies:

In line with the recommended PRISMA guidelines (30) particularly to show the conduct (and therefore rigour) of a review, data relevant to the aims of the review will be extracted from the data sources and 'charted' using an extraction form to summarise the sources' main findings. Whilst this is not a systematic review and therefore methodological quality of the sources is not the main aim, quality cannot be disregarded and in order to optimise the organisation of the synthesis (and thus quality) the Critical Appraisal Skills Programme (CASP) tool forms matched to the method of the paper (i.e. qualitative) will be used as the extraction forms (36).

Grey literature:

Review of the grey literature will use the AACODS (Authority, Accuracy, Coverage, Objectivity, Date and Significance) checklist designed by Jess Tyndall of Flinders University in 2010 and accepted as the baseline of good review practice (32).

Analysis and synthesis

As Popay (28) notes, in a review, analysis and synthesis co-exist in that evidence synthesis involves some form of analysis e.g. the finding of common themes across studies or the "interpretation of results from multiple studies, with the aim of producing new knowledge/findings".

Popay goes on to state that these different types of synthesis can be located along a continuum from quantitative approaches to qualitative approaches and as recommended in best practice literature

For peer review only

the narrative synthesis is the bridge between all of these with other synthesis approaches used in an iterative way depending on the heterogeneity of research designs and findings.

Therefore - after the data extraction, the data from each phase of the search strategy will be entered into their own matrix (using Excel) which will allow for the collation and therefore review of key themes. This framework method which comes from the family of content and thematic analysis methods (37) will lead to the production of the following -

1) An umbrella scoping review of social cognition will be presented through a textual description (alongside a tabular form) in clustered groupings (illustrating types of studies) with relationships between and across studies in a narrative and visual form.
2) A scoping review of social behaviour and functioning which will include the qualitative and grey literature as well as the grey data will also be presented in a tabular form with the main concepts (and relationships) described in a narrative and visual form.
3) A final synthesis will be in a narrative form of key points but also illustrated with a visual concepts map (38).

Public and service user involvement

This protocol is part of a wider PhD study on social cognition in HD using ethnography as a method. As the engagement of the HD community was of primary importance, the PPI group of the HDA was approached to comment on the method to see if it would be possible and acceptable but also whether the outcomes would be of value to them.

Ethics and dissemination

The PhD study which forms the background to this protocol has received ethical approval from the University of Birmingham's (UOB) STEM ERB with a further addendum specific to this protocol also approved for the internet mediated search of grey data.

The HD community will continue to be involved throughout the life cycle of the study with regular updates at HDA family events and participants will receive personal updates and summaries as requested. The final study will form a standalone piece on the HDA's website.

Discussion

Social cognition is a set of fundamental skills which are thought to enable successful social interaction. People with HD are known to have both problems with the brain structures associated with social cognition and when tested show this. The tests often take place in controlled settings. HD scientists and neuroscientists want to know more about what happens with social cognition in real life.

This protocol represents the plan for the weaving together of the information we already know from controlled settings alongside of additional observations from the scientific literature on socially related activity in HD as well as evidence from grey literature and grey data. This is intended to form a holistic map of the state of our knowledge of social cognition in HD so that gaps in that knowledge can be targeted; but also, that the knowledge from the review can inform clinical practice.

Author affiliations

1. Institute of Applied Heath Research, University of Birmingham, UK
2. Neuropsychiatry, Department of Clinical & Experimental Medicine, University of Birmingham, UK

For peer review only

3. West Midlands Huntington's Disease Service, Birmingham and Solihull Mental Health Foundation Trust, Birmingham, UK

Contributors: The final version was read and approved by all authors (AF, AL, SG and HR)

Funding: The protocol has no associated funding

Competing interests: AF, AL, SG and HR have no conflicting interests to declare

Correspondence to: alf944@bham.ac.uk

1. Henry JD. Clinical assessment of social cognitive function in neurological disorders. Nature Reviews Neurology. 2016;12(1):28-40.
2. Eddy CM, Rickards HE. Theory of mind can be impaired prior to motor onset in Huntington's disease. Neuropsychology. 2015;29(5):792.
3. Henley SM, Novak MJ, Frost C, King J, Tabrizi SJ, Warren JD. Emotion recognition in Huntington's disease: a systematic review. Neuroscience & Biobehavioral Reviews. 2012;36(1):237-53.
4. Bora E, Velakoulis D, Walterfang M. Social cognition in Huntington's disease: A meta-analysis. Behav Brain Res. 2016;297:131-40.
5. Kennedy DP, Adolphs R. The social brain in psychiatric and neurological disorders. Trends Cogn Sci. 2012;16(11):559-72.
6. Hendel RK, Hellem MN, Larsen IU, Vinther-Jensen T, Hjermind LE, Nielsen JE, et al. Impairments of social cognition significantly predict the progression of functional decline in Huntington's disease: A 6-year follow-up study. Applied Neuropsychology: Adult. 2022:1-10.
7. Mason SL, Schaepers M, Barker RA. Problems with Social Cognition and Decision-Making in Huntington's Disease: Why Is it Important? Brain Sciences. 2021;11(7):838.
8. Aubeeluck A. Huntington's disease. Part 3: family aspects of HD. British Journal of Nursing. 2008;17(5):328-32.
9. Vamos M, Hambridge J, Edwards M, Conaghan J. The impact of Huntington's disease on family life. Psychosomatics. 2007;48(5):400-4.
10. Brossman B, Williams JK, Downing N, Mills JA, Paulsen JS, Investigators P-H, et al. Development of the Huntington disease work function scale. Journal of occupational and environmental medicine/American College of Occupational and Environmental Medicine. 2012;54(10):1300.
11. Downing NR, Williams JK, Paulsen JS. Couples' attributions for work function changes in prodromal Huntington disease. Journal of genetic counseling. 2010;19(4):343-52.
12. Fisher F, Andrews S, Churchyard A, Mathers S. Home or Residential Care? The Role of Behavioral and Psychosocial Factors in Determining Discharge Outcomes for Inpatients with Huntington's Disease. Journal of Huntington's disease. 2012;1(2):187-93.
13. Stout JC, Glikmann-Johnston Y, Andrews SC. Cognitive assessment strategies in Huntington's disease research. Journal of neuroscience methods. 2016;265:19-24.
14. Hadley LV, Naylor G, Hamilton AFdC. A review of theories and methods in the science of face-to-face social interaction. Nature Reviews Psychology. 2022;1(1):42-54.
15. Arksey H, O'Malley L. Scoping studies: towards a methodological framework. International journal of social research methodology. 2005;8(1):19-32.
16. Munn Z, Peters MD, Stern C, Tufanaru C, McArthur A, Aromataris E. Systematic review or scoping review? Guidance for authors when choosing between a systematic or scoping review approach. BMC medical research methodology. 2018;18(1):1-7.

For peer review only

17. Simpson J, Dale M, Theed R, Gunn S, Zarotti N, Eccles FJ. Validity of irritability in Huntington's disease: A scoping review. Cortex. 2019;120:353-74.
18. Mahmood S, Law S, Bombard Y. "I have to start learning how to live with becoming sick": A scoping review of the lived experiences of people with Huntington's disease. Clinical Genetics. 2022;101(1):3-19.
19. Gibson JS, Springer K. Social Withdrawal in Huntington's Disease: A Scoping Review. Journal of Huntington's Disease. 2022;11:17-24.
20. Elamin M, Pender N, Hardiman O, Abrahams S. Social cognition in neurodegenerative disorders: a systematic review. Journal of Neurology, Neurosurgery & Psychiatry. 2012;83(11):1071-9.
21. Hoffecker L. Grey Literature Searching for Systematic Reviews in the Health Sciences. The Serials Librarian. 2020;79(3-4):252-60.
22. Pope C. Synthesizing qualitative and quantitative health evidence : a guide to methods / Catherine Pope, Nicholas Mays and Jennie Popay. Mays N, Popay J, editors: Maidenhead, England ; New York, NY : Open University Press, McGraw Hill Education, 2007.; 2007.
23. Paez A. Gray literature: An important resource in systematic reviews. Journal of Evidence-Based Medicine. 2017;10(3):233-40.
24. Adams J, Hillier-Brown FC, Moore HJ, Lake AA, Araujo-Soares V, White M, et al. Searching and synthesising 'grey literature'and 'grey information'in public health: critical reflections on three case studies. Systematic reviews. 2016;5(1):1-11.
25. Coulson NS, Buchanan H, Aubeeluck A. Social support in cyberspace: a content analysis of communication within a Huntington's disease online support group. Patient education and counseling. 2007;68(2):173-8.
26. Booth A, Sutton A, Clowes M, Martyn-St James M. Systematic approaches to a successful literature review. 2021.
27. Lisy K, Porritt K. Narrative synthesis: considerations and challenges. JBI Evidence Implementation. 2016;14(4):201.
28. Popay J, Roberts H, Sowden A, Petticrew M, Arai L, Rodgers M, et al. Guidance on the conduct of narrative synthesis in systematic reviews. A product from the ESRC methods programme Version. 2006;1(1):b92.
29. Aromataris E, Fernandez R, Godfrey CM, Holly C, Khalil H, Tungpunkom P. Summarizing systematic reviews: methodological development, conduct and reporting of an umbrella review approach. JBI Evidence Implementation. 2015;13(3):132-40.
30. Tricco AC, Lillie E, Zarin W, O'Brien KK, Colquhoun H, Levac D, et al. PRISMA extension for scoping reviews (PRISMA-ScR): checklist and explanation. Annals of internal medicine. 2018;169(7):467-73.
31. Stern C, Lizarondo L, Carrier J, Godfrey C, Rieger K, Salmond S, et al. Methodological guidance for the conduct of mixed methods systematic reviews. JBI evidence synthesis. 2020;18(10):2108-18.
32. Chang S. Health Libraries Inc. presentation:'Health librarians and grey literature-what do we need to know?'. Health Inform. 2014;23(2):45-6.
33. Savić D. When is 'grey'too 'grey'?-A case of grey data. The Grey Journal. 2019;15(2):71-6.
34. Borgman CL. Open data, grey data, and stewardship: Universities at the privacy frontier. Berkeley Technology Law Journal. 2018;33(2):365-412.
35. Hewson C. British Psychological Society - Ethics Guidelines for Internet Mediated Research www.bps.org.uk2021 [Available from: https://www.bps.org.uk/sites/www.bps.org.uk/files/Policy/Policy%20-%20Files/Ethics%20Guidelines%20for%20Internet-mediated%20Research.pdf.
36. Long HA, French DP, Brooks JM. Optimising the value of the critical appraisal skills programme (CASP) tool for quality appraisal in qualitative evidence synthesis. Research Methods in Medicine & Health Sciences. 2020;1(1):31-42.

For peer review only

37. Hughes SE, Aiyegbusi OL, Lasserson DS, Collis P, Rivera SC, McMullan C, et al. Protocol for a scoping review exploring the use of patient-reported outcomes in adult social care. BMJ open. 2021;11(4):e045206.
38. Ligita T, Nurjannah I, Wicking K, Harvey N, Francis K. From textual to visual: the use of concept mapping as an analytical tool in a grounded theory study. Qualitative Research. 2022;22(1):126-42.

For peer review only

Search strategy

Database	Stage	Keywords	Limits / other details
Embase / Ovid Medline (R) / Psychinfo	1. The umbrella scoping review of pre-existing systematic reviews	Huntington* AND (Social cogniti* OR Social function OR Social behaviour OR Social interaction OR Social judgement OR Social psychology OR Social understanding OR Social skills OR Social competence OR Social communication OR Social decision making OR Social withdrawal OR avoidance OR disengage)	Humans / English Language / Review articles / 2003 to current (June 2023)<br><br>Reference lists of articles which are clear **review articles** will be scanned for additional reviews related to the key words and these included in the scope if they meet the inclusion criteria
Web of Science	Stage 1 – as above	Huntingtons* Social* Cog*	Limits as previously applied
Pubmed	Stage 1 – as above	Huntington* AND social AND cognition	Defaulted to 2004 to current day<br><br>No other limits applied but searching for titles/abstracts with clear review article hallmarks
Scopus	Stage 1 – as above	Huntington* AND social AND cognition	Limits as previously applied + review articles only
Cinahl	Stage 1 – as above	Social cognition AND Huntingtons disease	Limits as previously applied
Embase / Ovid Medline (R) / Psychinfo	2. A further (non-umbrella) scoping review of quantitative and mixed methods literature on social cognition, social behaviour, and social functioning to the current date	Huntington* AND (Social cogniti* OR Social function OR Social behaviour OR Social interaction OR Social judgement OR Social psychology OR Social understanding OR Social skills OR Social competence OR Social communication	Humans / English Language / 2003 to current (June 2023)<br><br>Due to there being a maximum of key words (which differs in each database), separate searches will be undertaken for different concepts i.e. social cognitive processes such as

For peer review only

		OR Social decision making OR<br>Social withdrawal OR avoidance OR disengage)	emotional recognition, or social functioning which will include work<br><br>These will be then be combined if the parameters of the database allow
Embase / Ovid Medline (R) / Psychinfo<br><br>Web of Science<br><br>Pubmed<br><br>Scopus<br><br>Cinahl	**2.** A further (non-umbrella) scoping review of quantitative and mixed methods literature on social cognition, social behaviour, and social functioning	Huntington* AND Non-verbal communication<br><br>Huntington* AND Body language<br><br>Huntington* AND Facial expression<br><br>Huntington* AND Recognition<br><br>Huntington* AND (Theory of mind OR TOM OR simulation theory)<br><br>Huntington* AND (Empath* OR empath* communication)<br><br>Huntington* AND (Mentalizing OR mental state)<br><br>Huntington* AND Emotion*<br><br>Huntington* AND (Inference OR Intuition)	Reflecting social cognitive processes
Embase / Ovid Medline (R) / Psychinfo<br><br>Web of Science<br><br>Pubmed	**2.** A further (non-umbrella) scoping review of quantitative and mixed methods literature on social cognition, social behaviour, and social functioning	Huntington* AND (Relationships OR interpersonal relationships)<br>Huntington* AND (Social networks OR social connections)	Reflecting social behaviour and functioning

For peer review only

Scopus<br><br>Cinahl		Huntington* AND Work<br><br>Huntington* AND Sex*<br><br>Huntington* AND (Function OR skills)	
Embase / Ovid Medline (R) / Psychinfo<br><br>Web of Science<br><br>Pubmed<br><br>Scopus<br><br>Cinahl	**3.** A scoping review of the qualitative literature concerning social cognition, social behaviour and social functioning.	Huntington* AND Non-verbal communication<br><br>Huntington* AND Body language<br><br>Huntington* AND Facial expression<br><br>Huntington* AND Recognition<br><br>Huntington* AND (Theory of mind OR TOM OR simulation theory)<br><br>Huntington* AND (Empath* OR empath* communication)<br><br>Huntington* AND (Mentalizing OR mental state)<br><br>Huntington* AND Emotion*<br><br>Huntington* AND (Inference OR Intuition)	Reflecting social cognitive processes
Embase / Ovid Medline (R) / Psychinfo<br><br>Web of Science<br><br>Pubmed	**3.** A scoping review of the qualitative literature concerning social cognition, social behaviour and social functioning.	Huntington* AND (Relationships OR interpersonal relationships)<br><br>Huntington* AND (Social networks OR social connections)	Reflecting social behaviour and functioning

For peer review only

Scopus<br><br>Cinahl		Huntington* AND Work<br><br>Huntington* AND Sex*<br><br>Huntington* AND (Function OR skills)	
Open Grey (EU and UK version)	**4.** A grey literature scope	Huntingtons disease	Neither databases allow for limits
Google Scholar	A grey literature scope	Huntington* AND (Relationships OR interpersonal relationships)<br><br>Huntington* AND (Social networks OR social connections)<br><br>Huntington* AND Work<br><br>Huntington* AND Sex*<br><br>Huntington* AND (Function OR skills)	Limits 2003 – 2023
Researchgate	A grey literature scope	Huntingtons disease	Author to scan first 25 pages on results for all literature relevant to the concepts of social cognition OR social functioning

Stage 5 - Please apply to the author for the search details of the message board as per the protocol published

For peer review only

Archived at the Flinders Academic Commons:
http://dspace.flinders.edu.au/dspace/

The **AACODS** checklist is designed to enable evaluation and critical appraisal of grey literature.

The Fourth International Conference on Grey Literature held in Washington, DC, in October 1999 **defined grey literature** as: "that which is produced on all levels of government, academics, business and industry in print and electronic formats, but which is not controlled by commercial publishers."
Grey literature includes theses or dissertations (reviewed by examiners who are subject specialists); conference papers (often peer-reviewed or presented by those with specialist knowledge) and various types of reports from those working in the field. All of these fall into the **"expert opinion"**
Critical appraisal is "the process of carefully and systematically examining research to judge its trustworthiness, and its relevance and value in a particular context" (Burls 2009)

Grey (unpublished) studies and RCTs should be appraised using the same tools as their black (published) counterparts.

**AACODS**		**YES**	**NO**	**?**
**Authority**	Identifying who is responsible for the intellectual content.<br><br>**Individual author:**<br>• Associated with a reputable organisation?<br>• Professional qualifications or considerable experience?<br>• Produced/published other work (grey/black) in the field?<br>• Recognised expert, identified in other sources?<br>• Cited by others? (use Google Scholar as a quick check)<br>• Higher degree student under "expert" supervision?<br><br>**Organisation or group**:<br>• Is the organisation reputable? (e.g. W.H.O)<br>• Is the organisation an authority in the field?<br><br>**In all cases:**<br>• Does the item have a detailed reference list or bibliography?			
**Accuracy**	• Does the item have a clearly stated aim or brief?<br>• Is so, is this met?<br>• Does it have a stated methodology?<br>• If so, is it adhered to?<br>• Has it been peer-reviewed?<br>• Has it been edited by a reputable authority?<br>• Supported by authoritative, documented references or credible sources?<br>• Is it representative of work in the field?<br>• If No, is it a valid counterbalance?<br>• Is any data collection explicit and appropriate for the research?<br>• If item is secondary material (e.g. a policy brief of a technical report) refer to<br>• the original. Is it an accurate, unbiased interpretation or analysis?			

For peer review only

Archived at the Flinders Academic Commons:
http://dspace.flinders.edu.au/dspace/

**Coverage**	All items have parameters which define their content coverage. These limits might mean that a work refers to a particular population group, or that it excluded certain types of publication. A report could be designed to answer a particular question, or be based on statistics from a particular survey.<br><br>• Are any limits clearly stated?			
**Objectivity**	It is important to identify bias, particularly if it is unstated or unacknowledged.<br><br>• Opinion, expert or otherwise, is still opinion: is the author's standpoint clear?<br>• Does the work seem to be balanced in presentation?			
**Date**	For the item to inform your research, it needs to have a date that confirms relevance<br><br>• Does the item have a clearly stated date related to content? No easily discernible date is a strong concern.<br>• If no date is given, but can be closely ascertained, is there a valid reason for its absence?<br>• Check the bibliography: have key contemporary material been included?			
**Significance**	This is a value judgment of the item, in the context of the relevant research area<br><br>• Is the item meaningful? (this incorporates feasibility, utility and relevance)<br>• Does it add context?<br>• Does it enrich or add something unique to the research?<br>• Does it strengthen or refute a current position?<br>• Would the research area be lesser without it?<br>• Is it integral, representative, typical?<br>• Does it have impact? (in the sense of influencing the work or behaviour of others)			

Burls, A. 2009, *What is critical appraisal*?, Bandolier, viewed 4 November 2009. <http://www.medicine.ox.ac.uk/bandolier/painres/download/whatis/What_is_critical_appraisal.pdf>

Jess Tyndall,
Flinders University,
Nov 2010

For peer review only
